# The structure of *Lactococcus lactis* thioredoxin reductase reveals molecular features of photo-oxidative damage

**DOI:** 10.1038/srep46282

**Published:** 2017-04-11

**Authors:** Nicklas Skjoldager, Maria Blanner Bang, Martin Rykær, Olof Björnberg, Michael J. Davies, Birte Svensson, Pernille Harris, Per Hägglund

**Affiliations:** 1 Department of Biotechnology and Biomedicine, Technical University of Denmark, DK-2800 Kgs. Lyngby, Denmark; 2 Department of Chemistry, Technical University of Denmark; 3 Department of Biomedical Sciences, University of Copenhagen.

## Abstract

The NADPH-dependent homodimeric flavoenzyme thioredoxin reductase (TrxR) provides reducing equivalents to thioredoxin, a key regulator of various cellular redox processes. Crystal structures of photo-inactivated thioredoxin reductase (TrxR) from the Gram-positive bacterium *Lactococcus lactis* have been determined. These structures reveal novel molecular features that provide further insight into the mechanisms behind the sensitivity of this enzyme toward visible light. We propose that a pocket on the *si*-face of the isoalloxazine ring accommodates oxygen that reacts with photo-excited FAD generating superoxide and a flavin radical that oxidize the isoalloxazine ring C7α methyl group and a nearby tyrosine residue. This tyrosine and key residues surrounding the oxygen pocket are conserved in enzymes from related bacteria, including pathogens such as *Staphylococcus aureus*. Photo-sensitivity may thus be a widespread feature among bacterial TrxR with the described characteristics, which affords applications in clinical photo-therapy of drug-resistant bacteria.

The protein disulfide reductase thioredoxin (Trx) maintains thiol groups in a reduced form through thiol-disulfide exchange reactions involving a redox-active WCXXC motif. Trx also provides reducing equivalents to enzymes like ribonucleotide reductase, peroxiredoxins and methionine sulfoxide reductase^1^. The recycling of Trx is catalysed by the homodimeric flavoprotein thioredoxin reductase (TrxR) that utilizes electrons from NADPH which are shuttled through a tightly bound FAD co-enzyme and a redox active dithiol motif. The TrxR monomers in bacteria, fungi and plants are about 35 kDa and composed of an NADPH- and an FAD-binding domain. By contrast, the monomers of TrxR from mammals, dipteran insects and protozoan parasites are larger (ca 50–55 kDa) and harbor an interface domain and a C-terminal flexible extension with an additional redox-active motif. In mammalian enzymes this motif contains a selenocysteine residue, while in e.g. *Drosophila melanogaster* this is replaced by a cysteine apparently without altering the catalytic efficiency^2^. Based on pioneering work on the three-dimensional structure and biophysical characteristics of *Escherichia coli* TrxR (EcTrxR) it was proposed that this bacterial enzyme undergoes a major molecular re-arrangement from the so-called Flavin Oxidizing (FO) conformation to the Flavin Reducing (FR) conformation in order to complete a catalytic cycle^3^,^4^,^5^. The FR conformation structure of EcTrxR in complex with Trx indeed confirmed a 66° rotation of the NADPH domain relative to the FAD domain, which relocates NADPH for hydride transfer to FAD and positions the TrxR CXXC motif in proximity of Trx^6^. Mammalian TrxR does not undergo such a conformational change during catalysis and the reducing equivalents delivered on the *re*-face of FAD are instead directly transferred to a redox-active CVNVGC motif positioned on the *si*-face^7^,^8^. Subsequently, the electrons are transferred to the C-terminal redox center, in turn reducing the CXXC motif in Trx. These mechanistic differences make TrxR an attractive target for antimicrobial drugs and a range of inhibitors have been explored for this purpose^9^,^10^.

The physiological importance of Trx and TrxR are highly dependent on the lifestyle of the organisms as well as access to alternative redox control systems and antioxidant enzymes. For example in *E. coli* and other glutathione-producing bacteria, Trx is complemented by glutaredoxin as an electron donor to ribonucleotide reductase, whereas glutathione-negative bacteria such as *Bacillus subtilis* and *Staphylococcus aureus* lack this pathway^11^,^12^. The function of Trx as an electron donor to peroxiredoxins is particularly important in catalase-negative bacteria that rely on thiol-dependent peroxidases to remove damaging peroxides. Many lactic acid bacteria (LAB) lack both glutathione and catalase, suggesting that Trx plays a particularly central role in protection against oxidative stress in these bacteria. *Lactococcus lactis*, an industrially important LAB model bacterium, contains a TrxR that recycles two Trx (TrxA and TrxD) as well as NrdH, a glutaredoxin-like protein providing electrons to ribonucleotide reductase class Ib^13^. Additionally, it was recently discovered that reducing equivalents for ribonucleotide reduction can be provided in a TrxR-independent manner through flavoredoxin^14^. This may explain why knock-out mutants lacking either TrxR or Trx are viable even under oxidative stress conditions^15^. While investigating the biochemical properties of *L. lactis* TrxR (LlTrxR) we discovered that the enzyme is susceptible to photo-inactivation by visible light in an oxygen-dependent manner^16^. This inactivation coincided with a shift in the absorbance spectrum of the tightly bound FAD co-enzyme and oxidation of an isoalloxazine methyl group. To characterize the consequences of this photo-oxidation process in greater detail the three-dimensional structure of inactivated LlTrxR is here solved by X-ray crystallography. We propose possible mechanisms that account for the observed oxidative damage of the enzyme.

## Results

### TrxR from *L. lactis* is sensitive to photo-inactivation

**Figure 1:**
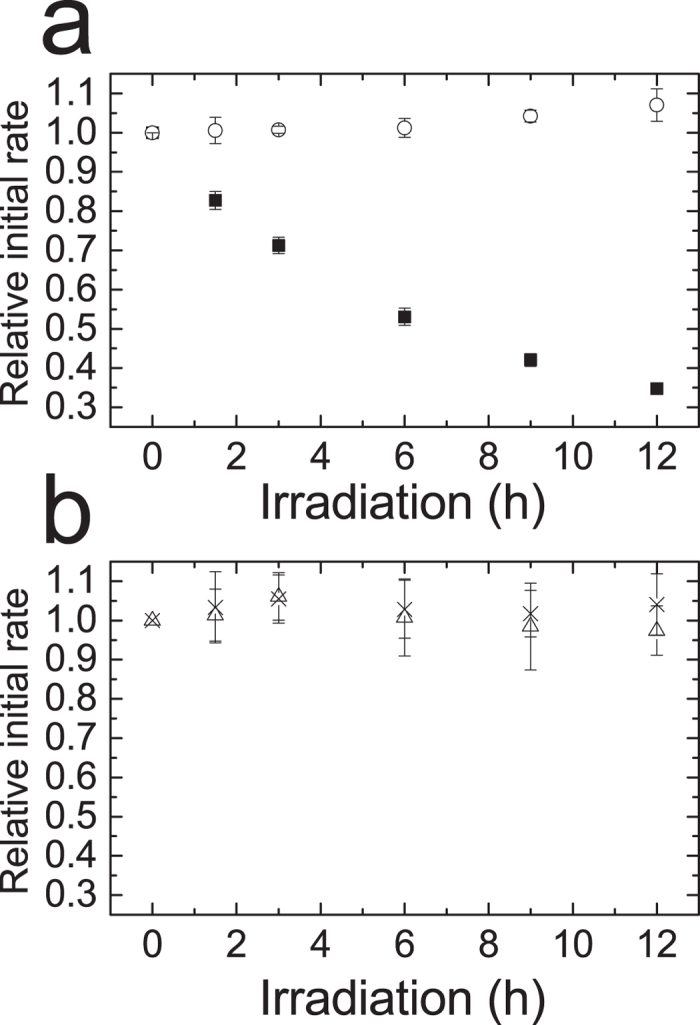
Inactivation of native TrxR in cell extracts. Activity of TrxR quantified by DTNB reduction, was assayed in light exposed cell extracts from *L. lactis* (**a,** ■) and *E. coli* (**b,** △) by adding 0.2 mM DTNB, 0.2 mM NADPH and 5 μM of the respective Trx. “No Light” controls (○ (**a**) and × (**b**)) were samples wrapped in foil; these exhibited no loss of activity. A similar relative change in activity was obtained in assays without addition of Trx (data not shown). All experiments were performed with three biological replicates.

We have previously shown that recombinant TrxR from *L. lactis* is inactivated by visible light *in vitro*, while the corresponding enzyme from *E. coli* is more resistant to photo-inactivation under these conditions^16^. In order to evaluate the sensitivity of native TrxR in an environment resembling the conditions *in vivo*, cell extracts of mid-late exponential phase cultures of *L. lactis* and *E. coli* K-12 were subjected to irradiation over a period of 12 h and TrxR activity was measured at different time points by use of a coupled assay with Trx, applying DTNB as the final electron acceptor. A marked decrease in TrxR activity was observed in the *L. lactis* cell extracts over the course of irradiation, while the activity in the *E. coli* cell extract was essentially unchanged (Fig. 1). After 12 h a ~65% drop in activity was observed in *L. lactis* cell extract. This apparent slower rate of inactivation of native LlTrxR compared to the recombinant enzyme may be due to light-quenching by endogenous chromophores in the cell extracts.

### Overall structure of LlTrxR

**Figure 2:**
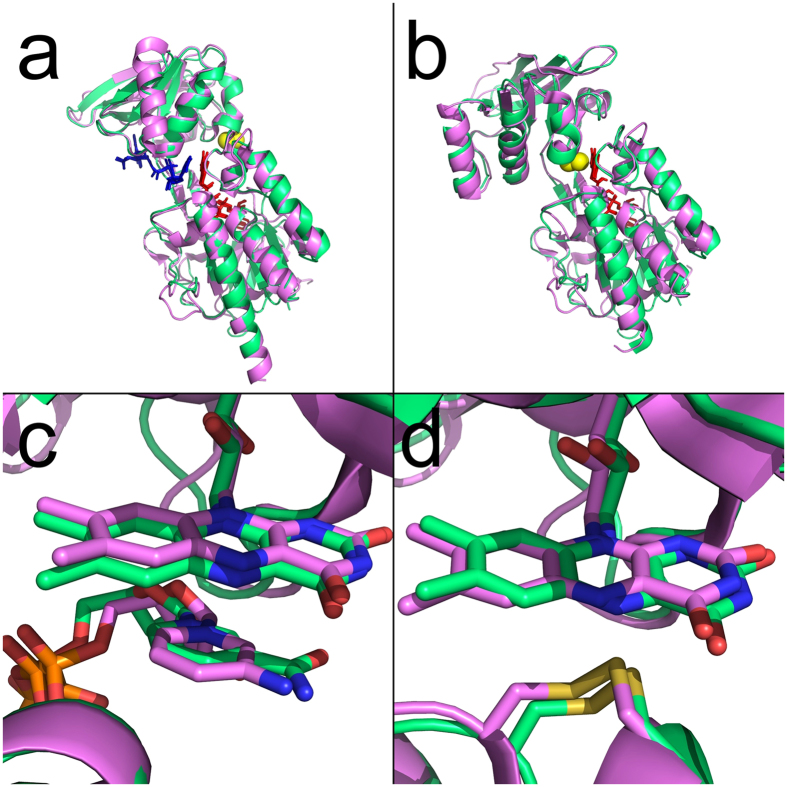
Overall structure of LlTrxR. Superposition of LlTrxR (green) and EcTrxR (violet) in FR (**a**) and FO (**b**) conformations with RMSD values of 0.426 and 1.137 Å, respectively. The FAD and NADPH domains are connected by two anti-parallel β-strands (amino acid residues 113–115 and 240–242). In both the FR and FO conformations the FAD (red sticks), as well as NADP^+^ and the NADP^+^ analog AADP^+^ (blue sticks in the FR conformation) align well as indicated. The active site disulfide bond is marked as yellow spheres. Close-up of the active site of LlTrxR (green) and EcTrxR (violet) in the FR (**c**) and FO (**d**) conformations, indicating that FAD and NADP^+^/AADP^+^ are oriented identically in the two enzymes. PDB IDs for the EcTrxR structures are 1F6M and 1TDE for FR and FO conformation, respectively.

The overall structure of LlTrxR is similar to other low molecular weight (LMW) TrxRs^3^,^17^ with a dimeric organization in which each monomer is composed of NADPH and FAD binding domains (Fig. 2). The secondary structure of each subunit consists of 11 α-helices and 19 β-sheets. The FAD-domain consists of residues 1–112 and 243–308 and the NADPH-domain consists of residues 116–239. The LlTrxR structures were obtained mainly in the FR conformation (Fig. 2a,c), which was unexpected since all available structures of LMW TrxR from other organisms are in the FO conformation, except for the engineered complex between EcTrxR and EcTrx^6^. LlTrxR crystallized in the FO conformation (Fig. 2b,d) only under reducing conditions in the presence of DTT (Table 1). The propensity of LlTrxR to crystallize in the FR conformation may indicate that the enzyme is stabilized in this conformation. Superposition of LlTrxR C_α_-atoms with the structure of EcTrxR^3^,^6^, gives RMSD of 0.426 Å and 1.137 Å for the enzymes in FR (Fig. 2a,c) and FO conformation (Fig. 2b,d), respectively. The relative orientation of the structures thus matches perfectly with the *E. coli* enzyme and consolidates the catalytic model proposed by Waksman *et al*. (1994) where the NADPH domain rotates 66° relative to the FAD domain in order to switch between the FO and FR conformations in the catalytic cycle.

### Oxidation of the FAD co-enzyme and Tyr237 in photo-inactivated LlTrxR

**Figure 3:**
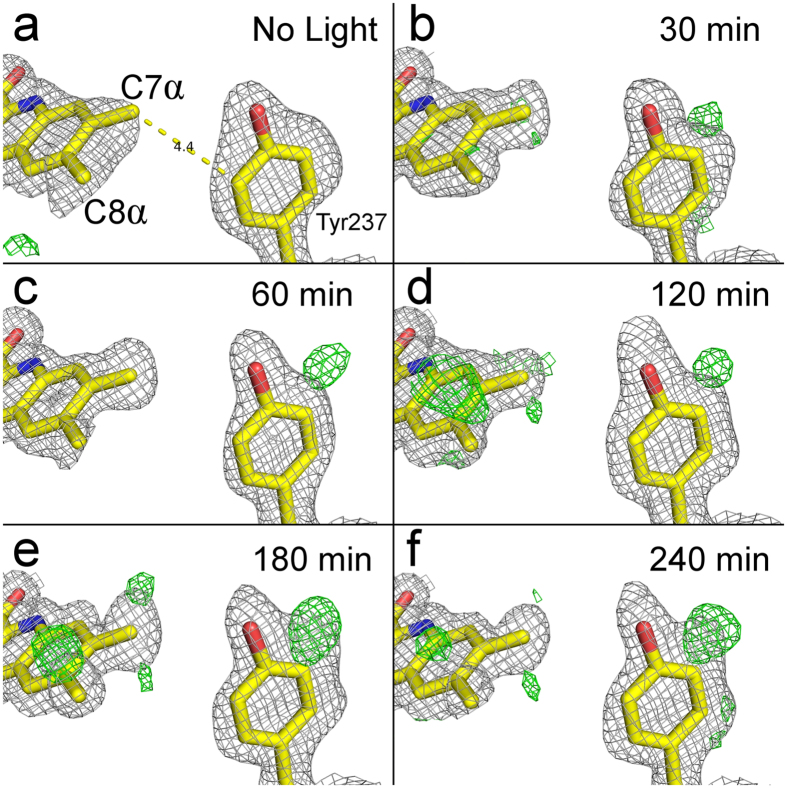
Oxidation of the isoalloxazine ring and Tyr237 in photo-inactivated LlTrxR. Increased electron density around the C7α methyl group of the isoalloxazine ring, and the meta-position of Tyr237 in LlTrxR develops over the course of irradiation (0–240 min). The *σ*_A_-weighted 2*F*_o_–*F*_c_ maps (grey) have been contoured at 1.0 *σ*. The difference *F*_o_–*F*_c_ maps (green/red) have been contoured at ± 3.0 *σ*, respectively. The C7α methyl group and the meta-position of Tyr237 develop increased difference densities mainly in the *σ*_A_-weighted 2*F*_o_–*F*_c_ maps and *F*_o_–*F*_c_ maps, respectively.

No apparent change in the overall structure of LlTrxR in the FR conformation was observed upon exposure to visible light. Close examination of the isoalloxazine ring however reveals increased electron densities around the C7α methyl group over the course of irradiation (Fig. 3a–f), even though the FAD co-enzyme in general has well-defined electron densities with B-factors typically in the range from 20–24 Å^2^. This observation is in accordance with our previous mass spectrometry data demonstrating formation of an aldehyde group in FAD extracted from light-inactivated LlTrxR, which was concluded to be confined to the C7α methyl group based on the spectrophotometric features^16^. Moreover, an increase in electron density at the meta-position (ortho to the hydroxyl group) of Tyr237, located 4.4 Å from C7α is observed as a function of light-exposure (Fig. 3a–f). This density most likely represents the formation of 3,4-dihydroxyphenylalanine (DOPA). Mass spectrometric analysis of proteolytic digests of photo-inactivated LlTxR indeed confirms a mass shift of 16 Da confined to Tyr237 (Supplementary Figure 1). Tyr237 is only close to the isoalloxazine ring when LlTrxR is in the FR conformation (Fig. 3a), suggesting that photo-oxidation reactions are primarily taking place while the enzyme is in this conformation. In the FO conformation, Tyr237 is distant from the flavin-binding site and another tyrosine (Tyr133) is in proximity to the isoalloxazine ring (4.9 Å from C7α), but no indications of oxidation of Tyr133 were observed in the irradiated structures of LlTrxR. Remarkably, Tyr237 appears to be conserved among most TrxR from Gram-positive bacteria in the phylum *Firmicutes*, but is replaced mainly by Ala or Phe in other organisms (Supplementary Figure 2).

### An oxygen pocket on the *si*-face of the isoalloxazine ring

**Figure 4:**
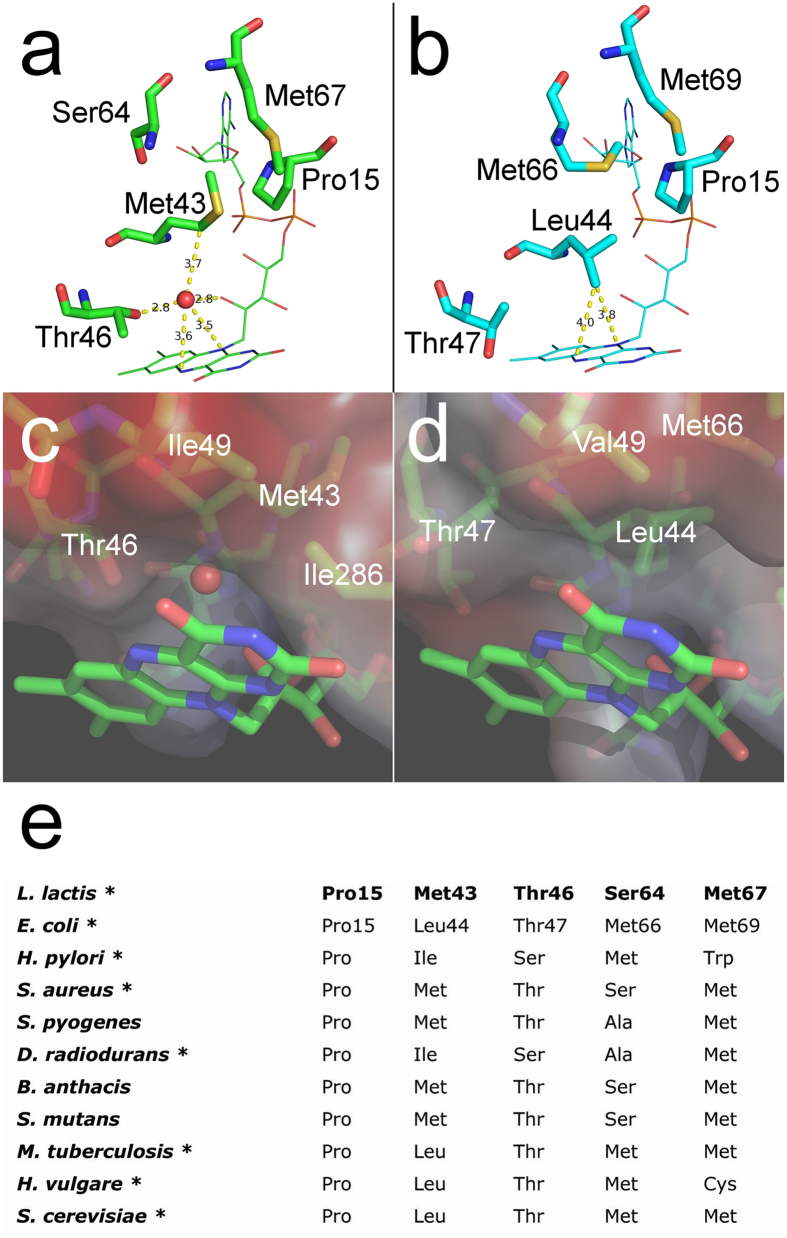
An oxygen pocket on the *si*-face of the FAD isoalloxazine ring. (**a**) LlTrxR has a pocket on the *si*-face of the isoalloxazine ring occupied by a water molecule (red sphere), which is in hydrogen bonding distance from the hydroxyl group of the Thr46 sidechain and the 2′-ribityl hydroxyl group of the FAD. Met43 bends towards Pro15 exposing a pocket which is occupied with a water molecule in all obtained structures of LlTrxR. (**b**) In EcTrxR (PDB 1F6M) Met43_LlTrxR_ is substituted with Leu44_EcTrxR_ that is centered at the *si*-face of the isoalloxazine ring and restricts access due to steric hindrance. In addition, Ser64_LlTrxR_ is replaced by the more bulky Met66_EcTrxR_ that hinders Leu44_EcTrxR_ from bending towards Pro15. Distances are given in Å and marked with dashed lines. (**c**) Electrostatic environment at the *si*-face of isoalloxazine in LlTrxR as viewed from the *re*-face showing the pocket where O_2_ can be accommodated. The pocket is confined by the π-electron cloud of the isoalloxazine, key hydrophobic residues Ile49, Ile286, Met43 and the methyl group of Thr46. (**d**) Corresponding electrostatic environment at the *si*-face of isoalloxazine in EcTrxR, where the *si*-face pocket is blocked by Leu44. (**e**) Alignment of selected residues that are positioned near the isoalloxazine *si*-face (sequences of structure determined TrxR are marked with an asterisk).

The observed oxygen- and triplet isoalloxazine-dependent photo-inactivation of LlTrxR^16^ suggests that molecular oxygen is positioned in the vicinity of the FAD and interacts with the photo-excited co-enzyme. A closer look at the isoalloxazine ring indeed reveals a pocket that potentially may accommodate molecular oxygen due to its hydrophobic character. Met43 positioned on the *si*-face bends away from the isoalloxazine towards Pro15 and the sidechains of Met18 and Met67 (Fig. 4a) and as a consequence a pocket on the *si*-face is formed. In the space between the β-carbon of Met43 and the isoalloxazine ring (7.2–7.4 Å distance to N10 and N5) a well-defined electron density appears, assigned to a water molecule perfectly centered above the central ring of the isoalloxazine. This water molecule is present in all structures listed in Table 1. The water is in hydrogen bond distance from the 2′-ribityl hydroxyl group (2.8 Å) and the alcohol group of Thr46 (2.8 Å), and the distance from the water to the N5 and N10 atom of the isoalloxazine is 3.5–3.6 Å (Fig. 4a). A closer look at the electrostatic environment on the *si*-face reveals several hydrophobic residues, most noteworthy are Ile286, Ile49, Leu63 and the β- and γ-carbons of Met43 that together with the π-electron cloud of the conjugated isoalloxazine ring system constitute an overall hydrophobic environment facilitating accommodation of O_2_ (Fig. 4c). In the presence of O_2_ at this location, it can be speculated that the Thr46 sidechain can flip so the methyl group points towards the O_2_, while the 2′-ribityl hydroxyl group can form a hydrogen bond with the 4′-ribityl hydroxyl group. These changes will further increase the hydrophobicity of the *si*-face, thus increasing the O_2_ affinity. The *re*-face of the isoalloxazine ring in the FO conformation is occupied by the Cys134-Cys137 disulfide while in the FR conformation it is surrounded by hydrophilic residues (Asp154, Glu158, Ser155 and Gln285). These features further underlines that the *si*-face open space is the most likely site of O_2_ accommodation and thus oxidant generation. The oxygen pocket described here is conserved in *Staphylococcus aureus* TrxR (SaTrxR), the most closely related structure-determined enzyme (53% sequence identity).

Even though several residues on the *si*-face of the isoxoalloxazine ring are conserved among TrxR (Fig. 4e) some subtle yet crucial differences in the local environment are observed. Met43_LlTrxR_ appears to be conserved among bacteria in the phylum *Firmicutes* (Supplementary Figure 2), but in EcTrxR this residue is substituted with Leu44_EcTrxR_ that is centered at the *si*-face of the isoalloxazine with a 3.8–4.0 Å distance from the δ-carbon to the N10 and N5 atoms (Fig. 4b). In addition, Ser64_LlTrxR_ is replaced by the more bulky Met66_EcTrxR_ that hinders Leu44_EcTrxR_ from bending towards Pro15. Leu44_EcTrxR_ thus blocks the pocket on the *si*-face of the isoalloxazine and restricts access of O_2_ (Fig. 4d). Noticeably, all available bacterial TrxR structures except those from *L. lactis* and *S. aureus* display Leu/Ile in similar positions as Leu44 in EcTrxR (Fig. 4e).

## Discussion

**Figure 5:**
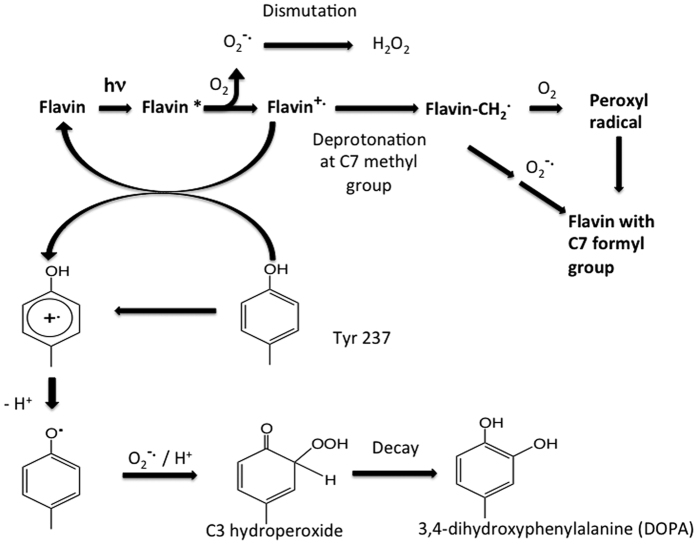
Proposed mechanisms for oxidation of Tyr237 and the FAD methyl group. Upon photo-induced excitation of FAD an electron is transferred to O_2_ generating superoxide. The one-electron deficient flavin cation radical can oxidize Tyr237 forming a radical-cation, which rapidly deprotonates to give Tyr phenoxyl radical. The Tyr phenoxyl radical is a target for superoxide and undergoes rapid reaction to give hydroperoxides at either C3 or C5. The resulting Tyr-OOH decays to give 3,4-dihydroxyphenylalanine (DOPA). The flavin radical-cation can upon deprotonation at C7α generate a primary C7α radical (R-CH_2_^.^). In the presence of O_2_ this would lead to formation of a peroxyl radical, while in the presence of O_2_^−.^ a hydroperoxide would be formed. Subsequent decay of the peroxyl radical via the Russell mechanism, or decomposition of the hydroperoxide, would yield an aldehyde.

Based on the structural information provided here we propose mechanisms that may account for the observed photo-inactivation of LlTrxR with the involvement of photo-excited FAD and O_2_ (Fig. 5). The initial step involves light absorption by the isoalloxazine ring and subsequent electron transfer from the ring to O_2_ with concomitant generation of superoxide radicals (O_2_^**−.**^) and an isoalloxazine radical-cation. Subsequent rapid, spontaneous dismutation of O_2_^**−.**^would give H_2_O_2_^18^, as observed in other flavin photo-oxidation reactions^19^. The isoalloxazine radical-cation may then undergo one of two processes: electron transfer with the nearby Tyr237 to regenerate the isoalloxazine and generate a Tyr237 radical-cation (which would be expected to rapidly deprotonate to give a Tyr phenoxyl radical due to the very low p*K*_a_ of these species), or deprotonation at one of the ring methyl groups. Such reactions are supported by literature data on the intramolecular photo-oxidation of target substrates by flavins subject to blue light, by electron transfer pathways^19^.

Tyr phenoxyl radicals undergo rapid reaction with O_2_^**−.**^/HOO. to give hydroperoxides, with these being formed at either C3/C5 or C1 due to the high spin density at these sites^20^,^21^. Such peroxides are unstable and undergo ready decay to the corresponding catechols, potentially accounting for the formation of 3,4-dihydroxyphenyalanine (DOPA) from Tyr237. An alternative route for decay of the isoalloxazine radical-cation involves deprotonation at a methyl group (a well-established decay pathway of radical-cations^22^,^23^) to give a primary alkyl radical (–CH_2_^.^). In the presence of O_2_ this would lead to formation of a peroxyl radical, while in the presence of O_2_^**−.**^a hydroperoxide would be formed. Subsequent decay of the peroxyl radical via the Russell mechanism^24^, or decomposition of the hydroperoxide^25^, would yield an aldehyde.

An alternative route to oxidation of the C7α methyl group may be direct hydrogen atom abstraction from the methyl group by the Tyr237 phenoxyl radical (i.e. Tyr237-O^.^ + flavin-CH_3_ → Tyr237-OH + flavin-CH_2_^.^ → formyl group) but such a pathway would be energetically less favorable due to the relative bond strengths of the C-H and O-H bonds. An alternative process involving formation of singlet oxygen (^1^O_2_) by the excited state isoalloxazine could also be envisaged, with this then undergoing decay to H_2_O_2_ or formation of an endoperoxide from the Tyr237 residue^26^. Subsequent decay of the unstable endoperoxide, potentially involving radical formation^26^, could account for the formation of DOPA from Tyr237 and oxidation of the C7α methyl group on the isoalloxazine ring to the formyl group.

The structure of LlTrxR also reveals an oxygen pocket at the *si*-face of the isoalloxazine ring, which is absent in EcTrxR. EcTrxR has previously been shown be highly photo-reactive and forms high yields of FAD semiquinones upon irradiation under anaerobic reaction conditions^27^. In light of the data obtained in the present study, it may therefore be suggested that the difference in photo-sensitivity between LlTrxR and EcTrxR is related to the accessibility of O_2_ in the active site, rather than the light-absorbing properties of the isoalloxazine ring *per se*. Besides its proposed role in photo-sensitivity the oxygen pocket might also have implications for the ability of these enzymes to reduce O_2._ We have reported that LlTrxR displays a ~10-fold increased rate of O_2_ reduction in the presence of NADPH as compared to EcTrxR^16^. LlTrxR thus shows a similar tendency to reduce O_2_ as the TrxR homologue AhpF, a dedicated reductase of the peroxide scavenger AhpC in eubacteria^28^,^29^. Interestingly, X-ray crystal structures of AhpF from *S. typhimurium* and *E. coli* (PDB 1HYU and 1FL2, respectively), also display a pocket on the *si*-face of the isoalloxazine and a tyrosine (Tyr344) located in the same position relative to the C7α as Tyr237_LlTrxR_. It can thus be hypothesized that AhpF also may be sensitive to photo-oxidation. However, it is important to emphasize that a direct comparison between reduction and photo-activation of O_2_ cannot be made since the molecular mechanisms and kinetic properties of these processes are different.

The novel molecular features of LlTrxR reported here provide insight into the photo-inactivation mechanism involving a protein-bound FAD acting as a photosensitizer that is highly relevant in a wider context. The conservation of Tyr237 and Met43 among TrxR from related Gram-positive bacteria suggests that photo-sensitivity may be widespread among these types of organisms. This opens up interesting perspectives in terms of clinical light treatment, e.g. blue light therapy of pathogenic and multi-drug resistant bacteria such as *S. aureus, Streptococcus pyogenes* and *Bacillus anthracis*, which all have TrxR predicted to harbour the described oxygen pocket.

## Methods

### Photo-inactivation of native TrxR in cell extracts

*Lactococcus lactis* subsp. *cremoris* (strain MG1363) was grown in SA medium^30^ containing 1% (w/v) glucose and 2 mg/L lipoic acid (GSAL medium). *E. coli* K-12 (strain MG1655) was grown in LB-medium. One liter synchronized cell culture (in biological triplicate) at exponential phase (OD_600_ 0.6–0.7) was placed on ice for 30 min and then centrifuged (4 °C). The cell pellet was resuspended in 0.9% NaCl and distributed in ten 2 mL screw-cap microcentrifuge tubes, centrifuged, and the pellets were stored at −20 °C until use. Proteins were extracted by adding 600 μL 100 mM potassium phosphate pH 7.5, 1 mM EDTA and 400 μL glass beads ≤106 μm (Sigma) to each tube and performing bead beating on a FastPrep FP120 homogenizer (Qbiogene), setting 6 for 30 s, followed by 1 min rest on ice. Bead beating was repeated 6 times followed by centrifugation for 15 min at 13,000 g. Benzonase (2 μL) was added to pooled supernatants (~4.5 mL) and incubated for 1 h at 21 °C. The protein concentration was determined (Bio-Rad Protein Assay, Life Science Research) using BSA as a standard and the samples were aliquoted and stored at −20 °C. Cell extracts were thawed, diluted 1:1 with 100 mM potassium phosphate pH 7.5, 1 mM EDTA and transferred to original 1.5 mL Eppendorf tubes with 6 × 3mm magnets, and placed on a stirrer in a cold room at 4 °C. The samples were irradiated with a 1225 Lumen (21 W) lamp at 15 cm distance as previously described^16^. Control samples wrapped in foil are referred to as “No Light”. After 0, 1.5, 3, 6, 9 and 12 h cell extracts were removed and stored at −20 °C for subsequent activity measurements. TrxR activity was assayed as 5,5′-dithiobis(2-nitrobenzoic acid) (DTNB) reduction monitored at 412 nm in 150 μL format with 100 mM potassium phosphate, pH 7.5, 2 mM EDTA, 0.20 mM DTNB and 0.20 mM NADPH. For *L. lactis* 30 μL of undiluted cell extract was used in the assay while the *E. coli* cell extracts were diluted 3 times in 100 mM potassium phosphate, pH 7.5, 1 mM EDTA before being added to the assay mixture. The activity of the cell extracts was measured directly or after addition of their respective recombinant thioredoxins (5 μM final concentration), LlTrxA and EcTrx1^13^. The addition of recombinant thioredoxin in the assay boosted the signal ~4 and ~2.3 times for *L. lactis* and *E. coli*, respectively. The initial activities were determined in at least technical triplicates for each biological replicate.

### Photo-inactivation of recombinant TrxR

Production and purification of recombinant His-tagged *L. lactis* and *E. coli* TrxR and Trx and subsequent light inactivation of TrxR were performed essentially as described previously^16^. For crystallization trials 50 μM LlTrxR was irradiated for 30, 60, 120, 180, and 240 min (with remaining relative initial activities of ~73, 27, 26, 15 and 10%, respectively) and subsequently loaded on a HiLoad Superdex 75 gel filtration column (prep grade, 16/60; GE Healthcare) using 10 mM HEPES, 200 mM NaCl, 2 mM Na-EDTA, pH 7.0 as eluent. Fractions that appeared homogeneous according to the chromatogram and SDS-PAGE were pooled, dialyzed (6−8 kDa MWCO, Spectra/Por 1) against 10 mM HEPES, 2 mM Na-EDTA, pH7.0, and concentrated (Amicon Ultra centrifugal filter unit, 10 kDa MWCO) to 8–10 mg/mL as assessed from absorbance (ɛ_456_ = 11,300 M^−1^cm^−1^)^31^.

### Crystallization and data collection of LlTrxR

Crystals obtained in FR conformation: LlTrxR (2 μL, 8–10 mg/mL) was mixed with 2 μL reservoir buffer and co-crystallized with 1 μL 25 mM NADP^+^ (Sigma-Aldrich) and equilibrated as hanging-drops over a 500 μL reservoir containing 35% PEG 1500 (Fluka), 400 mM Li_2_SO_4_, 20 mM HEPES of pH varying in the range 6.0–8.5. Bright yellow octahedron crystals (100 μm) appeared after about 5 days at 19 °C and grew to about 200 μm in length by 14 days. Crystals of light-exposed (30, 60, 120, 180 and 240 min) and light-protected LlTrxR were harvested and flash-cooled directly from the drop without additional cryoprotectants.

Crystals obtained in FO conformation: LlTrxR (2 μL, 8–10 mg/mL) was mixed with 2 μL reservoir buffer, and 2 μL 60 mM DTT and equilibrated as hanging-drops over a 500 μL reservoir containing 20% PEG 4000 (Fluka), 400 mM Li_2_SO_4_. Only non-irradiated LlTrxR crystallized under these reducing conditions. Crystals appeared after ~14 days at 19 °C. Crystals were transferred to a cryoprotectant solution consisting of reservoir buffer with 10% ethylene glycol before storing in liquid nitrogen.

### Data collection, structure determination and refinement

X-ray diffraction data were collected at 100 K at I911–3, MAXII, Sweden and at ID30A-1, ESRF, France, with wavelengths ranging from 0.97–1.00 Å. Diffraction data were indexed, integrated, scaled and merged with XDS and XSCALE^32^ or MOSFLM^33^. Molecular replacement was performed with the program MOLREP^34^ from the CCP4 suite^35^ using the structure of SaTrxR (PDB 4GCM) as the initial search model for the FO conformation, while EcTrxR (PDB 1F6M) was used as template for the FR conformation. Restrained refinement was carried out using Refmac5^36^ and manual inspection was done in Coot^37^. Local non-crystallographic symmetry restraints were imposed when appropriate. LlTrxR obtained in FR conformation crystallized in space group P4_1_2_1_2_1_ with 1 monomer per asymmetric unit, while FO conformations crystallized in P2_1_ with 4 monomers per asymmetric unit. After refinement, the Ramachandran plots of the structures had 94.4–97.3%% residues in favored regions, 2.5–5.0% residues in allowed regions and 0–0.66% residues outliers. Details on the data collection statistics are provided in Table 1. All RMSD values for structural alignments of the Cα-atoms were calculated using the program Superpose in CCP4^38^. Figures displaying structural data were made with PyMOL (http://www.pymol.org/).

### Mass spectrometry

LlTrxR (20 μg; irradiated or light-protected) in 8 M urea, 50 mM NH_4_CO_3_ (45 μL) was added to 2.4 μL 100 mM DTT and incubated (40 min, 21 °C), followed by addition of 2.5 μL 200 mM iodoacetamide and incubation in the dark (40 min, 21 °C). After 1:10 dilution with 50 mM NH_4_CO_3_, endoproteinase GluC (Roche) was added to the samples in a 1:40 enzyme:protein ratio (w/w) and incubated overnight at 29 °C. Trifluoroacetic acid was added to the digested samples to a final concentration of 0.5% and subjected to StageTip purification with empore C18 disks (3 M) as previously described^39^. Samples (1 μg) were analysed on a Q-Exactive Orbitrap (Thermo Fisher Scientific) coupled to an EASY-nLC 1000 liquid chromatograph (Thermo Fischer Scientific). Peptides were loaded onto a custom-made nanoLC column (15 cm, C18, 100 Å, 1.9 μm particle size, 75 μm ID) packed into a Picofrit emitter (New Objectives) and eluted using a 70 min gradient at a flow rate of 250 nL min^−1^. Resolution of 70,000, automatic gain control (AGC) value of 3·10^6^, maximum injection time (IT) of 20 ms and scan range of 300 to 1500 *m/z* were used for MS scans. MS/MS spectra were acquired in data-dependent mode (Top 10 method) with resolution of 17,500, AGC value of 1·10^6^, and maximum fragmentation accumulation time of 60 ms.

## Additional Information

**Accession codes:** Coordinates and structure factors have been deposited in the Protein Data Bank under accession codes: 5MH4 (FR conformation, no light); 5MIP (FR conformation, 30 min light); 5MIQ (FR conformation, 60 min light); 5MIR (FR conformation, 120 min light); 5MIS (FR conformation, 180 min light); 5MIT (FR conformation, 240 min light); 5MJK (FO conformation, no light).

**How to cite this article**: Skjoldager, N. *et al*. The Structure of *Lactococcus lactis* thioredoxin reductase reveals molecular features of photo-oxidative damage. *Sci. Rep.* **7**, 46282; doi: 10.1038/srep46282 (2017).

**Publisher's note:** Springer Nature remains neutral with regard to jurisdictional claims in published maps and institutional affiliations.

## Supplementary Material

Supplementary Information

## Figures and Tables

**Table 1:** Data collection and refinement statistics (molecular replacement).

	LlTrxR^a^ No Light (PDB 5MH4)	LlTrxR^a^ 30 min Light (PDB 5MIP)	LlTrxR^a^ 60 min Light (PDB 5MIQ)	LlTrxR^a^ 120 min Light (PDB 5MIR)	LlTrxR^a^ 180 min Light (PDB 5MIS)	LlTrxR^a^ 240 min Light (PDB 5MIT)	LlTrxR^b^ No Light (PDB: 5MJK)
**Data collection**							
Space group	*P4*_*1*_*2*_*1*_*2*	*P4*_*1*_*2*_*1*_*2*	*P4*_*1*_*2*_*1*_*2*	*P4*_*1*_*2*_*1*_*2*	*P4*_*1*_*2*_*1*_*2*	*P4*_*1*_*2*_*1*_*2*	*P2*_*1*_
Cell dimensions							
*a, b, c* (Å)	120.54, 120.54, 60.47	121.43, 121.43 60.7	121.46, 121.46, 60.71	121.17, 121.17, 60.62	120.92, 120.92, 60.45	120.69, 120.69, 60.33	73.61, 132.26, 73.5
α, β, γ (°)	90, 90, 90	90, 90, 90	90, 90, 90	90, 90, 90	90, 90, 90	90, 90, 90	90, 112.62, 90
Resolution (Å)	85.24–2.14 (2.22–2.14)	42.94–2.0 (2.05–2.00)^c^	42.95–1.92 (1.97–1.92)	42.86–2.00 (2.05–2.00)	60.45–1.81 (1.84–1.81)	60.34 (1.84–1.80)	47.36–2.00 (2.05–2.00)^c^
*R*_merge_	0.127 (1.74)	0.116 (1.224)	0.096 (1.461)	0.073 (0.509)^c^	0.088 (1.063)	0.134 (1.783)	0.088(0.826)^c^
*I* /σ (I)	12.3 (1.3)	13.24 (1.74)^c^	15.69 (1.18) ^c^	20.13 (3.66)^c^	17.5 (1.9)^c^	14.6 (1.5)^c^	12.2(1.8)^c^
CC(½)	99.7(47.7)	99.8(61.9)	99.9(49.5)	99.9(88.9)	99.9(52.1)	99.9(31.7)	99.7(63.3)
Completeness (%)	99.9 (100)	96.2 (98.1)^c^	99.6 (99.8)^c^	99.8 (100.0)^c^	99.9 (99.9)^c^	99.8 (98.9)^c^	99.0 (98.4)^c^
Redundancy	8.4 (8.5)	6.6 (6.5)^c^	7.4 (6.3)^c^	7.5 (7.8)^c^	10.2 (8.1)^c^	13.5 (10.7)^c^	3.8 (3.8)^c^
Refinement							
Resolution (Å)	49.37–2.14	42.94–2.00	42.95–1.92	42.86–2.00	54.13–1.81	54.03–1.80	47.36–2.00
No. reflections	210,842 (20,402)	198,639 (14,547)^c^	260,449 (16,150)^c^	231,711 (17,615)^c^	423,277 (19,394)^c^	560,996 (25,330)^c^	332,553 (24,494)^c^
*R*_work_/*R*_free_	0.1767/0.2373	0.1947/0.2276	0.1947/0.2276	0.1826/0.2166	0.1895/0.2320	0.1937/0.2335	0.2299/0.2760
No. atoms							
Protein	2,347	2,347	2,347	2,347	2,347	2,347	9,356
Ligand/ion	118	125	125	125	125	125	232
Water	166	224	239	247	256	250	409
*B*-factors							
Protein	44.3	34.7	35.3	31.5	29.9	31.4	35.1
Ligand/ion	39.1	33.5	33.6	28.0	27.4	29.3	25.1
Water	50.2	44.7	44.8	40.1	39.5	40.9	33.2
R.m.s. deviations							
Bond lengths (Å)	0.018	0.019	0.019	0.018	0.022	0.019	0.015
Bond angles (°)	2.0	2.2	2.0	2.1	2.3	2.1	1.7

^a^The structure was obtained in FR conformation. ^b^The structure was obtained in FO conformation. ^c^Values in parentheses are for highest-resolution shell.

^*^Number of xtals for each structure should be noted in footnote.
