# Supplementary material for: The structure of Lactococcus lactis thioredoxin reductase reveals molecular features of photo-oxidative damage
Source: Sci Rep. 2017 Apr 11;7:46282. doi: 10.1038/srep46282 (PMC5387739; doi:10.1038/srep46282)
Supplement: Supplementary Information [file srep46282-s1.pdf]

Supplementary information

The structure of *Lactococcus lactis* thioredoxin reductase reveals molecular features of photo-oxidative damage

Nicklas Skjoldager[1], Maria Blanner Bang[2], Martin Rykær[1], Olof Björnberg[1], Michael J Davies[3], Birte Svensson[1], Pernille Harris[2*] & Per Hägglund[1*]

[1]Department of Biotechnology and Biomedicine, Technical University of Denmark, DK-2800 Kgs. Lyngby, Denmark

[2]Department of Chemistry, Technical University of Denmark

[3]Department of Biomedical Sciences, University of Copenhagen

[*]Correspondence should be addressed to Per Hägglund (ph@bio.dtu.dk) or Pernille Harris

(ph@kemi.dtu.dk).

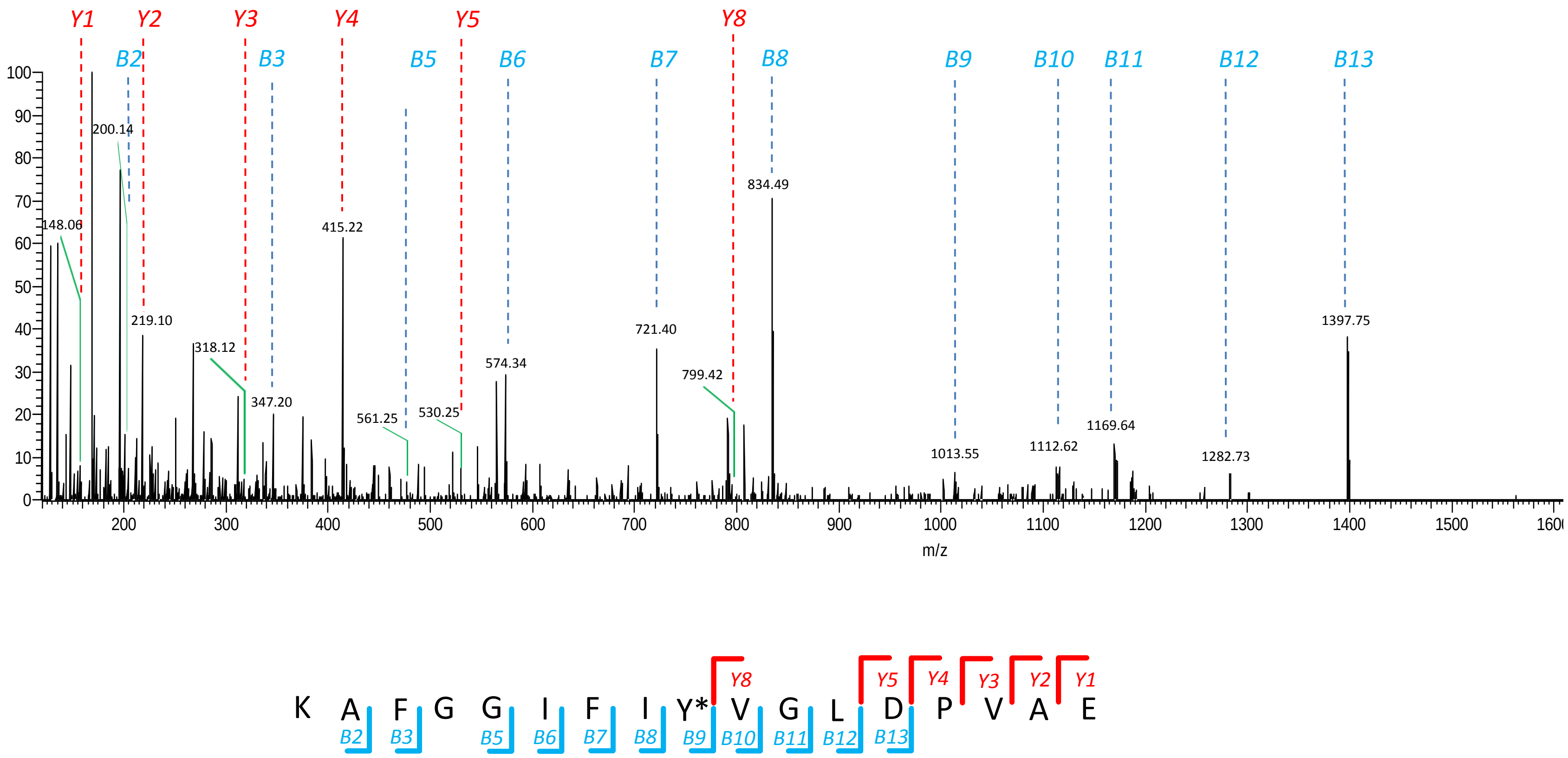

Figure S1a. Annotated MS/MS spectrum of the precursor at m/z 906.98 corresponding to the peptide KAFGGIFIYVGLDPVAE with an oxidized tyrosine (Y*)

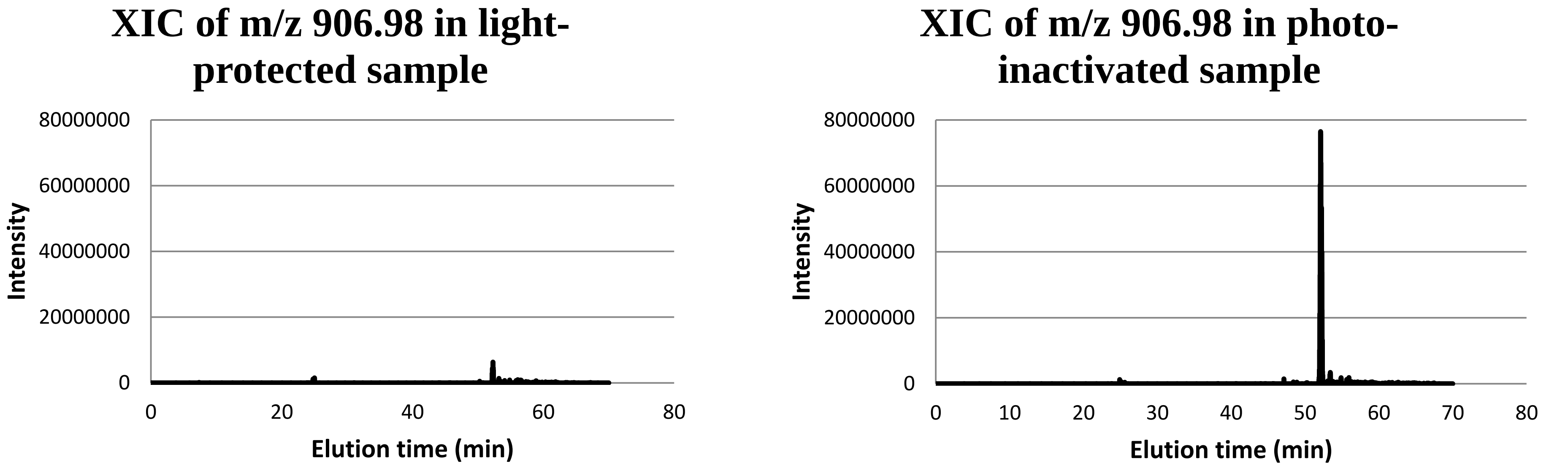

Figure S1b. Extracted ion chromatograms (XIC) of the precursor at m/z 906.98 corresponding to the peptide KAFGGIFIYVGLDPVAE with an oxidized tyrosine

Figure S2. Alignment of TrxR sequences. The positions corresponding to Met43 and Tyr237 in LlTrxR are highlighted in yellow and green, respectively. Accession numbers are from OmaBrowser (http://omabrowser.org/oma/home/)

```
CLUSTAL O(1.2.2) multiple sequence alignment


STRT101547      ------------------------------------------------------------
STRT201508      ------------------------------------------------------------
STRTD01356      ------------------------------------------------------------
STRTN01533      ------------------------------------------------------------
STRE500345      ------------------------------------------------------------
STRE801625      ------------------------------------------------------------
STREH01636      ------------------------------------------------------------
STREC01656      ------------------------------------------------------------
STREM01495      ------------------------------------------------------------
STRE401629      ------------------------------------------------------------
STRS700387      ------------------------------------------------------------
STRDG01631      ------------------------------------------------------------
STRP301388      ------------------------------------------------------------
STRPZ01205      ------------------------------------------------------------
STRPQ00464      ------------------------------------------------------------
STRPD01453      ------------------------------------------------------------
STRP601399      ------------------------------------------------------------
STRP801366      ------------------------------------------------------------
STRPF01453      ------------------------------------------------------------
STRPG00427      ------------------------------------------------------------
STRA300284      ------------------------------------------------------------
STRA500290      ------------------------------------------------------------
STRA100288      ------------------------------------------------------------
STRA200291      ------------------------------------------------------------
STRIC00348      ------------------------------------------------------------
STRPX00375      ------------------------------------------------------------
STRMD00432      ------------------------------------------------------------
STRS201734      ------------------------------------------------------------
STRSY01721      ------------------------------------------------------------
STRSX01553      ------------------------------------------------------------
STRSE01478      ------------------------------------------------------------
STREJ01635      ------------------------------------------------------------
STRGZ01544      ------------------------------------------------------------
STRS401593      ------------------------------------------------------------
LACGT00720      ------------------------------------------------------------
LACGL00738      ------------------------------------------------------------
STRSV01774      ------------------------------------------------------------
STRIJ00350      ------------------------------------------------------------
STROU01248      ------------------------------------------------------------
STRM601286      ------------------------------------------------------------
STRES00962      ------------------------------------------------------------
STRP701387      ------------------------------------------------------------
STRZT00756      ------------------------------------------------------------
STRP001079      ------------------------------------------------------------
STRZO01270      ------------------------------------------------------------
STRZ600815      ------------------------------------------------------------
STRET00826      ------------------------------------------------------------
STRPS01429      ------------------------------------------------------------
STRZN01287      ------------------------------------------------------------
STRR601306      ------------------------------------------------------------
STRP201217      ------------------------------------------------------------
STRZP01342      ------------------------------------------------------------
STRZI01226      ------------------------------------------------------------
STRPN01360      ------------------------------------------------------------
STRP401357      ------------------------------------------------------------
STRZJ01282      ------------------------------------------------------------
STRPJ01336      ------------------------------------------------------------
STRPI01436      ------------------------------------------------------------
MARHT00044      ------------------------------------------------------------
```

```
THEP300413      ------------------------------------------------------------
THEPX00884      ------------------------------------------------------------
THESX01857      ------------------------------------------------------------
THEM301788      ------------------------------------------------------------
THEIA01812      ------------------------------------------------------------
THETC00443      ------------------------------------------------------------
THESW01026      ------------------------------------------------------------
THEXL00360      ------------------------------------------------------------
THEID01127      ------------------------------------------------------------
THEOJ00147      ------------------------------------------------------------
DESAS01237      ------------------------------------------------------------
DESK701373      ------------------------------------------------------------
KYRT200729      ------------------------------------------------------------
STACT00410      ------------------------------------------------------------
STAS101952      ------------------------------------------------------------
STALH01969      ------------------------------------------------------------
STAEQ00422      ------------------------------------------------------------
STAES00543      ------------------------------------------------------------
STAAB00714      ------------------------------------------------------------
STAA500765      ------------------------------------------------------------
STAAM00757      ------------------------------------------------------------
STAAW00726      ------------------------------------------------------------
STAAS00733      ------------------------------------------------------------
STAAN00727      ------------------------------------------------------------
STAAC00807      ------------------------------------------------------------
STAA300727      ------------------------------------------------------------
STAA800734      ------------------------------------------------------------
STAA100752      ------------------------------------------------------------
STAA200774      ------------------------------------------------------------
STAA900759      ------------------------------------------------------------
STAAE00716      ------------------------------------------------------------
STAAT00767      ------------------------------------------------------------
STAAD00695      ------------------------------------------------------------
STAA000815      ------------------------------------------------------------
STAAH02407      ------------------------------------------------------------
STAAF00763      ------------------------------------------------------------
STAAK00744      ------------------------------------------------------------
STAAJ00703      ------------------------------------------------------------
STAAG00690      ------------------------------------------------------------
STAA400732      ------------------------------------------------------------
STAAR00788      ------------------------------------------------------------
LISSS02378      ------------------------------------------------------------
LISIN02590      ------------------------------------------------------------
LISW602421      ------------------------------------------------------------
EXISA00870      ------------------------------------------------------------
EXIS202358      ------------------------------------------------------------
EXIAB02180      ------------------------------------------------------------
OCEIH02462      ------------------------------------------------------------
BACIE01058      ------------------------------------------------------------
BACCJ03468      ------------------------------------------------------------
BACHD03507      ------------------------------------------------------------
BACPE03382      ------------------------------------------------------------
SOLSS00743      ------------------------------------------------------------
BACC600715      ------------------------------------------------------------
ANOFW02486      ------------------------------------------------------------
GEOKA03042      ------------------------------------------------------------
GEOSY02976      ------------------------------------------------------------
GEOTN02952      ------------------------------------------------------------
GEOSW02588      ------------------------------------------------------------
GEOS000381      ------------------------------------------------------------
GEOTC00378      ------------------------------------------------------------
BACMD04906      ------------------------------------------------------------
BACMQ04908      ------------------------------------------------------------
BACWK04830      ------------------------------------------------------------
BACAN04889      ------------------------------------------------------------
```

```
BACC105210      ------------------------------------------------------------
BACC705051      ------------------------------------------------------------
BACC005112      ------------------------------------------------------------
BACC305043      ------------------------------------------------------------
BACAC05232      ------------------------------------------------------------
BACAA04720      ------------------------------------------------------------
BACT005085      ------------------------------------------------------------
BACC205124      ------------------------------------------------------------
BACC405064      ------------------------------------------------------------
BACT104712      ------------------------------------------------------------
BACLD03769      ------------------------------------------------------------
BACPZ03376      ------------------------------------------------------------
BACSU03600      ------------------------------------------------------------
BACST01653      ------------------------------------------------------------
BACPT03541      ------------------------------------------------------------
LEUGG00661      ------------------------------------------------------------
LEUGJ00629      ------------------------------------------------------------
LEUCJ00485      ------------------------------------------------------------
LACAR00657      ------------------------------------------------------------
LACA300647      ------------------------------------------------------------
LACAL00643      ------------------------------------------------------------
LACKZ00964      ------------------------------------------------------------
LACRJ00357      ------------------------------------------------------------
LACRD00361      ------------------------------------------------------------
LACRS01501      ------------------------------------------------------------
LACSM00455      ------------------------------------------------------------
LACRG00878      ------------------------------------------------------------
LACRL00939      ------------------------------------------------------------
LACC300844      ------------------------------------------------------------
LACCZ00824      ------------------------------------------------------------
LACCB00988      ------------------------------------------------------------
LACCD01016      ------------------------------------------------------------
LACCC01014      ------------------------------------------------------------
LACBN01277      ------------------------------------------------------------
LACBA00605      ------------------------------------------------------------
LACPL00650      ------------------------------------------------------------
LACPJ00628      ------------------------------------------------------------
LACPS00585      ------------------------------------------------------------
PEDCP00497      ------------------------------------------------------------
CARS100359      ------------------------------------------------------------
AERUA00263      ------------------------------------------------------------
ELUMP00590      ------------------------------------------------------------
SPHPG02940      ------------------------------------------------------------
SPHGB01868      ------------------------------------------------------------
TREPZ00273      ------------------------------------------------------------
TREAZ03414      ------------------------------------------------------------
SPITD00734      ------------------------------------------------------------
SPITZ01373      ------------------------------------------------------------
TREPA00803      ------------------------------------------------------------
TREPS00802      ------------------------------------------------------------
TREPC00747      ------------------------------------------------------------
TREPM00823      ------------------------------------------------------------
TREPD00824      ------------------------------------------------------------
TREPU00781      ------------------------------------------------------------
ENCCU00216      ------------------------------------------------------------
HELM100964      ------------------------------------------------------------
HELCP01490      ------------------------------------------------------------
ARCFU01526      ------------------------------------------------------------
FERPA02445      ------------------------------------------------------------
ARCVS01910      ------------------------------------------------------------
METEZ00677      ------------------------------------------------------------
METHD00869      ------------------------------------------------------------
METMA02304      ------------------------------------------------------------
METAC01311      ------------------------------------------------------------
KOSOT00298      ------------------------------------------------------------
```

```
MARPK01613      ------------------------------------------------------------
SLAHD02439      ------------------------------------------------------------
FILAD00976      ------------------------------------------------------------
BUTPB02463      ------------------------------------------------------------
CLOPH00250      ------------------------------------------------------------
CLOSW00592      ------------------------------------------------------------
LACFC00207      ------------------------------------------------------------
CRYCD00853      ------------------------------------------------------------
EGGLE01847      ------------------------------------------------------------
PYRFU01410      ------------------------------------------------------------
PYRHO01476      ------------------------------------------------------------
PYRAB00730      ------------------------------------------------------------
PYRSN00014      ------------------------------------------------------------
THEGJ00181      ------------------------------------------------------------
THEKO02097      ------------------------------------------------------------
THEON01610      ------------------------------------------------------------
THES401476      ------------------------------------------------------------
SYNWW02368      ------------------------------------------------------------
UNCTG00012      ------------------------------------------------------------
THEA101460      ------------------------------------------------------------
DENA201647      ------------------------------------------------------------
DEFDS00488      ------------------------------------------------------------
CALNY01292      ------------------------------------------------------------
SYNGF01652      ------------------------------------------------------------
DESB201123      ------------------------------------------------------------
DEIPM00845      ------------------------------------------------------------
DEIRA01924      ------------------------------------------------------------
DEIML01437      ------------------------------------------------------------
DEIGD02532      ------------------------------------------------------------
DEIDV00622      ------------------------------------------------------------
TRURR01079      ------------------------------------------------------------
THETG01863      ------------------------------------------------------------
THET201543      ------------------------------------------------------------
THET801911      ------------------------------------------------------------
OCEP502115      ------------------------------------------------------------
DEHLB00585      ------------------------------------------------------------
DEHMG00439      ------------------------------------------------------------
DEHMB00497      ------------------------------------------------------------
DEHMC00427      ------------------------------------------------------------
THELD00726      ------------------------------------------------------------
ANAMD00624      ------------------------------------------------------------
THEAS00815      ------------------------------------------------------------
AMICL00707      ------------------------------------------------------------
CLOCE01684      ------------------------------------------------------------
HYDS000616      ------------------------------------------------------------
HYDTT00224      ------------------------------------------------------------
PELTS01405      ------------------------------------------------------------
DESRL02179      ------------------------------------------------------------
BORBP00506      ------------------------------------------------------------
BORAP00518      ------------------------------------------------------------
BORBU00514      ------------------------------------------------------------
BORBZ00490      ------------------------------------------------------------
BORBN00493      ------------------------------------------------------------
BORRA00486      ------------------------------------------------------------
BORDL00498      ------------------------------------------------------------
BORHD00497      ------------------------------------------------------------
BORT900497      ------------------------------------------------------------
METKA01560      ------------------------------------------------------------
MYCA500361      ------------------------------------------------------------
METVS00145      ------------------------------------------------------------
METOI01431      ------------------------------------------------------------
MYCHN00051      ------------------------------------------------------------
MYCSL00465      ------------------------------------------------------------
MYCS300420      ------------------------------------------------------------
DESK101258      ------------------------------------------------------------
```

```
STAHD01472      ------------------------------------------------------------
THEC100270      ------------------------------------------------------------
NANEQ00478      ------------------------------------------------------------
CALLD01225      ------------------------------------------------------------
SULSO02155      ------------------------------------------------------------
SULS900210      ------------------------------------------------------------
SULIA00208      ------------------------------------------------------------
SULIM00207      ------------------------------------------------------------
SULIK00225      ------------------------------------------------------------
IGNH400907      ------------------------------------------------------------
KORCO01040      ------------------------------------------------------------
THESM01133      ------------------------------------------------------------
THEBM01534      ------------------------------------------------------------
METST01363      ------------------------------------------------------------
METTH00703      ------------------------------------------------------------
METSL02406      ------------------------------------------------------------
METLA02406      ------------------------------------------------------------
METPW00194      ------------------------------------------------------------
RUBXD00226      ------------------------------------------------------------
RHOM400178      ------------------------------------------------------------
GARV400051      ------------------------------------------------------------
BIFAP01644      ------------------------------------------------------------
SEGRD00014      ------------------------------------------------------------
GORB404535      ------------------------------------------------------------
GORPV04920      ------------------------------------------------------------
MYCA904903      ------------------------------------------------------------
MYCSS05356      ------------------------------------------------------------
MYCSJ05684      ------------------------------------------------------------
MYCSK05387      ------------------------------------------------------------
MYCS206581      ------------------------------------------------------------
MYCCN05158      ------------------------------------------------------------
MYCVP05890      ------------------------------------------------------------
MYCGI00775      ------------------------------------------------------------
MYCSR04994      ------------------------------------------------------------
AMYSD04545      ------------------------------------------------------------
MYCLE02681      ------------------------------------------------------------
MYCLB02681      ------------------------------------------------------------
MYCSD04323      ------------------------------------------------------------
MYCPA04306      ------------------------------------------------------------
MYCA105023      ------------------------------------------------------------
MYCUA04107      ------------------------------------------------------------
MYCMM05378      ------------------------------------------------------------
MYCA003905      ------------------------------------------------------------
MYCTU03943      ------------------------------------------------------------
MYCTF03846      ------------------------------------------------------------
MYCTA03980      ------------------------------------------------------------
MYCTK04010      ------------------------------------------------------------
MYCTC03612      ------------------------------------------------------------
MYCTD03548      ------------------------------------------------------------
MYCCP03898      ------------------------------------------------------------
MYCBO02863      ------------------------------------------------------------
MYCBP03913      ------------------------------------------------------------
MYCBT03913      ------------------------------------------------------------
TSUPD04078      ------------------------------------------------------------
ACTMD06897      ------------------------------------------------------------
SACES08402      ------------------------------------------------------------
SACVD03814      ------------------------------------------------------------
AMYMU09190      ------------------------------------------------------------
AMYMS10178      ------------------------------------------------------------
PSEUX06421      ------------------------------------------------------------
NOCFA05681      ------------------------------------------------------------
NOCCG05476      ------------------------------------------------------------
RHOE406010      ------------------------------------------------------------
RHOOB03468      ------------------------------------------------------------
RHOE104515      ------------------------------------------------------------
```

```
CORDI02302      ------------------------------------------------------------
CORD202219      ------------------------------------------------------------
CORDL02208      ------------------------------------------------------------
CORDJ02208      ------------------------------------------------------------
CORDH02221      ------------------------------------------------------------
CORD702316      ------------------------------------------------------------
CORD302337      ------------------------------------------------------------
CORDD02235      ------------------------------------------------------------
CORDV02170      ------------------------------------------------------------
CORDW02254      ------------------------------------------------------------
CORDK02230      ------------------------------------------------------------
COREF02870      ------------------------------------------------------------
CORGL03082      ------------------------------------------------------------
CORGK02974      ------------------------------------------------------------
CORGB03038      ------------------------------------------------------------
CORK402006      ------------------------------------------------------------
CORJK02028      ------------------------------------------------------------
CORVD02951      ------------------------------------------------------------
ARCHD01706      ------------------------------------------------------------
THET101205      ------------------------------------------------------------
PROAC02247      ------------------------------------------------------------
PROAS02302      ------------------------------------------------------------
CAERE29798      ------------------------------------------------------------
CELFA03749      ------------------------------------------------------------
JONDD02484      ------------------------------------------------------------
XYLCX03324      ------------------------------------------------------------
ACIC102144      ------------------------------------------------------------
FRADG04042      ------------------------------------------------------------
FRASU07065      ------------------------------------------------------------
FRASN07115      ------------------------------------------------------------
FRASC04424      ------------------------------------------------------------
FRAAA06712      ------------------------------------------------------------
KYTSD02521      ------------------------------------------------------------
KINRD04462      ------------------------------------------------------------
NOCDD04789      ------------------------------------------------------------
NOCAA01941      ------------------------------------------------------------
STRRD08913      ------------------------------------------------------------
THECD04863      ------------------------------------------------------------
THEBD03528      ------------------------------------------------------------
CATAD08894      ------------------------------------------------------------
KRIFD06917      ------------------------------------------------------------
KITSK03730      ------------------------------------------------------------
STRBB05366      ------------------------------------------------------------
STRVP03660      ------------------------------------------------------------
STRSW04381      ------------------------------------------------------------
STRGG03562      ------------------------------------------------------------
STRFA03098      ------------------------------------------------------------
STRAW04303      ------------------------------------------------------------
STRCO02929      ------------------------------------------------------------
STRHJ05105      ------------------------------------------------------------
MONBE04991      ------------------------------------------------------------
CHLRE01313      ------------------------------------------------------------
MEDTR25591      ------------------------------------------------------------
SOLLC13750      ------------------------------------------------------------
PRUPE10733      ------------------------------------------------------------
MANES18605      ------------------------------------------------------------
THECC00884      ------------------------------------------------------------
PHYPA31147      --------MLAKQSKALHKPPAL-----------------------LAAFNFSRSCYHSF
AMBTC19471      -------------------------------------------MRRFLALFFESS--RPI
MUSAC26038      -------------------------------------------MN---------------
MUSAM33177      -------------------------------------------MN---------------
SETIT03079      MGHRIVLFIPVHSNKTLHPNHHLFEPLLREPKGESVDSRLRVPMNVFVLLQSPES--KSM
ORYBR12195      ------------------------------------------------------------
COCLU07729      ------------------------------------------------------------
PHANO13702      ------------------------------------------------------------
```

```
PHAND10804      ------------------------------------------------------------
AURPU02089      ------------------------------------------------------------
ZYMTR07711      ------------------------------------------------------------
DICPU05926      ------------------------------------------------------------
ENTHI00522      ------------------------------------------------------------
LEPBA02231      ------------------------------------------------------------
LEPBP02301      ------------------------------------------------------------
LEPBL01462      ------------------------------------------------------------
LEPBJ01276      ------------------------------------------------------------
LEPIN02475      ------------------------------------------------------------
LEPII01996      ------------------------------------------------------------
LEPIC01426      ------------------------------------------------------------
SPIAZ00697      ------------------------------------------------------------
PENRW10140      ------------------------------------------------------------
PENCH09104      ------------------------------------------------------------
EURHE07269      ------------------------------------------------------------
ASPAC07301      ------------------------------------------------------------
EMENI10387      ------------------------------------------------------------
EMEND02596      ------------------------------------------------------------
ASPTN06742      ------------------------------------------------------------
ASPCL04014      ------------------------------------------------------------
ASPFU05647      ------------------------------------------------------------
NEOFI00452      ------------------------------------------------------------
CRYPA10563      ------------------------------------------------------------
BLUGR03498      ------------------------------------------------------------
SCLS112814      ------------------------------------------------------------
MAGGR04266      ------------------------------------------------------------
NEUCR01575      ------------------------------------------------------------
NEUT908941      ------------------------------------------------------------
VERDA02342      ------------------------------------------------------------
COLSU12486      ------------------------------------------------------------
HYPAI01684      ------------------------------------------------------------
HYPVG06080      ------------------------------------------------------------
HYPJE05895      ------------------------------------------------------------
NECHA05020      ------------------------------------------------------------
FUSO415847      ------------------------------------------------------------
GIBZA01026      ------------------------------------------------------------
SCHPO04025      ------------------------------------------------------------
YARLI03635      ------------------------------------------------------------
ASHGO00946      ------------------------------------------------------------
KLULA02190      ------------------------------------------------------------
ZYGRO00676      ------------------------------------------------------------
DEKBR01813      ------------------------------------------------------------
PICPG04776      ------------------------------------------------------------
CANTE00916      ------------------------------------------------------------
LODEL03891      ------------------------------------------------------------
DEBHA05546      ------------------------------------------------------------
SPAPN03477      ------------------------------------------------------------
CANAW04800      ------------------------------------------------------------
PICST04701      ------------------------------------------------------------
PUCGT10887      ------------------------------------------------------------
PUCGR11813      ------------------------------------------------------------
PHYBL11006      ------------------------------------------------------------
USTMA03757      ------------------------------------------------------------
USTHO04132      ------------------------------------------------------------
WALSE04527      ------------------------------------------------------------
TREME07701      ------------------------------------------------------------
AURST04751      ------------------------------------------------------------
FOMME10177      ------------------------------------------------------------
CONPW06392      ------------------------------------------------------------
STEHR07076      ------------------------------------------------------------
HETAN06295      ------------------------------------------------------------
GLOTR06982      ------------------------------------------------------------
PUNST01981      ------------------------------------------------------------
LACBI02877      ------------------------------------------------------------
```

```
COPCI16429      ------------------------------------------------------------
DICSQ11618      ------------------------------------------------------------
TRAVS13180      ------------------------------------------------------------
WOLCO03584      ------------------------------------------------------------
FOMPI05979      ------------------------------------------------------------
PHLGI10219      ------------------------------------------------------------
PHACH05757      ------------------------------------------------------------
RICTY00419      ------------------------------------------------------------
RICPR00429      ------------------------------------------------------------
RICPP00461      ------------------------------------------------------------
RICBR00434      ------------------------------------------------------------
RICB800988      ------------------------------------------------------------
RICCK00600      ------------------------------------------------------------
RICAH00614      ------------------------------------------------------------
RICAC00782      ------------------------------------------------------------
RICFE00656      ------------------------------------------------------------
RICMS00042      ------------------------------------------------------------
RICM500453      ------------------------------------------------------------
RICR300665      ------------------------------------------------------------
RICAG00651      ------------------------------------------------------------
RICP300630      ------------------------------------------------------------
RICRS00632      ------------------------------------------------------------
RICRO00663      ------------------------------------------------------------
RICCN00618      ------------------------------------------------------------
RICPT00620      ------------------------------------------------------------
RICAE00497      ------------------------------------------------------------
RICJY00467      ------------------------------------------------------------
RICPU00072      ------------------------------------------------------------
RICS100537      ------------------------------------------------------------
BARBK00891      ------------------------------------------------------------
BARVW00932      ------------------------------------------------------------
BART100617      ------------------------------------------------------------
BARGA00466      ------------------------------------------------------------
BARHE01107      ------------------------------------------------------------
BARQU00895      ------------------------------------------------------------
OCHA401642      ------------------------------------------------------------
BRUAB01390      ------------------------------------------------------------
BRUA201507      ------------------------------------------------------------
BRUA101339      ------------------------------------------------------------
BRUSU01458      ------------------------------------------------------------
BRUME00510      ------------------------------------------------------------
BRUSI01461      ------------------------------------------------------------
BRUC201446      ------------------------------------------------------------
BRUMC01440      ------------------------------------------------------------
BRUMB01421      ------------------------------------------------------------
BRUM501487      ------------------------------------------------------------
BRUO201285      ------------------------------------------------------------
RHILO01973      ------------------------------------------------------------
CHESB02097      ------------------------------------------------------------
METPB00996      ------------------------------------------------------------
METEP01063      ------------------------------------------------------------
METEA00810      ------------------------------------------------------------
METED01453      ------------------------------------------------------------
METS403554      ------------------------------------------------------------
METNO05482      ------------------------------------------------------------
METSZ03234      ------------------------------------------------------------
BEII900056      ------------------------------------------------------------
METSB02743      ------------------------------------------------------------
MAGMM00401      ------------------------------------------------------------
HYPNA00542      ------------------------------------------------------------
KETVY00902      ------------------------------------------------------------
KETVW00472      ------------------------------------------------------------
ROSDO03242      ------------------------------------------------------------
ROSLO02615      ------------------------------------------------------------
RUEPO00888      ------------------------------------------------------------
```

```
RUEST00613      ------------------------------------------------------------
PHAIB02390      ------------------------------------------------------------
PARDP02130      ------------------------------------------------------------
DINSH02620      ------------------------------------------------------------
RHOCB02768      ------------------------------------------------------------
RHOS500248      ------------------------------------------------------------
RHOS400150      ------------------------------------------------------------
RHOS100227      ------------------------------------------------------------
RHOSK02952      ------------------------------------------------------------
MIDMI00790      ------------------------------------------------------------
ACEP301595      ------------------------------------------------------------
MICAA01566      ------------------------------------------------------------
TISMK03676      ------------------------------------------------------------
AZOL402409      ------------------------------------------------------------
PSEUV04348      ------------------------------------------------------------
HIRBI01095      ------------------------------------------------------------
PARL102241      ------------------------------------------------------------
MARMM02139      ------------------------------------------------------------
PHEZH02588      ------------------------------------------------------------
CAUCR02826      ------------------------------------------------------------
CAUCN02939      ------------------------------------------------------------
CAUST00891      ------------------------------------------------------------
PARBH01522      ------------------------------------------------------------
PELHB02084      ------------------------------------------------------------
HYPDA03273      ------------------------------------------------------------
HYPSM04642      ------------------------------------------------------------
OLICO01107      ------------------------------------------------------------
OLICM02790      ------------------------------------------------------------
RHOPS01446      ------------------------------------------------------------
RHOPA04062      ------------------------------------------------------------
RHOPT04533      ------------------------------------------------------------
RHOPX04235      ------------------------------------------------------------
BRADU07321      ------------------------------------------------------------
BRASO01239      ------------------------------------------------------------
BRASB06352      ------------------------------------------------------------
RHOPB03773      ------------------------------------------------------------
NITWN02324      ------------------------------------------------------------
NITHX02707      ------------------------------------------------------------
AZOC501143      ------------------------------------------------------------
XANP202679      ------------------------------------------------------------
CHLTF01983      ------------------------------------------------------------
IGNAJ02236      ------------------------------------------------------------
MELRP00403      ------------------------------------------------------------
ANADF00468      ------------------------------------------------------------
CHLCH01125      ------------------------------------------------------------
PELPB01419      ------------------------------------------------------------
CHLL200781      ------------------------------------------------------------
CHLTE00830      ------------------------------------------------------------
CHLP800854      ------------------------------------------------------------
CHLL701225      ------------------------------------------------------------
CHLPM00993      ------------------------------------------------------------
WOLTR00562      ------------------------------------------------------------
WOLPP00135      ------------------------------------------------------------
WOLPM00650      ------------------------------------------------------------
WOLWR00416      ------------------------------------------------------------
EHRCR00696      ------------------------------------------------------------
ANAMM00345      ------------------------------------------------------------
ANAMF00341      ------------------------------------------------------------
ANAPZ00610      ------------------------------------------------------------
NEOSM00537      ------------------------------------------------------------
NEORI00513      ------------------------------------------------------------
PELUB00076      ------------------------------------------------------------
PELSM00736      ------------------------------------------------------------
PUNMI01409      ------------------------------------------------------------
ASTEC00651      ------------------------------------------------------------
```

```
ZYMMT00154      ------------------------------------------------------------
ZYMMO00984      ------------------------------------------------------------
ZYMMA00176      ------------------------------------------------------------
ZYMMN00183      ------------------------------------------------------------
SPHAL00143      ------------------------------------------------------------
SPHWW03978      ------------------------------------------------------------
SPHJU02313      ------------------------------------------------------------
NOVAD02319      ------------------------------------------------------------
ERYLH00934      ------------------------------------------------------------
GRABC00720      ------------------------------------------------------------
GLUDA03075      ------------------------------------------------------------
KOMMN00774      ------------------------------------------------------------
HALVD01097      ------------------------------------------------------------
HALHT01699      ------------------------------------------------------------
METI401038      ------------------------------------------------------------
PLAL201612      ------------------------------------------------------------
GEMAT01983      ------------------------------------------------------------
CYAAP02456      ------------------------------------------------------------
CHLPN00303      ------------------------------------------------------------
CHLPP00439      ------------------------------------------------------------
CHLPE00727      ------------------------------------------------------------
CHLTR00101      ------------------------------------------------------------
CHLTA00102      ------------------------------------------------------------
CHLTJ00101      ------------------------------------------------------------
CHLTD00098      ------------------------------------------------------------
CHLT700102      ------------------------------------------------------------
CHLT000101      ------------------------------------------------------------
CHLT500104      ------------------------------------------------------------
CHLTL00098      ------------------------------------------------------------
CHLTG00102      ------------------------------------------------------------
CHLTS00101      ------------------------------------------------------------
CHLT900102      ------------------------------------------------------------
CHLTZ00101      ------------------------------------------------------------
CHLT400432      ------------------------------------------------------------
CHLT100103      ------------------------------------------------------------
CHLT200350      ------------------------------------------------------------
CHLTB00350      ------------------------------------------------------------
CHLTC00378      ------------------------------------------------------------
SIMNZ00831      ------------------------------------------------------------
PARUW01706      ------------------------------------------------------------
WADCW00943      ------------------------------------------------------------
SINAD04980      ------------------------------------------------------------
BIFLB01558      ------------------------------------------------------------
BIFAB00498      ------------------------------------------------------------
BIFAV01561      ------------------------------------------------------------
BIFAS01557      ------------------------------------------------------------
BIFA001518      ------------------------------------------------------------
BIFBA01814      ------------------------------------------------------------
BIFAA01619      ------------------------------------------------------------
BIFDB02112      ------------------------------------------------------------
TERSS03091      ------------------------------------------------------------
GRATM03160      ------------------------------------------------------------
GRAMM03836      ------------------------------------------------------------
AKKM801247      ------------------------------------------------------------
OPITP04130      ------------------------------------------------------------
CORAD01794      ------------------------------------------------------------
BUCCC00180      ------------------------------------------------------------
CENSY00347      ------------------------------------------------------------
NITMS00668      ------------------------------------------------------------
MEIRD02823      ------------------------------------------------------------
HERA203780      ------------------------------------------------------------
CHLAA02018      ------------------------------------------------------------
CHLSY02175      ------------------------------------------------------------
CHLAD02599      ------------------------------------------------------------
CALAS01745      ------------------------------------------------------------
```

```
ANATU01696      ------------------------------------------------------------
SULMS00235      ------------------------------------------------------------
BLASB00041      ------------------------------------------------------------
BLASP00551      ------------------------------------------------------------
AZOPC00437      ------------------------------------------------------------
LEPBD01878      ------------------------------------------------------------
SALRD02577      ------------------------------------------------------------
SALRM02866      ------------------------------------------------------------
RIEPU00166      ------------------------------------------------------------
ORITB00015      ------------------------------------------------------------
ORITI01234      ------------------------------------------------------------
PREMB01541      ------------------------------------------------------------
PREDF01155      ------------------------------------------------------------
PREI702011      ------------------------------------------------------------
ALIFI01035      ------------------------------------------------------------
ODOSD00362      ------------------------------------------------------------
PRER201250      ------------------------------------------------------------
PALPW00206      ------------------------------------------------------------
PORGI00953      ------------------------------------------------------------
PORG301168      ------------------------------------------------------------
BACV803426      ------------------------------------------------------------
BACT601486      ------------------------------------------------------------
BACTN04290      ------------------------------------------------------------
BACFR01032      ------------------------------------------------------------
BACFN00913      ------------------------------------------------------------
BACF600963      ------------------------------------------------------------
OWEHD03413      ------------------------------------------------------------
PSYTT00390      ------------------------------------------------------------
NONDD00011      ------------------------------------------------------------
ROBBH02711      ------------------------------------------------------------
CELAD02592      ------------------------------------------------------------
CELLC02474      ------------------------------------------------------------
MARSH03072      ------------------------------------------------------------
MURRD00159      ------------------------------------------------------------
ZOBGA00152      ------------------------------------------------------------
GRAFK00949      ------------------------------------------------------------
ZUNPS02344      ------------------------------------------------------------
AEQSU02079      ------------------------------------------------------------
HALH105745      ------------------------------------------------------------
SAPGL02893      ------------------------------------------------------------
AMOA500014      ------------------------------------------------------------
FLAIG01802      ------------------------------------------------------------
FLACA02326      ------------------------------------------------------------
FLAJ100198      ------------------------------------------------------------
FLABF01528      ------------------------------------------------------------
CAPOD01333      ------------------------------------------------------------
CAPCC00414      ------------------------------------------------------------
FLELS01898      ------------------------------------------------------------
SOLCM01166      ------------------------------------------------------------
PEDHD01598      ------------------------------------------------------------
SPHS203207      ------------------------------------------------------------
LEAB401188      ------------------------------------------------------------
EMTOG00092      ------------------------------------------------------------
SPILD01737      ------------------------------------------------------------
DYAFD00084      ------------------------------------------------------------
CYCMS03063      ------------------------------------------------------------
ECHVK03326      ------------------------------------------------------------
BELBD02542      ------------------------------------------------------------
NITGG02112      ------------------------------------------------------------
THEM700111      ------------------------------------------------------------
CREAS01350      ------------------------------------------------------------
TURPD02772      ------------------------------------------------------------
SORC507603      ------------------------------------------------------------
BDEBA00337      ------------------------------------------------------------
STIAD02752      ------------------------------------------------------------
```

```
MYXXD01876      ------------------------------------------------------------
MYXFH03454      ------------------------------------------------------------
CORCM01960      ------------------------------------------------------------
MYXSD02209      ------------------------------------------------------------
LEPFC02126      ------------------------------------------------------------
LEPFM02294      ------------------------------------------------------------
SOLUE00582      ------------------------------------------------------------
KORVE01333      ------------------------------------------------------------
ACIC502553      ------------------------------------------------------------
ACIFD00030      ------------------------------------------------------------
HALMS01402      ------------------------------------------------------------
CHLPD01133      ------------------------------------------------------------
CHLPB01137      ------------------------------------------------------------
PROA200897      ------------------------------------------------------------
WIGBR00492      ------------------------------------------------------------
BUCA500289      ------------------------------------------------------------
BUCAI00292      ------------------------------------------------------------
BUCAF00306      ------------------------------------------------------------
BUCAT00287      ------------------------------------------------------------
BUCA000302      ------------------------------------------------------------
BAUCH00290      ------------------------------------------------------------
BLOVB00369      ------------------------------------------------------------
BLOFL00367      ------------------------------------------------------------
BLOPB00375      ------------------------------------------------------------
BUCAP00289      ------------------------------------------------------------
COXBU01001      ------------------------------------------------------------
COXBN01188      ------------------------------------------------------------
COXBR01117      ------------------------------------------------------------
COXB200717      ------------------------------------------------------------
COXB100914      ------------------------------------------------------------
FRAP200241      ------------------------------------------------------------
FRANT01012      ------------------------------------------------------------
FRACN00522      ------------------------------------------------------------
FRATT00465      ------------------------------------------------------------
FRAT100465      ------------------------------------------------------------
FRATE00462      ------------------------------------------------------------
FRATW01216      ------------------------------------------------------------
FRATM01110      ------------------------------------------------------------
FRACF00566      ------------------------------------------------------------
FRATO01212      ------------------------------------------------------------
FRATH01486      ------------------------------------------------------------
FRATF01216      ------------------------------------------------------------
FRATN00564      ------------------------------------------------------------
ACIF500512      ------------------------------------------------------------
ACIF200362      ------------------------------------------------------------
DECAR01284      ------------------------------------------------------------
NEIG100525      ------------------------------------------------------------
NEIG201314      ------------------------------------------------------------
NEIM800926      ------------------------------------------------------------
NEIMP01199      ------------------------------------------------------------
NEIMB01212      ------------------------------------------------------------
NEIMF01143      ------------------------------------------------------------
NEIML01119      ------------------------------------------------------------
NEIMM00780      ------------------------------------------------------------
NEIMH00827      ------------------------------------------------------------
NEIMG01168      ------------------------------------------------------------
NEIMN01254      ------------------------------------------------------------
NEIMO00810      ------------------------------------------------------------
NEIM701229      ------------------------------------------------------------
NEIMA01334      ------------------------------------------------------------
NEIMW01125      ------------------------------------------------------------
DICNV01037      ------------------------------------------------------------
VESOH00047      ------------------------------------------------------------
RUTMC00043      ------------------------------------------------------------
HALHL02255      ------------------------------------------------------------
```

```
PELPD03177      ------------------------------------------------------------
GEOS804030      ------------------------------------------------------------
GEOBB03636      ------------------------------------------------------------
GEOSM03700      ------------------------------------------------------------
HALNC00046      ------------------------------------------------------------
HAEPS00887      ------------------------------------------------------------
GALAU02285      ------------------------------------------------------------
HISS201000      ------------------------------------------------------------
HAES101151      ------------------------------------------------------------
PASMU00573      ------------------------------------------------------------
PASMH00579      ------------------------------------------------------------
ACTSZ01560      ------------------------------------------------------------
AGGAN00985      ------------------------------------------------------------
NITHN03181      ------------------------------------------------------------
NITOC00311      ------------------------------------------------------------
NITWC02498      ------------------------------------------------------------
METNJ00648      ------------------------------------------------------------
METFJ01616      ------------------------------------------------------------
ALKEH00244      ------------------------------------------------------------
MARMS03166      ------------------------------------------------------------
MARM102688      ------------------------------------------------------------
THICR00763      ------------------------------------------------------------
THICA00974      ------------------------------------------------------------
THIV600851      ------------------------------------------------------------
THISH01973      ------------------------------------------------------------
METAA00813      ------------------------------------------------------------
META200813      ------------------------------------------------------------
METMM02895      ------------------------------------------------------------
FRAAD00813      ------------------------------------------------------------
XYLFA01416      ------------------------------------------------------------
XYLFT00623      ------------------------------------------------------------
XYLF200667      ------------------------------------------------------------
XYLFG01622      ------------------------------------------------------------
XYLFM00690      ------------------------------------------------------------
PSEUP01680      ------------------------------------------------------------
STRM501972      ------------------------------------------------------------
PSEUU01524      ------------------------------------------------------------
XANAP01407      ------------------------------------------------------------
XANCP01918      ------------------------------------------------------------
XANC802154      ------------------------------------------------------------
XANCB02263      ------------------------------------------------------------
XANOR02379      ------------------------------------------------------------
XANOM02295      ------------------------------------------------------------
XANOP02318      ------------------------------------------------------------
XANAC01951      ------------------------------------------------------------
CYCSP01155      ------------------------------------------------------------
GEOLS03275      ------------------------------------------------------------
GEOUR03858      ------------------------------------------------------------
GEODF00692      ------------------------------------------------------------
GEOSL00482      ------------------------------------------------------------
GEOSK00469      ------------------------------------------------------------
GEOMG02998      ------------------------------------------------------------
MORCR00228      ------------------------------------------------------------
ACIAD00798      ------------------------------------------------------------
ACIBC00821      ------------------------------------------------------------
ACIBY02725      ------------------------------------------------------------
ACIB302691      ------------------------------------------------------------
ACIB500882      ------------------------------------------------------------
ACIB100850      ------------------------------------------------------------
ACIBD00843      ------------------------------------------------------------
ACIBS02229      ------------------------------------------------------------
ACICP00137      ------------------------------------------------------------
ACISD03052      ------------------------------------------------------------
LEGLN02402      ------------------------------------------------------------
LEGPA01726      ------------------------------------------------------------
```

```
LEGPH00846      ------------------------------------------------------------
LEGPC01171      ------------------------------------------------------------
LEGP201857      ------------------------------------------------------------
LEGPL01727      ------------------------------------------------------------
COLP302668      ------------------------------------------------------------
KANKD01106      ------------------------------------------------------------
IDILO00659      ------------------------------------------------------------
PSEU901284      ------------------------------------------------------------
PSEA602346      ------------------------------------------------------------
ALTSS01871      ------------------------------------------------------------
ALTMD01611      ------------------------------------------------------------
ALTME01705      ------------------------------------------------------------
ALTMB01797      ------------------------------------------------------------
ALTMS01652      ------------------------------------------------------------
SACD201683      ------------------------------------------------------------
TERTT01748      ------------------------------------------------------------
SIMAS00609      ------------------------------------------------------------
ALCDB01962      ------------------------------------------------------------
CHRSD02939      ------------------------------------------------------------
HALED02811      ------------------------------------------------------------
SIDLE02655      ------------------------------------------------------------
GALCS01621      ------------------------------------------------------------
LARHH01446      ------------------------------------------------------------
CHRVO02807      ------------------------------------------------------------
PSEUL01648      ------------------------------------------------------------
NITEU01859      ------------------------------------------------------------
NITEC00745      ------------------------------------------------------------
NITMU00023      ------------------------------------------------------------
NITSI03064      ------------------------------------------------------------
ACCPU02128      ------------------------------------------------------------
THIDA01000      ------------------------------------------------------------
METS601221      ------------------------------------------------------------
METGS01186      ------------------------------------------------------------
METFK00972      ------------------------------------------------------------
METML01428      ------------------------------------------------------------
AROAE03899      ------------------------------------------------------------
THASP01686      ------------------------------------------------------------
AZOSB01359      ------------------------------------------------------------
BORA102682      ------------------------------------------------------------
BORPA03415      ------------------------------------------------------------
BORBM03577      ------------------------------------------------------------
BORPE02280      ------------------------------------------------------------
BORPC02060      ------------------------------------------------------------
BORP102392      ------------------------------------------------------------
BORBR03870      ------------------------------------------------------------
BORPD01524      ------------------------------------------------------------
ACHXA01179      ------------------------------------------------------------
RHOFT03114      ------------------------------------------------------------
VEREI01701      ------------------------------------------------------------
VARPE01375      ------------------------------------------------------------
VARPS01266      ------------------------------------------------------------
DELAS05272      ------------------------------------------------------------
DELSC01235      ------------------------------------------------------------
COMT200883      ------------------------------------------------------------
ACIAC03267      ------------------------------------------------------------
ACIAP03249      ------------------------------------------------------------
ACIET02648      ------------------------------------------------------------
ALIDK03528      ------------------------------------------------------------
RUBGI03408      ------------------------------------------------------------
LEPCP00749      ------------------------------------------------------------
POLSJ03719      ------------------------------------------------------------
POLNA03144      ------------------------------------------------------------
METPP01110      ------------------------------------------------------------
RAMTT03218      ------------------------------------------------------------
RALPJ02457      ------------------------------------------------------------
```

```
RALP102102      ------------------------------------------------------------
RALSO02303      ------------------------------------------------------------
RALS801097      ------------------------------------------------------------
HERSS01878      ------------------------------------------------------------
HERAR00955      ------------------------------------------------------------
JANMA01152      ------------------------------------------------------------
THIK102324      ------------------------------------------------------------
POLSQ00682      ------------------------------------------------------------
POLNS00989      ------------------------------------------------------------
BURP800627      ------------------------------------------------------------
BURPP00988      ------------------------------------------------------------
BURSC00728      ------------------------------------------------------------
BURXL00982      ------------------------------------------------------------
BURSG00869      ------------------------------------------------------------
BURRH00629      --------MAEHRA--------------DGRRGYHADR-----WQNIVPM----------
BURGB00781      ------------------------------------------------------------
BURGS00853      ------------------------------------------------------------
BURPS02618      ------------------------------------------------------------
BURMA01741      ------------------------------------------------------------
BURP103016      ------------------------------------------------------------
BURP002984      ------------------------------------------------------------
BURM701866      ------------------------------------------------------------
BURP602945      ------------------------------------------------------------
BURM902465      ------------------------------------------------------------
BURMS00740      ------------------------------------------------------------
BURTA01515      ------------------------------------------------------------
BURM102386      ------------------------------------------------------------
BURL300890      ------------------------------------------------------------
BURVG00860      ------------------------------------------------------------
BURCM00829      ------------------------------------------------------------
BURA400832      ------------------------------------------------------------
BURCA00489      ------------------------------------------------------------
BURCH00966      ------------------------------------------------------------
BURCC00927      ------------------------------------------------------------
BURCJ02922      ------------------------------------------------------------
EDWI902348      ------------------------------------------------------------
EDWTF01971      ------------------------------------------------------------
EDWTE02176      ------------------------------------------------------------
SODGM01094      ------------------------------------------------------------
MOREP00394      ------------------------------------------------------------
RAHSY01432      ------------------------------------------------------------
RAHAC01419      ------------------------------------------------------------
ERWBE01489      ------------------------------------------------------------
PANAM01339      ------------------------------------------------------------
PANAA00662      ------------------------------------------------------------
PANSA01303      ------------------------------------------------------------
ERWT902154      ------------------------------------------------------------
ERWAC01330      ------------------------------------------------------------
ERWAE01326      ------------------------------------------------------------
ERWPE02241      ------------------------------------------------------------
ERWP602414      ------------------------------------------------------------
ERWSE02396      ------------------------------------------------------------
PECCP01694      ------------------------------------------------------------
PECWW01889      ------------------------------------------------------------
PECSS01870      ------------------------------------------------------------
PECAS02624      ------------------------------------------------------------
DICDC02220      ------------------------------------------------------------
DICZE02284      ------------------------------------------------------------
DICD302017      ------------------------------------------------------------
DICD502250      ------------------------------------------------------------
XENBS00829      ------------------------------------------------------------
XENNA01480      ------------------------------------------------------------
PHOLL01537      ------------------------------------------------------------
PHOAA02804      ------------------------------------------------------------
SERP501669      ------------------------------------------------------------
```

```
SERSA01625      ------------------------------------------------------------
YERPE01271      ------------------------------------------------------------
YERPS01390      ------------------------------------------------------------
YERPA00627      ------------------------------------------------------------
YERPN02489      ------------------------------------------------------------
YERPP02190      ------------------------------------------------------------
YERP302544      ------------------------------------------------------------
YERPB01460      ------------------------------------------------------------
YERPY02633      ------------------------------------------------------------
YERPG01392      ------------------------------------------------------------
YERPD01171      ------------------------------------------------------------
YERP100890      ------------------------------------------------------------
YERPZ01206      ------------------------------------------------------------
YERPH02469      ------------------------------------------------------------
YERE801438      ------------------------------------------------------------
YERE302565      ------------------------------------------------------------
YERE100392      ------------------------------------------------------------
PROMH00687      ------------------------------------------------------------
PROSM03196      ------------------------------------------------------------
TOLAT02279      ------------------------------------------------------------
AERVB02262      ------------------------------------------------------------
AERHH01811      ---------------------------------MGSAH-----WQTRLQTPFPTS-----
AERS402185      ------------------------------------------------------------
PSYIN02070      ------------------------------------------------------------
SHELP02018      ------------------------------------------------------------
SHEVD02297      ------------------------------------------------------------
SHEPW02341      ------------------------------------------------------------
SHEPA02202      ------------------------------------------------------------
SHEHH02007      ------------------------------------------------------------
SHESH02117      ------------------------------------------------------------
SHEWM02454      ------------------------------------------------------------
SHEAM01758      ------------------------------------------------------------
SHEON02159      ------------------------------------------------------------
SHESM01945      ------------------------------------------------------------
SHESR01991      ------------------------------------------------------------
SHESA02025      ------------------------------------------------------------
SHESW01953      ------------------------------------------------------------
SHEPC01976      ------------------------------------------------------------
SHEP201929      ------------------------------------------------------------
SHEB502016      ------------------------------------------------------------
SHEB802168      ------------------------------------------------------------
SHEB202117      ------------------------------------------------------------
SHEB902270      ------------------------------------------------------------
SHEB602258      ------------------------------------------------------------
SHEDO01680      ------------------------------------------------------------
SHEFN01895      ------------------------------------------------------------
PSEHT01677      ------------------------------------------------------------
ALISL01611      ------------------------------------------------------------
VIBF100893      ------------------------------------------------------------
VIBFM00891      ------------------------------------------------------------
OCESG01399      ------------------------------------------------------------
VIBA701872      ------------------------------------------------------------
VIBVY01451      ------------------------------------------------------------
VIBVU02545      ------------------------------------------------------------
VIBVM01748      ------------------------------------------------------------
VIBCH01157      ------------------------------------------------------------
VIBCM01114      ------------------------------------------------------------
VIBCJ02064      ------------------------------------------------------------
VIBC300725      ------------------------------------------------------------
FERBD01969      ------------------------------------------------------------
VIBFN02085      ------------------------------------------------------------
VIBTL01089      ------------------------------------------------------------
VIBPA01248      ------------------------------------------------------------
VIBAE02128      ------------------------------------------------------------
VIBCB00599      ------------------------------------------------------------
```

```
PANVC00701      ------------------------------------------------------------
SHIBC02443      ------------------------------------------------------------
ENTBF02792      ------------------------------------------------------------
KLEP700892      ------------------------------------------------------------
KLEPH01782      ------------------------------------------------------------
KLEP303551      ------------------------------------------------------------
KLEVT03420      ------------------------------------------------------------
ENTAK02985      ------------------------------------------------------------
KLEOK03151      ------------------------------------------------------------
SALAR01925      ------------------------------------------------------------
SALBC00812      ------------------------------------------------------------
SALPC00926      ------------------------------------------------------------
SALTI00868      ------------------------------------------------------------
SALCH00905      ------------------------------------------------------------
SALPA01706      ------------------------------------------------------------
SALTY00927      ------------------------------------------------------------
SALPK01786      ------------------------------------------------------------
SALHS00978      ------------------------------------------------------------
SALEP00854      ------------------------------------------------------------
SALDC00926      ------------------------------------------------------------
SALA400892      ------------------------------------------------------------
SALG200876      ------------------------------------------------------------
SALTS00888      ------------------------------------------------------------
SALT400905      ------------------------------------------------------------
SALPS01908      ------------------------------------------------------------
SALT101040      ------------------------------------------------------------
SALTD00960      ------------------------------------------------------------
SALPB02472      ------------------------------------------------------------
SALNS00928      ------------------------------------------------------------
SALSV01000      ------------------------------------------------------------
ECOS500812      ------------------------------------------------------------
ECOL600981      ------------------------------------------------------------
ECOL500883      ------------------------------------------------------------
ECOUT00876      ------------------------------------------------------------
ECOK100776      ------------------------------------------------------------
ECOSM02148      ------------------------------------------------------------
ECOLU01057      ------------------------------------------------------------
ECO7I02136      ------------------------------------------------------------
ECO8100828      ------------------------------------------------------------
ECO4500862      ------------------------------------------------------------
ECOAB00879      ------------------------------------------------------------
ECO4400961      ------------------------------------------------------------
ECOUM02550      ------------------------------------------------------------
ECOKI00869      ------------------------------------------------------------
ECOC100920      ------------------------------------------------------------
ECOC200920      ------------------------------------------------------------
ECO8N00803      ------------------------------------------------------------
ECO2700862      ------------------------------------------------------------
ECO2600974      ------------------------------------------------------------
ECOH100942      ------------------------------------------------------------
SHIB301848      ------------------------------------------------------------
ECOLI00846      ------------------------------------------------------------
ECO5700970      ------------------------------------------------------------
SHISS00756      ------------------------------------------------------------
SHIBS00679      ------------------------------------------------------------
SHIDS01857      ------------------------------------------------------------
ECO2400906      ------------------------------------------------------------
ECODH00784      ------------------------------------------------------------
ECOHS00922      ------------------------------------------------------------
ECOLC02627      ------------------------------------------------------------
ECO5E00946      ------------------------------------------------------------
ECOSE00936      ------------------------------------------------------------
ECO5500894      ------------------------------------------------------------
ECO8A00877      ------------------------------------------------------------
ECOBB00863      ------------------------------------------------------------
```

```
ECO5T00943      ------------------------------------------------------------
ECOBW00723      ------------------------------------------------------------
ECO1000919      ------------------------------------------------------------
ECOBD02604      ------------------------------------------------------------
ECOD102659      ------------------------------------------------------------
ECOBR00856      ------------------------------------------------------------
ECOLX02610      ------------------------------------------------------------
ECO1A00939      ------------------------------------------------------------
ECOCB01054      ------------------------------------------------------------
ECOKO02859      ------------------------------------------------------------
ECO1E03320      ------------------------------------------------------------
ECOLW01217      ------------------------------------------------------------
SHIFL01399      ------------------------------------------------------------
SHIF800759      ------------------------------------------------------------
SHIF200805      ------------------------------------------------------------
CITK802118      ------------------------------------------------------------
CITRI00924      ------------------------------------------------------------
CROS802385      ------------------------------------------------------------
CROTZ01500      ------------------------------------------------------------
ENTLS02865      ------------------------------------------------------------
ENT3801400      ------------------------------------------------------------
ENTAL01384      ------------------------------------------------------------
ENTCC02679      ------------------------------------------------------------


STRT101547      ------------------------------------------------------------
STRT201508      ------------------------------------------------------------
STRTD01356      ------------------------------------------------------------
STRTN01533      ------------------------------------------------------------
STRE500345      ------------------------------------------------------------
STRE801625      ------------------------------------------------------------
STREH01636      ------------------------------------------------------------
STREC01656      ------------------------------------------------------------
STREM01495      ------------------------------------------------------------
STRE401629      ------------------------------------------------------------
STRS700387      ------------------------------------------------------------
STRDG01631      ------------------------------------------------------------
STRP301388      ------------------------------------------------------------
STRPZ01205      ------------------------------------------------------------
STRPQ00464      ------------------------------------------------------------
STRPD01453      ------------------------------------------------------------
STRP601399      ------------------------------------------------------------
STRP801366      ------------------------------------------------------------
STRPF01453      ------------------------------------------------------------
STRPG00427      ------------------------------------------------------------
STRA300284      ------------------------------------------------------------
STRA500290      ------------------------------------------------------------
STRA100288      ------------------------------------------------------------
STRA200291      ------------------------------------------------------------
STRIC00348      ------------------------------------------------------------
STRPX00375      ------------------------------------------------------------
STRMD00432      ------------------------------------------------------------
STRS201734      ------------------------------------------------------------
STRSY01721      ------------------------------------------------------------
STRSX01553      ------------------------------------------------------------
STRSE01478      ------------------------------------------------------------
STREJ01635      ------------------------------------------------------------
STRGZ01544      ------------------------------------------------------------
STRS401593      ------------------------------------------------------------
LACGT00720      ------------------------------------------------------------
LACGL00738      ------------------------------------------------------------
STRSV01774      ------------------------------------------------------------
STRIJ00350      ------------------------------------------------------------
STROU01248      ------------------------------------------------------------
STRM601286      ------------------------------------------------------------
```

```
STRES00962      ------------------------------------------------------------
STRP701387      ------------------------------------------------------------
STRZT00756      ------------------------------------------------------------
STRP001079      ------------------------------------------------------------
STRZO01270      ------------------------------------------------------------
STRZ600815      ------------------------------------------------------------
STRET00826      ------------------------------------------------------------
STRPS01429      ------------------------------------------------------------
STRZN01287      ------------------------------------------------------------
STRR601306      ------------------------------------------------------------
STRP201217      ------------------------------------------------------------
STRZP01342      ------------------------------------------------------------
STRZI01226      ------------------------------------------------------------
STRPN01360      ------------------------------------------------------------
STRP401357      ------------------------------------------------------------
STRZJ01282      ------------------------------------------------------------
STRPJ01336      ------------------------------------------------------------
STRPI01436      ------------------------------------------------------------
MARHT00044      ------------------------------------------------------------
THEP300413      ------------------------------------------------------------
THEPX00884      ------------------------------------------------------------
THESX01857      ------------------------------------------------------------
THEM301788      ------------------------------------------------------------
THEIA01812      ------------------------------------------------------------
THETC00443      ------------------------------------------------------------
THESW01026      ------------------------------------------------------------
THEXL00360      ------------------------------------------------------------
THEID01127      ------------------------------------------------------------
THEOJ00147      ------------------------------------------------------------
DESAS01237      ------------------------------------------------------------
DESK701373      ------------------------------------------------------------
KYRT200729      ------------------------------------------------------------
STACT00410      ------------------------------------------------------------
STAS101952      ------------------------------------------------------------
STALH01969      ------------------------------------------------------------
STAEQ00422      ------------------------------------------------------------
STAES00543      ------------------------------------------------------------
STAAB00714      ------------------------------------------------------------
STAA500765      ------------------------------------------------------------
STAAM00757      ------------------------------------------------------------
STAAW00726      ------------------------------------------------------------
STAAS00733      ------------------------------------------------------------
STAAN00727      ------------------------------------------------------------
STAAC00807      ------------------------------------------------------------
STAA300727      ------------------------------------------------------------
STAA800734      ------------------------------------------------------------
STAA100752      ------------------------------------------------------------
STAA200774      ------------------------------------------------------------
STAA900759      ------------------------------------------------------------
STAAE00716      ------------------------------------------------------------
STAAT00767      ------------------------------------------------------------
STAAD00695      ------------------------------------------------------------
STAA000815      ------------------------------------------------------------
STAAH02407      ------------------------------------------------------------
STAAF00763      ------------------------------------------------------------
STAAK00744      ------------------------------------------------------------
STAAJ00703      ------------------------------------------------------------
STAAG00690      ------------------------------------------------------------
STAA400732      ------------------------------------------------------------
STAAR00788      ------------------------------------------------------------
LISSS02378      ------------------------------------------------------------
LISIN02590      ------------------------------------------------------------
LISW602421      ------------------------------------------------------------
EXISA00870      ------------------------------------------------------------
EXIS202358      ------------------------------------------------------------
```

```
EXIAB02180      ------------------------------------------------------------
OCEIH02462      ------------------------------------------------------------
BACIE01058      ------------------------------------------------------------
BACCJ03468      ------------------------------------------------------------
BACHD03507      ------------------------------------------------------------
BACPE03382      ------------------------------------------------------------
SOLSS00743      ------------------------------------------------------------
BACC600715      ------------------------------------------------------------
ANOFW02486      ------------------------------------------------------------
GEOKA03042      ------------------------------------------------------------
GEOSY02976      ------------------------------------------------------------
GEOTN02952      ------------------------------------------------------------
GEOSW02588      ------------------------------------------------------------
GEOS000381      ------------------------------------------------------------
GEOTC00378      ------------------------------------------------------------
BACMD04906      ------------------------------------------------------------
BACMQ04908      ------------------------------------------------------------
BACWK04830      ------------------------------------------------------------
BACAN04889      ------------------------------------------------------------
BACC105210      ------------------------------------------------------------
BACC705051      ------------------------------------------------------------
BACC005112      ------------------------------------------------------------
BACC305043      ------------------------------------------------------------
BACAC05232      ------------------------------------------------------------
BACAA04720      ------------------------------------------------------------
BACT005085      ------------------------------------------------------------
BACC205124      ------------------------------------------------------------
BACC405064      ------------------------------------------------------------
BACT104712      ------------------------------------------------------------
BACLD03769      ------------------------------------------------------------
BACPZ03376      ------------------------------------------------------------
BACSU03600      ------------------------------------------------------------
BACST01653      ------------------------------------------------------------
BACPT03541      ------------------------------------------------------------
LEUGG00661      ------------------------------------------------------------
LEUGJ00629      ------------------------------------------------------------
LEUCJ00485      ------------------------------------------------------------
LACAR00657      ------------------------------------------------------------
LACA300647      ------------------------------------------------------------
LACAL00643      ------------------------------------------------------------
LACKZ00964      ------------------------------------------------------------
LACRJ00357      ------------------------------------------------------------
LACRD00361      ------------------------------------------------------------
LACRS01501      ------------------------------------------------------------
LACSM00455      ------------------------------------------------------------
LACRG00878      ------------------------------------------------------------
LACRL00939      ------------------------------------------------------------
LACC300844      ------------------------------------------------------------
LACCZ00824      ------------------------------------------------------------
LACCB00988      ------------------------------------------------------------
LACCD01016      ------------------------------------------------------------
LACCC01014      ------------------------------------------------------------
LACBN01277      ------------------------------------------------------------
LACBA00605      ------------------------------------------------------------
LACPL00650      ------------------------------------------------------------
LACPJ00628      ------------------------------------------------------------
LACPS00585      ------------------------------------------------------------
PEDCP00497      ------------------------------------------------------------
CARS100359      ------------------------------------------------------------
AERUA00263      ------------------------------------------------------------
ELUMP00590      ------------------------------------------------------------
SPHPG02940      ------------------------------------------------------------
SPHGB01868      ------------------------------------------------------------
TREPZ00273      ------------------------------------------------------------
TREAZ03414      ------------------------------------------------------------
```

SPITD00734 --
SPITZ01373 --
TREPA00803 --
TREPS00802 --
TREPC00747 --
TREPM00823 --
TREPD00824 --
TREPU00781 --
ENCCU00216 --
HELM100964 --
HELCP01490 --
ARCFU01526 --
FERPA02445 --
ARCVS01910 --
METEZ00677 --
METHD00869 --
METMA02304 --
METAC01311 --
KOSOT00298 --
MARPK01613 --
SLAHD02439 --
FILAD00976 --
BUTPB02463 --
CLOPH00250 --
CLOSW00592 --
LACFC00207 --
CRYCD00853 --
EGGLE01847 --
PYRFU01410 -----MFYGITHKCSKMLLS---------------------------RHH--QVRA----
PYRHO01476 ---M----
PYRAB00730 ----------------MLLN---------------------------IHQ--ESYV----
PYRSN00014 --
THEGJ00181 --
THEKO02097 --
THEON01610 --
THES401476 --
SYNWW02368 --
UNCTG00012 --
THEA101460 --
DENA201647 --
DEFDS00488 --
CALNY01292 --
SYNGF01652 --
DESB201123 --
DEIPM00845 ----------------MV--
DEIRA01924 --
DEIML01437 --
DEIGD02532 --
DEIDV00622 --
TRURR01079 --
THETG01863 --
THET201543 --
THET801911 --
OCEP502115 --
DEHLB00585 --
DEHMG00439 --
DEHMB00497 --
DEHMC00427 --
THELD00726 --
ANAMD00624 --
THEAS00815 --
AMICL00707 --
CLOCE01684 --
HYDS000616 --
HYDTT00224 --

```
PELTS01405      ------------------------------------------------------------
DESRL02179      ------------------------------------------------------------
BORBP00506      -----------------------------------------------------MLE----
BORAP00518      -----------------------------------------------------MLE----
BORBU00514      -----------------------------------------------------MLE----
BORBZ00490      -----------------------------------------------------MLE----
BORBN00493      -----------------------------------------------------MLE----
BORRA00486      -----------------------------------------------------MLN----
BORDL00498      -----------------------------------------------------MLN----
BORHD00497      -----------------------------------------------------MLK----
BORT900497      -----------------------------------------------------MLK----
METKA01560      ------------------------------------------------------------
MYCA500361      ------------------------------------------------------------
METVS00145      ------------------------------------------------------------
METOI01431      ------------------------------------------------------------
MYCHN00051      ------------------------------------------------------------
MYCSL00465      ------------------------------------------------------------
MYCS300420      ------------------------------------------------------------
DESK101258      -----------------------------------------------------MVV----
STAHD01472      -------------------------------------------------------M----
THEC100270      ------------------------------------------------------------
NANEQ00478      ------------------------------------------------------------
CALLD01225      ------------------------------------------------------------
SULSO02155      ------------------------------------------------------------
SULS900210      ------------------------------------------------------------
SULIA00208      ------------------------------------------------------------
SULIM00207      ------------------------------------------------------------
SULIK00225      ------------------------------------------------------------
IGNH400907      ------------------------------------------------------------
KORCO01040      ------------------------------------------------------------
THESM01133      ------------------------------------------------------------
THEBM01534      ------------------------------------------------------------
METST01363      ------------------------------------------------------------
METTH00703      ------------------------------------------------------------
METSL02406      ------------------------------------------------------------
METLA02406      ------------------------------------------------------------
METPW00194      ------------------------------------------------------------
RUBXD00226      ------------------------------------------------------------
RHOM400178      ------------------------------------------------------------
GARV400051      ------------------------------------------------------------
BIFAP01644      ------------------------------------------------------------
SEGRD00014      ------------------------------------------------------------
GORB404535      ------------------------------------------------------------
GORPV04920      ------------------------------------------------------------
MYCA904903      ------------------------------------------------------------
MYCSS05356      ------------------------------------------------------------
MYCSJ05684      ------------------------------------------------------------
MYCSK05387      ------------------------------------------------------------
MYCS206581      ------------------------------------------------------------
MYCCN05158      ------------------------------------------------------------
MYCVP05890      ------------------------------------------------------------
MYCGI00775      ------------------------------------------------------------
MYCSR04994      ------------------------------------------------------------
AMYSD04545      ------------------------------------------------------------
MYCLE02681      ------------------------------------------------------------
MYCLB02681      ------------------------------------------------------------
MYCSD04323      ------------------------------------------------------------
MYCPA04306      ------------------------------------------------------------
MYCA105023      ------------------------------------------MPAVLFAT---AGNSH--
MYCUA04107      ------------------------------------------------------------
MYCMM05378      ------------------------------------------------------------
MYCA003905      ------------------------------------------------------------
MYCTU03943      ------------------------------------------------------------
MYCTF03846      ------------------------------------------------------------
MYCTA03980      ------------------------------------------------------------
```

```
MYCTK04010      ------------------------------------------------------------
MYCTC03612      ------------------------------------------------------------
MYCTD03548      ------------------------------------------------------------
MYCCP03898      ------------------------------------------------------------
MYCBO02863      ------------------------------------------------------------
MYCBP03913      ------------------------------------------------------------
MYCBT03913      ------------------------------------------------------------
TSUPD04078      ------------------------------------------------------------
ACTMD06897      ------------------------------------------------------------
SACES08402      ------------------------------------------------------------
SACVD03814      ------------------------------------------------------------
AMYMU09190      ------------------------------------------------------------
AMYMS10178      ------------------------------------------------------------
PSEUX06421      ------------------------------------------------------------
NOCFA05681      ------------------------------------------------------------
NOCCG05476      ------------------------------------------------------------
RHOE406010      ------------------------------------------------------------
RHOOB03468      ------------------------------------------------------------
RHOE104515      ------------------------------------------------------------
CORDI02302      ------------------------------------------------------------
CORD202219      ------------------------------------------------------------
CORDL02208      ------------------------------------------------------------
CORDJ02208      ------------------------------------------------------------
CORDH02221      ------------------------------------------------------------
CORD702316      ------------------------------------------------------------
CORD302337      ------------------------------------------------------------
CORDD02235      ------------------------------------------------------------
CORDV02170      ------------------------------------------------------------
CORDW02254      ------------------------------------------------------------
CORDK02230      ------------------------------------------------------------
COREF02870      ------------------------------------------------------------
CORGL03082      ------------------------------------------------------------
CORGK02974      ------------------------------------------------------------
CORGB03038      ------------------------------------------------------------
CORK402006      ------------------------------------------------------------
CORJK02028      ------------------------------------------MNQPFLQA---SGILN-N
CORVD02951      ------------------------------------------------------------
ARCHD01706      ------------------------------------------------------------
THET101205      ------------------------------------------------------------
PROAC02247      ------------------------------------------MTTQALTP---NTILG-N
PROAS02302      ------------------------------------------MTTQALTP---NTILG-N
CAERE29798      ------------------------------------------------------------
CELFA03749      ------------------------------------------------------------
JONDD02484      ------------------------------------------------------------
XYLCX03324      ------------------------------------------------------------
ACIC102144      ------------------------------------------------------------
FRADG04042      ------------------------------------------------------------
FRASU07065      ------------------------------------------------------------
FRASN07115      ------------------------------------------------------------
FRASC04424      ------------------------------------------------------------
FRAAA06712      ------------------------------------------------------------
KYTSD02521      -----------------------------------------------------MSIFT-Q
KINRD04462      ------------------------------------------------------------
NOCDD04789      ------------------------------------------------------------
NOCAA01941      ------------------------------------------------------------
STRRD08913      ------------------------------------------------------------
THECD04863      ------------------------------------------------------------
THEBD03528      ------------------------------------------------------------
CATAD08894      ------------------------------------------------------------
KRIFD06917      ------------------------------------------------------------
KITSK03730      ------------------------------------------------------------
STRBB05366      ------------------------------------------------------------
STRVP03660      ------------------------------------------------------------
STRSW04381      ------------------------------------------------------------
STRGG03562      ------------------------------------------------------------
```

```
STRFA03098      ------------------------------------------------------------
STRAW04303      ------------------------------------------------------------
STRCO02929      ------------------------------------------------------------
STRHJ05105      ------------------------------------------------------------
MONBE04991      ------------------------------------------------------------
CHLRE01313      ------------------------------------------------------------
MEDTR25591      --------------------------------MKFRFCPNNNWITLFNKA---RNLISTQ
SOLLC13750      --------------------------------MTGNCW-P-KLINTFKKA---CNLIPVA
PRUPE10733      --------------------------------MKFYPN-PNKVLSLLRRA---RSAVGVA
MANES18605      ----------------------------------MNRT-PSRLRSLLKRA---RSFIGLA
THECC00884      --------------------------------MNYRSN-PSRLKTLWRKA---RTLVGLA
PHYPA31147      GLPFFLSRGFAAVCQAALL-------FVAWRPSSLSPPPPPFYMLLLRHS---RLHL---
AMBTC19471      SSA--SA----SACASASASASASAS----ASASASA----------------SASLTFS
MUSAC26038      --------------------------------------LRSKLSAL---L---KNACSFV
MUSAM33177      --------------------------------------LRSKLSAL---L---KNACSFV
SETIT03079      TSYYFVQ----QSCSASLEGPNKKLNKHAYHRHNKSAPYGSQFARTMRRI---SVISEFR
ORYBR12195      ------------------------------------------------------------
COCLU07729      ------------------------------------------------------------
PHANO13702      ------------------------------------------------------------
PHAND10804      ------------------------------------------------------------
AURPU02089      ------------------------------------------------------------
ZYMTR07711      ------------------------------------------------------------
DICPU05926      ------------------------------------------------------------
ENTHI00522      ------------------------------------------------------------
LEPBA02231      ------------------------------------------------------------
LEPBP02301      ------------------------------------------------------------
LEPBL01462      ------------------------------------------------------------
LEPBJ01276      ------------------------------------------------------------
LEPIN02475      ------------------------------------------------------------
LEPII01996      ------------------------------------------------------------
LEPIC01426      ------------------------------------------------------------
SPIAZ00697      ------------------------------------------------------------
PENRW10140      ------------------------------------------------------------
PENCH09104      ------------------------------------------------------------
EURHE07269      ------------------------------------------------------------
ASPAC07301      ------------------------------------------------------------
EMENI10387      ------------------------------------------------------------
EMEND02596      ------------------------------------------------------------
ASPTN06742      ------------------------------------------------------------
ASPCL04014      ------------------------------------------------------------
ASPFU05647      -----------------------------------------------------MV-----
NEOFI00452      ------------------------------------------------------------
CRYPA10563      ------------------------------------------------------------
BLUGR03498      ------------------------------------------------------------
SCLS112814      ------------------------------------------------------------
MAGGR04266      ------------------------------------------------------------
NEUCR01575      ------------------------------------------------------------
NEUT908941      ------------------------------------------------------------
VERDA02342      ------------------------------------------------------------
COLSU12486      ------------------------------------------------------------
HYPAI01684      ------------------------------------------------------------
HYPVG06080      ------------------------------------------------------------
HYPJE05895      ------------------------------------------------------------
NECHA05020      ------------------------------------------------------------
FUSO415847      ------------------------------------------------------------
GIBZA01026      ------------------------------------------------------------
SCHPO04025      ------------------------------------------------------------
YARLI03635      ------------------------------------------------------------
ASHGO00946      ------------------------------------------------------------
KLULA02190      -------------------------------------------MLLVRNS---TL-----
ZYGRO00676      -------------------------------------------MLV-------NS-----
DEKBR01813      ------------------------------------------------------------
PICPG04776      ------------------------------------------------------------
CANTE00916      ------------------------------------------------------------
LODEL03891      ------------------------------------------------------------
```

```
DEBHA05546      ------------------------------------------------------------
SPAPN03477      ------------------------------------------------------------
CANAW04800      ------------------------------------------------------------
PICST04701      ------------------------------------------------------------
PUCGT10887      -----------------------------------------------------MA-----
PUCGR11813      -----------------------------------------------------MA-----
PHYBL11006      ------------------------------------------------------------
USTMA03757      ------------------------------------------------------------
USTHO04132      ------------------------------------------------------------
WALSE04527      ------------------------------------------------------------
TREME07701      -----------------------------------------------------MA-----
AURST04751      ------------------------------------------------------------
FOMME10177      ------------------------------------------------------------
CONPW06392      ------------------------------------------------------------
STEHR07076      --------------------------------MGRSSEEKLPWHFIVARY---AT-----
HETAN06295      ------------------------------------------------------------
GLOTR06982      ------------------------------------------------------------
PUNST01981      -----------------------------------------------------MA-----
LACBI02877      ------------------------------------------------------------
COPCI16429      ------------------------------------------------------------
DICSQ11618      -----------------------------------------------------MA-----
TRAVS13180      -----------------------------------------------------MA-----
WOLCO03584      -----------------------------------------------------MA-----
FOMPI05979      -----------------------------------------------------MA-----
PHLGI10219      ------------------------------------------------------------
PHACH05757      ------------------------------------------------------------
RICTY00419      ------------------------------------------------------------
RICPR00429      ------------------------------------------------------------
RICPP00461      ------------------------------------------------------------
RICBR00434      ------------------------------------------------------------
RICB800988      ------------------------------------------------------------
RICCK00600      ------------------------------------------------------------
RICAH00614      ------------------------------------------------------------
RICAC00782      ------------------------------------------------------------
RICFE00656      ------------------------------------------------------------
RICMS00042      ------------------------------------------------------------
RICM500453      ------------------------------------------------------------
RICR300665      ------------------------------------------------------------
RICAG00651      ------------------------------------------------------------
RICP300630      ------------------------------------------------------------
RICRS00632      ------------------------------------------------------------
RICRO00663      ------------------------------------------------------------
RICCN00618      ------------------------------------------------------------
RICPT00620      ------------------------------------------------------------
RICAE00497      ------------------------------------------------------------
RICJY00467      ------------------------------------------------------------
RICPU00072      ------------------------------------------------------------
RICS100537      ------------------------------------------------------------
BARBK00891      ------------------------------------------------------------
BARVW00932      ------------------------------------------------------------
BART100617      ------------------------------------------------------------
BARGA00466      ------------------------------------------------------------
BARHE01107      ------------------------------------------------------------
BARQU00895      ------------------------------------------------------------
OCHA401642      ------------------------------------------------------------
BRUAB01390      ------------------------------------------------------------
BRUA201507      ------------------------------------------------------------
BRUA101339      ------------------------------------------------------------
BRUSU01458      ------------------------------------------------------------
BRUME00510      ------------------------------------------------------------
BRUSI01461      ------------------------------------------------------------
BRUC201446      ------------------------------------------------------------
BRUMC01440      ------------------------------------------------------------
BRUMB01421      ------------------------------------------------------------
BRUM501487      ------------------------------------------------------------
```

```
BRUO201285      ------------------------------------------------------------
RHILO01973      ------------------------------------------------------------
CHESB02097      ------------------------------------------------------------
METPB00996      ------------------------------------------------------------
METEP01063      ------------------------------------------------------------
METEA00810      ------------------------------------------------------------
METED01453      ------------------------------------------------------------
METS403554      ------------------------------------------------------------
METNO05482      ------------------------------------------------------------
METSZ03234      ------------------------------------------------------------
BEII900056      ------------------------------------------------------------
METSB02743      ------------------------------------------------------------
MAGMM00401      ------------------------------------------------------------
HYPNA00542      ------------------------------------------------------------
KETVY00902      ------------------------------------------------------------
KETVW00472      ------------------------------------------------------------
ROSDO03242      ------------------------------------------------------------
ROSLO02615      ------------------------------------------------------------
RUEPO00888      ------------------------------------------------------------
RUEST00613      ------------------------------------------------------------
PHAIB02390      ------------------------------------------------------------
PARDP02130      ------------------------------------------------------------
DINSH02620      ------------------------------------------------------------
RHOCB02768      ------------------------------------------------------------
RHOS500248      -----------MRASGP---------------------------------------LRQV
RHOS400150      ------------------------------------------------------------
RHOS100227      ------------------------------------------------------------
RHOSK02952      ------------------------------------------------------------
MIDMI00790      ------------------------------------------------------------
ACEP301595      ------------------------------------------------------------
MICAA01566      ------------------------------------------------------------
TISMK03676      ------------------------------------------------------------
AZOL402409      ------------------------------------------------------------
PSEUV04348      ------------------------------------------------------------
HIRBI01095      ------------------------------------------------------------
PARL102241      ------------------------------------------------------------
MARMM02139      ------------------------------------------------------------
PHEZH02588      ------------------------------------------------------------
CAUCR02826      ------------------------------------------------------------
CAUCN02939      ------------------------------------------------------------
CAUST00891      ------------------------------------------------------------
PARBH01522      ------------------------------------------------------------
PELHB02084      ------------------------------------------------------------
HYPDA03273      ------------------------------------------------------------
HYPSM04642      ------------------------------------------------------------
OLICO01107      -----------MRCGGGR----------------------FS------AF---RTDFSTV
OLICM02790      ------------------------------------------------------------
RHOPS01446      ------------------------------------------------------------
RHOPA04062      ------------------------------------------------------------
RHOPT04533      ------------------------------------------------------------
RHOPX04235      ------------------------------------------------------------
BRADU07321      ------------------------------------------------------------
BRASO01239      ------------------------------------------------------------
BRASB06352      ------------------------------------------------------------
RHOPB03773      ------------------------------------------------------------
NITWN02324      ------------------------------------------------------------
NITHX02707      ------------------------------------------------------------
AZOC501143      ------------------------------------------------------------
XANP202679      ------------------------------------------------------------
CHLTF01983      ------------------------------------------------------------
IGNAJ02236      ------------------------------------------------------------
MELRP00403      ------------------------------------------------------------
ANADF00468      ------------------------------------------------------------
CHLCH01125      ------------------------------------------------------------
PELPB01419      ------------------------------------------------------------
```

```
CHLL200781      ------------------------------------------------------------
CHLTE00830      ------------------------------------------------------------
CHLP800854      ------------------------------------------------------------
CHLL701225      ------------------------------------------------------------
CHLPM00993      ------------------------------------------------------------
WOLTR00562      ------------------------------------------------------------
WOLPP00135      ------------------------------------------------------------
WOLPM00650      ------------------------------------------------------------
WOLWR00416      ------------------------------------------------------------
EHRCR00696      ------------------------------------------------------------
ANAMM00345      ------------------------------------------------------------
ANAMF00341      ------------------------------------------------------------
ANAPZ00610      ------------------------------------------------------------
NEOSM00537      ------------------------------------------------------------
NEORI00513      ------------------------------------------------------------
PELUB00076      ------------------------------------------------------------
PELSM00736      ------------------------------------------------------------
PUNMI01409      ------------------------------------------------------------
ASTEC00651      ------------------------------------------------------------
ZYMMT00154      ------------------------------------------------------------
ZYMMO00984      ------------------------------------------------------------
ZYMMA00176      ------------------------------------------------------------
ZYMMN00183      ------------------------------------------------------------
SPHAL00143      ------------------------------------------------------------
SPHWW03978      ------------------------------------------------------------
SPHJU02313      ------------------------------------------------------------
NOVAD02319      ------------------------------------------------------------
ERYLH00934      ------------------------------------------------------------
GRABC00720      ------------------------------------------------------------
GLUDA03075      ------------------------------------------------------------
KOMMN00774      ------------------------------------------------------------
HALVD01097      ------------------------------------------------------------
HALHT01699      ------------------------------------------------------------
METI401038      ------------------------------------------------------------
PLAL201612      ------------------------------------------------------------
GEMAT01983      ------------------------------------------------------------
CYAAP02456      ------------------------------------------------------------
CHLPN00303      ------------------------------------------------------------
CHLPP00439      ------------------------------------------------------------
CHLPE00727      ------------------------------------------------------------
CHLTR00101      ------------------------------------------------------------
CHLTA00102      ---------MNVVCGKSR----------------------VGYSSFFCKS---REPFSCV
CHLTJ00101      ---------MNVVCGKSR----------------------VGYSSFFCKS---REPFSCV
CHLTD00098      ---------MNVVCGKSR----------------------VGYSSFFCKS---REPFSCV
CHLT700102      ---------MNVVCGKSR----------------------VGYSSFFCKS---REPFSCV
CHLT000101      ---------MNVVCGKSR----------------------VGYSSFFCKS---REPFSCV
CHLT500104      ---------MNVVCGKSR----------------------VGYSSFFCKS---REPFSCV
CHLTL00098      ---------MNVVCGKSR----------------------VGYSSFFCKS---REPFSCV
CHLTG00102      ---------MNVVCGKSR----------------------VGYSSFFCKS---REPFSCV
CHLTS00101      ---------MNVVCGKSR----------------------VGYSSFFCKS---REPFSCV
CHLT900102      ---------MNVVCGKSR----------------------VGYSSFFCKS---REPFSCV
CHLTZ00101      ---------MNVVCGKSR----------------------VGYSSFFCKS---REPFSCV
CHLT400432      ---------MNVVCGKSR----------------------VGYSSFFCKS---REPFSCV
CHLT100103      ---------MNVVCGKSR----------------------VGYSSFFCKS---REPFSCV
CHLT200350      ---------MNVVCGKSR----------------------VGYSSFFCKS---REPFSCV
CHLTB00350      ---------MNVVCGKSR----------------------VGYSSFFCKS---REPFSCV
CHLTC00378      ------------------------------------------------------------
SIMNZ00831      ------------------------------------------------------------
PARUW01706      ------------------------------------------------------------
WADCW00943      ------------------------------------------------------------
SINAD04980      ------------------------------------------------------------
BIFLB01558      ------------------------------------------------------------
BIFAB00498      ------------------------------------------------------------
BIFAV01561      ------------------------------------------------------------
BIFAS01557      ------------------------------------------------------------
```

```
BIFA001518      ------------------------------------------------------------
BIFBA01814      ------------------------------------------------------------
BIFAA01619      ------------------------------------------------------------
BIFDB02112      ----------------------------------------MEY------------ALSSY
TERSS03091      ------------------------------------------------------------
GRATM03160      ------------------------------------------------------------
GRAMM03836      ------------------------------------------------------------
AKKM801247      ------------------------------------------------------------
OPITP04130      ------------------------------------------------------------
CORAD01794      ------------------------------------------------------------
BUCCC00180      ------------------------------------------------------------
CENSY00347      ------------------------------------------------------------
NITMS00668      ------------------------------------------------------------
MEIRD02823      ------------------------------------------------------------
HERA203780      ------------------------------------------------------------
CHLAA02018      ------------------------------------------------------------
CHLSY02175      ------------------------------------------------------------
CHLAD02599      ------------------------------------------------------------
CALAS01745      ------------------------------------------------------------
ANATU01696      ------------------------------------------------------------
SULMS00235      ------------------------------------------------------------
BLASB00041      ------------------------------------------------------------
BLASP00551      ------------------------------------------------------------
AZOPC00437      ------------------------------------------------------------
LEPBD01878      ------------------------------------------------------------
SALRD02577      ------------------------------------------MPAPFARS---VLTFSTA
SALRM02866      ------------------------------------------MPAPFARS---VLTFSTA
RIEPU00166      ------------------------------------------------------------
ORITB00015      ------------------------------------------------------------
ORITI01234      ------------------------------------------------------------
PREMB01541      ------------------------------------------------------------
PREDF01155      ------------------------------------------------------------
PREI702011      ------------------------------------------------------------
ALIFI01035      ------------------------------------------------------------
ODOSD00362      ------------------------------------------------------------
PRER201250      ------------------------------------------------------------
PALPW00206      ------------------------------------------------------------
PORGI00953      ------------------------------------------------------------
PORG301168      ------------------------------------------------------------
BACV803426      ------------------------------------------------------------
BACT601486      ------------------------------------------------------------
BACTN04290      ------------------------------------------------------------
BACFR01032      ------------------------------------------------------------
BACFN00913      ------------------------------------------------------------
BACF600963      ------------------------------------------------------------
OWEHD03413      ------------------------------------------------------------
PSYTT00390      ------------------------------------------------------------
NONDD00011      ------------------------------------------------------------
ROBBH02711      ------------------------------------------------------------
CELAD02592      ------------------------------------------------------------
CELLC02474      ------------------------------------------------------------
MARSH03072      ------------------------------------------------------------
MURRD00159      ------------------------------------------------------------
ZOBGA00152      ------------------------------------------------------------
GRAFK00949      ------------------------------------------------------------
ZUNPS02344      ------------------------------------------------------------
AEQSU02079      ------------------------------------------------------------
HALH105745      ------------------------------------------------------------
SAPGL02893      ------------------------------------------------------------
AMOA500014      ------------------------------------------------------------
FLAIG01802      ------------------------------------------------------------
FLACA02326      ------------------------------------------------------------
FLAJ100198      ------------------------------------------------------------
FLABF01528      ------------------------------------------------------------
CAPOD01333      ------------------------------------------------------------
```

```
CAPCC00414      ------------------------------------------------------------
FLELS01898      ------------------------------------------------------------
SOLCM01166      ------------------------------------------------------------
PEDHD01598      ------------------------------------------------------------
SPHS203207      ------------------------------------------------------------
LEAB401188      ------------------------------------------------------------
EMTOG00092      ------------------------------------------------------------
SPILD01737      ------------------------------------------------------------
DYAFD00084      ------------------------------------------------------------
CYCMS03063      ------------------------------------------------------------
ECHVK03326      ------------------------------------------------------------
BELBD02542      ------------------------------------------------------------
NITGG02112      --------------------------------------------MLFT--------TSLQ
THEM700111      ------------------------------------------------------------
CREAS01350      ------------------------------------------------------------
TURPD02772      ------------------------------------------------------------
SORC507603      ------------------------------------------------------------
BDEBA00337      ------------------------------------------------------------
STIAD02752      ------------------------------------------------------------
MYXXD01876      ------------------------------------------------------------
MYXFH03454      ------------------------------------------------------------
CORCM01960      ------------------------------------------------------------
MYXSD02209      ------------------------------------------------------------
LEPFC02126      ------------------------------------------------------------
LEPFM02294      ------------------------------------------------------------
SOLUE00582      ------------------------------------------------------------
KORVE01333      ------------------------------------------------------------
ACIC502553      ------------------------------------------------------------
ACIFD00030      ------------------------------------------------------------
HALMS01402      ------------------------------------------------------------
CHLPD01133      ------------------------------------------------------------
CHLPB01137      ------------------------------------------------------------
PROA200897      ------------------------------------------------------------
WIGBR00492      ------------------------------------------------------------
BUCA500289      ------------------------------------------------------------
BUCAI00292      ------------------------------------------------------------
BUCAF00306      ------------------------------------------------------------
BUCAT00287      ------------------------------------------------------------
BUCA000302      ------------------------------------------------------------
BAUCH00290      ------------------------------------------------------------
BLOVB00369      ------------------------------------------------------------
BLOFL00367      ------------------------------------------------------------
BLOPB00375      ------------------------------------------------------------
BUCAP00289      ------------------------------------------------------------
COXBU01001      ------------------------------------------------------------
COXBN01188      ------------------------------------------------------------
COXBR01117      ------------------------------------------------------------
COXB200717      ------------------------------------------------------------
COXB100914      ------------------------------------------------------------
FRAP200241      ------------------------------------------------------------
FRANT01012      ------------------------------------------------------------
FRACN00522      ------------------------------------------------------------
FRATT00465      ------------------------------------------------------------
FRAT100465      ------------------------------------------------------------
FRATE00462      ------------------------------------------------------------
FRATW01216      ------------------------------------------------------------
FRATM01110      ------------------------------------------------------------
FRACF00566      ------------------------------------------------------------
FRATO01212      ------------------------------------------------------------
FRATH01486      ------------------------------------------------------------
FRATF01216      ------------------------------------------------------------
FRATN00564      ------------------------------------------------------------
ACIF500512      ------------------------------------------------------------
ACIF200362      ------------------------------------------------------------
DECAR01284      ------------------------------------------------------------
```

```
NEIG100525      ------------------------------------------------------------
NEIG201314      ------------------------------------------------------------
NEIM800926      ------------------------------------------------------------
NEIMP01199      ------------------------------------------------------------
NEIMB01212      ------------------------------------------------------------
NEIMF01143      ------------------------------------------------------------
NEIML01119      ------------------------------------------------------------
NEIMM00780      ------------------------------------------------------------
NEIMH00827      ------------------------------------------------------------
NEIMG01168      ------------------------------------------------------------
NEIMN01254      ------------------------------------------------------------
NEIMO00810      ------------------------------------------------------------
NEIM701229      ------------------------------------------------------------
NEIMA01334      ------------------------------------------------------------
NEIMW01125      ------------------------------------------------------------
DICNV01037      ------------------------------------------------------------
VESOH00047      ------------------------------------------------------------
RUTMC00043      ------------------------------------------------------------
HALHL02255      ------------------------------------------------------------
PELPD03177      ------------------------------------------------------------
GEOS804030      ------------------------------------------------------------
GEOBB03636      ------------------------------------------------------------
GEOSM03700      ------------------------------------------------------------
HALNC00046      ------------------------------------------------------------
HAEPS00887      ------------------------------------------------------------
GALAU02285      ------------------------------------------------------------
HISS201000      ------------------------------------------------------------
HAES101151      ------------------------------------------------------------
PASMU00573      ------------------------------------------------------------
PASMH00579      ------------------------------------------------------------
ACTSZ01560      ------------------------------------------------------------
AGGAN00985      ------------------------------------------------------------
NITHN03181      ------------------------------------------------------------
NITOC00311      ------------------------------------------------------------
NITWC02498      ------------------------------------------------------------
METNJ00648      ------------------------------------------------------------
METFJ01616      ------------------------------------------------------------
ALKEH00244      ------------------------------------------------------------
MARMS03166      ------------------------------------------------------------
MARM102688      ------------------------------------------------------------
THICR00763      ------------------------------------------------------------
THICA00974      ------------------------------------------------------------
THIV600851      ------------------------------------------------------------
THISH01973      ------------------------------------------------------------
METAA00813      ------------------------------------------------------------
META200813      ------------------------------------------------------------
METMM02895      ------------------------------------------------------------
FRAAD00813      ------------------------------------------------------------
XYLFA01416      ------------------------------------------------------------
XYLFT00623      ------------------------------------------------------------
XYLF200667      ------------------------------------------------------------
XYLFG01622      ------------------------------------------------------------
XYLFM00690      ------------------------------------------------------------
PSEUP01680      ------------------------------------------------------------
STRM501972      ------------------------------------------------------------
PSEUU01524      ------------------------------------------------------------
XANAP01407      --------------------------------M------------VQCHDSAAISP----
XANCP01918      ------------------------------------------------------------
XANC802154      ------------------------------------------------------------
XANCB02263      ------------------------------------------------------------
XANOR02379      ------------------------------------------------------------
XANOM02295      ------------------------------------------------------------
XANOP02318      ------------------------------------------------------------
XANAC01951      ------------------------------------------------------------
CYCSP01155      ------------------------------------------------------------
```

```
GEOLS03275      ------------------------------------------------------------
GEOUR03858      ------------------------------------------------------------
GEODF00692      ------------------------------------------------------------
GEOSL00482      ------------------------------------------------------------
GEOSK00469      ------------------------------------------------------------
GEOMG02998      ------------------------------------------------------------
MORCR00228      ------------------------------------------------------------
ACIAD00798      ------------------------------------------------------------
ACIBC00821      ------------------------------------------------------------
ACIBY02725      ------------------------------------------------------------
ACIB302691      ------------------------------------------------------------
ACIB500882      ------------------------------------------------------------
ACIB100850      ------------------------------------------------------------
ACIBD00843      ------------------------------------------------------------
ACIBS02229      ------------------------------------------------------------
ACICP00137      ------------------------------------------------------------
ACISD03052      ------------------------------------------------------------
LEGLN02402      ------------------------------------------------------------
LEGPA01726      ------------------------------------------------------------
LEGPH00846      ----------------------------MFSRM------------LFCHRYFNVFIFVI-
LEGPC01171      ------------------------------------------------------------
LEGP201857      ------------------------------------------------------------
LEGPL01727      ------------------------------------------------------------
COLP302668      ------------------------------------------------------------
KANKD01106      ------------------------------------------------------------
IDILO00659      ------------------------------------------------------------
PSEU901284      ------------------------------------------------------------
PSEA602346      ------------------------------------------------------------
ALTSS01871      ------------------------------------------------------------
ALTMD01611      ------------------------------------------------------------
ALTME01705      ------------------------------------------------------------
ALTMB01797      ------------------------------------------------------------
ALTMS01652      ------------------------------------------------------------
SACD201683      ------------------------------------------------------------
TERTT01748      ------------------------------------------------------------
SIMAS00609      ------------------------------------------------------------
ALCDB01962      ------------------------------------------------------------
CHRSD02939      ------------------------------------------------------------
HALED02811      ------------------------------------------------------------
SIDLE02655      ------------------------------------------------------------
GALCS01621      ------------------------------------------------------------
LARHH01446      ------------------------------------------------------------
CHRVO02807      ------------------------------------------------------------
PSEUL01648      ------------------------------------------------------------
NITEU01859      ------------------------------------------------------------
NITEC00745      ------------------------------------------------------------
NITMU00023      ------------------------------------------------------------
NITSI03064      ------------------------------------------------------------
ACCPU02128      ------------------------------------------------------------
THIDA01000      ----------------------------------------------------MEMPLPVL
METS601221      ------------------------------------------------------------
METGS01186      ------------------------------------------------------------
METFK00972      ------------------------------------------------------------
METML01428      ------------------------------------------------------------
AROAE03899      ------------------------------------------------------------
THASP01686      ------------------------------------------------------------
AZOSB01359      ------------------------------------------------------------
BORA102682      ------------------------------------------------------------
BORPA03415      ------------------------------------------------------------
BORBM03577      ------------------------------------------------------------
BORPE02280      ------------------------------------------------------------
BORPC02060      ------------------------------------------------------------
BORP102392      ------------------------------------------------------------
BORBR03870      ------------------------------------------------------------
BORPD01524      ------------------------------------------------------------
```

```
ACHXA01179      ------------------------------------------------------------
RHOFT03114      ------------------------------------------------------------
VEREI01701      ------------------------------------------------------------
VARPE01375      ------------------------------------------------------------
VARPS01266      ------------------------------------------------------------
DELAS05272      ---------------------------------------------MYLF---------FL
DELSC01235      ------------------------------------------------------------
COMT200883      ------------------------------------------------------------
ACIAC03267      ------------------------------------------------------------
ACIAP03249      ------------------------------------------------------------
ACIET02648      ------------------------------------------------------------
ALIDK03528      ------------------------------------------------------------
RUBGI03408      ------------------------------------------------------------
LEPCP00749      ------------------------------------------------------------
POLSJ03719      -----------------------------------------------MT-----------
POLNA03144      ------------------------------------------------------------
METPP01110      ------------------------------------------------------------
RAMTT03218      ---------------------------------------------MYTG-----------
RALPJ02457      ------------------------------------------------------------
RALP102102      ------------------------------------------------------------
RALSO02303      ------------------------------------------------------------
RALS801097      ------------------------------------------------------------
HERSS01878      ------------------------------------------------------------
HERAR00955      ------------------------------------------------------------
JANMA01152      ------------------------------------------------------------
THIK102324      ------------------------------------------------------------
POLSQ00682      ------------------------------------------------------------
POLNS00989      ------------------------------------------------------------
BURP800627      ------------------------------------------------------------
BURPP00988      ------------------------------------------------------------
BURSC00728      --------------------------------MRKLD-GAATRPALYNAGLIPIYSFSSK
BURXL00982      ------------------------------------------------------------
BURSG00869      ------------------------------------------------------------
BURRH00629      --------------G-----------AGVITPMQKSN-QANAGQPLYNVRSDITH---AL
BURGB00781      ------------------------------------------------------------
BURGS00853      ------------------------------------------------------------
BURPS02618      ------------------------------------------------------------
BURMA01741      ------------------------------------------------------------
BURP103016      ------------------------------------------------------------
BURP002984      ------------------------------------------------------------
BURM701866      ------------------------------------------------------------
BURP602945      ------------------------------------------------------------
BURM902465      ------------------------------------------------------------
BURMS00740      ------------------------------------------------------------
BURTA01515      ------------------------------------------------------------
BURM102386      ------------------------------------------------------------
BURL300890      --------------------------------MTAS------RSP-----RIVRR---AL
BURVG00860      --------------------------------MTAY------RST-----QTVRR---AL
BURCM00829      --------------------------------MTAS------RST-----QTVRR---AL
BURA400832      ------------------------------------------------------------
BURCA00489      ------------------------------------------------------------
BURCH00966      ------------------------------------------------------------
BURCC00927      ------------------------------------------------------------
BURCJ02922      ------------------------------------------------------------
EDWI902348      ------------------------------------------------------------
EDWTF01971      ------------------------------------------------------------
EDWTE02176      ------------------------------------------------------------
SODGM01094      ------------------------------------------------------------
MOREP00394      ------------------------------------------------------------
RAHSY01432      ------------------------------------------------------------
RAHAC01419      ------------------------------------------------------------
ERWBE01489      ------------------------------------------------------------
PANAM01339      -----------------------------MQQREFLPPFGANRRQKVQSS---STPFAVI
PANAA00662      ------------------------------------------------------------
PANSA01303      ------------------------------------------------------------
```

```
ERWT902154      -------------------------------------------------M---AQAFSLI
ERWAC01330      ------------------------------------------------------------
ERWAE01326      ------------------------------------------------------------
ERWPE02241      -----------------------------------------------------MPPFSLI
ERWP602414      ------------------------------------------------------------
ERWSE02396      ------------------------------------------------------------
PECCP01694      ------------------------------------------------------------
PECWW01889      ------------------------------------------------------------
PECSS01870      ------------------------------------------------------------
PECAS02624      ------------------------------------------------------------
DICDC02220      ------------------------------------------------------------
DICZE02284      ------------------------------------------------------------
DICD302017      ------------------------------------------------------------
DICD502250      ------------------------------------------------------------
XENBS00829      ------------------------------------------------------------
XENNA01480      ------------------------------------------------------------
PHOLL01537      ------------------------------------------------------------
PHOAA02804      ------------------------------------------------------------
SERP501669      ------------------------------------------------------------
SERSA01625      ------------------------------------------------------------
YERPE01271      ------------------------------------------------------------
YERPS01390      ------------------------------------------------------------
YERPA00627      ------------------------------------------------------------
YERPN02489      ------------------------------------------------------------
YERPP02190      ------------------------------------------------------------
YERP302544      ------------------------------------------------------------
YERPB01460      ------------------------------------------------------------
YERPY02633      ------------------------------------------------------------
YERPG01392      ------------------------------------------------------------
YERPD01171      ------------------------------------------------------------
YERP100890      ------------------------------------------------------------
YERPZ01206      ------------------------------------------------------------
YERPH02469      ------------------------------------------------------------
YERE801438      ------------------------------------------------------------
YERE302565      ------------------------------------------------------------
YERE100392      ------------------------------------------------------------
PROMH00687      ------------------------------------------------------------
PROSM03196      ------------------------------------------------------------
TOLAT02279      ------------------------------------------------------------
AERVB02262      ------------------------------------------------------------
AERHH01811      ------SIGMRATPG-----------CGAFRDRACFQPFLTPLPP--------RQP----
AERS402185      ------------------------------------------------------------
PSYIN02070      ------------------------------------------------------------
SHELP02018      ------------------------------------------------------------
SHEVD02297      ------------------------------------------------------------
SHEPW02341      ------------------------------------------------------------
SHEPA02202      ------------------------------------------------------------
SHEHH02007      ------------------------------------------------------------
SHESH02117      ------------------------------------------------------------
SHEWM02454      ------------------------------------------------------------
SHEAM01758      ------------------------------------------------------------
SHEON02159      ------------------------------------------------------------
SHESM01945      ------------------------------------------------------------
SHESR01991      ------------------------------------------------------------
SHESA02025      ------------------------------------------------------------
SHESW01953      ------------------------------------------------------------
SHEPC01976      ------------------------------------------------------------
SHEP201929      ------------------------------------------------------------
SHEB502016      ------------------------------------------------------------
SHEB802168      ------------------------------------------------------------
SHEB202117      ------------------------------------------------------------
SHEB902270      ------------------------------------------------------------
SHEB602258      ------------------------------------------------------------
SHEDO01680      ------------------------------------------------------------
SHEFN01895      ------------------------------------------------------------
```

```
PSEHT01677      ------------------------------------------------------------
ALISL01611      ------------------------------------------------------------
VIBF100893      ------------------------------------------------------------
VIBFM00891      ------------------------------------------------------------
OCESG01399      ------------------------------------------------------------
VIBA701872      ------------------------------------------------------------
VIBVY01451      ------------------------------------------------------------
VIBVU02545      ------------------------------------------------------------
VIBVM01748      ------------------------------------------------------------
VIBCH01157      ------------------------------------------------------------
VIBCM01114      ------------------------------------------------------------
VIBCJ02064      ------------------------------------------------------------
VIBC300725      ------------------------------------------------------------
FERBD01969      ------------------------------------------------------------
VIBFN02085      ------------------------------------------------------------
VIBTL01089      ------------------------------------------------------------
VIBPA01248      ------------------------------------------------------------
VIBAE02128      ------------------------------------------------------------
VIBCB00599      ------------------------------------------------------------
PANVC00701      ------------------------------------------------------------
SHIBC02443      ------------------------------------------------------------
ENTBF02792      ------------------------------------------------------------
KLEP700892      ------------------------------------------------------------
KLEPH01782      ------------------------------------------------------------
KLEP303551      ------------------------------------------------------------
KLEVT03420      ------------------------------------------------------------
ENTAK02985      ------------------------------------------------------------
KLEOK03151      ------------------------------------------------------------
SALAR01925      ------------------------------------------------------------
SALBC00812      -----------------------------------------------------------M
SALPC00926      ------------------------------------------------------------
SALTI00868      ------------------------------------------------------------
SALCH00905      ------------------------------------------------------------
SALPA01706      ------------------------------------------------------------
SALTY00927      ------------------------------------------------------------
SALPK01786      ------------------------------------------------------------
SALHS00978      ------------------------------------------------------------
SALEP00854      ------------------------------------------------------------
SALDC00926      ------------------------------------------------------------
SALA400892      ------------------------------------------------------------
SALG200876      ------------------------------------------------------------
SALTS00888      ------------------------------------------------------------
SALT400905      ------------------------------------------------------------
SALPS01908      ------------------------------------------------------------
SALT101040      ------------------------------------------------------------
SALTD00960      ------------------------------------------------------------
SALPB02472      ------------------------------------------------------------
SALNS00928      ------------------------------------------------------------
SALSV01000      ------------------------------------------------------------
ECOS500812      ------------------------------------------------------------
ECOL600981      ---------------------------------------------MTCLS---MLPIAQI
ECOL500883      ------------------------------------------------------------
ECOUT00876      ---------------------------------------------MTCLS---MLPIAQI
ECOK100776      -----------------------------------------------------MLPIAQI
ECOSM02148      ------------------------------------------------------------
ECOLU01057      ------------------------------------------------------------
ECO7I02136      ------------------------------------------------------------
ECO8100828      ------------------------------------------------------------
ECO4500862      ------------------------------------------------------------
ECOAB00879      ------------------------------------------------------------
ECO4400961      ------------------------------------------------------------
ECOUM02550      ------------------------------------------------------------
ECOKI00869      ------------------------------------------------------------
ECOC100920      ---------------------------------------------MTCLS---MLPIAQI
ECOC200920      ---------------------------------------------MTCLS---MLPIAQI
```

```
ECO8N00803      ------------------------------------------------------------
ECO2700862      ------------------------------------------------------------
ECO2600974      ------------------------------------------------------------
ECOH100942      ------------------------------------------------------------
SHIB301848      ------------------------------------------------------------
ECOLI00846      ------------------------------------------------------------
ECO5700970      ------------------------------------------------------------
SHISS00756      ------------------------------------------------------------
SHIBS00679      ------------------------------------------------------------
SHIDS01857      ------------------------------------------------------------
ECO2400906      ------------------------------------------------------------
ECODH00784      ------------------------------------------------------------
ECOHS00922      ------------------------------------------------------------
ECOLC02627      ------------------------------------------------------------
ECO5E00946      ------------------------------------------------------------
ECOSE00936      ------------------------------------------------------------
ECO5500894      ------------------------------------------------------------
ECO8A00877      ------------------------------------------------------------
ECOBB00863      ------------------------------------------------------------
ECO5T00943      ------------------------------------------------------------
ECOBW00723      ------------------------------------------------------------
ECO1000919      ------------------------------------------------------------
ECOBD02604      ------------------------------------------------------------
ECOD102659      ------------------------------------------------------------
ECOBR00856      ------------------------------------------------------------
ECOLX02610      ------------------------------------------------------------
ECO1A00939      ------------------------------------------------------------
ECOCB01054      ------------------------------------------------------------
ECOKO02859      ------------------------------------------------------------
ECO1E03320      ------------------------------------------------------------
ECOLW01217      ------------------------------------------------------------
SHIFL01399      ------------------------------------------------------------
SHIF800759      ------------------------------------------------------------
SHIF200805      ------------------------------------------------------------
CITK802118      ------------------------------------------------------------
CITRI00924      ------------------------------------------------------------
CROS802385      ------------------------------------------------------------
CROTZ01500      ------------------------------------------------------------
ENTLS02865      ------------------------------------------------------------
ENT3801400      ------------------------------------------------------------
ENTAL01384      ------------------------------------------------------------
ENTCC02679      ------------------------------------------------------------

STRT101547      -------------------------------------MYDTIVIGAGPAGMTAALYAARA
STRT201508      -------------------------------------MYDTIVIGAGPAGMTAALYAARA
STRTD01356      -------------------------------------MYDTIVIGAGPAGMTAALYAARA
STRTN01533      -------------------------------------MYDTIVIGAGPAGMTAALYAARA
STRE500345      -------------------------------------MYDTIVIGVGPAGMTAALYAARA
STRE801625      -------------------------------------MYDTIVIGAGPAGMTAALYAARA
STREH01636      -------------------------------------MYDTIVIGAGPAGMTAALYAARA
STREC01656      -------------------------------------MYDTLIIGSGPAGMTAALYAARS
STREM01495      -------------------------------------MYDTLIIGSGPAGMTAALYAARS
STRE401629      -------------------------------------MYDTLIIGSGPAGMTAALYAARS
STRS700387      -------------------------------------MYDTLIIGSGPAGMTAALYAARS
STRDG01631      -------------------------------------MYDTLIIGSGPAGMTAALYAARS
STRP301388      -------------------------------------MYDTLIIGSGPAGMTAALYAARS
STRPZ01205      -------------------------------------MYDTLIIGSGPAGMTAALYAARS
STRPQ00464      -------------------------------------MYDTLIIGSGPAGMTAALYAARS
STRPD01453      -------------------------------------MYDTLIIGSGPAGMTAALYAARS
STRP601399      -------------------------------------MYDTLIIGSGPAGMTAALYAARS
STRP801366      -------------------------------------MYDTLIIGSGPAGMTAALYAARS
STRPF01453      -------------------------------------MYDTLIIGSGPAGMTAALYAARS
STRPG00427      -------------------------------------MYDTLIIGSGPAGMTAALYAARS
STRA300284      -------------------------------------MYDTLIIGSGPGGMTAALYAARS
```

```
STRA500290      -------------------------------------MYDTLIIGSGPGGMTAALYAARS
STRA100288      -------------------------------------MYDTLIIGSGPGGMTAALYAARS
STRA200291      -------------------------------------MYDTLIIGSGPGGMTAALYAARS
STRIC00348      -------------------------------------MYDTLIIGSGPAGMTAGLYAARA
STRPX00375      -------------------------------------MYDTLIIGSGPAGMTAGLYAARS
STRMD00432      -------------------------------------MYDTLIIGSGPAGMTAGLYAARS
STRS201734      -------------------------------------MYDTVVIGAGPAGMTAALYAGRS
STRSY01721      -------------------------------------MYDTVVIGAGPAGMTAALYAGRS
STRSX01553      -------------------------------------MYDTVVIGAGPAGMTAALYAGRS
STRSE01478      -------------------------------------MYDTVVIGAGPAGMTAALYAGRS
STREJ01635      -------------------------------------MYDTVVIGAGPAGMTAALYAGRS
STRGZ01544      -------------------------------------MYDTVVIGAGPAGMTAALYAGRS
STRS401593      -------------------------------------MYDTVVIGAGPAGMTAALYAGRS
LACGT00720      ---------------------------------MTEKIYDVVVIGAGPAGMTAAMYSARS
LACGL00738      ---------------------------------MTEKIYDVVVIGAGPAGMTAAMYSARS
STRSV01774      -------------------------------------MYDTIIIGAGPAGMTAALYAARS
STRIJ00350      -------------------------------------MYDTIIIGAGPAGITAALYAARS
STROU01248      -------------------------------------MYDTIIIGAGPAGMTAALYAARS
STRM601286      -------------------------------------MYDTIIIGAGPAGMTAALYAARS
STRES00962      -------------------------------------MYDTIIIGAGPAGMTAALYAARS
STRP701387      -------------------------------------MYDTIIIGAGPAGMTAALYAARS
STRZT00756      -------------------------------------MYDTIIIGAGPAGMTAALYAARS
STRP001079      -------------------------------------MYDTIIIGAGPAGMTAALYAARS
STRZO01270      -------------------------------------MYDTIIIGAGPAGMTAALYAARS
STRZ600815      -------------------------------------MYDTIIIGAGPAGMTAALYAARS
STRET00826      -------------------------------------MYDTIIIGAGPAGMTAALYAARS
STRPS01429      -------------------------------------MYDTIIIGAGPAGMTAALYAARS
STRZN01287      -------------------------------------MYDTIIIGAGPAGMTAALYAARS
STRR601306      -------------------------------------MYDTIIIGAGPAGMTAALYAARS
STRP201217      -------------------------------------MYDTIIIGAGPAGMTAALYAARS
STRZP01342      -------------------------------------MYDTIIIGAGPAGMTAALYAARS
STRZI01226      -------------------------------------MYDTIIIGAGPAGMTAALYAARS
STRPN01360      -------------------------------------MYDTIIIGAGPAGMTAALYAARS
STRP401357      -------------------------------------MYDTIIIGAGPAGMTAALYAARS
STRZJ01282      -------------------------------------MYDTIIIGAGPAGMTAALYAARS
STRPJ01336      -------------------------------------MYDTIIIGAGPAGMTAALYAARS
STRPI01436      -------------------------------------MYDTIIIGAGPAGMTAALYAARS
MARHT00044      -------------------MEFK--IGEHGGTKPQDDAYDVVIIGGGPAGLTAGIYTGRA
THEP300413      -------------------------------------MYDLIILGGGPAGLTAGLYAARS
THEPX00884      -------------------------------------MYDLIILGGGPAGLTAGLYAARS
THESX01857      -------------------------------------MYDLIILGGGPAGLTAGLYAARS
THEM301788      -------------------------------------MYDLIILGGGPAGLTAGLYAARS
THEIA01812      -------------------------------------MYDLIILGGGPAGLTAGLYAARS
THETC00443      -------------------------------------MYDLIILGGGPAGLAAGLYACRS
THESW01026      -------------------------------------MYDLIILGGGPAGLAAGLYACRS
THEXL00360      -------------------------------------MYDLIILGGGPAGLAAGLYACRS
THEID01127      ----------------------------------MSDIFDLAIIGGGPAGLTAYLYAARA
THEOJ00147      -------------------------------------MYDLAIIGAGPAGLSAAIYGARA
DESAS01237      ----------------------------------MDNLKDLIIIGGGPGGLAAGIYAARA
DESK701373      -------------------------------------MYDVAIIGGGPAGLTAGIYAARA
KYRT200729      ------------------------------------MVYDTIILGGGPAGLSAAVYALRS
STACT00410      -------------------------------MAIQQPDYDVVIIGAGPAGMTAAVYASRA
STAS101952      --------------------------------MAEQVDFDIAIIGAGPAGMTAAVYASRA
STALH01969      ---------------------------------MTEVNYDVAIIGAGPAGMTAAVYASRA
STAEQ00422      ---------------------------------MTEVDFDVAIIGAGPAGMTAAVYASRA
STAES00543      ---------------------------------MTEVDFDVAIIGAGPAGMTAAVYASRA
STAAB00714      ---------------------------------MTEIDFDIAIIGAGPAGMTAAVYASRA
STAA500765      ---------------------------------MTEIDFDIAIIGAGPAGMTAAVYASRA
STAAM00757      ---------------------------------MTEIDFDIAIIGAGPAGMTAAVYASRA
STAAW00726      ---------------------------------MTEIDFDIAIIGAGPAGMTAAVYASRA
STAAS00733      ---------------------------------MTEIDFDIAIIGAGPAGMTAAVYASRA
STAAN00727      ---------------------------------MTEIDFDIAIIGAGPAGMTAAVYASRA
STAAC00807      ---------------------------------MTEIDFDIAIIGAGPAGMTAAVYASRA
STAA300727      ---------------------------------MTEIDFDIAIIGAGPAGMTAAVYASRA
STAA800734      ---------------------------------MTEIDFDIAIIGAGPAGMTAAVYASRA
```

```
STAA100752      ---------------------------------MTEIDFDIAIIGAGPAGMTAAVYASRA
STAA200774      ---------------------------------MTEIDFDIAIIGAGPAGMTAAVYASRA
STAA900759      ---------------------------------MTEIDFDIAIIGAGPAGMTAAVYASRA
STAAE00716      ---------------------------------MTEIDFDIAIIGAGPAGMTAAVYASRA
STAAT00767      ---------------------------------MTEIDFDIAIIGAGPAGMTAAVYASRA
STAAD00695      ---------------------------------MTEIDFDIAIIGAGPAGMTAAVYASRA
STAA000815      ---------------------------------MTEIDFDIAIIGAGPAGMTAAVYASRA
STAAH02407      ---------------------------------MTEIDFDIAIIGAGPAGMTAAVYASRA
STAAF00763      ---------------------------------MTEIDFDIAIIGAGPAGMTAAVYASRA
STAAK00744      ---------------------------------MTEIDFDIAIIGAGPAGMTAAVYASRA
STAAJ00703      ---------------------------------MTEIDFDIAIIGAGPAGMTAAVYASRA
STAAG00690      ---------------------------------MTEIDFDIAIIGAGPAGMTAAVYASRA
STAA400732      ---------------------------------MTEIDFDIAIIGAGPAGMTAAVYASRA
STAAR00788      ---------------------------------MTEIDFDIAIIGAGPAGMTAAVYASRA
LISSS02378      -------------------------------MASEGKIYDVIIIGAGPAGMTAALYTSRA
LISIN02590      -------------------------------MASEEKIYDVIIIGAGPAGMTAALYTSRA
LISW602421      -------------------------------MASEEKIYDVIIIGAGPAGMTAALYTSRA
EXISA00870      --------------------------------MTEEKIYDVIIIGAGPAGMTAALYASRA
EXIS202358      ------------------------------MTETEQKIYDVIIIGAGPAGMTAALYASRA
EXIAB02180      ------------------------------MTGTDQKIYDVIIIGAGPAGMTAALYASRA
OCEIH02462      --------------------------------MSEEKMYDVIIAGAGPAGMTAAVYASRA
BACIE01058      --------------------------------MSEERIYDVVIIGAGPAGMTAAVYTSRA
BACCJ03468      --------------------------------MSEEKIYDVAIIGAGPAGMTAAVYTSRA
BACHD03507      -------------------------------MGEEQKVYDVVIAGAGPAGMTAAVYTSRA
BACPE03382      --------------------------------MSEEKVYDVIIAGAGPAGMTAAVYTSRA
SOLSS00743      --------------------------------MSEEKIYDVVIIGAGPAGMTAAVYASRA
BACC600715      --------------------------------MSEEQIYDVIIIGAGPAGMTAAVYASRS
ANOFW02486      MES----------------DAYR--KGE--KNVSQENIYDVIIIGAGPAGMTAAVYTSRA
GEOKA03042      --------------------------------MADEKIYDVIIAGAGPAGLTAAVYTSRA
GEOSY02976      --------------------------------MADEKIYDVIIAGAGPAGLTAAVYTSRA
GEOTN02952      --------------------------------MADEKIYDVIIAGAGPAGLTAAVYTSRA
GEOSW02588      --------------------------------MSEEKIYDVIIAGAGPAGMTAAVYTSRA
GEOS000381      --------------------------------MSEEKIYDVIIAGAGPAGMTAAVYTSRA
GEOTC00378      --------------------------------MSEEKIYDVIIAGAGPAGMTAAVYTSRA
BACMD04906      --------------------------------MTEEKIYDVIIIGAGPAGMTAAVYTSRA
BACMQ04908      --------------------------------MTEEKIYDVIIIGAGPAGMTAAVYTSRA
BACWK04830      --------------------------------MSEEKIYDVIIIGAGPAGMTAAVYTSRA
BACAN04889      --------------------------------MSEEKIYDVIIIGAGPAGMTAAVYTSRA
BACC105210      --------------------------------MSEEKIYDVIIIGAGPAGMTAAVYTSRA
BACC705051      --------------------------------MSEEKIYDVIIIGAGPAGMTAAVYTSRA
BACC005112      --------------------------------MSEEKIYDVIIIGAGPAGMTAAVYTSRA
BACC305043      --------------------------------MSEEKIYDVIIIGAGPAGMTAAVYTSRA
BACAC05232      --------------------------------MSEEKIYDVIIIGAGPAGMTAAVYTSRA
BACAA04720      --------------------------------MSEEKIYDVIIIGAGPAGMTAAVYTSRA
BACT005085      --------------------------------MSEEKIYDVIIIGAGPAGMTAAVYTSRA
BACC205124      --------------------------------MSEEKIYDVVIIGAGPAGMTAAVYTSRA
BACC405064      --------------------------------MSEEKIYDVVIIGAGPAGMTAAVYTSRA
BACT104712      --------------------------------MSEEKIYDVVIIGAGPAGMTAAVYTSRA
BACLD03769      --------------------------------MSEEKMYDVIIIGAGPAGMTAAVYTSRA
BACPZ03376      --------------------------------MSEEKVYDVIIIGAGPAGMTAAVYTSRA
BACSU03600      --------------------------------MSEEKIYDVIIIGAGPAGMTAAVYTSRA
BACST01653      --------------------------------MSEEKIYDVIIIGAGPAGMTAAVYTSRA
BACPT03541      --------------------------------MSEEKIYDVIIIGAGPAGMTAAVYTSRA
LEUGG00661      --------------------------------MAKDKQYDVVIIGAGPAGMTAATYASRA
LEUGJ00629      --------------------------------MAKDKQYDVVIIGAGPAGMTAATYASRA
LEUCJ00485      --------------------------------MVEYKQYDVIIIGAGPAGMTAATYASRA
LACAR00657      ----------------------------------MADKYDAIVIGAGPGGMTSALYATRA
LACA300647      ----------------------------------MADKYDAIVIGAGPGGMTSALYATRA
LACAL00643      ----------------------------------MADKYDAIVIGAGPGGMTSALYATRA
LACKZ00964      ----------------------------------MADKYDVIVVGAGPGGMTAALYAARA
LACRJ00357      --------------------------------MAESKQYDVVIIGAGPGGMTAAMYASRA
LACRD00361      --------------------------------MAESKQYDVVIIGAGPGGMTAAMYASRA
LACRS01501      --------------------------------MAENKQYDVVIIGAGPGGMTAAMYASRA
LACSM00455      ----------------------------------MTKKYDVIVIGAGPAGMTAALYASRA
LACRG00878      ----------------------------------MAKKYDVIVIGAGPGGMTAALYASRA
```

```
LACRL00939      ----------------------------------MAKKYDVIVIGAGPGGMTAALYASRA
LACC300844      ----------------------------------MAKKYDVIVIGAGPGGMTAALYASRA
LACCZ00824      ----------------------------------MAKKYDVIVIGAGPGGMTAALYASRA
LACCB00988      ----------------------------------MAKKYDVIVIGAGPGGMTAALYASRA
LACCD01016      ----------------------------------MAKKYDVIVIGAGPGGMTAALYASRA
LACCC01014      ----------------------------------MAKKYDVIVIGAGPGGMTAALYASRA
LACBN01277      ----------------------------------MAKQYDVVIIGAGPGGMTAALYASRA
LACBA00605      -----------------------------------MKSYDVIVIGAGPGGMTGALYASRA
LACPL00650      ----------------------------------MAKSYDVIIIGAGPAGMTAALYASRA
LACPJ00628      ----------------------------------MAKSYDVIIIGAGPAGMTAALYASRA
LACPS00585      ----------------------------------MAKSYDVIIIGAGPAGMTAALYASRA
PEDCP00497      ----------------------------------MAKEYDIIIIGAGPAGMTAALYASRA
CARS100359      -------------------------------METEEKIYDVVVIGSGPAGMTAALYASRS
AERUA00263      -------------------------------MSEEVKTYDVIVIGAGPAGLTAALYASRA
ELUMP00590      -----------------------------------MQTSDIIVIGNGPAGVTAAIYGVRA
SPHPG02940      -----------------------------------MVEQDILIIGSGVAGMAAAQYGARA
SPHGB01868      -----------------------------------MIEQDILIIGSGVAGMSAAQYAARA
TREPZ00273      ---------------------------------MAQLEADLLILGAGPAGLSAAQYGSRA
TREAZ03414      ---------------------------------MTETDADLIIIGAGPAGLAAAQYGARA
SPITD00734      ----------------------------------MTPDRDVIIVGAGPAGLAAAQYAARA
SPITZ01373      ----------------------------------MTPDHDVIIVGAGPAGLAAAQYAARA
TREPA00803      ----------------------------------METDYDVIIVGAGAAGLSAAQYACRA
TREPS00802      ----------------------------------METDYDVIIVGAGAAGLSAAQYACRA
TREPC00747      ----------------------------------METDYDVIIVGAGAAGLSAAQYACRA
TREPM00823      ----------------------------------METDYDVIIVGAGAAGLSAAQYACRA
TREPD00824      ----------------------------------METDYDVIIVGAGAAGLSAAQYACRA
TREPU00781      ----------------------------------MKTDYDVIIVGAGAAGLSAAQYACRA
ENCCU00216      -------------------------------------MENIVIIGSGPAAYNAALYAMDK
HELM100964      -------------------------------------MIDLAIIGGGPAGLSAGLYATRG
HELCP01490      -------------------------------------MIDVAIIGGGPAGLSAGLYATRG
ARCFU01526      -------------------------------------MYDVAIIGGGPAGLTAALYSARY
FERPA02445      -ME----------------AI-----LDIEGEKSEEREFDVAIIGAGPAGLTAAIYATRY
ARCVS01910      ------------------------------------MEYDVAIIGAGPAGLTAAIYAGRY
METEZ00677      -------------------------------------MNDLIIIGGGPAGIAAGIYAVRY
METHD00869      -------------------------------------MYDLIIIGGGPAGLTAGIYAVRY
METMA02304      -------------------------------------MYDLIIIGGGPAGLTAGIYAVRY
METAC01311      --------------------MNS-------FLFSGGNMYDLIIIGGGPAGLAAGIYAVRF
KOSOT00298      --------------------MAFFDLGSAKQKSEVKDYYDILIIGGGPGGITAGIYAVQA
MARPK01613      ---------------------MFFDLGSSK-KHDLKEYYDMVIIGGGPAGVAAGIYAVQG
SLAHD02439      ----------------------------------MENVYDVIILGAGPAGLAAGLYAGRS
FILAD00976      ----------------------------------MKQIYDVIIIGSGPAGLSAGLYAGRA
BUTPB02463      -------------------------------------MYDLVIIGSGPAGLSAAVYAKRA
CLOPH00250      ---------------------------------MNKIIHDLAIIGSGPAGLTASIYASRA
CLOSW00592      ----------------------------------MNEIYDVVIIGSGPAGLSAAVYGKRA
LACFC00207      ------------------------------MAEEKQMNYDVVVIGAGPGGMTAAMYASRA
CRYCD00853      -----------------------------------METFDIAVIGAGPAGMTAALYSARA
EGGLE01847      MTE----------------ETASNETSAACKAASTNDVIDVAVIGAGPAGLTAGLYAARA
PYRFU01410      GGE----------------DMFSLG-GLTKSSVDTSKVWDVIIIGAGPAGYTAAIYAARF
PYRHO01476      EVK----------------EMFSLGGGLGRSKVDESKVWDVIIIGAGPAGYTAAIYAARF
PYRAB00730      EVV----------------KMFSLG-GLGKSRVDESKVWDVIIIGAGPAGYTAAIYAARF
PYRSN00014      --------------------MFSLG-GLGRSTVDESKVWDVIIIGAGPAGYTAAIYAARF
THEGJ00181      -MG----------------EMFSLG-GFSRGGEYEKKLWDVLIIGAGPAGFTAAIYAARF
THEKO02097      --------------------MFSLG-GFSRGGEYEKKTWDVLIIGAGPAGFTAAIYAARY
THEON01610      --------------------MFSLG-GFSRGGEYEKKAWDVLIIGAGPAGFTAAIYAARF
THES401476      --------------------MFSLG-GFSRGGEYENKTWDVLIIGAGPAGFTAAIYAARF
SYNWW02368      -------------------------------------MYDVVIIGSGPAGLTAAIYTSRA
UNCTG00012      ------------------------------------MIYDVIIIGGGPAGLSAAIYASRA
THEA101460      -----------------------------------MEIYDVLIAGAGPAGLAAGIYAGRS
DENA201647      -------------------------MQDFFNVQDIKDEYDIVVLGAGPAGLTAAMYAARD
DEFDS00488      -------------------------MEDFFNFDNLKEEYDVVIIGGGPAGLTAGIYAARD
CALNY01292      -------------------------MENFFNYDDLYDIYDIVIIGGGPAGLTAGIYAARD
SYNGF01652      -------------------------------------MYDLLIVGSGPAGLTAAIYGARG
DESB201123      -----------------------------------METRDLVIVGGGPAGLTAGLYAARA
DEIPM00845      -PD----------------PAAEAGGCYLESMTEQPQQYDVIIVGGGPAGLTAAIYTGRG
DEIRA01924      ------------------------------MTAPTAHDYDVVIIGGGPAGLTAAIYTGRA
```

DEIML01437	-----------------------------------MTQYDVVIIGGGPAGLTAGIYTGRA
DEIGD02532	----------------------------------MKQNYDVVIVGGGPAGLTAAIYTGRA
DEIDV00622	----------------------------MTAHTPQTQDYDVVIVGGGPAGLTAAIYTGRA
TRURR01079	-----------------------MTDGQRDAQPTQITRADVIIVGGGPAGLTAGIYAGRA
THETG01863	---------------------MEFNLSALGSTPAAEETYDVVIIGGGPAGLTAGIYAGRA
THET201543	---------------------MEFNLSALGSTPAAEETYDVVIIGGGPAGLTAGIYAGRA
THET801911	---------------------MEFNLSALGSAPAAEETYDVVIIGGGPAGLTAGIYAGRA
OCEP502115	---------------------MDFKIGELGGSAQQDQTYDVVIIGGGPAGLTAGIYAGRG
DEHLB00585	--------------------------------MPTQLPYDIIIIGGGPAGLTAGLYTSRA
DEHMG00439	---------------------------------MSTLLYDVIIIGGGPAGLTAALYTGRS
DEHMB00497	---------------------------------MSTLLYDVIIIGGGPAGLTAALYTGRS
DEHMC00427	---------------------------------MSTLLYDVIIIGGGPAGLTAALYTGRS
THELD00726	-----------------------------------MEKRELVIIGAGPAGLTAAIYGRRS
ANAMD00624	-----------------------------------MEKHELVIVGAGPAGLTAAIYGRRA
THEAS00815	-----------------------------------MERRELVILGAGPAGLTAAIYGRRA
AMICL00707	-----------------------------------MEKRELVIIGAGPAGLTSAIYGRRA
CLOCE01684	-------------------------------------MNDVIIIGGGPAGYTAALYSSRA
HYDS000616	-------------------------------MINTHKIYDCIIVGGGPAGLTAGLYCARA
HYDTT00224	-----------------------------MLEISEDVLYDCIIIGGGPAGLTAGLYTARA
PELTS01405	-----------------------------------MEKKELVIIGGGPAGLSAGLYGSRA
DESRL02179	-----------------------------------MLEKELVIIGGGPAGYAAGLYAARA
BORBP00506	FET----------------INVNLTKKENLSQKEIEFIEDVIILGSGPAGLTAGIYSVMS
BORAP00518	FET----------------IDINLTQKENLSQKEVDFIEDVIIVGSGPAGLTAGIYSVMS
BORBU00514	FET----------------IDINLTKKKNLSQKEVDFIEDVIIVGSGPAGLTAGIYSVMS
BORBZ00490	FET----------------IDINLTKKKNLSQKEVDFIEDVIIVGSGPAGLTAGIYSVMS
BORBN00493	FET----------------IDINLTKKKNLSQKEVDFIEDVIIVGSGPAGLTAGIYSVMS
BORRA00486	FEV----------------LDIKKCNR-EIIRTEVDFVEDVIIVGSGPAGLTAGIYTVMS
BORDL00498	FEV----------------LDIKKCNR-EIIRTEVDFVEDVIIVGSGPAGLTAGIYTVMS
BORHD00497	FET----------------LDIKKSNK-RIVRTEMGSVEDVVIVGSGPAGLTAGIYTVMS
BORT900497	FEV----------------LDIKKSNK-KVLRTEMNSIEDVIIVGSGPAGLTAGIYTVMS
METKA01560	----------------------------------MAKPYDVIVIGAGPAGLSAAIHAARA
MYCA500361	-----------------------------------MAKYDVLIIGAGPAGLTAAIYLARN
METVS00145	------------------------------------MAYDLIIIGGGPAGLTAGIYAMRA
METOI01431	--------------------------MGDNMDNTYGKVYDLIIIGGGPAGLTAGIYAMRA
MYCHN00051	---------------------------MSSNSQVPDELFDLFIIGGGPASLTAAIYSARA
MYCSL00465	---------------------------------MSDRIWDVIIIGSGPAGATAAIYCARS
MYCS300420	---------------------------------MSDRIWDVIIIGSGPAGATAAIYCARS
DESK101258	FMS----------------SRFRITGLTTTI-YKSGEKYDVIVIGGGPAGLTAALYSARY
STAHD01472	TAS----------------VRFRLTGIIPTPSKKEEKTYDVVVVGGGPAGLTAALYAARY
THEC100270	MLN----------------MSFKL-KIVPIKAPTREEVYDVVVVGGGPAGLTAALYSARY
NANEQ00478	-----------------------MFRISRTSFKPLDKQWDVIIIGGGPAGVVAGLYSARY
CALLD01225	MK------------------NLSLRLGTKISRPPKGELYDVLIVGGGPAGFSAAVYASRF
SULSO02155	-----------------------MSLLPRTTSVKPGEKFDVIIVGLGPAAYGAALYSARY
SULS900210	-----------------------MSLLPRTTSVKPGEKFDVIIVGLGPAAYGAALYSARY
SULIA00208	-----------------------MSLLPRTASVKPGEKFDVIIVGLGPAAYGAALYTARY
SULIM00207	-----------------------MSLLPRTASVKPGEKFDVIIVGLGPAAYGAALYTARY
SULIK00225	-----------------------MSLLPRTASVKPGEKFDVIIVGLGPAAYGAALYTARY
IGNH400907	--------------------MLRLGA-P--AKRPKGGEFDVVVIGAGPGGLTAAMYAARL
KORCO01040	--------------------MFFM------PQARVERDNDVIIVGGGPGGLTAAIYLARY
THESM01133	-MK----------------EMFSLGS-LSQSGIDETKTWDILIIGAGPAGFTAAIYSARY
THEBM01534	--------------------MFSLTG-FS-KGKEEKTTWDVVIIGAGPAGYTAAIYTARF
METST01363	-------------------------------------MYDLVIIGAGPAGLTAGIYAGRS
METTH00703	----------------------------------MMTDYDMIVIGAGPAGLTAGIYGGRQ
METSL02406	-----------------------------------MEEYDIIIVGAGPAGLTAGIYAGRE
METLA02406	-----------------------------------MEEYDIIIVGAGPAGLTAGIYAGRE
METPW00194	-----------------------------------MEQYDIVIAGAGPAGLTAGIYAGRE
RUBXD00226	----------------------------------MSEQYDVVVVGSGPAGYTAALYAARA
RHOM400178	-------------------MTQPDLSAFEGIDFEGAEHHTLVIIGTGPAGLTAALYAARA
GARV400051	---------------------------------MENKLRKVIIIGSGPAGYTAAIYLGRA
BIFAP01644	---------------------------------MKQTVYDAIVIGSGPAGYTAAIYLGRA
SEGRD00014	---------------------------------MPETLENLIIIGSGPAGYTAAVYAARA
GORB404535	-----------------------------MSQTDTQQIHDLIIIGSGPAGYTAAVYAARA
GORPV04920	-----------------------------MTQPDNQQIHELIIVGSGPAGYTAAVYAARA
MYCA904903	-------------------------MSQAPSTGTESDVHELIIIGSGPAGYTAAVYAARA
MYCSS05356	---------------------MPVRNGRKAHMTSSSTVHDVIIIGSGPAGYTAAVYAARA

```
MYCSJ05684      ---------------------MPVRNGRKAHMTSSSTVHDVIIIGSGPAGYTAAVYAARA
MYCSK05387      ---------------------MPVRNGRKAHMTSSSTVHDVIIIGSGPAGYTAAVYAARA
MYCS206581      -------------------------------MSTSQTVHDVIIIGSGPAGYTAAIYAARA
MYCCN05158      -------------------------------MTSSPTVHDLIIIGSGPAGYTAAVYAARA
MYCVP05890      -------------------------------MTSSETIHDVIVIGSGPAGYTAAIYAARA
MYCGI00775      -------------------------------MTSSDTVHDVIIIGSGPAGYTAAVYAARA
MYCSR04994      -------------------------------MTSSDTVHDVIIIGSGPAGYTAAVYAARA
AMYSD04545      -----------------------MTTAQRSTSNESADVHDVIIIGSGPAGYTAAVYAARA
MYCLE02681      ---------------------------MNTTPSAHETIHEVIVIGSGPAGYTAALYAARA
MYCLB02681      ---------------------------MNTTPSAHETIHEVIVIGSGPAGYTAALYAARA
MYCSD04323      -------------------------------MSETPTIHDVIIIGSGPAGYTAAIYAARA
MYCPA04306      --------------------------------MTADTVHDVIIIGSGPAGYTAALYTARA
MYCA105023      PYP-----------G----VYTDPETIARKVFMTADTVHDVIIIGSGPAGYTAALYTARA
MYCUA04107      -----------------------M----TTDSSADATIHDVIVIGSGPAGYTAALYTARA
MYCMM05378      -----------------------M----TTDSSADATIHDVIVIGSGPAGYTAALYTARA
MYCA003905      --------------------MTAP----PVHDRAHHPVRDVIVIGSGPAGYTAALYAARA
MYCTU03943      --------------------MTAP----PVHDRAHHPVRDVIVIGSGPAGYTAALYAARA
MYCTF03846      --------------------MTAP----PVHDRAHHPVRDVIVIGSGPAGYTAALYAARA
MYCTA03980      --------------------MTAP----PVHDRAHHPVRDVIVIGSGPAGYTAALYAARA
MYCTK04010      --------------------MTAP----PVHDRAHHPVRDVIVIGSGPAGYTAALYAARA
MYCTC03612      --------------------MTAP----PVHDRAHHPVRDVIVIGSGPAGYTAALYAARA
MYCTD03548      --------------------MTAP----PVHDRAHHPVRDVIVIGSGPAGYTAALYAARA
MYCCP03898      --------------------MTAP----PVHDRAHHPVRDVIVIGSGPAGYTAALYAARA
MYCBO02863      --------------------MTAP----PVHDRAHHPVRDVIVIGSGPAGYTAALYAARA
MYCBP03913      --------------------MTAP----PVHDRAHHPVRDVIVIGSGPAGYTAALYAARA
MYCBT03913      --------------------MTAP----PVHDRAHHPVRDVIVIGSGPAGYTAALYAARA
TSUPD04078      ------------------------------MTAAGTDIADVIIIGSGPAGYTAGVYAGRA
ACTMD06897      ----------------------------------MSDVRNLIIVGSGPAGYTAAVYAARA
SACES08402      ----------------------------------MSEVRNLIVVGSGPAGYTAAVYAARA
SACVD03814      ---------------------------------MSEDIRNLIIVGSGPAGYTAAIYAARA
AMYMU09190      --------------------------------MAAEEIRNLIIVGSGPAGYTAAVYAARA
AMYMS10178      --------------------------------MAAEEIRNLIIVGSGPAGYTAAVYAARA
PSEUX06421      --------------------------------MSTDDVRELIIIGSGPAGYTAAVYAARA
NOCFA05681      ---------------------------------MSTPVRDLIIVGSGPAGYTAAVYAARA
NOCCG05476      ---------------------------------MSTPVRDLIIVGSGPAGYTAAVYAARA
RHOE406010      ---------------------------MTTQLETTPAVHDVIIVGSGPAGYTAGVYTARA
RHOOB03468      -------------------------------MTTPSTVHELIIVGSGPAGYTAAVYAARA
RHOE104515      -------------------------------MTTPTTVRDLIIVGSGPAGYTAGVYAARA
CORDI02302      -----------------------------MTDTTAGTIHDVAIIGSGPAGYTAALYAARA
CORD202219      -----------------------------MTDTTAGTIHDVAIIGSGPAGYTAALYAARA
CORDL02208      -----------------------------MTDTTAGTIHDVAIIGSGPAGYTAALYAARA
CORDJ02208      -----------------------------MTDTTAGTIHDVAIIGSGPAGYTAALYAARA
CORDH02221      -----------------------------MTDTTAGTIHDVAIIGSGPAGYTAALYAARA
CORD702316      -----------------------------MTDTTAGTIHDVAIIGSGPAGYTAALYAARA
CORD302337      -----------------------------MTDTTAGTIHDVAIIGSGPAGYTAALYAARA
CORDD02235      -----------------------------MTDTTAGTIHDVAIIGSGPAGYTAALYAARA
CORDV02170      -----------------------------MTDTTAGTIHDVAIIGSGPAGYTAALYAARA
CORDW02254      -----------------------------MTDTTAGTIHDVAIIGSGPAGYTAALYAARA
CORDK02230      -----------------------------MTDTTAGTIHDVAIIGSGPAGYTAALYAARA
COREF02870      ------------------------------MSEEQSKIHDVAIIGSGPAGYTAAIYAARA
CORGL03082      --------------------------MSEEQSAVAPKIHDVAIIGSGPAGYTAAVYAARA
CORGK02974      --------------------------MSEEQSAVAPKIHDVAIIGSGPAGYTAAVYAARA
CORGB03038      --------------------------MSEEQSAVAPKIHDVAIIGSGPAGYTAAVYAARA
CORK402006      ----------------------------MPCSIARMSVHDVIIVGSGPAGYTAAIYAARA
CORJK02028      DSK-----------GAADADNASATSAAGAAEAEAGKVHDVIIIGSGPAGYTAAVYAARA
CORVD02951      ------------------------------MSEQNPTLHDVIIIGSGPAGYTAAVYAARA
ARCHD01706      ----------------------------------MSTIHDVIIVGSGPAGWTAATYTGRA
THET101205      --------------------------------MAQTDTRSVIIIGSGPAGYTAAIYAARA
PROAC02247      ISL---------SDPSEIDTTQPFPETVGADVHGNDEPRDVIIVGSGPAGYTAAVYTARA
PROAS02302      ISL---------SDPSEIDTTQPFPETVGADVHGNDEPRDVIIVGSGPAGYTAAVYTARA
CAERE29798      ----------------------------------------LVIVGSGPAGYTAAVYAARA
CELFA03749      ------------------------------MSDQPSTVRDLVIVGSGPAGYTAAIYAARA
JONDD02484      --------------------------------MSDTATHDVIIIGSGPAGYTAAIYAARA
XYLCX03324      -----------------------------MTEQATPPVHDVVIVGSGPAGYTAAVYAARA
ACIC102144      ------------------------------MASESDTIRSLIIIGSGPAGYTAALYAARA
```

```
FRADG04042      MTA--------------HSSGNE----SRIPDARDPDVRDVIIIGSGPAGYTAAIYTARA
FRASU07065      ------------------------------MTAQDSGVRDVIIVGSGPAGYTAAIYTARA
FRASN07115      MTA--------------Q--GKDQAGQAAGAERSGERVLDVVIIGSGPAGYTAAIYTARA
FRASC04424      MTA--------------QEDGQDVTGRS-SQPDGDDDIRDVVIVGSGPAGYTAAVYTARA
FRAAA06712      -------------------------------------MRDVIIVGSGPAGYTAAIYTARA
KYTSD02521      TSA--------------VALNAQPATQSETSPAGEGEVREVVIVGSGPAGYTAAVYAARA
KINRD04462      -----------------------------MSDVTTNEIRNVIVVGSGPSGYTAALYAARA
NOCDD04789      ----------------------------------MSDVRNVIIIGSGPAGYTAAVYAARA
NOCAA01941      ----------------------------------MSDVRNVIIIGSGPAGYTAAVYAARA
STRRD08913      -----------------------------------MDVRNVIIIGSGPAGYTSAVYSARA
THECD04863      ----------------------------------MSDVRNVIIIGSGPAGYTAAIYAARA
THEBD03528      ----------------------------------MSDVRNVIIIGSGPAGYTAALYTARA
CATAD08894      ----------------------------------MSEIRNVIIIGSGPAGYTAAVYTARA
KRIFD06917      ---------------------------MNRASMSDPAVRNVIVIGSGPAGYTAAVYAARA
KITSK03730      ----------------------------------MSDVRNVIIIGSGPSGYTAALYTARA
STRBB05366      ----------------------------------MSDVRNVIIIGSGPAGYTAALYTARA
STRVP03660      ----------------------------------MSDVRNVIIIGSGPAGYTAALYTARA
STRSW04381      ----------------------------------MSDVRNVIIIGSGPAGYTAALYTARA
STRGG03562      ----------------------------------MSDVRNVIIIGSGPAGYTAALYTARA
STRFA03098      ----------------------------------MSDVRNVIIIGSGPAGYTAALYTARA
STRAW04303      ----------------------------------MSDVRNVIIIGSGPAGYTAALYTARA
STRCO02929      ----------------------------------MSDVRNVIIIGSGPAGYTAALYTARA
STRHJ05105      ----------------------------------MSDVRNVIIIGSGPAGYTAALYTARA
MONBE04991      --------------------------------MAEPLHTKVCIIGSGPAGHTAAIYAARA
CHLRE01313      --------------------------------MVEKMHTKVCIIGSGPAAHTAAVYTARA
MEDTR25591      RASVSSAASA------------ASATAMTDTTTLPTVKTKLCIIGSGPAAHTAAVYAARA
SOLLC13750      TLTT--------------TAAANSPKFSSMDDIHKTLKTSVCIIGSGPAAHTAAIYAARA
PRUPE10733      AAASFSSLPSSSSFPS-SSAYSSAPKLASAMDNLQTLKTKLCIIGSGPAAHTAAIYAARA
MANES18605      ATTTSLAASSLST--------SSAPKV-TTSTAMEDLKTRLCIIGSGPAAHTAAIYAARA
THECC00884      TTSAAATVTA--A--------VSFSNS-ATSAMDSPLRTKVCIIGSGPAAHTAAIYTARA
PHYPA31147      HSVVRSL-------DTCCPALAWRRVWSSSAMALEVLDTQVGIIGSGPAAHTAAVYCARA
AMBTC19471      KTITTNSASASASLTF-SKTITTNSASSGAMAGLEPLRTRVCIVGCGPAAHTAAIYSARA
MUSAC26038      GGAVASDSSAAAAAPSPHPVKPFSTATAMADAPTKTLRTRLCIIGSGPAAHTAAIYAARA
MUSAM33177      GGAVASDSSAAAAAPSPHPVKPFSTATAMADAPTKTLRTRLCIIGSGPAAHTAAIYAARA
SETIT03079      GALLDRR-------RRRRRRRQQAEQAAMEGSAAAPLRTRVCIIGSGPAAHTAAIYAARA
ORYBR12195      ----------------------------MEGSAGAPLRTRVCIIGSGPAAHTAAIYAARA
COCLU07729      ------------------------------------MHSKLVIIGSGPAGHTAAIYAARA
PHANO13702      ------------------------------------MHSKVVIIGSGPAGHTAAIYAARA
PHAND10804      ------------------------------------MHSK-VVSDSGPAGHTAAIYAARA
AURPU02089      ------------------------------------MHSKVVIIGSGPAGHTAAIYAARA
ZYMTR07711      ------------------------------------MHSKVVIIGSGPAGHTAAIYAARA
DICPU05926      ---------------------------------MSTNVEKVVIIGSGPAGHTAGIYAGRA
ENTHI00522      ----------------------------------MSNIHDVVIIGSGPAAHTAAIYLGRS
LEPBA02231      ------------------------------------MNHKVVIIGSGPAGHTAAIYAARA
LEPBP02301      ------------------------------------MNHKVVIIGSGPAGHTAAIYAARA
LEPBL01462      ------------------------------------MVHKIVIVGSGPAGHTAAIYAARA
LEPBJ01276      ------------------------------------MVHKIVIVGSGPAGHTAAIYAARA
LEPIN02475      ------------------------------------MAHKIVIIGSGPAGHTAAIYAARA
LEPII01996      ------------------------------------MAHKIVIIGSGPAGHTAAIYAARA
LEPIC01426      ------------------------------------MAHKIVIIGSGPAGHTAAIYAARA
SPIAZ00697      ----------------------------------MADTRKVIIIGSGPAAHTAAIYAARA
PENRW10140      -----------------------------------MVHSKVVIIGSGPGAHTAAIYLSRA
PENCH09104      -----------------------------------MVHSKVVIIGSGPGAHTAAIYLSRA
EURHE07269      -----------------------------------MVHTKVVIIGSGPAAHTAAVYLSRA
ASPAC07301      -----------------------------------MVHTNVVIIGSGPAAHTAAIYLSRA
EMENI10387      -----------------------------------MVHSKVVIIGSGPAAHTAAIYLSRA
EMEND02596      -----------------------------------MVHSKVVIIGSGPAAHTAAIYLSRA
ASPTN06742      -----------------------------------MVHNKVVIIGSGPAAHTAAIYLSRA
ASPCL04014      ---------------------------MAHAKVLLTSTPSFPVIGSGPAAHTAAIYLSRA
ASPFU05647      --------------HT----KVTIDCLLCWLYVLLTWYPFKIVIGSGPAAHTAAIYLSRA
NEOFI00452      -----------------------------------MVHTKVVIIGSGPAAHTAAIYLSRA
CRYPA10563      ----------------------------------MSSHTKVLVIGSGPAAHTAAIYLARA
BLUGR03498      ------------------------------------MHHKVVIIGSGPAAHTAAIYLARA
SCLS112814      ------------------------------------MHSKVVIIGSGPAAHTAAVYLARA
MAGGR04266      ------------------------------------MHSKVVIIGSGPAAHTAAIYLARA
```

```
NEUCR01575      ------------------------------------MHSKVVIIGSGPAAHTAAIYLARA
NEUT908941      ------------------------------------MHSKVVIIGSGPAAHTAAIYLARA
VERDA02342      ------------------------------------MHSKIVIIGSGPAAHTAAVYLARA
COLSU12486      ------------------------------------MHSKVVIIGSGPAAHTAAIYLARA
HYPAI01684      ------------------------------------MHSKVVVIGSGPAAHTAAIYLARA
HYPVG06080      ------------------------------------MHSKVVVIGSGPAAHTAAIYLARA
HYPJE05895      --------------------------------------MSAAVIGSGPAAHTAAIYLARA
NECHA05020      ------------------------------------MHSKVVIIGSGPAAHTAAVYLARA
FUSO415847      ------------------------------------MHSKVVIIGSGPAAHTAAVYLARA
GIBZA01026      ------------------------------------MHSKVVIIGSGPAAHTAAVYLARA
SCHPO04025      -----------------------------------MTHNKVVIIGSGPAGHTAAIYLARG
YARLI03635      -----------------------------------MTHSPVVIIGSGPAAHTAAIYLSRA
ASHGO00946      ------------------MLAAATRYRLGQALQTRMVHHKVTIIGSGPAAHTAAIYLARA
KLULA02190      --------------GRLSSLRGFFRNINESNIFYRMVHHKVTIIGSGPAAHTAAIYLARA
ZYGRO00676      --------------GRTLFSRASSTTSTTNTIIKRMVHNKVTIIGSGPAAHTAAIYLARA
DEKBR01813      -----------------------------------MTHNKVTIIGSGPAAHTAAIYLARA
PICPG04776      -----------------------------------MVHNKVTIIGSGPAAHTAAIYLARA
CANTE00916      -----------------------------------MVHNEVTIIGSGPAAHTAAIYLSRA
LODEL03891      -----------------------------------MVHHKVTIIGSGPAAHTAAIYLTRA
DEBHA05546      ---------------------MLNKFRNTAIQFKRMVHHNVTIIGSGPAAHTAAIYLSRA
SPAPN03477      -----------------------------------MTHHKVTIIGSGPAAHTAAIYLARA
CANAW04800      -----------------------------------MVHHKVTIIGSGPAAHTAAIYLARA
PICST04701      -----------------------------------MVHHRVTIIGSGPAAHTAAIYLARA
PUCGT10887      --------------PTT-------SAAEGSTNPNGKSHNKVVIIGSGPAGHTAAIYLARA
PUCGR11813      --------------PTT-------SAAEGSTNPNGKSHNKVVIIGSGPAGHTAAIYLARA
PHYBL11006      ---------------------------------MAPQHHKVVIIGSGPAGHTAAIYLARA
USTMA03757      ----------------------MTEHTNGASSSSGPKVHKVVIIGSGPAGHTAAIYLARA
USTHO04132      ----------------------MTEHVNGASSSSGQKVHKVVIIGSGPAGHTAAIYLARA
WALSE04527      --------------------------------MSERVHNRVVIIGSGPAGHTAAVYLARA
TREME07701      --------------PIPIGPEV--REPEQHGEKSKKMHSKVVIIGSGPAGHTAAIYLARA
AURST04751      ----------------------MAPIPYQPERKSTTRHSKVVIIGSGPAGHTAAIYLARA
FOMME10177      ------------------------MAPVSVNGRSSKKHSKVVVIGSGPAGHTAAIYLARA
CONPW06392      -------------------------MAPLANGKSSKMHSSVIIIGSGPAGHTAAIYLARA
STEHR07076      --------------PRLAYPPSLLFRSLHSYANLTFHPPPQVIIGSGPAGHTAAIYLARA
HETAN06295      -----------------------------------------VIIGSGPAGHTAAIYLARA
GLOTR06982      --------------------MA--PIN-GKNQKSSKMHSKVVIIGSGPAGHTAAIYLARA
PUNST01981      ---------------------P--VTN-GKGARSSKMHSKVVIIGSGPAGHTAAIYLARA
LACBI02877      ------------------------------------MHSKVVIIGSGPAGHTAAIYLARA
COPCI16429      --------------------MA--PTT-QRVEKSKKMHSKVVIIGSGPAGHTAAIYLARA
DICSQ11618      --------------PT-----N--VPP-TTVPKSNKLHSKVVIIGSGPAGHTAAIYLARA
TRAVS13180      ---------------------P--TTA-PTVPQSSKLHSKVVIIGSGPAGHTAAIYLARA
WOLCO03584      --------------PTTNGANG--TTN-GKVEKSSKMHSKVVIIGSGPAGHTAAIYLARA
FOMPI05979      --------------PTTNGANG--TNG-VHHPPSSKTHSKVVIIGSGPAGHTAAIYLARA
PHLGI10219      ------------------------------------MHSSVVIIGSGPAGHTAAIYLGRA
PHACH05757      ------------------------------------MHSKVVIIGSGPAGHTAAIYLGRA
RICTY00419      ----------------------------------MKITTKVLIIGSGPAGLSAAIYTARS
RICPR00429      ----------------------------------MKITTKVLIIGSGPAGLSAAIYTARS
RICPP00461      ----------------------------------MKITTKVLIIGSGPAGLSAAIYTARS
RICBR00434      ----------------------------------MKITTKVLIIGSGPAGLSAAIYAARA
RICB800988      ----------------------------------MKITTKVLIIGSGPAGLSAAIYAARA
RICCK00600      ----------------------------------MHITAKVLIIGSGPAGLSAAIYTARA
RICAH00614      ----------------------------------MKITTKVLIIGSGPAGLSAAIYTARA
RICAC00782      ----------------------------------MKITTKVLIIGSGPAGLSAAIYTARA
RICFE00656      ----------------------------------MKITTKVLIIGSGPAGLSAAIYTARA
RICMS00042      ----------------------------------MKITAKVLIIGSGPAGLSAAIYTARA
RICM500453      -------------------------------MIIMKITTKVLIIGSGPAGLSAAIYTARA
RICR300665      ----------------------------------MKITTKVLIIGSGPAGLSAAIYTARA
RICAG00651      ----------------------------------MKITTKVLIIGSGPAGLSAAIYTARA
RICP300630      ----------------------------------MKITTKVLIIGSGPAGLSAAIYTARA
RICRS00632      ----------------------------------MKITTKVLIIGSGPAGLSAAIYTARA
RICRO00663      ----------------------------------MKITTKVLIIGSGPAGLSAAIYTARA
RICCN00618      ----------------------------------MKITTKVLIIGSGPAGLSAAIYTARA
RICPT00620      ----------------------------------MKITTKVLIIGSGPAGLSAAIYTARA
RICAE00497      ----------------------------------MKITTKVLIIGSGPAGLSAAIYTARA
RICJY00467      ----------------------------------MKITTKVLIIGSGPAGLSAAIYTARA
```

```
RICPU00072      ----------------------------------MKITTKVLIIGSGPAGLSAAIYTARA
RICS100537      ----------------------------------MKITTKVLIIGSGPAGLSAAIYTARA
BARBK00891      ---------------------------------MERPHIRVLVIGSGPAGYTAAIYAARA
BARVW00932      ---------------------------------MVQSRIRLLIIGSGPAGYTAAIYAARA
BART100617      ---------------------------------MIQSHIRLLIIGSGPAGYTAAVYAARA
BARGA00466      ---------------------------------MVQSHIRLLIIGSGPAGYTAAIYAARA
BARHE01107      ---------------------------------MVQSHVRLLIIGSGPAGYTAAIYAARG
BARQU00895      ---------------------------------MVQPHIRLLIVGSGPAGYTAAIYAARG
OCHA401642      ---------------------------------MSQRHAPVIVIGSGPAGYTAAIYAARA
BRUAB01390      ---------------------------------MTQRHAPVIVIGSGPAGYTAAIYAARA
BRUA201507      ---------------------------------MTQRHAPVIVIGSGPAGYTAAIYAARA
BRUA101339      ---------------------------------MTQRHAPVIVIGSGPAGYTAAIYAARA
BRUSU01458      ---------------------------------MTQRHAPVIVIGSGPAGYTAAIYAARA
BRUME00510      ---------------------------------MTQRHAPVIVIGSGPAGYTAAIYAARA
BRUSI01461      ---------------------------------MTQRHAPVIVIGSGPAGYTAAIYAARA
BRUC201446      ---------------------------------MTQRHAPVIVIGSGPAGYTAAIYAARA
BRUMC01440      ---------------------------------MTQRHAPVIVIGSGPAGYTAAIYAARA
BRUMB01421      ---------------------------------MTQRHAPVIVIGSGPAGYTAAIYAARA
BRUM501487      ---------------------------------MTQRHAPVIVIGSGPAGYTAAIYAARA
BRUO201285      ---------------------------------MTQRHAPVIVIGSGPAGYAAAIYAARA
RHILO01973      ---------------------------------MTTKHAPVLIIGSGPAGYTAAVYAARA
CHESB02097      ---------------------------------MTTRHAPVLIIGSGPAGYTAAIYAARA
METPB00996      ---------------------------------MAHTHARLVIIGSGPAGYTAAIYAARA
METEP01063      ---------------------------------MAHTHARLVIIGSGPAGYTAAIYAARA
METEA00810      ---------------------------------MAHTHARLVIIGSGPAGYTAAIYAARA
METED01453      ---------------------------------MAHTHARLVIIGSGPAGYTAAIYAARA
METS403554      ---------------------------------MSTTHEKLIIIGSGPAGYTAAIYAARA
METNO05482      ---------------------------------MSITHEKLIIIGSGPAGYTAAIYAARA
METSZ03234      ----------------------------------MPQHARVVIIGSGPAGYTAAIYAARA
BEII900056      ----------------------------MSAASLSPLHARLIIIGSGPAGYTTAIYAARA
METSB02743      ---------------------------MSASPAARSSHAKVIIVGSGPAGYTAAIYAARA
MAGMM00401      --------------------------------MSEAKHHKLIILGSGPAGFTAAIYAARA
HYPNA00542      --------------------------------MAETQHAEILIIGSGPAGWTAAVYAARA
KETVY00902      --------------------------------MSNPRHTRVLIIGSGPAGYTAAVYAARA
KETVW00472      --------------------------------MSNPRHTRVLIIGSGPAGYTAAVYAARA
ROSDO03242      --------------------------------MGDTRRTRVLIIGSGPAGYTAGVYASRA
ROSLO02615      --------------------------------MGETRHTRVLIIGSGPAGYTAGVYASRA
RUEPO00888      --------------------------------MAETRHTKVLIIGSGPAGYTAGVYASRA
RUEST00613      --------------------------------MSDTRHTKVLIIGSGPAGYTAAVYASRA
PHAIB02390      --------------------------------MSDTRHTKVLIIGSGPAGYTAGVYASRA
PARDP02130      M-------------------SHAPA--EIAADTSLPAHSRVLIVGSGPAGYTAAVYAARA
DINSH02620      -------------------------------MSSETRHLKVLIIGSGPAGYTAAVYASRA
RHOCB02768      --------------------------------MSDTKHTKVLIIGSGPAGYTAAVYASRA
RHOS500248      L-------------------YEAPGKDHEGQGMSETRHTRVLIIGSGPAGYTAAVYSARA
RHOS400150      --------------------------------MAETRHTRVLIIGSGPAGYTAAVYSARA
RHOS100227      --------------------------------MAETRHTRVLIIGSGPAGYTAAVYSARA
RHOSK02952      ---------------------------MRGKAMAETRHTRVLIIGSGPAGYTAAVYSARA
MIDMI00790      ------------------------------------MHTKILIIGSGPAGYSAAIYAARS
ACEP301595      --------------------------------MAETITTDLLVIGAGPAGYTAAIYAARA
MICAA01566      ----------------------------MSTGADTATHTKVLIIGSGPAGYTAAIYAARA
TISMK03676      --------------------------------MSATHRTKVLILGSGPAGLTAAIYASRA
AZOL402409      --------------------------------MATTHHTKVLIIGAGPAGYTAAIYAARA
PSEUV04348      ---------------------------------MTVKHTKLLIVGSGPAGYTAAIYGARA
HIRBI01095      --------------------------------MSNTEHRSLIILGSGPAGYTAAIYAARA
PARL102241      --------------------------------MSEKKHSKVLIVGGGAAGYTAAIYAARA
MARMM02139      --------------------------------MSETRHSRLIIIGSGAAGYTAAIYAARA
PHEZH02588      --------------------------MSENAPRPEPRSVRCLIVGSGPAGYTAAIYAARA
CAUCR02826      MSP-----------------LRRIHTISPPMSTLSPRQTRCLIIGSGPAGYTAAIYAARA
CAUCN02939      MSP-----------------LRRIHTISPPMSTLSPRQTRCLIIGSGPAGYTAAIYAARA
CAUST00891      ------------------------------MSTTAPRQTRCLIIGSGPAGYTAAIYAARA
PARBH01522      --------------------------------MADRLHTKLLIIGSGPAGYTAAIYAARA
PELHB02084      ------------------------------MIGTTTMHAKTIIIGSGPAGYTAAIYAARA
HYPDA03273      -------------------------------MTKKPHHSKVIVLGSGPAGYTAAIYAARA
HYPSM04642      -------------------------------MTKKPHHAKVIVLGSGPAGYTAAIYTARA
OLICO01107      CPG-----------------ARCPQGQVERLSMAGPISAKVVIIGSGPAGYTAAIYAARA
```

```
OLICM02790      --------------------------------MAGPISAKVVIIGSGPAGYTAAIYAARA
RHOPS01446      --------------------------------MPSPVHAKVVIIGSGPAGYTAAIYAARA
RHOPA04062      --------------------------------MPSPVQAKVVIIGSGPAGYTAAIYASRA
RHOPT04533      --------------------------------MPSPVQAKVVIIGSGPAGYTAAIYASRA
RHOPX04235      --------------------------------MSSPVQAKVVIIGSGPAGYTAAIYASRA
BRADU07321      --------------------------------MSAPVHAKVVIIGSGPAGYTAAIYAARA
BRASO01239      --------------------------------MQAPIHAKVVIIGSGPAGYTAAIYAARA
BRASB06352      --------------------------------MQAPIHAKVVIIGSGPAGYTAAIYAARA
RHOPB03773      --------------------------------MADPVHAKVVIIGSGPAGYTAAIYAARA
NITWN02324      --------------------------------MPAPIHAKVVIIGSGPAGYTAAIYAARA
NITHX02707      --------------------------------MPAPAHAKVVIIGSGPAGYTAAIYAARA
AZOC501143      ----------------------------------MASHSKVIIIGSGPAGYTAAIYAARA
XANP202679      ---------------------------------MTAHTVKVVIIGSGPAGYTAAIYAARA
CHLTF01983      ---------------------------------MTSQHRRVIIIGSGPAGFTAAIYAARA
IGNAJ02236      -------------------------------MSEQTNHFKVAIIGSGPAGLTAAIYTARA
MELRP00403      ---------------------------------MNENHHKVIVIGSGPAGLTAALYTGRA
ANADF00468      ----------------------------------MAQHERLVIIGSGPAGYTAALYAARA
CHLCH01125      ---------------------------------MERTVRDLIIMGTGPAGYTAAIYTGRA
PELPB01419      ---------------------------------MESNVRDIIIMGTGPAGYTSAIYMGRA
CHLL200781      ---------------------------------MEKEIRDVVIIGTGPAGYTAAIYTGRA
CHLTE00830      ---------------------------------MDKDIRDVVIIGTGPAGYTSAIYTGRA
CHLP800854      ---------------------------------MDKDIRDVVIIGTGPAGYTAAIYTGRA
CHLL701225      ---------------------------------MEGDVRDIVIIGTGPAGYTAAIYTGRA
CHLPM00993      ---------------------------------MEREVRDIVIIGTGPAGYTAAIYTGRA
WOLTR00562      -------------------------------MKNYRFGTKVLIIGSGAAGYAAAIYAARA
WOLPP00135      -------------------------------MQNYKLSTKVLIIGSGAAGYAAAIYAARA
WOLPM00650      -------------------------------MKNYKFSTKVLIIGSGAAGYAAAIYAARA
WOLWR00416      -------------------------------MKNYKFSTKVLIIGSGAAGYAAAIYAARA
EHRCR00696      --------------------------------MTQTYNTKVLIIGSGAAGCTAAIYAARA
ANAMM00345      ---------------------------MTEPQQQAKAPVKVLVMGSGAAGCTAAIYAGRA
ANAMF00341      ---------------------------MTEPQQQAKAPVKVLVMGSGAAGCTAAIYAGRA
ANAPZ00610      ------------------------------MSEVHKDAVKVLILGSGAAGCTAAIYAGRA
NEOSM00537      --------------------------------MCDALESDVIIIGSGPAGCTAGIYAARA
NEORI00513      --------------------------------MRDALESDVLIIGSGPAGCTAGIYAARA
PELUB00076      ------------------------------MSENPIKHTKVLIIGSGPAGYTAAVYAARA
PELSM00736      ------------------------------MSQNKESHSKVLIIGSGPAGYTAAIYAARA
PUNMI01409      ------------------------------MSASSSTHEHVVIIGAGAAGLSAGIYTARA
ASTEC00651      --------------------------------MTTPRTVRCAIIGSGPAGWTAAIYAARG
ZYMMT00154      -------------------------------MSADPLFTRIFILGSGPAGLTAAIYAARA
ZYMMO00984      -------------------------------MSADPISTRVFILGSGPAGLTAAIYAARA
ZYMMA00176      -------------------------------MSADPISTRVFILGSGPAGLTAAIYAARA
ZYMMN00183      -------------------------------MSADPISTRVFILGSGPAGLTAAIYAARA
SPHAL00143      --------------------------------MPSTHHTKMLILGSGPAGLSAAIYAARA
SPHWW03978      --------------------------------MTASHSTRMLILGSGPAGLSAAIYGARA
SPHJU02313      --------------------------------MTATHSTRMLILGSGPAGLSAAIYGARA
NOVAD02319      --------------------------------MATTHKTRMLIIGSGPAGLSAAIYGARA
ERYLH00934      ---------------------------------MTTHRTKMLIIGSGPAGYSAAIYGARA
GRABC00720      ----------------------------------MIHRSKVLIIGAGPAGYTAAIYAARA
GLUDA03075      ---------M-SSALDGH--IPCIVITSTPESMTQTHTTDLLVIGAGPAGYTAAIYAARA
KOMMN00774      --------------------------------MPQTCSTDLLVIGAGPAGYTAAIYAARA
HALVD01097      ---------------------------MTEDADSFVEHRKLIIAGSGISGLSSAIYAARS
HALHT01699      -------------------------------MTESVEHRRLIVAGTGAAGLTAAIYAARS
METI401038      -----------------------------------MSMEETIIIGSGCAGWTAAIYLARA
PLAL201612      ------------------------------------MTERVVIIGSGPAGWSAAIYAARA
GEMAT01983      --------------------------------MTDTRTENLVIIGSGPAAWTAAIYAARA
CYAAP02456      ------------------------------------MVENLVIIGSGPAGYTAAIYAGRA
CHLPN00303      -----------------------------------MIHSRLIIIGSGPSGYTAAIYASRA
CHLPP00439      -----------------------------------MIHSRLIIIGSGPSGYTAAIYASRA
CHLPE00727      -----------------------------------MSHSPVIIIGSGPAGYTAAVYAARA
CHLTR00101      -----------------------------------MTHAKLVIIGSGPAGYTAAIYASRA
CHLTA00102      FKA----------------------LLSKFILGVPMTHAKLVIIGSGPAGYTAAIYASRA
CHLTJ00101      FKA----------------------LLSKFILGVPMTHAKLVIIGSGPAGYTAAIYASRA
CHLTD00098      FKA----------------------LLSKFILGVPMTHAKLVIIGSGPAGYTAAIYASRA
CHLT700102      FKA----------------------LLSKFILGVPMTHAKLVIIGSGPAGYTAAIYASRA
CHLT000101      FKA----------------------LLSKFILGVPMTHAKLVIIGSGPAGYTAAIYASRA
```

```
CHLT500104      FKA----------------------LLSKFILGVPMTHAKLVIIGSGPAGYTAAIYASRA
CHLTL00098      FKA----------------------LLSKFILGVPMTHAKLVIIGSGPAGYTAAIYASRA
CHLTG00102      FKA----------------------LLSKFILGVPMTHAKLVIIGSGPAGYTAAIYASRA
CHLTS00101      FKA----------------------LLSKFILGVPMTHAKLVIIGSGPAGYTAAIYASRA
CHLT900102      FKA----------------------LLSKFILGVPMTHAKLVIIGSGPAGYTAAIYASRA
CHLTZ00101      FKA----------------------LLSKFILGVPMTHAKLVIIGSGPAGYTAAIYASRA
CHLT400432      FKA----------------------LLSKFILGVPMTHAKLVIIGSGPAGYTAAIYASRA
CHLT100103      FKA----------------------LLSKFILGVPMTHAKLVIIGSGPAGYTAAIYASRA
CHLT200350      FKA----------------------LLSKFILGVLMTHAKLVIIGSGPAGYTAAIYASRA
CHLTB00350      FKA----------------------LLSKFILGVLMTHAKLVIIGSGPAGYTAAIYASRA
CHLTC00378      -----------------------------------MTHAKLVIIGSGPAGYTAAIYASRA
SIMNZ00831      -----------------------------------MEKRKVVIIGSGPAGYTAAIYASRA
PARUW01706      -----------------------------------MEKAKLIIIGSGPAGYTAAIYAARA
WADCW00943      -------------------------------MEIKMKKTKVAIIGSGPAGYTAAIYASRA
SINAD04980      -----------------------------------MSHSRVIVIGSGSAGLTSALYTARA
BIFLB01558      -------------------------------MNSTATVHNVIIVGSGPAGYTAAIYLARA
BIFAB00498      ----------------------------MGVMNSTATVHNVIIVGSGPAGYTAAIYLARA
BIFAV01561      -------------------------------MNSTATVHNVIIVGSGPAGYTAAIYLARA
BIFAS01557      -------------------------------MNSTATVHNVIIVGSGPAGYTAAIYLARA
BIFA001518      -------------------------------MNSTATVHNVIIVGSGPAGYTAAIYLARA
BIFBA01814      ------------------------------------MEHNVIIIGSGPAGYTAAIYLGRA
BIFAA01619      ----------------------------------MSDMRDVVIIGSGPAGYTAAIYLGRA
BIFDB02112      GMA----------------------RRKCSYGGTMSDLRDVVIIGSGPAGYTAAIYLGRA
TERSS03091      -------------------------------MATETMTRDTVILGSGCSGLTAAIYTGRA
GRATM03160      -------------------------MTPESSIPAAIETRDTVILGSGCSGLTAAIYAARS
GRAMM03836      -------------------------------MSDQIEVRDTVILGSGCSGLTAAIYAARS
AKKM801247      ------------------------------------MSEQVIIIGAGCAGYTAAIYTARA
OPITP04130      ---------------------------------MSSSVENVVIVGTGCAGLTAAIYTARA
CORAD01794      -------------------------------------MEDVIILGTGCAGLTAAIYTGRA
BUCCC00180      -----------------------------MIKKTKKKIVQLIIIGSGPAGYTAAIYAARS
CENSY00347      -MAAEPEGG-----------VL--VEKGPPKEPRKKTKYDVIIIGAGPAGYTAGIYCSRA
NITMS00668      MMAADAGST-----------VLESKDDGPKMPDKKKTKFDVVIIGAGPSGYTAGIYCSRA
MEIRD02823      ----------------------------------MSQLYDVVIIGGGPAGLTAGIYTGRA
HERA203780      -----------------------------------MEKQKVTVIGSGPAGLTAALYAARA
CHLAA02018      ------------------------------------MHHKVVIIGSGPAGLTAALYAARA
CHLSY02175      ------------------------------------MHHKVVIIGSGPAGLTAALYAARA
CHLAD02599      ------------------------------------MHHKVVIIGSGPAGLTAALYAARA
CALAS01745      ---------------------M----TTVNGHGNTPQVENIIIVGSGPAGFTAGLYAARA
ANATU01696      -MD--FNLM-----------NL----TSHAEDEKEHHPYKVVILGSGPAGLAAALYAARA
SULMS00235      ----------------------------------MKKIFDCIIIGSGPAGYTAAIYIARA
BLASB00041      ---------------------------------MLKKIQDCVIIGSGPAGYSAAIYASRA
BLASP00551      -------------------------------MLKKNKIMDCVILGSGPAGYTAAIYAARA
AZOPC00437      ---------------MKH-------NTNNKSSLQDEEQVKCLIIGSGPAGYSSAIYTARA
LEPBD01878      -------------------------------------MYDSVIIGSGPAGLTAAIYLSRA
SALRD02577      LMHENGALAN-GNQTNGH--QHPDLAAFEDVDFSEAEMRDVVVIGTGPAGWTAALYTARA
SALRM02866      LMHENGALAN-GNQTNGH--QHPDLAAFEDVDFSEAEMRDVVVIGTGPAGWTAALYTARA
RIEPU00166      ----------------------------MKRTNDNFLHRKVIILGSGPAGCTSAIYTSRA
ORITB00015      ----------------------------------MSRKVNVLIMGSGVAGSTAALYLARS
ORITI01234      --------------------------------MIISRKVNVLIMGSGVAGSTAALYLARS
PREMB01541      -----------------------------------MEKVKTLIIGSGPAGYTAAIYAGRA
PREDF01155      -----------------------------------MEKTKTLIIGSGPAGYTAAIYAGRA
PREI702011      -----------------------------------MEKVKTLIIGSGPAGYTAAIYASRS
ALIFI01035      ----------------------------------MEETIKCLIIGSGPAGYTAAIYTSRA
ODOSD00362      -----------------------------MAEENAIERIRCVIIGSGPAGYTAAIYAARA
PRER201250      -----------------------------------MEHTKCLIIGSGPAGYTAAIYASRA
PALPW00206      ----------------------------------MNEKVRCLIIGSGPAGYTAAIYASRA
PORGI00953      ----------------------------------MSEHARCLIIGSGPAGYTAAIYASRA
PORG301168      ----------------------------------MSEHARCLIIGSGPAGYTAAIYASRA
BACV803426      ---------------------------------MSTEKVKCLIIGSGPAGYTAAIYTGRA
BACT601486      ---------------------------------MEIEKVKCLIIGSGPAGYTAAIYAGRA
BACTN04290      --------------------------------MAEIEKVKCLIIGSGPAGYTAAIYAGRA
BACFR01032      ---------------------------------METERIKCLIIGSGPAGYTAAIYAGRA
BACFN00913      ---------------------------------METERIKCLIIGSGPAGYTAAIYAGRA
BACF600963      ---------------------------------METERIKCLIIGSGPAGYTAAIYAGRA
OWEHD03413      -----------------------------------MEKHKCVIIGTGPAGYTAAIYAARA
```

```
PSYTT00390      -------------------------------MSDTIERIKCLIVGSGPAGYTAAIYAARA
NONDD00011      -------------------------------MSENIERIKCLIIGSGPAGYTAAIYAARA
ROBBH02711      -------------------------------MSESIERIKTLILGSGPAGYTAAIYAARA
CELAD02592      -------------------------------MSDKIERIKTLIIGSGPAGYTAAIYAARA
CELLC02474      -------------------------------MSDTIERIKCLIIGSGPAGYTAAIYASRA
MARSH03072      -------------------------------MSEKVERVKTLIIGSGPAGYTAAIYASRA
MURRD00159      -------------------------------MSEQVERIKTLIIGSGPAGYTAAIYAARA
ZOBGA00152      -------------------------------MSEKIERLKTLIIGSGPAGYTAAIYAARA
GRAFK00949      -------------------------------MSDTIERVKCLIIGSGPAGYTAAIYAARA
ZUNPS02344      -------------------------------MSETIEKIKCLIIGSGPAGYTAAIYAARA
AEQSU02079      -------------------------------MSDTIERIKCLIIGSGPAGYTAAIYAARA
HALH105745      -------------------------------MATTAEHLHCVIIGSGPAGYTAAVYAARA
SAPGL02893      ----------------------------------MPKTVKCLIIGSGPAGYTAAIYASRA
AMOA500014      -----------------------------MDNASTSSPVSVAIIGSGPAGYTAAIYAARA
FLAIG01802      -------------------------------MSDTIEKVKCLIIGSGPAGYTAAIYAARA
FLACA02326      -------------------------------MSDTIERVKCLIIGSGPAGYTAAIYAARA
FLAJ100198      -------------------------------MSDTIEKIKCLIIGSGPAGYTAAIYAARA
FLABF01528      --------------------------------MATIEKVKCLIIGSGPAGYTAAIYASRA
CAPOD01333      --------------------------------MANIEKIKCLIIGSGPAGYTAAIYASRA
CAPCC00414      --------------------------------MAEIEKIKCLIIGSGPAGYTAAIYASRA
FLELS01898      --------------------------------MSEIHKTKCLIIGSGPAGYTAAIYAARA
SOLCM01166      -------------------------------MSNEVEHIHTLIIGSGPAGYTAAIYAARA
PEDHD01598      -----------------------------MSQEQEIEHVQCLIIGSGPAGYTAAIYAARA
SPHS203207      -------------------------------MTEEIEHVKCLIIGSGPAGYTAAIYAARA
LEAB401188      -----------------------------------MEKVDVLILGSGPAGYTAAIYAARA
EMTOG00092      ---------------------------------MTQEKVDVLILGSGPAGYTAAIYAARA
SPILD01737      ---------------------------------MTSEHVKCLIIGSGPAGYTAAIYAARA
DYAFD00084      ---------------------------------MSSEKVSCLIIGSGPAGYTAAIYASRA
CYCMS03063      -------------------------------MDKEIEKIKVLIIGSGPAGYTAAIYASRA
ECHVK03326      -------------------------------MNKEAEKIKVLIVGSGPAGYTAAIYASRA
BELBD02542      -------------------------------MNTDIEKVKVLIIGSGPAGYTAAIYAARA
NITGG02112      FMQ--FEAIP-NGLLRGHG-IVTLLSENESGSGGNRKHCDVLIIGSGPAGYTAGVYTSRA
THEM700111      -----------------------------------MSSQRVVIVGSGPAGLTAAIYAARA
CREAS01350      -----------------------------------MVTRKIIIIGSGPAGYTAAIYASRA
TURPD02772      ---------------------------------MSANHYDTIIIGSGPAGYTAAIYAGRA
SORC507603      --------------------------------MSESNVRNLVIIGSGPAGLTAAIYAARA
BDEBA00337      -------------------------------MTQDQKVENVIIIGSGPAGLTSAIYSSRA
STIAD02752      --------------------------------MADEKINKVTIIGSGPAGYTAAVYAARA
MYXXD01876      --------------------------------MAEEKINKVTIIGSGPAGYTAAIYAARA
MYXFH03454      --------------------------------MAEEKTNKVTIIGSGPAGYTAAIYAARA
CORCM01960      --------------------------------MSQDKIQKVTIIGSGPAGYTAAIYAARA
MYXSD02209      --------------------------------MAEEKINKVTIIGSGPAGYTAAIYAARA
LEPFC02126      -------------------------------------MEDVVILGSGPAGLTAALYTARA
LEPFM02294      -------------------------------------MEKVVILGSGPAGLTAALYTARA
SOLUE00582      -------------------------------------MRNVVIIGSGCAGNTAAIYTARA
KORVE01333      ---------------------------------MTDNVRNTVILGSGCSGLTAAIYAARA
ACIC502553      ---------------------------------MTEAIFDTVILGSGCAGLTAAIYTARA
ACIFD00030      ----------------------------------MPEHVKVVIIGSGPAGLTAAIYAARA
HALMS01402      -----------------------------------METRKVVIVGSGPAGYTAALYASRA
CHLPD01133      ---------------------------------MERDIRDIVIMGTGPAGYTAAIYSGRA
CHLPB01137      ---------------------------------MDNGIQDIVIIGTGPAGLTAAIYTGRA
PROA200897      ---------------------------------MANQVRDVVIMGTGPAGLTAAIYTGRT
WIGBR00492      ----------------------------------MKIKSKLLILGSGPAGFTSAIYSSRA
BUCA500289      --------------------------------MDKVKHSKIIILGSGPAGYTAAIYAARA
BUCAI00292      --------------------------------MDKVKHSKIIILGSGPAGYTAAIYAARA
BUCAF00306      --------------------------------MDKVKHSKIIILGSGPAGYTAAIYAARA
BUCAT00287      --------------------------------MDKVKHSKIIILGSGPAGYTAAIYAARA
BUCA000302      --------------------------------MDKVKHSKIIILGSGPAGYTAAIYAARA
BAUCH00290      --------------------------------MHNIRHNNLIILGSGPAGYTAAIYAARA
BLOVB00369      -------------------------------MKNKNKHCKLLILGSGPAGYTAAIYSARA
BLOFL00367      -----------------------------MPITTLKQHCKLLILGSGPAGYTAAIYASRA
BLOPB00375      --------------------------------MMIKKYCKLLILGAGPAGYTAAIYAARA
BUCAP00289      --------------------------------MELKNHKKIIILGSGPAGYTAAIYSSRA
COXBU01001      --------------------------------MNKPQHHSLIILGSGPAGYTAAIYAARA
COXBN01188      --------------------------------MNKPQHHSLIILGSGPAGYTAAIYAARA
```

```
COXBR01117      --------------------------------MNKPQHHSLIILGSGPAGYTAAIYAARA
COXB200717      --------------------------------MNKPQHHSLIILGSGPAGYTAAIYAARA
COXB100914      --------------------------------MNKPQHHSLIILGSGPAGYTAAIYAARA
FRAP200241      ----------------------------------MANHHKLIILGSGPAGYTAAIYAARA
FRANT01012      ----------------------------------MATHHKLIILGSGPAGYTAAIYAARA
FRACN00522      ----------------------------------MANHHKLIILGSGPAGYTAAIYAARA
FRATT00465      ----------------------------------MANHHKLIILGSGPAGYTAAIYAARA
FRAT100465      ----------------------------------MANHHKLIILGSGPAGYTAAIYAARA
FRATE00462      ----------------------------------MANHHKLIILGSGPAGYTAAIYAARA
FRATW01216      ----------------------------------MANHHKLIILGSGPAGYTAAIYAARA
FRATM01110      ----------------------------------MANHHKLIILGSGPAGYTAAIYAARA
FRACF00566      ----------------------------------MANHHKLIILGSGPAGYTAAIYAARA
FRATO01212      ----------------------------------MANHHKLIILGSGPAGYTAAIYAARA
FRATH01486      ----------------------------------MANHHKLIILGSGPAGYTAAIYAARA
FRATF01216      ----------------------------------MANHHKLIILGSGPAGYTAAIYAARA
FRATN00564      ----------------------------------MANHHKLIILGSGPAGYTAAIYAARA
ACIF500512      --------------------------MSDPKSVIDEKHCRLLILGSGPGGYTAAIYAARA
ACIF200362      --------------------------MSDPKSVIDEKHCRLLILGSGPGGYTAAIYAARA
DECAR01284      -------------------------------MPQNVRHNPLIILGSGPAGYTAAVYAARA
NEIG100525      ----------------------------------MSQHRKLIILGSGPAGYTAAVYAARA
NEIG201314      ----------------------------------MSQHRKLIILGSGPAGYTAAVYAARA
NEIM800926      ----------------------------------MSQHRKLIILGSGPAGYTAAVYAARA
NEIMP01199      ----------------------------------MSQHRKLIILGSGPAGYTAAVYAARA
NEIMB01212      ----------------------------------MSQHRKLIILGSGPAGYTAAVYAARA
NEIMF01143      ----------------------------------MSQHRKLIILGSGPAGYTAAVYAARA
NEIML01119      ----------------------------------MSQHRKLIILGSGPAGYTAAVYAARA
NEIMM00780      ----------------------------------MSQHRKLIILGSGPAGYTAAVYAARA
NEIMH00827      ----------------------------------MSQHRKLIILGSGPAGYTAAVYAARA
NEIMG01168      ----------------------------------MSQHRKLIILGSGPAGYTAAVYAARA
NEIMN01254      ----------------------------------MSQHRKLIILGSGPAGYTAAVYAARA
NEIMO00810      ----------------------------------MSQHRKLIILGSGPAGYTAAVYAARA
NEIM701229      ----------------------------------MSQHRKLIILGSGPAGYTAAVYAARA
NEIMA01334      ----------------------------------MSQHRKLIILGSGPAGYTAAVYAARA
NEIMW01125      ----------------------------------MSQHRKLIILGSGPAGYTAAVYAARA
DICNV01037      -----------------------------------MIHHRLIILGAGPAGYTAAIYAARA
VESOH00047      --------------------------------MSENKHCKVLIIGSGPAGYSAAVYAARA
RUTMC00043      --------------------------------MSENKHCKVLIIGSGPAGYSAAVYAARA
HALHL02255      -------------------------------MAQQSKHCNVLILGSGPAGYTAAIYAARA
PELPD03177      ---------------------------------MDSTHHPLLILGAGPAGYTAAIYAARA
GEOS804030      ---------------------------------MEPIHHRLIILGSGPAGFTAAIYAARA
GEOBB03636      ---------------------------------MEVTHHRLIILGSGPAGYTAAVYAARA
GEOSM03700      ---------------------------------MEVTHHRLIILGSGPAGYTAAVYAARA
HALNC00046      ------------------------------MSETNTRHARLLILGSGPAGYAAAVYAARA
HAEPS00887      ---------------------------------MATKHAKLLILGSGPAGYTAAVYAARA
GALAU02285      ---------------------------------METKHSKLLILGSGPAGYTAAIYAARA
HISS201000      --------------------------------MSDFKHSQLLILGSGPAGYTAAIYAARA
HAES101151      --------------------------------MSDFKHSQLLILGSGPAGYTAAIYAARA
PASMU00573      --------------------------------MNAMKHSQLLILGSGPAGYTAAIYAARA
PASMH00579      --------------------------------MNAMKHSQLLILGSGPAGYTAAIYAARA
ACTSZ01560      --------------------------------MSDVKHSKLLILGSGPAGYTAAIYAARA
AGGAN00985      --------------------------------MSNIKHAKLLILGSGPAGYTAAIYAARA
NITHN03181      ------------------------------MSETKHKHCRLLILGSGPAGYTAAVYAARA
NITOC00311      --------------------------------MSKIKHCRLLILGSGPAGYTAAVYAARA
NITWC02498      --------------------------------MSKIKHCRLLILGSGPAGYTAAVYAARA
METNJ00648      --------------------------------MADSKHCRLLILGSGPAGYTAAVYAARA
METFJ01616      --------------------------------MADSKHCRLLILGSGPAGYTAAVYAARA
ALKEH00244      --------------------------------MSETKHCKLLILGSGPAGYTAAIYAARA
MARMS03166      --------------------------------MSDVKHAKLMILGSGPAGYTAAVYAARA
MARM102688      --------------------------------MSEVKHAKLLILGSGPAGYTAAVYAARA
THICR00763      ---------------------------------MSTKHCKLLILGSGPAGYTAAVYAARA
THICA00974      ---------------------------------MTTKHCKLLILGSGPAGYSAAVYAARA
THIV600851      --------------------------------MSETKHCRLLILGSGPAGYTAAVYAARA
THISH01973      --------------------------------MSDPKHSRLLILGSGPAGYTAAVYAARA
METAA00813      --------------------------------MTDTKHCRLLILGSGPAGYTAAVYGARA
META200813      --------------------------------MTDTKHCRLLILGSGPAGYTAAVYGARA
```

```
METMM02895     --------------------------------MADAKHCKLLILGSGPAGYTAAVYAARA
FRAAD00813     --------------------------------MSSPKHSRLLILGSGPAGYTAAVYAARA
XYLFA01416     ----------------------------MSDYPASAKHSRLLILGSGPAGWTAAVYAARA
XYLFT00623     ----------------------------MSDYPASAKHSRLLILGSGPAGWTAAVYAARA
XYLF200667     ----------------------------MSDYPASAKHSRLLILGSGPAGWTAAVYAARA
XYLFG01622     ----------------------------MSDYPASAKHSRLLILGSGPAGWTAAVYAARA
XYLFM00690     ----------------------------MSDYPASAKHSRLLILGSGPAGWTAAVYAARA
PSEUP01680     --------------------------------MSTSKHSRLLILGSGPAGWTAAVYAARA
STRM501972     --------------------------------MSTTRHIPLLILGSGPAGWTAAVYAARA
PSEUU01524     --------------------------------MTASKHSRLLILGSGPAGWTAAVYAARA
XANAP01407     ----------------------CPRVYMSPSAAHSAKHSRLLILGSGPAGWTAAVYAARA
XANCP01918     ---------------------------MSASSASPAKHSRLLILGSGPAGWTAAVYAARA
XANC802154     ---------------------------MSASSASPAKHSRLLILGSGPAGWTAAVYAARA
XANCB02263     ---------------------------MSASSASPAKHSRLLILGSGPAGWTAAVYAARA
XANOR02379     ---------------------------MSASSASPAKHSRLLILGSGPAGWTAAVYAARA
XANOM02295     ---------------------------MSASSASPAKHSRLLILGSGPAGWTAAVYAARA
XANOP02318     ---------------------------MSASSASPAKHSRLLILGSGPAGWTAAVYAARA
XANAC01951     ---------------------------MSASSASPAKHSRLLILGSGPAGWTAAVYAARA
CYCSP01155     --------------------------------MSDNSHHRLIILGSGPAGYTAAVYAARA
GEOLS03275     --------------------------------MAEMQHHRLIILGSGPAGYTAAVYAARA
GEOUR03858     ---------------------------------MSIIHHRLIILGSGPAGYTAAIYAARG
GEODF00692     ---------------------------------MAIVHHRLIILGSGPAGYTAAIYAARA
GEOSL00482     ---------------------------------MQATHHRLLILGSGPAGYTAAVYAARA
GEOSK00469     ---------------------------------MQATHHRLLILGSGPAGYTAAVYAARA
GEOMG02998     ---------------------------------METTHHRLIILGSGPAGYTAAVYAARA
MORCR00228     ----------------------------------MSQHHRLIILGSGPAGYSAAVYAARA
ACIAD00798     ---------------------------------MSARHSRLIILGSGPAGYSAAVYAARA
ACIBC00821     ---------------------------------MSARHSRLIILGSGPAGYSAAVYAARA
ACIBY02725     ---------------------------------MSARHSRLIILGSGPAGYSAAVYAARA
ACIB302691     ---------------------------------MSARHSRLIILGSGPAGYSAAVYAARA
ACIB500882     ---------------------------------MSARHSRLIILGSGPAGYSAAVYAARA
ACIB100850     ---------------------------------MSARHSRLIILGSGPAGYSAAVYAARA
ACIBD00843     ---------------------------------MSARHSRLIILGSGPAGYSAAVYAARA
ACIBS02229     ---------------------------------MSARHSRLIILGSGPAGYSAAVYAARA
ACICP00137     ---------------------------------MSARHSRLIILGSGPAGYSAAVYAARA
ACISD03052     ---------------------------------MSARHSRLIILGSGPAGYSAAVYAARA
LEGLN02402     --------------------------------MSASNHHRLIILGSGPAGYTAAVYAARA
LEGPA01726     --------------------------------MSISNHHRLIILGSGPAGYTAAVYAARA
LEGPH00846     ----------------IFAI--LTQKIRKAETMSISNHHRLIILGSGPAGYTAAVYAARA
LEGPC01171     --------------------------------MSISNHHRLIILGSGPAGYTAAVYAARA
LEGP201857     --------------------------------MSISNHHRLIILGSGPAGYTAAVYAARA
LEGPL01727     --------------------------------MSISNHHRLIILGSGPAGYTAAVYAARA
COLP302668     --------------------------------MSDVRHCPLLILGSGPAGYTAAVYAARA
KANKD01106     --------------------------------MSETRHFKCLILGSGPAGYSAAVYAARA
IDILO00659     --------------------------------MAEAKHCKLLILGSGPAGYTAAVYAARA
PSEU901284     --------------------------------MTEAKHCKLLILGSGPAGYTAAVYAARA
PSEA602346     --------------------------------MSESRHVKLLILGSGPAGYSAAVYAARA
ALTSS01871     --------------------------------MADNRHVRLLILGSGPAGYSAAVYAARA
ALTMD01611     -------------------------------------------MGSGPAGYSAAVYAARA
ALTME01705     --------------------------------MAESRHVRLLILGSGPAGYSAAVYAARA
ALTMB01797     --------------------------------MAESRHVRLLILGSGPAGYSAAVYAARA
ALTMS01652     --------------------------------MAESRHVRLLILGSGPAGYSAAVYAARA
SACD201683     --------------------------------MTDVQHHQVIVLGSGPAGYTAAIYAARA
TERTT01748     --------------------------------MSDAKHFPLIILGSGPAGYTAAIYAARA
SIMAS00609     --------------------------------MSDIKHHRLIILGSGPAGYTAAIYAARA
ALCDB01962     --------------------------------MSDVQHHRLIILGSGPAGYTAAVYAARA
CHRSD02939     --------------------------------MSDVRHERLIILGSGPAGYTAAVYAARA
HALED02811     --------------------------------MSEARHERLIILGSGPAGYTAAVYAARA
SIDLE02655     -------------------------------MEKPAQHHRLIILGSGPAGYTAAIYAARA
GALCS01621     ---------------------------------MTTQHHRLIILGSGPAGYTAAVYAARA
LARHH01446     --------------------------------MSQTRHSSLLILGSGPAGYTAAVYSARA
CHRVO02807     --------------------MNSHLFDDRKTHMSAPRHSPLLILGSGPAGYTAAVYAARA
PSEUL01648     --------------------------------MSTTRHSPLLILGSGPAGYTAAVYAARA
NITEU01859     --------------------------------MTTTRHCKLLILGSGPAGYTAAIYAARA
NITEC00745     --------------------------------MTTTRHCKLLILGSGPAGYTAAIYAARA
```

```
NITMU00023      ---------------------------------MTSKHARLLILGSGPAGYTAAVYAARA
NITSI03064      ---------------------------------MTTKHSALLILGSGPAGYTAAVYAARA
ACCPU02128      -------------------------------MTPATRHCRLLILGSGPAGYTAAVYAARA
THIDA01000      V-----------R---IS-HCPIPPLGSQRTRAMSTQHHKLVILGSGPAGYSAAVYAARA
METS601221      ---------------------------------MATKHAHLLILGSGPAGYSAAVYAARA
METGS01186      ---------------------------------MATKHAHLLILGSGPAGYSAAVYAARA
METFK00972      ---------------------------------MATKHARLLILGSGPAGYTAAVYAARA
METML01428      ---------------------------------MATRHAKLLILGSGPAGYSAAVYAARA
AROAE03899      ---------------------------------MTTKHARLLILGSGPAGYTAAVYAARA
THASP01686      ---------------------------------MTTKHARLLILGSGPAGYTAAVYAARA
AZOSB01359      ---------------------------------MTTKHARLLILGSGPAGYTAAVYAARA
BORA102682      ----------------------------------MSTHAKVLILGSGPAGYTAAVYAARA
BORPA03415      -----------------------MGVILKIPAMSTPKHAKVLILGSGPAGYTAAVYAARA
BORBM03577      --------------------------------MSTPKHAKVLILGSGPAGYTAAVYAARA
BORPE02280      --------------------------------MSTPKHAKVLILGSGPAGYTAAVYAARA
BORPC02060      --------------------------------MSTPKHAKVLILGSGPAGYTAAVYAARA
BORP102392      --------------------------------MSTPKHAKVLILGSGPAGYTAAVYAARA
BORBR03870      --------------------------------MSTPKHAKVLILGSGPAGYTAAVYAARA
BORPD01524      --------------------------------MPTPKHAKVLILGSGPAGYTAAVYAARA
ACHXA01179      --------------------------------MSTPTHAKVLILGSGPAGYTAAVYAARA
RHOFT03114      --------------------------------MSTTTHAKVLILGSGPAGYTAAIYAARA
VEREI01701      ------------------------MSTPTATAAATTRHAQVLILGSGPAGYTAAVYAARA
VARPE01375      --------------------------------MSAPQHAKVLILGSGPAGYTAAVYAARA
VARPS01266      --------------------------------MSAPQHAKVLILGSGPAGYTAAVYAARA
DELAS05272      H-----------A---GC----RRAPFSRTASMSSTQHAKVLILGSGPAGYTAAIYAARA
DELSC01235      --------------------------------MSSTQHAKVLILGSGPAGYTAAIYAARA
COMT200883      --------------------------------MSSTKHAQVLILGSGPAGYTAAVYAARA
ACIAC03267      --------------------------------MSTTQHAKVMILGSGPAGYTAAVYAARA
ACIAP03249      --------------------------------MSTTQHAKVMILGSGPAGYTAAVYAARA
ACIET02648      --------------------------------MSTTQHAKVMILGSGPAGYTAAVYAARA
ALIDK03528      --------------------------------MSTTQHAKVMILGSGPAGYTAAVYAARA
RUBGI03408      -------------------------------MTQNTTHARVLILGSGPAGYTAAIYAARA
LEPCP00749      -------------------------------MSSTHQHAQVLILGSGPAGYTAAIYAARA
POLSJ03719      -----------------L----P-DFQHFPNQFFTMKHSKVLILGSGPAGYTAAVYAARA
POLNA03144      -----------------------------------MKHSRVLILGSGPAGYTAAVYAARA
METPP01110      --------------------------------MTTPLHARVLILGSGPAGYTAAVYAARA
RAMTT03218      -----------------R----Q-RARTAFTDTMTTRHAKVLILGSGPAGYTAAVYAARA
RALPJ02457      ----------------------------------MAKHAKVLILGSGPAGYTAAIYAARA
RALP102102      ----------------------------------MAKHAKVLILGSGPAGYTAAIYAARA
RALSO02303      ----------------------------------MAKHAKVLILGSGPAGYTAAIYAARA
RALS801097      ----------------------------------MAKHAKVLILGSGPAGYTAAIYAARA
HERSS01878      -------------------------------MSTTPKHAKVLILGSGPAGYSAAVYAARA
HERAR00955      --------------------------------MPSPKHAHVLILGSGPAGYSAAVYAARA
JANMA01152      --------------------------------MSSAKHAHVLILGSGPAGYSAAVYAARA
THIK102324      ---------------------------------MTSKHAKVLILGSGPAGYSAAVYAARA
POLSQ00682      ------------------------------MTTNTPKHSKVLILGSGPAGYTAAVYAARA
POLNS00989      ------------------------------MTTNTPKHSKVLILGSGPAGYTAVVYAARA
BURP800627      ------------------------------MPASSTKHAKVLILGSGPAGYTAAVYAARA
BURPP00988      ------------------------------MPATSTKHAKVLILGSGPAGYTAAVYAARA
BURSC00728      T-----------H---GYAMASRAPFKDSIMPATATKHAKVLILGSGPAGYTAAVYAARA
BURXL00982      ------------------------------MPATSTKHAKVLILGSGPAGYTAAVYAARA
BURSG00869      ------------------------------MPATSTKHAKVLILGSGPAGYTAAVYAARA
BURRH00629      P-----------R---GAALRTAVLQGSTAM--SERKHAKVLILGSGPAGYSAAVYAARA
BURGB00781      ---------------------------------MSTKHAKVLILGSGPAGYTAAVYAARA
BURGS00853      ------------------------------M--STPKHAKVLILGSGPAGYTAAVYAARA
BURPS02618      ------------------------------M--STPKHAKVLILGSGPAGYTAAVYAARA
BURMA01741      ------------------------------M--STPKHAKVLILGSGPAGYTAAVYAARA
BURP103016      ------------------------------M--STPKHAKVLILGSGPAGYTAAVYAARA
BURP002984      ------------------------------M--STPKHAKVLILGSGPAGYTAAVYAARA
BURM701866      ------------------------------M--STPKHAKVLILGSGPAGYTAAVYAARA
BURP602945      ------------------------------M--STPKHAKVLILGSGPAGYTAAVYAARA
BURM902465      ------------------------------M--STPKHAKVLILGSGPAGYTAAVYAARA
BURMS00740      ------------------------------M--STPKHAKVLILGSGPAGYTAAVYAARA
BURTA01515      ------------------------------M--STPKHAKVLILGSGPAGYTAAVYAARA
BURM102386      ------------------------------M--STPKHAKVLILGSGPAGYTAAVYAARA
```

```
BURL300890      P----------R---PP----RPPFTDSHM--STPKHAKVLIILGSGPAGYTAAVYAARA
BURVG00860      S----------R---TP----RPPFTDSHM--STPKHAKVLIILGSGPAGYTAAVYAARA
BURCM00829      S----------R---PP----RPPFTDSHM--STPKHAKVLIILGSGPAGYTAAVYAARA
BURA400832      -----------------------------M--STPKHAKVLIILGSGPAGYTAAVYAARA
BURCA00489      -----------------------------M--STPKHAKVLIILGSGPAGYTAAVYAARA
BURCH00966      -----------------------------M--STPKHAKVLIILGSGPAGYTAAVYAARA
BURCC00927      -----------------------------M--STPKHAKVLIILGSGPAGYTAAVYAARA
BURCJ02922      -----------------------------M--STPKHAKVLIILGSGPAGYTAAVYAARA
EDWI902348      --------------------------------MSSVKHCKLLILGSGPAGYTAAVYAARA
EDWTF01971      --------------------------------MSSVKHCKLLILGSGPAGYTAAVYAARA
EDWTE02176      --------------------------------MSSVKHCKLLILGSGPAGYTAAVYAARA
SODGM01094      --------------------------------MSTVKHSKLLILGSGPAGYTAAVYAARA
MOREP00394      --------------------------------MSTVKYSKLIILGSGPAGYTAALYAARA
RAHSY01432      --------------------------------MSTVKHSKLLILGSGPAGYTAAVYAARA
RAHAC01419      --------------------------------MSTVKHSKLLILGSGPAGYTAAVYAARA
ERWBE01489      --------------------------------MSTAKHSKLLILGSGPAGYTAAVYAARA
PANAM01339      VNKRAQVLSM-TRLI-PYNR-RLCQANETEERMSTAKHSKLLILGSGPAGYTAAVYAARA
PANAA00662      ---------M-TRLI-PYNR-RLCQANETEERMSTAKHSKLLILGSGPAGYTAAVYAARA
PANSA01303      --------------------------------MSTAKHSKLLILGSGPAGYTAAVYAARA
ERWT902154      VNKIAECFFCLVKFP-TILI-SVCQLN-NEGLMSTAKHSKLLILGSGPAGYTAAVYAARA
ERWAC01330      --------------------------------MSTAKHSKLLILGSGPAGYTAAVYAARA
ERWAE01326      --------------------------------MSTAKHSKLLILGSGPAGYTAAVYAARA
ERWPE02241      VNKIAECFFCLVKFP-TILS-SVCQLN-NEGLMSTAKHSKLLILGSGPAGYTAAVYAARA
ERWP602414      --------------------------------MSTAKHSKLLILGSGPAGYTAAVYAARA
ERWSE02396      --------------------------------MSTAKHSKLLILGSGPAGYTAAVYAARA
PECCP01694      --------------------------------MGTVKHHKLLILGSGPAGYTAAVYAARA
PECWW01889      --------------------------------MGTVKHHKLLILGSGPAGYTAAVYAARA
PECSS01870      --------------------------------MGTVKHHKLLILGSGPAGYTAAVYAARA
PECAS02624      --------------------------------MGTVKHHKLLILGSGPAGYTAAVYAARA
DICDC02220      --------------------------------MSTAKHRKLLILGSGPAGYTAAVYAARA
DICZE02284      --------------------------------MSTAKHRKLLILGSGPAGYTAAVYAARA
DICD302017      --------------------------------MSTAKHRKLLILGSGPAGYTAAVYAARA
DICD502250      --------------------------------MSTAKHRKLLILGSGPAGYTAAVYAARA
XENBS00829      --------------------------------MSTAKHSKLIILGSGPAGYTAAVYAARA
XENNA01480      --------------------------------MSTAKHSKLIILGSGPAGYTAAVYAARA
PHOLL01537      --------------------------------MSAAKHHKLIILGSGPAGYTAAVYAARA
PHOAA02804      ---------------------------------MSTAHHKLIILGSGPAGYTAAVYAARA
SERP501669      --------------------------------MGTAKHSKLLILGSGPAGYTAAVYAARA
SERSA01625      --------------------------------MGTAKHSKLLILGSGPAGYTAAVYAARA
YERPE01271      --------------------------------MSTAKHSKLIILGSGPAGYTAAVYAARA
YERPS01390      --------------------------------MSTAKHSKLIILGSGPAGYTAAVYAARA
YERPA00627      --------------------------------MSTAKHSKLIILGSGPAGYTAAVYAARA
YERPN02489      --------------------------------MSTAKHSKLIILGSGPAGYTAAVYAARA
YERPP02190      --------------------------------MSTAKHSKLIILGSGPAGYTAAVYAARA
YERP302544      --------------------------------MSTAKHSKLIILGSGPAGYTAAVYAARA
YERPB01460      --------------------------------MSTAKHSKLIILGSGPAGYTAAVYAARA
YERPY02633      --------------------------------MSTAKHSKLIILGSGPAGYTAAVYAARA
YERPG01392      --------------------------------MSTAKHSKLIILGSGPAGYTAAVYAARA
YERPD01171      --------------------------------MSTAKHSKLIILGSGPAGYTAAVYAARA
YERP100890      --------------------------------MSTAKHSKLIILGSGPAGYTAAVYAARA
YERPZ01206      --------------------------------MSTAKHSKLIILGSGPAGYTAAVYAARA
YERPH02469      --------------------------------MSTAKHSKLIILGSGPAGYTAAVYAARA
YERE801438      --------------------------------MSTAKHSKLIILGSGPAGYTAAVYAARA
YERE302565      --------------------------------MSTAKHSKLIILGSGPAGYTAAVYAARA
YERE100392      --------------------------------MSTAKHSKLIILGSGPAGYTAAVYAARA
PROMH00687      --------------------------------MSTIKHSKLIILGSGPAGYTAAVYAARA
PROSM03196      --------------------------------MSTAIHRKLIILGSGPAGYTAAVYAARA
TOLAT02279      --------------------------------MTQVKHSKLLILGSGPAGYTAAVYAARA
AERVB02262      --------------------------------MSQVTHSKLLILGSGPAGYTAAVYAARA
AERHH01811      IDAIDSLAFDPLSRI-TYNRANSSR--LGNHIMSQVTHSKLLILGSGPAGYTAAVYAARA
AERS402185      --------------------------------MSQVTHSKLLILGSGPAGYTAAVYAARA
PSYIN02070      --------------------------------MSETKHQKLLILGSGPAGYTAAVYAARA
SHELP02018      --------------------------------MSQARHCNLLILGSGPAGYTAAVYAARA
SHEVD02297      --------------------------------MSQVRHCELLILGSGPAGYTAAVYAARA
SHEPW02341      --------------------------------MTQARHCNLLILGSGPAGYTAAVYAARA
```

SHEPA02202 --------------------------------MTQARHCDLLILGSGPAGYTAAVYAARA
SHEHH02007 --------------------------------MTQARHCELLILGSGPAGYTAAVYAARA
SHESH02117 --------------------------------MSQARHCELLILGSGPAGYTAAVYAARA
SHEWM02454 --------------------------------MSQARHCELLILGSGPAGYTAAVYAARA
SHEAM01758 --------------------------------MSQAKHSQLLILGSGPAGYTAAVYAARA
SHEON02159 --------------------------------MSQVRHSNLLILGSGPAGYTAAVYAARA
SHESM01945 --------------------------------MSQVRHSNLLILGSGPAGYTAAVYAARA
SHESR01991 --------------------------------MSQVRHSNLLILGSGPAGYTAAVYAARA
SHESA02025 --------------------------------MSQVRHSNLLILGSGPAGYTAAVYAARA
SHESW01953 --------------------------------MSQIRHCNLLILGSGPAGYTAAVYAARA
SHEPC01976 --------------------------------MSQIRHCNLLILGSGPAGYTAAVYAARA
SHEP201929 --------------------------------MSQIRHCNLLILGSGPAGYTAAVYAARA
SHEB502016 --------------------------------MSQVRHCNLLILGSGPAGYTAAVYAARA
SHEB802168 --------------------------------MSQVRHCNLLILGSGPAGYTAAVYAARA
SHEB202117 --------------------------------MSQVRHCNLLILGSGPAGYTAAVYAARA
SHEB902270 --------------------------------MSQVRHCNLLILGSGPAGYTAAVYAARA
SHEB602258 --------------------------------MSQVRHCNLLILGSGPAGYTAAVYAARA
SHEDO01680 --------------------------------MSEARHSNLLILGSGPAGYTAAVYAARA
SHEFN01895 --------------------------------MSEARHVNLLILGSGPAGYTAAVYAARA
PSEHT01677 --------------------------------MTEAKHCKLLILGSGPAGYTAAVYAARA
ALISL01611 --------------------------------MSNVKHSKLLILGSGPAGYTAAVYAARA
VIBF100893 --------------------------------MSNVKHSKLLILGSGPAGYTAAVYAARA
VIBFM00891 --------------------------------MSNVKHSKLLILGSGPAGYTAAVYAARA
OCESG01399 --------------------------------MSQAKHAKLLILGSGPAGYTAAVYAARA
VIBA701872 ----------MTRDI-PYNRVKFSSIFDTGVPMSNVKHCKLLILGSGPAGYTAAVYAARA
VIBVY01451 ------------------------------MQMSDMKHSKLLILGSGPAGYTAAVYAARA
VIBVU02545 --------------------------------MSDMKHSKLLILGSGPAGYTAAVYAARA
VIBVM01748 --------------------------------MSDMKHSKLLILGSGPAGYTAAVYAARA
VIBCH01157 --------------------------------MSNVKHSKLLILGSGPAGYTAAVYAARA
VIBCM01114 --------------------------------MSNVKHSKLLILGSGPAGYTAAVYAARA
VIBCJ02064 --------------------------------MSNVKHSKLLILGSGPAGYTAAVYAARA
VIBC300725 --------------------------------MSNVKHSKLLILGSGPAGYTAAVYAARA
FERBD01969 --------------------------------MSDVKHCQLLILGSGPAGYTAAVYAARA
VIBFN02085 --------------------------------MSDVKHCKLLILGSGPAGYTAAVYAARA
VIBTL01089 --------------------------------MSDVKHCNLLILGSGPAGYTAAVYAARA
VIBPA01248 --------------------------------MSDVKHCKLLILGSGPAGYTAAVYAARA
VIBAE02128 --------------------------------MSDVKHCKLLILGSGPAGYTAAVYAARA
VIBCB00599 --------------------------------MSDVKHCKLLILGSGPAGYTAAVYAARA
PANVC00701 --------------------------------MSTAKHSKLLILGSGPAGYTAAVYAARA
SHIBC02443 --------------------------------MSTAKHSKLLILGSGPAGYTAAVYAARA
ENTBF02792 -----------------------MSANNYGDLMGTAKHSKLLILGSGPAGYTAAVYAARA
KLEP700892 -----------------------MYANYYGDLMGTAKHSKLLILGSGPAGYTAAVYAARA
KLEPH01782 --------------------------------MGTAKHSKLLILGSGPAGYTAAVYAARA
KLEP303551 --------------------------------MGTAKHSKLLILGSGPAGYTAAVYAARA
KLEVT03420 --------------------------------MGTAKHSKLLILGSGPAGYTAAVYAARA
ENTAK02985 --------------------------------MGTAKHSKLLILGSGPAGYTAAVYAARA
KLEOK03151 --------------------------------MGTAKHSKLLILGSGPAGYTAAVYAARA
SALAR01925 --------------------------------MGTTKHSKLLILGSGPAGYTAAVYAARA
SALBC00812 LTKSLYSFFTSVNSL-QFCL---LSANNYGDLMGTTKHSKLLILGSGPAGYTAAVYAARA
SALPC00926 --------------------------------MGTTKHSKLLILGSGPAGYTAAVYAARA
SALTI00868 --------------------------------MGTTKHSKLLILGSGPAGYTAAVYAARA
SALCH00905 --------------------------------MGTTKHSKLLILGSGPAGYTAAVYAARA
SALPA01706 --------------------------------MGTTKHSKLLILGSGPAGYTAAVYAARA
SALTY00927 --------------------------------MGTTKHSKLLILGSGPAGYTAAVYAARA
SALPK01786 --------------------------------MGTTKHSKLLILGSGPAGYTAAVYAARA
SALHS00978 --------------------------------MGTTKHSKLLILGSGPAGYTAAVYAARA
SALEP00854 --------------------------------MGTTKHSKLLILGSGPAGYTAAVYAARA
SALDC00926 --------------------------------MGTTKHSKLLILGSGPAGYTAAVYAARA
SALA400892 --------------------------------MGTTKHSKLLILGSGPAGYTAAVYAARA
SALG200876 --------------------------------MGTTKHSKLLILGSGPAGYTAAVYAARA
SALTS00888 --------------------------------MGTTKHSKLLILGSGPAGYTAAVYAARA
SALT400905 --------------------------------MGTTKHSKLLILGSGPAGYTAAVYAARA
SALPS01908 --------------------------------MGTTKHSKLLILGSGPAGYTAAVYAARA
SALT101040 --------------------------------MGTTKHSKLLILGSGPAGYTAAVYAARA
SALTD00960 --------------------------------MGTTKHSKLLILGSGPAGYTAAVYAARA

```
SALPB02472      --------------------------------MGTTKHSKLLILGSGPAGYTAAVYAARA
SALNS00928      --------------------------------MGTTKHSKLLILGSGPAGYTAAVYAARA
SALSV01000      --------------------------------MGTTKHSKLLILGSGPAGYTAAVYAARA
ECOS500812      --------------------------------MGTTKHSKLLILGSGPAGYTAAVYAARA
ECOL600981      VNKIVILFFYVCKFP-TILP---LSANNYGDLMGTTKHSKLLILGSGPAGYTAAVYAARA
ECOL500883      --------------------------------MGTTKHSKLLILGSGPAGYTAAVYAARA
ECOUT00876      VNKIVILFFYVCKFP-TILP---LSANNYGDLMGTTKHSKLLILGSGPAGYTAAVYAARA
ECOK100776      VNKIVILFFYVCKFP-TILP---LSANNYGDLMGTTKHSKLLILGSGPAGYTAAVYAARA
ECOSM02148      --------------------------------MGTTKHSKLLILGSGPAGYTAAVYAARA
ECOLU01057      --------------------------------MGTTKHSKLLILGSGPAGYTAAVYAARA
ECO7I02136      --------------------------------MGTTKHSKLLILGSGPAGYTAAVYAARA
ECO8100828      --------------------------------MGTTKHSKLLILGSGPAGYTAAVYAARA
ECO4500862      --------------------------------MGTTKHSKLLILGSGPAGYTAAVYAARA
ECOAB00879      --------------------------------MGTTKHSKLLILGSGPAGYTAAVYAARA
ECO4400961      --------------------------------MGTTKHSKLLILGSGPAGYTAAVYAARA
ECOUM02550      --------------------------------MGTTKHSKLLILGSGPAGYTAAVYAARA
ECOKI00869      --------------------------------MGTTKHSKLLILGSGPAGYTAAVYAARA
ECOC100920      VNKIVILFFYVCKFP-TILP---LSANNYGDLMGTTKHSKLLILGSGPAGYTAAVYAARA
ECOC200920      VNKIVILFFYVCKFP-TILP---LSANNYGDLMGTTKHSKLLILGSGPAGYTAAVYAARA
ECO8N00803      --------------------------------MGTTKHSKLLILGSGPAGYTAAVYAARA
ECO2700862      --------------------------------MGTTKHSKLLILGSGPAGYTAAVYAARA
ECO2600974      --------------------------------MGTTKHSKLLILGSGPAGYTAAVYAARA
ECOH100942      --------------------------------MGTTKHSKLLILGSGPAGYTAAVYAARA
SHIB301848      --------------------------------MGTTKHSKLLILGSGPAGYTAAVYAARA
ECOLI00846      --------------------------------MGTTKHSKLLILGSGPAGYTAAVYAARA
ECO5700970      --------------------------------MGTTKHSKLLILGSGPAGYTAAVYAARA
SHISS00756      --------------------------------MGTTKHSKLLILGSGPAGYTAAVYAARA
SHIBS00679      --------------------------------MGTTKHSKLLILGSGPAGYTAAVYAARA
SHIDS01857      --------------------------------MGTTKHSKLLILGSGPAGYTAAVYAARA
ECO2400906      --------------------------------MGTTKHSKLLILGSGPAGYTAAVYAARA
ECODH00784      --------------------------------MGTTKHSKLLILGSGPAGYTAAVYAARA
ECOHS00922      --------------------------------MGTTKHSKLLILGSGPAGYTAAVYAARA
ECOLC02627      --------------------------------MGTTKHSKLLILGSGPAGYTAAVYAARA
ECO5E00946      --------------------------------MGTTKHSKLLILGSGPAGYTAAVYAARA
ECOSE00936      --------------------------------MGTTKHSKLLILGSGPAGYTAAVYAARA
ECO5500894      --------------------------------MGTTKHSKLLILGSGPAGYTAAVYAARA
ECO8A00877      --------------------------------MGTTKHSKLLILGSGPAGYTAAVYAARA
ECOBB00863      --------------------------------MGTTKHSKLLILGSGPAGYTAAVYAARA
ECO5T00943      --------------------------------MGTTKHSKLLILGSGPAGYTAAVYAARA
ECOBW00723      --------------------------------MGTTKHSKLLILGSGPAGYTAAVYAARA
ECO1000919      --------------------------------MGTTKHSKLLILGSGPAGYTAAVYAARA
ECOBD02604      --------------------------------MGTTKHSKLLILGSGPAGYTAAVYAARA
ECOD102659      --------------------------------MGTTKHSKLLILGSGPAGYTAAVYAARA
ECOBR00856      --------------------------------MGTTKHSKLLILGSGPAGYTAAVYAARA
ECOLX02610      --------------------------------MGTTKHSKLLILGSGPAGYTAAVYAARA
ECO1A00939      --------------------------------MGTTKHSKLLILGSGPAGYTAAVYAARA
ECOCB01054      --------------------------------MGTTKHSKLLILGSGPAGYTAAVYAARA
ECOKO02859      --------------------------------MGTTKHSKLLILGSGPAGYTAAVYAARA
ECO1E03320      --------------------------------MGTTKHSKLLILGSGPAGYTAAVYAARA
ECOLW01217      --------------------------------MGTTKHSKLLILGSGPAGYTAAVYAARA
SHIFL01399      --------------------------------MGTTKHSKLLILGSGPAGYTAAVYAARA
SHIF800759      --------------------------------MGTTKHSKLLILGSGPAGYTAAVYAARA
SHIF200805      --------------------------------MGTTKHSKLLILGSGPAGYTAAVYAARA
CITK802118      --------------------------------MGTTKHSKLLILGSGPAGYTAAVYAARA
CITRI00924      --------------------------------MGTTKHSKLLILGSGPAGYTAAVYAARA
CROS802385      --------------------------------MGTAKHSKLLILGSGPAGYTAAVYAARA
CROTZ01500      --------------------------------MGTAKHSKLLILGSGPAGYTAAVYAARA
ENTLS02865      -----------------------MSANNYGDLMGTAKHSKLLILGSGPAGYTAAVYAARA
ENT3801400      --------------------------------MGTAKHSKLLILGSGPAGYTAAVYAARA
ENTAL01384      -----------------------MSANNNGDLMGTAKHSKLLILGSGPAGYTAAVYAARA
ENTCC02679      --------------------------------MGTAKHSKLLILGSGPAGYTAAVYAARA
                                                              * ..     :

STRT101547      NLKVA-TLEQG--------------APGGQMNNTSDIENYPGF-E---------------
STRT201508      NLKVA-TLEQG--------------APGGQMNNTSDIENYPGF-E---------------
```

```
STRTD01356      NLKVA-TLEQG--------------APGGQMNNTSDIENYPGF-E---------------
STRTN01533      NLKVA-TLEQG--------------APGGQMNNTSDIENYPGF-E---------------
STRE500345      NLKVA-TLEQG--------------APGGQMNNTSDIENYPGF-E---------------
STRE801625      NLKVA-TLEQG--------------APGGQMNNTSDIENYPGF-E---------------
STREH01636      NLKVA-TLEQG--------------APGGQMNNTSDIENYPGF-E---------------
STREC01656      NLRVG-IIEQG--------------APGGQMNNTSDIENYPGY-D---------------
STREM01495      NLRVG-IIEQG--------------APGGQMNNTSDIENYPGY-D---------------
STRE401629      NLRVG-IIEQG--------------APGGQMNNTSDIENYPGY-D---------------
STRS700387      NLRVG-IIEQG--------------APGGQMNNTSDIENYPGY-D---------------
STRDG01631      NLRVG-IIEQG--------------APGGQMNNTSEIENYPGY-D---------------
STRP301388      NLSVA-IIEQG--------------APGGQMNNTFDIENYPGY-D---------------
STRPZ01205      NLSVA-IIEQG--------------APGGQMNNTFDIENYPGY-D---------------
STRPQ00464      NLSVA-IIEQG--------------APGGQMNNTFDIENYPGY-D---------------
STRPD01453      NLSVA-IIEQG--------------APGGQMNNTFDIENYPGY-D---------------
STRP601399      NLSVA-IIEQG--------------APGGQMNNTFDIENYPGY-D---------------
STRP801366      NLSVA-IIEQG--------------APGGQMNNTFDIENYPGY-D---------------
STRPF01453      NLSVA-IIEQG--------------APGGQMNNTFDIENYPGY-D---------------
STRPG00427      NLSVA-IIEQG--------------APGGQMNNTFDIENYPGY-D---------------
STRA300284      NLKVG-LIEQG--------------APGGQMNNTAEIENYPGY-D---------------
STRA500290      NLKVG-LIEQG--------------APGGQMNNTAEIENYPGY-D---------------
STRA100288      NLKVG-LIEQG--------------APGGQMNNTAEIENYPGY-D---------------
STRA200291      NLKVG-LIEQG--------------APGGQMNNTAEIENYPGY-D---------------
STRIC00348      NLKIG-IIEQG--------------APGGQMNNTSEIENYPGY-D---------------
STRPX00375      NLKVG-IIEQG--------------APGGQMNNTSEIENYPGY-D---------------
STRMD00432      NLKVG-IIEQG--------------APGGQMNNTSEIENYPGY-D---------------
STRS201734      NLKVA-LLERG--------------IYGGQMNNTAEIENYPGY-D---------------
STRSY01721      NLKVA-LLERG--------------IYGGQMNNTAEIENYPGY-D---------------
STRSX01553      NLKVA-LLERG--------------IYGGQMNNTAEIENYPGY-D---------------
STRSE01478      NLKVA-LLERG--------------IYGGQMNNTAEIENYPGY-D---------------
STREJ01635      NLKVA-LLERG--------------IYGGQMNNTAEIENYPGY-D---------------
STRGZ01544      NLKVA-LLERG--------------IYGGQMNNTAEIENYPGY-D---------------
STRS401593      NLKVA-LLERG--------------IYGGQMNNTAEIENYPGY-D---------------
LACGT00720      EMKTL-LLERG--------------VPGGQMNNTAEIENYPGY-G---------------
LACGL00738      EMKTL-LLERG--------------VPGGQMNNTAEIENYPGY-G---------------
STRSV01774      NLKVA-LLERG--------------IPGGQMNNTADIENYPGY-A---------------
STRIJ00350      NLKVA-LIERG--------------IPGGQMNNTSDIENYPGY-T---------------
STROU01248      NLKVA-LIEGG--------------LPGGQMNNTSDIENYPGY-A---------------
STRM601286      NLKVA-LIEGG--------------LPGGQMNNTSDIENYPGY-A---------------
STRES00962      NLKVA-LIEGG--------------LPGGQMNNTSDIENYPGY-A---------------
STRP701387      NLKVA-LIEGG--------------LPGGQMNNTSDIENYPGY-A---------------
STRZT00756      NLKVA-LIEGG--------------LPGGQMNNTSDIENYPGY-A---------------
STRP001079      NLKVA-LIEGG--------------LPGGQMNNTSDIENYPGY-A---------------
STRZO01270      NLKVA-LIEGG--------------LPGGQMNNTSDIENYPGY-A---------------
STRZ600815      NLKVA-LIEGG--------------LPGGQMNNTSDIENYPGY-A---------------
STRET00826      NLKVA-LIEGG--------------LPGGQMNNTSDIENYPGY-A---------------
STRPS01429      NLKVA-LIEGG--------------LPGGQMNNTSDIENYPGY-A---------------
STRZN01287      NLKVA-LIEGG--------------LPGGQMNNTSDIENYPGY-A---------------
STRR601306      NLKVA-LIEGG--------------LPGGQMNNTSDIENYPGY-A---------------
STRP201217      NLKVA-LIEGG--------------LPGGQMNNTSDIENYPGY-A---------------
STRZP01342      NLKVA-LIEGG--------------LPGGQMNNTSDIENYPGY-A---------------
STRZI01226      NLKVA-LIEGG--------------LPGGQMNNTSDIENYPGY-A---------------
STRPN01360      NLKVA-LIEGG--------------LPGGQMNNTSDIENYPGY-A---------------
STRP401357      NLKVA-LIEGG--------------LPGGQMNNTSDIENYPGY-A---------------
STRZJ01282      NLKVA-LIEGG--------------LPGGQMNNTSDIENYPGY-A---------------
STRPJ01336      NLKVA-LIEGG--------------LPGGQMNNTSDIENYPGY-A---------------
STRPI01436      NLKVA-LIEGG--------------LPGGQMNNTSDIENYPGY-A---------------
MARHT00044      ELKTV-IIEKG--------------LPGGQIAQTEEVENYPGFPE---------------
THEP300413      RLKTV-LIEKT--------------YLGGQIVNTYQLENYPGY-E---------------
THEPX00884      RLKTV-LIEKT--------------YLGGQIVNTYQLENYPGY-E---------------
THESX01857      RLKTV-LIEKT--------------YLGGQIVNTYQLENYPGY-E---------------
THEM301788      RLKTV-LIEKT--------------YLGGQIVNTYQLENYPGY-E---------------
THEIA01812      RLKTV-LIEKT--------------YLGGQIVNTYQLENYPGY-E---------------
THETC00443      KLDTV-MIEQM--------------YVGGQIVTTYEIENYPGF-D---------------
THESW01026      KLDTV-MIEQM--------------YVGGQIVTTYEIENYPGF-D---------------
THEXL00360      KLDTV-MIEQM--------------YVGGQIVTTYEIENYPGF-D---------------
```

```
THEID01127    RLNII-LVEKT--------------SPGGQVLITDFVENYPGFPD---------------
THEOJ00147    RLSTV-MIEKM--------------YPGGQAAITDIIENYPGFPE---------------
DESAS01237    DLKTV-LIEKG--------------VPGGLAATTEFIENYPGFSE---------------
DESK701373    KLKSL-LIERG--------------MTGGLAATTEFIENYPGFSE---------------
KYRT200729    GLKML-LVERG--------------LYGGQMQNTEEIENYPGF-T---------------
STACT00410    DLKTL-MIERG--------------VPGGQMANTEDVENFPGF-E---------------
STAS101952    NLSTV-MIERG--------------MPGGQMANTEEVENFPGF-E---------------
STALH01969    NLSTV-MIERG--------------MPGGQMANTEEVENFPGF-E---------------
STAEQ00422    NLKTV-MIERG--------------MPGGQMANTEEVENFPGF-E---------------
STAES00543    NLKTV-MIERG--------------MPGGQMANTEEVENFPGF-E---------------
STAAB00714    NLKTV-MIERG--------------IPGGQMANTEEVENFPGF-E---------------
STAA500765    NLKTV-MIERG--------------IPGGQMANTEEVENFPGF-E---------------
STAAM00757    NLKTV-MIERG--------------IPGGQMANTEEVENFPGF-E---------------
STAAW00726    NLKTV-MIERG--------------IPGGQMANTEEVENFPGF-E---------------
STAAS00733    NLKTV-MIERG--------------IPGGQMANTEEVENFPGF-E---------------
STAAN00727    NLKTV-MIERG--------------IPGGQMANTEEVENFPGF-E---------------
STAAC00807    NLKTV-MIERG--------------IPGGQMANTEEVENFPGF-E---------------
STAA300727    NLKTV-MIERG--------------IPGGQMANTEEVENFPGF-E---------------
STAA800734    NLKTV-MIERG--------------IPGGQMANTEEVENFPGF-E---------------
STAA100752    NLKTV-MIERG--------------IPGGQMANTEEVENFPGF-E---------------
STAA200774    NLKTV-MIERG--------------IPGGQMANTEEVENFPGF-E---------------
STAA900759    NLKTV-MIERG--------------IPGGQMANTEEVENFPGF-E---------------
STAAE00716    NLKTV-MIERG--------------IPGGQMANTEEVENFPGF-E---------------
STAAT00767    NLKTV-MIERG--------------IPGGQMANTEEVENFPGF-E---------------
STAAD00695    NLKTV-MIERG--------------IPGGQMANTEEVENFPGF-E---------------
STAA000815    NLKTV-MIERG--------------IPGGQMANTEEVENFPGF-E---------------
STAAH02407    NLKTV-MIERG--------------IPGGQMANTEEVENFPGF-E---------------
STAAF00763    NLKTV-MIERG--------------IPGGQMANTEEVENFPGF-E---------------
STAAK00744    NLKTV-MIERG--------------IPGGQMANTEEVENFPGF-E---------------
STAAJ00703    NLKTV-MIERG--------------IPGGQMANTEEVENFPGF-E---------------
STAAG00690    NLKTV-MIERG--------------IPGGQMANTEEVENFPGF-E---------------
STAA400732    NLKTV-MIERG--------------IPGGQMANTEEVENFPGF-E---------------
STAAR00788    NLKTV-MIERG--------------IPGGQMANTEEVENFPGF-E---------------
LISSS02378    DLDTL-MIERG--------------VPGGQMVNTAEVENYPGF-D---------------
LISIN02590    DLDTL-MIERG--------------VPGGQMVNTAEVENYPGF-D---------------
LISW602421    DLDTL-MIERG--------------VPGGQMVNTAEVENYPGF-D---------------
EXISA00870    NLSTL-MIERG--------------IPGGQMANTEDIENYPGY-D---------------
EXIS202358    NLSTL-MIERG--------------IPGGQMANTEDIENYPGY-D---------------
EXIAB02180    NLSTL-MIERG--------------IPGGQMANTEDIENYPGY-D---------------
OCEIH02462    NLDTL-MLERG--------------IPGGQMANTEDVENYPGF-D---------------
BACIE01058    NLDTV-MIERG--------------MPGGQMANTEDVENYPGY-D---------------
BACCJ03468    NLSTV-MIERG--------------VPGGQMANTEDVENYPGY-E---------------
BACHD03507    NLSTV-MVERG--------------VPGGQMANTEDVENYPGF-D---------------
BACPE03382    NLSTI-MLERG--------------VPGGQMANTEDVENYPGF-D---------------
SOLSS00743    NLSTL-MIERG--------------IPGGQMANTEAVENYPGF-D---------------
BACC600715    NLKTL-MIERG--------------VPGGQMVNTEEVENYPGY-E---------------
ANOFW02486    NLSTL-MLERG--------------VPGGQMANTEEVENYPGY-E---------------
GEOKA03042    NLSTL-MIERG--------------VPGGQMVNTEEVENYPGF-E---------------
GEOSY02976    NLSTL-MIERG--------------VPGGQMVNTEEVENYPGF-E---------------
GEOTN02952    NLSTL-MIERG--------------VPGGQMVNTEEVENYPGF-E---------------
GEOSW02588    NLSTL-MIERG--------------VPGGQMVNTEDVENYPGY-E---------------
GEOS000381    NLSTL-MIERG--------------VPGGQMVNTEEVENYPGY-E---------------
GEOTC00378    NLSTL-MIERG--------------VPGGQMVNTEEVENYPGY-E---------------
BACMD04906    DLSTL-MIERG--------------IPGGQMANTEDVENYPGY-D---------------
BACMQ04908    DLSTL-MIERG--------------IPGGQMANTEDVENYPGY-D---------------
BACWK04830    NLSTL-MLERG--------------IPGGQMANTEDVENYPGY-E---------------
BACAN04889    NLSTL-MLERG--------------IPGGQMANTEDVENYPGY-E---------------
BACC105210    NLSTL-MLERG--------------IPGGQMANTEDVENYPGY-E---------------
BACC705051    NLSTL-MLERG--------------IPGGQMANTEDVENYPGY-E---------------
BACC005112    NLSTL-MLERG--------------IPGGQMANTEDVENYPGY-E---------------
BACC305043    NLSTL-MLERG--------------IPGGQMANTEDVENYPGY-E---------------
BACAC05232    NLSTL-MLERG--------------IPGGQMANTEDVENYPGY-E---------------
BACAA04720    NLSTL-MLERG--------------IPGGQMANTEDVENYPGY-E---------------
BACT005085    NLSTL-MLERG--------------IPGGQMANTEDVENYPGY-E---------------
BACC205124    NLSTL-MLERG--------------IPGGQMANTEDVENYPGY-E---------------
```

BACC405064 NLSTL-MLERG--------------IPGGQMANTEDVENYPGY-E---------------
BACT104712 NLSTL-MLERG--------------IPGGQMANTEDVENYPGY-E---------------
BACLD03769 NLSTL-MVERG--------------VPGGQMANTEDVENYPGF-E---------------
BACPZ03376 NLSTL-MIERG--------------IPGGQMANTEDVENYPGF-E---------------
BACSU03600 NLSTL-MIERG--------------IPGGQMANTEDVENYPGF-E---------------
BACST01653 NLSTL-MIERG--------------IPGGQMANTEDVENYPGF-E---------------
BACPT03541 NLSTL-MIERG--------------IPGGQMANTEDVENYPGF-E---------------
LEUGG00661 NLSVL-MLDRG--------------IYGGQMNNTAEVENYPGF-D---------------
LEUGJ00629 NLSVL-MLDRG--------------IYGGQMNNTAEVENYPGF-D---------------
LEUCJ00485 NLSVL-MLDRG--------------IYGGQMNNTAEVENYPGF-N---------------
LACAR00657 NLNVL-VLDRG--------------PYGGQMNNTDAIDNYPGF-T---------------
LACA300647 NLNVL-VLDRG--------------PYGGQMNNTDAIDNYPGF-T---------------
LACAL00643 NLNVL-VLDRG--------------PYGGQMNNTDAIDNYPGF-T---------------
LACKZ00964 NLNVL-VLDRG--------------PYGGQMNNTDAIDNYPGF-T---------------
LACRJ00357 NLSVL-MLDRG--------------IYGGNLNNTAEIENYTGF-K---------------
LACRD00361 NLSVL-MLDRG--------------IYGGNLNNTAEIENYTGF-K---------------
LACRS01501 NLSVL-MLDRG--------------IYGGNLNNTAEIENYTGF-K---------------
LACSM00455 ELSVL-MLDRG--------------VYGGQMNNTAEIENYPGF-K---------------
LACRG00878 NLSVL-MLDRG--------------VYGGQMNNTAEVENYPGY-K---------------
LACRL00939 NLSVL-MLDRG--------------VYGGQMNNTAEVENYPGY-K---------------
LACC300844 NLSVL-MLDRG--------------VYGGQMNNTAEVENYPGF-K---------------
LACCZ00824 NLSVL-MLDRG--------------VYGGQMNNTAEVENYPGF-K---------------
LACCB00988 NLSVL-MLDRG--------------VYGGQMNNTAEVENYPGF-K---------------
LACCD01016 NLSVL-MLDRG--------------VYGGQMNNTAEVENYPGF-K---------------
LACCC01014 NLSVL-MLDRG--------------VYGGQMNNTAEVENYPGF-K---------------
LACBN01277 NLSVA-MIDRG--------------IYGGQMNNTAAIENYPGF-K---------------
LACBA00605 NLSVL-MLDRG--------------IYGGQMNNTAAIENYPGF-K---------------
LACPL00650 NLSVL-LLDRG--------------IYGGQMNNTAAIENYPGF-K---------------
LACPJ00628 NLSVL-LLDRG--------------IYGGQMNNTAAIENYPGF-K---------------
LACPS00585 NLSVL-LLDRG--------------IYGGQMNNTAAIENYPGF-K---------------
PEDCP00497 NMSVL-MLDRG--------------IYGGQMNNTASIENYPGF-K---------------
CARS100359 NLSTL-MLERG--------------VPGGQMINTAEIENYPGF-N---------------
AERUA00263 NLQVA-VLERG--------------VPGGELINTATVENYPGY-K---------------
ELUMP00590 ALDVL-VFAGP--------------ISGGQILQTNDMENYPGFIE---------------
SPHPG02940 GRSVT-LIEEM--------------APGGQTMYIDMVENYPGFDK---------------
SPHGB01868 GRSVT-LLESI--------------APGGQTMYIDMIENYPGFDQ---------------
TREPZ00273 NLKVL-AIEQL--------------SPGGQALSIDKLENYPGDPT---------------
TREAZ03414 NLRVL-VLEQM--------------APGGQVLLIDILENYPGIAQ---------------
SPITD00734 ALDTL-VIEQM--------------APGGQALIIDTLENYPGFPE---------------
SPITZ01373 ALDTL-VIEQM--------------APGGQALIIDTLENYPGFPE---------------
TREPA00803 NLRTL-VIESK--------------AHGGQALLIDSLENYPGYAT---------------
TREPS00802 NLRTL-VIESK--------------AHGGQALLIDSLENYPGYAT---------------
TREPC00747 NLRTL-VIESK--------------AHGGQALLIDSLENYPGYAT---------------
TREPM00823 NLRTL-VIESK--------------AHGGQALLIDSLENYPGYAT---------------
TREPD00824 NLRTL-VIESK--------------AHGGQALLIDSLENYPGYAT---------------
TREPU00781 NLRTL-VIESK--------------AHGGQALLIDSLENYPGYAT---------------
ENCCU00216 E--PL-LFEGGYI---------GNNGPGGQLTTTTSVDNYPGFPD---------------
HELM100964 GIKNVVLFEKG--------------MPGGQITGSSEIENYPGVKE---------------
HELCP01490 GLKNVVLFEKG--------------MPGGQITGSSEIENYPGVKE---------------
ARCFU01526 GLKTV-FFETV--------------DPVSQLSLAAKIENYPGFEG---------------
FERPA02445 GLKTV-VFETV--------------EA-SQLSLAPKIENYPGFEG---------------
ARCVS01910 GLRTV-FFESM---------------P-SQLAVVPFIENYPGFEG---------------
METEZ00677 GLDTY-LLERT--------------AIGGQISSSQEVENYPGFSS---------------
METHD00869 GLNTI-VLEKD--------------VLPGQIAATDLIENYTGFTA---------------
METMA02304 GLDTL-ILERN--------------EISGQISMADIVENYPGFPS---------------
METAC01311 GLDTL-VLEKS--------------EISGQISMSDIVENYPGFPS---------------
KOSOT00298 GLDPL-IIERA--------------LEGGQINNTEKVENWTGFPS---------------
MARPK01613 GIQPL-IIEKD--------------LEGGQINLTEYVENYPGFKS---------------
SLAHD02439 RMNAL-LIEKG--------------QDGGQIATTSEIENYPGQAV---------------
FILAD00976 KMKAL-MIEKG--------------QHGGQIMSTNSIENYPGSIA---------------
BUTPB02463 GLNMI-IIEKN-------------PVSGGQIIDTYEVDNYLGIPG---------------
CLOPH00250 ELNTI-VIEKE-------------PMSGGQIINTYEVDNYPGIPG---------------
CLOSW00592 ELKMV-VIEKE-------------MASGGQVLNTYEVDNYPGLPG---------------
LACFC00207 NLSVL-LLDRG--------------IYGGNLNNTATIENYTGFKT---------------
CRYCD00853 GLSVV-MFEEM--------------MPGGQLAQTDKIENYPGFPG---------------

```
EGGLE01847      GLNVV-LFERI--------------SPGGQLAQTEHMENYPGFPE---------------
PYRFU01410      GLDTL-IITKD--------------L-GGNMALTDLIENYPGFPE---------------
PYRHO01476      GLDTI-IITKD--------------L-GGNMAITDLIENYPGFPE---------------
PYRAB00730      GLDTI-IITKD--------------L-GGNMAITDLIENYPGFPE---------------
PYRSN00014      GLDTI-IITKD--------------L-GGNMAITDLIENYPGFPE---------------
THEGJ00181      GLETL-ILSKD--------------L-GGNMALTDLIENYPGFPE---------------
THEKO02097      GLETL-IISKD--------------L-GGNMALTDLIENYPGFPE---------------
THEON01610      GLETL-IISKD--------------L-GGNMALTDLIENYPGFPE---------------
THES401476      GLETL-ILSKD--------------L-GGNMALTDMIENYPGFPE---------------
SYNWW02368      KLKTL-VLESS--------------VFGGNAALIDHIDNYPGFPF---------------
UNCTG00012      RLKTL-IIEKT--------------GCGGQMTTTDLLENYPGFNG---------------
THEA101460      QLNTL-ILDQM--------------MPGGQLLITEQIENYPGFYE---------------
DENA201647      KQTVL-VLEKQ--------------FPGGFVAITEWVENYPGFFD---------------
DEFDS00488      DLNTL-ILEKN--------------FPGGQVAITEIIENYPGFLE---------------
CALNY01292      NLKTL-ILEKN--------------YPGGQVAITEIIENYPGFPD---------------
SYNGF01652      GLKTA-VLESM--------------MPGGQAALTEKIDNYPGFPD---------------
DESB201123      RLDVV-LYERL--------------SPGGQVLSTDWVENWPGAVE---------------
DEIPM00845      ELKTL-ILERG--------------LPGGQIAQTEEVENYPGFPE---------------
DEIRA01924      QLSTL-ILEKG--------------MPGGQIAWSEEVENFPGFPE---------------
DEIML01437      NLKTL-ILEKG--------------QPGGQIAQTEEVENYPGFPE---------------
DEIGD02532      SLSTL-VLEKG--------------LPGGQIAQTEEVENYPGFPE---------------
DEIDV00622      SLSTV-ILEKG--------------LPGGQIAQTEEVENYPGFPE---------------
TRURR01079      QLSTL-ILEKG--------------LPGGQIAQTQEVENYPGFDE---------------
THETG01863      QLKTV-IVEKG--------------LPGGQIAQTDEVENYPGFPE---------------
THET201543      QLKTV-IVEKG--------------LPGGQIAQTDEVENYPGFPE---------------
THET801911      QLKTV-IVEKG--------------LPGGQIAQTDEVENYPGFPE---------------
OCEP502115      GLKTV-ILEKG--------------LPGGQIAQTEEVENYPGFPE---------------
DEHLB00585      KLKTA-IIEGS--------------AIGGRMAEAWEIENYPGFVE---------------
DEHMG00439      KLKTL-VIERA--------------FVGGQITRSEKVDNYPGFPE---------------
DEHMB00497      KLKTL-VIERA--------------FVGGQITRSEKVDNYPGFPE---------------
DEHMC00427      KLKTL-VIERA--------------FVGGQITRSEKVDNYPGFPE---------------
THELD00726      GLDVL-VLEKG--------------VPGGQINLTDEIENWTGVIH---------------
ANAMD00624      GLDVL-VLEKG--------------IAGGQITVTAEIENWPGTPM---------------
THEAS00815      GLDVL-IIERG--------------MPGGQITITDEIENWPGVQH---------------
AMICL00707      GLDVL-LIEKG--------------VPGGQINITAEIENWPGVAH---------------
CLOCE01684      QLDTL-VIEKM--------------FSGGQMATTDVMENYPGFEE---------------
HYDS000616      KMDVI-LLEKS--------------TLGGQIAITDLVENYPGFPE---------------
HYDTT00224      KLNTL-LLEKG--------------TIGGQIAITDLVENYPGFPE---------------
PELTS01405      KLDTV-LLERG--------------IPGGLVISTDQVENYPGFPE---------------
DESRL02179      DIDTM-LVERG--------------MPGGQAASTEWIENYPGFPG---------------
BORBP00506      NYKVT-ILEGP--------------EPGGQLTTTTEVYNYPGFKN---------------
BORAP00518      NYKAT-ILEGP--------------EPGGQLTTTTEVYNYPGFKN---------------
BORBU00514      NYKAA-ILEGP--------------EPGGQLTTTTEVYNYPGFKN---------------
BORBZ00490      NYKAA-ILEGP--------------EPGGQLTTTTEVYNYPGFKN---------------
BORBN00493      NYKAA-ILEGP--------------EPGGQLTTTTEVYNYPGFKN---------------
BORRA00486      GYKTS-ILEGP--------------EPGGQLTTTTEVYNYPGFKN---------------
BORDL00498      GYKTS-ILEGP--------------EPGGQLTTTTEVYNYPGFKN---------------
BORHD00497      GYKTV-VLEGP--------------EPGGQLTTTTEVYNYPGFKN---------------
BORT900497      GYKTV-ILEGP--------------EPGGQLTTTTEVYNYPGFKN---------------
METKA01560      GAEVP-VVAQE---------------LGGQLTTAPEVGNYPGFP----------------
MYCA500361      NMDVA-FIESY--------------APGGKIIEQSKIENYPGFD----------------
METVS00145      RLNVL-CIEKE--------------NEGGKIAEAGIVENYPGFK----------------
METOI01431      KLNTI-CIEKE--------------NEGGKIAEAGIVENYPGFD----------------
MYCHN00051      CLKTA-FIEKD--------------WPGGKLNKTLYIENYPGYL----------------
MYCSL00465      CLNVL-ILEKA--------------LVGGKLTKTLFIDNYPGYL----------------
MYCS300420      CLNVL-ILEKA--------------LVGGKLTKTLFIDNYPGYL----------------
DESK101258      GFKTI-IVTKL---------------VGGAVSEAPLVDDYPGIP----------------
STAHD01472      QLQAI-VITKL---------------LGGLVTEAPIVDDYPGLP----------------
THEC100270      ELKTV-VVTKL---------------VGGYVTEATIVDDYPGLP----------------
NANEQ00478      GLKTL-LISPD---------------KGGMLTEASFIENYPGFI----------------
CALLD01225      LLKSA-VVSED---------------IGGQLNLTDVVDDYPGTL----------------
SULSO02155      MLKTL-VIGET---------------PGGQLTEAGIVDDYLGLI----------------
SULS900210      MLKTL-VIGET---------------PGGQLTEAGIVDDYLGLI----------------
SULIA00208      MLKTL-VIGET---------------PGGQLTEAGIVDDYLGLI----------------
SULIM00207      MLKTL-VIGET---------------PGGQLTEAGIVDDYLGLI----------------
```

```
SULIK00225      MLKTL-VIGET---------------PGGQLTEAGIVDDYLGLI----------------
IGNH400907      GLKTV-VLEKD-------------NKPGGRTSLAPVVEDYPGID----------------
KORCO01040      GLKVL-VIEKS--------------VPGGKININPVIENYPGFE----------------
THESM01133      GFDTL-IISKD--------------IGG-NVALTDIIENYPGFPE---------------
THEBM01534      GLETI-IISKD--------------LGG-NMALTDLIENYPGFPE---------------
METST01363      GLNTI-ILDEN--------------QSGGTVNVAPLIENYPGIN----------------
METTH00703      GSSVL-MLDKG--------------PAGGLGLEVPMMENYPGFE----------------
METSL02406      GLKSA-VIEKN--------------LRGGNANTAPVILNYPGYK----------------
METLA02406      GLKSA-VIEKN--------------LRGGNANTAPVILNYPGYK----------------
METPW00194      GLKAI-ILEKA--------------LKGGMGNVAPLVLNFPGFK----------------
RUBXD00226      NLRTL-VFQGFE--------------AGGQLMLTTDVENYPGYPE---------------
RHOM400178      NLAPV-VFRGPE--------------PGGQLITTTEVENYPGFPE---------------
GARV400051      GYKPL-VIAGALT-------------PGGQLMNTTEVENYPGFPE---------------
BIFAP01644      GYAPL-VIAGALT-------------PGGQLMNTTEVENFPGFPK---------------
SEGRD00014      QLNPL-VFEGTQ--------------FGGSLMTTTEVENFPGFQE---------------
GORB404535      DLAPI-VFEGTQ--------------FGGALMTTTEVENFPGFQN---------------
GORPV04920      ELAPL-VFEGTQ--------------FGGALMTTTEVENFPGFQN---------------
MYCA904903      QLKPI-VFEGTQ--------------FGGALMTTTEVENYPGFRE---------------
MYCSS05356      QLKPL-VFEGSQ--------------FGGALMTTTEVENYPGFRN---------------
MYCSJ05684      QLKPL-VFEGSQ--------------FGGALMTTTEVENYPGFRN---------------
MYCSK05387      QLKPL-VFEGSQ--------------FGGALMTTTEVENYPGFRN---------------
MYCS206581      QLKPL-VFEGTQ--------------FGGALMTTTEVENYPGFRE---------------
MYCCN05158      QLQPL-VFEGTQ--------------FGGALMTTTEVENYPGFRD---------------
MYCVP05890      QLNPL-VFEGSQ--------------FGGALMTTTEVENYPGFRN---------------
MYCGI00775      QLTPL-VFEGSQ--------------FGGALMTTTEVENYPGFRN---------------
MYCSR04994      QLTPL-VFEGSQ--------------FGGALMTTTEVENYPGFRN---------------
AMYSD04545      QLNPL-MFEGTQ--------------FGGALMTTTEVENFPGFRE---------------
MYCLE02681      QLTPL-VFEGTS--------------FGGALMTTTEVENYPGFRN---------------
MYCLB02681      QLTPL-VFEGTS--------------FGGALMTTTEVENYPGFRN---------------
MYCSD04323      QLAPL-VFEGTS--------------FGGALMTTTEVENFPGFRN---------------
MYCPA04306      QLAPV-VFEGTS--------------FGGALMTTTEVENYPGFRD---------------
MYCA105023      QLAPV-VFEGTS--------------FGGALMTTTEVENYPGFRD---------------
MYCUA04107      QLAPL-VFEGTS--------------FGGALMTTTEVENYPGFRE---------------
MYCMM05378      QLAPL-VFEGTS--------------FGGALMTTTEVENYPGFRE---------------
MYCA003905      QLAPL-VFEGTS--------------FGGALMTTTGVENYPGFRN---------------
MYCTU03943      QLAPL-VFEGTS--------------FGGALMTTTDVENYPGFRN---------------
MYCTF03846      QLAPL-VFEGTS--------------FGGALMTTTDVENYPGFRN---------------
MYCTA03980      QLAPL-VFEGTS--------------FGGALMTTTDVENYPGFRN---------------
MYCTK04010      QLAPL-VFEGTS--------------FGGALMTTTDVENYPGFRN---------------
MYCTC03612      QLAPL-VFEGTS--------------FGGALMTTTDVENYPGFRN---------------
MYCTD03548      QLAPL-VFEGTS--------------FGGALMTTTDVENYPGFRN---------------
MYCCP03898      QLAPL-VFEGTS--------------FGGALMTTTDVENYPGFRN---------------
MYCBO02863      QLAPL-VFEGTS--------------FGGALMTTTGVENYPGFRN---------------
MYCBP03913      QLAPL-VFEGTS--------------FGGALMTTTGVENYPGFRN---------------
MYCBT03913      QLAPL-VFEGTS--------------FGGALMTTTGVENYPGFRN---------------
TSUPD04078      ELKTI-LFEGTN--------------FGGALMTTTEVENYPGFKS---------------
ACTMD06897      QLEPL-VFEGSQ--------------YGGALMTTTEVENFPGFRD---------------
SACES08402      QLEPL-VFEGSQ--------------YGGALMTTTEVENYPGFRD---------------
SACVD03814      QLEPL-VFEGTQ--------------FGGALMTTTEVENYPGFRS---------------
AMYMU09190      QLEPL-VFEGTQ--------------FGGALMTTTEVENFPGFRD---------------
AMYMS10178      QLEPL-VFEGTQ--------------FGGALMTTTEVENFPGFRD---------------
PSEUX06421      QLAPL-VFEGSQ--------------FGGALMTTTEVENYPGFTD---------------
NOCFA05681      ELKPL-LFEGTQ--------------FGGALMTTTEVENFPGFRN---------------
NOCCG05476      ELQPL-LFEGTQ--------------FGGALMTTTEVENFPGFRD---------------
RHOE406010      ELQPL-LFEGTQ--------------FGGALMTTTEVENFPGFRE---------------
RHOOB03468      ELEPL-QFEGTQ--------------FGGALMTTTEVENFPGFRE---------------
RHOE104515      ELEPL-LFEGTQ--------------FGGSLMTTTEVENFPGFRE---------------
CORDI02302      ELKPI-VFEGIE--------------YGGSLMTTTEVENFPGFPE---------------
CORD202219      ELKPI-VFEGIE--------------YGGSLMTTTEVENFPGFPE---------------
CORDL02208      ELKPI-VFEGIE--------------YGGSLMTTTEVENFPGFPE---------------
CORDJ02208      ELKPI-VFEGIE--------------YGGSLMTTTEVENFPGFPE---------------
CORDH02221      ELKPI-VFEGIE--------------YGGSLMTTTEVENFPGFPE---------------
CORD702316      ELKPI-VFEGIE--------------YGGSLMTTTEVENFPGFPE---------------
CORD302337      ELKPI-VFEGIE--------------YGGSLMTTTEVENFPGFPE---------------
CORDD02235      ELKPI-VFEGIE--------------YGGSLMTTTEVENFPGFPE---------------
```

```
CORDV02170      ELKPI-VFEGIE--------------YGGSLMTTTEVENFPGFPE---------------
CORDW02254      ELKPI-VFEGIE--------------YGGSLMTTTEVENFPGFPE---------------
CORDK02230      ELKPI-VFEGIE--------------YGGSLMTTTEVENFPGFPE---------------
COREF02870      DLKPI-MFEGYE--------------YGGSLMTTTDVENFPGFQD---------------
CORGL03082      DLNPI-MFEGYE--------------YGGSLMTTTDVENFPGFEK---------------
CORGK02974      DLNPI-MFEGYE--------------YGGSLMTTTDVENFPGFEK---------------
CORGB03038      DLNPI-MFEGYE--------------YGGSLMTTTDVENFPGFEK---------------
CORK402006      NLKPL-VFEGIE--------------YGGLLMQTTEVENYPGFHD---------------
CORJK02028      ELNPV-VFEGVE--------------YGGLLMQTTEVENFPGFQK---------------
CORVD02951      ELKPL-VFEGMD--------------FGGLLMQTTEVENFPGFPA---------------
ARCHD01706      GLSPL-VLAGTLE-------------AGGALMNTTEVENFPGWPE---------------
THET101205      NLSPL-VLAGSQ--------------MGGQLMITTDVENYPGFPD---------------
PROAC02247      GLKPL-VFEGSMD-------------AGGALMQTTEVENYPGFSE---------------
PROAS02302      GLKPL-VFEGSMD-------------AGGALMQTTEVENYPGFSE---------------
CAERE29798      GLAPV-VIAGSVT-------------AGGALMTTTEVENFPGFVD---------------
CELFA03749      GLAPL-VVAGSVT-------------AGGALMNTTEVENFPGFPD---------------
JONDD02484      GLSPL-VLAGSIT-------------AGGALMNTTEVENFPGFPD---------------
XYLCX03324      GLAPV-VIAGSVT-------------AGGALMNTTEVENFPGFPE---------------
ACIC102144      KLQPL-VFEGSVG-------------AGGALMTTTEVENFPGFPD---------------
FRADG04042      NLRPL-VFEGAVS-------------AGGALMTTTEVENFPGFPE---------------
FRASU07065      SLHPL-VFEGAVS-------------AGGALMTTTEVENFPGWPD---------------
FRASN07115      NLKPV-VFEGAVS-------------AGGALMTTTEVENFPGFPE---------------
FRASC04424      NLRPL-VFEGAVS-------------AGGALMNTTEVENFPGFPD---------------
FRAAA06712      NLRPL-VFEGAVA-------------AGGALMNTTEVENFPGFPE---------------
KYTSD02521      NLRPL-VFEGSVT-------------MGGALMNTTDVENYPGFTD---------------
KINRD04462      ELRPL-VFEGSVT-------------AGGALMNTTEVENFPGFPE---------------
NOCDD04789      ELRPL-VFEGSIT-------------AGGALMNTTEVENFPGFPD---------------
NOCAA01941      ELRPL-VFEGSIT-------------AGGALMNTTEVENFPGFPD---------------
STRRD08913      DLKPL-IFEGSVT-------------AGGALMNTTEVENFPGFPD---------------
THECD04863      DLKPL-VFEGSVT-------------AGGALMNTTEVENFPGFPD---------------
THEBD03528      DLKPL-VFEGSVT-------------AGGALMNTTEVENFPGFPD---------------
CATAD08894      GLNPL-VFAGSVT-------------AGGALMNTTDVENFPGFPE---------------
KRIFD06917      QLEPL-VFEGSVT-------------AGGALMTTTEVENYPGFSQ---------------
KITSK03730      SLKPL-VFEGAVT-------------AGGALMNTTEVENFPGYRD---------------
STRBB05366      SLKPL-VFEGSVT-------------AGGALMNTTDVENFPGFRE---------------
STRVP03660      SLNPL-VFEGAVT-------------AGGALMNTTEVENFPGFRD---------------
STRSW04381      SLRPL-VFEGAVT-------------AGGALMNTTEVENFPGFRD---------------
STRGG03562      SLNPL-VFEGAVT-------------AGGALMNTTDVENFPGFQD---------------
STRFA03098      SLNPL-VFEGAVT-------------AGGALMNTTDVENFPGFQD---------------
STRAW04303      SLKPL-VFEGAVT-------------AGGALMNTTEVENFPGFQD---------------
STRCO02929      SLKPL-VFEGAVT-------------AGGALMNTTEVENFPGFQD---------------
STRHJ05105      SLKPL-VFEGAVT-------------AGGALMNTTEVENFPGFRD---------------
MONBE04991      ELKPV-MFEGFLA---------NDVAAGGQLTTTTDVENFPGFPE---------------
CHLRE01313      ELQPI-LFEGFMA---------NGIAAGGQLTTTTDVENFPGFPE---------------
MEDTR25591      ELKPI-LFEGWMA---------NDIAPGGQLTTTTDVENFPGFPD---------------
SOLLC13750      ELKPI-LFEGWMA---------NDIAPGGQLTTTTDVENFPGFPE---------------
PRUPE10733      ELKPI-LFEGWMA---------NDIAPGGQLTTTTDVENFPGFPD---------------
MANES18605      ELKPI-LFEGWMA---------NDIAPGGQLTTTSEVENFPGFPD---------------
THECC00884      ELKPI-LFEGWMA---------NDIAPGGQLTTTTDVENFPGFPD---------------
PHYPA31147      ELHPV-MLEGWLA---------NGIAAGGQLTTTTDVENFPGFPD---------------
AMBTC19471      ELKPL-LFEGWMA---------NGIAPGGQLTTTSEVENFPGFPE---------------
MUSAC26038      ELKPI-LFEGWMA---------NDIAAGGQLTTTTDVENFPGFPE---------------
MUSAM33177      ELKPI-LFEGWMA---------NDIAAGGQLTTTTDVENFPGFPE---------------
SETIT03079      ELKPV-LFEGWMA---------NDIAAGGQLTTTTDVENFPGFPD---------------
ORYBR12195      ELKPV-LFEGWLA---------NDIAAGGQLTTTTDVENFPGFPE---------------
COCLU07729      DLNPV-MYEGFMA---------LGIAAGGQLTTTDEVENFPGFT----------------
PHANO13702      DLKPV-MYEGFMA---------LGIAAGGQLTTTDEVENFPGFL----------------
PHAND10804      DLKPV-MYEGFMA---------LGIAAGGQLTTTDEVENFPGFL----------------
AURPU02089      DLQPV-LYEGFLA---------LGIAAGGQLTTTDEVENFPGFM----------------
ZYMTR07711      DLKPV-LYEGFMA---------LGIAAGGQLTTTDEVENFPGFK----------------
DICPU05926      KLNPL-MFEGFMA---------AGVAAGGQLTTTTEIENFPGFPT---------------
ENTHI00522      SLKPV-MYEGFMA---------GGVAAGGQLTTTTIIENFPGFPN---------------
LEPBA02231      NLNPV-MYEGFMA---------GGVAAGGQLTTTTEVENFPGFPE---------------
LEPBP02301      NLNPV-MYEGFMA---------GGVAAGGQLTTTTEVENFPGFPE---------------
LEPBL01462      NLNPV-MYEGFMA---------GGIAAGGQLTTTTEVENFPGFPN---------------
```

```
LEPBJ01276      NLNPV-MYEGFMA---------GGIAAGGQLTTTTEVENFPGFPN---------------
LEPIN02475      NLNPV-MYEGFMA---------GGIAAGGQLTTTTEVENFPGFPE---------------
LEPII01996      NLNPV-MYEGFMA---------GGIAAGGQLTTTTEVENFPGFPE---------------
LEPIC01426      NLNPV-MYEGFMA---------GGIAAGGQLTTTTEVENFPGFPE---------------
SPIAZ00697      ELQPL-LFEGFMA---------GGVAAGGQLTTTTEVENFPGFPE---------------
PENRW10140      ELQPV-LYEGMLA---------NGTAAGGQLTTTTDVENFPGFPS---------------
PENCH09104      ELQPV-LYEGMLA---------NGTAAGGQLTTTTDVENFPGFPS---------------
EURHE07269      ELKPV-LYEGMLA---------NGTAAGGQLTTTTDIENFPGFPD---------------
ASPAC07301      ELKPV-LYEGMLA---------NGTAAGGQLTTTTDIENFPGFPD---------------
EMENI10387      ELKPV-LYEGMLA---------NGTAAGGQLTTTTDVENFPGFPD---------------
EMEND02596      ELKPV-LYEGMLA---------NGTAAGGQLTTTTDVENFPGFPD---------------
ASPTN06742      ELQPV-LYEGMLA---------NGTAAGGQLTTTTDIENFPGFPD---------------
ASPCL04014      ELKPT-LYEGMLA---------NGTAAGGQLTTTTDIENFPGFPD---------------
ASPFU05647      ELKPV-LYEGMLA---------NGTAAGGQLTTTTDIENFPGFPD---------------
NEOFI00452      ELKPV-LYEGMLA---------NGTAAGGQLTTTTDIENFPGFPD---------------
CRYPA10563      NLEPT-LYEGFMA---------NGIAAGGQLTTTTDVENFPGFPD---------------
BLUGR03498      ELKPV-LYEGFLA---------NGIAPGGQLTTTTDVENYPGFPT---------------
SCLS112814      ELKP------------------NGIAAGGQLTTTTDVENFPGFPK---------------
MAGGR04266      NLKPV-MYEGFMA---------NGTAAGGQLTTTTDVENFPGFPK---------------
NEUCR01575      ELKPV-LYEGFMA---------NGIAAGGQLTTTTEIENFPGFPD---------------
NEUT908941      ELKPV-LYEGFMA---------NGIAAGGQLTTTTEIENFPGFPD---------------
VERDA02342      DMKPV-LYEGFLA---------NGIAAGGQLTTTTDVENFPGFPD---------------
COLSU12486      ELKPV-LYEGFMA---------NGIAAGGQLTTTTDVENFPGFPK---------------
HYPAI01684      ELKPV-LYEGFMA---------NGTAAGGQLTTTTEVENFPGFPK---------------
HYPVG06080      ELKPV-LYEGFMA---------NGTAAGGQLTTTTEIENFPGFPK---------------
HYPJE05895      ELKPV-LFEGFMA---------NGTAAGGQLTTTTEIENFPGFPK---------------
NECHA05020      ELKPV-LYEGFMA---------NGIAAGGQLTTTTDVENFPGFPK---------------
FUSO415847      ELKPV-LYEGFMA---------NGIAAGGQLTTTTEVENFPGFPK---------------
GIBZA01026      ELKPV-LYEGFMA---------NGIAAGGQLTTTTEVENFPGFPK---------------
SCHPO04025      ELKPV-MYEGMLA---------NGIAAGGQLTTTTDVENFPGFPD---------------
YARLI03635      EIKPT-LYEGMMA---------NGIAAGGQLTTTTEIENFPGFPD---------------
ASHGO00946      EIKPT-LYEGMFA---------NGVAAGGQLTTTTEIENFPGFPD---------------
KLULA02190      EIKPT-LYEGFMA---------NGIAAGGQLTTTTEIENFPGFPD---------------
ZYGRO00676      EIKPT-LYEGMMA---------NGIAAGGQLTTTTDIENFPGFPQ---------------
DEKBR01813      ELKPV-LYEGMMA---------NGFAPGGQLTTTTEVENFPGFPA---------------
PICPG04776      EMKPT-LYEGMLA---------NGIAAGGQLTTTTDVENFPGFPE---------------
CANTE00916      EVKPV-LYEGMLA---------NGFAAGGQLTTTTDIENFPGFPE---------------
LODEL03891      EIKTT-LYEGMLA---------NGVAAGGQLTTTTDIENFPGFPQ---------------
DEBHA05546      EIKPT-LYEGMLA---------NGTAAGGQLTTTTDVENFPGFPK---------------
SPAPN03477      EIKPT-LYEGMLA---------NGIAAGGQLTTTTDIENFPGFPE---------------
CANAW04800      EIKPT-LYEGMLA---------NGIAAGGQLTTTTDIENFPGFPN---------------
PICST04701      EIKPT-MYEGMLA---------NGIAAGGQLTTTTDIENFPGFPN---------------
PUCGT10887      NLEPV-MFEGMLA---------NGFAPGGQLTTTTDVENFPGFPE---------------
PUCGR11813      NLEPV-MFEGMLA---------NGFAPGGQLTTTTDVENFPGFPE---------------
PHYBL11006      NLNPF-MFEGMMA---------NGFAPGGQLTTTTDVENFPGFPE---------------
USTMA03757      NLEPV-LFEGMLA---------NGLAPGGQLTTTTDVENFPGFPE---------------
USTHO04132      NLEPV-LFEGMLA---------NGLAPGGQLTTTTDVENFPGFPD---------------
WALSE04527      EMQPV-LFEGMLA---------NGIAPGGQLTTTTDVENYPGFPD---------------
TREME07701      NLEPV-LYEGMLA---------NGFAPGGQLTTTTDVENFPGFPD---------------
AURST04751      NLAPV-MFEGFMA---------NGFAAGGQLTTTTDVENFPGFPD---------------
FOMME10177      NLTPV-LFEGFLA---------NGFAAGGQLTTTTEVENFPGFPE---------------
CONPW06392      NLNPV-LFEGFLA---------NGFAAGGQLTTTTDVENFPGFPS---------------
STEHR07076      NLAPV-MYEGFMA---------NGFAAGGQLTTTTDVENFPGFPT---------------
HETAN06295      NLSPV-LFEGFMA---------NGFAAGGQLTTTTDVENFPGFPT---------------
GLOTR06982      NLDPV-LFEGFMA---------NGFAAGGQLTTTTDVENFPGFPQ---------------
PUNST01981      NLNPV-LFEGFMA---------NGFAAGGQLTTTTDVENFPGFPS---------------
LACBI02877      NLDPV-MFEGFMA---------NGFAAGGQLTTTTDVENFPGFPT---------------
COPCI16429      NLQPV-MFEGFMA---------NGFAAGGQLTTTTDVENFPGFPS---------------
DICSQ11618      NLQPV-LFEGFMA---------NGFAAGGQLTTTTDVENFPGFPS---------------
TRAVS13180      NLNPV-LFEGFMA---------NGFAAGGQLTTTTDVENFPGFPS---------------
WOLCO03584      NLNPV-LFEGFMA---------NGFAAGGQLTTTTDIENFPGFPS---------------
FOMPI05979      NLNPV-LFEGFMA---------NGFAAGGQLTTTTDIENFPGFPS---------------
PHLGI10219      NLNPV-LFEGFMA---------NGFAAGGQLTTTTDIENFPGFPS---------------
PHACH05757      NLNPV-LFEGFMA---------NGFAAGGQLTTTTDIENFPGFPS---------------
RICTY00419      SLKPI-LINGM----------Q----PGGQLTMTTDVENYPGFAK---------------
```

```
RICPR00429      ALKPI-LINGM----------Q----PGGQLTMTTDVENYPGFAE---------------
RICPP00461      ALKPI-LINGM----------Q----PGGQLTMTTDVENYPGFAE---------------
RICBR00434      SLNPI-LINGI----------Q----PGGQLTITTDVENYPGFAE---------------
RICB800988      SLNPI-LINGI----------Q----PGGQLTITTDVENYPGFAE---------------
RICCK00600      ALKPI-LINGM----------Q----PGGQLTITTDVENYPGFAE---------------
RICAH00614      ALKPI-LINGM----------R----PGGQLTITTNVENYPGFVE---------------
RICAC00782      ALKPI-LINGM----------Q----PGGQLTITTDVENYPGFAE---------------
RICFE00656      ALKPI-LINGM----------Q----PGGQLTITTDVENYPGFAE---------------
RICMS00042      ALKPI-LINGM----------Q----PGGQLTITTDVENYPGFAE---------------
RICM500453      ALKPI-LISGM----------Q----PGGQLTITTDVENYPGFAE---------------
RICR300665      ALKPI-LINGM----------Q----PGGQLTITTDVENYPGFAE---------------
RICAG00651      ALKPI-LINGM----------Q----PGGQLTITTDVENYPGFAE---------------
RICP300630      ALKPI-LINGM----------Q----PGGQLTITTGVENYPGFAE---------------
RICRS00632      ALKPI-LINGM----------Q----PGGQLTITTGVENYPGFAE---------------
RICRO00663      ALKPI-LINGM----------Q----PGGQLTITTGVENYPGFAE---------------
RICCN00618      ALKPI-LINGM----------Q----PGGQLTITTDVENYPGFAE---------------
RICPT00620      ALKPI-LINGM----------Q----PGGQLTITTDVENYPGFAE---------------
RICAE00497      ALKPI-LINGM----------Q----PGGQLTSTTDVENYPGFAE---------------
RICJY00467      ALKPI-LINGM----------Q----PGGQLTITTDVENYPGFAE---------------
RICPU00072      ALKPI-LINGM----------Q----PGGQLTITTGVENYPGFAE---------------
RICS100537      ALKPI-LINGM----------Q----PGGQLTITTDVENYPGFAE---------------
BARBK00891      MLKPV-LITGL----------Q----QGGQLTITTDIENYPGFAE---------------
BARVW00932      MLKPV-LVTGV----------Q----QGGQLTITIDVENYPGFAD---------------
BART100617      MLQPV-LVTGL----------Q----QGGQLTITTDVENYPGFSD---------------
BARGA00466      MLKPV-LVTGL----------Q----QGGQLTITTDVENYPGFAD---------------
BARHE01107      MLKPV-LITGL----------Q----QGGQLMITTDVENYPGFAD---------------
BARQU00895      MLEPV-LVTGL----------Q----QGGQLTITTDVENYPGFAD---------------
OCHA401642      MLKPL-IIAGL----------Q----QGGQLMITTDVENYPGYAD---------------
BRUAB01390      MLKPV-VIAGL----------Q----QGGQLMITTDVENYPGYAE---------------
BRUA201507      MLKPV-VIAGL----------Q----QGGQLMITTDVENYPGYAE---------------
BRUA101339      MLKPV-VIAGL----------Q----QGGQLMITTDVENYPGYAE---------------
BRUSU01458      MLKPV-VIAGL----------Q----QGGQLMITTDVENYPGYAE---------------
BRUME00510      MLKPV-VIAGL----------Q----QGGQLMITTDVENYPGYAE---------------
BRUSI01461      MLKPV-VIAGL----------Q----QGGQLMITTDVENYPGYAE---------------
BRUC201446      MLKPV-VIAGL----------Q----QGGQLMITTDVENYPGYAE---------------
BRUMC01440      MLKPV-VIAGL----------Q----QGGQLMITTDVENYPGYAE---------------
BRUMB01421      MLKPV-VIAGL----------Q----QGGQLMITTDVENYPGYAE---------------
BRUM501487      MLKPV-VIAGL----------Q----QGGQLMITTDVENYPGYAE---------------
BRUO201285      MLKPV-VIAGL----------Q----QGGQLMITTDVENYPGYAE---------------
RHILO01973      MLKPM-LVAGL----------Q----QGGQLMITTDVENYPGFAD---------------
CHESB02097      MLKPL-LVSGL----------E----QGGQLMITTEVENYPGFAE---------------
METPB00996      MVEPL-LISGF----------Q----PGGQLMITTDVENYPGFAE---------------
METEP01063      MVEPL-LISGF----------Q----PGGQLMITTDVENYPGFAD---------------
METEA00810      MVEPL-LISGF----------Q----PGGQLMITTDVENYPGFAD---------------
METED01453      LVEPL-LISGF----------Q----PGGQLMITTDVENYPGFAD---------------
METS403554      MVEPL-LISGF----------Q----PGGQLMITTDVENYPGFAQ---------------
METNO05482      MVEPL-LISGF----------Q----PGGQLMITTDVENYPGFAQ---------------
METSZ03234      LLEPM-LIAGF----------D----QGGQLMITTEVENYPGFAE---------------
BEII900056      MLEPV-LISGI----------E----TGGQLMITTDVENYPGFAE---------------
METSB02743      MLEPV-VIAGF----------E----AGGQLMITTDVENYPGFAS---------------
MAGMM00401      NLSPV-VIQGL----------Q----PGGQLTITTDVDNYPGFAD---------------
HYPNA00542      LRDTL-VVTGM----------Q----PGGQLTITTEVENWPGES----------------
KETVY00902      MLQPI-LVQGM----------E----PGGQLTTTTDVENWPGEK----------------
KETVW00472      MLQPI-LVQGM----------E----PGGQLTTTTDVENWPGEK----------------
ROSDO03242      MLEPI-LVQGI----------E----PGGQLTTTTEVENWPGDS----------------
ROSLO02615      MLEPI-LVQGI----------E----PGGQLTTTTEVENWPGDS----------------
RUEPO00888      MLEPI-LVQGI----------E----PGGQLTTTTEVENWPGDT----------------
RUEST00613      MLEPI-LVQGI----------E----PGGQLTTTTEVENWPGHT----------------
PHAIB02390      MLEPI-LVQGI----------E----PGGQLTTTTEVENWPGDT----------------
PARDP02130      MLSPV-LIQGM----------Q----PGGQLTITTEVENWPGET----------------
DINSH02620      MLNPV-LVQGI----------Q----PGGQLTITTDVENWPGDS----------------
RHOCB02768      MLKPI-LVQGM----------Q----PGGQLTITTEVENWPGRT----------------
RHOS500248      MLNPL-LIQGL----------Q----PGGQLTITTEVENWPGDR----------------
RHOS400150      MLNPL-LIQGL----------Q----PGGQLTITTEVENWPGDR----------------
RHOS100227      MLNPL-LIQGL----------Q----PGGQLTITTEVENWPGDR----------------
```

```
RHOSK02952      MLNPL-LIQGL----------Q----PGGQLTITTEVENWPGDR----------------
MIDMI00790      ALKPI-LIAGQ----------Q----PGGQLTITTDVENYPGFAE---------------
ACEP301595      NLSPV-LVAGL----------Q----PGGQLTITTEVENYPGFAT---------------
MICAA01566      NLNPI-QILGL----------E----PGGQLTITTDVENFPGFAD---------------
TISMK03676      MLEPI-LVHGL----------Q----PGGQMTITTDVENFPGFPD---------------
AZOL402409      NLEPL-MVQGM----------Q----PGGQLMITTDVENFPGFAD---------------
PSEUV04348      MLEPL-LVTGV----------Q----TGGQLTITTDVENYPGFAD---------------
HIRBI01095      MRKPM-LIAGM----------Q----PGGQLTITTEVENYPGFAE---------------
PARL102241      MLEPV-LIQGI----------Q----PGGQLTITTDVENYPGFAE---------------
MARMM02139      MLEPT-LIAGL----------Q----PGGQLTITTDVENYPGFAD---------------
PHEZH02588      LLKPV-LIQGI----------Q----PGGQLTITTDVENYPGFAD---------------
CAUCR02826      LLKPV-LIAGI----------Q----PGGQLTITTDVENYPGFAD---------------
CAUCN02939      LLKPV-LIAGI----------Q----PGGQLTITTDVENYPGFAD---------------
CAUST00891      LLKPV-LIAGI----------Q----PGGQLTITTDVENYPGFAD---------------
PARBH01522      MLEPV-IVHGL----------Q----PGGQLTITTDVENYPGFAD---------------
PELHB02084      MLEPL-MIAGM----------Q----PGGQLTITTDVENYPGFAD---------------
HYPDA03273      MLAPT-LIQGS----------Q----PGGQLTITTDVENYPGFAE---------------
HYPSM04642      MLSPT-LIQGT----------Q----PGGQLTITTDVENYPGFAD---------------
OLICO01107      MLEPV-LIQGI----------Q----PGGQLTITTDVENFPGFAE---------------
OLICM02790      MLEPV-LIQGI----------Q----PGGQLTITTDVENFPGFAE---------------
RHOPS01446      MLEPI-LIQGI----------Q----PGGQLTITTDVENYPGFAD---------------
RHOPA04062      MLEPI-LIQGM----------Q----PGGQLTITTDVENYPGFAD---------------
RHOPT04533      MLEPI-LIQGM----------Q----PGGQLTITTDVENYPGFAD---------------
RHOPX04235      MLEPI-LIQGM----------Q----PGGQLTITTDVENYPGFAD---------------
BRADU07321      MLEPI-LIQGI----------Q----PGGQLTITTDVENYPGFAD---------------
BRASO01239      MLEPV-LIQGI----------Q----PGGQLTITTDVENYPGFAD---------------
BRASB06352      MLEPV-LIQGI----------Q----PGGQLTITTDVENYPGFAD---------------
RHOPB03773      MLEPI-LLQGM----------Q----PGGQLTITTDVENYPGFAD---------------
NITWN02324      MLEPV-LIQGI----------Q----PGGQLTITTDVENYPGFAD---------------
NITHX02707      MLEPV-LIQGI----------Q----PGGQLTITTDVENYPGFAD---------------
AZOC501143      MLEPI-LFEGI----------Q----PGGQLTITTDVENYPGFAD---------------
XANP202679      MLEPV-LFEGI----------Q----PGGQLTITTDVENYPGFAD---------------
CHLTF01983      NLKPL-LFEGE----------GPENIPGGQLMITTDVDNYPGFQH---------------
IGNAJ02236      NLNPV-VFEGL----------Q----PGGQLTITTEVENFPGFEH---------------
MELRP00403      NLEPI-VFEGM----------Q----PGGQLTITTEVENYPGFEN---------------
ANADF00468      NLNPL-MFEGM----------Q----PGGQLTITSEVENFPGFPE---------------
CHLCH01125      NIRPL-VIEGI----------Q----PGGQLMITSEIENFPGFPE---------------
PELPB01419      NLKPL-VIEGF----------Q----PGGQLMITTEIENFPGFPE---------------
CHLL200781      NLKPL-VIEGV----------Q----PGGQLMITTEIENFPGFPD---------------
CHLTE00830      NLKPL-VIEGP----------Q----PGGQLMITTDIENFPGFPE---------------
CHLP800854      NLKPL-VIEGH----------Q----PGGQLMITTDIENFPGFPE---------------
CHLL701225      NLKPL-VIDGY----------Q----PGGQLMITSEIENFPGFPE---------------
CHLPM00993      NLNPL-VIDGY----------Q----PGGQLMITSEIENFPGFPE---------------
WOLTR00562      NLEPV-VVTGM----------Q----PGGQLTITMGVENYPGFV----------------
WOLPP00135      NLEPI-VVTGM----------Q----PGGQLTITTDVENYPGFI----------------
WOLPM00650      NLEPI-VVTGM----------Q----PGGQLTITTDVENYPGFV----------------
WOLWR00416      NLEPI-VVTGM----------Q----PGGQLTITTDVENYPGFV----------------
EHRCR00696      NLKPI-LITGM----------C----PGGQLTITTDVENFPGFAH---------------
ANAMM00345      NLEPL-LVTGM----------Q----PGGQLTITTDVENYPGFS----------------
ANAMF00341      NLEPL-LVTGM----------Q----PGGQLTITTDVENYPGFS----------------
ANAPZ00610      NLNPV-LVTGM----------Q----PGGQLTITTDVENYPGFS----------------
NEOSM00537      SLSVV-IVSGN----------Q----SGGQLTTTTEVENYPGFAL---------------
NEORI00513      SLSVI-IVSGN----------Q----SGGQLSTTTEVENYPGFAL---------------
PELUB00076      MLNPI-LVYGV----------Q----PGGQLTTTTDVENYPGFSD---------------
PELSM00736      MLKPT-LISGM----------E----PGGQLTTTTDVENYPGFSD---------------
PUNMI01409      NLSPV-IIAGL----------Q----PGGQMTITTDVENYPGFAD---------------
ASTEC00651      LLHPV-VFTGP----------Q----TGGQLTITTDVENYPGFAE---------------
ZYMMT00154      GLSPI-VAQGL----------Q----PGGQLTITTDVENFPGFRE---------------
ZYMMO00984      GLNPI-VAQGL----------Q----PGGQLTITTEVENFPGFRE---------------
ZYMMA00176      GLNPI-VAQGL----------Q----PGGQLTITTEVENFPGFRE---------------
ZYMMN00183      GLNPI-VAQGL----------Q----PGGQLTITTEVENFPGFRE---------------
SPHAL00143      GMQPI-VVQGL----------Q----PGGQLTITTDVENYPGFAE---------------
SPHWW03978      GLAPI-VVQGL----------Q----PGGQLTITTDVENYPGFRD---------------
SPHJU02313      GLAPI-VVQGM----------Q----PGGQLTITTDVENYPGFRD---------------
NOVAD02319      GMEPI-VVQGL----------Q----PGGQLTITTDVENYPGFRD---------------
```

```
ERYLH00934      MMEPI-VVQGI----------Q----PGGQLTITTDVENYPGFEE---------------
GRABC00720      SMKPI-LVCGL----------Q----PGGQLTITTDVENYPGFAE---------------
GLUDA03075      NLKPI-LVAGL----------Q----PGGQLMITTDVYNYPGFAR---------------
KOMMN00774      NLSPV-LVAGL----------Q----PGGQLMITTDVENYPGFGK---------------
HALVD01097      NNEPL-VLEGD----------E----PGGQLTLTTEVDNYPGFPE---------------
HALHT01699      NNDPL-VLEGD----------E----PGGQLTLTSEVENFPGFPE---------------
METI401038      KLSPL-LVTG----------TE----PGGLLTTTTIVENYPGFPM---------------
PLAL201612      SLEPL-VFEGAISEENRIRGTL----PLGQLALTTEVENYAGFPAANLTQFLETALPKER
GEMAT01983      NLNPL-VFEGEPVGT-----EL----PGGQLMLTTDIENFPGFPE---------------
CYAAP02456      QLNPL-MFEGFSVG------GV----PGGQLMTTTEVENYPGFAQ---------------
CHLPN00303      LLHPL-LFEGFFS-------GI----SGGQLMTTTEVENFPGFPE---------------
CHLPP00439      LLHPL-LFEGFFS-------GI----SGGQLMTTTEVENFPGFPE---------------
CHLPE00727      LLQPR-LFEGFFS-------GT----AGGQLMTTTEVENFPGFPE---------------
CHLTR00101      LLTPV-LFEGFFS-------GI----AGGQLMTTTEVENFPGFPE---------------
CHLTA00102      LLTPV-LFEGFFS-------GI----AGGQLMTTTEVENFPGFPE---------------
CHLTJ00101      LLTPV-LFEGFFS-------GI----AGGQLMTTTEVENFPGFPE---------------
CHLTD00098      LLTPV-LFEGFFS-------GI----AGGQLMTTTEVENFPGFPE---------------
CHLT700102      LLTPV-LFEGFFS-------GI----AGGQLMTTTEVENFPGFPE---------------
CHLT000101      LLTPV-LFEGFFS-------GI----AGGQLMTTTEVENFPGFPE---------------
CHLT500104      LLTPV-LFEGFFS-------GI----AGGQLMTTTEVENFPGFPE---------------
CHLTL00098      LLTPV-LFEGFFS-------GI----AGGQLMTTTEVENFPGFPE---------------
CHLTG00102      LLTPV-LFEGFFS-------GI----AGGQLMTTTEVENFPGFPE---------------
CHLTS00101      LLTPV-LFEGFFS-------GI----AGGQLMTTTEVENFPGFPE---------------
CHLT900102      LLTPV-LFEGFFS-------GI----AGGQLMTTTEVENFPGFPE---------------
CHLTZ00101      LLTPV-LFEGFFS-------GI----AGGQLMTTTEVENFPGFPE---------------
CHLT400432      LLTPV-LFEGFFS-------GI----AGGQLMTTTEVENFPGFPE---------------
CHLT100103      LLTPV-LFEGFFS-------GI----AGGQLMTTTEVENFPGFPE---------------
CHLT200350      LLTPV-LFEGFFS-------GI----AGGQLMTTTEVENFPGFPE---------------
CHLTB00350      LLTPV-LFEGFFS-------GI----AGGQLMTTTEVENFPGFPE---------------
CHLTC00378      LLTPV-LFEGFFS-------GI----AGGQLMTTTEVENFPGFPE---------------
SIMNZ00831      ELSPL-LFEGFFS-------GP----AGGQLMTTTEVENFPGFPE---------------
PARUW01706      NLEPI-LFEGFFT-------GA----AGGQLMTTTEVENFPGFPD---------------
WADCW00943      NLEPV-LYEGFQT-------GP----AGGQLMITTDVENFPGFPE---------------
SINAD04980      NLSPL-VLEGH----------E----PGGQLTWTTVVENFPGFPE---------------
BIFLB01558      GLKPV-VVAGAL---------T----PGGQLINTTEVENYPGFPD---------------
BIFAB00498      GLKPV-VVAGAL---------T----PGGQLINTTEVENYPGFPD---------------
BIFAV01561      GLKPV-VVAGAL---------T----PGGQLINTTEVENYPGFPD---------------
BIFAS01557      GLKPV-VVAGAL---------T----PGGQLINTTEVENYPGFPD---------------
BIFA001518      GLKPV-VVAGAL---------T----PGGQLINTTEVENYPGFPD---------------
BIFBA01814      GLNPV-LVTGAL---------A----PGGQLVNTTEVENFPGFPD---------------
BIFAA01619      GFEPL-VVAGAL---------T----PGGQLVNTTEVENFPGFPD---------------
BIFDB02112      GYRPL-VIAGAL---------T----PGGQLVNTTEVENFPGFPD---------------
TERSS03091      NLKPL-VLEGH----------E----PGGQLSITTLVENFPGWPE---------------
GRATM03160      NLKPL-VLEGH----------E----PGGQLSITTLVENFPGWPE---------------
GRAMM03836      NLKPL-VLEGH----------E----PGGQLSITTLVENFPGWPD---------------
AKKM801247      NLSPL-LITGS----------Q----IGGQLTTTTEVENFPGFPD---------------
OPITP04130      NLNPL-VLEGT----------L----PGGQLTTTSEVENFPGFPE---------------
CORAD01794      NLSPL-VLEGT----------Q----PGGQLTTTSEVENFPGFPE---------------
BUCCC00180      NIKTI-LITGP----------Q----PGGQLIQTNEIENWPGNYN---------------
CENSY00347      RRDTL-ILSGI----------L----PGGQLVNTTDVENFPGFEN---------------
NITMS00668      GYDTL-ILSGI----------L----PGGQLVNTTEVENYPGFEN---------------
MEIRD02823      NLKTL-ILEKG----------L----PGGQIAQTEEVENYPGFPE---------------
HERA203780      NLTPL-VIRGI----------Q----PGGLIATTSEVENYPGFPD---------------
CHLAA02018      NLEPL-VIRGL----------Q----PGGLIATTSEVENYPGFVD---------------
CHLSY02175      NLEPL-VIRGL----------Q----PGGLIATTSEVENYPGFVD---------------
CHLAD02599      NLEPL-VIRGL----------Q----PGGLIATTSEVENYPGFPE---------------
CALAS01745      NLSPL-LITGN----------E----YGGQVSLTYDIENYPGFPE---------------
ANATU01696      ELNPL-VLTGM----------E----LGGQAALTHTIENYPGFPE---------------
SULMS00235      DLNHI-LYTGP----------I----PGGQLIKTNEIENYPGFPK---------------
BLASB00041      DLNPI-LFVGF----------Q----PGGQLTTTTNIDNYLGFPA---------------
BLASP00551      DLHPI-LFVGM----------Q----PGGQLTSTTKVDNYPGFPV---------------
AZOPC00437      SLSPI-LYEGT----------Q----SGGQLTTTFEIENFPGYPD---------------
LEPBD01878      GLKNI-IINGM----------E----PGGQLTTTTEVENFPGFPQ---------------
SALRD02577      DLSPL-IFMGP----------E----PGGQLTTTTDVENYPGFPE---------------
SALRM02866      DLSPL-IFMGP----------E----PGGQLTTTTDVENYPGFPE---------------
```

```
RIEPU00166      GLRPI-LITGF----------Q----KGGQIALANSVENWTGLYP---------------
ORITB00015      HSNPV-LLHGN----------Q----PGGQLMTTLEVENYPGFKN---------------
ORITI01234      HSNPV-LLHGN----------Q----PGGQLMTTLEVENYPGFTN---------------
PREMB01541      NLQPV-LYSGL----------Q----PGGQLTTTTIVENFPGFHE---------------
PREDF01155      NLRPV-LYSGM----------Q----PGGQLTTTTLVENFPGFHE---------------
PREI702011      NLQPV-LYAGL----------Q----PGGQLTTTTIIENFPGFKD---------------
ALIFI01035      NLRPV-LYEGI----------E----PGGQLTTTTDVENFPGYPD---------------
ODOSD00362      NLKPV-LYTGL----------Q----MGGQLTTTTEVENFPGYPE---------------
PRER201250      NLSPI-EYSGM----------Q----PGGQLTQTTEVENFPGYPQ---------------
PALPW00206      NLSPV-LYEGM----------Q----PGGQLTTTTEVENFPGYPS---------------
PORGI00953      NLNPI-LYEGI----------Q----PGGQLTTTTEVENFPGYPE---------------
PORG301168      NLNPI-LYEGI----------Q----PGGQLTTTTEVENFPGYPE---------------
BACV803426      NIAPV-LYEGL----------Q----PGGQLTITTEVENFPGYPE---------------
BACT601486      NLSPV-LYAGL----------Q----PGGQLTTTTDVENFPGYPV---------------
BACTN04290      NLCPV-LYEGL----------Q----PGGQLTTTTDVENFPGYPE---------------
BACFR01032      NLSPV-LYEGI----------Q----PGGQLTTTTDVENFPGYPQ---------------
BACFN00913      NLSPV-LYEGI----------Q----PGGQLTTTTDVENFPGYPQ---------------
BACF600963      NLSPV-LYEGI----------Q----PGGQLTTTTDVENFPGYPQ---------------
OWEHD03413      NLKPV-VYTGL----------E----PGGQLTTTTDVENFPGYPE---------------
PSYTT00390      DMNPV-VYTGI----------E----PGGQLTTTTEVDNFPGYPQ---------------
NONDD00011      DMKPV-VYTGM----------E----PGGQLTTTTEVDNFPGYPD---------------
ROBBH02711      DLKPV-VYTGM----------E----PGGQLTTTTEVENFPGYPD---------------
CELAD02592      DLKPV-VYTGM----------E----PGGQLTTTTEVDNFPGYPE---------------
CELLC02474      DLKPV-MYTGM----------E----PGGQLTTTTEVDNFPGYPE---------------
MARSH03072      DLKPL-LYTGM----------E----PGGQLTTTTEVDNFPGYPE---------------
MURRD00159      DLKPV-MYTGM----------E----PGGQLTTTTEVDNFPGYPE---------------
ZOBGA00152      DLKPV-VYTGM----------E----PGGQLTTTTEVDNFPGYPE---------------
GRAFK00949      DMKPL-MYTGM----------E----PGGQLTTTTEVDNFPGYPQ---------------
ZUNPS02344      DLKPI-MYTGM----------E----PGGQLTTTTEVDNFPGYPE---------------
AEQSU02079      DLKPI-MYTGM----------E----PGGQLTTTTEVDNFPGYPQ---------------
HALH105745      NMNPV-LFTGK----------D----PGGQLMITNDVENYPGYPS---------------
SAPGL02893      NMEPV-LYTGK----------E----PGGQLMITTDVENFPGYPK---------------
AMOA500014      GLDVV-LYQGL----------Q----PGGQLTTTTEVENFPGYPE---------------
FLAIG01802      NMNPV-LYQGQ----------Q----PGGQLTTTNEVENFPGYPE---------------
FLACA02326      NMSPV-LYQGT----------Q----PGGQLTTTNEVENFPGYPE---------------
FLAJ100198      NMNPV-LYQGM----------Q----PGGQLTTTNEVENFPGYVD---------------
FLABF01528      NLNPV-LYQGM----------Q----PGGQLTTTNEVENFPGYVD---------------
CAPOD01333      NLSPV-LYQGE----------Q----PGGQLTTTNEVENFPGYPE---------------
CAPCC00414      NMQPV-LYQGE----------Q----PGGQLTTTNEVENFPGYPN---------------
FLELS01898      GLEPI-LYKGQ----------Q----PGGQLTITNDVENFPGYAD---------------
SOLCM01166      DLKPV-MYTGL----------Q----PGGQLTTTTEVENFPGYPE---------------
PEDHD01598      DLKPV-MYTGM----------E----PGGQLTQTTDVDNFPGYPN---------------
SPHS203207      DLKPV-MYTGI----------V----PGGQLTQTTDVENFPGYPA---------------
LEAB401188      GLKPV-MYQGM----------Q----PGGQLTITNDVENFPGYPE---------------
EMTOG00092      GLKPM-MYQGG----------Q----PGGQLTITTEVENFPGYPE---------------
SPILD01737      NVQPV-LYQGG----------Q----PGGQLTITTEVDNFPGYPN---------------
DYAFD00084      GLNPV-LYQGA----------Q----PGGQLTITTEVDNYPGYPD---------------
CYCMS03063      GLSPV-MYTGV----------Q----PGGQLTITNDVENYPGYPE---------------
ECHVK03326      GLSPV-LYTGG----------Q----PGGQLTITNDVENYPGYPD---------------
BELBD02542      GLNPI-LYTGG----------Q----PGGQLTITTDVENYPGYPN---------------
NITGG02112      KLSTL-IISGT----------L----PGGQLMTTSEVENYPGFPN---------------
THEM700111      NLDTT-VVAGW----------E----AGGQLMLTTEVENFPGFPD---------------
CREAS01350      LLNPL-LIAGY----------K----PGGQLMLTTLVENFPGFPD---------------
TURPD02772      NLKTL-VLEGI----------E----RGGQLMITSEVENFPGFAQ---------------
SORC507603      NLKPL-LIEGFS---A--GGLI----PGGQLMFTTDVENYPGFPE---------------
BDEBA00337      NLEPL-MIEGE----------E----AGGQLMTTTEVENFPGFDH---------------
STIAD02752      NLEPV-VFAGGPTMED--PQRV----PGGQLMVTTDVENYPGFPE---------------
MYXXD01876      NLQPV-VFAGGPTLEH--PQRV----PGGQLMVTTDVENYPGFPE---------------
MYXFH03454      NLQPV-VFAGGPTLEH--PQRV----PGGQLMVTTDVENYPGFPE---------------
CORCM01960      NLEPV-VFAGGPTLEH--PQRV----PGGQLMVTTDVENYPGFPE---------------
MYXSD02209      NLEPV-VFAGGPTLEH--PQRV----PGGQLMVTTDVENYPGFPE---------------
LEPFC02126      GLSPV-VLEGP----------Q----HGGQLTTTTEVDNFPGFPD---------------
LEPFM02294      FLSPL-LIEGP----------Q----SGGQLTTTTDVDNFPGFPK---------------
SOLUE00582      NLKPL-VIAGH----------E----AGGQLSLTTLVENFPGFPE---------------
KORVE01333      NLKPL-LIQGH----------E----PGGQLSMTTLVENFPGWPE---------------
```

```
ACIC502553      NLNPL-VLEGH----------E----PGGQLSITTLVENFPGWPE---------------
ACIFD00030      SLEPV-VIEGEPSST----SDQ----PGGQLMLTTEIENFPGFPE---------------
HALMS01402      NLNPL-VIEGH----------E----PGGQLTTTTDVDNFPGFPE---------------
CHLPD01133      NLKPL-VIEGS----------Q----PGGQLMITSEIENFPGFPE---------------
CHLPB01137      NLNPL-VIDGC----------Q----PGGQLMITSEIENYPGFPE---------------
PROA200897      NLKPL-VIDGV----------Q----PGGQLMITSEIENFPGFPS---------------
WIGBR00492      GINPI-LITGS----------Q----LGGQLTTTDKIENWPGDSK---------------
BUCA500289      NLDPF-LITGT----------N----KGGQLMNTNEIENWPGDYN---------------
BUCAI00292      NLDPF-LITGT----------N----KGGQLMNTNEIENWPGDYN---------------
BUCAF00306      NLDPF-LITGT----------N----KGGQLMNTNEIENWPGDYN---------------
BUCAT00287      NLDPF-LITGT----------N----KGGQLMNTNEIENWPGDYN---------------
BUCA000302      NLDPF-LITGT----------N----KGGQLMNTNEIENWPGDYN---------------
BAUCH00290      NLNPV-LITGI----------E----KGGQLVTTTEVENWPGDSK---------------
BLOVB00369      NLSPI-LISGL----------E----IGGQLSTTLSIENWPGDSE---------------
BLOFL00367      NLNPT-LITGL----------E----HGGQLNTTTSIENWPGDPE---------------
BLOPB00375      NLNPV-LVTGL----------E----IGGQLNTTTDIENWPGDPK---------------
BUCAP00289      NLNPL-LITGI----------N----KGGQLMNTNEIENWPGDFK---------------
COXBU01001      NLKPI-MITGM----------E----QGGQLMTTTDVDNWPGEAP---------------
COXBN01188      NLKPI-MITGM----------E----QGGQLMTTTDVDNWPGEAP---------------
COXBR01117      NLKPI-MITGM----------E----QGGQLMTTTDVDNWPGEAP---------------
COXB200717      NLKPI-MITGM----------E----QGGQLMTTTDVDNWPGEAP---------------
COXB100914      NLKPI-MITGM----------E----QGGQLMTTTDVDNWPGEAP---------------
FRAP200241      NLNPV-IITGM----------Q----PGGQLTTTTDVDNWPGEAD---------------
FRANT01012      NLNPV-IITGM----------Q----PGGQLTTTTDVDNWPGEAD---------------
FRACN00522      NLKPV-IITGM----------Q----PGGQLTTTTDVDNWPGEPD---------------
FRATT00465      NLKPV-IITGM----------Q----PGGQLTTTTDVYNWPGEPD---------------
FRAT100465      NLKPV-IITGM----------Q----PGGQLTTTTDVYNWPGEPD---------------
FRATE00462      NLKPV-IITGM----------Q----PGGQLTTTTDVYNWPGEPD---------------
FRATW01216      NLKPV-IITGM----------Q----PGGQLTTTTDVDNWPGEPD---------------
FRATM01110      NLKPV-IITGM----------Q----PGGQLTTTTDVDNWPGEPD---------------
FRACF00566      NLKPV-IITGM----------Q----PGGQLTTTTDVDNWPGEPD---------------
FRATO01212      NLKPV-IITGM----------Q----PGGQLTTTTDVDNWPGEPD---------------
FRATH01486      NLKPV-IITGM----------Q----PGGQLTTTTDVDNWPGEPD---------------
FRATF01216      NLKPV-IITGM----------Q----PGGQLTTTTDVDNWPGEPD---------------
FRATN00564      NLKPV-IITGM----------Q----PGGQLTTTTDVDNWPGEPD---------------
ACIF500512      NLSPI-LVQGM----------E----PGGQLMTTTEVDNWPGAAD---------------
ACIF200362      NLSPI-LVQGM----------E----PGGQLMTTTEVDNWPGAAD---------------
DECAR01284      NLKPV-LITGM----------A----QGGQLMTTTEVDNWPAAAD---------------
NEIG100525      NLNPV-IITGI----------A----QGGQLMTTTEVDNWPADAD---------------
NEIG201314      NLNPV-IITGI----------A----QGGQLMTTTEVDNWPADAD---------------
NEIM800926      NLNPV-IITGI----------A----QGGQLMTTTEVDNWPADAD---------------
NEIMP01199      NLNPV-IITGI----------A----QGGQLMTTTEVDNWPADAD---------------
NEIMB01212      NLNPV-IITGI----------A----QGGQLMTTTEVDNWPADAD---------------
NEIMF01143      NLNPV-IITGI----------A----QGGQLMTTTEVDNWPADAD---------------
NEIML01119      NLNPV-IITGI----------A----QGGQLMTTTEVDNWPADAD---------------
NEIMM00780      NLNPV-IITGI----------A----QGGQLMTTTEVDNWPADAD---------------
NEIMH00827      NLNPV-IITGI----------A----QGGQLMTTTEVDNWPADAD---------------
NEIMG01168      NLNPV-IITGI----------A----QGGQLMTTTEVDNWPADAD---------------
NEIMN01254      NLNPV-IITGI----------A----QGGQLMTTTEVDNWPADAD---------------
NEIMO00810      NLNPV-IITGI----------A----QGGQLMTTTEVDNWPADAD---------------
NEIM701229      NLNPV-IITGI----------A----QGGQLMTTTEVDNWPADAD---------------
NEIMA01334      NLNPV-IITGI----------A----QGGQLMTTTEVDNWPADAD---------------
NEIMW01125      NLNPV-IITGI----------A----QGGQLMTTTEVDNWPADAD---------------
DICNV01037      GLEPA-LITGL----------E----PGGQLMTTTHVDNWPSAFE---------------
VESOH00047      NLTPV-VVSGM----------E----QGGQLMNTTDVDNWPGDDA---------------
RUTMC00043      NLNPI-IVSGM----------E----QGGQLMSTMGVDNWPGDVA---------------
HALHL02255      NLEPV-LVTGM----------E----MGGQLTTTTEVDNWPGEPE---------------
PELPD03177      NIVPT-LITGL----------Q----PGGQLTTTTEVDNWPGDYQ---------------
GEOS804030      NLNPV-LITGL----------Q----QGGQLMTTTEVDNWPGDPE---------------
GEOBB03636      NLNPV-LITGL----------Q----PGGQLMTTTEVDNWPGDPE---------------
GEOSM03700      NLNPV-LIAGL----------Q----PGGQLMTTTEVDNWPGDPE---------------
HALNC00046      NLNPV-LITGM----------E----MGGQLTTTTEVENWPGDPG---------------
HAEPS00887      NLNPV-LVTGM----------Q----QGGQLTTTTEIENWPGEFG---------------
GALAU02285      NLKPV-LVTGL----------Q----QGGQLTTTNEIENWPGDAG---------------
HISS201000      NLKPV-LVTGL----------Q----QGGQLTTTTEIENWPGDFG---------------
```

HAES101151	NLKPV-LVTGL----------Q----QGGQLTTTTEIENWPGDFG---------------
PASMU00573	NLKPV-LVTGL----------Q----QGGQLTTTTEIENWPGDFG---------------
PASMH00579	NLKPV-LVTGL----------Q----QGGQLTTTTEIENWPGDFG---------------
ACTSZ01560	NLKPV-LVTGL----------Q----QGGQLTTTTEIENWPGDFG---------------
AGGAN00985	NLNPV-LVTGL----------Q----QGGQLTTTTEIENWPGDFG---------------
NITHN03181	ALDPV-LVTGV----------E----QGGQLMTTTDVDNWPGDDQ---------------
NITOC00311	ALEPV-LITGV----------E----QGGQLMTTTDVDNWPGDDQ---------------
NITWC02498	ALEPV-LITGV----------E----QGGQLMTTTDVDNWPGDDQ---------------
METNJ00648	ALEPV-LITGI----------E----QGGQLTTTTDVDNWPGDAD---------------
METFJ01616	ALDPV-LITGI----------E----QGGQLTTTTDVDNWPGDAD---------------
ALKEH00244	NLNPV-LVTGL----------E----MGGQLTTTTDVDNWPGDAE---------------
MARMS03166	NLNPV-MITGM----------Q----MGGQLTTTTEVDNWPGDDQ---------------
MARM102688	NLSPV-MVTGM----------Q----MGGQLTTTTEVDNWPGDDQ---------------
THICR00763	NLEPV-MVTGM----------Q----QGGQLTTTTEVDNWPGDVE---------------
THICA00974	NLEPV-IVTGM----------Q----QGGQLTTTTEVDNWPGDPE---------------
THIV600851	NLNPV-LVTGL----------E----QGGQLMTTTEVDNWAGDPD---------------
THISH01973	NLNPV-LITGL----------E----QGGQLTTTTDVDNWPGDDA---------------
METAA00813	NLNPV-MITGM----------Q----QGGQLTTTTDVDNWPGDVE---------------
META200813	NLNPV-MITGM----------Q----QGGQLTTTTDVDNWPGDVE---------------
METMM02895	NLSPV-MITGM----------Q----QGGQLTTTTEVDNWPGDVD---------------
FRAAD00813	NLQPT-MITGL----------Q----QGGQLMTTTEVDNWPGDQN---------------
XYLFA01416	NLQPV-LITGL----------Q----QGGQLMTTTEVDNWPGDAH---------------
XYLFT00623	NLQPV-LITGL----------Q----QGGQLMTTTEVDNWPGDAH---------------
XYLF200667	NLQPV-LITGL----------Q----QGGQLMTTTEVDNWPGDAH---------------
XYLFG01622	NLQPV-LITGL----------Q----QGGQLMTTTEVDNWPGDAH---------------
XYLFM00690	NLQPV-LITGL----------Q----QGGQLMTTTEVDNWPGDAH---------------
PSEUP01680	NLKPV-VITGL----------Q----QGGQLMTTTEVDNWPGDAH---------------
STRM501972	NLNPV-VITGL----------Q----QGGQLMTTTEVDNWPGDAH---------------
PSEUU01524	NLKPV-VITGL----------Q----QGGQLMTTTEVDNWPGDAH---------------
XANAP01407	NLKPV-VITGL----------Q----QGGQLMTTTEVDNWPGDAH---------------
XANCP01918	NLKPV-VITGL----------Q----QGGQLMTTTEVDNWPGDPH---------------
XANC802154	NLKPV-VITGL----------Q----QGGQLMTTTEVDNWPGDPH---------------
XANCB02263	NLKPV-VITGL----------Q----QGGQLMTTTEVDNWPGDPH---------------
XANOR02379	NLKPV-VITGL----------Q----QGGQLMTTTEVDNWPGDPH---------------
XANOM02295	NLKPV-VITGL----------Q----QGGQLMTTTEVDNWPGDPH---------------
XANOP02318	NLKPV-VITGL----------Q----QGGQLMTTTEVDNWPGDPH---------------
XANAC01951	NLKPV-VITGL----------Q----QGGQLMTTTEVDNWPGDPH---------------
CYCSP01155	NLNPL-LITGI----------E----QGGQLMTTTEVDNWPGDVE---------------
GEOLS03275	NLNPV-LIAGL----------Q----PGGQLTTTTEVDNWPGDFE---------------
GEOUR03858	NLNPV-IITGM----------Q----QGGQLMTTTEVDNWPGDPD---------------
GEODF00692	NLNPV-IITGL----------Q----QGGQLTTTTEVDNWPGDFE---------------
GEOSL00482	NLSPA-LITGL----------Q----QGGQLMTTTEVDNWPGDPD---------------
GEOSK00469	NLSPA-LITGL----------Q----QGGQLMTTTEVDNWPGDPD---------------
GEOMG02998	NLNPT-LITGL----------Q----QGGQLMTTTEVDNWPGDPN---------------
MORCR00228	NLNPV-IITGL----------Q----VGGQLTTTTEVDNWPGDAH---------------
ACIAD00798	NLKPT-LIAGL----------Q----LGGQLTTTTEVDNWPGDPE---------------
ACIBC00821	NLKPT-LIAGL----------Q----LGGQLTTTTEVDNWPGDPE---------------
ACIBY02725	NLKPT-LIAGL----------Q----LGGQLTTTTEVDNWPGDPE---------------
ACIB302691	NLKPT-LIAGL----------Q----LGGQLTTTTEVDNWPGDPE---------------
ACIB500882	NLKPT-LIAGL----------Q----LGGQLTTTTEVDNWPGDPE---------------
ACIB100850	NLKPT-LIAGL----------Q----LGGQLTTTTEVDNWPGDPE---------------
ACIBD00843	NLKPT-LIAGL----------Q----LGGQLTTTTEVDNWPGDPE---------------
ACIBS02229	NLKPT-LIAGL----------Q----LGGQLTTTTEVDNWPGDPE---------------
ACICP00137	NLKPT-LIAGL----------Q----LGGQLTTTTEVDNWPGDPE---------------
ACISD03052	NLKPT-LIAGL----------Q----LGGQLTTTTEVDNWPGDPE---------------
LEGLN02402	NLNPV-LITGM----------Q----PGGQLTTTTEVDNWPGDIE---------------
LEGPA01726	NLNPV-LITGM----------Q----PGGQLTTTTDVDNWPGDIE---------------
LEGPH00846	NLNPV-LITGM----------Q----PGGQLTTTTDVDNWPGDIE---------------
LEGPC01171	NLNPV-LITGM----------Q----PGGQLTTTTDVDNWPGDIE---------------
LEGP201857	NLNPV-LITGM----------Q----PGGQLTTTTDVDNWPGDIE---------------
LEGPL01727	NLNPV-LITGM----------Q----PGGQLTTTTDVDNWPGDIE---------------
COLP302668	NLKPV-MITGM----------Q----QGGQLTTTTDVENWPGDAD---------------
KANKD01106	NLNPA-IITGI----------Q----QGGQLTTTTDVDNWPGDNE---------------
IDILO00659	NLNPV-MVTGM----------Q----QGGQLTTTTDVENWPGDAE---------------
PSEU901284	NLNPV-LITGI----------Q----QGGQLTTTTEVENWPGDAH---------------

```
PSEA602346      NLNPV-LLTGI----------Q----QGGQLTTTTEVENWPGDAE---------------
ALTSS01871      NLEPV-LLTGI----------Q----QGGQLTTTTEVENWPGDPE---------------
ALTMD01611      NLEPV-LLTGI----------Q----QGGQLTTTTEVENWPGDPE---------------
ALTME01705      NLKPV-LLTGI----------Q----QGGQLTTTTEVENWPGDPE---------------
ALTMB01797      NLKPV-LLTGI----------Q----QGGQLTTTTEVENWPGDPE---------------
ALTMS01652      NLKPV-LLTGI----------Q----QGGQLTTTAEVENWPGDPE---------------
SACD201683      NLKPV-VITGM----------Q----QGGQLTTTTEVENWPGGVA---------------
TERTT01748      NLKPV-VITGM----------Q----QGGQLTTTTEVENWPGGSH---------------
SIMAS00609      NLKPV-VITGM----------Q----QGGQLTTTTEVENWPGGVH---------------
ALCDB01962      NLNPV-VITGI----------Q----PGGQLTTTTEVDNWPGDVE---------------
CHRSD02939      NLKPL-LITGM----------Q----AGGQLTTTTDVDNWPGDAE---------------
HALED02811      NLKPL-LITGI----------Q----AGGQLTTTTDVDNWPGDDI---------------
SIDLE02655      NLKPT-LITGM----------A----QGGQLMTTTEVDNWPADVN---------------
GALCS01621      NLGPV-LITGM----------A----QGGQLMTTTEVDNWPGDVS---------------
LARHH01446      NLNPV-LITGL----------A----QGGQLMTTTEVDNWPADAA---------------
CHRVO02807      NLKPV-VITGL----------A----QGGQLMTTTDVDNWPADAE---------------
PSEUL01648      NLKPV-MITGL----------A----QGGQLMTTTDVDNWPADAD---------------
NITEU01859      NLNPV-LITGM----------A----QGGQLTTTTDVDNWPADVA---------------
NITEC00745      NLNPV-LITGM----------A----QGGQLTTTTDVDNWPADVA---------------
NITMU00023      NLKPM-LVTGL----------A----QGGQLMTTTEVDNWPADAM---------------
NITSI03064      NLNPV-VITGL----------A----QGGQLMTTTDVDNWPADAM---------------
ACCPU02128      NLQPV-LITGM----------A----QGGQLMTTTDVDNWPADAK---------------
THIDA01000      NLSPV-VITGM----------Q----QGGQLMTTTEVDNWPADVD---------------
METS601221      NLKPV-LVTGI----------A----QGGQLMTTTEVDNWPADAD---------------
METGS01186      NLKPV-LVTGI----------A----QGGQLMTTTEVDNWPADAD---------------
METFK00972      NLKPV-LITGI----------A----QGGQLMTTTEVDNWPADAD---------------
METML01428      NLKPV-LITGI----------A----QGGQLMTTTEVDNWPADAD---------------
AROAE03899      NLDPV-LITGL----------A----QGGQLMTTTDVDNWPADAD---------------
THASP01686      NLNPV-LITGL----------A----QGGQLMTTTEVDNWPADAD---------------
AZOSB01359      NLKPV-LVTGL----------A----QGGQLMTTTEVDNWPADAD---------------
BORA102682      NLKPL-LVTGL----------A----QGGQLMTTTEVDNWPADVD---------------
BORPA03415      NLSPA-LITGL----------A----QGGQLMTTTEVDNWPADVQ---------------
BORBM03577      NLSPA-LITGL----------A----QGGQLMTTTEVDNWPADVQ---------------
BORPE02280      NLSPA-LITGL----------A----QGGQLMTTTEVDNWPADVQ---------------
BORPC02060      NLSPA-LITGL----------A----QGGQLMTTTEVDNWPADVQ---------------
BORP102392      NLSPA-LITGL----------A----QGGQLMTTTEVDNWPADVQ---------------
BORBR03870      NLSPA-LITGL----------A----QGGQLMTTTEVDNWPADVQ---------------
BORPD01524      NLAPV-LVTGL----------A----QGGQLMTTTDVDNWPADAT---------------
ACHXA01179      NLSPV-LVTGL----------A----QGGQLMTTTDVDNWPADAD---------------
RHOFT03114      NLKPL-LITGI----------A----QGGQLMTTTEVDNWPADVD---------------
VEREI01701      NLNPV-LITGL----------A----QGGQLMTTTDVDNWPADAD---------------
VARPE01375      NLQPL-LITGI----------A----QGGQLMTTTEVDNWPADVH---------------
VARPS01266      NLQPL-LITGI----------A----QGGQLMTTTEVDNWPADVH---------------
DELAS05272      NLNPL-LVTGM----------A----QGGQLMTTTEVDNWPADVN---------------
DELSC01235      NLNPL-LVTGM----------A----QGGQLMTTTEVDNWPADVN---------------
COMT200883      NLKPL-LITGM----------A----QGGQLMTTTEVDNWPADVM---------------
ACIAC03267      NLNPV-LITGI----------A----QGGQLMTTTEVDNWPADVN---------------
ACIAP03249      NLNPV-LITGI----------A----QGGQLMTTTEVDNWPADVN---------------
ACIET02648      NLNPV-LITGL----------A----QGGQLMTTTEVDNWPADVN---------------
ALIDK03528      NLDPV-LITGM----------A----QGGQLMTTTEVDNWPADVN---------------
RUBGI03408      NLEPL-LVTGI----------A----QGGQLMTTTEVDNWPADVN---------------
LEPCP00749      NLQPM-LVTGM----------A----QGGQLMTTTEVDNWPADVA---------------
POLSJ03719      NLDPV-LITGI----------A----QGGQLMTTTDVDNWPADAQ---------------
POLNA03144      NLNPV-LITGI----------A----QGGQLMTTTDVDNWPADVD---------------
METPP01110      NLEPV-LITGM----------A----QGGQLMTTTEVDNWPADVH---------------
RAMTT03218      NLHPL-LITGI----------A----QGGQLMTTTEVDNWPADVH---------------
RALPJ02457      NLNPV-LVTGL----------A----QGGQLMTTTDVENWPADKE---------------
RALP102102      NLNPV-LVTGL----------A----QGGQLMTTTDVENWPADKE---------------
RALSO02303      NLNPM-LITGL----------A----QGGQLMTTTEVENWPADRE---------------
RALS801097      NLNPM-LITGL----------A----QGGQLMTTTDVENWPADKE---------------
HERSS01878      NLNPV-LITGV----------E----QGGQLMTTTDVENWPGDPL---------------
HERAR00955      NLKPV-LITGV----------E----QGGQLMTTTDVENWPGDPL---------------
JANMA01152      NLKPV-LITGV----------E----QGGQLMTTTDVENWPGDPL---------------
THIK102324      NLNPL-LITGL----------A----QGGQLMTTTEVDNWPADVD---------------
POLSQ00682      NLKPT-LITGL----------A----QGGQLMTTTDVENWPADAD---------------
```

```
POLNS00989      NLNPT-LITGL----------A----QGGQLMTTTDVENWPADAD---------------
BURP800627      NLSPL-LITGL----------A----QGGQLMTTTDVENWPADPN---------------
BURPP00988      NLAPV-LVTGL----------A----QGGQLMTTTDVENWPADPN---------------
BURSC00728      NLAPV-LVTGL----------A----QGGQLMTTTDVENWPADAN---------------
BURXL00982      NLAPV-LVTGL----------A----QGGQLMTTTDVENWPADAN---------------
BURSG00869      NLAPV-LVTGL----------A----QGGQLMTTTDVENWPADAN---------------
BURRH00629      NLSPM-LITGL----------A----QGGQLMTTTDVENWPGDPN---------------
BURGB00781      NLSPL-LITGI----------A----QGGQLMTTTDVENWPADPN---------------
BURGS00853      NLSPL-LITGL----------A----QGGQLMTTTDVENWPADPN---------------
BURPS02618      NLSPL-LITGI----------A----QGGQLMTTTDVENWPADAD---------------
BURMA01741      NLSPL-LITGI----------A----QGGQLMTTTDVENWPADAD---------------
BURP103016      NLSPL-LITGI----------A----QGGQLMTTTDVENWPADAD---------------
BURP002984      NLSPL-LITGI----------A----QGGQLMTTTDVENWPADAD---------------
BURM701866      NLSPL-LITGI----------A----QGGQLMTTTDVENWPADAD---------------
BURP602945      NLSPL-LITGI----------A----QGGQLMTTTDVENWPADAD---------------
BURM902465      NLSPL-LITGI----------A----QGGQLMTTTDVENWPADAD---------------
BURMS00740      NLSPL-LITGI----------A----QGGQLMTTTDVENWPADAD---------------
BURTA01515      NLSPL-LITGI----------A----QGGQLMTTTDVENWPADPN---------------
BURM102386      NLSPV-LITGI----------A----QGGQLMTTTDVENWPADAK---------------
BURL300890      NLSPV-LITGI----------A----QGGQLMTTTDVENWPADAK---------------
BURVG00860      NLSPV-LITGI----------A----QGGQLMTTTDVENWPADAK---------------
BURCM00829      NLSPV-LVTGI----------A----QGGQLMTTTDVENWPADAK---------------
BURA400832      NLSPV-LVTGI----------A----QGGQLMTTTDVENWPADAK---------------
BURCA00489      NLSPV-LITGI----------A----QGGQLMTTTDVENWPADAK---------------
BURCH00966      NLSPV-LITGI----------A----QGGQLMTTTDVENWPADAK---------------
BURCC00927      NLSPV-LITGI----------A----QGGQLMTTTDVENWPADAK---------------
BURCJ02922      NLSPV-LITGI----------A----QGGQLMTTTDVENWPADAK---------------
EDWI902348      NLNPV-LITGM----------E----KGGQLTTTSELENWPGDPE---------------
EDWTF01971      NLNPV-LITGM----------E----KGGQLTTTSELENWPGDPE---------------
EDWTE02176      NLNPV-LITGM----------E----KGGQLTTTSELENWPGDPE---------------
SODGM01094      NLNPV-LITGM----------E----QGGQLTTTTEVENWPGDTE---------------
MOREP00394      NLNPV-LITGM----------E----QGGQLTTTTTVENWPGDPE---------------
RAHSY01432      NLNPV-LITGV----------E----KGGQLTTTTEVENWPGDPE---------------
RAHAC01419      NLNPV-LITGV----------E----KGGQLTTTTEVENWPGDPE---------------
ERWBE01489      NLNPV-LITGL----------E----KGGQLTTTTEVENWPGDPN---------------
PANAM01339      NLNPV-LITGL----------E----KGGQLTTTTEVENWPGDPH---------------
PANAA00662      NLNPV-LITGL----------E----KGGQLTTTTEVENWPGDPH---------------
PANSA01303      NLNPV-LITGL----------E----KGGQLTTTTEVENWPGDPH---------------
ERWT902154      NLNPV-LITGL----------E----KGGQLTTTTDVENWPGDAH---------------
ERWAC01330      NLNPV-LITGL----------E----KGGQLTTTTDVENWPGDAH---------------
ERWAE01326      NLNPV-LITGL----------E----KGGQLTTTTDVENWPGDAH---------------
ERWPE02241      NLNPV-LITGL----------E----KGGQLTTTTDVENWPGDAH---------------
ERWP602414      NLNPV-LITGL----------E----KGGQLTTTTDVENWPGDAH---------------
ERWSE02396      NLNPV-LITGL----------E----KGGQLTTTTDVENWPGDAH---------------
PECCP01694      NLQPV-LITGM----------E----KGGQLTTTTEVENWPGDAD---------------
PECWW01889      NLQPV-LITGM----------E----KGGQLTTTTEVENWPGDAD---------------
PECSS01870      NLQPV-LITGM----------E----KGGQLTTTTEVENWPGDAD---------------
PECAS02624      NLQPV-LITGM----------E----KGGQLTTTTEVENWPGDAD---------------
DICDC02220      NLQPL-LITGM----------E----KGGQLTTTTEVENWPGDPN---------------
DICZE02284      NLNPL-LITGM----------E----KGGQLTTTTEVENWPGDPD---------------
DICD302017      NLNPL-LITGM----------E----KGGQLTTTTEVENWPGDPD---------------
DICD502250      NLSPL-LITGM----------E----KGGQLTTTTEVENWPGDAD---------------
XENBS00829      NLNPV-LITGV----------E----KGGQLTTTTEVENWPGDPE---------------
XENNA01480      NLNPV-LITGV----------E----KGGQLTTTTEVENWPGDPE---------------
PHOLL01537      NLNPV-LITGV----------E----RGGQLTTTTEVENWPGDPE---------------
PHOAA02804      NLNPA-LITGV----------E----KGGQLTTTTEVENWPGDPE---------------
SERP501669      NLSPV-LITGM----------E----QGGQLTTTTEVENWPGDAE---------------
SERSA01625      NLSPV-LITGM----------E----QGGQLTTTTEVENWPGDAE---------------
YERPE01271      NLKPV-LITGM----------E----KGGQLTTTTEVENWPGDPE---------------
YERPS01390      NLKPV-LITGM----------E----KGGQLTTTTEVENWPGDPE---------------
YERPA00627      NLKPV-LITGM----------E----KGGQLTTTTEVENWPGDPE---------------
YERPN02489      NLKPV-LITGM----------E----KGGQLTTTTEVENWPGDPE---------------
YERPP02190      NLKPV-LITGM----------E----KGGQLTTTTEVENWPGDPE---------------
YERP302544      NLKPV-LITGM----------E----KGGQLTTTTEVENWPGDPE---------------
YERPB01460      NLKPV-LITGM----------E----KGGQLTTTTEVENWPGDPE---------------
```

```
YERPY02633      NLKPV-LITGM----------E----KGGQLTTTTEVENWPGDPE---------------
YERPG01392      NLKPV-LITGM----------E----KGGQLTTTTEVENWPGDPE---------------
YERPD01171      NLKPV-LITGM----------E----KGGQLTTTTEVENWPGDPE---------------
YERP100890      NLKPV-LITGM----------E----KGGQLTTTTEVENWPGDPE---------------
YERPZ01206      NLKPV-LITGM----------E----KGGQLTTTTEVENWPGDPE---------------
YERPH02469      NLKPV-LITGM----------E----KGGQLTTTTEVENWPGDPE---------------
YERE801438      NLNPV-LITGM----------E----KGGQLTTTTDVENWPGDPE---------------
YERE302565      NLNPV-LITGM----------E----KGGQLTTTTDVENWPGDPE---------------
YERE100392      NLNPV-LITGM----------E----KGGQLTTTTDVENWPGDPE---------------
PROMH00687      NLEPV-LITGV----------E----KGGQLTTTTEVENWPGDPE---------------
PROSM03196      NLSPA-LITGV----------E----KGGQLTTTTEVENWPGDPE---------------
TOLAT02279      NLNPV-LITGM----------Q----QGGQLTTTTEVENWPGDAE---------------
AERVB02262      NLNPL-LITGM----------Q----QGGQLTTTTEVENWPGDPE---------------
AERHH01811      NLNPL-LITGM----------Q----QGGQLTTTTEVENWPGDPE---------------
AERS402185      NLNPL-LITGM----------Q----QGGQLTTTTEVENWPGDSE---------------
PSYIN02070      NLNPV-LITGM----------E----QGGQLTTTTDVENWPGGSA---------------
SHELP02018      NLKPV-LITGM----------Q----QGGQLTTTTEVENWPGDAD---------------
SHEVD02297      NLKPV-LITGI----------Q----QGGQLTTTTEVENWPGDAD---------------
SHEPW02341      NLKPV-MITGM----------Q----QGGQLTTTTEVENWPGDAD---------------
SHEPA02202      NLKPV-LITGI----------Q----QGGQLTTTTEVENWPGDAE---------------
SHEHH02007      NLKPV-LITGI----------Q----QGGQLTTTTEVENWPGDAE---------------
SHESH02117      NLKPV-LVTGL----------Q----QGGQLTTTTEVENWPGDAE---------------
SHEWM02454      NLKPV-LITGI----------Q----QGGQLTTTTEVENWPGDAE---------------
SHEAM01758      NLNPV-MITGL----------Q----QGGQLTTTTEVENWPGDAE---------------
SHEON02159      NLKPV-MITGM----------Q----QGGQLTTTTEVENWPGDAE---------------
SHESM01945      NLKPV-MITGM----------Q----QGGQLTTTTEVENWPGDAE---------------
SHESR01991      NLKPV-MITGM----------Q----QGGQLTTTTEVENWPGDAE---------------
SHESA02025      NLKPV-MITGM----------Q----QGGQLTTTTEVENWPGDAE---------------
SHESW01953      NLKPV-MITGM----------Q----QGGQLTTTTEVENWPGDAD---------------
SHEPC01976      NLKPV-MITGM----------Q----QGGQLTTTTEVENWPGDAD---------------
SHEP201929      NLKPV-MITGM----------Q----QGGQLTTTTEVENWPGDAD---------------
SHEB502016      NLKPV-MITGM----------Q----QGGQLTTTTEVENWPGDAD---------------
SHEB802168      NLKPV-MITGM----------Q----QGGQLTTTTEVENWPGDAD---------------
SHEB202117      NLKPV-MITGM----------Q----QGGQLTTTTEVENWPGDAD---------------
SHEB902270      NLKPV-MITGM----------Q----QGGQLTTTTEVENWPGDAD---------------
SHEB602258      NLKPV-MITGM----------Q----QGGQLTTTTEVENWPGDAD---------------
SHEDO01680      NLKPV-MITGM----------Q----QGGQLTTTTEVENWPGDAD---------------
SHEFN01895      NLNPV-MITGM----------Q----QGGQLTTTTEVENWPGDAE---------------
PSEHT01677      NLNPV-LITGM----------Q----QGGQLTTTTEVENWPGDAH---------------
ALISL01611      NLNPV-MITGM----------Q----QGGQLTTTTEVENWPGGAT---------------
VIBF100893      NLNPV-MITGM----------Q----QGGQLTTTTEVENWPGGAA---------------
VIBFM00891      NLNPV-MITGM----------Q----QGGQLTTTTEVENWPGGAA---------------
OCESG01399      NLSPV-LVTGI----------Q----QGGQLTTTTEVENWPGDAE---------------
VIBA701872      NLNPV-LVTGM----------Q----QGGQLTTTTEVENWPGDAE---------------
VIBVY01451      NLNPV-LITGM----------Q----QGGQLTTTTEVENWPGDPE---------------
VIBVU02545      NLNPV-LITGM----------Q----QGGQLTTTTEVENWPGDPE---------------
VIBVM01748      NLNPV-LITGM----------Q----QGGQLTTTTEVENWPGDPE---------------
VIBCH01157      NLKPV-LVTGM----------Q----QGGQLTTTTEVENWPGDAE---------------
VIBCM01114      NLKPV-LVTGM----------Q----QGGQLTTTTEVENWPGDAE---------------
VIBCJ02064      NLKPV-LVTGM----------Q----QGGQLTTTTEVENWPGDAE---------------
VIBC300725      NLKPV-LVTGM----------Q----QGGQLTTTTEVENWPGDAE---------------
FERBD01969      NLNPV-MITGM----------Q----QGGQLTTTTEVENWPGDPE---------------
VIBFN02085      NLNPV-LVTGM----------Q----QGGQLTTTTEVENWPGDAE---------------
VIBTL01089      NLNPV-LVTGM----------Q----QGGQLTTTTEVENWPGDPE---------------
VIBPA01248      NLNPV-LVTGM----------Q----QGGQLTTTTEVENWPGDAE---------------
VIBAE02128      NLNPV-LVTGM----------Q----QGGQLTTTTEVENWPGDAE---------------
VIBCB00599      NLNPV-LVTGM----------Q----QGGQLTTTTEVENWPGDAE---------------
PANVC00701      NLNPV-LITGL----------E----KGGQLTTTTEVENWPGDPH---------------
SHIBC02443      NLNPV-LITGV----------E----KGGQLTTTTEVENWPGDAS---------------
ENTBF02792      NLHPV-LITGM----------E----KGGQLTTTTEVENWPGDPN---------------
KLEP700892      NLQPV-LITGM----------E----KGGQLTTTTEVENWPGDPN---------------
KLEPH01782      NLQPV-LITGM----------E----KGGQLTTTTEVENWPGDPN---------------
KLEP303551      NLQPV-LITGM----------E----KGGQLTTTTEVENWPGDPN---------------
KLEVT03420      NLQPV-LITGM----------E----KGGQLTTTTEVENWPGDPN---------------
ENTAK02985      NLQPV-LITGM----------E----KGGQLTTTTEVENWPGDPN---------------
```

```
KLEOK03151      NLQPV-LITGM----------E----KGGQLTTTTEVENWPGDPN---------------
SALAR01925      NLQPV-LITGM----------E----KGGQLTTTTEVENWPGDPN---------------
SALBC00812      NLQPV-LITGM----------E----KGGQLTTTTDVENWPGDPN---------------
SALPC00926      NLQPV-LITGM----------E----KGGQLTTTTEVENWPGDPN---------------
SALTI00868      NLQPV-LITGM----------E----KGGQLTTTTEVENWPGDPN---------------
SALCH00905      NLQPV-LITGM----------E----KGGQLTTTTEVENWPGDPN---------------
SALPA01706      NLQPV-LITGM----------E----KGGQLTTTTEVENWPGDPN---------------
SALTY00927      NLQPV-LITGM----------E----KGGQLTTTTEVENWPGDPN---------------
SALPK01786      NLQPV-LITGM----------E----KGGQLTTTTEVENWPGDPN---------------
SALHS00978      NLQPV-LITGM----------E----KGGQLTTTTEVENWPGDPN---------------
SALEP00854      NLQPV-LITGM----------E----KGGQLTTTTEVENWPGDPN---------------
SALDC00926      NLQPV-LITGM----------E----KGGQLTTTTEVENWPGDPN---------------
SALA400892      NLQPV-LITGM----------E----KGGQLTTTTEVENWPGDPN---------------
SALG200876      NLQPV-LITGM----------E----KGGQLTTTTEVENWPGDPN---------------
SALTS00888      NLQPV-LITGM----------E----KGGQLTTTTEVENWPGDPN---------------
SALT400905      NLQPV-LITGM----------E----KGGQLTTTTEVENWPGDPN---------------
SALPS01908      NLQPV-LITGM----------E----KGGQLTTTTEVENWPGDPN---------------
SALT101040      NLQPV-LITGM----------E----KGGQLTTTTEVENWPGDPN---------------
SALTD00960      NLQPV-LITGM----------E----KGGQLTTTTEVENWPGDPN---------------
SALPB02472      NLQPV-LITGM----------E----KGGQLTTTTEVENWPGDPN---------------
SALNS00928      NLQPV-LITGM----------E----KGGQLTTTTEVENWPGDPN---------------
SALSV01000      NLQPV-LITGM----------E----KGGQLTTTTEVENWPGDPN---------------
ECOS500812      NLQPV-LITGM----------E----KGGQLTTTTEVENWPGDPN---------------
ECOL600981      NLQPV-LITGM----------E----KGGQLTTTTEVENWPGDPN---------------
ECOL500883      NLQPV-LITGM----------E----KGGQLTTTTEVENWPGDPN---------------
ECOUT00876      NLQPV-LITGM----------E----KGGQLTTTTEVENWPGDPN---------------
ECOK100776      NLQPV-LITGM----------E----KGGQLTTTTEVENWPGDPN---------------
ECOSM02148      NLQPV-LITGM----------E----KGGQLTTTTEVENWPGDPN---------------
ECOLU01057      NLQPV-LITGM----------E----KGGQLTTTTEVENWPGDPN---------------
ECO7I02136      NLQPV-LITGM----------E----KGGQLTTTTEVENWPGDPN---------------
ECO8100828      NLQPV-LITGM----------E----KGGQLTTTTEVENWPGDPN---------------
ECO4500862      NLQPV-LITGM----------E----KGGQLTTTTEVENWPGDPN---------------
ECOAB00879      NLQPV-LITGM----------E----KGGQLTTTTEVENWPGDPN---------------
ECO4400961      NLQPV-LITGM----------E----KGGQLTTTTEVENWPGDPN---------------
ECOUM02550      NLQPV-LITGM----------E----KGGQLTTTTEVENWPGDPN---------------
ECOKI00869      NLQPV-LITGM----------E----KGGQLTTTTEVENWPGDPN---------------
ECOC100920      NLQPV-LITGM----------E----KGGQLTTTTEVENWPGDPN---------------
ECOC200920      NLQPV-LITGM----------E----KGGQLTTTTEVENWPGDPN---------------
ECO8N00803      NLQPV-LITGM----------E----KGGQLTTTTEVENWPGDPN---------------
ECO2700862      NLQPV-LITGM----------E----KGGQLTTTTEVENWPGDPN---------------
ECO2600974      NLQPV-LITGM----------E----KGGQLTTTTEVENWPGDPN---------------
ECOH100942      NLQPV-LITGM----------E----KGGQLTTTTEVENWPGDPN---------------
SHIB301848      NLQPV-LITGM----------E----KGGQLTTTTEVENWPGDPN---------------
ECOLI00846      NLQPV-LITGM----------E----KGGQLTTTTEVENWPGDPN---------------
ECO5700970      NLQPV-LITGM----------E----KGGQLTTTTEVENWPGDPN---------------
SHISS00756      NLQPV-LITGM----------E----KGGQLTTTTEVENWPGDPN---------------
SHIBS00679      NLQPV-LITGM----------E----KGGQLTTTTEVENWPGDPN---------------
SHIDS01857      NLQPV-LITGM----------E----KGGQLTTTTEVENWPGDPN---------------
ECO2400906      NLQPV-LITGM----------E----KGGQLTTTTEVENWPGDPN---------------
ECODH00784      NLQPV-LITGM----------E----KGGQLTTTTEVENWPGDPN---------------
ECOHS00922      NLQPV-LITGM----------E----KGGQLTTTTEVENWPGDPN---------------
ECOLC02627      NLQPV-LITGM----------E----KGGQLTTTTEVENWPGDPN---------------
ECO5E00946      NLQPV-LITGM----------E----KGGQLTTTTEVENWPGDPN---------------
ECOSE00936      NLQPV-LITGM----------E----KGGQLTTTTEVENWPGDPN---------------
ECO5500894      NLQPV-LITGM----------E----KGGQLTTTTEVENWPGDPN---------------
ECO8A00877      NLQPV-LITGM----------E----KGGQLTTTTEVENWPGDPN---------------
ECOBB00863      NLQPV-LITGM----------E----KGGQLTTTTEVENWPGDPN---------------
ECO5T00943      NLQPV-LITGM----------E----KGGQLTTTTEVENWPGDPN---------------
ECOBW00723      NLQPV-LITGM----------E----KGGQLTTTTEVENWPGDPN---------------
ECO1000919      NLQPV-LITGM----------E----KGGQLTTTTEVENWPGDPN---------------
ECOBD02604      NLQPV-LITGM----------E----KGGQLTTTTEVENWPGDPN---------------
ECOD102659      NLQPV-LITGM----------E----KGGQLTTTTEVENWPGDPN---------------
ECOBR00856      NLQPV-LITGM----------E----KGGQLTTTTEVENWPGDPN---------------
ECOLX02610      NLQPV-LITGM----------E----KGGQLTTTTEVENWPGDPN---------------
ECO1A00939      NLQPV-LITGM----------E----KGGQLTTTTEVENWPGDPN---------------
```

```
ECOCB01054      NLQPV-LITGM----------E----KGGQLTTTTEVENWPGDPN---------------
ECOKO02859      NLQPV-LITGM----------E----KGGQLTTTTEVENWPGDPN---------------
ECO1E03320      NLQPV-LITGM----------E----KGGQLTTTTEVENWPGDPN---------------
ECOLW01217      NLQPV-LITGM----------E----KGGQLTTTTEVENWPGDPN---------------
SHIFL01399      NLQPV-LITGM----------E----KGGQLTTTTEVENWPGDPN---------------
SHIF800759      NLQPV-LITGM----------E----KGGQLTTTTEVENWPGDPN---------------
SHIF200805      NLQPV-LITGM----------E----KGGQLTTTTEVENWPGDPN---------------
CITK802118      NLQPV-LITGM----------E----KGGQLTTTTEVENWPGDPN---------------
CITRI00924      NLQPV-LITGM----------E----KGGQLTTTTEVENWPGDPN---------------
CROS802385      NLKPV-LITGM----------E----KGGQLTTTTEVENWPGDPH---------------
CROTZ01500      NLKPV-LITGM----------E----KGGQLTTTTEVENWPGDPH---------------
ENTLS02865      NLQPV-LITGM----------E----KGGQLTTTTEVENWPGDPH---------------
ENT3801400      NLQPV-LITGM----------E----KGGQLTTTTEVENWPGDPH---------------
ENTAL01384      NLQPV-LITGM----------E----KGGQLTTTTEVENWPGDPN---------------
ENTCC02679      NLQPV-LITGM----------E----KGGQLTTTTEVENWPGDPN---------------
                                                    : :: .

STRT101547      ----------SISGPELSMKMFEPLEKL-GV--EN--L-YGI-V-SGIED-RGNY-----
STRT201508      ----------SISGPELSMKMFEPLEKL-GV--EN--L-YGI-V-SGIED-RGNY-----
STRTD01356      ----------SISGPELSMKMFEPLEKL-GV--EN--L-YGI-V-SGIED-RGNY-----
STRTN01533      ----------SISGPELSMKMFEPLEKL-GV--EN--L-YGI-V-SGIED-RGNY-----
STRE500345      ----------SISGPELSMKMFEPLEKL-GV--EN--L-YGI-V-SGIED-KGDY-----
STRE801625      ----------SISGPELSMKMFEPLEKL-GV--EN--L-YGI-V-SGIED-KGDY-----
STREH01636      ----------SISGPELSMKMFEPLEKL-GV--EN--L-YGI-V-SGIED-KGDY-----
STREC01656      ----------HISGPELSLKMYEPLEKF-KV--ES--I-YGI-V-QRVED-CGDY-----
STREM01495      ----------HISGPELSLKMYEPLEKF-KV--EN--I-YGI-V-QRVED-CGDY-----
STRE401629      ----------HISGPELSLKMYEPLEKF-KV--EN--I-YGI-V-QRVED-CGDY-----
STRS700387      ----------HISGPELSLKMYEPLEKF-KV--EN--I-YGI-V-QRVED-CGDY-----
STRDG01631      ----------HISGPELSMKMYEPLEKF-KV--EN--I-YGI-V-QKVED-FGSY-----
STRP301388      ----------HISGPELAMKMYEPLEKF-NV--EN--I-YGI-V-QKIEN-FGDY-----
STRPZ01205      ----------HISGPELAMKMYEPLEKF-NV--EN--I-YGI-V-QKIEN-FGDY-----
STRPQ00464      ----------HISGPELAMKMYEPLEKF-NV--EN--I-YGI-V-QKIEN-FGDY-----
STRPD01453      ----------HISGPELAMKMYEPLEKF-NV--EN--I-YGI-V-QKIEN-FGDY-----
STRP601399      ----------HISGPELAMKMYEPLEKF-NV--EN--I-YGI-V-QKIEN-FGDY-----
STRP801366      ----------HISGPELAMKMYEPLEKF-NV--EN--I-YGI-V-QKIEN-FGDY-----
STRPF01453      ----------HISGPELAMKMYEPLEKF-NV--EN--I-YGI-V-QKIEN-FGDY-----
STRPG00427      ----------HISGPELAMKMYEPLEKF-NV--EN--I-YGT-V-QKIEN-FGDY-----
STRA300284      ----------HISGPELSMKMYEPLEKF-EV--EH--I-YGI-V-QRVEN-DGDV-----
STRA500290      ----------HISGPELSMKMYEPLEKF-EV--EH--I-YGI-V-QRVEN-DGDV-----
STRA100288      ----------HISGPELSMKMYEPLEKF-EV--EH--I-YGI-V-QRVEN-DGDV-----
STRA200291      ----------HISGPELSMKMYEPLEKF-EV--EH--I-YGI-V-QRVEN-DGDV-----
STRIC00348      ----------HISGPELSMKMHAPLEKF-GV--EN--I-YGI-V-KSIED-AGDV-----
STRPX00375      ----------HISGPELSMNMHAPLEKF-GV--EN--I-YGI-V-KSIED-AGDV-----
STRMD00432      ----------HISGPELSMNMHAPLEKF-GV--EN--I-YGI-V-KSIED-AGDV-----
STRS201734      ----------HISGPALAEKMFEPLEKF-GV--DH--I-FGT-L-VRIEE-EGQI-----
STRSY01721      ----------HISGPALAEKMFEPLEKF-GV--DH--I-FGT-L-VRIEE-EGQI-----
STRSX01553      ----------HISGPALAEKMFEPLEKF-GV--DH--I-FGT-L-VRIEE-EGQI-----
STRSE01478      ----------HISGPALAEKMFEPLEKF-GV--DH--I-FGT-L-VRIEE-EGQI-----
STREJ01635      ----------HISGPALAEKMFEPLEKF-GV--DH--I-FGT-L-VRIEE-EGQI-----
STRGZ01544      ----------HISGPALAEKMFEPLEKF-GV--DH--I-FGT-L-VRIEE-EGQI-----
STRS401593      ----------HISGPALAEKMFEPLEKF-GV--DH--I-FGT-L-VRIEE-EGQI-----
LACGT00720      ----------QIMGPELSMKMYEPLADL-GV--EN--A-YGF-V-TSIVD-NGAT-----
LACGL00738      ----------QIMGPELSMKMYEPLADL-GV--EN--A-YGF-V-TSIVD-NGAT-----
STRSV01774      ----------NISGPELAEKMFEPLENL-GV--EH--L-FGL-V-EKIED-RGDF-----
STRIJ00350      ----------NISGPELAEKMFEPLENL-GV--EH--L-FGV-V-EKIEQ-QGTI-----
STROU01248      ----------NISGPELAEKMFEPLENL-GV--EH--L-YGY-V-ENIED-KGDY-----
STRM601286      ----------NISGPELAEKMFEPLENL-GV--EH--I-YGY-V-ENVED-HGHF-----
STRES00962      ----------NISGPELAEKMFEPLENL-GV--EH--I-YGY-V-ENVED-HGDF-----
STRP701387      ----------NISGPELAEKMFEPLENL-GV--EH--I-YGY-V-ENVED-HGDF-----
STRZT00756      ----------NISGPELAEKMFEPLENL-GV--EH--I-YGY-V-ENVED-HGDF-----
STRP001079      ----------NISGPELAEKMFEPLENL-GV--EH--I-YGY-V-ENVED-HGDF-----
STRZO01270      ----------NISGPELAEKMFEPLENL-GV--EH--I-YGY-V-ENVED-HGDF-----
STRZ600815      ----------NISGPELAEKMFEPLENL-GV--EH--I-YGY-V-ENVED-HGDF-----
STRET00826      ----------NISGPELAEKMFEPLENL-GV--EH--I-YGY-V-ENVED-HGDF-----
STRPS01429      ----------NISGPELAEKMFEPLENL-GV--EH--I-YGY-V-ENVED-HGDF-----
```

```
STRZN01287      ----------NISGPELAEKMFEPLENL-GV--EH--I-YGY-V-ENVED-HGDF-----
STRR601306      ----------NISGPELAEKMFEPLENL-GV--EH--I-YGY-V-ENVED-HGDF-----
STRP201217      ----------NISGPELAEKMFEPLENL-GV--EH--I-YGY-V-ENVED-HGDF-----
STRZP01342      ----------NISGPELAEKMFEPLENL-GV--EH--I-YGY-V-ENVED-HGDF-----
STRZI01226      ----------NISGPELAEKMFEPLENL-GV--EH--I-YGY-V-ENVED-HGDF-----
STRPN01360      ----------NISGPELAEKMFEPLENL-GV--EH--I-YGY-V-ENVED-HGDF-----
STRP401357      ----------NISGPELAEKMFEPLENL-GV--EH--I-YGY-V-ENVED-HGDF-----
STRZJ01282      ----------NISGPELAEKMFEPLENL-GV--EH--I-YGY-V-ENVED-HGDF-----
STRPJ01336      ----------NISGPELAEKMFEPLENL-GV--EH--I-YGY-V-ENVED-HGDF-----
STRPI01436      ----------NISGPELAEKMFEPLENL-GV--EH--I-YGY-V-ENVED-HGDF-----
MARHT00044      ----------GISGPELAQRMAEQAQKF-GA--EI--V-MDE-V-QGLER-TEDG-----
THEP300413      ----------EISGADLIAKMEAQVRKH-GL--EI--V-NED-V-ESLDI-TGDV-----
THEPX00884      ----------EISGADLIAKMEAQVRKH-GL--EI--V-NED-V-ESLDI-TGDV-----
THESX01857      ----------EISGADLIAKMEAQVRKH-GL--EI--V-NED-V-ESLDI-TGDV-----
THEM301788      ----------EISGADLVAKMEAQVRKH-GL--EI--V-NED-V-ESLDI-TGDV-----
THEIA01812      ----------EISGADLVAKMEAQVRKH-GL--EI--V-NED-V-ESLDI-TGDV-----
THETC00443      ----------GISGPDLINKMEAQAKSY-GL--QI--Y-NEE-V-VGLDI-TGKV-----
THESW01026      ----------GISGPDLINKMESQAKRY-GL--QI--Y-NEE-V-VGLDI-TGSV-----
THEXL00360      ----------GISGPDLINKMESQAKRY-GL--QI--Y-NEE-V-VGLDI-TGNV-----
THEID01127      ----------GISGFELIESFVTQVKKL-GF--EP--L-REE-V-IRLED-LGQN-----
THEOJ00147      ----------GIGGAELTEAMKKQAERF-GA--QF--L-NGN-V-EKIEK-VGEK-----
DESAS01237      ----------GISGPELAMNMESQAKRF-GL--EV--I-YDY-V-EELKP-LNNN-----
DESK701373      ----------GIGGPELMSRMEAQARRF-GL--EF--L-NSN-V-EALKK-ENLN-----
KYRT200729      ----------SILGPDLSEKMREHAESL-GL--QT--E-RGE-V-ESLEF-GPPV-----
STACT00410      ----------SISGPDLSTKMFDHAQKF-GA--VY--Q-YGD-I-KSIED-EGDV-----
STAS101952      ----------MVTGPDLSTKMFEHAKKF-GA--QY--Q-YGD-I-KSIED-KGAY-----
STALH01969      ----------MITGPDLSTKMFEHAKKF-GA--EY--Q-YGD-I-KSIED-QGDY-----
STAEQ00422      ----------MITGPDLSTKMFEHAKKF-GA--EY--Q-YGD-I-KSVED-KGDY-----
STAES00543      ----------MITGPDLSTKMFEHAKKF-GA--EY--Q-YGD-I-KSVED-KGDY-----
STAAB00714      ----------MITGPDLSTKMFEHAKKF-GA--VY--Q-YGD-I-KSVED-KGEY-----
STAA500765      ----------MITGPDLSTKMFEHAKKF-GA--VY--Q-YGD-I-KSVED-KGEY-----
STAAM00757      ----------MITGPDLSTKMFEHAKKF-GA--VY--Q-YGD-I-KSVED-KGEY-----
STAAW00726      ----------MITGPDLSTKMFEHAKKF-GA--VY--Q-YGD-I-KSVED-KGEY-----
STAAS00733      ----------MITGPDLSTKMFEHAKKF-GA--VY--Q-YGD-I-KSVED-KGEY-----
STAAN00727      ----------MITGPDLSTKMFEHAKKF-GA--VY--Q-YGD-I-KSVED-KGEY-----
STAAC00807      ----------MITGPDLSTKMFEHAKKF-GA--VY--Q-YGD-I-KSVED-KGEY-----
STAA300727      ----------MITGPDLSTKMFEHAKKF-GA--VY--Q-YGD-I-KSVED-KGEY-----
STAA800734      ----------MITGPDLSTKMFEHAKKF-GA--VY--Q-YGD-I-KSVED-KGEY-----
STAA100752      ----------MITGPDLSTKMFEHAKKF-GA--VY--Q-YGD-I-KSVED-KGEY-----
STAA200774      ----------MITGPDLSTKMFEHAKKF-GA--VY--Q-YGD-I-KSVED-KGEY-----
STAA900759      ----------MITGPDLSTKMFEHAKKF-GA--VY--Q-YGD-I-KSVED-KGEY-----
STAAE00716      ----------MITGPDLSTKMFEHAKKF-GA--VY--Q-YGD-I-KSVED-KGEY-----
STAAT00767      ----------MITGPDLSTKMFEHAKKF-GA--VY--Q-YGD-I-KSVED-KGEY-----
STAAD00695      ----------MITGPDLSTKMFEHAKKF-GA--VY--Q-YGD-I-KSVED-KGEY-----
STAA000815      ----------MITGPDLSTKMFEHAKKF-GA--VY--Q-YGD-I-KSVED-KGEY-----
STAAH02407      ----------MITGPDLSTKMFEHAKKF-GA--VY--Q-YGD-I-KSVED-KGEY-----
STAAF00763      ----------MITGPDLSTKMFEHAKKF-GA--VY--Q-YGD-I-KSVED-KGEY-----
STAAK00744      ----------MITGPDLSTKMFEHAKKF-GA--VY--Q-YGD-I-KSVED-KGEY-----
STAAJ00703      ----------MITGPDLSTKMFEHAKKF-GA--VY--Q-YGD-I-KSVED-KGEY-----
STAAG00690      ----------MITGPDLSTKMFEHAKKF-GA--VY--Q-YGD-I-KSVED-KGEY-----
STAA400732      ----------MITGPDLSTKMFEHAKKF-GA--VY--Q-YGD-I-KSVED-KGEY-----
STAAR00788      ----------MITGPDLSTKMFEHAKKF-GA--VY--Q-YGD-I-KSVED-KGEY-----
LISSS02378      ----------SILGPDLSDKMLSGAKQF-GA--EY--A-YGD-I-KEVID-GKEF-----
LISIN02590      ----------SILGPDLSDKMLSGAKQF-GA--EY--A-YGD-I-KEVID-GKEF-----
LISW602421      ----------SILGPDLSDKMLSGAKQF-GA--EY--A-YGD-I-KEVVD-GKEF-----
EXISA00870      ----------SILGPDLSQKMFDHSKAF-GA--EY--A-YGD-V-QRISD-ETEY-----
EXIS202358      ----------SILGPDLSQKMFDHSKAF-GA--EY--A-YGD-V-RSIED-GPAF-----
EXIAB02180      ----------SILGPDLSQKMFDHSKAF-GA--EY--A-YGD-V-RSIED-GPAY-----
OCEIH02462      ----------NILGPDLSNKMFEHAKKF-GA--AY--A-YGD-I-KEVED-HGEY-----
BACIE01058      ----------SILGPDLSQKMFEHSRKF-GA--EY--Q-YGD-V-KEIID-GKEY-----
BACCJ03468      ----------SILGPELSTKMFDHARKF-GA--EY--Q-YGD-V-KEIID-GKEY-----
BACHD03507      ----------HILGPELSTKMFEHAKKF-GA--EY--A-YGD-I-KEIID-QGDL-----
BACPE03382      ----------HILGPELSTKMFEHAKKF-GA--EY--G-YGD-V-KEIID-EGNY-----
SOLSS00743      ----------TILGPELSTKMFEHAKKF-GA--EY--A-YGD-V-NEIID-GEEY-----
BACC600715      ----------SILGPELSNKMFEHAKKF-GA--EY--A-YGD-I-KGIDT-DGDY-----
```

ANOFW02486	----------HILGPELATKMFEHAKKF-GA--EY--A-YGD-V-KEVID-GEEY-----
GEOKA03042	----------TILGPELAAKMFEHAKKF-GA--EY--A-YGD-V-KEIID-GEAY-----
GEOSY02976	----------TILGPELATKMFEHAKKF-GA--EY--A-YGD-V-KEIID-GEAY-----
GEOTN02952	----------TILGPELATKMFEHAKKF-GA--EY--A-YGD-V-KEIID-GEAY-----
GEOSW02588	----------NILGPELATKMFEHAKKF-GA--EY--A-YGE-V-KEIID-GEAY-----
GEOS000381	----------QILGPELATKMFEHAKKF-GA--EY--A-YGE-V-KEIID-GEAY-----
GEOTC00378	----------QILGPELATKMFEHAKKF-GA--EY--A-YGE-V-KEIID-GEAY-----
BACMD04906	----------HILGPDLSNKMFEHAKKF-GA--EY--A-YGD-I-KEIID-GEEY-----
BACMQ04908	----------HILGPDLSNKMFEHAKKF-GA--EY--A-YGD-I-KEIID-GEEY-----
BACWK04830	----------SILGPDLSNKMFEHAKKF-GA--EY--A-YGD-V-KAIID-GKEY-----
BACAN04889	----------SILGPDLSNKMFEHAKKF-GA--EY--A-YGD-V-KEVID-GKEY-----
BACC105210	----------SILGPDLSNKMFEHAKKF-GA--EY--A-YGD-V-KEVID-GKEY-----
BACC705051	----------SILGPDLSNKMFEHAKKF-GA--EY--A-YGD-V-KEVID-GKEY-----
BACC005112	----------SILGPDLSNKMFEHAKKF-GA--EY--A-YGD-V-KEVID-GKEY-----
BACC305043	----------SILGPDLSNKMFEHAKKF-GA--EY--A-YGD-V-KEVID-GKEY-----
BACAC05232	----------SILGPDLSNKMFEHAKKF-GA--EY--A-YGD-V-KEVID-GKEY-----
BACAA04720	----------SILGPDLSNKMFEHAKKF-GA--EY--A-YGD-V-KEVID-GKEY-----
BACT005085	----------SILGPDLSNKMFEHAKKF-GA--EY--A-YGD-V-KEVID-GKEY-----
BACC205124	----------SILGPDLSNKMFEHAKKF-GA--EY--A-YGD-V-KEVID-GKEY-----
BACC405064	----------SILGPDLSNKMFEHAKKF-GA--EY--A-YGD-V-KEVID-GKEY-----
BACT104712	----------SILGPDLSNKMFEHAKKF-GA--EY--A-YGD-V-KEVID-GKEY-----
BACLD03769	----------SILGPELSNKMFEHAKKF-GA--EY--A-YGD-I-KEVVD-EGDY-----
BACPZ03376	----------SILGPELSNKMFEHAKKF-GA--EY--A-YGD-I-KEVVD-GKEY-----
BACSU03600	----------SILGPELSNKMFEHAKKF-GA--EY--A-YGD-I-KEVID-GKEY-----
BACST01653	----------SILGPELSNKMFEHAKKF-GA--EY--A-YGD-I-KEVID-GKEY-----
BACPT03541	----------SILGPELSNKMFEHAKKF-GA--EY--A-YGD-I-KEVVD-GKEY-----
LEUGG00661	----------SILGPDLSEKMYASSTRF-GA--EY--G-FGT-V-ENIEI-DGHN-----
LEUGJ00629	----------SILGPDLSEKMYASSTRF-GA--EY--G-FGT-V-ENIEI-DGHN-----
LEUCJ00485	----------SIMGPDLAEKMYASSTQF-GA--EY--G-FGT-V-ESIEM-AGDL-----
LACAR00657	----------EIKGPELSQKMYDTLMKF-EP--DY--K-YGN-V-QSVEL-DGDE-----
LACA300647	----------EIKGPELSQKMYDTLMKF-EP--DY--K-YGN-V-QSVEL-DGDE-----
LACAL00643	----------EIKGPELSQKMYDTLMKF-EP--DY--K-YGN-V-QSVEL-DGDE-----
LACKZ00964	----------EIKGPELSQKMYDTLMKF-EP--DY--K-YGN-V-QTVAL-DGNE-----
LACRJ00357	----------SVKGPELAQQMYEGATQF-GA--EY--A-YGT-V-TKVEL-DGDL-----
LACRD00361	----------SVKGPELAQQMYEGATQF-GA--EY--A-YGT-V-TKVEL-DGDL-----
LACRS01501	----------SVKGPELAQQMYEGATQF-GA--EY--A-YGT-V-TKVEL-DGDL-----
LACSM00455	----------SILGPKLSEEMYESSTQF-GV--EY--G-YGE-V-TGIEI-DGDK-----
LACRG00878	----------SILGPDLGQKMYDGATQF-GA--EY--A-YGN-V-ISVQN-HGAT-----
LACRL00939	----------SILGPDLGQKMYDGATQF-GA--EY--A-YGN-V-ISVQN-HGAT-----
LACC300844	----------SILGPDLGQKMYDGATQF-GA--EY--A-YGN-V-VSVQN-RGAV-----
LACCZ00824	----------SILGPDLGQKMYDGATQF-GA--EY--A-YGN-V-VSVQN-RGAV-----
LACCB00988	----------SILGPDLGQKMYDGATQF-GA--EY--A-YGN-V-VSVQN-RGAV-----
LACCD01016	----------SILGPDLGQKMYDGATQF-GA--EY--A-YGN-V-VSVQN-RGAV-----
LACCC01014	----------SILGPDLGQKMYDGATQF-GA--EY--A-YGN-V-VSVQN-RGAV-----
LACBN01277	----------SIMGPDLAQNMYDSSINF-GA--EY--V-YGT-V-SAVED-HGDY-----
LACBA00605	----------SVLGPDLAKDMYEGSTQF-GA--EY--A-YGS-V-TAIED-HGDH-----
LACPL00650	----------SVLGPDLAKDMYESATQF-GA--EY--A-YGS-V-ESVED-RGDV-----
LACPJ00628	----------SVLGPDLAKDMYESATQF-GA--EY--A-YGS-V-ESVED-RGDV-----
LACPS00585	----------SVLGPDLAKDMYESATQF-GA--EY--A-YGS-V-ESVED-RGDV-----
PEDCP00497	----------SILGPDLAKEMYDSSTQF-GA--EY--S-YGS-V-ESVVN-DGDT-----
CARS100359	----------SIAGPELSEKMFEGSKQF-GV--EY--A-YAD-V-KEIQQ-GTEY-----
AERUA00263	----------SIAGPDLANKMYESAMQF-GA--EY--V-FGN-V-KKVTP-GKPY-----
ELUMP00590	----------PVSGYELMDKMHKQAERL-GA--KV--V-HTE-I-SSIDT-SSKP-----
SPHPG02940	----------PVSGYDLGIKFQSQAEQF-GA--KI--V-YGS-V-TSLKK-EKDV-----
SPHGB01868	----------PISGYEIGMKFHAQAEAF-GA--KL--V-YAT-V-SKLSK-KGEI-----
TREPZ00273	----------PRSGFDFAEDMHRQAENF-GA--KI--L-SDA-V-LSLKN-EKNS-----
TREAZ03414	----------GKTGFDFSDDLHRQAVSF-GA--QF--L-TEQ-V-LSIKK-EGDI-----
SPITD00734	----------PISGFELAQRFETQARNF-GA--SF--L-NAT-V-KRISK-KEKV-----
SPITZ01373	----------PISGFELAQRFETQARNF-GA--SF--L-NAT-V-KRISK-KEKV-----
TREPA00803	----------PISGFEYAENMKKQAVAF-GA--QI--A-YEE-V-TTIGK-RDSV-----
TREPS00802	----------PISGFEYAENMKKQAVAF-GA--QI--A-YEE-V-TTIGK-RDSV-----
TREPC00747	----------PISGFEYAENMKKQAVAF-GA--QI--A-YEE-V-TTIGK-RDSV-----
TREPM00823	----------PISGFEYAENMKKQAVAF-GA--QI--A-YEE-V-TTIGK-RDSV-----
TREPD00824	----------PISGFEYAENMKKQAVAF-GA--QI--A-YEE-V-TTIGK-RDSV-----
TREPU00781	----------PISGFEYAENMKKQAVAF-GA--QI--A-YEE-V-TTIGK-RDSV-----

```
ENCCU00216      ----------GVEGPVLTDLMKDHAVSR-GL--RV--V-KET-V-SDLRK-EGEC-----
HELM100964      ----------ILSGLDFMQPWQEQCFRF-GL--KH--E-MVE-V-ERIEK-RGNH-----
HELCP01490      ----------IVSGMDFMEPWQEQCFRF-GL--KH--K-MAE-V-KQITK-ENGI-----
ARCFU01526      ------------SGMELLEKMKEQAVKA-GA--EW--K-LEK-V-ERVER-NGET-----
FERPA02445      ------------SGFELLERIKRQALKF-GA--VH--K-FEH-V-ERLRK-DGDY-----
ARCVS01910      ------------SGYELLEKMKEQATKF-A---EH--R-FES-V-EELKK-DGDI-----
METEZ00677      -----------INGMELMNTFKAHAESI-GV--PI--E-NKG-V-TGVRP-EDDK-----
METHD00869      -----------ISGPELMQRFKEHAQTV-GV--KI--E-SAE-V-SSIIS-EDGR-----
METMA02304      -----------ISGLELMERFRTHAQEV-GV--KT--T-ITE-V-LSVRS-EGTK-----
METAC01311      -----------ISGLELMEKYRTHAQEV-GV--KT--K-ITE-V-LSVRT-EGAK-----
KOSOT00298      -----------ISGMDLAEKMAEHARAF-DV--SF--L-NAE-V-VELEV-EGEK-----
MARPK01613      -----------ITGEELATKLGEHAREF-GV--EF--Y-DGE-V-INVDF-SKDE-----
SLAHD02439      --------DTEESGPSLIARMSKQVEHF-GC--NR--V-ADT-V-TAVEL-QGDV-----
FILAD00976      ----------EESGESLTARMVEQCKTF-GL--EI--V-QDT-V-LKVQL-EGAV-----
BUTPB02463      -----------VNGFDLGMKFREHADKQ-GA--EF--V-NAT-V-TAVECVEKGS---DT
CLOPH00250      -----------IGGFDLAVKFREHSDKL-GA--TH--V-YGE-V-KAFEVEDG-------
CLOSW00592      -----------INGYDLGMKFREHAEKL-GA--EF--S-TDE-V-LRIEASNG-------
LACFC00207      -----------VQGPELAQNMYDGATQF-GA--TY--G-YGT-V-TALTVNEDGT-----
CRYCD00853      ----------GIGGFELSMAMKEQDDGF-GV--VY--R-NEA-V-SAVDF-TGDV-----
EGGLE01847      ----------GTGGFELAFAMKQQADRF-GA--SN--V-GEE-V-VSVDF-TQNP-----
PYRFU01410      ----------GISGAELSKRMYEQVRKY-GV--DV--I-FDE-V-VRIDPAECAY---YE
PYRHO01476      ----------GISGSELSKKMYDQVKKY-GV--EV--I-IDE-V-IRIDPAECAY---YE
PYRAB00730      ----------GISGSELAKRMYEHVKKY-GV--DV--I-FDE-V-VRIDPAECAY---YE
PYRSN00014      ----------GISGSELSKRMYEQVRKY-GV--DV--I-IDE-V-VRIDPAECAY---YE
THEGJ00181      ----------GISGSELTARMHEHVKRL-GV--DI--V-FDE-V-ERIDPAECAY---YE
THEKO02097      ----------GISGSELTNRMHEQVKKL-GV--DV--I-FDE-V-VRIDPAECAY---YE
THEON01610      ----------GISGSELTTRMHEHVKKL-GV--DI--V-FDE-V-ERIDPTECAY---YE
THES401476      ----------GISGSELTNRMHEHVKRL-GV--DI--V-FDE-V-ERIDPAECAY---YE
SYNWW02368      ----------GVSGADLMENFRLQAERF-GA--EL--R-MEE-VMDLKNA-SGAI-----
UNCTG00012      ----------GINGFELAVKLEKQARDF-GA--EI--I-YDE-V-GAIEQ-GLSK-----
THEA101460      ----------GITGFELSEKMRQHAEKF-GA--KI--Q-SGA-PVTSVDF-DGEL-----
DENA201647      ----------GIMGADLSEKFYQHAVKY-GA--LV--R-SGN-C-TNIEK-DGKY-----
DEFDS00488      ----------GISGGELTEKLFQHAKQF-GV--EI--K-NGM-C-KGIEF-CDGY-----
CALNY01292      ----------GISGGDLTEQMYKHAIHF-GV--QV--K-NGE-C-CGVEF-EGDY-----
SYNGF01652      ----------GISGYELMNTFHRQALNQ-GA--EF--L-FEA-A-TGFDF-TGDT-----
DESB201123      ----------GVSGFDLADKMRDHALKF-GL--EI--V-SRE-I-SGLSA-EG-------
DEIPM00845      ----------AISGMELSQRMVQQAEKF-GA--VI--E-MDE-V-QSIQH-DAG-----G
DEIRA01924      ----------PIAGMELAQRMHQQAEKF-GA--KV--E-MDE-V-QGVQH-DAT-----S
DEIML01437      ----------PIHGMELAQRMVQQAEKF-GA--VL--D-MDE-V-QSISH-DISS-----
DEIGD02532      ----------PISGMELAQRMVQQAEKF-GA--RI--E-MDE-V-EAITH-ADDD---RE
DEIDV00622      ----------PIPGMELASRMQQQAEKF-GA--RI--E-MEE-V-QGITR-NE-----HD
TRURR01079      ----------VISGAELSQRMVRQAEKF-GA--KI--L-MEE-V-QRVEP-QD-------
THETG01863      ----------GISGPELASRMVQQAEKF-GA--RI--V-MDE-V-LGLEK-AE-------
THET201543      ----------GISGPELASRMVRQAEKF-GA--RI--V-MDE-V-LGLEK-AE-------
THET801911      ----------GISGPELASRMVRQAEKF-GA--RI--V-MDE-V-LGLEK-AE-------
OCEP502115      ----------GISGPELANRMAEQARKF-GA--EV--V-MDE-V-QALEK-TD-------
DEHLB00585      ----------VINGYDLTQRMHEQTNKF-GV--EH--I-QTG-V-NGIED-KGSY-----
DEHMG00439      ----------GITGFDLTQQMQIQAEKY-GV--EI--T-SAE-V-NAIHK-TKDH-----
DEHMB00497      ----------GITGFDLTQQMQIQAEKY-GV--EI--T-SAE-V-NAIHK-TKDH-----
DEHMC00427      ----------GITGFDLTQQMQIQAEKY-GV--EI--T-SAE-V-NAIHK-TKDH-----
THELD00726      -----------ASGPELAESFRKHAEHF-GP--EF--R-EAE-V-QKIEI-KDGS-----
ANAMD00624      -----------ISGEELARAFREHAERF-SP--EF--R-EAE-V-KKISI-DAGK-----
THEAS00815      -----------ASGQELADSFRRHAEKF-SP--EF--R-DAT-V-TSLEV-RDGR-----
AMICL00707      -----------ASGQELGDMFRVHAEKF-NT--EF--R-DAD-V-KKIEL-RDGS-----
CLOCE01684      ----------PIGGPDLALRMEKQARKF-GT--VV--L-NDE-V-LELEL-DSPI-----
HYDS000616      ----------GISGKELTSKFKAQAERF-GL--KI--H-RQG-V-ISVEN-DNDN-----
HYDTT00224      ----------GISGKELSLRFKQQAERF-GL--KV--A-KAE-A-TKIEK-SGKE-----
PELTS01405      ----------GIGGFELMQKMEEQARRF-GL--EI--V-SVN-V-ESIRV-DGKG-----
DESRL02179      ----------GVGGVDLAMKMDEQARSF-NL--DV--E-FAD-V-ERLEQ-REGG-----
BORBP00506      ----------GISGRDLMLNMREQVVNL-GA--K---TFPET-V-FSIKRENS-------
BORAP00518      ----------GISGRDLMLNMREQVVNL-GA--K---TFLET-V-FSIKKRNS-------
BORBU00514      ----------GISGRNLMLNMREQVVNL-GA--K---TFPET-V-FSIKRKGN-------
BORBZ00490      ----------GISGRNLMLNMREQVVNL-GA--K---TFPET-V-FSIKRKGN-------
BORBN00493      ----------GISGRNLMLNMREQVVNL-GA--K---TFPET-V-FSIKRKGN-------
BORRA00486      ----------GINGRELMLNMKEQAINL-GA--T---TYFET-V-RSIKKRGD-------
```

```
BORDL00498       ----------GINGRELMLNMKEQAINL-GA--T---TYFET-V-RSIKKRGD-------
BORHD00497       ----------GVNGRELMLNMKEQVINL-GA--T---TYLET-V-RFIEKRDN-------
BORT900497       ----------GVNGRELMLNMKDQVINL-GA--I---TYLET-V-TSIEKRDN-------
METKA01560       ----------PAPGYELVDRMVEHAETV-GV--ELNVKYSNR-V-EGIELTDG-------
MYCA500361       ----------NITGPSLATHFYNQATRN-GA--SL--IFGKA-I-KIISN-GD-------
METVS00145       ----------SIKGFELAEQFSKHAEYF-EL--PI--M-HEE-V-IGIDTKSK-------
METOI01431       ----------SISGFELSQKFVEHAKKF-NL--NI--V-HDT-I-KKID-ISK-------
MYCHN00051       ----------KLNGSTLGESMLKQVLDN-GV--KQ--Y-FGT-V-IDVEKEEK-------
MYCSL00465       ----------DRSGFQLSDNLLTQLKGL-NV--EI--K-TEE-V-LNIVESQN-------
MYCS300420       ----------DRSGFQLSDNLFTQLKGL-NV--EI--K-TEE-V-LNIVESQN-------
DESK101258       ----------DIPGNDLVDRFVKHVKKY-NV--PI--VIDEV-I-NLVKKPEE-------
STAHD01472       ----------EIPGNELVDRFAKHVRKY-NV--PI--IIDEV-I-NMTRK--N-------
THEC100270       ----------DIPGEELVNKFVNHVKKY-NV--PI--IQDEV-V-DMYRK--D-------
NANEQ00478       ----------SISGPDLAERLFEQYEKNGGH------YLKDK-V-SD-IKKDK-------
CALLD01225       ----------SIKASELVSRFRDHAEKLFKV--PV--YEFIS-V-KKFDKVDD-------
SULSO02155       ----------EIQASDMIKVFNKHIEKY-EV--PV--LLD-I-V-EKIENRGD-------
SULS900210       ----------EIQASDMIKVFNKHIEKY-EV--PV--LLD-I-V-EKIENRGD-------
SULIA00208       ----------EIQASDMIKVFNKHIEKY-EV--PV--LLD-I-V-EKIENRGD-------
SULIM00207       ----------EIQASDMIKVFNKHIEKY-EV--PV--LLD-I-V-EKIENRGD-------
SULIK00225       ----------EIQASDMIKVFNKHIEKY-EV--PV--LLD-I-V-EKIENRGD-------
IGNH400907       ----------KIGGEELAQRFLNQATKF-GA--QV--VFGER-V-VDADF---------S
KORCO01040       ----------SISGEELGRRMHEHALKS-GA--RI--LSPEE-V-VKLELTGE-------
THESM01133       ----------GVKGSELANKMHEQVKKL-NV--P---IVFDE-V-ERVDPAECAY---YE
THEBM01534       ----------GISGSELNRRMYDQVRKY-NV--D---VIFDE-V-ERIEKGECPY---YE
METST01363       ----------EIPGVDLARSMTEQAKKY-VD--I---REFSL-V-ESISKSIG-------
METTH00703       ----------MIAGMSLVTKMKKQATAV-AE--L---REMEE-V-KEIEK-GD-------
METSL02406       ----------SIPGMELLKKIAEQAEKY-TE--I---KENEE-I-KKIEK-ID-------
METLA02406       ----------SIPGMELLKKIAEQAEKY-TE--I---KENEE-I-KKIEK-ID-------
METPW00194       ----------SITGLELLKRIGDHASGY-MD--I---KESEE-I-QKVEK-MD-------
RUBXD00226       ----------GVTGPEMMEDFERQAARF-GA--EM--R-PDN-V-DRVDF----S-G---
RHOM400178       ----------GILGPELMQRFEQQAARF-GA--DL--R-YGT-V-TAVDF----S-R---
GARV400051       ----------GILGPDLMESMQKQAEKF-GA--EV--L-LNE-V-ASVDF----K-S---
BIFAP01644       ----------AVLGPDLMDQMREQAEHF-GA--QI--E-YDD-V-VSVDL----S-G---
SEGRD00014       ----------GVQGPDLMVEMRSQAERF-GA--RL--Q-TKD-V-EWVEL----G-G---
GORB404535       ----------GIQGPALMDEMREQALRF-GA--DL--R-MED-V-DAVRL----T-G---
GORPV04920       ----------GIQGPALMDEMREQAIRF-GA--DL--R-MED-V-DELRL----D-G---
MYCA904903       ----------GIMGPDLMDQMREQAIRF-GA--DL--R-TED-V-DEVSL----R-G---
MYCSS05356       ----------GITGPELMDEMREQALRF-GA--DL--R-MED-V-DEVSL----D-G---
MYCSJ05684       ----------GITGPELMDEMREQALRF-GA--DL--R-MED-V-DEVSL----D-G---
MYCSK05387       ----------GITGPELMDEMREQALRF-GA--DL--R-MED-V-DEVSL----D-G---
MYCS206581       ----------GITGPELMDQMREQALRF-GA--DL--R-MED-V-DAVQL----E-G---
MYCCN05158       ----------GITGPELMDQMREQALRF-GA--DL--H-MED-V-DAVDL----A-G---
MYCVP05890       ----------GITGPELMDEMREQALRF-GA--DL--R-MED-V-DAVDL----T-G---
MYCGI00775       ----------GITGPELMDEMREQALRF-GA--DL--R-MED-V-DAVDL----T-G---
MYCSR04994       ----------GITGPELMDEMREQALRF-GA--DL--R-MED-V-DAVDL----T-G---
AMYSD04545       ----------GIMGPALMDEMREQALRF-GT--DI--R-TED-V-ESVTL----D-G---
MYCLE02681       ----------GITGPELMDDMREQALRF-GA--EL--R-TED-V-ESVSL----R-G---
MYCLB02681       ----------GITGPELMDDMREQALRF-GA--EL--R-TED-V-ESVSL----R-G---
MYCSD04323       ----------GIMGPELMDEMREQALRF-GA--DL--R-MED-V-ESVAL----D-G---
MYCPA04306       ----------GITGPELMDQMREQALRF-GA--DL--R-MED-V-ESVSL----A-G---
MYCA105023       ----------GITGPELMDQMREQALRF-GA--DL--R-MED-V-ESVSL----A-G---
MYCUA04107       ----------GITGPELMDEMREQALRF-GA--DL--R-MED-V-QSVSL----D-G---
MYCMM05378       ----------GITGPELMDEMREQALRF-GA--DL--R-MED-V-ESVSL----D-G---
MYCA003905       ----------GITGPELMDEMREQALRF-GA--DL--R-MED-V-ESASL----H-G---
MYCTU03943       ----------GITGPELMDEMREQALRF-GA--DL--R-MED-V-ESVSL----H-G---
MYCTF03846       ----------GITGPELMDEMREQALRF-GA--DL--R-MED-V-ESVSL----H-G---
MYCTA03980       ----------GITGPELMDEMREQALRF-GA--DL--R-MED-V-ESVSL----H-G---
MYCTK04010       ----------GITGPELMDEMREQALRF-GA--DL--R-MED-V-ESVSL----H-G---
MYCTC03612       ----------GITGPELMDEMREQALRF-GA--DL--R-MED-V-ESVSL----H-G---
MYCTD03548       ----------GITGPELMDEMREQALRF-GA--DL--R-MED-V-ESVSL----H-G---
MYCCP03898       ----------GITGPELMDEMREQALRF-GA--DL--R-MED-V-ESVSL----H-G---
MYCBO02863       ----------GITGPELMDEMREQALRF-GA--DL--R-MED-V-ESVSL----H-G---
MYCBP03913       ----------GITGPELMDEMREQALRF-GA--DL--R-MED-V-ESVSL----H-G---
MYCBT03913       ----------GITGPELMDEMREQALRF-GA--DL--R-MED-V-ESVSL----H-G---
TSUPD04078       ----------GIMGTELMDEMREQAIRF-GA--DL--R-MED-V-ESARL----D-G---
```

ACTMD06897	----------GIMGPDLMEQMREQAKRF-GA--EL--R-AED-V-DAVEL----E-G---
SACES08402	----------GIMGPDLMEQMREQAKRF-GA--EL--R-AED-V-ESVEL----A-G---
SACVD03814	----------GIQGPDLMQEMREQAKVF-GA--DL--R-QED-V-EELEL----T-G---
AMYMU09190	----------GIMGPDLMEEMRKQAERF-GA--EL--R-AED-V-ESLEL----T-G---
AMYMS10178	----------GIMGPDLMEEMRKQAERF-GA--EL--R-AED-V-ESLEL----T-G---
PSEUX06421	----------GIMGPELMEQMRNQAKRF-GA--EL--R-SED-V-DSVDL----T-G---
NOCFA05681	----------GIMGPDLMEEMREQAKRF-GA--EI--R-TED-V-DALDL----T-G---
NOCCG05476	----------GIMGPDLMEQMRDQAKRF-GA--DI--R-TED-V-DAIDL----S-G---
RHOE406010	----------GIMGPDLMEQMREQALRF-DT--DI--R-TED-V-EEIDL----S-G---
RHOOB03468	----------GIMGPDLMDQMREQAKRF-GT--DI--R-TED-V-EELDL----T-G---
RHOE104515	----------GIMGPDLMDEMREQAKRF-GA--DI--R-TED-V-EEIEL----D-G---
CORDI02302	----------GIMGPELMQNMREQAEKF-GA--EL--R-MEL-V-TKVEL----E-G---
CORD202219	----------GIMGPELMQNMREQAEKF-GA--EL--R-MEL-V-TKVEL----E-G---
CORDL02208	----------GIMGPELMQNMREQAEKF-GA--EL--R-MEL-V-TKVEL----E-G---
CORDJ02208	----------GIMGPELMQNMREQAEKF-GA--EL--R-MEL-V-TKVEL----E-G---
CORDH02221	----------GIMGPELMQNMREQAEKF-GA--EL--R-MEL-V-TKVEL----E-G---
CORD702316	----------GIMGPELMQNMREQAEKF-GA--DL--R-MEL-V-TKVEL----E-G---
CORD302337	----------GIMGPELMQNMREQAEKF-GA--DL--R-MEL-V-TKVEL----E-G---
CORDD02235	----------GIMGPELMQNMREQAEKF-GA--DL--R-MEL-V-TKVEL----E-G---
CORDV02170	----------GIMGPELMQNMREQAEKF-GA--DL--R-MEL-V-TKVEL----E-G---
CORDW02254	----------GIMGPELMQNMREQAEKF-GA--DL--R-MEL-V-TKVEL----E-G---
CORDK02230	----------GIMGPELMQNMREQAEKF-GA--DL--R-MEL-V-TKVEL----E-G---
COREF02870	----------GIMGPELMENMRAQAERF-GT--DL--R-MEL-V-DRVDL----T-G---
CORGL03082	----------GILGPELMENMRAQAERF-GT--DM--H-MEL-V-DRVDL----T-G---
CORGK02974	----------GILGPELMENMRAQAERF-GT--DM--H-MEL-V-DRVDL----T-G---
CORGB03038	----------GILGPELMENMRAQAERF-GT--DM--H-MEL-V-DRVDL----T-G---
CORK402006	----------GIMGPSLMEEMRQQAERF-GA--EL--K-QDM-V-ESMDL----T-G---
CORJK02028	----------GIMGPDLMEEMRAQAERF-GA--DL--R-QED-V-EKVDL----T-G---
CORVD02951	----------GIMGPDLMENMRNQAERF-GA--DL--R-MED-V-VRVEL----D-G---
ARCHD01706	----------PILGPELMMRMQQQAERF-GA--QV--R-YED-A-ISCDL----A-S---
THET101205	----------GVQGPELMALFQQQAERF-GA--EI--V-PVD-V-TKVDF----S-S---
PROAC02247	----------GIMGPDLMAQMRAQAERF-GA--EL--I-ADD-V-TDIDL----T-G---
PROAS02302	----------GIMGPDLMAQMRAQAERF-GA--EL--I-ADD-V-TDIDL----T-G---
CAERE29798	----------GVQGPELMESMRAQAERF-GA--RI--V-YDD-G-TRLEL----D-G---
CELFA03749	----------GIQGPELMDALQKQAEKF-GA--EV--L-WDD-A-VSLSL----E-G---
JONDD02484	----------AIMGPALMENMQKQAETF-GA--TI--E-WDD-A-ETLEL----T-S---
XYLCX03324	----------AVMGPDLMDKMREQAEKF-GA--TV--V-WDD-A-ERVSL----S-G---
ACIC102144	----------GIQGPDLMERMRAQAAKF-GA--EL--V-PHD-V-VAVDL----R-E---
FRADG04042	----------GVQGPDLMDGIRRQAERF-GA--EL--L-AED-V-TAVDL----R-A---
FRASU07065	----------GIQGPDLMDNLRKQAERF-GA--EL--V-TDD-V-TEVDL----T-A---
FRASN07115	----------GIQGPELMENLRAQAERF-GA--EL--I-ADD-V-TEVDL----A-A---
FRASC04424	----------GIQGPELMENLRRQAERF-GA--EL--V-ADD-V-TELDL----A-A---
FRAAA06712	----------GVQGPELMEKLRQQAERF-GA--EL--V-ADD-V-TELDL----T-A---
KYTSD02521	----------GIMGPDLMMQMRSQAERF-GA--EL--V-TDD-I-VEMDL----T-A---
KINRD04462	----------GILGPDLMDNMRQQAERF-GA--EL--I-TDD-V-VDVRL----E-G---
NOCDD04789	----------GVMGPDLMDHMRKQAERF-GA--EL--V-PED-V-TEVDL----T-K---
NOCAA01941	----------AIMGPDLMDSMRKQAERF-GA--EL--V-PED-V-TSVDL----T-K---
STRRD08913	----------GIMGPDLMDNMRKQAERF-GA--EL--I-ADD-V-VEVDL----T-V---
THECD04863	----------GIMGPDLMDNMRKQAERF-GA--DL--I-ADD-V-TEVDL----T-V---
THEBD03528	----------GIMGPELMDNMRKQAERF-GA--EL--V-ADD-V-VEVDL----L-A---
CATAD08894	----------GIMGPDLMDNMQKQAERF-GA--EL--V-FDD-V-TAVDL----S-G---
KRIFD06917	----------GIMGPALMDEMRTQAERF-GA--EL--V-ADD-V-VEVDL----T-G---
KITSK03730	----------GIMGPELMDNMRAQAERF-GA--EL--V-PDD-V-IAVDL----T-G---
STRBB05366	----------GIMGPDLMDNMRAQAERF-GA--EL--V-PDD-V-LSVDL----T-E---
STRVP03660	----------GIMGPDLMDNMRAQAERF-GA--EL--V-PDD-V-IAVDL----T-G---
STRSW04381	----------GIMGPDLMDNMRGQAERF-GA--EL--I-PDD-I-VAVDL----S-G---
STRGG03562	----------GIMGPELMDNMRAQAERF-GA--EL--I-PDD-V-VAVDL----T-G---
STRFA03098	----------GIMGPELMDNMRAQAERF-GA--EL--I-PDD-V-VSVDL----T-G---
STRAW04303	----------GIMGPELMDNMRAQAERF-GA--EL--V-PDD-V-VAVDL----T-G---
STRCO02929	----------GIMGPELMDNMRAQAERF-GA--EL--I-PDD-V-VAVDL----S-G---
STRHJ05105	----------GIMGPELMDNMRAQAERF-GA--EL--I-PDD-I-VSVDL----T-G---
MONBE04991	----------GVMGQEITDRFRAQSARF-GT--EI--F-TET-V-SDVDL----S-A---
CHLRE01313	----------GILGAELTTRFREQSERF-GT--RI--Y-SET-V-DSIDT----S-R---
MEDTR25591	----------GILGGELMERCRQQSAKF-GT--EI--F-TET-V-SKVDF----S-T---
SOLLC13750	----------GIGGGELMEKCRAQSVRF-GT--QI--Y-TET-V-TKVDF----S-K---

```
PRUPE10733      ----------GIMGGELMDRCRNQSLRF-GT--EI--F-TET-V-NKVDF----S-S---
MANES18605      ----------GIQGGELMDRCRAQSVRF-GT--QI--Y-TET-V-NKVDF----S-S---
THECC00884      ----------GIMGMELMDRCRNQSLRF-GT--AI--Y-TET-V-NKVDL----S-S---
PHYPA31147      ----------GILGSELTDKFRAQSIRF-GT--RV--F-SET-V-NRVDF----S-S---
AMBTC19471      ----------GISGLDLTDKFRAQSVRF-GT--TI--F-TET-I-EKMDL----E-T---
MUSAC26038      ----------GIMGYELMDRCRAQSLRF-GT--EI--L-SET-V-TTVDL----A-V---
MUSAM33177      ----------GIMGYELMDRCRAQSLRF-GT--EI--L-SET-V-TTVDL----A-V---
SETIT03079      ----------GIMGADLMDRCRAQSARF-GT--NI--L-SET-V-TAVDF----A-A---
ORYBR12195      ----------GILGADLMDRCRAQSVRF-GT--RI--F-SET-V-TAVDF----S-S---
COCLU07729      ----------KIGGGKLMEQMREQSEAC-GT--EI--I-SQT-V-AKVDL----K-S---
PHANO13702      ----------KIGGGKLMEHMREQSEAC-GT--EI--I-SQT-V-AKVDL----K-S---
PHAND10804      ----------KIGGGKLMEHMREQSEAC-GT--EI--I-SQT-V-AKVDL----K-S---
AURPU02089      ----------NIQGATLMDNMRAQSEAC-GT--QI--V-TQT-V-GKVDL----S-Q---
ZYMTR07711      ----------MIKGTTLMDNMRAQSEEC-GT--EI--V-SQT-V-GKVDL----S-S---
DICPU05926      ----------DISGQELMDKMREQNEKC-GT--RI--E-TKT-I-AKVDL----K-S---
ENTHI00522      ----------GIDGNELMMNMRTQSEKY-GT--TI--I-TET-I-DHVDF----S-T---
LEPBA02231      ----------GIDGTKLTQLFREQSAKY-GT--TI--H-TQT-I-TKVDF----S-K---
LEPBP02301      ----------GIDGTKLTQLFREQSAKY-GT--TI--H-TQT-I-TKVDF----S-K---
LEPBL01462      ----------GIDGTQLTQLFREQSVKY-GT--KI--L-TQT-I-TKVDF----S-S---
LEPBJ01276      ----------GIDGTQLTQLFREQSVKY-GT--KI--L-TQT-I-TKVDF----S-S---
LEPIN02475      ----------GIDGTKLTQLFREQSIKY-GT--KI--I-TQT-I-TKVDF----S-S---
LEPII01996      ----------GIDGTKLTQLFREQSIKY-GT--KI--I-TQT-I-TKVDF----S-S---
LEPIC01426      ----------GIDGTKLTQLFREQSIKY-GT--KI--I-TQT-I-TKVDF----S-S---
SPIAZ00697      ----------GISGPALMDQMRAQSLRF-GT--EI--K-TET-V-SAVDM----S-K---
PENRW10140      ----------GIGGAELMDNMRAQSERF-GT--EI--I-TET-I-SKLDL----S-S---
PENCH09104      ----------GIGGAELMDNMRAQSERF-GT--EI--I-TET-I-SKLDL----S-S---
EURHE07269      ----------GIGGTELMEAMRKQSIRF-GT--EV--I-TET-I-SKLDL----S-Q---
ASPAC07301      ----------GIGGGELMENMRKQSIRF-GT--EV--I-TET-I-SKVDF----S-Q---
EMENI10387      ----------GIGGSELMDAMRKQSIRF-GT--EV--I-TET-I-SRVDL----S-Q---
EMEND02596      ----------GIGGSELMDAMRKQSIRF-GT--EV--I-TET-I-SRVDL----S-Q---
ASPTN06742      ----------GIGGGELMDNMRKQSIRF-GT--EV--I-TET-I-SRVDL----S-Q---
ASPCL04014      ----------GIGGAELMENMRKQSIRF-GT--EV--I-TET-I-SRIDL----S-S---
ASPFU05647      ----------GIGGAELMENMRKQSIRF-GT--EV--I-TET-I-SRVDL----S-S---
NEOFI00452      ----------GIGGAELMENMRKQSIRF-GT--EV--I-TET-I-SRVDL----S-S---
CRYPA10563      ----------GIMGGALMDNMRKQSERF-GT--KI--I-SET-I-ADLDL----S-S---
BLUGR03498      ----------GITGPALMDAMRNQSERF-GT--RI--I-TET-V-SRVDL----S-Q---
SCLS112814      ----------GIGGQELMDNMRAQSERF-GT--QI--I-TET-V-AKVDL----S-K---
MAGGR04266      ----------GIGGQELMDAMREQSSRF-ET--EI--I-SET-V-AKVDL----S-S---
NEUCR01575      ----------GIMGQELMDKMKAQSERF-GT--QI--I-SET-V-AKVDL----S-A---
NEUT908941      ----------GIMGQELMDKMKAQSERF-GT--QI--I-SET-V-AKVDL----S-A---
VERDA02342      ----------GIMGQELMDKMRAQSVRF-GT--EI--V-SET-V-ATLDL----S-Q---
COLSU12486      ----------GIMGGELMENMKAQSARF-GT--EI--I-TDT-V-SKLDL----S-S---
HYPAI01684      ----------GIMGQELMDSMRAQSERF-GT--EI--V-TDT-V-TKLDL----S-Q---
HYPVG06080      ----------GIMGQELMDNMRAQSERF-GT--EI--V-TDT-V-TKLDL----S-Q---
HYPJE05895      ----------GIMGQELMDNMRAQSERF-GT--EI--I-TDT-V-TTLDL----S-S---
NECHA05020      ----------GIMGQELMDNMRAQSERF-GT--EI--V-TDT-V-ATLDL----S-N---
FUSO415847      ----------GIMGGELMDNMRAQSERF-GT--EI--V-TDT-V-TTLDL----S-S---
GIBZA01026      ----------GIMGGELMDNMRAQSERF-GT--EI--I-TDT-V-ATLDL----S-S---
SCHPO04025      ----------GINGTTLTENFRAQSLRF-GT--EI--I-TET-V-SKLDL----S-S---
YARLI03635      ----------GIMGSQLMEDMRKQSIRF-GT--EI--I-TET-V-SKVDL----S-Q---
ASHGO00946      ----------GLTGSDLMERMKAQSVKF-GT--EV--V-TET-V-AKVDL----S-A---
KLULA02190      ----------GLTGSELMDRMKAQSIKF-GT--DV--I-TET-V-SKVDL----S-S---
ZYGRO00676      ----------GLLGSELMDRMKEQSVKF-GT--EV--I-TET-I-SKVDL----S-S---
DEKBR01813      ----------GIMGSDLMDNMKKQSEKF-GT--KI--I-TET-I-SKVDF----S-S---
PICPG04776      ----------GISGTGLMDKLREQSLRF-GT--EI--I-TET-I-SKVDL----S-S---
CANTE00916      ----------GINGTSLMDNMRKQSERF-GT--KI--I-TET-I-SKVDF----S-S---
LODEL03891      ----------GISGTTLMERMREQSERF-GT--EI--I-TET-I-SKCDL----S-Q---
DEBHA05546      ----------GINGTELMDQMREQSVRF-GT--DI--I-TET-I-SKCDL----S-S---
SPAPN03477      ----------GINGTELMENMKKQSQRF-GT--DI--I-TET-I-SKVDL----S-A---
CANAW04800      ----------GIGGSELMEKMKEQSQRF-GT--EI--I-TET-I-SKVDF----S-K---
PICST04701      ----------GINGTTLMEQMKEQSERF-GT--EI--I-TET-I-SKVDF----S-A---
PUCGT10887      ----------GIRGPEMMDLFRAQSIRF-GT--KI--L-TET-V-SKIDL----S-N---
PUCGR11813      ----------GIRGPEMMDLFRAQSIRF-GT--KI--L-TET-V-SKIDL----S-N---
PHYBL11006      ----------GVMGGELMEKMREQSVRF-GT--EI--E-TET-I-TKLDT----S-A---
USTMA03757      ----------GIRGPEIMDKFRAQSVRF-GT--EI--H-TET-I-AKLDL----S-S---
```

```
USTHO04132      ----------GIRGPEIMDKFRAQSVRF-GT--EI--H-TET-I-SRVDL----S-S---
WALSE04527      ----------GIRGPEMMDKFRAQSERF-GT--TI--H-TET-I-SKVDL----S-K---
TREME07701      ----------GVRGPEMMDKFRAQSERF-GT--NI--I-TET-I-ARVDF----T-K---
AURST04751      ----------GVRGPEMMDKFRAQSMRF-GT--TI--I-TET-V-SRIDL----S-A---
FOMME10177      ----------GIMGPDLMDKFRAQSARF-GT--EI--I-TET-V-SKIDL----S-R---
CONPW06392      ----------GILGPELMQKFREQSIRF-GT--RV--I-TET-V-SKIDL----S-Q---
STEHR07076      ----------GVLGSELMDKFRAQSLRF-GT--NI--I-TET-V-SKIDL----S-K---
HETAN06295      ----------GVLGPELMDKFRAQSLRF-GT--RI--I-TET-V-SKIDL----S-R---
GLOTR06982      ----------GIMGPELMDKFRAQSLRF-GT--NI--I-TET-V-SKIDL----S-A---
PUNST01981      ----------GILGPELMDKFRAQSLRF-GT--QI--I-TET-I-TKLDL----S-Q---
LACBI02877      ----------GILGPELMDKFREQSVRF-GT--TI--I-TET-V-SKIDL----S-S---
COPCI16429      ----------GILGPELMDKFREQSLRF-GT--KI--I-TET-V-SKIDL----S-A---
DICSQ11618      ----------GILGPELMDKFRAQSLRF-GT--QI--I-TET-V-AKVDL----S-A---
TRAVS13180      ----------GILGPELMDKFRAQSLRF-GT--KI--I-TET-V-SKVDL----S-A---
WOLCO03584      ----------GILGPELMDKFRAQSQRF-GT--NI--I-TET-V-SKVDL----S-A---
FOMPI05979      ----------GILGPELMDKFRAQSLRF-GT--QI--I-TET-V-SKVDL----S-A---
PHLGI10219      ----------GILGPELMDKFRAQSLRF-GT--RI--I-TET-I-SKVDL----S-A---
PHACH05757      ----------GILGPELMDKFRAQSLRF-GT--KI--I-TET-V-SKVDL----S-A---
RICTY00419      ----------TIQGPWLMEQMSIQAKNV-GT--EI--I-NDY-V-ERVDL----S-K---
RICPR00429      ----------TIQGPWLMEQMSMQAKNV-GT--EI--I-SDY-V-ERVDL----S-K---
RICPP00461      ----------TIQGPWLMEQMSMQAKNV-GT--EI--I-SDY-V-ERVDL----S-K---
RICBR00434      ----------SVQGPWLMEQMRMQAENV-GT--KI--V-NDY-V-EKVDL----S-Q---
RICB800988      ----------SVQGPWLMEQMRMQAENV-GT--KI--V-NDY-V-EKVDL----S-Q---
RICCK00600      ----------TVQGPWLMEQMYMQAKNV-GT--EI--V-SDY-V-EKVDL----S-K---
RICAH00614      ----------TVQGPWLMEQMSMQAQNV-GT--EI--I-SDY-V-EKVDL----S-K---
RICAC00782      ----------TVPGSWLMEQMSMQAKNV-GT--EI--I-SDY-V-EKVDL----S-K---
RICFE00656      ----------TVQGPWLMEQMSMQAKNV-GT--EI--I-SDY-V-EKVDL----S-K---
RICMS00042      ----------TVQGPWLMEQMSMQAKNV-GT--EI--V-SDY-V-EKVDL----S-K---
RICM500453      ----------TVQGPWLMEQMSMQAKNV-GT--EI--V-SDY-V-EKVDL----S-K---
RICR300665      ----------TVQGPWLMEQMSMQAKNV-GT--EI--V-SDY-V-EKVDL----S-K---
RICAG00651      ----------TVQGPWLMEQMSMQAKNV-GT--EI--V-SDY-V-EKVDL----S-K---
RICP300630      ----------TVQGPWLMEQMYMQAKNV-GT--EI--V-SDY-V-EKVDL----S-K---
RICRS00632      ----------TVQGPWLMEQMYMQAKNV-GT--EI--V-SDY-V-EKVDL----S-K---
RICRO00663      ----------TVQGPWLMEQMYMQAKNV-GT--EI--V-SDY-V-EKVDL----S-K---
RICCN00618      ----------TVQGPWLMEQMYMQAKNV-GT--EI--V-SDY-V-EKVDL----S-K---
RICPT00620      ----------TVQGPWLMEQMYMQAKNV-GT--EI--V-SDY-V-EKVDL----S-K---
RICAE00497      ----------TVQGPWLMEQMYMQAKNV-GT--EI--V-SDY-V-EKVDL----S-K---
RICJY00467      ----------TVQGPWLMEQMSMQAKNV-GT--EI--V-SDY-V-EKVDL----S-K---
RICPU00072      ----------TVQGPWLMEQMSMQAKNV-GT--EI--V-SDY-V-EKVDL----S-K---
RICS100537      ----------TVQGPWLMEQMSMQAKNV-GT--EI--V-SDY-V-EKVDL----S-K---
BARBK00891      ----------PVQGAWLMEQMKKQAQHV-GT--EI--I-YDN-I-VKADL----S-K---
BARVW00932      ----------SIQGPWLMEQMAKQAENM-GT--KI--V-YDT-I-TKAEL----L-K---
BART100617      ----------PIQGPWLMEQMAKQAENM-GA--KI--V-YDT-I-IKADL----L-K---
BARGA00466      ----------PIQGPWLMEQMAKQAENM-GT--KI--V-YDT-I-IKADL----L-K---
BARHE01107      ----------PIQGPWLMEQMAKQAENM-GT--KI--V-YDN-I-TKADL----S-K---
BARQU00895      ----------PIQGPWLMEQMAKQAENM-GT--KV--V-YDS-I-IKADL----L-K---
OCHA401642      ----------PVQGPWMMEQMAKQAENV-GS--EI--V-HDI-I-TEVET----A-V---
BRUAB01390      ----------PVQGPWMMEQMARQAENV-GA--QI--V-HDI-I-TEVET----T-V---
BRUA201507      ----------PVQGPWMMEQMARQAENV-GA--QI--V-HDI-I-TEVET----T-V---
BRUA101339      ----------PVQGPWMMEQMARQAENV-GA--QI--V-HDI-I-TEVET----T-V---
BRUSU01458      ----------PVQGPWMMEQMARQAENV-GA--QI--V-HDI-I-TEVET----T-V---
BRUME00510      ----------PVQGPWMMEQMARQAENV-GA--QI--V-HDI-I-TEVET----T-V---
BRUSI01461      ----------PVQGPWMMEQMARQAENV-GA--QI--V-HDI-I-TEVET----T-V---
BRUC201446      ----------PVQGPWMMEQMARQAENV-GA--QI--V-HDI-I-TEVET----T-V---
BRUMC01440      ----------PVQGPWMMEQMARQAENV-GA--QI--V-HDI-I-TEVET----T-V---
BRUMB01421      ----------PVQGPWMMEQMARQAENV-GA--QI--V-HDI-I-TEVET----T-V---
BRUM501487      ----------PVQGPWMMEQMARQAENV-GA--QI--V-HDI-I-TEVET----T-V---
BRUO201285      ----------PVQGPWMMEQMARQAENV-GA--QI--V-HDI-I-TEVET----T-V---
RHILO01973      ----------PIQGPWLMEQMMKQAEHV-GT--DI--I-NDI-I-TEVDL----N-V---
CHESB02097      ----------PIQGPWLMEQMRLQAEHV-GT--EI--V-NDL-I-VEADI----R-S---
METPB00996      ----------AIQGPWLMEQMRLQAEHV-GT--KI--V-SEY-I-TKVDL----K-V---
METEP01063      ----------AIQGPWLMEQMRLQAEHV-GT--KI--V-SEY-I-TSVDL----N-V---
METEA00810      ----------AIQGPWLMEQMRLQAEHV-GT--KI--V-SEY-I-TSVDL----N-V---
METED01453      ----------AIQGPWLMEQMRLQAEHV-GT--KI--V-SEY-I-TSVDL----N-V---
METS403554      ----------AVQGPWLMEQMRLQAEHV-GT--RI--V-SEY-I-AKVDL----K-Q---
```

```
METNO05482      ----------AVQGPWLMEQMRLQAEHV-GT--RI--V-SEY-I-AKVDL----K-Q---
METSZ03234      ----------PIQGPWLMEQMRLQAEHV-GT--KL--V-SDH-I-VEAHL----D-E---
BEII900056      ----------PIQGPWLMEQMRTQAEHV-GT--KL--I-ADH-V-IKVEL----G-Q---
METSB02743      ----------PVQGPWLMEQMRAQAENV-GA--RM--I-SDH-I-VKVEL----D-R---
MAGMM00401      ----------GIQGPELMQRMQAQAERF-NT--EV--I-FDT-V-MHADL----S-K---
HYPNA00542      ----------HIQGPELMVKMQEHAEKM-GA--KV--A-NDH-I-ASVDF----E-T---
KETVY00902      ----------SILGPDLMVRMEDHAREM-GA--EI--V-MDL-I-TSLDL----Q-Q---
KETVW00472      ----------SILGPDLMVRMEDHAREM-GA--EI--V-MDL-I-TSLDL----Q-Q---
ROSDO03242      ----------EVQGPDLMIRMQDHAKAM-GT--EI--I-GDI-I-TDLNL----E-Q---
ROSLO02615      ----------EVQGPDLMIRMQDHAKAV-GT--EI--I-GDI-I-TDLNL----E-Q---
RUEPO00888      ----------EVQGPDLMVRMEAHAKAM-GC--EI--I-GDI-I-TSLDT----S-A---
RUEST00613      ----------EIQGPDLMVNMESHARAM-GT--EI--I-GDI-I-TSLDT----S-S---
PHAIB02390      ----------EVQGPDLMVRMQDHAKAM-GC--EI--I-GDI-I-TDLDT----T-S---
PARDP02130      ----------EIQGPDLMVRMEAHARAM-GA--RI--F-VDT-V-TRLDL----G-R---
DINSH02620      ----------SVMGPDLMVRMEEHAKAM-GT--EI--I-ADH-I-NRLDL----S-S---
RHOCB02768      ----------EVQGPELMVQMEEHARAM-GA--EV--I-TDI-I-TKLDL----G-T---
RHOS500248      ----------EVQGPDLMVRMEEHAKAM-GA--EV--I-SDY-I-ASLDL----S-Q---
RHOS400150      ----------EVQGPELMVRMEDHARAM-GA--EI--V-SDY-I-SSLDL----S-Q---
RHOS100227      ----------EVQGPELMVRMEDHARAM-GA--EI--V-SDY-I-SSLDL----S-Q---
RHOSK02952      ----------EVQGPELMVRMEDHARAM-GA--EI--V-SDY-I-SSLDL----S-Q---
MIDMI00790      ----------VIQGPWLMEQMKEQAKSV-GV--EI--I-DDY-I-TDIDL----K-I---
ACEP301595      ----------AIQGPWLMEQMAEQAQNV-GT--RI--E-YDI-I-TSVDF----KAG---
MICAA01566      ----------VIQGPWLMDQMKAQAEHV-GT--KM--V-YDF-I-KEVDF----N-V---
TISMK03676      ----------VIQGPWLMEQMQKQAEHV-GT--RF--H-HDI-I-TDIDL----K-T---
AZOL402409      ----------PIQGPWLMEQMQKQAEHV-GT--KM--V-FDL-I-TDVDF----T-R---
PSEUV04348      ----------PIQGPWLVEEMRKQAENV-GT--KI--D-YDT-I-LEADL----S-K---
HIRBI01095      ----------TIQGPWLMEQMKGQAEHV-GT--EI--V-EDH-I-KSVDL----N-N---
PARL102241      ----------AVQGPWLMEQMEAQARHM-GT--EM--V-ADT-I-VSADL----S-K---
MARMM02139      ----------VIQGPWLMDQMREQAEKV-GT--DM--V-ADI-I-VEADL----K-A---
PHEZH02588      ----------VIQGPWLMEQMQAQAEHV-GT--EI--V-NDI-V-VKADL----S-Q---
CAUCR02826      ----------VIQGPWLMDQMRAQAEHV-GT--EF--V-SDI-V-TSVDL----S-K---
CAUCN02939      ----------VIQGPWLMDQMRAQAEHV-GT--EF--V-SDI-V-TSVDL----S-K---
CAUST00891      ----------VIQGPWLMDQMRAQAEHV-GT--EL--V-NDI-V-TSVDL----S-K---
PARBH01522      ----------PIQGPWLMEQMQKQAENV-GT--KM--V-SDI-I-TEVDT----A-S---
PELHB02084      ----------PIQGPWLVEQMRAQAEHM-GT--RI--E-NDL-I-VDVDF----N-I---
HYPDA03273      ----------PIQGPWLMEQMQAQAEHV-GT--NI--V-MDQ-I-NKVDL----R-A---
HYPSM04642      ----------PIQGPWLMEQMQAQAEHV-GT--HI--I-MDH-I-NKVDL----R-V---
OLICO01107      ----------TIQGPWLMTEMEKQAIHV-GT--KI--V-TDH-V-NKLEL----A-Q---
OLICM02790      ----------TIQGPWLMTEMEKQAIHV-GT--KI--V-TDH-V-NKLEL----A-Q---
RHOPS01446      ----------VIQGPWLMEQMEKQALHV-GT--KI--V-TDL-V-VNLDL----S-Q---
RHOPA04062      ----------VIQGPWLMEQMEKQALHV-GT--RI--K-TDL-V-VDLDL----S-Q---
RHOPT04533      ----------VIQGPWLMEQMEKQALHV-GT--RI--K-TDL-V-VDLDL----S-Q---
RHOPX04235      ----------VIQGPWLMEQMEKQALHV-GT--RI--K-TDL-V-VELDL----S-Q---
BRADU07321      ----------VIQGPWLMEQMEKQAVHV-GT--KI--V-TDL-V-TKLET----G-R---
BRASO01239      ----------VIQGPWLMEQMEKQATHV-GA--RI--R-TDL-V-TKLEL----A-Q---
BRASB06352      ----------VIQGPWLMEQMEKQATHV-GA--QI--K-IDL-V-TRLEL----A-Q---
RHOPB03773      ----------VIQGPWLMEQMEKQATHV-GT--RI--V-TDL-V-TDLDL----S-Q---
NITWN02324      ----------VIQGPWLMEQMEKQAAHV-GT--RI--V-TDT-V-NSLDV----S-Q---
NITHX02707      ----------VIQGPWLMEQMEKQATHV-GT--RI--V-TDT-V-NSLDV----S-Q---
AZOC501143      ----------VIQGPWLMEQMRAQAEHV-GT--RI--L-SEH-V-ASLDL----S-R---
XANP202679      ----------PIQGPWLMEQMRAQAEHV-GT--KI--V-TDH-V-ASLDL----S-H---
CHLTF01983      ----------GILGPELMANMRAQALRF-ET--EI--L-TRN-V-TAVDF----K-T---
IGNAJ02236      ----------GIQGPELMDIMRKQAHRF-GA--QS--I-YKD-I-TEVDF----S-K---
MELRP00403      ----------GIQGPELMDIMRKQACKF-GA--KC--V-FKN-V-TEVDF----S-K---
ANADF00468      ----------GILGPELMELMKKQAERF-GT--RF--E-LGE-V-TRVDF----T-R---
CHLCH01125      ----------GIRGPELMGRMREQAAKF-GA--EF--V-AGS-V-TEVDL----S-K---
PELPB01419      ----------GIRGPELMAKMREQAAKF-GT--EF--V-GGS-V-TEVDL----S-R---
CHLL200781      ----------GINGPELMSRMRQQAERF-NA--EF--A-YGS-V-VEADI----S-R---
CHLTE00830      ----------GIPGPELMGRMREQAARF-GV--EF--Q-FGS-I-TEVDV----S-R---
CHLP800854      ----------GIPGPELMGRMREQAARF-GV--EF--K-FGS-V-TEADV----S-R---
CHLL701225      ----------GIQGPELMQRMRDQATKF-NA--EF--K-FGS-V-TEADL----S-R---
CHLPM00993      ----------GIAGPELMGRMRDQAAKF-NA--EF--V-FGS-V-TEADL----S-R---
WOLTR00562      ----------SIQGPELMKQMRLHVEKV-GA--EI--V-NDE-I-KSVEQ----L--EYS
WOLPP00135      ----------SIQGPELMEQKRLHAEKA-GA--RI--I-DDE-I-KSVEQ----L--EDS
WOLPM00650      ----------SIQGPELMEQMRFHAEKV-GA--KI--I-DDE-I-KSVEQ----L--EDF
```

```
WOLWR00416      ----------SIQGPELMEQMRLHAEKV-GA--KI--I-DDE-I-KSVEQ----L--EDS
EHRCR00696      ----------AVQGPDLMEQMKQQAHNS-GA--QI--I-SDE-I-KEIHS----------
ANAMM00345      ----------LVQGPELMEHMKQQALHC-GA--TE--I-MDE-I-TQIDA----------
ANAMF00341      ----------LVQGPELMEHMKQQALHC-GA--TE--I-MDE-I-TQIDA----------
ANAPZ00610      ----------AIEGPKLMEYMQEQAVKS-GA--TL--L-TDE-I-SEIDA----------
NEOSM00537      ----------PVQGPWLMEQMQQQAVNV-GC--RL--V-NDH-I-LQIEA----P-Y---
NEORI00513      ----------PVQGPWLMEQMQQQAVNV-GC--RF--V-NDH-I-SQVEA----P-Y---
PELUB00076      ----------VIQGPWLMDQMRDQAKAV-GT--DL--I-EDH-I-SSVNL----K-S---
PELSM00736      ----------VIQGPWLMEEMQKQAEAV-GT--IF--E-NDM-I-KEVNF----T-S---
PUNMI01409      ----------VIQGPWLMTEMQTQAENV-GA--RV--I-YDL-V-TTIDA----S-T---
ASTEC00651      ----------VIQGPWLMDQMKAQAEHM-GT--EV--I-TDI-V-VKADL----S-Q---
ZYMMT00154      ----------PVQGPWLMEQMQAQAENV-GA--RL--V-WDV-I-TSVDL------S---
ZYMMO00984      ----------PIQGPWLMEEMQAQAENV-GA--KL--V-WDI-I-TSVDF------S---
ZYMMA00176      ----------PIQGPWLMEEMQAQAENV-GA--KL--V-WDI-I-TSVDF------S---
ZYMMN00183      ----------PIQGPWLMEEMQAQAENV-GA--KL--V-WDI-I-TSVDF------S---
SPHAL00143      ----------VIQGPWLMEQMTAQAVHV-GT--SM--I-WDT-I-VDVDL------S---
SPHWW03978      ----------VIQGPWLMQEMQAQAEHV-GA--TM--M-WDT-I-VDVDL------S---
SPHJU02313      ----------VIQGPWLMEQMQAQAEHV-GA--QM--M-YDQ-I-VDVDL------S---
NOVAD02319      ----------VIQGPWLMQEMQAQAEHV-GT--RM--M-WDT-I-LEVSL------D---
ERYLH00934      ----------VIQGPWLMEQMQKQAEHV-GT--RM--M-WDT-I-VDVDI----E-N---
GRABC00720      ----------SVQGPWLMEQMEAQARHV-GT--EI--V-YDL-I-TDVDF----T-R---
GLUDA03075      ----------GIQGPELMEQMAAQAAHV-GT--QI--I-HDI-I-TECTL----K-GHGG
KOMMN00774      ----------GIQGPDLMMQMAEQAGNV-GT--RL--I-DDI-I-VSCDF----S-RSDG
HALVD01097      ----------GISGPELINNMKEQAERF-GT--EI--R-HGI-I-EDVDA----S-E---
HALHT01699      ----------GLSGPDLINNMKEQAERF-GT--EL--K-HGI-V-ADIDD----S-E---
METI401038      ----------GIQGPELMEAMRKQAERF-GT--RV--R-YLSVV-TEVEF----T-Q---
PLAL201612      RQYMAPHSGHGVTGPELMELMRAQAVNF-GT--RI--I-TAD-I-VSADL----S-Q---
GEMAT01983      ----------AVSGPELMDRIKAQAVHH-GA--RV--V-SEL-V-KSVDF----S-Q---
CYAAP02456      ----------GITGPNLMKEMRQQAQRW-GA--RL--I-RED-V-IKVDF----S-Q---
CHLPN00303      ----------GILGPKLMNNMKEQAVRF-GT--KT--L-AQD-I-ISVDF----S-V---
CHLPP00439      ----------GILGPKLMDNMKEQAVRF-GT--KT--L-AQD-I-ISVDF----S-V---
CHLPE00727      ----------GILGPVLMEKMKAQAVRY-GT--EV--L-SKD-I-TSVDF----S-K---
CHLTR00101      ----------GVLGHQLMDLMKTQAQRF-GT--QV--L-SKD-I-TAVDF----S-V---
CHLTA00102      ----------GVLGHQLMDLMKTQAQRF-GT--QV--L-SKD-I-TAVDF----S-V---
CHLTJ00101      ----------GVLGHQLMDLMKTQAQRF-GT--QV--L-SKD-I-TAVDF----S-V---
CHLTD00098      ----------GVLGHQLMDLMKTQAQRF-GT--QV--L-SKD-I-TAVDF----S-V---
CHLT700102      ----------GVLGHQLMDLMKTQAQRF-GT--QV--L-SKD-I-TAVDF----S-V---
CHLT000101      ----------GVLGHQLMDLMKTQAQRF-GT--QV--L-SKD-I-TAVDF----S-V---
CHLT500104      ----------GVLGHQLMDLMKTQAQRF-GT--QV--L-SKD-I-TAVDF----S-V---
CHLTL00098      ----------GVLGHQLMDLMKTQAQRF-GT--QV--L-SKD-I-TAVDF----S-V---
CHLTG00102      ----------GVLGHQLMDLMKTQAQRF-GT--QV--L-SKD-I-TAVDF----S-V---
CHLTS00101      ----------GVLGHQLMDLMKTQAQRF-GT--QV--L-SKD-I-TAVDF----S-V---
CHLT900102      ----------GVLGHQLMDLMKTQAQRF-GT--QV--L-SKD-I-TAVDF----S-V---
CHLTZ00101      ----------GVLGHQLMDLMKTQAQRF-GT--QV--L-SKD-I-TAVDF----S-V---
CHLT400432      ----------GVLGHQLMDLMKTQAQRF-GT--QV--L-SKD-I-TAVDF----S-V---
CHLT100103      ----------GVLGHQLMDLMKTQAQRF-GT--QV--L-SKD-I-TAVDF----S-V---
CHLT200350      ----------GVLGHQLMDLMKTQAQRF-GT--QV--L-SKD-I-TAVDF----S-V---
CHLTB00350      ----------GVLGHQLMDLMKTQAQRF-GT--QV--L-SKD-I-TAVDF----S-V---
CHLTC00378      ----------GVLGHQLMDLMKTQAQRF-GT--QV--L-SKD-I-TAVDF----S-V---
SIMNZ00831      ----------GISGPDLMDACKKQALRF-GT--EI--L-TED-V-KEVDL----T-T---
PARUW01706      ----------GITGPELVDRFRQQAIRF-GT--TI--I-SED-V-DSVDF----Q-V---
WADCW00943      ----------GVMGPELMMTMRKQAERF-GT--EV--I-TDD-V-VSVDF----S-E---
SINAD04980      ----------GVLGPELMELMRKQAQKF-GA--DC--R-YET-V-TEVDF----T-Q---
BIFLB01558      ----------GVMGPDLMESMQRQAERF-GA--EI--V-FDD-V-ESADL----------
BIFAB00498      ----------GVMGPDLMESMQRQAERF-GA--EI--V-FDD-V-ESADL----------
BIFAV01561      ----------GVMGPDLMESMQRQAERF-GA--EI--V-FDD-V-ESADL----------
BIFAS01557      ----------GVMGPDLMESMQRQAERF-GA--EI--V-FDD-V-ESADL----------
BIFA001518      ----------GVMGSDLMESMQRQAERF-GA--EI--V-FDD-V-ESADL----------
BIFBA01814      ----------GIMGPDLMDRMRDQAEKF-GT--QF--I-ADD-V-ESVEQ----V-E---
BIFAA01619      ----------GIMGPDLMDNMRKQAERF-GT--HI--V-WDD-V-VSVAS----N-A---
BIFDB02112      ----------GILGPDLMDNMRKQAEKF-GA--EI--I-WDD-V-VSVTV----D-V---
TERSS03091      ----------GVQGPELVENMRKQAQKF-GA--EM--E-MGH-L-VSVDL----T-K---
GRATM03160      ----------GIQGPELIENMKQQATRF-GA--EL--R-MAH-L-SSAEF----T-P---
GRAMM03836      ----------GVQGPELIENMKKQATRF-GA--EL--R-LAH-L-TSVDL----T-T---
AKKM801247      ----------GVMGPDLMFLMQQQAEKF-GT--RF--A-YED-V-KSVVR----D-E---
```

```
OPITP04130      ----------GIDGFQLMQNLREQATRF-GT--RF--E-QAL-V-TAVDF----T-S---
CORAD01794      ----------GIDGFTLMDNLRKQASRF-GA--RF--E-HAK-V-DKIEV----E-G---
BUCCC00180      ----------SISGIKLMERMLLHVKKF-SI--NI--I-DDI-I-QSVNF----S-R---
CENSY00347      ----------GIMGPDLMINFRKQAERM-GT--TI--V-DDE-V-VNVDF----R-H---
NITMS00668      ----------GIMGPDLMIEMRKQCQRM-GT--TI--V-DDE-A-VDVDF----R-R---
MEIRD02823      ----------PISGAELSERMVQQAKRF-GA--EI--V-MDE-A-QAVEK----T-P---
HERA203780      ----------AINGFDLADKMEKQAARF-GT--HF--L-DGI-V-EKVEL----G-E---
CHLAA02018      ----------GIGGFELAEAMEKQAARF-GA--QF--L-DSL-V-TKVEV----A-Q---
CHLSY02175      ----------GIGGFELAEAMEKQAARF-GA--QF--L-DSL-V-TKVEV----A-Q---
CHLAD02599      ----------GIGGFELAEAMEKQAARF-GT--QY--L-DSM-V-TKVDV----D-Q---
CALAS01745      ----------SLSGPELVEKFKAQAEKF-GT--RV--E-FDY-V-TELVL----D-R---
ANATU01696      ----------GVGGSQLGELFQKQAERF-GA--RV--E-FDI-A-SRVDL----S-K---
SULMS00235      ----------GVKGYLLMENLKKQAEKF-GT--KI--Y-FNN-V-IDINFK--LN-----
BLASB00041      ----------GIGGIDFMENCKKQAERF-NT--KI--I-NQS-V-TNVFLSNHKG-----
BLASP00551      ----------GISGNELMENCKKQAERF-NT--II--I-HKT-I-TKVDFSDKKG-----
AZOPC00437      ----------AITGIKMMENFKKQAQKF-GT--DV--R-KGT-V-TACDL----S-R---
LEPBD01878      ----------GISGPQLIEDIKAQSKNF-GT--EF--L-QAV-V-KDIESIENNG-K---
SALRD02577      ----------GLMGPEMMDRFQDQAERF-GT--ES--R-YGT-V-THVDF----R-E---
SALRM02866      ----------GLMGPEMMDRFQDQAERF-GT--ES--R-YGT-V-THVDF----R-E---
RIEPU00166      ----------SQTGSFIMENTLRQVRNF-IPFSDI--I-ED-QI-YKVDL----T-N---
ORITB00015      ----------TVSGPWIMDQMHNQVKKF-EA--EV--I-DD-HI-KAVNF----K-T---
ORITI01234      ----------TVSGPWIMDQMHNQVKKF-EV--EV--I-DD-HI-KAVNF----K-T---
PREMB01541      ----------GIDANQLMSEMREQAKLY-GA--DL--R-DG-SI-VKVDL----S-S---
PREDF01155      ----------GIDANQLMNEMRQQAALY-GA--DL--R-DG-SI-VKADL----S-S---
PREI702011      ----------GIDANQLMLEMKAQAINV-GA--DV--R-DG-SI-VKADL----S-K---
ALIFI01035      ----------GVSGQQMMADMRRQAERF-GA--DI--R-IG-TV-TSVDL----S-S---
ODOSD00362      ----------GVTGPVMMEDMRKQAERF-GT--DI--R-FG-IV-TAVDF----S-G---
PRER201250      ----------GVDGNQMMMDLLEQAQRF-GT--DI--R-NG-EI-VKVDF----S-K---
PALPW00206      ----------GTTGTELMEDLKKQAERF-GA--DI--R-FG-AA-TATDL----S-A---
PORGI00953      ----------GITGTELMEDLRKQATRF-GA--DI--R-SG-IA-TKADL----S-K---
PORG301168      ----------GITGTELMEDLRKQATRF-GA--DI--R-SG-IA-TKADL----S-K---
BACV803426      ----------GISGTQLMEDLRKQAERF-GA--DI--R-NG-IA-TSADL----S-K---
BACT601486      ----------GIGGSELMEDLRKQAERF-GA--DL--R-YG-VA-TAADL----G-Q---
BACTN04290      ----------GISGPQLMEDLRAQASRF-GT--DV--R-FG-IA-TAADL----S-K---
BACFR01032      ----------GISGPQLMEDLRTQAERF-GA--DI--R-FG-IA-TASDL----G-Q---
BACFN00913      ----------GISGPQLMEDLRTQAERF-GA--DI--R-FG-IA-TASDL----G-Q---
BACF600963      ----------GISGPQLMEDLRTQAERF-GA--DI--R-FG-IA-TASDL----G-Q---
OWEHD03413      ----------GIMGPEMMEDLKKQAERF-GT--DV--R-FG-MV-TKTEL----S-EI--
PSYTT00390      ----------GIDGPRMMVELQQQAERF-GT--DV--R-IG-MV-TKVEL----S-DK--
NONDD00011      ----------GIDGPAMMVDLQKQAERF-GT--QV--R-IG-MI-TEVNF----A-TE--
ROBBH02711      ----------GVDGPTMMVQLQQQAERF-GT--DV--R-IG-IA-TAVEF----S-DE--
CELAD02592      ----------GIDGPTMMMQLQTQAERF-GT--EV--R-IG-MI-TAVEL----S-TE--
CELLC02474      ----------GIDGPTMMTQLQAQAERF-GT--EV--R-IG-MV-TAVEF----N-ET--
MARSH03072      ----------GIDGPTMMVQLQQQAERF-GT--EV--R-IG-MI-TAVEL----S-TE--
MURRD00159      ----------GIDGPTMMIQLQQQAERF-GT--EV--R-IG-MV-TAVEL----S-DE--
ZOBGA00152      ----------GIDGPSMMVQLQQQAERF-GT--EV--R-IG-MV-TAVEL----S-DT--
GRAFK00949      ----------GIDGPKMMMELQEQAERF-GT--EV--R-IG-MI-TEVDF----S-KE--
ZUNPS02344      ----------GIDGPAMMMDLQKQAERF-GT--EV--R-MG-MV-TEVQF----S-KE--
AEQSU02079      ----------GIDGPTMMVQLQEQAERF-GT--VV--R-IG-MV-TNVEF----S-DK--
HALH105745      ----------GIMGPEMMEDFRKQAERF-GT--QI--R-YE-LI-NKVDF----S-G---
SAPGL02893      ----------GIMGPEMMEDFRNQAARF-GT--EI--H-TGVMI-EKVDF----S-G---
AMOA500014      ----------GALGSQMMVDFQTQAQRF-GT--DI--R-NA-TV-TAVNF----S-C---
FLAIG01802      ----------GITGPEMMVQLQAQAQRF-NA--DI--R-DG-WA-TKVDF----S-G---
FLACA02326      ----------GVTGPEMMVQLETQAKRF-GT--DI--R-NG-WV-TKVDF----S-A---
FLAJ100198      ----------GVTGPEMMIQLQEQAKRF-GA--DI--R-DG-WA-TKVDF----S-G---
FLABF01528      ----------GVTGPEMMLQLQAQAKRF-GA--DI--R-DG-WA-TKVDF----S-G---
CAPOD01333      ----------GVMGSEMMIQLEQQAKRF-GA--DI--R-NG-WV-TKVDF----S-A---
CAPCC00414      ----------GITGPEMMVELEQQAKRF-GA--DI--R-NG-WV-TKVDF----S-A---
FLELS01898      ----------GVMGPQMMQDLDKQAGRF-GT--DL--R-YG-VI-EKVEF----S-KEG-
SOLCM01166      ----------GTQGPEMMEDFKKQAERF-GT--EV--R-FG-IV-TAVDF----S-S---
PEDHD01598      ----------GIMGPEMMEDFRKQAERF-GT--DI--R-FG-YV-SGVDF----S-T---
SPHS203207      ----------GIMGPEMMEDFRKQAERF-GT--DI--R-FG-YA-TGVDF----S-G---
LEAB401188      ----------GIQGPAMMEDLRKQAERF-GL--DN--R-YG-LA-TSVDF----S-TP--
EMTOG00092      ----------GIQGPEMMEDFRKQAERF-GL--DN--R-YG-MA-TKVDF----S-TP--
SPILD01737      ----------GVQGPQMMQDLEAQARRF-GT--DI--R-YG-MV-TKVVF----A-DQPS
```

```
DYAFD00084      ----------GITGPEMMINFEKQARRF-GT--DV--R-YG-LA-TSVDF----T-G---
CYCMS03063      ----------GVMGPQMMEDFRKQAERF-GT--DV--R-YG-VV-TGIDA----G-K---
ECHVK03326      ----------GVMGPQMMEDFKKQAERF-GT--DV--R-YG-LV-TAVDF----S-T---
BELBD02542      ----------GIMGPEMMEDFRKQAERF-GT--KV--R-YG-LV-TKVDF----S-S---
NITGG02112      ----------GIFGPELMMNMRQQAERF-GG--VI--V-DD-EV-IKVDF----K-K---
THEM700111      ----------GILGPDLMARMRQQAERA-GA--RF--V-DG-DV-TGVDF----S-Q---
CREAS01350      ----------GILGPELMMNMRAQAERF-GT--EI--I-DK-DV-TKVDF----S-Q---
TURPD02772      ----------GIQGPELMDHFRGQAERF-GA--EL--K-SV-LA-DKAEL----S-GG--
SORC507603      ----------KITGQELMQRFRDQAAHQ-GT--EI--V-TA-DV-TKVEL----T-G---
BDEBA00337      ----------GITGPDLITVMRKQAERF-GT--RF--I-TR-NV-TKVDF----S-Q---
STIAD02752      ----------GITGPELMERFQKQAERF-GT--QI--H-ME-NV-VKVDL----S-S---
MYXXD01876      ----------AITGPELMDRFQKQAERF-GT--TI--H-ME-NV-VKVDF----S-Q---
MYXFH03454      ----------AITGPELMERFQKQAERF-GT--TI--H-ME-NV-VKVDF----S-Q---
CORCM01960      ----------AITGPELMERFQKQAERF-GT--VI--H-ME-NV-TKVDF----S-K---
MYXSD02209      ----------AITGPELMERFQKQAERF-GT--AI--H-ME-NV-VKVDF----S-Q---
LEPFC02126      ----------GITGPELIERMKAQVLRF-GT--RF--M-TR-SI-ESVVP----E-K---
LEPFM02294      ----------GVTGPELIEFMREQVLRF-GT--RF--E-TR-VV-EKVTR----E-K---
SOLUE00582      ----------GINGPDLVENMRVQAQNF-GA--EF--M-HG-SV-IDTDF----S-Q---
KORVE01333      ----------GIQGPELIENMHKQAERF-GT--EF--H-TG-HL-HSADL----S-K---
ACIC502553      ----------GIQGPELIDNMRKQASRF-GA--QF--R-LG-HL-VKADL----S-K---
ACIFD00030      ----------GILGPELMANMRQQAERF-GA--RF--R-GT-KV-SRLDL----A-E---
HALMS01402      ----------GIMGPELMANMKKQTQRF-GT--EY--L-QT-HV-TAVDL----S-K---
CHLPD01133      ----------GIFGPELMARMRQQIIRF-GV--EI--A-YG-SV-QDVDL----S-R---
CHLPB01137      ----------GVRGPAMMGMFRQQAAKF-GA--KF--L-SG-SA-VEVDV----S-R---
PROA200897      ----------GIRGPELMDKFREQAARF-DV--EF--A-LG-NV-REVDL----S-R---
WIGBR00492      ----------SLTGSELIKRMRDHAIKF-GT--KI--F-YDH-I-NEVNF----N-K---
BUCA500289      ----------KISGSELMNRMYKHAIEL-KT--KV--I-CDT-V-ISVNF----K-K---
BUCAI00292      ----------KISGSELMNRMYKHAIEL-KT--KV--I-CDT-V-ISVNF----K-K---
BUCAF00306      ----------KISGSELMNRMYKHAIEL-KT--KV--I-CDT-V-ISVNF----K-K---
BUCAT00287      ----------KISGSELMNRMYKHAIEL-KT--KV--I-CDT-V-ISVNF----K-K---
BUCA000302      ----------KISGSELMNRMYKHAIEL-KT--KV--I-CDT-V-ISVNF----K-K---
BAUCH00290      ----------DLTGSILMERMHIHATKY-NT--EI--I-SDN-I-IKVNL----K-Q---
BLOVB00369      ----------NLTGPSLMNRMKAHAIKF-NT--NI--I-DDH-I-IKVNF----Q-K---
BLOFL00367      ----------NLTGPQLMNRMKMHAINL-NT--KI--I-NDH-I-IKVNF----Q-K---
BLOPB00375      ----------NLTGPTLMNRMNTHAVSL-HT--EI--I-SDH-I-FKVNF----K-Q---
BUCAP00289      ----------KITGPELMNRMHEHSLKF-KT--EI--V-YDN-I-ISVEF----K-K---
COXBU01001      ----------GLQGPQLMERMQKHAERL-DT--QF--I-FDH-I-NEADL----N-Q---
COXBN01188      ----------GLQGPQLMERMQKHAERL-DT--QF--I-FDH-I-NEADL----N-Q---
COXBR01117      ----------GLQGPQLMERMQKHAERL-DT--QF--I-FDH-I-NEADL----N-Q---
COXB200717      ----------GLQGPQLMERMQKHAERL-DT--QF--I-FDH-I-NEADL----N-Q---
COXB100914      ----------GLQGPQLMERMQKHAERL-DT--QF--I-FDH-I-NEADL----N-Q---
FRAP200241      ----------GIMGPELMEKLQKQAERF-DT--QI--T-YDT-I-NSVDL----Q-T---
FRANT01012      ----------GIMGPELMEKLQKQAERF-DT--QI--T-YDT-I-NSVDL----Q-T---
FRACN00522      ----------GIMGPELMDKLQKQAERF-DT--QI--I-YDT-I-NAVDL----Q-N---
FRATT00465      ----------GIMGPELMEKLQKQAERF-DT--QI--V-YDT-I-NAVDL----Q-N---
FRAT100465      ----------GIMGPELMEKLQKQAERF-DT--QI--V-YDT-I-NAVDL----Q-N---
FRATE00462      ----------GIMGPELMEKLQKQAERF-DT--QI--V-YDT-I-NAVDL----Q-N---
FRATW01216      ----------GIMGPELMEKLQKQAERF-DT--QI--V-YDT-I-NAVDL----Q-N---
FRATM01110      ----------GIMGPELMEKLQKQAERF-DT--QI--V-YDT-I-NAVDL----Q-N---
FRACF00566      ----------GIMGPELMEKLQKQAERF-DT--QI--V-YDT-I-NAVDL----Q-N---
FRATO01212      ----------GIIGPELMEKLQKQAERF-DT--QI--V-YDT-I-NAVDL----Q-N---
FRATH01486      ----------GIIGPELMEKLQKQAERF-DT--QI--V-YDT-I-NAVDL----Q-N---
FRATF01216      ----------GIIGPELMEKLQKQAERF-DT--QI--V-YDT-I-NAVDL----Q-N---
FRATN00564      ----------GIMGPELMEKLQKQAERF-DT--QI--V-YDT-I-NAVDL----Q-N---
ACIF500512      ----------GIMGPELMQGLEKQARRF-DT--EI--L-FDH-I-HTADL----R-Q---
ACIF200362      ----------GIMGPELMQGLEKQARRF-DT--EI--L-FDH-I-HTADL----R-Q---
DECAR01284      ----------GIQGPELMANFEAHARRF-ET--EI--V-FDH-I-HTTQL----T-E---
NEIG100525      ----------GVQGPELMARFLAHAERF-GT--EI--I-FDQ-I-NAVDL----Q-K---
NEIG201314      ----------GVQGPELMARFLAHAERF-GT--EI--I-FDQ-I-NAVDL----Q-K---
NEIM800926      ----------GVQGPELMARFLAHAERF-GT--EI--I-FDQ-I-NAVDL----Q-K---
NEIMP01199      ----------GVQGPELMARFLAHAERF-GT--EI--I-FDQ-I-NAVDL----Q-K---
NEIMB01212      ----------GVQGPELMARFLAHAERF-GT--EI--I-FDQ-I-NAVDL----Q-K---
NEIMF01143      ----------GVQGPELMARFLAHAERF-GT--EI--I-FDQ-I-NAVDL----Q-K---
NEIML01119      ----------GVQGPELMARFLAHAERF-GT--EI--I-FDQ-I-NAVDL----Q-K---
NEIMM00780      ----------GVQGPELMARFLAHAERF-GT--EI--I-FDQ-I-NAVDL----Q-K---
```

```
NEIMH00827      ----------GVQGPELMARFLAHAERF-GT--EI--I-FDQ-I-NAVDL----Q-K---
NEIMG01168      ----------GVQGPELMARFLAHAERF-GT--EI--I-FDQ-I-NAVDL----Q-K---
NEIMN01254      ----------GVQGPELMARFLAHAERF-GT--EI--I-FDQ-I-NAVDL----Q-K---
NEIMO00810      ----------GVQGPELMARFLAHAERF-GT--EI--I-FDQ-I-NAVDL----Q-K---
NEIM701229      ----------GVQGPELMARFLAHAERF-GT--EI--I-FDQ-I-NAVDL----Q-K---
NEIMA01334      ----------GVQGTELMARFLAHAERF-GT--EI--I-FDQ-I-NAVDL----Q-K---
NEIMW01125      ----------GVQGTELMARFLAHAERF-GT--EI--I-FDQ-I-NAVDL----Q-K---
DICNV01037      ----------GILGSELMTNMCQHAQRF-QT--QI--I-YDH-V-TEVNL----Q-H---
VESOH00047      ----------GVQGPDLMARMKKHAEYF-DT--QV--I-NDT-I-ISVNF----V-K---
RUTMC00043      ----------GVQGPDLMARMKKHAERF-DT--QI--L-NDT-I-TSVNF----T-K---
HALHL02255      ----------GIQGPELMDRMRRQAERF-ET--EI--I-FDH-I-HTARV----T-G---
PELPD03177      ----------GVQGPELMERMRLHAERF-NT--RI--I-YDH-I-TRADL----G-R---
GEOS804030      ----------GVMGPELMERMRQHAERF-GT--QF--V-FDH-I-NKAQV----S-K---
GEOBB03636      ----------GVMGPDLMERMRLHAERF-GT--QF--I-YDH-I-SKAQV----T-K---
GEOSM03700      ----------GVLGPDLMERMRLHAERF-GT--QF--I-YDH-I-SKAQV----T-K---
HALNC00046      ----------DLTGPALMDRMKAHAEKF-DT--EI--I-FDH-I-QSTNL----K-Q---
HAEPS00887      ----------DITGSELMNKMQKHAEKF-NT--EI--I-FDH-I-NSVDL----S-Q---
GALAU02285      ----------ETTGPELMQRMLEHAEKF-EA--EI--V-FDH-I-NKVDL----S-K---
HISS201000      ----------DTTGPELMQRMLQHAEKF-DT--EI--V-FDH-I-NNVDL----S-E---
HAES101151      ----------DTTGPELMQRMLQHAEKF-DT--EI--V-FDH-I-NNVDL----S-E---
PASMU00573      ----------ETTGPELMQRMLQHAEKF-DT--EI--I-FDH-I-NKVDL----S-S---
PASMH00579      ----------ETTGPELMQRMLQHAEKF-DT--EI--I-FDH-I-NKVDL----S-S---
ACTSZ01560      ----------DTTGPELMQRMLQHAEKF-ET--EI--V-FDH-I-NSVDL----S-S---
AGGAN00985      ----------ETTGPELMQRMLQHAEKF-ET--EI--V-FDH-I-NRVDL----S-S---
NITHN03181      ----------GVQGPELMERMRKHAERF-GT--EI--V-FDQ-I-TEVDL----S-Q---
NITOC00311      ----------GVQGPELMERMRKHAERF-GT--KV--I-LEH-I-NEVDF----S-Q---
NITWC02498      ----------GVQGPELMERMRKHAERF-GT--EV--I-LEH-I-NEVDF----S-Q---
METNJ00648      ----------GVQGPELMQRMQKHAERF-GT--DI--I-FDH-I-HTADV----T-Q---
METFJ01616      ----------GVQGPELMQRMQKHAERF-GT--EI--V-FDH-I-HTADL----Q-Q---
ALKEH00244      ----------GVQGPELMERMKRHAERF-ET--EV--V-FDH-I-HTAHL----K-A---
MARMS03166      ----------GVQGPELMERMRKHAERF-ET--EI--I-FDH-V-NKVDL----K-N---
MARM102688      ----------GVQGPELMERMRKHAERF-ET--EI--I-FDH-V-GKVDL----Q-Q---
THICR00763      ----------GLTGPDLMVRMQKHAERF-GT--EI--L-FDH-I-HTAEL----N-Q---
THICA00974      ----------GLTGPDLMVRMQKHAERF-GT--EI--L-FDH-I-HTADL----S-K---
THIV600851      ----------GVQGPELMERMRRHAERF-GT--EI--L-FDH-I-NAVDL----A-E---
THISH01973      ----------GVQGPELMERMKRHAERF-NT--EI--I-FDH-I-NTVDF----S-K---
METAA00813      ----------GLQGPDLMERMRQHAERF-NT--EI--V-FDH-I-HTADL----S-A---
META200813      ----------GLQGPDLMERMRQHAERF-NT--EI--V-FDH-I-HTADL----S-A---
METMM02895      ----------GVQGPELMDRMLRHAERF-NT--EV--I-FDH-I-HTADL----S-K---
FRAAD00813      ----------GVQGPELMQRMAEHAERF-KT--DM--I-FDH-I-HTAEL----G-Q---
XYLFA01416      ----------GLMGPDLMERMQAHAERF-DT--KV--I-FDQ-I-YKADL----S-T---
XYLFT00623      ----------GLMGPDLMERMQAHAERF-ET--EV--I-FDQ-I-YKADL----S-T---
XYLF200667      ----------GLMGPDLMERMQAHAERF-ET--EV--I-FDQ-I-YKADL----S-T---
XYLFG01622      ----------GLMGPDLMERMQAHAERF-ET--EV--I-FDQ-I-YKADL----S-T---
XYLFM00690      ----------GLMGPDLMERMQAHAERF-ET--EV--I-FDQ-I-YKADL----S-T---
PSEUP01680      ----------GLMGPDLMARMQAHAERF-ET--EV--I-FDH-I-HTADL----S-Q---
STRM501972      ----------GLMGPDLMARMQAHAERF-ET--EV--I-FDH-I-HTADL----S-Q---
PSEUU01524      ----------GLMGPDLMARMQAHAERF-DT--EV--I-FDH-I-HTADL----S-Q---
XANAP01407      ----------GLMGPDLMARMQAHAERF-DT--EV--I-FDH-I-HTADL----S-Q---
XANCP01918      ----------GLMGPDLMSRMQAHAERF-DT--EV--I-FDH-I-HTADL----S-Q---
XANC802154      ----------GLMGPDLMSRMQAHAERF-DT--EV--I-FDH-I-HTADL----S-Q---
XANCB02263      ----------GLMGPDLMSRMQAHAERF-DT--EV--I-FDH-I-HTADL----S-Q---
XANOR02379      ----------GLMGPDLMTRMQAHAERF-ET--EV--I-FDH-I-HTADL----S-Q---
XANOM02295      ----------GLMGPDLMTRMQAHAERF-ET--EV--I-FDH-I-HTADL----S-Q---
XANOP02318      ----------GLMGPDLMTRMQAHAERF-ET--EV--I-FDH-I-HTADL----S-Q---
XANAC01951      ----------GLMGPDLMSRMQAHAERF-ET--EV--I-FDH-I-HTADL----S-Q---
CYCSP01155      ----------GLQGPALMERMKQHAERF-DT--KI--Q-FDH-I-HTTEL----Q-Q---
GEOLS03275      ----------GVQGPDLMERMRQHAERF-NT--QI--I-YDT-I-VSTDL----T-Q---
GEOUR03858      ----------GVQGPELMDRMLRHAERF-DT--QV--I-YDH-I-HKADL----K-Q---
GEODF00692      ----------GVQGPELMERMLKHAERF-NT--RI--I-YDH-I-NKTDL----Q-Q---
GEOSL00482      ----------GVLGPDLMDRMRRHAERF-NT--AM--I-YDH-I-HTANL----R-E---
GEOSK00469      ----------GVLGPDLMDRMRRHAERF-NT--AM--I-YDH-I-HTANL----R-E---
GEOMG02998      ----------GVQGPDLMERMRQHAERF-NT--TM--V-FDH-I-GKANL----R-E---
MORCR00228      ----------GLTGNGLMERMKAHAERF-GT--KL--I-YDS-I-TQVDL----H-N---
ACIAD00798      ----------GLTGPALMERMQAHAERF-GT--EI--V-YDH-I-NEVDL----K-T---
```

ACIBC00821	----------GLTGPVLMDRMQAHAERF-GT--EL--V-YDH-I-NEVDL----N-V---
ACIBY02725	----------GLTGPVLMDRMQAHAERF-GT--EL--V-YDH-I-NEVDL----N-V---
ACIB302691	----------GLTGPVLMDRMQAHAERF-GT--EL--V-YDH-I-NEVDL----N-V---
ACIB500882	----------GLTGPVLMDRMQAHAERF-GT--EL--V-YDH-I-NEVDL----N-V---
ACIB100850	----------GLTGPVLMDRMQAHAERF-GT--EL--V-YDH-I-NEVDL----N-V---
ACIBD00843	----------GLTGPVLMDRMQAHAERF-GT--EL--V-YDH-I-NEVDL----N-V---
ACIBS02229	----------GLTGPVLMDRMQAHAERF-GT--EL--V-YDH-I-NEVDL----N-V---
ACICP00137	----------GLTGPALMDRMQAHAERF-GT--EL--V-YDH-I-NEVDL----N-V---
ACISD03052	----------GLTGPALMDRMQAHAERF-GT--EL--V-YDH-I-NEVDL----N-V---
LEGLN02402	----------GLQGPALMERMQKHAERF-ET--KV--I-FDH-I-VKADL----A-Q---
LEGPA01726	----------GLQGPALMERMQKHAERF-DT--QV--I-FDH-I-VKADL----A-Q---
LEGPH00846	----------GLQGPALMERMQKHAERF-DT--QV--I-FDH-I-VKADL----A-Q---
LEGPC01171	----------GLQGPALMERMQKHAERF-DT--QV--I-FDH-I-VKADL----A-Q---
LEGP201857	----------GLQGPALMERMQKHAERF-DT--QV--I-FDH-I-VKADL----A-Q---
LEGPL01727	----------GLQGPALMERMQKHAERF-DT--QV--I-FDH-I-VKADL----A-Q---
COLP302668	----------DLTGPALMERMQKHAEKF-DT--EI--I-FDH-I-ESVDF----S-S---
KANKD01106	----------GVQGPELMVRMQKHAERF-GT--EM--I-FDH-I-HTVEL----K-N---
IDILO00659	----------GLTGPDLMVRMQKHAERF-DT--EI--V-FDH-I-SSVDF----S-K---
PSEU901284	----------GLTGPALMDRMKEHAERF-ET--EI--V-FDH-I-NKVDV----S-K---
PSEA602346	----------GLTGPDLMVRMQQHAEKF-DT--EI--I-FDH-I-NKTDL----T-K---
ALTSS01871	----------GLTGPDLMVRMQKHAEKF-DT--EI--I-FDH-I-NKTDL----T-K---
ALTMD01611	----------GLTGPDLMVRMQKHAEKF-DT--EI--I-FDH-I-NKTDL----T-K---
ALTME01705	----------GLTGPDLMVRMQKHAEKF-DT--EI--I-FDH-I-NKTDF----S-K---
ALTMB01797	----------GLTGPDLMVRMQKHAEKF-DT--EI--I-FDH-I-NKTDF----S-K---
ALTMS01652	----------GLTGPDLMVRMQKHAEKF-DT--EI--I-FDH-I-NKTDF----S-K---
SACD201683	----------DLQGPDLMVQMQQHAERF-DT--DI--I-FDH-I-HECDL----S-K---
TERTT01748	----------DLQGPDLMVQMQQHAERF-NT--EI--I-FDH-I-HECDL----N-S---
SIMAS00609	----------DLQGPDLMVQMQQHAERF-NT--EI--I-FDH-I-DEVKL----N-E---
ALCDB01962	----------GLQGPDLMVRMQQHAERF-ET--QV--L-FDH-I-NEVVL----D-Q---
CHRSD02939	----------GVQGPELMERMRSHAERF-DT--EV--L-FDH-I-NEVEL----R-E---
HALED02811	----------GVQGPELMERMKRHAERF-ET--EV--L-FDH-V-HEVEL----R-E---
SIDLE02655	----------GVQGPELMQRFQQHAERF-NT--EI--L-FDH-I-HTARL----Q-Q---
GALCS01621	----------GVQGPELMARLQQHAERF-NT--LM--I-SDH-I-HTARL----Q-Q---
LARHH01446	----------GVMGPELMQRFQQHAERF-GT--EM--I-FDQ-I-HTVEL----R-Q---
CHRVO02807	----------GVQGPELMQRFQQHAERF-GT--EI--L-FDH-I-HTTHL----S-E---
PSEUL01648	----------GVQGPELMVRFQQHAERF-GT--EM--I-FDH-I-HTTHL----Q-E---
NITEU01859	----------GVQGPELMERFLKHAERF-QT--EV--I-FDH-I-HTANL----S-E---
NITEC00745	----------GVQGPELMERFLKHAERF-QT--EI--I-FDH-I-HTAKL----S-G---
NITMU00023	----------GVQGPELMERFQKHAERF-DT--EI--I-FDH-I-HTARL----S-E---
NITSI03064	----------GVQGPELMERFQKHAERF-HT--EI--I-FDH-I-HTAKL----T-E---
ACCPU02128	----------GVQGPELMQRFEEHARRF-ET--EI--I-FEH-I-HTAQL----A-Q---
THIDA01000	----------GVQGPELMDRFQRHAERF-QT--EI--I-FDQ-I-HTAKL----S-E---
METS601221	----------GVQGPELMARFQKHAERF-NT--EM--I-FDH-I-HTAKL----T-E---
METGS01186	----------GVQGPELMARFQKHAERF-NT--EM--I-FDH-I-HTAKL----T-E---
METFK00972	----------GVLGPELMARFQKHAERF-NT--EI--I-FDH-I-HTTHL----T-E---
METML01428	----------GVQGPELMSRFQKHAERF-NT--EM--I-FDH-I-HTTHL----T-E---
AROAE03899	----------GVMGPDLMARFQKHAERF-NT--EM--V-FDH-I-HTVKL----G-E---
THASP01686	----------GVMGPDLMARFQRHAERF-NT--EM--I-FDH-I-HTVKL----G-E---
AZOSB01359	----------GVQGPELMERFQKHAERF-NT--EM--I-FDH-I-HTVQL----Q-Q---
BORA102682	----------GVQGPALMERFQKHAERF-NT--EI--V-FDH-I-AKVDL----S-Q---
BORPA03415	----------GVQGPDLMQRFLQHAERF-NT--EI--L-FDH-V-AKVDL----S-R---
BORBM03577	----------GVQGPDLMQRFLQHAERF-NT--EI--L-FDH-I-AKVDL----S-R---
BORPE02280	----------GVQGPDLMQRFLQHAERF-NT--EI--L-FDH-I-AKVDL----S-R---
BORPC02060	----------GVQGPDLMQRFLQHAERF-NT--EI--L-FDH-I-AKVDL----S-R---
BORP102392	----------GVQGPDLMQRFLQHAERF-NT--EI--L-FDH-I-AKVDL----S-R---
BORBR03870	----------GVQGPDLMQRFLQHAERF-NT--EI--L-FDH-I-AKVDL----S-R---
BORPD01524	----------GVQGPDLMQRFQQHAERF-NT--DI--V-FDH-I-AKVDL----S-Q---
ACHXA01179	----------GVQGPDLMQRFQKHAERF-NT--EM--I-FDH-I-AKVDV----S-K---
RHOFT03114	----------GVQGPDLMQRFLAHAERF-KT--EI--V-FDH-I-NTVDF----S-K---
VEREI01701	----------GVQGPDLMQRFLQHAERF-KT--QV--I-FDH-I-HRADL----G-Q---
VARPE01375	----------GVQGPELMQRFLEHAERF-KT--QI--V-FDH-I-NKVDL----S-K---
VARPS01266	----------GVQGPELMQRFLEHAERF-KT--QI--V-FDH-I-NKVDL----G-K---
DELAS05272	----------GVQGPELMQRFLEHAERF-KT--QV--V-FDH-I-HSVDF----S-K---
DELSC01235	----------GVQGPELMQRFLEHAERF-KT--QV--V-FDH-I-HSVDF----S-K---
COMT200883	----------GVQGPELMQRFQEHAERF-KT--EI--V-FDH-I-NQVDL----S-K---

```
ACIAC03267      ----------GVQGPELMQRFLEHAERF-KT--QV--V-FDH-I-NQVDL----S-K---
ACIAP03249      ----------GVQGPELMQRFLEHAERF-KT--QV--V-FDH-I-NQVDL----S-K---
ACIET02648      ----------GVQGPELMQRFQEHAERF-KT--QI--V-FDH-I-SQVDL----S-K---
ALIDK03528      ----------GVQGPELMQRFQEHAERF-KT--RI--V-FDH-I-GKVDL----S-R---
RUBGI03408      ----------GVMGPDLMQRFQQHAERF-NT--KM--V-FDH-V-NAVDF----S-Q---
LEPCP00749      ----------GVQGPELMQRFQKHAERF-NT--KM--V-FDH-I-NEVDL----V-Q---
POLSJ03719      ----------GVQGPDLMQRFLEHAERF-KT--EI--I-FDH-I-NQVDL----S-K---
POLNA03144      ----------GVQGPELMQRFLAHAERF-KT--EI--I-FDH-I-NQVDL----S-Q---
METPP01110      ----------GVQGPELMQRFLEHAERF-KT--QV--V-FDH-I-NAVDF----S-K---
RAMTT03218      ----------GVQGPELMQRFLEHAERF-KT--EI--V-FDH-I-NAVDF----G-K---
RALPJ02457      ----------HLQGPELMQRFLEHAERF-KT--EV--L-FDH-I-HTAHL----K-E---
RALP102102      ----------HLQGPELMQRFLEHAERF-KT--EV--L-FDH-I-HTAHL----N-E---
RALSO02303      ----------GLQGPELMQRFLEHAERF-ST--DV--V-FDH-I-HTAHL----T-E---
RALS801097      ----------GLQGPELMQRFLEHAERF-ST--EV--V-FDH-I-HTAQL----A-E---
HERSS01878      ----------GVQGPELMQRLLQHAERF-KT--EI--I-FDH-I-HTTKL----S-E---
HERAR00955      ----------GVQGPELMQRLLQHAERF-NT--EI--I-FDH-I-HTTKL----D-E---
JANMA01152      ----------GVQGPELMQRLLQHAERF-NT--EI--I-FDH-I-HTTKL----D-E---
THIK102324      ----------GVQGPDLMARFQAHAERF-NT--EI--V-FDH-I-HTAHL----R-E---
POLSQ00682      ----------GVQGPELMDRFLKHAERF-NT--EI--I-FDH-I-HTAAL----K-E---
POLNS00989      ----------GVQGPELMDRFLKHAERF-NT--EI--I-FDH-I-HTAAL----K-E---
BURP800627      ----------GVQGPELMARFLEHAERF-NT--EI--V-FDH-I-HTARL----D-E---
BURPP00988      ----------GVQGPELMTRFLEHAERF-NT--EI--I-FDH-I-HTAKL----D-E---
BURSC00728      ----------GVQGPELMARFLEHAERF-NT--EI--I-FDH-I-HTAKL----D-E---
BURXL00982      ----------GVQGPELMARFLEHAERF-NT--EI--I-FDH-I-HTAKL----D-E---
BURSG00869      ----------GVQGPELMARFLEHAERF-NT--EI--I-FDH-I-HTAKL----N-E---
BURRH00629      ----------GVQGPELMQRLLEHAQRF-NT--EI--V-FDH-I-HTAKL----D-E---
BURGB00781      ----------GVQGPELMQRFLEHAERF-NT--EI--V-FDH-I-HTARL----D-E---
BURGS00853      ----------GVQGPELMQRFLEHAERF-NT--EI--V-FDH-I-HTAKL----E-E---
BURPS02618      ----------GVQGPELMQRFLAHAQRF-NT--EI--V-FDH-I-HTAKL----H-E---
BURMA01741      ----------GVQGPELMQRFLAHAQRF-NT--EI--V-FDH-I-HTAKL----H-E---
BURP103016      ----------GVQGPELMQRFLAHAQRF-NT--EI--V-FDH-I-HTAKL----H-E---
BURP002984      ----------GVQGPELMQRFLAHAQRF-NT--EI--V-FDH-I-HTAKL----H-E---
BURM701866      ----------GVQGPELMQRFLAHAQRF-NT--EI--V-FDH-I-HTAKL----H-E---
BURP602945      ----------GVQGPELMQRFLAHAQRF-NT--EI--V-FDH-I-HTAKL----H-E---
BURM902465      ----------GVQGPELMQRFLAHAQRF-NT--EI--V-FDH-I-HTAKL----H-E---
BURMS00740      ----------GVQGPELMQRFLAHAQRF-NT--EI--V-FDH-I-HTAKL----H-E---
BURTA01515      ----------GVQGPELMQRFLAHAERF-NT--EI--V-FDH-I-HTAKL----H-E---
BURM102386      ----------GVQGPELMARFLEHAERF-NT--EI--V-FDH-I-HTAKL----H-E---
BURL300890      ----------GVQGPELMARFLEHAERF-NT--EI--I-FDH-I-HTAKL----H-E---
BURVG00860      ----------GVQGPELMARFQEHAERF-NT--EI--V-FDH-I-HTAKL----H-E---
BURCM00829      ----------GVQGPELMARFQEHAERF-NT--EI--V-FDH-I-HTAKL----H-E---
BURA400832      ----------GVQGPELMARFQEHAERF-NT--EI--V-FDH-I-HTAKL----H-E---
BURCA00489      ----------GVQGPELMARFQEHAERF-NT--EI--V-FDH-I-HTAKL----H-E---
BURCH00966      ----------GVQGPELMARFQEHAERF-NT--EI--V-FDH-I-HTAKL----H-E---
BURCC00927      ----------GVQGPELMARFQEHAERF-NT--EI--V-FDH-I-HTAKL----H-E---
BURCJ02922      ----------GVQGPELMARFQEHAERF-NT--EI--V-FDH-I-HTAKL----H-E---
EDWI902348      ----------GLTGPLLMERMNQHAAKF-NT--EI--I-LDH-I-QRVDL----S-Q---
EDWTF01971      ----------GLTGPLLMERMNQHAAKF-NT--EI--I-LDH-I-QRVDL----S-Q---
EDWTE02176      ----------GLTGPLLMERMNQHAAKF-NT--EI--I-LDH-I-QRVDL----S-Q---
SODGM01094      ----------GLTGPLLMERMYAHATKF-NT--EI--I-FDH-I-TQVNL----Q-T---
MOREP00394      ----------GLTGTLLMERMYAHAKKF-NT--EI--I-VDH-I-TQVAL----Q-N---
RAHSY01432      ----------GLTGPGLMERMHEHATKF-NT--EI--I-FDH-I-NKVDL----Q-N---
RAHAC01419      ----------GLTGPALMERMHEHATKF-NT--EI--I-FDH-I-NKVDL----Q-N---
ERWBE01489      ----------DLTGPALMERMQEHAEKF-NT--EI--I-FDH-I-HTVDL----Q-T---
PANAM01339      ----------DLTGPSLMERMHEHAEKF-NT--EI--I-FDH-I-HSVDL----Q-N---
PANAA00662      ----------DLTGPSLMERMHEHAEKF-NT--EI--I-FDH-I-HSVDL----Q-N---
PANSA01303      ----------DLTGPSLMERMHEHAEKF-NT--EI--I-FDH-I-HTVDL----Q-N---
ERWT902154      ----------DLTGPLLMERMQEHALKF-NT--EI--I-FDH-I-HSVDL----Q-N---
ERWAC01330      ----------ELTGPRLMERMHEHAVKF-NT--EI--V-FDH-I-HRVDL----Q-N---
ERWAE01326      ----------ELTGPRLMERMHEHAVKF-NT--EI--V-FDH-I-HRVDL----Q-N---
ERWPE02241      ----------ELTGPGLMDRMHEHAVKF-NT--EI--V-FDH-I-HSVDL----Q-H---
ERWP602414      ----------ELTGPGLMDRMHEHAVKF-NT--EI--V-FDH-I-HSVDL----Q-H---
ERWSE02396      ----------ELTGPGLMDRMHEHAVKF-NT--EI--V-FDH-I-HSVDL----Q-H---
PECCP01694      ----------DLTGPLLMERMHAHATKF-NT--EV--I-FDH-I-ERVDL----Q-N---
PECWW01889      ----------DLTGPLLMERMHAHATKF-NT--EV--I-FDH-I-ERVDL----Q-N---
```

```
PECSS01870      ----------DLTGPLLMERMHAHATKF-NT--EV--I-FDH-I-ERVDL----Q-N---
PECAS02624      ----------DLTGPLLMERMHAHATKF-NT--EV--I-FDH-I-ERVDL----Q-N---
DICDC02220      ----------DLTGPLLMERMHDHAVKF-NT--EI--V-FDH-I-NRVDL----Q-Q---
DICZE02284      ----------DLTGPLLMERMHAHAAKF-NT--EI--V-FDH-I-TRVDL----Q-T---
DICD302017      ----------DLTGPLLMERMHAHAAKF-NT--EI--V-FDH-I-TRVDL----Q-T---
DICD502250      ----------DLTGPLLMERMHAHAVKF-NT--EI--V-FDH-I-TRVDL----Q-T---
XENBS00829      ----------GLTGPDLMERMFQHAEKF-QT--EI--I-SDH-I-QKVDF----E-K---
XENNA01480      ----------GLTGPNLMERMFQHAEKF-QT--EI--I-SDH-I-KKVDF----S-K---
PHOLL01537      ----------GLTGPGLMERMHQHAEKF-QT--EI--I-SDH-I-QKVDL----Q-S---
PHOAA02804      ----------GLTGPGLMDRMHQHAEKF-QT--DI--I-FDH-I-QKVDL----Q-N---
SERP501669      ----------GLTGPALMERMREHAEKF-QT--EI--V-FDH-I-NSVDL----Q-N---
SERSA01625      ----------GLTGPALMERMREHAEKF-QT--EI--V-FDH-I-NSVDL----Q-N---
YERPE01271      ----------GLTGPALMERMHEHAEKF-QT--EI--I-FDH-I-SSVDL----Q-N---
YERPS01390      ----------GLTGPALMERMHEHAEKF-QT--EI--I-FDH-I-SSVDL----Q-N---
YERPA00627      ----------GLTGPALMERMHEHAEKF-QT--EI--I-FDH-I-SSVDL----Q-N---
YERPN02489      ----------GLTGPALMERMHEHAEKF-QT--EI--I-FDH-I-SSVDL----Q-N---
YERPP02190      ----------GLTGPALMERMHEHAEKF-QT--EI--I-FDH-I-SSVDL----Q-N---
YERP302544      ----------GLTGPALMERMHEHAEKF-QT--EI--I-FDH-I-SSVDL----Q-N---
YERPB01460      ----------GLTGPALMERMHEHAEKF-QT--EI--I-FDH-I-SSVDL----Q-N---
YERPY02633      ----------GLTGPALMERMHEHAEKF-QT--EI--I-FDH-I-SSVDL----Q-N---
YERPG01392      ----------GLTGPALMERMHEHAEKF-QT--EI--I-FDH-I-SSVDL----Q-N---
YERPD01171      ----------GLTGPALMERMHEHAEKF-QT--EI--I-FDH-I-SSVDL----Q-N---
YERP100890      ----------GLTGPALMERMHEHAEKF-QT--EI--I-FDH-I-SSVDL----Q-N---
YERPZ01206      ----------GLTGPALMERMHEHAEKF-QT--EI--I-FDH-I-SSVDL----Q-N---
YERPH02469      ----------GLTGPALMERMHEHAEKF-QT--EI--I-FDH-I-SSVDL----Q-N---
YERE801438      ----------GLTGPALMERMHAHAEKF-QT--EI--L-FDH-I-HKVDL----Q-N---
YERE302565      ----------GLTGPALMERMHAHAEKF-QT--EI--L-FDH-I-HKVDL----Q-N---
YERE100392      ----------GLTGPALMERMHAHAEKF-QT--EI--L-FDH-I-HKVDL----Q-N---
PROMH00687      ----------GLTGPGLMDRMFQHAEKF-NT--EI--I-SDH-I-NKVDL----K-N---
PROSM03196      ----------GLTGPGLMDRMYEHATKF-QT--EI--I-SDH-I-NKVDL----K-Q---
TOLAT02279      ----------GLTGPALMDRMKAHAEHF-DT--QI--L-FDH-I-NAVEL----Q-Q---
AERVB02262      ----------GLTGPALMERMKEHAEKF-DT--RI--L-FDH-I-NSVEL----T-Q---
AERHH01811      ----------GLTGPALMERMKEHAEKF-DT--RI--L-FDH-I-NEVQL----T-Q---
AERS402185      ----------GLTGPALMERMKAHAEKF-DT--RI--L-FDH-I-NEVEL----T-Q---
PSYIN02070      ----------DMTGPGLMDSMKDHAERF-DT--KI--I-FDH-I-NEVEL----T-K---
SHELP02018      ----------DLTGPALMERMQKHAEKF-ET--EI--I-FDH-I-NEVNL----Q-E---
SHEVD02297      ----------DLTGPALMERMQKHAEKF-DT--EI--I-FDH-I-NEVNL----D-V---
SHEPW02341      ----------DLTGPALMERMQKHAEKF-ET--EI--I-FDH-I-NEVNL----N-V---
SHEPA02202      ----------DLTGPALMERMQKHAEKF-ET--EI--I-FDH-I-NEVNL----N-V---
SHEHH02007      ----------DLTGPALMERMQKHAEKF-ET--EI--I-FDH-I-NEVNL----N-V---
SHESH02117      ----------DLTGPALMERMQKHAEKF-ET--EI--L-FDH-I-NEVDL----K-V---
SHEWM02454      ----------DLTGPALMERMQKHAEKF-ET--EI--I-FDH-I-NEVDL----Q-V---
SHEAM01758      ----------GLTGPGLMARMQEHAEKF-NT--EI--I-FDH-I-NEVTL----T-E---
SHEON02159      ----------DLTGPALMERMQKHAEKF-DT--EI--L-FDH-I-NEVTL----T-E---
SHESM01945      ----------DLTGPALMERMQKHAEKF-DT--EI--L-FDH-I-NEVTL----T-E---
SHESR01991      ----------DLTGPALMERMQKHAEKF-DT--EI--L-FDH-I-NEVTL----T-E---
SHESA02025      ----------DLTGPALMERMQKHAEKF-DT--EI--L-FDH-I-NEVTL----T-E---
SHESW01953      ----------DLTGPALMERMQKHAEKF-DT--EI--L-FDH-I-NEVTL----T-E---
SHEPC01976      ----------DLTGPALMERMQKHAEKF-DT--EI--L-FDH-I-NEVTL----T-E---
SHEP201929      ----------DLTGPALMERMQKHAEKF-DT--EI--L-FDH-I-NEVTL----T-E---
SHEB502016      ----------DLTGPALMERMQKHAEKF-DT--EI--L-FDH-I-NEVTL----T-E---
SHEB802168      ----------DLTGPALMERMQKHAEKF-DT--EI--L-FDH-I-NEVTL----T-E---
SHEB202117      ----------DLTGPALMERMQKHAEKF-DT--EI--L-FDH-I-NEVTL----T-E---
SHEB902270      ----------DLTGPALMERMQKHAEKF-DT--EI--L-FDH-I-NEVTL----T-E---
SHEB602258      ----------DLTGPALMERMQKHAEKF-DT--EI--L-FDH-I-NEVTL----T-E---
SHEDO01680      ----------DLTGPALMERMQKHAEKF-DT--EI--L-FDH-I-NEVTL----T-E---
SHEFN01895      ----------DLTGPALMDRMQKHAEKF-DT--EI--I-FDH-I-NEVTL----T-E---
PSEHT01677      ----------GLTGPALMDRMKEHAERF-ET--EI--I-FDH-I-NKVDV----S-K---
ALISL01611      ----------DMTGPGLMEEMKAHAERF-NT--EI--I-FDH-I-NETDF----S-Q---
VIBF100893      ----------DMTGPGLMEEMKAHAERF-NT--EI--I-FDH-I-NETDF----S-Q---
VIBFM00891      ----------DMTGPGLMEEMKAHAERF-NT--EI--I-FDH-I-NETDF----S-Q---
OCESG01399      ----------GLTGPALMERMKAHAERF-ET--EI--V-FDH-I-HTVEL----T-Q---
VIBA701872      ----------GLTGPALMERMKEHAERF-ET--EI--V-FDH-I-NEVDF----S-Q---
VIBVY01451      ----------GLTGPGLMNRMKEHAERF-ET--EI--I-FDH-I-NEVDF----S-T---
VIBVU02545      ----------GLTGPGLMDRMKEHAERF-ET--EI--I-FDH-I-NEVDF----S-T---
```

```
VIBVM01748      ----------GLTGPGLMDRMKEHAERF-ET--EI--I-FDH-I-NEVDF----S-T---
VIBCH01157      ----------GLTGPALMERMKEHAERF-DT--EI--V-FDH-I-NSVDL----S-S---
VIBCM01114      ----------GLTGPALMERMKEHAERF-DT--EI--V-FDH-I-NSVDL----S-S---
VIBCJ02064      ----------GLTGPALMERMKEHAERF-DT--EI--V-FDH-I-NSVDL----S-S---
VIBC300725      ----------GLTGPALMERMKEHAERF-DT--EI--V-FDH-I-NSVDL----S-S---
FERBD01969      ----------GLTGPGLMERMKAHAERF-ET--EI--V-FDH-I-NEVDL----K-S---
VIBFN02085      ----------GLTGPALMERMKEHAKRF-ET--EI--V-FDH-I-NQVDF----S-Q---
VIBTL01089      ----------GLTGPALMDRMKEHAERF-ET--EI--L-FDH-I-NEVDL----S-N---
VIBPA01248      ----------GLTGPALMERMKEHAERF-ET--EI--V-FDH-I-NEVEL----S-Q---
VIBAE02128      ----------GLTGPALMDRMKEHAERF-ET--EI--V-FDH-I-NEVEL----S-Q---
VIBCB00599      ----------GLTGPGLMERMKEHAERF-ET--EI--V-FDH-I-NEVEL----S-Q---
PANVC00701      ----------DLTGPSLMERMHEHAEKF-NT--EI--I-FDH-I-HTVDL----Q-N---
SHIBC02443      ----------DLTGPLLMERMHEHALKF-DT--EI--I-TDH-I-NSVDL----Q-Q---
ENTBF02792      ----------DLTGPLLMERMHEHATKF-ET--EI--L-FDH-I-TSVDL----Q-N---
KLEP700892      ----------DLTGPLLMERMHEHAAKF-ET--EI--I-FDH-I-SRVDL----Q-N---
KLEPH01782      ----------DLTGPLLMERMHEHAAKF-ET--EI--I-FDH-I-SRVDL----Q-N---
KLEP303551      ----------DLTGPLLMERMHEHAAKF-ET--EI--I-FDH-I-SRVDL----Q-N---
KLEVT03420      ----------DLTGPLLMERMHEHAAKF-ET--EI--I-FDH-I-SRVDL----Q-N---
ENTAK02985      ----------DLTGPLLMERMHEHATKF-ET--EI--I-FDH-I-SSVDL----Q-N---
KLEOK03151      ----------DLTGPLLMERMHEHATKF-ET--EI--I-FDH-I-NSVDL----Q-N---
SALAR01925      ----------DLTGPLLMERMHEHAEKF-AT--EI--I-FDH-I-NSVDL----Q-N---
SALBC00812      ----------DLTGPLLMERMHEHATKF-ET--EI--I-FDH-I-NSVDL----Q-N---
SALPC00926      ----------DLTGPLLIERMHEHAAKF-ET--EI--I-FDH-I-NNVDL----Q-N---
SALTI00868      ----------DLTGPLLMERMHEHAAKF-ET--EI--I-FDH-I-NNVDL----Q-N---
SALCH00905      ----------DLTGPLLMERMHEHAAKF-ET--EI--I-FDH-I-NNVDL----Q-N---
SALPA01706      ----------DLTGPLLMERMHEHAAKF-ET--EI--I-FDH-I-NNVDL----Q-N---
SALTY00927      ----------DLTGPLLMERMHEHAAKF-ET--EI--I-FDH-I-NNVDL----Q-N---
SALPK01786      ----------DLTGPLLMERMHEHAAKF-ET--EI--I-FDH-I-NNVDL----Q-N---
SALHS00978      ----------DLTGPLLMERMHEHAAKF-ET--EI--I-FDH-I-NNVDL----Q-N---
SALEP00854      ----------DLTGPLLMERMHEHAAKF-ET--EI--I-FDH-I-NNVDL----Q-N---
SALDC00926      ----------DLTGPLLMERMHEHAAKF-ET--EI--I-FDH-I-NNVDL----Q-N---
SALA400892      ----------DLTGPLLMERMHEHAAKF-ET--EI--I-FDH-I-NNVDL----Q-N---
SALG200876      ----------DLTGPLLMERMHEHAAKF-ET--EI--I-FDH-I-NNVDL----Q-N---
SALTS00888      ----------DLTGPLLMERMHEHAAKF-ET--EI--I-FDH-I-NNVDL----Q-N---
SALT400905      ----------DLTGPLLMERMHEHAAKF-ET--EI--I-FDH-I-NNVDL----Q-N---
SALPS01908      ----------DLTGPLLMERMHEHAAKF-ET--EI--I-FDH-I-NNVDL----Q-N---
SALT101040      ----------DLTGPLLMERMHEHAAKF-ET--EI--I-FDH-I-NNVDL----Q-N---
SALTD00960      ----------DLTGPLLMERMHEHAAKF-ET--EI--I-FDH-I-NNVDL----Q-N---
SALPB02472      ----------DLTGPLLMERMHEHAAKF-ET--EI--I-FDH-I-NKVDL----Q-N---
SALNS00928      ----------DLTGPLLMERMHEHAAKF-ET--EI--I-FDH-I-NKVDL----Q-N---
SALSV01000      ----------DLTGPLLMERMHEHAAKF-ET--EI--I-FDH-I-NKVDL----Q-N---
ECOS500812      ----------DLTGPLLMERMHEHATKF-ET--EI--I-FDH-I-NKVDL----Q-N---
ECOL600981      ----------DLTGPLLMERMHEHATKF-ET--EI--I-FDH-I-NKVDL----Q-N---
ECOL500883      ----------DLTGPLLMERMHEHATKF-ET--EI--I-FDH-I-NKVDL----Q-N---
ECOUT00876      ----------DLTGPLLMERMHEHATKF-ET--EI--I-FDH-I-NKVDL----Q-N---
ECOK100776      ----------DLTGPLLMERMHEHATKF-ET--EI--I-FDH-I-NKVDL----Q-N---
ECOSM02148      ----------DLTGPLLMERMHEHATKF-ET--EI--I-FDH-I-NKVDL----Q-N---
ECOLU01057      ----------DLTGPLLMERMHEHATKF-ET--EI--I-FDH-I-NKVDL----Q-N---
ECO7I02136      ----------DLTGPLLMERMHEHATKF-ET--EI--I-FDH-I-NKVDL----Q-N---
ECO8100828      ----------DLTGPLLMERMHEHATKF-ET--EI--I-FDH-I-NKVDL----Q-N---
ECO4500862      ----------DLTGPLLMERMHEHATKF-ET--EI--I-FDH-I-NKVDL----Q-N---
ECOAB00879      ----------DLTGPLLMERMHEHATKF-ET--EI--I-FDH-I-NKVDL----Q-N---
ECO4400961      ----------DLTGPLLMERMHEHATKF-ET--EI--I-FDH-I-NKVDL----Q-N---
ECOUM02550      ----------DLTGPLLMERMHEHATKF-ET--EI--I-FDH-I-NKVDL----Q-N---
ECOKI00869      ----------DLTGPLLMERMHEHATKF-ET--EI--I-FDH-I-NKVDL----Q-N---
ECOC100920      ----------DLTGPLLMERMHEHATKF-ET--EI--I-FDH-I-NKVDL----Q-N---
ECOC200920      ----------DLTGPLLMERMHEHATKF-ET--EI--I-FDH-I-NKVDL----Q-N---
ECO8N00803      ----------DLTGPLLMERMHEHATKF-ET--EI--I-FDH-I-NKVDL----Q-N---
ECO2700862      ----------DLTGPLLMERMHEHATKF-ET--EI--I-FDH-I-NKVDL----Q-N---
ECO2600974      ----------DLTGPLLMERMHEHATKF-ET--EI--I-FDH-I-NKVDL----Q-N---
ECOH100942      ----------DLTGPLLMERMHEHATKF-ET--EI--I-FDH-I-NKVDL----Q-N---
SHIB301848      ----------DLTGPLLMERMHEHATKF-ET--EI--I-FDH-I-NKVDL----Q-N---
ECOLI00846      ----------DLTGPLLMERMHEHATKF-ET--EI--I-FDH-I-NKVDL----Q-N---
ECO5700970      ----------DLTGPLLMERMHEHATKF-ET--EI--I-FDH-I-NKVDL----Q-N---
SHISS00756      ----------DLTGPLLMERMHEHATKF-ET--EI--I-FDH-I-NKVDL----Q-N---
```

```
SHIBS00679      ----------DLTGPLLMERMHEHATKF-ET--EI--I-FDH-I-NKVDL----Q-N---
SHIDS01857      ----------DLTGPLLMERMHEHATKF-ET--EI--I-FDH-I-NKVDL----Q-N---
ECO2400906      ----------DLTGPLLMERMHEHATKF-ET--EI--I-FDH-I-NKVDL----Q-N---
ECODH00784      ----------DLTGPLLMERMHEHATKF-ET--EI--I-FDH-I-NKVDL----Q-N---
ECOHS00922      ----------DLTGPLLMERMHEHATKF-ET--EI--I-FDH-I-NKVDL----Q-N---
ECOLC02627      ----------DLTGPLLMERMHEHATKF-ET--EI--I-FDH-I-NKVDL----Q-N---
ECO5E00946      ----------DLTGPLLMERMHEHATKF-ET--EI--I-FDH-I-NKVDL----Q-N---
ECOSE00936      ----------DLTGPLLMERMHEHATKF-ET--EI--I-FDH-I-NKVDL----Q-N---
ECO5500894      ----------DLTGPLLMERMHEHATKF-ET--EI--I-FDH-I-NKVDL----Q-N---
ECO8A00877      ----------DLTGPLLMERMHEHATKF-ET--EI--I-FDH-I-NKVDL----Q-N---
ECOBB00863      ----------DLTGPLLMERMHEHATKF-ET--EI--I-FDH-I-NKVDL----Q-N---
ECO5T00943      ----------DLTGPLLMERMHEHATKF-ET--EI--I-FDH-I-NKVDL----Q-N---
ECOBW00723      ----------DLTGPLLMERMHEHATKF-ET--EI--I-FDH-I-NKVDL----Q-N---
ECO1000919      ----------DLTGPLLMERMHEHATKF-ET--EI--I-FDH-I-NKVDL----Q-N---
ECOBD02604      ----------DLTGPLLMERMHEHATKF-ET--EI--I-FDH-I-NKVDL----Q-N---
ECOD102659      ----------DLTGPLLMERMHEHATKF-ET--EI--I-FDH-I-NKVDL----Q-N---
ECOBR00856      ----------DLTGPLLMERMHEHATKF-ET--EI--I-FDH-I-NKVDL----Q-N---
ECOLX02610      ----------DLTGPLLMERMHEHATKF-ET--EI--I-FDH-I-NKVDL----Q-N---
ECO1A00939      ----------DLTGPLLMERMHEHATKF-ET--EI--I-FDH-I-NKVDL----Q-N---
ECOCB01054      ----------DLTGPLLMERMHEHATKF-ET--EI--I-FDH-I-NKVDL----Q-N---
ECOKO02859      ----------DLTGPLLMERMHEHATKF-ET--EI--I-FDH-I-NKVDL----Q-N---
ECO1E03320      ----------DLTGPLLMERMHEHATKF-ET--EI--I-FDH-I-NKVDL----Q-N---
ECOLW01217      ----------DLTGPLLMERMHEHATKF-ET--EI--I-FDH-I-NKVDL----Q-N---
SHIFL01399      ----------DLTGPLLMARMHEHATKF-ET--EI--I-FDH-I-NKVDL----Q-N---
SHIF800759      ----------DLTGPLLMARMHEHATKF-ET--EI--I-FDH-I-NKVDL----Q-N---
SHIF200805      ----------DLTGPLLMARMHEHATKF-ET--EI--I-FDH-I-NKVDL----Q-N---
CITK802118      ----------DLTGPLLMERMHEHATKF-DT--EI--I-FDH-I-NSVDL----Q-N---
CITRI00924      ----------DLTGPLLMERMHEHATKF-ET--EI--I-FDH-I-NKVDL----Q-N---
CROS802385      ----------DLTGPLLMERMHEHAAKF-ET--EI--L-FDH-I-NKVDL----Q-N---
CROTZ01500      ----------DLTGPLLMERMHEHAAKF-ET--EI--L-FDH-I-NKVDL----Q-N---
ENTLS02865      ----------DLTGPLLMERMHEHATKF-DT--EI--I-FDH-I-SKVDL----Q-N---
ENT3801400      ----------DLTGPLLMERMHEHATKF-DT--EI--L-FDH-I-NKVDL----Q-N---
ENTAL01384      ----------DLTGPLLMERMHEHATKF-DT--EI--L-FDH-I-NKVDL----Q-N---
ENTCC02679      ----------DLTGPLLMERMHEHAAKF-ET--EI--L-FDH-I-NKVDL----Q-N---
                             .

STRT101547      ----KVV---------KT--GD----E----EYQTKTVIIATGAKHRH-IGVAG--EE--
STRT201508      ----KVV---------KT--GD----E----EYQTKTVIIATGAKHRH-IGVAG--EE--
STRTD01356      ----KVV---------KT--GD----E----EYQTKTVIIATGAKHRH-IGVAG--EE--
STRTN01533      ----KVV---------KT--GD----E----EYQTKTVIIATGAKHRH-IGVAG--EE--
STRE500345      ----KVV---------KT--GD----E----EYQTKTVIIATGAKHRH-IGVAG--EE--
STRE801625      ----KVV---------KT--GD----E----EYQTKTVIIATGAKHRH-IGVAG--EE--
STREH01636      ----KVV---------KT--GD----E----EYQTKTVIIATGAKHRH-IGVAG--EE--
STREC01656      ----KRV---------YT--ED----T----HYDTKTVIVATGAKHRL-LGVAG--EE--
STREM01495      ----KRV---------YT--ED----T----HYDTKTVIVATGAKHRL-LGIAG--EE--
STRE401629      ----KRV---------YT--ED----T----HYDTKTVIVATGAKHRL-LGVAG--EE--
STRS700387      ----KRV---------YT--ED----T----HYDTKTVIVATGAKHRL-LGVAG--EE--
STRDG01631      ----KRV---------ST--ED----D----SYEAKTIIIATGAKYRV-LGVPG--EE--
STRP301388      ----KCV---------LT--ED----A----SYEAKTVIIATGAKYRV-LGVPG--EE--
STRPZ01205      ----KCV---------LT--ED----A----SYEAKTVIIATGAKYRV-LGVPG--EE--
STRPQ00464      ----KCV---------LT--ED----A----SYEAKTVIIATGAKYRV-LGVPG--EE--
STRPD01453      ----KCV---------LT--ED----A----SYEAKTVIIATGAKYRV-LGVPG--EE--
STRP601399      ----KCV---------LT--ED----A----SYEAKTVIIATGAKYRV-LGVPG--EE--
STRP801366      ----KCV---------LT--ED----A----SYEAKTVIIATGAKYRV-LGVPG--EE--
STRPF01453      ----KCV---------LT--ED----A----SYEAKTVIIATGAKYRV-LGVPG--EE--
STRPG00427      ----KCV---------LT--ED----A----SYEAKTVIIATGAKYRV-LGVPG--EE--
STRA300284      ----KRV---------IT--ED----E----SYEAKTVILATGAKNSL-LGVPG--EE--
STRA500290      ----KRV---------IT--ED----E----SYEAKTVILATGAKNSL-LGVPG--EE--
STRA100288      ----KRV---------IT--ED----E----SYEAKTVILATGAKNSL-LGVPG--EE--
STRA200291      ----KRV---------IT--ED----E----SYEAKTVILATGAKNSL-LGVPG--EE--
STRIC00348      ----KRV---------IT--ED----A----TYEAKTIILATGAKNRS-LGVAG--EE--
STRPX00375      ----KRV---------IT--ED----T----SYEAKTIILATGAKYRT-LDVPG--EE--
STRMD00432      ----KRV---------IT--ED----A----SYEAKTIILATGAKYRT-LDVPG--EE--
STRS201734      ----KKV---------IT--ED----G----VLETKTVVLAMGAKHRL-LGIPG--ED--
STRSY01721      ----KKV---------IT--ED----G----VLETKTVVLAMGAKHRL-LGIPG--ED--
```

```
STRSX01553      ----KKV---------IT--ED----G----VLETKTVVLAMGAKHRL-LGIPG--ED--
STRSE01478      ----KKV---------IT--ED----G----VLETKTVVLAMGAKHRL-LGIPG--ED--
STREJ01635      ----KKV---------IT--ED----G----VLETKTVVLAMGAKHRL-LGIPG--ED--
STRGZ01544      ----KKV---------IT--ED----G----VLETKTVVLAMGAKHRL-LGIPG--ED--
STRS401593      ----KKV---------IT--ED----G----VLETKTVVLAMGAKHRL-LGIPG--ED--
LACGT00720      ----KTI---------NT--EE----E----VFETKSIIIATGANHRK-LGVPG--EE--
LACGL00738      ----KTI---------NT--EE----E----VFETKSIIIATGANHRK-LGVPG--EE--
STRSV01774      ----KEI---------IT--ED----E----RFEAKTVIIASGANHRH-LGVPG--EE--
STRIJ00350      ----KKV---------YT--ED----N----VFESKTLIIATGAFHRH-LGVPG--EE--
STROU01248      ----KKV---------IT--DD----Q----VYETRTVIVATGSKHRL-LGVPG--EE--
STRM601286      ----KKV---------MT--DD----Q----TYETRTVIVATGSKHRP-LGVPG--EE--
STRES00962      ----KKV---------MT--DD----Q----TYETRTVIVATGSKHRP-LGVPG--EE--
STRP701387      ----KKV---------MT--DD----Q----TYETRTVIVATGSKHRP-LGVLG--EE--
STRZT00756      ----KKV---------MT--DD----Q----TYETRTVIVATGSKHRP-LGVPG--EE--
STRP001079      ----KKV---------MT--DD----Q----TYETRTVIVATGSKHRP-LGVPG--EE--
STRZO01270      ----KKV---------MT--DD----Q----TYETRTVIVATGSKHRP-LGVPG--EE--
STRZ600815      ----KKV---------MT--DD----Q----TYETRTVIVATGSKHRP-LGVPG--EE--
STRET00826      ----KKV---------MT--DD----Q----TYETRTVIVATGSKHRP-LGVPG--EE--
STRPS01429      ----KKV---------MT--DD----Q----TYETRTVIVATGSKHRP-LGVPG--EE--
STRZN01287      ----KKV---------MT--DD----Q----TYETRTVIVATGSKHRP-LGVPG--EE--
STRR601306      ----KKV---------MT--DD----Q----TYETRTVIVATGSKHRP-LGVPG--EE--
STRP201217      ----KKV---------MT--DD----Q----TYETRTVIVATGSKHRP-LGVPG--EE--
STRZP01342      ----KKV---------MT--DD----Q----TYETRTVIVATGSKHRP-LGVPG--EE--
STRZI01226      ----KKV---------MT--DD----Q----TYETRTVIVATGSKHRP-LGVPG--EE--
STRPN01360      ----KKV---------MT--DD----Q----TYETRTVIVATGSKHRP-LGVPG--EE--
STRP401357      ----KKV---------MT--DD----Q----TYETRTVIVATGSKHRP-LGVPG--EE--
STRZJ01282      ----KKV---------MT--DD----Q----TYETRTVIVATGSKHRP-LGVPG--EE--
STRPJ01336      ----KKV---------MT--DD----Q----TYETRTVIVATGSKHRP-LGVPG--EE--
STRPI01436      ----KKV---------MT--DD----Q----TYETRTVIVATGSKHRP-LGVPG--EE--
MARHT00044      ----FL----------VR-GFE----R----TYRAKAVIVASGANPRR-LGVPG--ED--
THEP300413      ----KK----------VK-TSN----K----TYEAKAIILAMGATPKK-LGVPN--ED--
THEPX00884      ----KK----------VK-TSN----K----TYEAKAIILAMGATPKK-LGVPN--ED--
THESX01857      ----KK----------VK-TSN----K----TYEAKAIILAMGATPKK-LGVPN--ED--
THEM301788      ----KK----------VK-TSN----K----TYEAKAIILAMGATPKK-LGVPN--ED--
THEIA01812      ----KK----------VK-TSN----K----TYEAKAIILAMGATPKK-LGVPN--ED--
THETC00443      ----KK----------VT-TNK----K----TYEAKAIILATGATPKE-LGFDK--ER--
THESW01026      ----KK----------VT-TNK----K----TYEAKAIIIATGATPKE-LGFDK--ER--
THEXL00360      ----KK----------VT-TNK----K----TYEAKAIIIATGATPKE-LGFDK--ER--
THEID01127      ----KKL---------IL-ASG----N----ELLAQTVIIATGAKPNT-LGVPG--EK--
THEOJ00147      ----FL----------IR-LKT----E----TLEAKTVILAMGAEARK-LGVKG--EK--
DESAS01237      ----FA----------VK-TGS----G----ELYARAVILATGASPQL-LGVRG--ES--
DESK701373      ----FI----------VK-TED----T----EIAARTVIVATGAQPQR-LNVRG--EE--
KYRT200729      ----HRV---------HL-SGG----E----VLETKTVIIATGCEPKK-LGIPG--EK--
STACT00410      ----KKI---------VY--GD----K----SVTAYAVIITTGAEYKK-IGVPG--EE--
STAS101952      ----KEV---------NL--GN----K----VITAHAVIISTGAEYKK-IGVPG--EQ--
STALH01969      ----KVI---------HL--GN----K----EVTARAVIIATGAEYKK-IGVPG--EQ--
STAEQ00422      ----KVI---------NL--GN----K----EITAHAVIISTGAEYKK-IGVPG--EQ--
STAES00543      ----KVI---------NL--GN----K----EITAHAVIISTGAEYKK-IGVPG--EQ--
STAAB00714      ----KVI---------NF--GN----K----ELTAKAVIIATGAEYKK-IGVPG--EQ--
STAA500765      ----KVI---------NF--GN----K----ELTAKAVIIATGAEYKK-IGVPG--EQ--
STAAM00757      ----KVI---------NF--GN----K----ELTAKAVIIATGAEYKK-IGVPG--EQ--
STAAW00726      ----KVI---------NF--GN----K----ELTAKAVIIATGAEYKK-IGVPG--EQ--
STAAS00733      ----KVI---------NF--GN----K----ELTAKAVIIATGAEYKK-IGVPG--EQ--
STAAN00727      ----KVI---------NF--GN----K----ELTAKAVIIATGAEYKK-IGVPG--EQ--
STAAC00807      ----KVI---------NF--GN----K----ELTAKAVIIATGAEYKK-IGVPG--EQ--
STAA300727      ----KVI---------NF--GN----K----ELTAKAVIIATGAEYKK-IGVPG--EQ--
STAA800734      ----KVI---------NF--GN----K----ELTAKAVIIATGAEYKK-IGVPG--EQ--
STAA100752      ----KVI---------NF--GN----K----ELTAKAVIIATGAEYKK-IGVPG--EQ--
STAA200774      ----KVI---------NF--GN----K----ELTAKAVIIATGAEYKK-IGVPG--EQ--
STAA900759      ----KVI---------NF--GN----K----ELTAKAVIIATGAEYKK-IGVPG--EQ--
STAAE00716      ----KVI---------NF--GN----K----ELTAKAVIIATGAEYKK-IGVPG--EQ--
STAAT00767      ----KVI---------NF--GN----K----ELTAKAVIIATGAEYKK-IGVPG--EQ--
STAAD00695      ----KVI---------NF--GN----K----ELTAKAVIIATGAEYKK-IGVPG--EQ--
STAA000815      ----KVI---------NF--GN----K----ELTAKAVIIATGAEYKK-IGVPG--EQ--
STAAH02407      ----KVI---------NF--GN----K----ELTAKAVIIATGAEYKK-IGVPG--EQ--
```

STAAF00763	----KVI---------NF--GN----K----ELTAKAVIIATGAEYKK-IGVPG--EQ--
STAAK00744	----KVI---------NF--GN----K----ELTAKAVIIATGAEYKK-IGVPG--EQ--
STAAJ00703	----KVI---------NF--GN----K----ELTAKAVIIATGAEYKK-IGVPG--EQ--
STAAG00690	----KVI---------NF--GN----K----ELTAKAVIIATGAEYKK-IGVPG--EQ--
STAA400732	----KVI---------NF--GN----K----ELTAKAVIIATGAEYKK-IGVPG--EQ--
STAAR00788	----KVI---------NF--GN----K----ELTAKAVIIATGAEYKK-IGVPG--EQ--
LISSS02378	----KTV---------TA--GS----K----TYKARAIVIATGAEHRK-LGADG--EE--
LISIN02590	----KTV---------TA--GS----K----TYKARAIIIATGAEHRK-LGAAG--EE--
LISW602421	----KTV---------TA--GS----K----TYKARAIIIATGAEHRK-LGAAG--EE--
EXISA00870	----KIV---------HA--HN----R----DYKARAIILASGAQYKK-IGVPG--EE--
EXIS202358	----KTV---------HA--HN----K----DYHARAIIIASGAQYKK-IGVPG--EE--
EXIAB02180	----KTI---------HA--HN----K----DYYARAIIIASGAQYKK-IGVPG--EE--
OCEIH02462	----KLI---------KA--GS----K----EYYTRSLIIATGAQYKK-LGIEG--EE--
BACIE01058	----KTI---------VL--GN----G----EVKTRSIIIGTGAKYKH-LNVPG--EQ--
BACCJ03468	----KTI---------VA--GN----G----EYKARAIIITTGAKYKN-LGVPG--EK--
BACHD03507	----KLV---------KA--GN----K----EYKARAVIVATGAEYKK-LGVPG--EK--
BACPE03382	----KIV---------KA--GS----K----EYKARSVIVATGAEYKK-LGIPG--EK--
SOLSS00743	----KII---------IS--GK----K----QYKTRTIIITTGAEYKK-LGIPG--ET--
BACC600715	----KIV---------KA--GT----K----AYKALAVIIATGAQYKK-LGVKG--EN--
ANOFW02486	----KTV---------VT--SN----Q----QYKARAIIIATGAEYKK-LGVPG--EK--
GEOKA03042	----KTV---------IV--GD----K----EYKARAVIIATGAEYKK-LGVPG--EA--
GEOSY02976	----KTV---------IV--GD----K----EYKARAVIIATGAEYKK-LGVPG--EA--
GEOTN02952	----KTV---------VV--GD----K----EYKARAVIIATGAEYKK-LGVPG--EA--
GEOSW02588	----KTV---------VV--GD----K----QYKARAVIIATGAEYKK-LGVPG--EA--
GEOS000381	----KTV---------VV--GD----K----QYKGRAVIIATGAEYKK-LGVPG--EA--
GEOTC00378	----KTV---------VV--GD----K----QYKGRAVIIATGAEYKK-LGVPG--EA--
BACMD04906	----KTV---------RA--GS----K----EYKTRSVIVATGAEYKK-LGAPG--EK--
BACMQ04908	----KTV---------RA--GS----K----EYKTRSVIVATGAEYKK-LGAPG--EK--
BACWK04830	----KTI---------IA--GK----K----EYKARAIIVSSGAEYKK-IGVPG--ET--
BACAN04889	----KTI---------IA--GK----K----EYKARAIIVASGAEYKK-IGVPG--ET--
BACC105210	----KTI---------IA--GK----K----EYKARAIIVASGAEYKK-IGVPG--ET--
BACC705051	----KTI---------IA--GK----K----EYKARAIIVASGAEYKK-IGVPG--ET--
BACC005112	----KTI---------IA--GK----K----EYKARAIIVASGAEYKK-IGVPG--ET--
BACC305043	----KTI---------IA--GK----K----EYKARAIIVASGAEYKK-IGVPG--ET--
BACAC05232	----KTI---------IA--GK----K----EYKARAIIVASGAEYKK-IGVPG--ET--
BACAA04720	----KTI---------IA--GK----K----EYKARAIIVASGAEYKK-IGVPG--ET--
BACT005085	----KTI---------IA--GK----K----EYKARAIIVASGAEYKK-IGVPG--ET--
BACC205124	----KTI---------IA--GK----K----EYKARAIIVASGAEYKK-IGVPG--ET--
BACC405064	----KTI---------IA--GK----K----EYKARAIIVASGAEYKK-IGVPG--ET--
BACT104712	----KTI---------IA--GK----K----EYKARAIIVASGAEYKK-IGVPG--ET--
BACLD03769	----KIV---------KA--GS----K----EFKARAVIITAGAEYKK-LGVPG--EK--
BACPZ03376	----KVV---------KA--GS----K----EYKARAVIIAAGAEYKK-IGVPG--EK--
BACSU03600	----KVV---------KA--GS----K----EYKARAVIIAAGAEYKK-IGVPG--EK--
BACST01653	----KVV---------KA--GS----K----EYKARAVIIAAGAEYKK-IGVPG--EK--
BACPT03541	----KVV---------KA--GS----K----EYKARAVIIAAGAEYKK-IGVPG--EK--
LEUGG00661	----KII---------NT-DM-----G----RYIAKAIIIATGSEHVH-LGADG--EE--
LEUGJ00629	----KII---------NT-DM-----G----RYIAKAIIIATGSEHVH-LGADG--EE--
LEUCJ00485	----KIV---------HT-DM-----D----HYIAKAIIIGTGSEHIH-LGVTG--EE--
LACAR00657	----KVV---------KT-DDD----K----EYRAPILIIATGSDHRH-LNVPG--EE--
LACA300647	----KVV---------KT-DDD----K----EYRAPILIIATGSDHRH-LNVPG--EE--
LACAL00643	----KVV---------KT-DDD----K----EYRAPILIIATGSDHRH-LNVPG--EE--
LACKZ00964	----KVI---------KT-DDD----H----EYRAPILIIATGSDHRH-LGVPG--EE--
LACRJ00357	----KKI---------TT-DMD----E----TYTAKAVVIATGSDQRH-LNVPG--EE--
LACRD00361	----KKI---------TT-DMD----E----TYTAKAVVIATGSDQRH-LNVPG--EE--
LACRS01501	----KKI---------TT-DMD----E----TYTAKAVVIATGSDQRH-LNVPG--EE--
LACSM00455	----RIV---------KT-AD-----D----EYESSAVVIASGSEYRK-LGAPG--EK--
LACRG00878	----KLV---------KT-DE-----D----EFEAKAIVIATGAEHKK-LGVPG--EE--
LACRL00939	----KLV---------KT-DE-----D----EFEAKAIVIATGAEHKK-LGVPG--EE--
LACC300844	----KLV---------KT-DE-----D----EYEAKAIVIATGAEHKK-LGVPG--EE--
LACCZ00824	----KLV---------KT-DE-----D----EYEAKAIVIATGAEHKK-LGVPG--EE--
LACCB00988	----KLV---------KT-DE-----D----EYEAKAIVIATGAEHKK-LGVPG--EE--
LACCD01016	----KLV---------KT-DE-----D----EYEAKAIVIATGAEHKK-LGVPG--EE--
LACCC01014	----KLV---------KT-DE-----D----EYEAKAIVIATGAEHKK-LGVPG--EE--
LACBN01277	----KIV---------KT-DE-----D----EIQAKVVIIATGSEYKK-LGIPG--EH--
LACBA00605	----KIV---------KT-DD-----G----DYETKAVIVATGSEYKK-LGVPG--ED--

```
LACPL00650      ----KIV---------TT-D-S----D----TFETKALVIGTGSEYRK-LGVTG--ED--
LACPJ00628      ----KIV---------TT-D-S----D----TFETKALVIGTGSEYRK-LGVTG--ED--
LACPS00585      ----KIV---------TT-D-S----D----TFETKALVIGTGSEYRK-LGVTG--ED--
PEDCP00497      ----KTI---------TT-DMG----D----EFITKVLIIGTGSEYKK-LGVPG--EE--
CARS100359      ----KTI---------IA-GK-----K----IFKTRSIIIATGAEHRK-LSVGG--EN--
AERUA00263      ----HLI---------ET-DN-----G----DFKAKAIVIATGSVHRT-LDVPG--EE--
ELUMP00590      ----FKL---------TA-TDG----N----IYAAKTIVIASGVKPRW-TEADG--ED--
SPHPG02940      ----FIA---------T--TSG----E----TYQAKAVIIATGAKHRH-LGVEG--EE--
SPHGB01868      ----FLA---------ET-ADG----E----TYQAKAVIFATGANHRH-LGVEG--EE--
TREPZ00273      ----FTA---------VL-GSG----G----EIKARAVILATGATHRT-LGIPG--EA--
TREAZ03414      ----FSV---------TL-NKG----A----VRTAPALLIATGAKHRK-LGIPG--EE--
SPITD00734      ----FEV---------ET-TKG---------VLTSYAVILASGAAHRK-LGIPG--EK--
SPITZ01373      ----FEV---------ET-TKG---------VLTSYAVILASGAAHRK-LGIPG--EK--
TREPA00803      ----FHI---------TT-GTG---------AYTAMSVILATGAEHRK-MGIPG--ES--
TREPS00802      ----FHI---------TT-GTG---------AYTAMSVILATGAEHRK-MGIPG--ES--
TREPC00747      ----FHI---------TT-GTG---------AYTAMSVILATGAEHRK-MGIPG--ES--
TREPM00823      ----FHI---------TT-GTG---------AYTAMSVILATGAEHRK-MGIPG--ES--
TREPD00824      ----FHI---------TT-GTG---------AYTAMSVILATGAEHRK-MGIPG--ES--
TREPU00781      ----FHI---------TT-GTG---------ACTAMSVILATGAEHRK-MGIPG--ES--
ENCCU00216      ----FVV---------VS-EKG---------ERRARAVIVATGASARR-LFVPGTGDG--
HELM100964      ----FVI---------LR-NDG----K----EDEAKSVIITTGGSPKR-TGIKG--ES--
HELCP01490      ----FSI---------HL-ADK----S----TEQAKAVIFAAGGSPKR-ANLKG--EN--
ARCFU01526      ----FTV---------IA-EGG---------EYEAKAIIVATGGKHKE-AGIEG--ES--
FERPA02445      ----FVV---------KT-DMA---------EYKAKALIIATGGKHKE-LGVPG--EK--
ARCVS01910      ----FVV---------KT-DSG---------EYRVKAVIVATGGKHKE-LGVPG--EK--
METEZ00677      ----IVL---------ST-DEN----V----DIEAKAVIIATGAKPRK-LGIPG--ED--
METHD00869      ----KVV---------MT-DT-----G----NLVSKALVIATGANPKK-LGIPG--EK--
METMA02304      ----KII---------TT-DS-----G----DLEAKAVIIATGANPKH-LGVPG--EK--
METAC01311      ----KIV---------ST-DS-----G----DLEAKTLIIATGANPKH-LGVPG--EK--
KOSOT00298      ----KTV---------IL-DNG----K----KIQSRVLIIATGSNPRK-LNVPG--EA--
MARPK01613      ----KII---------SL-DNG----S----IVKAKTVVIATGATPRK-LGVPG--EM--
SLAHD02439      ----KKV---------IG-KKG---------EYCAKSIILATGAHSRP-IGCEN--EA--
FILAD00976      ----KTV---------EC-ENE---------VYETKTIIIATGTNTKN-IGCVG--EE--
BUTPB02463      KAPVYKV---------IT-DNG---------EFETHTVILATGAHHSK-LQIPG--EE--
CLOPH00250      --IKKIV---------LD-NGE---------EFYAKSLIIATGAASRK-LDVKG--EA--
CLOSW00592      --EFT-V---------VG-EEK---------TYATKTVIIATGAQHRK-LSVIG--EE--
LACFC00207      ----KSV---------TT-DMG----D----TFIAKAVVIATGSDQRK-LGAPG--EQ--
CRYCD00853      ----KTL---------TT-ANG---------QVSAKAVIIATGARSRK-LGIAG--EA--
EGGLE01847      ----KVL---------TT-AFN---------EYRAKSVIIATGARPRK-LGLEL--EE--
PYRFU01410      GPCKFTI---------KT-ANG----M----EYKSRTVIIAVGAEPRK-LNVPG--EK--
PYRHO01476      GPCNFVV---------KT-ANG----K----EYKAKTIIIAVGAEPRK-LNVPG--EK--
PYRAB00730      GPCQFEV---------KT-ANG----K----EYKGKTIIIAVGAEPRK-LHVPG--EK--
PYRSN00014      GPCQFTV---------KT-ANG----K----EYKSKTIIIAVGAEPRK-LNVPG--EK--
THEGJ00181      GPCKFTV---------KT-KNG----K----EYRAKTIIIAVGAAPRK-LKVPG--EE--
THEKO02097      GPCKFTV---------KT-ANG----K----EYKARTIIIAVGAAPRK-LRVPG--EE--
THEON01610      GPCKFVV---------RT-KNG----K----EYKGKTIIIAVGAAPRK-LNVPG--EA--
THES401476      GPCKFTV---------KT-KNG----K----EYKAKTIIITVGAAPRK-LHVPG--EE--
SYNWW02368      ----KTV---------VT-NMQ---------EYQTKSVIIAMGAKRRE-LEVDG--EK--
UNCTG00012      -----KV---------IT-ANS---------AYETKTVIIAAGTYAKK-MNIPG--ES--
THEA101460      ----FTV---------KS-DAG---------EFKGKTLIWAAGSTPRK-LGVPG--EV--
DENA201647      --KLVHV---------EN-RET---------PVKCKAVIITFGCEPKR-LDVPG--EN--
DEFDS00488      --KYVSL---------QY-KDI---------KIKTKTVIIASGAKPKH-LEVPG--EN--
CALNY01292      --KLIQL---------KH-SEL---------KIKTKSLIIAAGAKPKN-LNIPG--EP--
SYNGF01652      ----KKV---------IT-DKQ---------VYEAKAIIICAGSKPRF-LGVPG--EE--
DESB201123      --GRLLL---------HH-HGG---------QVTAKAVILAMGASPAN-LGIPG--EA--
DEIPM00845      AEYPFTV---------QG-FAG---------SYQGKSVILTTGAEPRR-LGVPG--EE--
DEIRA01924      HPYPFTV---------RG-YNG---------EYRAKAVILATGADPRK-LGIPG--ED--
DEIML01437      --HTFTI---------TG-YNG---------TYTAKAVILATGANPKR-LGIPG--EE--
DEIGD02532      HVYPFTI---------RG-YSG---------TYRAKAVILATGANPKR-LNVPG--EE--
DEIDV00622      PHYPFTV---------QG-YGG---------SYRAKAVILATGANPKR-LGIPG--EE--
TRURR01079      --GGFLV---------TG-YDA---------QYHARAVIIATGANPRR-LGVPG--ED--
THETG01863      --GGYLV---------RG-YER---------NYRARAVIVATGANPRR-LGVPG--ED--
THET201543      --GGYLV---------RG-YER---------NYRARAVIVATGANPRR-LGVPG--ED--
THET801911      --GGYLV---------RG-YER---------NYRARAVIVATGANPRR-LGVPG--ED--
OCEP502115      --EGFLV---------KA-FER---------NYRARSVIIATGANPRK-LGVPG--ED--
```

```
DEHLB00585      ----KVV---------KT-RLG---------DYQAKALIIAGGSERRK-LGVPG--EK--
DEHMG00439      ----FEV---------VT-ETG---------GLSGRCIIICGGTERNK-LEVPG--EE--
DEHMB00497      ----FEV---------VT-ETG---------GLSGRCIIICGGTERNK-LEVPG--EE--
DEHMC00427      ----FEV---------VT-ETG---------GLSGRCIIICGGTERNK-LEVPG--EE--
THELD00726      ----KLV---------VT-DNG---------TIEAEAIIVATGAKFKK-LGVPG--EE--
ANAMD00624      ----KII---------VT-DKG---------DIEAESIVIATGARFRK-LGCPG--EA--
THEAS00815      ----KVV---------VT-DKG---------EVEAEAVIIATGANFKR-LGCPG--EA--
AMICL00707      ----KVV---------VT-DKG---------EIEAEAIILATGAYFRK-LGCEG--EV--
CLOCE01684      ----KKV---------KT-KNN---------TFESKTIILSMGASPKM-LGLPK--EE--
HYDS000616      ---IKIL---------KL-DNG----S----ELRAKTVIITTGARMRT-LDVPG--EK--
HYDTT00224      ----VFV---------HL-RDG----R----MLRAKTLIVASGSNPRK-LGVPG--ED--
PELTS01405      ---F-CV---------KT-EDG---------ELLAGAVIIATGAQPQL-LGVKG--EE--
DESRL02179      ---F-IV---------HT-TQG---------SIGTKAVILATGAKPKF-LGVEG--EG--
BORBP00506      ---IFYL---------YT--EN----Y----IYKSKAVIIAVGSKPKKLETLKN--SD--
BORAP00518      ---IFYL---------YT--EN----Y----VYKSKAVIIAVGSKPKKLESLKN--SD--
BORBU00514      ---IFYL---------YT--EN----Y----IYKSKAVIIAVGSKPKKLETLKN--SG--
BORBZ00490      ---IFYL---------YT--EN----Y----IYKSKAVIIAVGSKPKKLETLKN--SG--
BORBN00493      ---IFYL---------YT--EN----Y----IYKSKAVIIAVGSKPKKLETLKN--SG--
BORRA00486      ---IFCL---------YT--DN----Y----IYKSKSIVIASGSIPKKLDTLKN--LD--
BORDL00498      ---IFCL---------YT--DN----Y----IYKSKSIVIAAGSIPKKLDTLKN--LD--
BORHD00497      ---IFYI---------FT--DN----Y----IYKSRAVIIAAGSVPKKLDTLKN--SD--
BORT900497      ---IFYL---------FT--DN----Y----IYKSKAVIIAAGSVPKKLNTLKN--SD--
METKA01560      ---TFRV---------LD--------------EECRAVVIATGARPKR-LGVPG--ER--
MYCA500361      --FDKTV---------IM-ESG----E----EYHAKAIIIATGMKNRIPSDIVN--IE--
METVS00145      ---PYKL---------TT--KN----G----IYEANSIIIASGSHYKN-VGI-N--ED--
METOI01431      ---PFKI---------IG--EN----N----TYITKSIIIATGTKDKK-LGL-N--ED--
MYCHN00051      ---TWKI---------RT-ANN----Q----LFRSKSILIASGMRERV-LDIDN--VK--
MYCSL00465      ---SWTI---------ET-KK-----S----SFKSRAILIATGMRERK-LEIEN--ET--
MYCS300420      ---SWTI---------ET-KK-----S----SFKSRAILIATGMRERK-LEIEN--ET--
DESK101258      --KTWCV---------ELR-SG----G----SICGYAIIIAVGSEKRK-LNVPG--EK--
STAHD01472      --NLWCV---------ELRTTR----R----TICGYTVIYAIGSEKRK-LGVPG--ED--
THEC100270      --KLWCV---------KTV-SE----K----ELCGYAVIIAVGSEKRK-LNVKG--EE--
NANEQ00478      --N-FIV---------IT-EGG----Y----ELEAKAIILATGSRRRK-LNVKG--EN--
CALLD01225      --E-FHI---------TG-SNG----L----DVYSKTIILAVGSKRRK-LNVPG--ED--
SULSO02155      --E-FVV---------KT-KRK----G----EFKADSVILGIGVKRRK-LGVPG--EQ--
SULS900210      --E-FVV---------KT-KRK----G----EFKADSVILGIGVKRRK-LGVPG--EQ--
SULIA00208      --G-FVV---------KT-KRK----G----EFKADSVILGIGVKRRK-LGVPG--EQ--
SULIM00207      --G-FVV---------KT-KRK----G----EFKADSVILGIGVKRRK-LGVPG--EQ--
SULIK00225      --G-FVV---------KT-KRK----G----EFKADSVILGIGVKRRK-LGVPG--EQ--
IGNH400907      KEEMKVV---------RT-HKG----R----EYKAPAVIIATGVSTKG-LGVKG--EK--
KORCO01040      ---LKRV---------YT-SSG----K----EFQARALIIATGAEERK-LGIPG--EG--
THESM01133      GPCKFEV---------KT-KNG----K----VYKARSVIIAVGAEPRK-LKVPG--ED--
THEBM01534      GKCYWMV---------YT-KNG----R----VYKAKTVIIAVGAEPRK-LNIPG--EK--
METST01363      --GTFEI---------KT--TN----D----IIDTRYILIATGSSYKT-LDCVG--VD--
METTH00703      ---VFTV---------KT--SR----D----TYTASAIIFATGSKHRQ-LGVPG--EN--
METSL02406      --GKFKI---------ES--SK----N----EYLTKSLIFCSGTTYRK-IGVAG--EE--
METLA02406      --GKFKI---------ES--SK----N----EYLTKSLIFCSGTTYRK-IGVAG--EE--
METPW00194      --GKIMV---------TT--NK----N----EYAAKSLIISTGTKYRE-LGVPG--EQ--
RUBXD00226      --RPFRL---------WA-EGQE---E----PVLARAVIVATGAKARW-LGLP--GEQ--
RHOM400178      --RPFRL---------LV-DD-Q---T----PVLADAVIIATGASAKY-LGLE--NER--
GARV400051      --AVKLV---------TT-DD-G---I----TYESDAVIVSTGSQVRK-LGVP--GEL--
BIFAP01644      --DIKRI---------TT-DG-G---E----EYQTRAVVITTGSQYRK-LEIP--GER--
SEGRD00014      --DRKLV----------H-VD-G---E----VHAARAVILAMGAAPRY-LGVP--GEQ--
GORB404535      --EVKEV----------E-VA-G---E----VYRARAVILAMGAAARY-LGVE--GEQ--
GORPV04920      --DIKEV----------V-AS-G---E----THRARAVILAMGAAARY-LGIE--GEQ--
MYCA904903      --PVKTV----------T-VG-D---E----VYRARAVILAMGAAPRY-LGVP--GED--
MYCSS05356      --PVKTV----------T-VG-D---E----THRARAVILAMGAAARH-LGVP--GED--
MYCSJ05684      --PVKTV----------T-VG-D---E----THRARAVILAMGAAARH-LGVP--GED--
MYCSK05387      --PVKTV----------T-VG-D---E----THRARAVILAMGAAARH-LGVP--GED--
MYCS206581      --PVKTV----------V-VG-D---E----THQARAVILAMGAAARH-LGVP--GEE--
MYCCN05158      --PIKTV----------T-VG-D---E----TYQSRAVILAMGAAARH-LGVP--GED--
MYCVP05890      --PVKSV----------T-VG-D---E----TFRARAVILAMGAAARH-LGVP--GED--
MYCGI00775      --PVKTV----------T-VG-D---E----THSARAVILAMGAAARH-LGVP--GED--
MYCSR04994      --PVKTV----------T-VG-D---E----NHSARAVILAMGAAARH-LGVP--GED--
AMYSD04545      --DVKI----------VS-AG-G---V----DYRARAVILAMGAAARY-LGVA--GEE--
```

```
MYCLE02681       --PIKSV---------VT-AE-G---Q----TYQARAVILAMGTSVRY-LQIP--GEQ--
MYCLB02681       --PIKSV---------VT-AE-G---Q----TYQARAVILAMGTSVRY-LQIP--GEQ--
MYCSD04323       --PVKSV---------VT-AD-G---E----THRARAVILAMGAAARY-LNIP--GEQ--
MYCPA04306       --PVKSV---------TT-AE-G---E----TVRARAVILAMGAAARY-LGVP--GEQ--
MYCA105023       --PVKSV---------TT-AE-G---E----TVRARAVILAMGAAARY-LGVP--GEQ--
MYCUA04107       --PIKSV---------VT-SD-G---E----THRARAVILAMGAAARY-LHVP--GEQ--
MYCMM05378       --PIKSV---------VT-SE-G---E----THRARAVILAMGAAARY-LHVP--GEQ--
MYCA003905       --PLKSV---------VT-AD-G---Q----THRARAVILAMGAAARY-LQVP--GEQ--
MYCTU03943       --PLKSV---------VT-AD-G---Q----THRARAVILAMGAAARY-LQVP--GEQ--
MYCTF03846       --PLKSV---------VT-AD-G---Q----THRARAVILAMGAAARY-LQVP--GEQ--
MYCTA03980       --PLKSV---------VT-AD-G---Q----THRARAVILAMGAAARY-LQVP--GEQ--
MYCTK04010       --PLKSV---------VT-AD-G---Q----THRARAVILAMGAAARY-LQVP--GEQ--
MYCTC03612       --PLKSV---------VT-AD-G---Q----THRARAVILAMGAAARY-LQVP--GEQ--
MYCTD03548       --PLKSV---------VT-AD-G---Q----THRARAVILAMGAAARY-LQVP--GEQ--
MYCCP03898       --PLKSV---------VT-AD-G---Q----THRARAVILAMGAAARY-LQVP--GEQ--
MYCBO02863       --PLKSV---------VT-AD-G---Q----THRARAVILAMGAAARY-LQVP--GEQ--
MYCBP03913       --PLKSV---------VT-AD-G---Q----THRARAVILAMGAAARY-LQVP--GEQ--
MYCBT03913       --PLKSV---------VT-AD-G---Q----THRARAVILAMGAAARY-LQVP--GEQ--
TSUPD04078       --EIKEV----------V-TS-E---G----SYRARAVILAMGAAARY-LGIP--GEE--
ACTMD06897       --DVKYV----------T-AN-G---T----RYAAKAVVLAMGAAARY-LNVP--GEQ--
SACES08402       --EVKYV----------T-AN-G---T----RYAAKTVVLAMGAAARY-LHVP--GEQ--
SACVD03814       --PIKYV----------T-AH-G---K----RYAAHAVILAMGAAARH-LHVP--GEQ--
AMYMU09190       --DVKYV----------H-AN-G---K----RYAARAVILAMGAAARY-LNVP--GEQ--
AMYMS10178       --DVKYV----------H-AN-G---K----RYAARAVILAMGAAARY-LNVP--GEQ--
PSEUX06421       --PVKSV----------T-AH-G---E----TYRARAVILATGAAARY-LGVP--GEQ--
NOCFA05681       --PVKKV----------T-VG-D---E----TYEAYAVILAMGSAARY-LNVP--GEE--
NOCCG05476       --PVKKV----------T-VG-D---E----TFEAYAVILAMGSAARY-LGVP--GEQ--
RHOE406010       --PIKTV----------V-AN-G---E----TYAAHAIILAMGAAARY-LGIP--GEE--
RHOOB03468       --PIKKV----------V-VN-G---E----TYLAHAVVLAMGAAARY-LGIP--GEE--
RHOE104515       --PVKKV----------V-AN-G---E----TYYARAVILAMGAAARY-LGIP--GEE--
CORDI02302       --DIKKI----------W-VD-D---Q----EFQARTVILATGSAPRY-LGVE--GEQ--
CORD202219       --DIKKI----------W-VD-D---Q----EFQARTVILATGSAPRY-LGVE--GEQ--
CORDL02208       --DIKKI----------W-VD-D---Q----EFQARTVILATGSAPRY-LGVE--GEQ--
CORDJ02208       --DIKKI----------W-VD-D---Q----EFQARTVILATGSAPRY-LGVE--GEQ--
CORDH02221       --DIKKI----------W-VD-D---Q----EFQARTVILATGSAPRY-LGVE--GEQ--
CORD702316       --DIKKI----------W-VD-D---Q----EFQARTVILATGSAPRY-LGVE--GEQ--
CORD302337       --DIKKI----------W-VD-D---Q----EFQARTVILATGSAPRY-LGVE--GEQ--
CORDD02235       --DIKKI----------W-VD-D---Q----EFQARTVILATGSAPRY-LGVE--GEQ--
CORDV02170       --DIKKI----------W-VD-D---Q----EFQARTVILATGSAPRY-LGVE--GEQ--
CORDW02254       --DIKKI----------W-VD-D---Q----EFQARTVILATGSAPRY-LGVE--GEQ--
CORDK02230       --DIKKI----------W-VD-D---Q----EFQARTVILATGSAPRY-LGVE--GEQ--
COREF02870       --DIKKI----------W-VG-D---D----EYQARAVILSMGSAPRY-LGIP--GEK--
CORGL03082       --DIKKL----------W-VG-D---D----EYHARAVILSMGSAPRY-LGVK--GEQ--
CORGK02974       --DIKKL----------W-VG-D---D----EYHARAVILSMGSAPRY-LGVK--GEQ--
CORGB03038       --DIKKL----------W-VG-D---D----EYHARAVILSMGSAPRY-LGVK--GEQ--
CORK402006       --DIKIV----------R-SE-G---E----EYQARAVIIATGAAPRY-LGVK--GEQ--
CORJK02028       --EIKKV----------W-AY-G---E----EYQARTVILATGAAPRY-LHVP--GEQ--
CORVD02951       --EVKKV----------F-TE-D---E----EFHAKSVILVTGAAPRY-LGVP--GEE--
ARCHD01706       --DIKTV---------TT-A--D---A----TYQAKTVILALGSEYRK-LGLE--GEA--
THET101205       --RPFKV---------WT-D--E---E----EYSALSVIIATGASAKF-LGVP--GES--
PROAC02247       --EIKKL---------VD-TD-G---N----TYKAHVVILAMGSAYRK-LGLE--DEP--
PROAS02302       --EIKKL---------VD-TD-G---N----TYKAHVVILAMGSAYRK-LGLE--DEP--
CAERE29798       --DVKTI---------ET-GA-G---M----TYRARAVILTMGSAYRK-LGLP--EEE--
CELFA03749       --PVKTV---------TV-SG-G---E----TYAARAVILSTGSAYRE-LGLD--DEK--
JONDD02484       --DIKTI---------TT-AN-G---D----TYRAKAIILATGSAYRE-LGLD--DEK--
XYLCX03324       --DVKEI---------VT-GG-G---E----TYLARAVILATGSAYRE-LGLE--DEK--
ACIC102144       --PVKVV---------RT-D--E---G----EYRSRALIIATGSKYRY-LGLA--NEQ--
FRADG04042       --DPKIV---------TV-E--D---Q----TYRAHTVIIATGSAYRR-LGLP--DEE--
FRASU07065       --NPKVV---------KV-G--E---E----TYYAKTVILATGSAYRK-LGVP--DEG--
FRASN07115       --DPKVV---------KV-G--D---E----TYLARSVIVATGSAYKK-LGVE--HED--
FRASC04424       --NPKVV---------KI-E--D---R----TYLARTIIVATGSAYRM-LGVE--HEE--
FRAAA06712       --NPKVV---------RV-E--D---Q----TFLARTVIVATGSAYRK-LGVE--HEE--
KYTSD02521       --PVKTL---------RD-GS-G---T----TYRARTVILAMGSAYRE-LGLP--REK--
KINRD04462       --DVKEV---------VT-GG-G---E----TYRAHAVILALGSAYRQ-LGLP--DEK--
NOCDD04789       --PVKEV---------VA-G--G---E----TFLAHTVILATGSGYRE-LGVP--GEK--
```

```
NOCAA01941      --PVKEV---------TA-G--D---E----TFLAHTVILATGSGYRE-LGVP--GEK--
STRRD08913      --TPKVV---------RT-A--T---D----TYHAKAVILAMGSGYRE-LGVP--NEK--
THECD04863      --HPKVV---------KT-G--D---T----EYRAKAVIIATGSGYRE-LGLE--NEK--
THEBD03528      --DPKVV---------KT-Y--T---A----AYHAKAVILAMGSQYRE-LGLE--SEK--
CATAD08894      --AIKKV---------TD-SA-G---T----VHEAHTVIVSTGSAYKE-LGLP--DEK--
KRIFD06917      --DVKVV---------TD-SA-G---A----QHRARTVILAMGSGYRK-LGLP--REE--
KITSK03730      --DIKTV---------TD-SE-G---T----VHRARAVIVATGSQHRK-LDLP--NED--
STRBB05366      --DIKTV---------TD-SA-G---T----VHRAKTVIVTTGSQHRK-LGLA--HED--
STRVP03660      --EIKTV---------TD-TA-G---T----VHRAKAVIVTTGSQHRK-LGLP--NED--
STRSW04381      --EIKTV---------TD-TG-G---T----VHRAKAVIVTTGSQHRK-LGLP--NED--
STRGG03562      --DIKTV---------TD-TA-G---T----VHRAKSVIVTTGSQHRK-LGLP--NED--
STRFA03098      --EIKTV---------TD-TA-G---T----VHRAKAVIVTTGSQHRK-LGLP--NED--
STRAW04303      --EIKTV---------TD-TA-G---T----VHQAKAVIVTTGSQHRK-LALP--NED--
STRCO02929      --EIKTV---------TD-TA-G---T----VHRAKAVIVTTGSQHRK-LGLP--NED--
STRHJ05105      --EIKTV---------TD-TA-G---T----VHRAKAVIVTTGSQHRK-LGLP--NED--
MONBE04991      --KPFVI---------KS-GD---------RTVTADTVIVATGAVARR-LHFPGAD----
CHLRE01313      --RPFTV---------RT-AD---------KEVTADSLIIATGAVARR-LEFPGSGEE-G
MEDTR25591      --RPFRV---------FT-DS---------RTVEADSVIVATGAVAKR-LPFTGSGDGPN
SOLLC13750      --SPFKV---------VS-DD---------RTVLADAVILATGAVAKR-LEFPGSGN--N
PRUPE10733      --TPFKV---------FT-DS---------KTVLADSVVVATGAVAKR-LVFPGSGEGEG
MANES18605      --NPFKV---------FT-DS---------KAVLADSVIVATGAVAKR-LNFPGS----D
THECC00884      --SPFRI---------IT-DA---------KTVLADSVIVTTGAVAKR-LNFQGSGDGPG
PHYPA31147      --RPFKL---------FT-DN---------KEIRAQSVIVATGAVAKR-LDFPGSGEE-S
AMBTC19471      --HPFRL---------YS-ES---------KVVEADSVIIATGAVAKR-LDFPGSGEN-G
MUSAC26038      --RPFRV---------IS-SS---------TSVEADAVVVATGAVARR-LHFTGAD----
MUSAM33177      --RPFRV---------IS-SS---------TSVEADAVVVATGAVARR-LHFTGAD----
SETIT03079      --RPFRV---------AS-DS---------TTVLADAVVVATGAVARR-LHFPGSD----
ORYBR12195      --RPFRV---------AS-ES---------TTVLADAVVVATGAVARR-LHFPGSD----
COCLU07729      --RPFKY---------WL-HPMGDETEIEEEEHTADALIIATGAKARR-LDLKGED----
PHANO13702      --RPFKY---------WL-HPMGDDEEIEEEEHTADSLIIATGAKARR-LDLPGEE----
PHAND10804      --RPFKY---------WL-HPMGDDEEIEEEEHTADSLIIATGAKARR-LDLPGEE----
AURPU02089      --RPFKF---------WL-HPLGDEENLEEETHTADSIIIATGAKARR-LDLEGEE----
ZYMTR07711      --RPFKF---------WL-NPMGDDDTLKEETHTADSIIIATGAKARR-LDLPGEE----
DICPU05926      --KPFKV---------FV-EDEED--K----PILTESIILATGATAKR-MGVPGEE----
ENTHI00522      --QPFKL---------FT-EEGK--------EVLTKSVIIATGATAKR-MHVPGED----
LEPBA02231      --RPFTI---------WS-DD---------EEIKADSIIIATGATAKR-MFVKGED----
LEPBP02301      --RPFTI---------WS-DD---------EEIKADSIIIATGATAKR-MFVKGED----
LEPBL01462      --RPFQL---------WS-DE---------ERIEAEAVIIATGATARR-MHVTGED----
LEPBJ01276      --RPFQL---------WS-DE---------ERIEAEAVIIATGATARR-MHVTGED----
LEPIN02475      --KPFKL---------WS-DD---------ELIEAQAVIIATGATAKR-MNVIGED----
LEPII01996      --KPFKL---------WS-DD---------ELIEAQAVIIATGATAKR-MNVIGED----
LEPIC01426      --RPFKL---------WS-DD---------ELIEAQAVIIATGATAKR-MNVIGED----
SPIAZ00697      --RPFTV---------TA-DSG---------EYLAEAVIVSTGATAKR-LQLPGED----
PENRW10140      --RPFKM---------WT-EWNDD--EGSEPVRTADAVIIATGANARR-LNLPGEE----
PENCH09104      --RPFKM---------WT-EWNDD--EGSEPVRTADAVIIATGANARR-LNLPGEE----
EURHE07269      --KPFKL---------WT-EYNDG--PDNEPAHTADAVIIATGANARR-LNLPGEE----
ASPAC07301      --RPFKL---------WT-EWNDG--PDNEPAHTADAVIIATGANARR-LDLPGEE----
EMENI10387      --RPFKL---------WT-EWNDG--PDNEPARTADAVIIATGANARR-LNLPGED----
EMEND02596      --RPFKL---------WT-EWNDG--PDNEPARTADAVIIATGANARR-LNLPGED----
ASPTN06742      --RPFKL---------WT-EWNDG--PDNEPARTADAVIIATGANARR-LNLPGEE----
ASPCL04014      --KPFKL---------WT-EWNDG--PEQGPACTADAVIIATGANARR-LNLPGEE----
ASPFU05647      --KPFKL---------WT-EWNDG--PDKEPACTADAVIIATGANARR-LNLPGEE----
NEOFI00452      --KPFKL---------WT-EWNDG--PDKEPACTADAVIIATGANARR-LNLPGEE----
CRYPA10563      --RPFKY---------AT-EWDSD------TKHTADTVIIATGATARR-LGLVGED----
BLUGR03498      --RPFRY---------WT-EFNEE-------VSTADALIIATGASARR-LGLSGEQ----
SCLS112814      --RPFKY---------WT-EWDDK------TEHTADSIIIATGASARR-LGLPGEE----
MAGGR04266      --RPFKY---------CT-EWSPE------VMHTADTLVIATGASARR-LGLPGED----
NEUCR01575      --RPFKY---------AT-EWSPE------EYHTADSIILATGASARR-LHLPGEE----
NEUT908941      --RPFKY---------AT-EWSPE------EYHTADSIILATGASARR-LHLPGEE----
VERDA02342      --RPFRY---------SL-EWSPD------TTHTADAIILATGASARR-LGIPGED----
COLSU12486      --RPFKY---------ST-EFSPD------ETHTADAIILATGASAKR-LNLPGEE----
HYPAI01684      --RPFSF---------ST-EFNPD------ETHTADSVIIATGASARR-LHLPGED----
HYPVG06080      --RPFKF---------ST-EFNPD------ETHTADSVIIATGASARR-LNLPGED----
HYPJE05895      --RPFKF---------ST-EFNPD------ETHTADAVIIATGASARR-LNLPGED----
NECHA05020      --RPFRY---------TT-EFNPE------ETHTADAVIIATGASARR-LHLPGED----
```

```
FUSO415847      --RPFKY---------ST-EFSPE------ETHTADSVIIATGASARR-LNLPGED----
GIBZA01026      --RPYKY---------TT-EFSPE------ETHTAETVILATGASARR-LNLPGED----
SCHPO04025      --RPFKY---------WL-EGAEE--E--E-PHTADSVILATGASARR-LHITGED----
YARLI03635      --RPFKY---------WT-EFNED--E--E-PHTADAIILATGASAKR-LSLPGED----
ASHGO00946      --RPFKL---------WT-EFNED--E--E-PTTTDAIILATGASAKR-LGLPGEE----
KLULA02190      --RPFKF---------WT-EFNED--Q--E-PETTDAIILSTGASAKR-LHLPGEE----
ZYGRO00676      --KPFKF---------WT-EFNED--G--P-ANTTDAMILATGASARR-VNLPGEE----
DEKBR01813      --KPFKL---------WT-EFNED--A--E-PITSDSVIISTGASAKR-LHLPGED----
PICPG04776      --RPFKL---------WT-EFNED--E--A-PITTDAVVIATGASAKR-LHLPGEE----
CANTE00916      --RPFKL---------WT-EFYED--D--E-PITTDAVIIATGASAKR-MHLPGED----
LODEL03891      --RPFKL---------WT-EFNED--E--E-PITTDAIVIATGASAKR-MHLPGED----
DEBHA05546      --RPFKL---------WT-EWNED--S--E-PITTDAVVIATGASAKR-MHLPGED----
SPAPN03477      --KPFKL---------WT-EWNED--A--E-PITTDAVIIATGASAKR-MHLPGEE----
CANAW04800      --RPFKL---------WT-EWNED--A--E-PITTDAVIIATGASAKR-MHLPGED----
PICST04701      --RPFKL---------WT-EWNED--S--E-PVTTDAVIIATGASAKR-MHIPGEE----
PUCGT10887      --RPFKY---------WR-EGHES--D--E-SETADSIIIATGASARR-LNLPGED----
PUCGR11813      --RPFKY---------WR-EGHES--D--E-SETADSIIIATGASARR-LNLPGED----
PHYBL11006      --RPFKL---------WR-EGSES--EKEP-TDTADAVIIATGASAKR-MNLPGED----
USTMA03757      --RPFKF---------WR-EWAED--K--P-PELTETLIIATGASAKR-MHLTGED----
USTHO04132      --RPFKF---------WR-EFNED--Q--P-PELTETLIIATGASAKR-MHLPGEE----
WALSE04527      --RPFKY---------WR-EGSEE--DDNA-YELADSIILATGASAKR-LFLPGEE----
TREME07701      --RPFKY---------WT-EGEEE--DD-E-FMTADTIIIATGASAKR-LNLPGEE----
AURST04751      --RPFKY---------WR-EYSEG--DA-D-FESADTIIIATGASAKR-LHLPGEE----
FOMME10177      --RPFRY---------WR-EGAEN--D--E-PETADTVIIATGASAKR-LHLAGED----
CONPW06392      --RPFRY---------WR-EGQES--D--E-PETADTVVIATGASAKR-LSLKGED----
STEHR07076      --RPFKY---------WR-EYNEG--D--E-PETADTIIIATGASAKR-LGLRGED----
HETAN06295      --RPFRY---------WR-EYQEN--E--E-PETADTIIIATGASAKR-LGLKGEE----
GLOTR06982      --RPFKY---------WR-EGQED--E--E-AETADTVIIATGASAKR-LGLKGED----
PUNST01981      --RPFRY---------WR-EMQEG--G--E-PETADTIIIATGASAKR-LGLKGED----
LACBI02877      --RPFRY---------WR-EMQED--G--E-PETADAVIIATGASAKR-MGLKGEQ----
COPCI16429      --RPFRY---------WR-EGQED--Q--E-PETADTVIIATGASAKR-LGLKGEE----
DICSQ11618      --RPFRY---------WR-EFQED--Q--E-PETADTLILATGASAKR-LHLKGED----
TRAVS13180      --RPFRY---------WR-EYQED--S--E-PETADTLIIATGASAKR-LHLKGED----
WOLCO03584      --RPFRY---------WR-EFQED--Q--E-PETADTLIIATGASAKR-LGLKGEE----
FOMPI05979      --RPFRY---------WR-EFQED--Q--Q-PETADTLIIATGASAKR-LGLKGEE----
PHLGI10219      --RPFRY---------WR-EFQED--Q--E-PETADTLIIATGASAKR-MGLKGEE----
PHACH05757      --RPFRY---------WR-EFQED--Q--E-PETADTLIIATGASAKR-LGLKGEE----
RICTY00419      --RPFKI---------FT-GT--G--N----KYEADSIIICTGAESKW-LGIT--SEQ--
RICPR00429      --RPFKI---------FT-GT--G--N----EYEADSIIICTGAESKW-LGIA--SEQ--
RICPP00461      --RPFEI---------FT-GT--G--N----EYEADSIIICTGAESKW-LGIA--SEQ--
RICBR00434      --RPFKV---------ST-GS--R--T----EYEAESIIICTGAEARW-LGIP--TEQ--
RICB800988      --RPFKV---------ST-GS--R--T----EYEAESIIICTGAEARW-LGIP--TEQ--
RICCK00600      --RPFKV---------LT-GA--G--H----EYKAESIIICTGAEAKW-LGIA--SEQ--
RICAH00614      --RPFKV---------FT-GS--G--N----EYEAESIIICTGAEAKW-LGIA--SEQ--
RICAC00782      --RPFKV---------FT-GS--G--N----EYAAESIIICTGAEAKW-LGIA--SEQ--
RICFE00656      --RPFKV---------FT-GA--G--N----EYEAESIIICTGAEAKW-LSIA--SEQ--
RICMS00042      --RPFKV---------FT-GA--G--N----EYDAESMIICTGAEAKW-LGIA--SEQ--
RICM500453      --RPFKV---------FT-GA--G--N----EYDAESIIICTGAEAKW-LGIA--SEQ--
RICR300665      --RPFKV---------FT-GA--G--N----EYEAESIIICTGAEAKW-LGIA--SEQ--
RICAG00651      --RPFKV---------FT-GA--G--N----EYDAESIIICTGAEAKW-LGIA--SEQ--
RICP300630      --RPFKV---------FT-GA--G--N----EYDAESIIICTGAEAKW-LGIA--SEQ--
RICRS00632      --RPFKV---------FT-GA--G--N----EYDAESIIICTGAEAKW-LGIA--SEQ--
RICRO00663      --RPFKV---------FT-GA--G--N----EYDAESIIICTGAEAKW-LGIA--SEQ--
RICCN00618      --RPFKV---------FT-GA--G--N----EYDAESIIICTGAEAKW-LGIA--SEQ--
RICPT00620      --RPFKV---------FT-GA--G--N----EYDAESIIICTGAEAKW-LGIA--SEQ--
RICAE00497      --RPFKV---------FT-GA--G--N----EYDAESIIICTGAEAKW-LGIA--SEQ--
RICJY00467      --RPFKV---------FT-GA--G--N----EYDAESIIICTGAEAKW-LGIA--SEQ--
RICPU00072      --RPFKV---------FT-GA--G--N----EYDAESIIICTGAEAKW-LGIA--SEQ--
RICS100537      --RPFKV---------FT-GA--G--N----EYDAESIIICTGAEAKW-LGIA--SEQ--
BARBK00891      --RPFTL---------YG-DS--G--T----QYCCDALVIATGAQARW-LGLE--SEQ--
BARVW00932      --YPFIL---------YG-DS--G--T----QYCCDALIIATGAKARW-LGLE--SEQ--
BART100617      --YPFIL---------YG-DL--G--T----QYSCDALIIATGAKARW-LGLE--SEQ--
BARGA00466      --YPFVL---------YG-DS--G--T----QYSCDALIIATGAQARW-LGLE--SEQ--
BARHE01107      --RPFIL---------YG-DS--G--A----QYCCDALIIATGAQARW-LGLE--SEQ--
BARQU00895      --RPFIL---------YG-DS--G--T----QYCCDALIIATGAQVRW-LGLE--SEQ--
```

```
OCHA401642      --RPFRL---------KG-DS--G--T----IYTCDALIIATGAQAKW-LGLD--SEQ--
BRUAB01390      --RPFRL---------KG-DS--G--T----IYTCDALIIATGAQAKW-LGLE--SEQ--
BRUA201507      --RPFRL---------KG-DS--G--T----IYTCDALIIATGAQAKW-LGLE--SEQ--
BRUA101339      --RPFRL---------KG-DS--G--T----IYTCDALIIATGAQAKW-LGLE--SEQ--
BRUSU01458      --RPFRL---------KG-DS--G--T----IYTCDALIIATGAQAKW-LGLE--SEQ--
BRUME00510      --RPFRL---------KG-DS--G--T----IYTCDALIIATGAQAKW-LGLE--SEQ--
BRUSI01461      --RPFRL---------KG-DS--G--T----IYTCDALIIATGAQAKW-LGLE--SEQ--
BRUC201446      --RPFRL---------KG-DS--G--T----IYTCDALIIATGAQAKW-LGLE--SEQ--
BRUMC01440      --RPFRL---------KG-DS--G--T----IYTCDALIIATGAQAKW-LGLE--SEQ--
BRUMB01421      --RPFRL---------KG-DS--G--T----IYTCDALIIATGAQAKW-LGLE--SEQ--
BRUM501487      --RPFRL---------KG-DS--G--T----IYTCDALIIATGAQAKW-LGLE--SEQ--
BRUO201285      --RPFRL---------KG-DS--G--T----IYTCDALIIATGAQAKW-LGLE--SEQ--
RHILO01973      --RPFRA---------KG-DS--G--T----TYTADALIIATGAQAKW-LGIP--TEQ--
CHESB02097      --RPFRL---------KG-DS--G--T----VYTCDALIIATGAKARW-LGLP--SEN--
METPB00996      --RPFRL---------EA-DS--G--E----TYTCDSLIIATGAQAKW-LGLP--SEA--
METEP01063      --RPFRL---------EA-DS--G--E----TYTCDSLIIATGAQAKW-LGLP--SEA--
METEA00810      --RPFRL---------EA-DS--G--E----TYTCDSLIIATGAQAKW-LGLP--SEA--
METED01453      --RPFRL---------EA-DS--G--E----TYTCDSLIIATGAQAKW-LGLP--SEA--
METS403554      --RPFRL---------EA-DS--G--A----VYTCDALIVATGAQAKW-LGLP--SEA--
METNO05482      --RPFRL---------EA-DS--G--A----VYTCDALIIATGAQAKW-LGLP--SEA--
METSZ03234      --RPFRL---------TG-DS--G--K----SYTCDSLIVATGAKAKW-LGLP--SEE--
BEII900056      --RPFRL---------IG-DS--G--Q----IYTADSLVIATGAKAKW-LGLP--SEN--
METSB02743      --RPFRL---------FG-DS--G--E----AYVCDSLIIATGAKAKW-LGLP--SEQ--
MAGMM00401      --RPFTL---------NC-DS--G--D----VYTCDALIISTGASARW-LGME--SEK--
HYPNA00542      --RPFKA---------VG-ES--G--T----VYTADSVIISTGAQAKW-LDLP--SEE--
KETVY00902      --RPFTA---------VG-DS--G--T----VYTADAIILATGARAKW-LGLP--SEE--
KETVW00472      --RPFTA---------VG-DS--G--T----VYTADAIILATGARAKW-LGLP--SEE--
ROSDO03242      --RPFVA---------KG-DS--G--T----TYIADAVILATGARAKW-LGLP--SEE--
ROSLO02615      --RPFVA---------KG-DS--G--T----TYIADAVILATGARAKW-LGLP--SEE--
RUEPO00888      --RPFVA---------KG-DS--G--T----TYTADAVILATGARAKW-LGME--SEE--
RUEST00613      --RPFVA---------KG-DS--G--T----TYTADAVILATGARAKW-LGLP--SEE--
PHAIB02390      --RPFVA---------KG-DS--G--T----IYTADAVILATGARAKW-LGLP--SEE--
PARDP02130      --RPFVA---------EL-DS--G--A----RITADAVILATGAQARW-LGLS--SEE--
DINSH02620      --RPFTA---------YG-DS--G--T----IYKAEALILATGAQAKW-LGLP--SEE--
RHOCB02768      --RPFVA---------TG-DS--G--T----VYTADTVILATGAQARW-LGLP--SEQ--
RHOS500248      --RPFTA---------HA-DS--G--T----TYTADAVILATGAQARW-LGLP--SEE--
RHOS400150      --RPFTA---------QA-DS--G--M----TYTADAVILATGAQARW-LGLP--SEE--
RHOS100227      --RPFTA---------QA-DS--G--M----TYTADAVILATGAQARW-LGLP--SEE--
RHOSK02952      --RPFTA---------QA-DS--G--M----TYTADAVILATGAQARW-LGLP--SEE--
MIDMI00790      --RPFSA---------VG-AG--G--T----IYTAESVIISTGAQAKW-LGLP--SEE--
ACEP301595      --SPFLL---------TG-DS--G--T----IYKARAVVVATGAQARW-LGLP--SEK--
MICAA01566      --RPYRA---------VA-DS--G--T----VYTADTVIICTGAKARW-LGLE--GEQ--
TISMK03676      --RPFVA---------TG-DS--G--D----RYVADAVIISTGAQAKW-LGLP--DEE--
AZOL402409      --RPFVC---------KG-DS--G--D----TYTADSVIIATGAQARW-LGIS--TEE--
PSEUV04348      --RPFEL---------KG-DS--G--T----IYTADALVIATGAQAKW-LGLP--SEE--
HIRBI01095      --RPFTL---------IG-ES--G--T----TYTCDALIISTGAQAKW-LGLD--SEK--
PARL102241      --HPFEL---------KG-DS--G--T----LYTADALIIATGAQAKW-LGIP--SEE--
MARMM02139      --RPFVL---------KG-DS--G--T----VYTADSVIIATGAQAKW-LGLE--SEQ--
PHEZH02588      --RPFRL---------ET-DS--G--Q----TWLAETLIIATGAQAKW-LGLE--SEQ--
CAUCR02826      --RPFTV---------KT-DS--G--Q----DWIAETIIIATGAQAKW-LGLE--SEA--
CAUCN02939      --RPFTV---------KT-DS--G--Q----DWIAETIIIATGAQAKW-LGLE--SEA--
CAUST00891      --RPFTV---------KT-DS--G--Q----DWIAETIIIATGAQAKW-LGLE--SES--
PARBH01522      --RPFSF---------KG-DS--G--T----IYTADAVIVATGAQAKW-LGLP--SEK--
PELHB02084      --RPFRL---------TG-DS--G--K----TYTADSVIIATGAQAKW-LGLP--TEE--
HYPDA03273      --RPIRL---------EG-DS--G--D----IYTCDALIICTGAQARW-LGLP--SEA--
HYPSM04642      --RPFRL---------EG-DS--G--D----TYTCDALIIATGAQARW-LGLP--SEE--
OLICO01107      --SPFRI---------TC-DS--G--D----VYLADAVIIATGAQARW-LGLP--SED--
OLICM02790      --SPFRI---------TC-DS--G--D----VYLADAVIIATGAQARW-LGLP--SED--
RHOPS01446      --RPFRL---------SC-DS--G--D----VYIADTVILATGAQARW-LGIP--SEQ--
RHOPA04062      --QPFRL---------TC-DS--G--D----VYLADTLILATGAQARW-LGIP--SEQ--
RHOPT04533      --QPFRL---------TC-DS--G--D----VYLADTLILATGAQARW-LGIP--SEQ--
RHOPX04235      --RPFRL---------TC-DS--G--D----VYLADTLILATGAQARW-LGIP--SEQ--
BRADU07321      --RPFRL---------TC-DS--G--D----VYLAETVILATGAQARW-LGLP--SEG--
BRASO01239      --RPFRL---------TC-DS--G--D----VYLAETVILATGAQARW-LGLP--SEE--
BRASB06352      --RPFRL---------TC-DS--G--D----VYLAETVILATGAQARW-LGLP--SEE--
```

```
RHOPB03773      --RPFRL---------SC-DS--G--D----VYLAETVILATGAQARW-LGIP--SEE--
NITWN02324      --RPFRL---------TC-DS--G--E----VYLAETVILATGAQARW-LGLP--SED--
NITHX02707      --RPFRL---------TC-DS--G--D----VYLAETVILATGAQARW-LGLP--SED--
AZOC501143      --RPFKM---------VT-EG--G--E----TYVADVVILATGAQARW-LGLG--SEE--
XANP202679      --RPFRM---------VT-ES--G--D----TYVADVVILATGAQARW-LGLE--SEA--
CHLTF01983      --RPFRV---------WS-RD-----D----EYTADAVIVSSGASAKW-LGLP--SEE--
IGNAJ02236      --RPFKL---------KS-YD-----E----EYYADAVIISTGASARL-LGLE--SEA--
MELRP00403      --RPFVI---------KS-EN-----E----SHTADSVIIATGASAKL-LGIE--SEK--
ANADF00468      --RPFQL---------WQ-ED-----R----LFTADAVVVATGASAKW-LAIA--SEK--
CHLCH01125      --RPFSV---------TL-ED--G--Q----EFLTHALIVATGANARW-LGIE--SED--
PELPB01419      --SPFCL---------TL-ED--G--Q----EFLTHALIVATGANAKW-LGID--SES--
CHLL200781      --SPFSL---------TL-DD--G--R----EIVARSLIIATGANAKW-LGIA--SED--
CHLTE00830      --SPFSL---------ML-DN--G--Q----EILARTLIIATGANAKW-LGIE--SEE--
CHLP800854      --SPFSL---------TL-DD--G--S----EILARTIIIATGANAKW-LGIE--SEE--
CHLL701225      --RPFSL---------TL-ED--G--S----EILTRALIIATGANAKW-LGIE--SED--
CHLPM00993      --RPFCL---------TL-ED--G--S----EVLARSVIIATGANAKW-LGIE--SED--
WOLTR00562      NEYKFRS---------SG-N---N--D----DYYSNAVIIAAGSQAKW-LGLE--SER--
WOLPP00135      NEYRFRS---------CG-N---A--S----DYYSNAIIIAAGAQAKW-LGLE--SEK--
WOLPM00650      NEYRFRS---------SG-N---I--N----DYYSDAIIIASGAQAKW-LGLE--SEK--
WOLWR00416      NEYRFRS---------SG-N---T--N----DYYSDAIIIASGAQAKW-LGLK--SEK--
EHRCR00696      DVYPFKC---------IG-IF--G--D----QYIADSIIIATGAQAKW-LNIK--SEE--
ANAMM00345      NVYPFKC---------TS-MF--G--E----EYYAYSIIVATGAQAKW-LGMD--SEQ--
ANAMF00341      NVYPFKC---------TS-MF--G--E----EYYAYSIIVATGAQAKW-LGMD--SEQ--
ANAPZ00610      SVYPFKC---------TS-MF--G--E----TLQAYSIIVATGAQAKW-LGLE--SEQ--
NEOSM00537      -SSPFVL---------RG-EG--G--N----KYRARSIVVATGAQAKW-LGLE--SEK--
NEORI00513      -SSPFIL---------RG-EG--G--N----KYRARSIVVATGAQAKW-LGLE--SEK--
PELUB00076      --TPFEA---------IG-DS--G--Q----KYTADSIIISTGAQARW-LNLE--SEQ--
PELSM00736      --NPFIL---------IG-ES--G--T----TYTADSVIISTGAQARW-LNLE--SET--
PUNMI01409      --RPFRI---------SL-DS--G--A----SMTADVVILATGAQARW-LGID--SEQ--
ASTEC00651      --RPFRL---------ET-DA--G--N----VYLAETVIISTGAQAKW-LGLE--SEK--
ZYMMT00154      -KRPFRL---------TG-DG--G--Q----IYLADALVISTGAQARW-LGLQ--SET--
ZYMMO00984      -QRPYRL---------MG-DG--G--Q----VYLADSLIISTGAQARW-LGLE--SET--
ZYMMA00176      -QRPYRL---------TG-DG--G--Q----VYLADSLIISTGAQARW-LGLE--SET--
ZYMMN00183      -QRPYRL---------TG-DG--G--Q----VYLADSLIISTGAQARW-LGLE--SET--
SPHAL00143      -QRPFKL---------TG-DG--G--D----VYLAETLVIATGAQAKW-LGVP--GEQ--
SPHWW03978      -ERPFRL---------RG-DG--G--T----LYVCDTLVIATGAQAKW-LGVE--GEA--
SPHJU02313      -ERPFRL---------KG-DS--G--T----LYYADSLVIATGAQAKW-LGAE--GEQ--
NOVAD02319      -GSPFRA---------VG-DS--G--D----IYEGDVLVIATGAQAKW-LGVP--GEQ--
ERYLH00934      -GSPFRA---------VG-DS--G--D----EYIGDVLIIATGAQAKW-LGVP--GEQ--
GRABC00720      --TPFRC---------TA-DS--G--D----VFEADSVIIATGAQARW-LGLP--NEK--
GLUDA03075      AGEPFRL---------VG-DS--G--D----VYLARSVVIATGAQAKW-LGIP--GEA--
KOMMN00774      AGR-FYA---------TG-DS--G--T----LYEARTVIIATGAQAKW-LGVP--GEK--
HALVD01097      --RPFTV---------TL-KN--G--D----VYTADALIAASGASART-LGIP--GEE--
HALHT01699      --RPFRV---------EL-SN--G--D----VYTCDAFIAASGASART-LGVP--GED--
METI401038      --KIKRV---------KI-DE-----E----WIETKSVVIAVGARPRK-LSVP--GEA--
PLAL201612      --RPFVL---------KT-SD--D--T----VVEAHSLIIATGARANY-LGLP--SED--
GEMAT01983      --RPFRI---------TP-NY--A--E----PLTAHTVIVATGAAAKW-IGLESEMRL--
CYAAP02456      --RPFII---------TA-DE-----T----EVQALAVIICTGATAKR-LHIL--GEE--
CHLPN00303      --RPFIL---------KS-KE-----E----TYSCDACIIATGASAKR-LEIPGAGND--
CHLPP00439      --RPFIL---------KS-KE-----E----TYSCDACIIATGASAKR-LEIPGAGND--
CHLPE00727      --RPFVL---------YS-NE-----E----IYTCDACIIATGASAKR-LDIPGAGDD--
CHLTR00101      --RPFVL---------KS-GK-----E----TFTCDACIIATGASAKR-LSIPGAGDN--
CHLTA00102      --RPFVL---------KS-GK-----E----TFTCDACIIATGASAKR-LSIPGAGDN--
CHLTJ00101      --RPFVL---------KS-GK-----E----TFTCDACIIATGASAKR-LSIPGAGDN--
CHLTD00098      --RPFVL---------KS-GK-----E----TFTCDACIIATGASAKR-LSIPGAGDN--
CHLT700102      --RPFVL---------KS-GK-----E----TFTCDACIIATGASAKR-LSIPGAGDN--
CHLT000101      --RPFVL---------KS-GK-----E----TFTCDACIIATGASAKR-LSIPGAGDN--
CHLT500104      --RPFVL---------KS-GK-----E----TFTCDACIIATGASAKR-LSIPGAGDN--
CHLTL00098      --RPFVL---------KS-GK-----E----TFTCDACIIATGASAKR-LSIPGAGDN--
CHLTG00102      --RPFVL---------KS-GK-----E----TFTCDACIIATGASAKR-LSIPGAGDN--
CHLTS00101      --RPFVL---------KS-GK-----E----TFTCDACIIATGASAKR-LSIPGAGDN--
CHLT900102      --RPFVL---------KS-GK-----E----TFTCDACIIATGASAKR-LSIPGAGDN--
CHLTZ00101      --RPFVL---------KS-GK-----E----TFTCDACIIATGASAKR-LSIPGAGDN--
CHLT400432      --RPFVL---------KS-GK-----E----TFTCDACIIATGASAKR-LSIPGAGDN--
CHLT100103      --RPFVL---------KS-GE-----E----TFTCDACIIATGASAKR-LSIPGAGDN--
```

```
CHLT200350      --RPFVL---------KS-GE-----E----TFTCDACIIATGASAKR-LSIPGAGDN--
CHLTB00350      --RPFVL---------KS-GE-----E----TFTCDACIIATGASAKR-LSIPGAGDN--
CHLTC00378      --RPFVL---------KS-GE-----E----TFTCDACIIATGASAKR-LSIPGAGDN--
SIMNZ00831      --RPFLV---------KG-SK-----T----VVQAEALIIATGATAKR-LDIPGTGDH--
PARUW01706      --YPFII---------KG-KK-----S----HYQASSVIVSTGATAKR-LPIPGAGDD--
WADCW00943      --YPYKI---------KG-NK-----H----EHEAFAVIIATGASAKR-LNIPGTREG--
SINAD04980      --RPFRVKTSTDPK-DPE-AA--T--A----EYTADAVIVATGASART-LGLD--KEM--
BIFLB01558      ---------QGELKSVHL-SG--G--Q----DLQTRTVIVATGSNYRH-LNVP--GEL--
BIFAB00498      ---------QGELKSVHL-SG--G--Q----DLQTRTVIVATGSNYRH-LNVP--GEL--
BIFAV01561      ---------QGELKSVHL-SG--G--Q----DLQTRTVIVATGSNYRH-LNVP--GEL--
BIFAS01557      ---------QGELKSVHL-SG--G--Q----DLQTRTVIVATGSNYRH-LNVP--GEL--
BIFA001518      ---------QGELKSVHL-SG--G--Q----DLQTRTVIVATGSNYRH-LNVP--GEL--
BIFBA01814      --AADAADAPQPRYRLSL-SD--G--S----VLESRAIIVATGSNFRK-LGVP--GER--
BIFAA01619      ---------DSGIKTVTL-DD--G--D----VYDARALVIATGSNYRK-LGVP--GET--
BIFDB02112      ---------ESDTKTVTL-DQ--G--D----IYQTRAVVVATGSQYRK-LGVP--GES--
TERSS03091      -----------RPI-ELN-LG--N--K----TIKTRTLIVASGASARW-LGLP--SEQ--
GRATM03160      --G--------GPI-KLN-IG--G--E----IVHTRTLIIASGASARW-LNLP--SEQ--
GRAMM03836      -----------SPF-ELT-VG--K--D----IIKTRSLIIASGASARW-LGLP--SEQ--
AKKM801247      --AT-------GLF-TVK-TS--G--Q----NYETRSIIVATGASARY-LGIP--GEE--
OPITP04130      --Q---------PR-KLI-CG--D--R----TVLAKTVIIATGASPRM-TGVP--GEK--
CORAD01794      --D---------VK-KLI-AG--D--K----VYEAKTVIIGTGARPRL-LGIP--GEL--
BUCCC00180      --SFFEI---------TG--K--I--N----QYFSQSIIIATGSYTKF-LNLP--KEN--
CENSY00347      --RPFKV---------LT--S--E--E----EYEAKAVIVGTGATPRK-LGVE--GEK--
NITMS00668      --KPLKV---------LT--A--S--E----EYEGRAIIIATGANPRK-LGLE--GEE--
MEIRD02823      --EGFVV---------RG--Y--E--Q----DYRARSVILATGANPKK-LGVP--GEE--
HERA203780      --RPFKL---------HI-DN--G--T----IVETETLIIATGASPRK-LGVP--GEL--
CHLAA02018      --RPFTI---------HT-DS--G--Q----TVTADAIIVSTGASPRK-LGVP--GEE--
CHLSY02175      --RPFTI---------HT-DS--G--Q----TVTADAIIVSTGASPRK-LGVP--GEE--
CHLAD02599      --RPFVI---------HT-DG--G--Q----TVTADAIIVSTGASPRK-LGVP--GEA--
CALAS01745      --HPFLV---------RT-ES--G--A----EYQTRALILCTGARPRY-LEVP--GEK--
ANATU01696      --RPFVI---------ET-ES--G--Q----EIKAQTLIIATGASPNH-LNVP--GEK--
SULMS00235      --NIHEI---------LL-DN--N--K----KIQTKTIIIATGAYPKY-LNID--NEK--
BLASB00041      --GIHNI---------FF-E---K--E----CIESRGLIIATGSRPKF-LGIE--KEK--
BLASP00551      --GIHRV---------YM-VENEE--N----IIESKGIIIATGSSPKF-LGMD--KEK--
AZOPC00437      --QPYKV---------VI-DN--K--Q----TIETDTLVICTGATAKY-LGLS--DEQ--
LEPBD01878      --KIFKL---------HL-DN--G--N----IIEAKTIILSTGASAKY-LGIE--NEK--
SALRD02577      --RPFRL---------LI-DG--E--T----PVYAQTAIISTGASARY-LGLE--NEQ--
SALRM02866      --RPFRL---------LI-GG--E--T----PVYAQTAIISTGASARY-LGLE--NEQ--
RIEPU00166      --RPFLL---------FG-SL--K--R-----YSCDSLIIATGSSFRN-LGLS--FEK--
ORITB00015      --YPFEA---------TG-NK--A--L-----YYANSVVICTGAQAKW-LNIP--SET--
ORITI01234      --YPFEA---------TG-NK--A--L-----YYANSVVICTGAQAKW-LNIP--SET--
PREMB01541      --RPFHL---------ED-ER--G--N----QIEAETVIIATGASAKY-LGLP--DEE--
PREDF01155      --RPFHL---------ED-ER--G--N----EIEAETVIIATGASAKY-LGLA--DEE--
PREI702011      --RPFIV---------ED-ER--G--N----VIEANTLIIATGASAKY-LGLS--DEE--
ALIFI01035      --RPFHV---------VI-DG--T--T----QIKAETLIIATGASAKY-LGLP--SET--
ODOSD00362      --HPHKL---------TI-DD--C--K----QIEADAVIIATGASAKY-LGLP--TEE--
PRER201250      --APYVC---------TA-DD--G--T----EIEADTVIIATGASAKY-LGLD--DER--
PALPW00206      --APYKV---------TI-DG--E--K----VIEAETIIISTGATAKY-LGLP--DEQ--
PORGI00953      --APYRI---------TI-DG--E--K----EITADTLIISTGATAKY-LGLA--DEA--
PORG301168      --APYRI---------TI-DG--E--K----EITADTLIISTGATAKY-LGLA--DEA--
BACV803426      --RPYRI---------TI-DD--E--K----VIEAETVIISTGATAKY-LGLE--DEQ--
BACT601486      --MPYKI---------TI-DD--E--K----VIETETLIIATGAAAKY-LGLD--DEK--
BACTN04290      --APYQI---------TI-DG--D--K----VIETEALIISTGATAKY-LGLE--DEK--
BACFR01032      --APYKI---------TI-DG--E--K----VIEADSLIIATGATAKY-LGLD--DEK--
BACFN00913      --APYKI---------TI-DG--E--K----VIEADSLIIATGATAKY-LGLD--DEK--
BACF600963      --APYKI---------TI-DG--E--K----VIEADSLIIATGATAKY-LGLD--DEK--
OWEHD03413      PGEYHKL---------TI-DE--T--T----TILARTVIIATGASAQY-LGLE--SEK--
PSYTT00390      VGGIHKA---------QI-DN--K--T----WVEAETVLISTGASAKY-LGIP--SEQ--
NONDD00011      KGGIHTA---------VV-DG--K--T----KIEANTVIISTGASAKY-LNIP--SEQ--
ROBBH02711      PGGIHRV---------TI-DD--D--K----VVEAETVIISTGASAKY-LNIP--SEQ--
CELAD02592      IGGIHKA---------TV-DD--S--I----QIEAETVIISTGATAKY-LNIP--SEQ--
CELLC02474      VGGIHKV---------TV-DN--S--K----TIEAETVIISTGATAKY-LGLE--SEQ--
MARSH03072      VGGIHKA---------IV-DN--D--K----TIEAETIIISTGATAKY-LNIP--SEQ--
MURRD00159      VGGIHKV---------TV-DD--S--T----QLEAETVIISTGASAKY-LNIP--SEQ--
ZOBGA00152      VGGIHKV---------TI-DD--S--K----TIEAETIIISTGASAKY-LNIP--SEQ--
```

```
GRAFK00949      VGGIHKA---------CV-DN--N--K----WIEAESVIISTGATAKY-LGLP--SEQ--
ZUNPS02344      VGGIHKA---------CI-DN--S--K----WVEAETVIISTGATAKY-LGLP--SEQ--
AEQSU02079      VGGIHKI---------TV-DN--G--T----QLEAETVIISTGATAKY-LGLP--SEQ--
HALH105745      --PVHKL---------WT-ES--D--Q----EIHADAVIISTGATAKW-LGLD--SEK--
SAPGL02893      --PVHKA---------WT-EG--G--E----EIHAHSVVIATGASAKW-LGLD--SEK--
AMOA500014      --YPFQL---------TI-DT--T--T----TVEAKTVIIATGASAKW-LGLE--SEK--
FLAIG01802      --PIHKV---------WI-NG--E--K----EIHADTVIISTGASAKY-LGLE--SEQ--
FLACA02326      --DLHKV---------WI-HD--G--K----ELLCDTVIISTGASAKY-LGLP--SEQ--
FLAJ100198      --GIHKV---------WI-ND--S--I----ELHCETVIISTGASAKY-LGLP--SEQ--
FLABF01528      --GLHKV---------WI-ND--T--V----ALQCETVIISTGASAKY-LGLP--SEQ--
CAPOD01333      --HPHKV---------WV-DD-----Q----ELHADAVIIATGASAKY-LGLP--SEQ--
CAPCC00414      --TPHKV---------WI-NE--T--Q----EIHADAVIIATGASAKY-LGLD--SEQ--
FLELS01898      --GTHIA---------YD-DS--G--I----KIEAQTVVISTGAAAKW-LGLP--SEA--
SOLCM01166      --LPHKV---------TL-DD--G--K----TVTADAVIISTGASAKW-LGLP--SEQ--
PEDHD01598      --LPHKV---------IV-DD--I--K----TVTADTVIISTGATAKW-LGLP--SEQ--
SPHS203207      --PVHKV---------VI-DE--I--K----TITADTVIIATGATAKW-LGLD--SEQ--
LEAB401188      --LAHKV---------II-DE--T--K----EIEAKTVIIATGASAKW-LGLE--SES--
EMTOG00092      --GAHKV---------II-DD--A--Q----EIVAKTVIIATGASAKW-LGLE--SEQ--
SPILD01737      LDNPHRA---------IV-DD--K--H----EITADSIIISTGASAKW-LGLP--SEM--
DYAFD00084      --YPHKV---------II-DN--T--H----EITADAVIISTGASAKW-LGIE--GEE--
CYCMS03063      --RPFKV---------IV-DE--G--K----TLLAETVIISTGASAKW-LGIE--SET--
ECHVK03326      --GPHKV---------IV-DD--K--D----EILADTVIISTGASAKW-LGLE--SET--
BELBD02542      --KPHKV---------IV-DE--Q--V----EIHAETVLISTGASAKW-LGLE--SEE--
NITGG02112      --RPFTI---------TT-HS--E--T-----YTAEAVIVCTGASPRK-LGIP--AEQ--
THEM700111      --RPFKL---------KV-GS--K--E-----LEADAVIIATGASAKW-LGLP--GEK--
CREAS01350      --RPFKV---------WV-ED--E--L-----YEAWSIIIATGAKPRF-LGVK--GEQ--
TURPD02772      --APFRI---------TA-EG-----T----TYTSDTVIVATGATARF-LGIP--SEL--
SORC507603      --HPFKV---------WV-ED--T--L----YL-AKAVVIATGARANY-LGLD--SED--
BDEBA00337      --RPFKV---------WI-GD--K--L----HL-AKSIIISTGASAKY-LGLP--SEK--
STIAD02752      --RPFLI---------QG-ES-----A----SYRSEAIIIATGASAKW-LNVK--GED--
MYXXD01876      --RPFLI---------QG-ES-----V----SCRSETVIISTGATAKW-LGVK--GED--
MYXFH03454      --RPFLI---------QG-ES-----V----SYRSETVIISTGATAKW-LGVK--GED--
CORCM01960      --RPFLL---------ES-ES--G--I----QVRSETVIISTGATAKW-LNVK--GED--
MYXSD02209      --RPFLI---------ES-ES--G--L----KVRSETVIISTGATAKW-LGVK--GED--
LEPFC02126      --GGFI----------LS-GE--D--E----VLHTKTLIIASGASAKY-LGLP--SEK--
LEPFM02294      --DVIRV---------FC-DD--E--K----VLETKTLIVASGASAKY-LGLP--SEK--
SOLUE00582      --RPFR----------LN-IE--G--D----WMETRTVIIASGASARW-LGLE--SEH--
KORVE01333      --RPFE----------LH-MG--K--E----TILTKTLIIASGASARW-LNLK--SEQ--
ACIC502553      --RPFV----------LD-LG--R--E----VIHARTLIIASGASARW-LGLP--SEQ--
ACIFD00030      --RPFAV---------HT-ND--D--RSGEPAYLADAVIMATGARSLM-LGLE--REY--
HALMS01402      --RPFKL---------TC-EN--G--T----ELMAESLIISTGASAKY-LGLP--NEK--
CHLPD01133      --TPFSL---------SL-DD--G--T----EIVTRSLIVATGANAKW-LNID--SEK--
CHLPB01137      --TPFCV---------YL-ED--G--R----EILSRTLIVATGANAKW-LGLP--SEK--
PROA200897      --KPFCL---------YL-DD--E--R----EVLTRSLIIATGANARW-LGLP--SEE--
WIGBR00492      --KPFIC---------KG-E-K---------EYISDAIIIATGSSARY-LGLS--SEQ--
BUCA500289      --KPFFL---------IG-ENN---------KYTADSVIIATGANPRY-LGLQ--SES--
BUCAI00292      --NPFFL---------IG-ENN---------KYTADSVIIATGANPRY-LGLQ--SES--
BUCAF00306      --NPFFL---------IG-ENN---------KYTADSVIIATGANPRY-LGLQ--SES--
BUCAT00287      --KPFFL---------IG-ENN---------KYTADSVIIATGANPRY-LGLQ--SES--
BUCA000302      --KPFFL---------IG-ENN---------KYTADSVIIATGANPRY-LGLQ--SES--
BAUCH00290      --YPFSL---------KG-DHT----T----EYTCNVLIIATGASARY-LGLS--SEN--
BLOVB00369      --RPFQL---------YG--NT----N----QYTCDSLIISTGGSARS-LGIP--SEI--
BLOFL00367      --YPFHL---------YG-GNT----N----EYTCDSLIISTGGSARY-LGLP--SET--
BLOPB00375      --YPFCL---------YG--NT----H----EYTCHALIISTGGYARS-LGIP--SEE--
BUCAP00289      --KPFFL---------LG--EY----N----KYTCDAVIIATGANPRY-LGLS--SEN--
COXBU01001      --RPFLL---------KG--DN----A----TYSCDALIIATGASARY-LGLP--SEK--
COXBN01188      --RPFLL---------KG--DN----A----TYSCDALIIATGASARY-LGLP--SEK--
COXBR01117      --RPFLL---------KG--DN----A----TYSCDALIIATGASARY-LGLP--SEK--
COXB200717      --RPFLL---------KG--DN----A----TYSCDALIIATGASARY-LGLP--SEK--
COXB100914      --RPFLL---------KG--DN----A----TYSCDALIIATGASARY-LGLP--SEK--
FRAP200241      --RPFKL---------VG--EI----E----EYTCDALIISTGATAKY-LGLE--SEE--
FRANT01012      --RPFKL---------VG--EI----E----EYSCDALIIVTGATARY-LGLE--SEE--
FRACN00522      --KPFKL---------VG--EV----E----QYTCDALIIATGATAKY-LGLE--SEE--
FRATT00465      --KPFKL---------VG--EV----E----QYTCDTLIIATGATAKY-LGLE--SEE--
FRAT100465      --KPFKL---------VG--EV----E----QYTCDTLIIATGATAKY-LGLE--SEE--
```

```
FRATE00462      --KPFKL---------VG--EV----E----QYTCDTLIIATGATAKY-LGLE--SEE--
FRATW01216      --KPFKL---------VG--EV----E----QYTCDTLIIATGATAKY-LGLE--SEE--
FRATM01110      --KPFKL---------VG--EV----E----QYTCDTLIIATGATAKY-LGLE--SEE--
FRACF00566      --KPFKL---------VG--EV----E----QYTCDALIIATGATAKY-LGLE--SEE--
FRATO01212      --KPFKL---------VG--EV----E----QYTCDTLIIATGATAKY-LGLE--SEE--
FRATH01486      --KPFKL---------VG--EV----E----QYTCDTLIIATGATAKY-LGLE--SEE--
FRATF01216      --KPFKL---------VG--EV----E----QYTCDTLIIATGATAKY-LGLE--SEE--
FRATN00564      --KPFKL---------VG--EV----E----QYTCDALIIATGATAKY-LGLE--SEE--
ACIF500512      --RPFRL---------SG--DQ----H----CYTCDALILATGASAKY-LGLP--SED--
ACIF200362      --RPFRL---------SG--DQ----H----CYTCDALILATGASAKY-LGLP--SED--
DECAR01284      --KPFTL---------IG--DA----G----TYTCDALIIATGASAMY-LGLP--SEE--
NEIG100525      --RPFAL---------KG--DM----G----EYTCDALIVATGASAKY-LGLP--SEE--
NEIG201314      --RPFAL---------KG--DM----G----EYTCDALIVATGASAKY-LGLP--SEE--
NEIM800926      --RPFTL---------KG--DM----G----EYTCDALIVATGASAKY-LGLP--SEE--
NEIMP01199      --RPFTL---------KG--DM----G----EYTCDALIVATGASAKY-LGLP--SEE--
NEIMB01212      --RPFTL---------KG--DM----G----EYTCDALIVATGASAKY-LGLP--SEE--
NEIMF01143      --RPFTL---------KG--DM----G----EYTCDALIVATGASAKY-LGLP--SEE--
NEIML01119      --RPFTL---------KG--DM----G----EYTCDALIVATGASAKY-LGLP--SEE--
NEIMM00780      --RPFTL---------KG--DM----G----EYTCDALIVATGASAKY-LGLP--SEE--
NEIMH00827      --RPFTL---------KG--DM----G----EYTCDALIVATGASAKY-LGLP--SEE--
NEIMG01168      --RPFTL---------KG--DM----G----EYTCDALIVATGASAKY-LGLP--SEE--
NEIMN01254      --RPFTL---------KG--DM----G----EYTCDALIVATGASAKY-LGLP--SEE--
NEIMO00810      --RPFTL---------KG--DM----G----EYTCDALIVATGASAKY-LGLP--SEE--
NEIM701229      --RPFTL---------KG--DM----G----EYTCDALIVATGASAKY-LGLP--SEE--
NEIMA01334      --RPFTL---------KG--DM----G----EYTCDALIVATGASAKY-LGLP--SEE--
NEIMW01125      --RPFTL---------KG--DM----G----EYTCDALIVATGASAKY-LGLP--SEE--
DICNV01037      --RPFTL---------KT--EK----E----TYSCDALIIATGARAKY-LGLP--SET--
VESOH00047      --RPFVL---------KG--EI----I----TYTADSVIIATGASARY-LGLE--SEE--
RUTMC00043      --RPFVL---------KG--ET----T----TYTADAVIIATGSSAHY-LGLE--SEE--
HALHL02255      --QPITL---------EG--DQ----H----RYTCDALIIATGASAKY-LGLE--SEK--
PELPD03177      --RPFLL---------EG--DS----A----AYTCDALIIATGASARY-LGLP--SEQ--
GEOS804030      --PPFVL---------EG--DS----G----TYSCDALIIATGASAKY-LGLP--SEH--
GEOBB03636      --PPFVL---------EG--DN----G----SYSCDALIIATGASAKY-LGLP--SEH--
GEOSM03700      --PPFVL---------EG--DN----G----SYSCDALIIATGASAKY-LGLP--SEH--
HALNC00046      --RPFTL---------KG-DSG---------TYTCDALIVATGASAKY-LGLE--SES--
HAEPS00887      --RPFTL---------KG-DFN---------TFTCDALIIATGASAKY-LGLP--SEE--
GALAU02285      --RPFTL---------YG-DSQ---------TFTCDALIIATGASAKY-LGLP--SEE--
HISS201000      --RPFKL---------FG-DAG---------AYSCDALIIATGASARY-LGLP--SEE--
HAES101151      --RPFKL---------FG-DAG---------AYSCDALIIATGASARY-LGLP--SEE--
PASMU00573      --RPFKL---------YG-DVH---------TFSCDALIIATGASARY-LGLP--SEE--
PASMH00579      --RPFKL---------YG-DVH---------TFSCDALIIATGASARY-LGLP--SEE--
ACTSZ01560      --RPFKL---------YG-DEQ---------TFSCDALIIATGASARY-IGLE--SEE--
AGGAN00985      --RPFKL---------YG-DAQ---------TFSCDALIIATGASARY-LGLP--SEE--
NITHN03181      --LPFLL---------KG-DSG---------HYTADALIIATGASAKY-LGLP--SEE--
NITOC00311      --VPFLL---------KG-DTD---------HYTADALIIATGASAKY-LGLP--SEG--
NITWC02498      --LPFLL---------KG-DTD---------HYTADALIIATGASAQY-LGLP--SEG--
METNJ00648      --RPFKL---------TG-DAG---------EYTADALIIATGASAKY-LGMD--SEE--
METFJ01616      --RPFTL---------TG-DAG---------TYTADALIIATGASAKY-LGMP--SEE--
ALKEH00244      --KPFRL---------EG-DAG---------SYTCDALIIATGATAKY-LGLD--SEQ--
MARMS03166      --RPFRL---------EG-DSG---------VYTCDALIISTGASAQY-LGLE--SET--
MARM102688      --RPFRL---------EG-DSG---------VYTCDALIIATGASAQY-LGLE--SET--
THICR00763      --RPFKL---------IG-DSA---------EYTCDALIIATGASAKY-LGLE--SEE--
THICA00974      --RPFKL---------VG-DNS---------EYTCDALIIATGASAKY-LGLE--SET--
THIV600851      --RPFRL---------TG-DAA---------VYTCDALIVATGASAMY-LGLP--SEE--
THISH01973      --RPFTL---------TG-DSG---------SYTCDALIIATGASAMY-LGLP--SEE--
METAA00813      --RPYKL---------TG-DSG---------VYTCDALIIATGASAKY-LGME--SEE--
META200813      --RPYKL---------TG-DSG---------VYTCDALIIATGASAKY-LGME--SEE--
METMM02895      --RPFTL---------TG-DSG---------VYTCDALIIATGASAKY-LGLP--SEE--
FRAAD00813      --RPFKL---------IG-DSG---------EYTADALIITTGATAKY-LGIP--SEE--
XYLFA01416      --RPFTL---------FG-DSG---------LYTCDGLIIATGANAKY-LGIP--SEE--
XYLFT00623      --RPFTL---------FG-DSG---------IYTCDGLIIATGANAKY-LGIP--SEE--
XYLF200667      --RPFTL---------FG-DSG---------IYTCDGLIIATGANAKY-LGIP--SEE--
XYLFG01622      --RPFTL---------FG-DSG---------IYTCDGLIIATGANAKY-LGIP--SEE--
XYLFM00690      --RPFTL---------FG-DSG---------LYTCDGLIIATGANAKY-LGIP--SEE--
PSEUP01680      --RPFRL---------IG-DSA---------EYTCDALIIATGATARY-LGLE--SEE--
```

STRM501972	--RPFRL---------IG-DSH---------EYTCDALIIATGATAKY-LGIP--TED--
PSEUU01524	--RPFRL---------VG-DSA---------EYTCDALIIATGATAKY-LGIE--SEE--
XANAP01407	--RPFRL---------SG-DSG---------DYTCDALIIATGATAKY-LGIP--SEE--
XANCP01918	--RPFRL---------IG-DSA---------EYTCDALIIATGATAKY-LGIP--TEE--
XANC802154	--RPFRL---------IG-DSA---------EYTCDALIIATGATAKY-LGIP--TEE--
XANCB02263	--RPFRL---------IG-DSA---------EYTCDALIIATGATAKY-LGIP--TEE--
XANOR02379	--RPFRL---------TG-DGA---------EYTCDALIIATGATAKY-LGIP--TEE--
XANOM02295	--RPFRL---------TG-DGA---------EYTCDALIIATGATAKY-LGIP--TEE--
XANOP02318	--RPFRL---------TG-DGA---------EYTCDALIIATGATAKY-LGIP--TEE--
XANAC01951	--RPFRL---------IG-DGA---------EYTCDALIIATGATAKY-LGIP--TEE--
CYCSP01155	--RPFTL---------KG-DAG---------TYTCDALIIATGASAKY-LGLP--SEE--
GEOLS03275	--RPFIL---------QG-DGG---------RYSCDALIIATGASARY-LGLP--SEE--
GEOUR03858	--RPFVL---------EG-DSG---------TYSCDALIIATGASAKY-LGLP--SEE--
GEODF00692	--RPFIL---------EG-DSG---------TYSCDALIIATGASAQY-LGLP--SEQ--
GEOSL00482	--RPFRL---------EG-DSG---------IYTCDALIIATGASARY-LGLP--SEE--
GEOSK00469	--RPFRL---------EG-DSG---------IYTCDALIIATGASARY-LGLP--SEE--
GEOMG02998	--RPFRL---------EG-DNG---------IYTCDALIIATGATARY-LGLP--SED--
MORCR00228	--RPFTL---------VG-DKG---------VYTCDALIIATGATAQY-LGLE--SEQ--
ACIAD00798	--RPFVL---------KG-DMD---------EYTCDALIIATGATAQY-LGLE--SEA--
ACIBC00821	--RPFVL---------KG-DMD---------EYTCDALIIATGATAQY-LGLE--SEQ--
ACIBY02725	--RPFVL---------KG-DMD---------EYTCDALIIATGATAQY-LGLE--SEQ--
ACIB302691	--RPFVL---------KG-DMD---------EYTCDALIIATGATAQY-LGLE--SEQ--
ACIB500882	--RPFVL---------KG-DMD---------EYTCDALIIATGATAQY-LGLE--SEQ--
ACIB100850	--RPFVL---------KG-DMD---------EYTCDALIIATGATAQY-LGLE--SEQ--
ACIBD00843	--RPFVL---------KG-DMD---------EYTCDALIIATGATAQY-LGLE--SEQ--
ACIBS02229	--RPFVL---------KG-DMD---------EYTCDALIIATGATAQY-LGLE--SEQ--
ACICP00137	--RPFVL---------KG-DME---------EYTCDALIIATGATAQY-LGLE--SEQ--
ACISD03052	--RPFVL---------KG-DME---------EYTCDALIIATGATAQY-LGLE--SEQ--
LEGLN02402	--PPFVL---------QG-DSE---------TYTCDALIIATGASARY-LGLE--SES--
LEGPA01726	--APFTL---------QG-DSA---------TYTCDALIIATGASARY-LGLP--SET--
LEGPH00846	--APFTL---------QG-DST---------TYTCDALIIATGASARY-LGLP--SET--
LEGPC01171	--APFTL---------QG-DST---------TYTCDALIIATGASARY-LGLP--SET--
LEGP201857	--APFTL---------QG-DST---------TYTCDALIIATGASARY-LGLP--SET--
LEGPL01727	--APFTL---------QG-DST---------TYTCDALIIATGASARY-LGLP--SET--
COLP302668	--KPYKL---------TG-S-S---------EYTCDALIICTGASAQY-LGLP--SEE--
KANKD01106	--RPFTL---------TG-DSG---------TYTCDALIICTGASAKY-LGLP--SEE--
IDILO00659	--RPFQL---------KG-DVG---------EYTADSVIISTGASAKY-LGLD--SEE--
PSEU901284	--RPFTL---------TG-DQG---------TYTCDALIIATGASAKY-LGLE--SET--
PSEA602346	--RPFTL---------VG-DAG---------TYTCDSLIICTGASAKY-LGME--SET--
ALTSS01871	--RPFTL---------YG-DSG---------TYTCDALIIATGASAKY-LGME--SEQ--
ALTMD01611	--RPYTL---------YG-DSG---------TYTCDALIIATGASAKY-LGLE--SEQ--
ALTME01705	--RPFTL---------YG-DSG---------TYTCDALIIATGASAKY-LGLE--SEQ--
ALTMB01797	--RPFTL---------YG-DSG---------TYTCDALIIATGASAKY-LGLE--SEQ--
ALTMS01652	--RPFTL---------YG-DSG---------TYTCDALIIATGASAKY-LGLE--SEQ--
SACD201683	--RPFTL---------KG-N-N---------TYTCDALIISTGASAQY-LGLP--SEE--
TERTT01748	--TPKKL---------VG-N-E---------TYTCDALIIATGASAQY-LGLP--SEA--
SIMAS00609	--RPFLL---------KG-S-S---------TYSCDALIICTGASAQY-LGLP--SEE--
ALCDB01962	--RPFVL---------KG-DSG---------TYSCDALIIATGASAMY-LGLD--SEQ--
CHRSD02939	--RPFVL---------KG-ENG---------TYNCDALIISTGASARY-LGLP--SEE--
HALED02811	--RPFTL---------KG-DNG---------TYTCDALIIATGASARY-LGLE--SES--
SIDLE02655	--KPFLL---------IG-DAG---------SYTCDALIISTGASAQY-LGLP--SEE--
GALCS01621	--KPFLL---------EG-DAG---------SYTCDALIICTGASAQY-LGLP--SEE--
LARHH01446	--RPFKL---------TG-DAG---------EYTCDALIIATGASAQY-LGLP--SEE--
CHRVO02807	--KPIRL---------VG-DAG---------EYTCDALIIATGASAQY-LGLP--SEE--
PSEUL01648	--KPIRL---------VG-DAG---------EYTCDALIIATGASAQY-LGLA--SEE--
NITEU01859	--KPFEL---------IG-DQG---------TYTCDALIIATGASAKY-LGLP--SEE--
NITEC00745	--KSFEL---------TG-DQG---------TYTCDALIIATGASAKY-LGLP--SEE--
NITMU00023	--KPFTL---------IG-DQG---------TYTCDALIIATGASARY-LGMP--SEE--
NITSI03064	--KPITL---------TG-DQS---------TYTCDALIIATGASAQY-LGLP--SEE--
ACCPU02128	--RPFTL---------IG-DSA---------TYTCDALIIATGASAQY-LGLP--SEE--
THIDA01000	--KPFTL---------IG-DSA---------TYTCDALIIATGASAMY-LGLP--SEQ--
METS601221	--KPIRL---------IG-DSG---------EYTCDALIIATGASAQY-LGLP--SEE--
METGS01186	--KPIRL---------IG-DSG---------EYTCDALIIATGASAQY-LGLP--SEE--
METFK00972	--KPIRL---------VG-DAG---------EYTCDALIIATGASAKY-LGLP--SEE--
METML01428	--KPIRL---------VG-DSG---------EYTCDALIIATGASAKY-LGLP--SEE--

```
AROAE03899      --KPFRL---------VG-DAG---------EYTCDALIISTGATAKY-LGLP--SEE--
THASP01686      --KPFRL---------IG-DSG---------EYTCDALIISTGATAKY-LGLP--SEE--
AZOSB01359      --RPFRL---------IG-DAG---------EYTCDALIIATGATAKY-LGLP--SEE--
BORA102682      --RPFTL---------TG-DTG----N----VYTCDALIIATGASAKY-LGLP--SEE--
BORPA03415      --RPFTL---------TG-DTG----N----LYTCDALIIATGASAKY-LGLA--SEQ--
BORBM03577      --RPFTL---------TG-DTG----N----LYTCDALIIATGASAKY-LGLP--SEQ--
BORPE02280      --RPFTL---------TG-DTG----N----LYTCDALIIATGASAKY-LGLA--SEQ--
BORPC02060      --RPFTL---------TG-DTG----N----LYTCDALIIATGASAKY-LGLA--SEQ--
BORP102392      --RPFTL---------TG-DTG----N----LYTCDALIIATGASAKY-LGLA--SEQ--
BORBR03870      --RPFTL---------TG-DTG----N----LYTCDALIIATGASAKY-LGLA--SEQ--
BORPD01524      --RPFTL---------TG-DTG----N----VYTCDALIIATGASAKY-LGLP--SEE--
ACHXA01179      --RPFTL---------TG-DTG----K----VYTCDALIIATGASAKY-LGLP--SEE--
RHOFT03114      --RPFTL---------KG-DSG---------EYTCDALVLATGASAQY-LGLA--SET--
VEREI01701      --RPFTL---------SG-DSG---------TYTCDALILATGASAKY-LGLP--SEQ--
VARPE01375      --RPFTL---------TG-DSG---------TYTCDSLIIATGASAKY-LGLD--SEE--
VARPS01266      --RPFTL---------TG-DSG---------TYTCDSLIIATGASAKY-LGID--SEQ--
DELAS05272      --RPFTL---------TG-DSG---------TYTCDSLIIATGASAKY-LGLP--SEE--
DELSC01235      --RPFTL---------TG-DSG---------TYTCDSLIIATGASAKY-LGLP--SEE--
COMT200883      --RPFTL---------TG-DSG---------VYTCDSLIIATGASAKY-LGLP--SEE--
ACIAC03267      --RPFTL---------TG-DSG---------TYTCDALIIATGASAKY-LGLP--SEE--
ACIAP03249      --RPFTL---------TG-DSG---------TYTCDSLIIATGASAKY-LGLP--SEE--
ACIET02648      --RPFTL---------TG-DSG---------TYTCDSLIIATGASAKY-LGLP--SEE--
ALIDK03528      --RPFTL---------TG-DSG---------EYTCDALIIATGASAKY-LGLP--SEE--
RUBGI03408      --RPFVL---------TG-DSG---------RYTCDTLIVATGASAKY-LGLP--SEQ--
LEPCP00749      --RPFRL---------KG-DSG---------TYSCDALVIATGASAKY-LGLP--SEE--
POLSJ03719      --RPFTL---------TG-DSG---------TYTCDTLIIATGASAKY-LGLE--SES--
POLNA03144      --RPFTL---------TG-DSG---------TYTCDTLIIATGASAKY-LGLE--SET--
METPP01110      --RPFTL---------TG-DSG---------RYTCDALILATGASAMY-LGLP--SEE--
RAMTT03218      --RPFTL---------KG-DSG---------EYTCDALILATGASARY-LGLP--SEQ--
RALPJ02457      --KPIRL---------VG-DSG---------EYTCDALIVSTGASAQY-LGLP--SEE--
RALP102102      --KPIRL---------VG-DSG---------EYTCDALIVSTGASAQY-LGLP--SEE--
RALSO02303      --KPIRL---------VG-DSG---------EYTCDALIISTGASAQY-LGLP--SEE--
RALS801097      --KPIRL---------VG-DSG---------EYTCDALIISTGASAQY-LGLP--SEE--
HERSS01878      --RPFRL---------IG-DSG---------EYTADALIIATGASAQY-LGLP--SEQ--
HERAR00955      --KPIRL---------IG-DMG---------EYTCDSLIIATGASAQY-LGLP--SEQ--
JANMA01152      --KPIRL---------IG-DAG---------EYTCDSLIIATGASAQY-LGLP--SEQ--
THIK102324      --KPIRL---------EG-DSG---------VYTCDTLIIATGASAKY-LGLP--TEE--
POLSQ00682      --KPIRL---------VG-DSG---------TYTCDALIICTGASAQY-IGLP--SEE--
POLNS00989      --KPIRL---------VG-DSG---------TYTCDALIICTGASAQY-IGLP--SEE--
BURP800627      --KPIRL---------IG-DSG---------EYTCDSLIIATGASAQY-LGLP--SEE--
BURPP00988      --KPIRL---------IG-DSG---------EYTCDSLIISTGASAQY-LGLP--SEE--
BURSC00728      --KPIRL---------IG-DSG---------EYTCDSLIISTGASAQY-LGLP--SEE--
BURXL00982      --KPIRL---------IG-DSG---------EYTCDSLIIATGASAQY-LGLP--SEE--
BURSG00869      --KPIRL---------IG-DSG---------EYTCDSLIISTGASAQY-LGLP--SEE--
BURRH00629      --RPLRL---------IG-DSG---------EYTCDALIIATGASAQY-LGLP--SEQ--
BURGB00781      --KPIRL---------IG-DSG---------EYTCDALIISTGASAQY-LGLA--SEE--
BURGS00853      --KPIRL---------IG-DSG---------EYTCDSLIISTGASAQY-LGLA--SEE--
BURPS02618      --KPIRL---------IG-DSG---------EYTCDSLIIATGASAQY-LGLQ--SEE--
BURMA01741      --KPIRL---------IG-DSG---------EYTCDSLIIATGASAQY-LGLQ--SEE--
BURP103016      --KPIRL---------IG-DSG---------EYTCDSLIIATGASAQY-LGLQ--SEE--
BURP002984      --KPIRL---------IG-DSG---------EYTCDSLIIATGASAQY-LGLQ--SEE--
BURM701866      --KPIRL---------IG-DSG---------EYTCDSLIIATGASAQY-LGLQ--SEE--
BURP602945      --KPIRL---------IG-DSG---------EYTCDSLIIATGASAQY-LGLQ--SEE--
BURM902465      --KPIRL---------IG-DSG---------EYTCDSLIIATGASAQY-LGLQ--SEE--
BURMS00740      --KPIRL---------IG-DSG---------EYTCDSLIIATGASAQY-LGLQ--SEE--
BURTA01515      --KPIRL---------VG-DSG---------EYTCDSLIIATGASAQY-LGLP--SEE--
BURM102386      --RPIRL---------IG-DSG---------EYTCDALIIATGASAQY-LGLP--SEE--
BURL300890      --QPIRL---------IG-DSG---------EYTCDSLIIATGASAQY-LGLP--SEE--
BURVG00860      --KPIRL---------IG-DAG---------EYTCDALIIATGASAQY-LGLP--SEE--
BURCM00829      --QPIRL---------IG-DSG---------EYTCDSLIIATGASAQY-LGLP--SEE--
BURA400832      --QPIRL---------IG-DSG---------EYTCDSLIIATGASAQY-LGLP--SEE--
BURCA00489      --QPIRL---------IG-DSG---------EYTCDSLIIATGASAQY-LGLP--SEE--
BURCH00966      --QPIRL---------IG-DSG---------EYTCDSLIIATGASAQY-LGLP--SEE--
BURCC00927      --QPIRL---------IG-DSG---------EYTCDSLIIATGASAQY-LGLP--SEE--
BURCJ02922      --KPIRL---------IG-DSG---------EYTCDSLIIATGASAQY-LGLP--SEE--
```

```
EDWI902348      --RPFRL---------FG-DSQ---------EYSCDALIVATGASARY-LGLP--SEE--
EDWTF01971      --RPFRL---------FG-DSQ---------EYSCDALIVATGASARY-LGLP--SEE--
EDWTE02176      --RPFRL---------FG-DSQ---------EYSCDALIVATGASARY-LGLP--SEE--
SODGM01094      --RPFRL---------VG-DSG---------EYTCDALIIATGASARY-LGLP--SEE--
MOREP00394      --RPFSL---------VG-DSG---------EYTCDALIIATGASARY-LGLP--SEE--
RAHSY01432      --RPFRL---------TG-DSG---------EYTCDSLIIATGASARY-IGLP--SEE--
RAHAC01419      --RPFRL---------TG-DSG---------EYTCDALIIATGASARY-IGLP--SEE--
ERWBE01489      --RPFRL---------TG-DSA---------EYTADALIIATGASARY-LGLP--SEE--
PANAM01339      --RPFRL---------TG-DSG---------EYTADALIIATGASARY-LGLP--SED--
PANAA00662      --RPFRL---------TG-DSG---------EYTADALIIATGASARY-LGLP--SED--
PANSA01303      --RPFRL---------TG-DSG---------EYTADALIIATGASARY-LGLP--SEE--
ERWT902154      --RPFRL---------IG-DEG---------EYTADALIIATGASARY-LGLP--SEE--
ERWAC01330      --RPFRL---------TG-DEG---------EYTADALIIATGASARY-LGLP--SEE--
ERWAE01326      --RPFRL---------TG-DEG---------EYTADALIIATGASARY-LGLP--SEE--
ERWPE02241      --RPFRL---------TG-DEG---------EYTADALIIATGASARY-LGLP--SEE--
ERWP602414      --RPFRL---------TG-DEG---------EYTADALIIATGASARY-LGLP--SEE--
ERWSE02396      --RPFRL---------TG-DEG---------EYTADALIIATGASARY-LGLP--SEE--
PECCP01694      --RPFRL---------FG-DSD---------EYTCDALIIATGASARY-LGLP--SEE--
PECWW01889      --RPFRL---------FG-DSD---------EYTCDALIIATGASARY-LGLP--SED--
PECSS01870      --RPFRL---------FG-DSD---------EYTCDALIIATGASARY-LGLP--SED--
PECAS02624      --RPFRL---------FG-DSA---------EYTCDALIIATGASARY-LGLP--SED--
DICDC02220      --RPFRL---------FG-DSD---------EYTCDALIIATGASARY-LGLP--SED--
DICZE02284      --RPFRL---------FG-DSD---------EYTCDALIIATGASARY-LGLP--SED--
DICD302017      --RPFRL---------FG-DSD---------EYTCDALIIATGASARY-LGLP--SED--
DICD502250      --RPFRL---------FG-DSD---------EYTCDALIIATGASARY-LGLP--SED--
XENBS00829      --KPFRL---------YG-DDQ---------EYTCDALIIATGASARY-LGLP--SED--
XENNA01480      --RPFHL---------YG-DDQ---------EYTCDALIIATGASARY-LGLP--SED--
PHOLL01537      --RPLRL---------YG-DEQ---------EYTCDALIIATGASARY-LGLP--SEE--
PHOAA02804      --RPFRL---------YG-DEQ---------EYTCDALIIATGASARY-LGLP--SED--
SERP501669      --RPFRL---------FG-DSG---------EYTCDALIIATGASARY-LGLP--TEE--
SERSA01625      --RPFRL---------FG-DSG---------EYTCDALIIATGASARY-LGMP--SED--
YERPE01271      --RPFRL---------FG-DGA---------EYTCDALIIATGASARY-LGMA--SEE--
YERPS01390      --RPFRL---------FG-DGA---------EYTCDALIIATGASARY-LGMA--SEE--
YERPA00627      --RPFRL---------FG-DGA---------EYTCDALIIATGASARY-LGMA--SEE--
YERPN02489      --RPFRL---------FG-DGA---------EYTCDALIIATGASARY-LGMA--SEE--
YERPP02190      --RPFRL---------FG-DGA---------EYTCDALIIATGASARY-LGMA--SEE--
YERP302544      --RPFRL---------FG-DGA---------EYTCDALIIATGASARY-LGMA--SEE--
YERPB01460      --RPFRL---------FG-DGA---------EYTCDALIIATGASARY-LGMA--SEE--
YERPY02633      --RPFRL---------FG-DGA---------EYTCDALIIATGASARY-LGMA--SEE--
YERPG01392      --RPFRL---------FG-DGA---------EYTCDALIIATGASARY-LGMA--SEE--
YERPD01171      --RPFRL---------FG-DGA---------EYTCDALIIATGASARY-LGMA--SEE--
YERP100890      --RPFRL---------FG-DGA---------EYTCDALIIATGASARY-LGMA--SEE--
YERPZ01206      --RPFRL---------FG-DGA---------EYTCDALIIATGASARY-LGMA--SEE--
YERPH02469      --RPFRL---------FG-DGA---------EYTCDALIIATGASARY-LGMA--SEE--
YERE801438      --RPFRL---------FG-DSA---------EYTCDALIIATGASARY-LGME--SEE--
YERE302565      --RPFRL---------FG-DSA---------EYTCDALIIATGASARY-LGME--SEE--
YERE100392      --RPFRL---------FG-DSA---------EYTCDALIIATGASARY-LGME--SEE--
PROMH00687      --RPFRL---------FG-DEQ---------EYTCDALIIATGASARY-IGLP--SEE--
PROSM03196      --RPFRL---------FG-DEN---------EYTCDALIIATGASARY-IGLP--SEE--
TOLAT02279      --KPFRL---------KG-DNG---------EYTCDALIIATGASARY-LGLD--SEE--
AERVB02262      --RPFRL---------KG-DNG---------EYTCDALIIATGASAKY-LGLP--SEE--
AERHH01811      --RPFRL---------KG-DNG---------EYTCDALIIATGASAKY-LGLP--SEE--
AERS402185      --RPFRL---------KG-DSA---------EYTCDALIIATGASAKY-LGLP--SEE--
PSYIN02070      --SPFTL---------KG-DSD---------TYSCDALIIATGASATY-LGLD--SED--
SHELP02018      --RPFQL---------KG-DSG---------EYTCDALIISTGASAMY-LGLE--SEE--
SHEVD02297      --RPFRL---------KG-DNG---------EFTCDALIISTGASAMY-LGLD--SEE--
SHEPW02341      --RPFQL---------KG-DNG---------EFTCDALIISTGASAMY-LGLD--SEE--
SHEPA02202      --RPFQL---------KG-DNG---------EFTCDSLIISTGASAIY-LGLE--SEE--
SHEHH02007      --RPFKL---------KG-DNG---------EFTCDSLIISTGASAMY-LGLE--SEE--
SHESH02117      --RPFKL---------KG-DNG---------DFTCDALIISTGASAMY-LGLE--SEE--
SHEWM02454      --RPFKL---------KG-DNG---------EFTCDALIISTGASAMY-LGLD--SEE--
SHEAM01758      --RPFRL---------KG-DNG---------EYTCDALIIATGASAKY-LGLP--SED--
SHEON02159      --RPFRL---------KG-DNG---------EYTCDALIIATGASARY-LGLE--SEE--
SHESM01945      --RPFRL---------KG-DNG---------EYTCDALIIATGASARY-LGLE--SEE--
SHESR01991      --RPFRL---------KG-DNG---------EYTCDALIIATGASARY-LGLE--SEE--
```

```
SHESA02025      --RPFRL---------KG-DNG---------EYTCDALIIATGASARY-LGLE--SEE--
SHESW01953      --RPFRL---------KG-DNG---------EYSCDALIIATGASARY-LGLE--SEE--
SHEPC01976      --RPFRL---------KG-DNG---------EYSCDALIIATGASARY-LGLE--SEE--
SHEP201929      --RPFRL---------KG-DNG---------EYSCDALIIATGASARY-LGLE--SEE--
SHEB502016      --RPFRL---------KG-DNG---------EYTCDALIIATGASARY-LGLE--SEE--
SHEB802168      --RPFRL---------KG-DNG---------EYTCDALIIATGASARY-LGLE--SEE--
SHEB202117      --RPFRL---------KG-DNG---------EYTCDALIIATGASARY-LGLE--SEE--
SHEB902270      --RPFRL---------KG-DNS---------EYTCDALIIATGASARY-LGLE--SEE--
SHEB602258      --RPFRL---------KG-DNS---------EYTCDALIIATGASARY-LGLE--SEE--
SHEDO01680      --RPFRL---------KG-DNG---------EYTCDALIIATGASAKY-LGLP--SEE--
SHEFN01895      --RPFRL---------KG-DNG---------EFTCDALIIATGASAKY-LGLP--SEE--
PSEHT01677      --RPFTL---------TG-DQG---------TYTCDALIIATGASAKY-LGLE--SET--
ALISL01611      --RPFRL---------KG-DSS---------EYTCDALIISTGASAKY-LGLE--SEE--
VIBF100893      --RPFRL---------KG-DSG---------EYTCDALIISTGASAKY-LGLE--SEE--
VIBFM00891      --RPFRL---------KG-DSC---------EYTCDALIISTGASAKY-LGLE--SEE--
OCESG01399      --RPFRL---------KG-DTG---------EYTCDALIIATGASAKY-LGLP--SEE--
VIBA701872      --RPFRL---------KG-DSA---------EYSCDALIISTGASARY-LGLD--SEE--
VIBVY01451      --RPFVL---------KG-DAA---------SYSCDALIISTGASAKY-LGLE--SEE--
VIBVU02545      --RPFVL---------KG-DAA---------SYSCDALIISTGASAKY-LGLE--SEE--
VIBVM01748      --RPFVL---------KG-DAA---------SYSCDALIISTGASAKY-LGLE--SEE--
VIBCH01157      --RPFRL---------TG-DSQ---------EYTCDALIISTGASAKY-LGLE--SEE--
VIBCM01114      --RPFRL---------TG-DSQ---------EYTCDALIISTGASAKY-LGLE--SEE--
VIBCJ02064      --RPFRL---------TG-DSQ---------EYTCDALIISTGASAKY-LGLE--SEE--
VIBC300725      --RPFRL---------TG-DSL---------EYTCDALIISTGASAKY-LGLE--SEE--
FERBD01969      --RPYRL---------KG-DSG---------EYTCDALVIATGASAKY-LGLE--SET--
VIBFN02085      --RPYRL---------KG-DSG---------EYTCDALIITTGASAKY-LGLD--SEE--
VIBTL01089      --RPFRL---------KG-DSG---------EYTCDALIISTGASAKY-LGLD--SEE--
VIBPA01248      --RPFRL---------KG-DSG---------EYTCDALIISTGASAKY-LGLE--SEE--
VIBAE02128      --RPFRL---------KG-ESG---------EYTCDALIISTGASAKY-LGLE--SEE--
VIBCB00599      --RPFRL---------KG-DSG---------EYTCDALIISTGASAKY-LGLE--SEE--
PANVC00701      --RPFTL---------TG-DSG---------VYTADALIIATGASARY-LGLP--SEE--
SHIBC02443      --RPFRL---------TG-DNA---------EYTCDALIIATGASARY-LGLP--SEE--
ENTBF02792      --RPFRL---------IG-DSG---------EYTCDALIIATGASARY-LGLP--SED--
KLEP700892      --RPFRL---------TG-DSG---------EYTCDALIIATGASARY-LGLP--SEE--
KLEPH01782      --RPFRL---------TG-DSG---------EYTCDALIIATGASARY-LGLP--SEE--
KLEP303551      --RPFRL---------TG-DSG---------EYTCDALIIATGASARY-LGLP--SEE--
KLEVT03420      --RPFRL---------TG-DSG---------EYTCDALIIATGASARY-LGLP--SEE--
ENTAK02985      --RPFRL---------VG-DSG---------EYTCDALIIATGASARY-LGLP--SEE--
KLEOK03151      --RPFRL---------VG-DSG---------EYTCDALIIATGASARY-LGLP--SEE--
SALAR01925      --RPFRL---------KG-DSG---------EYTCDALIIATGASARY-LGLP--SEE--
SALBC00812      --RPFRL---------TG-DSG---------EYTCDALIIATGASARY-LGLP--SEE--
SALPC00926      --RPFRL---------TG-DSA---------EYTCDALIIATGASARY-LGLP--SEE--
SALTI00868      --RPFRL---------TG-DSA---------EYTCDALIIATGASARY-LGLP--SEE--
SALCH00905      --RPFRL---------TG-DSA---------EYTCDALIIATGASARY-LGLP--SEE--
SALPA01706      --RPFRL---------TG-DSA---------EYTCDALIIATGASARY-LGLP--SEE--
SALTY00927      --RPFRL---------TG-DSA---------EYTCDALIIATGASARY-LGLP--SEE--
SALPK01786      --RPFRL---------TG-DSA---------EYTCDALIIATGASARY-LGLP--SEE--
SALHS00978      --RPFRL---------TG-DSA---------EYTCDALIIATGASARY-LGLP--SEE--
SALEP00854      --RPFRL---------TG-DSA---------EYTCDALIIATGASARY-LGLP--SEE--
SALDC00926      --RPFRL---------TG-DSA---------EYTCDALIIATGASARY-LGLP--SEE--
SALA400892      --RPFRL---------TG-DSA---------EYTCDALIIATGASARY-LGLP--SEE--
SALG200876      --RPFRL---------TG-DSA---------EYTCDALIIATGASARY-LGLP--SEE--
SALTS00888      --RPFRL---------TG-DSA---------EYTCDALIIATGASARY-LGLP--SEE--
SALT400905      --RPFRL---------TG-DSA---------EYTCDALIIATGASARY-LGLP--SEE--
SALPS01908      --RPFRL---------TG-DSA---------EYTCDALIIATGASARY-LGLP--SEE--
SALT101040      --RPFRL---------TG-DSA---------EYTCDALIIATGASARY-LGLP--SEE--
SALTD00960      --RPFRL---------TG-DSA---------EYTCDALIIATGASARY-LGLP--SEE--
SALPB02472      --RPFRL---------TG-DSA---------EYTCDALIIATGASARY-LGLP--SEE--
SALNS00928      --RPFRL---------TG-DSA---------EYTCDALIIATGASARY-LGLP--SEE--
SALSV01000      --RPFRL---------TG-DSA---------EYTCDALIIATGASARY-LGLP--SEE--
ECOS500812      --RPFRL---------TG-DSG---------EYTCDALIIATGASARY-LGLP--SEE--
ECOL600981      --RPFRL---------TG-DSG---------EYTCDALIIATGASARY-LGLP--SEE--
ECOL500883      --RPFRL---------TG-DSG---------EYTCDALIIATGASARY-LGLP--SEE--
ECOUT00876      --RPFRL---------TG-DSG---------EYTCDALIIATGASARY-LGLP--SEE--
ECOK100776      --RPFRL---------TG-DSG---------EYTCDALIIATGASARY-LGLP--SEE--
```

```
ECOSM02148        --RPFRL---------TG-DSG---------EYTCDALIIATGASARY-LGLP--SEE--
ECOLU01057        --RPFRL---------TG-DSG---------EYTCDALIIATGASARY-LGLP--SEE--
ECO7I02136        --RPFRL---------TG-DSG---------EYTCDALIIATGASARY-LGLP--SEE--
ECO8100828        --RPFRL---------TG-DSG---------EYTCDALIIATGASARY-LGLP--SEE--
ECO4500862        --RPFRL---------TG-DSG---------EYTCDALIIATGASARY-LGLP--SEE--
ECOAB00879        --RPFRL---------TG-DSG---------EYTCDALIIATGASARY-LGLP--SEE--
ECO4400961        --RPFRL---------TG-DSG---------EYTCDALIIATGASARY-LGLP--SEE--
ECOUM02550        --RPFRL---------TG-DSG---------EYTCDALIIATGASARY-LGLP--SEE--
ECOKI00869        --RPFRL---------TG-DSG---------EYTCDALIIATGASARY-LGLP--SEE--
ECOC100920        --RPFRL---------TG-DSG---------EYTCDALIIATGASARY-LGLP--SEE--
ECOC200920        --RPFRL---------TG-DSG---------EYTCDALIIATGASARY-LGLP--SEE--
ECO8N00803        --RPFRL---------TG-DSG---------EYTCDALIIATGASARY-LGLP--SEE--
ECO2700862        --RPFRL---------NG-DSG---------EYTCDALIIATGASARY-LGLP--SEE--
ECO2600974        --RPFRL---------NG-DNG---------EYTCDALIIATGASARY-LGLP--SEE--
ECOH100942        --RPFRL---------NG-DNG---------EYTCDALIIATGASARY-LGLP--SEE--
SHIB301848        --RPFRL---------NG-DNG---------EYTCDALIIATGASARY-LGLP--SEE--
ECOLI00846        --RPFRL---------NG-DNG---------EYTCDALIIATGASARY-LGLP--SEE--
ECO5700970        --RPFRL---------NG-DNG---------EYTCDALIIATGASARY-LGLP--SEE--
SHISS00756        --RPFRL---------NG-DNG---------EYTCDALIIATGASARY-LGLP--SEE--
SHIBS00679        --RPFRL---------NG-DNG---------EYTCDALIIATGASARY-LGLP--SEE--
SHIDS01857        --RPFRL---------NG-DNG---------EYTCDALIIATGASARY-LGLP--SEE--
ECO2400906        --RPFRL---------NG-DNG---------EYTCDALIIATGASARY-LGLP--SEE--
ECODH00784        --RPFRL---------NG-DNG---------EYTCDALIIATGASARY-LGLP--SEE--
ECOHS00922        --RPFRL---------NG-DNG---------EYTCDALIIATGASARY-LGLP--SEE--
ECOLC02627        --RPFRL---------NG-DNG---------EYTCDALIIATGASARY-LGLP--SEE--
ECO5E00946        --RPFRL---------NG-DNG---------EYTCDALIIATGASARY-LGLP--SEE--
ECOSE00936        --RPFRL---------NG-DNG---------EYTCDALIIATGASARY-LGLP--SEE--
ECO5500894        --RPFRL---------NG-DNG---------EYTCDALIIATGASARY-LGLP--SEE--
ECO8A00877        --RPFRL---------NG-DNG---------EYTCDALIIATGASARY-LGLP--SEE--
ECOBB00863        --RPFRL---------NG-DNG---------EYTCDALIIATGASARY-LGLP--SEE--
ECO5T00943        --RPFRL---------NG-DNG---------EYTCDALIIATGASARY-LGLP--SEE--
ECOBW00723        --RPFRL---------NG-DNG---------EYTCDALIIATGASARY-LGLP--SEE--
ECO1000919        --RPFRL---------NG-DNG---------EYTCDALIIATGASARY-LGLP--SEE--
ECOBD02604        --RPFRL---------NG-DNG---------EYTCDALIIATGASARY-LGLP--SEE--
ECOD102659        --RPFRL---------NG-DNG---------EYTCDALIIATGASARY-LGLP--SEE--
ECOBR00856        --RPFRL---------NG-DNG---------EYTCDALIIATGASARY-LGLP--SEE--
ECOLX02610        --RPFRL---------NG-DNG---------EYTCDALIIATGASARY-LGLP--SEE--
ECO1A00939        --RPFRL---------NG-DNG---------EYTCDALIIATGASARY-LGLP--SEE--
ECOCB01054        --RPFRL---------NG-DNG---------EYTCDALIIATGASARY-LGLP--SEE--
ECOKO02859        --RPFRL---------NG-DNG---------EYTCDALIIATGASARY-LGLP--SEE--
ECO1E03320        --RPFRL---------NG-DNG---------EYTCDALIIATGASARY-LGLP--SEE--
ECOLW01217        --RPFRL---------NG-DNG---------EYTCDALIIATGASARY-LGLP--SEE--
SHIFL01399        --RPFRL---------NG-DNG---------EYTCDALIIATGASARY-LGLP--SEE--
SHIF800759        --RPFRL---------NG-DNG---------EYTCDALIIATGASARY-LGLP--SEE--
SHIF200805        --RPFRL---------NG-DNG---------EYTCDALIIATGASARY-LGLP--SEE--
CITK802118        --RPFRL---------TG-DSG---------EYTCDALIIATGASARY-LGLP--SEE--
CITRI00924        --RPFRL---------TG-DSA---------EYTCDALIIATGASARY-LGLP--SEE--
CROS802385        --RPFRL---------IG-DSG---------EYTCDALIIATGASARY-LGLP--SEE--
CROTZ01500        --RPFRL---------IG-DSG---------EYTCDALIIATGASARY-LGLP--SEE--
ENTLS02865        --RPFRL---------TG-DSG---------EYTCDALIIATGASARY-LGLP--SEE--
ENT3801400        --RPFRL---------TG-DNG---------EYTCDALIIATGASARY-LGLP--SEE--
ENTAL01384        --RPFRL---------TG-DSG---------EYTCDALIIATGASARY-LGLP--SEE--
ENTCC02679        --RPFRL---------TG-DSG---------EYTCDALIIATGASARY-LGLP--SEE--
                                                        :   *

STRT101547        EYN--SRGVSYCAVCDGA--FFRNQ-DLLVVGGGDSAVEEGIYLTRFANSVTIVHRRDE-
STRT201508        EYN--SRGVSYCAVCDGA--FFRNQ-DLLVVGGGDSAVEEGIYLTRFANSVTIVHRRDE-
STRTD01356        EYN--SRGVSYCAVCDGA--FFRNQ-DLLVVGGGDSAVEEGIYLTRFANSVTIVHRRDE-
STRTN01533        EYN--SRGVSYCAVCDGA--FFRNQ-DLLVVGGGDSAVEEGIYLTRFANSVTIVHRRDE-
STRE500345        EYN--SRGVSYCAVCDGA--FFRNQ-DLLVVGGGDSAVEEGIYLTRFANSVTIVHRRDE-
STRE801625        EYN--SRGVSYCAVCDGA--FFRNQ-DLLVVGGGDSAVEEGIYLTRFANSVTIVHRRDE-
STREH01636        EYN--SRGVSYCAVCDGA--FFRNQ-DLLVVGGGDSAVEEGIYLTRFANSVTIVHRRDE-
STREC01656        EYT--SRGVSYCAVCDGA--FFRNQ-ELLVVGGGDSAVEEAIYLTQFAKKVTIVHRRNQ-
STREM01495        EYT--SRGVSYCAVCDGA--FFRNQ-ELLVVGGGDSAVEEAIYLTQFAKKVTIVHRRDQ-
STRE401629        EYT--SRGVSYCAVCDGA--FFRNQ-ELLVVGGGDSAVEEAIYLTQFAKKVTIVHRRDQ-
```

```
STRS700387      EYT--SRGVSYCAVCDGA--FFRNQ-ELLVVGGGDSAVEEAIYLTQFAKKVTIVHRRDQ-
STRDG01631      DYT--SRGVSYCAVCDGA--FFRNQ-DLLVVGGGDSAVEEAIYLTQFAKKVTVVHRRDQ-
STRP301388      YYT--SRGVSYCAVCDGA--FFRDQ-DLLVVGGGDSAVEEAIYLTQFAKKVTVVHRRDQ-
STRPZ01205      YYT--SRGVSYCAVCDGA--FFRDQ-DLLVVGGGDSAVEEAIYLTQFAKKVTVVHRRDQ-
STRPQ00464      YYT--SRGVSYCAVCDGA--FFRDQ-DLLVVGGGDSAVEEAIYLTQFAKKVTVVHRRDQ-
STRPD01453      YYT--SRGVSYCAVCDGA--FFRDQ-DLLVVGGGDSAVEEAIYLTQFAKKVTVVHRRDQ-
STRP601399      YYT--SRGVSYCAVCDGA--FFRDQ-DLLVVGGGDSAVEEAIYLTQFAKKVTVVHRRDQ-
STRP801366      YYT--SRGVSYCAVCDGA--FFRDQ-DLLVVGGGDSAVEEAIYLTQFAKKVTVVHRRDQ-
STRPF01453      YYT--SRGVSYCAVCDGA--FFRDQ-DLLVVGGGDSAVEEAIYLTQFAKKVTVVHRRDQ-
STRPG00427      YYT--SRGVSYCAVCDGA--FFRDQ-DLLVVGGGDSAVEEAIYLTQFAKKVTVVHRRDQ-
STRA300284      EYT--SRGVSYCAVCDGA--FFRDQ-DLLVVGGGDSAVEEAVFLTQFAKSVTIIHRRDQ-
STRA500290      EYT--SRGVSYCAVCDGA--FFRDQ-DLLVVGGGDSAVEEAVFLTQFAKSVTIIHRRDQ-
STRA100288      EYT--SRGVSYCAVCDGA--FFRDQ-DLLVVGGGDSAVEEAVFLTQFAKSVTIIHRRDQ-
STRA200291      EYT--SRGVSYCAVCDGA--FFRDQ-DLLVVGGGDSAVEEAVFLTQFAKSVTIIHRRDQ-
STRIC00348      EYT--SRGVSYCAVCDGA--FFRNQ-DLLVVGGGDSAVEEAGYLTQFAKSVTIIHRRDE-
STRPX00375      EYT--SRGVSYCAVCDGA--FFRNQ-DLLVVGGGDSAVEEAVYLTQFAKSVTIIHRRDE-
STRMD00432      EYT--SRGVSYCAVCDGA--FFRNQ-DLLVVGGGDSAVEEAVYLTQFAKSVTIIHRRDE-
STRS201734      TYN--SRGVSYCAVCDGA--FFRGQ-KLLVVGGGDSAVEEALFLTQFAESVTIVHRRDQ-
STRSY01721      TYN--SRGVSYCAVCDGA--FFRGQ-KLLVVGGGDSAVEEALFLTQFAESVTIVHRRDQ-
STRSX01553      TYN--SRGVSYCAVCDGA--FFRGQ-KLLVVGGGDSAVEEALFLTQFAESVTIVHRRDQ-
STRSE01478      TYN--SRGVSYCAVCDGA--FFRGQ-KLLVVGGGDSAVEEALFLTQFAESVTIVHRRDQ-
STREJ01635      TYN--SRGVSYCAVCDGA--FFRGQ-KLLVVGGGDSAVEEALFLTQFAESVTIVHRRDQ-
STRGZ01544      TYN--SRGVSYCAVCDGA--FFRGQ-KLLVVGGGDSAVEEALFLTQFAESVTIVHRRDQ-
STRS401593      TYN--SRGVSYCAVCDGA--FFRGQ-KLLVVGGGDSAVEEALFLTQFAESVTIVHRRDQ-
LACGT00720      EYG--ARGVSYCAVCDGA--FFRDQ-DILVVGGGDSAVEEAIFLTRFGKTVTIMHRRDE-
LACGL00738      EYG--ARGVSYCAVCDGA--FFRDQ-DILVVGGGDSAVEEAIFLTRFGKTVTIMHRRDE-
STRSV01774      DYN--SRGVSYCAVCDGA--FFRDE-DLLVVGGGDSAVEEAIFLTRFAKSVTIVHRRDE-
STRIJ00350      ELN--SRGVSYCAVCDGA--FFRDE-DLLVVGGGDSAVEEAVFLTRFAKTVTIIHRRNE-
STROU01248      ELN--SRGVSYCAVCDGA--FFRDQ-DLLVVGGGDSAVEEALFLTRFAKTVTIVHRRDE-
STRM601286      ELN--SRGVSYCAVCDGA--FFRDQ-DLLVVGGGDSAVEEALFLTRFAKTVTIVHRRDQ-
STRES00962      ELN--SRGVSYCAVCDGA--FFRDQ-DLLVVGGGDSAVEEALFLTRFAKTVTIVHRRDQ-
STRP701387      ELN--SRGVSYCAVCDGA--FFRDQ-DLLVVGGGDSAVEEALFLTRFAKTVTIVHRRDQ-
STRZT00756      ELN--SRGVSYCAVCDGA--FFRDQ-DLLVVGGGDSAVEEALFLTRFAKTVTIVHRRDQ-
STRP001079      ELN--SRGVSYCAVCDGA--FFRDQ-DLLVVGGGDSAVEEALFLTRFAKTVTIVHRRDQ-
STRZO01270      ELN--SRGVSYCAVCDGA--FFRDQ-DLLVVGGGDSAVEEALFLTRFAKTVTIVHRRDQ-
STRZ600815      ELN--SRGVSYCAVCDGA--FFRDQ-DLLVVGGGDSAVEEALFLTRFAKTVTIVHRRDQ-
STRET00826      ELN--SRGVSYCAVCDGA--FFRDQ-DLLVVGGGDSAVEEALFLTRFAKTVTIVHRRDQ-
STRPS01429      ELN--SRGVSYCAVCDGA--FFRDQ-DLLVVGGGDSAVEEALFLTRFAKTVTIVHRRDQ-
STRZN01287      ELN--SRGVSYCAVCDGA--FFRDQ-DLLVVGGGDSAVEEALFLTRFAKTVTIVHRRDQ-
STRR601306      ELN--SRGVSYCAVCDGA--FFRDQ-DLLVVGGGDSAVEEALFLTRFAKTVTIVHRRDQ-
STRP201217      ELN--SRGVSYCAVCDGA--FFRDQ-DLLVVGGGDSAVEEALFLTRFAKTVTIVHRRDQ-
STRZP01342      ELN--SRGVSYCAVCDGA--FFRDQ-DLLVVGGGDSAVEEALFLTRFAKTVTIVHRRDQ-
STRZI01226      ELN--SRGVSYCAVCDGA--FFRDQ-DLLVVGGGDSAVEEALFLTRFAKTVTIVHRRDQ-
STRPN01360      ELN--SRGVSYCAVCDGA--FFRDQ-DLLVVGGGDSAVEEALFLTRFAKTVTIVHRRDQ-
STRP401357      ELN--SRGVSYCAVCDGA--FFRDQ-DLLVVGGGDSAVEEALFLTRFAKTVTIVHRRDQ-
STRZJ01282      ELN--SRGVSYCAVCDGA--FFRDQ-DLLVVGGGDSAVEEALFLTRFAKTVTIVHRRDQ-
STRPJ01336      ELN--SRGVSYCAVCDGA--FFRDQ-DLLVVGGGDSAVEEALFLTRFAKTVTIVHRRDQ-
STRPI01436      ELN--SRGVSYCAVCDGA--FFRDQ-DLLVVGGGDSAVEEALFLTRFAKTVTIVHRRDQ-
MARHT00044      KFY--GRGVSTCATCDGF--FYKGK-RVVVVGGGDAAVEEGLFLTKFAEKVTIIHRRDE-
THEP300413      RFI--GAGISFCATCDGA--FYRDA-TVAVIGGGNTAVEDALYLTKFAKKVYIIHRRNE-
THEPX00884      RFI--GAGISFCATCDGA--FYRDA-TVAVIGGGNTAVEDALYLTKFAKKVYIIHRRNE-
THESX01857      RFI--GAGISFCATCDGA--FYRDA-TVAVIGGGNTAVEDALYLTKFAKKVYIIHRRNE-
THEM301788      RFI--GAGISFCATCDGA--FYRDA-TVAVIGGGNTAVEDALYLTKFAKKVYIIHRRNE-
THEIA01812      RFI--GAGISFCATCDGA--FYRDA-TVAVIGGGNTAVEDALYLTKFAKKVYIIHRRNE-
THETC00443      KFR--GSGVSYCATCDGA--FYKDQ-VVAVVGGGDTAMEDSNYLTKFAKKVYVIHRRDK-
THESW01026      KFR--GSGVSYCATCDGA--FYKDQ-VVAVVGGGDTAMEDSNYLTKFAKKVYVIHRRDK-
THEXL00360      KFR--GSGVSYCATCDGA--FYKDQ-VVAVVGGGDTAMEDSNYLTKFAKKVYVIHRRDK-
THEID01127      KLT--GRGVSYCATCDGP--FFRDQ-VVAVVGGGNTAVQEAIFLTRFASKVYLIHRRDQ-
THEOJ00147      EFT--GRGVSYCATCDGA--FYTDR-PVMVVGGGDTAIGEAIYLTHFATSVTVVHRRNE-
DESAS01237      TLH--GRGVSYCATCDGA--FFRGK-KVAVVGGGDAAVEEALFLTKFAEEVFIIHRRGE-
DESK701373      TFY--GRGVSYCATCDGA--FFKDK-HVAVVGGGDAAVEEAMFLTKFATRVFIIHRRGE-
KYRT200729      EFS--GRGVSYCAVCDGA--FFKNK-NLYVIGGGDSACEEGVYLTRHAAKVTIVHRRDK-
STACT00410      ELG--GRGVSYCAVCDGA--FFKGK-KLFVIGGGDSAVEEGAFLTKFADSVTVVHRRDK-
STAS101952      ELG--GRGVSYCAVCDGA--FFKGK-KLFVIGGGDSAVEEGTFLTKFADSVTIVHRRDE-
STALH01969      ELG--GRGVSYCAVCDGA--FFKNK-NLFVIGGGDSAVEEGTFLTKFADKVTIVHRRDE-
```

```
STAEQ00422      ELG--GRGVSYCAVCDGA--FFKNK-RLFVIGGGDSAVEEGTFLTKFADKVTIVHRRDE-
STAES00543      ELG--GRGVSYCAVCDGA--FFKNK-RLFVIGGGDSAVEEGTFLTKFADKVTIVHRRDE-
STAAB00714      ELG--GRGVSYCAVCDGA--FFKNK-RLFVIGGGDSAVEEGTFLTKFADKITIVHRRDE-
STAA500765      ELG--GRGVSYCAVCDGA--FFKNK-RLFVIGGGDSAVEEGTFLTKFADKVTIVHRRDE-
STAAM00757      ELG--GRGVSYCAVCDGA--FFKNK-RLFVIGGGDSAVEEGTFLTKFADKVTIVHRRDE-
STAAW00726      ELG--GRGVSYCAVCDGA--FFKNK-RLFVIGGGDSAVEEGTFLTKFADKVTIVHRRDE-
STAAS00733      ELG--GRGVSYCAVCDGA--FFKNK-RLFVIGGGDSAVEEGTFLTKFADKVTIVHRRDE-
STAAN00727      ELG--GRGVSYCAVCDGA--FFKNK-RLFVIGGGDSAVEEGTFLTKFADKVTIVHRRDE-
STAAC00807      ELG--GRGVSYCAVCDGA--FFKNK-RLFVIGGGDSAVEEGTFLTKFADKVTIVHRRDE-
STAA300727      ELG--GRGVSYCAVCDGA--FFKNK-RLFVIGGGDSAVEEGTFLTKFADKVTIVHRRDE-
STAA800734      ELG--GRGVSYCAVCDGA--FFKNK-RLFVIGGGDSAVEEGTFLTKFADKVTIVHRRDE-
STAA100752      ELG--GRGVSYCAVCDGA--FFKNK-RLFVIGGGDSAVEEGTFLTKFADKVTIVHRRDE-
STAA200774      ELG--GRGVSYCAVCDGA--FFKNK-RLFVIGGGDSAVEEGTFLTKFADKVTIVHRRDE-
STAA900759      ELG--GRGVSYCAVCDGA--FFKNK-RLFVIGGGDSAVEEGTFLTKFADKVTIVHRRDE-
STAAE00716      ELG--GRGVSYCAVCDGA--FFKNK-RLFVIGGGDSAVEEGTFLTKFADKVTIVHRRDE-
STAAT00767      ELG--GRGVSYCAVCDGA--FFKNK-RLFVIGGGDSAVEEGTFLTKFADKVTIVHRRDE-
STAAD00695      ELG--GRGVSYCAVCDGA--FFKNK-RLFVIGGGDSAVEEGTFLTKFADKVTIVHRRDE-
STAA000815      ELG--GRGVSYCAVCDGA--FFKNK-RLFVIGGGDSAVEEGTFLTKFADKVTIVHRRDE-
STAAH02407      ELG--GRGVSYCAVCDGA--FFKNK-RLFVIGGGDSAVEEGTFLTKFADKVTIVHRRDE-
STAAF00763      ELG--GRGVSYCAVCDGA--FFKNK-RLFVIGGGDSAVEEGTFLTKFADKVTIVHRRDE-
STAAK00744      ELG--GRGVSYCAVCDGA--FFKNK-RLFVIGGGDSAVEEGTFLTKFADKVTIVHRRDE-
STAAJ00703      ELG--GRGVSYCAVCDGA--FFKNK-RLFVIGGGDSAVEEGTFLTKFADKVTIVHRRDE-
STAAG00690      ELG--GRGVSYCAVCDGA--FFKNK-RLFVIGGGDSAVEEGTFLTKFADKVTIVHRRDE-
STAA400732      ELG--GRGVSYCAVCDGA--FFKNK-RLFVIGGGDSAVEEGTFLTKFADKVTIVHRRDE-
STAAR00788      ELG--GRGVSYCAVCDGA--FFKNK-RLFVIGGGDSAVEEGTFLTKFADKVTIVHRRDE-
LISSS02378      ELS--GRGVSYCAVCDGA--FFKER-ELIVVGGGDSAVEEGTYLTRYADKVTIIHRRDK-
LISIN02590      ELS--GRGVSYCAVCDGA--FFKNR-ELVVVGGGDSAVEEGTYLTRYADKVTIVHRRDK-
LISW602421      ELS--GRGVSYCAVCDGA--FFKNR-ELVVVGGGDSAVEEGTYLTRYADKVTIVHRRDK-
EXISA00870      ELG--GRGVSYCAVCDGA--FFKGK-DLFVIGGGDSAVEEGVFLTRFANKVTIVHRRDE-
EXIS202358      ELG--GRGVSYCAVCDGA--FFKEK-ELFVIGGGDSAVEEGVYLTRFAKKVTIVHRREE-
EXIAB02180      ELG--GRGVSYCAVCDGA--FFKEK-ELFVIGGGDSAVEEGVYLTRFAKKVTIVHRREE-
OCEIH02462      ALS--GRGVSYCAVCDGA--FFKNR-NLVVIGGGDSAVEEGIYLTRFANKVTIVHRRDN-
BACIE01058      ELG--GRGVSYCAVCDGA--FFKEK-ELVVVGGGDSAVEEAVYLTRFASKVTIVHRRDQ-
BACCJ03468      ELS--GRGVSYCAVCDGA--FFKTK-ELVVVGGGDSAVEEAAYLTRFASKVTIVHRRDE-
BACHD03507      ELS--GRGVSYCAVCDGA--FFKGK-ELVVVGGGDSAVEEAVYLTRFASKVTIIHRRDQ-
BACPE03382      ELS--GRGVSYCAVCDGA--FFKGK-ELVVVGGGDSAVEEAVYLTRFASKVTVIHRRDQ-
SOLSS00743      ELG--GRGVSYCAVCDGA--FFKQK-KLIVIGGGDSAVEEGIYLTRFADKVTIVHRRDK-
BACC600715      ELG--GRGVSYCAVCDGA--FFKGK-DLVVVGGGDSAVEEGNYLTRFANKVTIVHRRDQ-
ANOFW02486      ELG--GRGVSYCAVCDGA--FFKGK-ELVVVGGGDSAVEEGVYLTRFASKVTIVHRRDQ-
GEOKA03042      ELG--GRGVSYCAVCDGA--FFKGK-DLVVVGGGDSAVEEGVYLTRFANKVTIVHRRDK-
GEOSY02976      ELG--GRGVSYCAVCDGA--FFKGK-DLVVVGGGDSAVEEGVYLTRFANKVTIVHRRDK-
GEOTN02952      ELG--GRGVSYCAVCDGA--FFKGK-DLVVVGGGDSAVEEGVYLTRFANKVTIVHRRDQ-
GEOSW02588      EFS--GRGVSYCAVCDGA--FFKGK-NLVVVGGGDSAVEEGIYLTRFANKVTIVHRRDQ-
GEOS000381      EYG--GRGVSYCAVCDGA--FFKGK-DLVVVGGGDSAVEEGVYLTRFANKVTIVHRRDQ-
GEOTC00378      EYG--GRGVSYCAVCDGA--FFKGK-DLVVVGGGDSAVEEGVYLTRFANKVTIVHRRDQ-
BACMD04906      ELG--GRGVSYCAVCDGA--FFKGK-ELVVVGGGDSAVEEGVYLTRFATKVTIIHRRDE-
BACMQ04908      ELG--GRGVSYCAVCDGA--FFKGK-ELVVVGGGDSAVEEGVYLTRFATKVTIIHRRDE-
BACWK04830      ELG--GRGVSYCAVCDGA--FFKGK-ELVVIGGGDSAVEEGVFLTRFASKVTIVHRRDT-
BACAN04889      ELG--GRGVSYCAVCDGA--FFKGK-ELVVIGGGDSAVEEGVFLTRFASKVTIVHRRDT-
BACC105210      ELG--GRGVSYCAVCDGA--FFKGK-ELVVIGGGDSAVEEGVFLTRFASKVTIVHRRDT-
BACC705051      ELG--GRGVSYCAVCDGA--FFKGK-ELVVIGGGDSAVEEGVFLTRFASKVTIVHRRDT-
BACC005112      ELG--GRGVSYCAVCDGA--FFKGK-ELVVIGGGDSAVEEGVFLTRFASKVTIVHRRDT-
BACC305043      ELG--GRGVSYCAVCDGA--FFKGK-ELVVIGGGDSAVEEGVFLTRFASKVTIVHRRDT-
BACAC05232      ELG--GRGVSYCAVCDGA--FFKGK-ELVVIGGGDSAVEEGVFLTRFASKVTIVHRRDT-
BACAA04720      ELG--GRGVSYCAVCDGA--FFKGK-ELVVIGGGDSAVEEGVFLTRFASKVTIVHRRDT-
BACT005085      ELG--GRGVSYCAVCDGA--FFKGK-ELVVIGGGDSAVEEGVFLTRFASKVTIVHRRDT-
BACC205124      ELG--GRGVSYCAVCDGA--FFKEK-ELIVIGGGDSAVEEGVFLTRFASKVTIVHRRDT-
BACC405064      ELG--GRGVSYCAVCDGA--FFKGK-ELIVIGGGDSAVEEGVFLTRFASKVTIVHRRDT-
BACT104712      ELG--GRGVSYCAVCDGA--FFKGK-ELIVIGGGDSAVEEGVFLTRFASKVTIVHRRDT-
BACLD03769      ELG--GRGVSYCAVCDGA--FFKNK-ELVVVGGGDSAVEEGVYLTRFASKVTIVHRRDK-
BACPZ03376      ELG--GRGVSYCAVCDGA--FFKGK-ELVVVGGGDSAVEEGVYLTRFASKVTIVHRRDK-
BACSU03600      ELG--GRGVSYCAVCDGA--FFKGK-ELVVVGGGDSAVEEGVYLTRFASKVTIVHRRDK-
BACST01653      ELG--GRGVSYCAVCDGA--FFKGK-ELVVVGGGDSAVEEGVYLTRFASKVTIVHRRDK-
BACPT03541      ELG--GRGVSYCAVCDGA--FFKGK-ELVVVGGGDSAVEEGVYLTRFASKVTIVHRRDK-
LEUGG00661      AYQ--GRGVSYCAVCDGA--FFRDE-DVVIVGGGDSAIEEGLYLTNIAKSVTVLHRRDS-
```

LEUGJ00629	AYQ--GRGVSYCAVCDGA--FFRDE-DVVIVGGGDSAIEEGLYLTNIAKSVTVLHRRDS-
LEUCJ00485	DYQ--GRGVSYCAVCDGA--FFRDE-DVLVIGGGDSAIEEGLYLTNLAKSVTVLHRRDK-
LACAR00657	EYS--GRGVSYCAVCDAA--FFKDE-DVVVIGGGDSAIEEGIYLSQLAKSVTVVHRRDK-
LACA300647	EYS--GRGVSYCAVCDAA--FFKDE-DVVVIGGGDSAIEEGIYLSQLAKSVTVVHRRDK-
LACAL00643	EYS--GRGVSYCAVCDAA--FFKDE-DVVVIGGGDSAIEEGIYLSQLAKSVTVVHRRDK-
LACKZ00964	EYS--GKGVSYCAVCDAA--FFKDE-DVTVIGGGDSAIEEGIYLAQLAKSVTVVHRRDE-
LACRJ00357	EFG--GRGVSYCAVCDGA--FFKGK-HLIVVGGGDAAVEEGVYLTQLASKVTVLVRRDE-
LACRD00361	EFG--GRGVSYCAVCDGA--FFKGK-HLIVVGGGDAAVEEGVYLTQLASKVTVLVRRDE-
LACRS01501	EFG--GRGVSYCAVCDGA--FFKGK-HLIVVGGGDAAVEEGVYLTQLASKVTVLVRRDE-
LACSM00455	EYG--GRGVSYCAVCDGA--FFKGD-NVIVVGGGDSAIQEATYLANIAAKVTVVHRRDQ-
LACRG00878	AFS--GRGVSYCAVCDGA--FFKDR-ELAVIGGGDSAIEEGLYLTQMAKKVTVIHRRDQ-
LACRL00939	AFS--GRGVSYCAVCDGA--FFKDR-ELAVIGGGDSAIEEGLYLTQMAKKVTVIHRRDQ-
LACC300844	AYS--GRGVSYCAVCDGA--FFKDR-ELAVIGGGDSAIEEGLYLTQMAKKVTVIHRRDQ-
LACCZ00824	AYS--GRGVSYCAVCDGA--FFKDR-ELAVIGGGDSAIEEGLYLTQMAKKVTVIHRRDQ-
LACCB00988	AYS--GRGVSYCAVCDGA--FFKDR-ELAVIGGGDSAIEEGLYLTQMAKKVTVIHRRDQ-
LACCD01016	AYS--GRGVSYCAVCDGA--FFKDR-ELAVIGGGDSAIEEGLYLTQMAKKVTVIHRRDQ-
LACCC01014	AYS--GRGVSYCAVCDGA--FFKDR-ELAVIGGGDSAIEEGLYLTQMAKKVTVIHRRDQ-
LACBN01277	EYG--GKGVSYCAVCDGA--FFKNK-EVVVVGGGDSAIEEASYLAGIVDHVTVIHRRDQ-
LACBA00605	KFG--GRGVSYCAVCDGA--FFKNR-EVVVIGGGDSAVEEGLYLAGLASKVTIIHRRDQ-
LACPL00650	TYG--GRGVSYCAVCDGA--FFRNK-HVVVVGGGDSAIEEGTYLTQLADKVTVIHRRDQ-
LACPJ00628	TYG--GRGVSYCAVCDGA--FFRNK-HVVVVGGGDSAIEEGTYLTQLADKVTVIHRRDQ-
LACPS00585	TYG--GRGVSYCAVCDGA--FFRNK-HVVVVGGGDSAIEEGTYLTQLADKVTVIHRRDQ-
PEDCP00497	DFS--GRGVSYCAVCDGA--FFKGM-HLVVIGGGDSAIEEGIYLTQLASKVTVIHRRDQ-
CARS100359	EYN--GRGVSYCAVCDGA--FFRNK-ELVVVGGGDSAVEEGTYLTQFAKKVTIIHRRDE-
AERUA00263	EYN--GHGVSYCAVCDGA--FYKGR-DIKVVGGGDSAVEEGSYLTQFANTVDIIHRRDQ-
ELUMP00590	KYK--GRGISSCATCDGM--FFKNK-DVVVIGGGNTAFEDVLYLSKICNKVYLVHRREG-
SPHPG02940	TYQ--GKGVSYCGTCDGP--FFKKK-KILVVGGGDTALTDALYLSKLSDDVVIVHRKDR-
SPHGB01868	KYN--GKGVSYCGTCDGP--FFKGK-RILVVGGGDTALTDGIFLSKLSDHITIVHRKDR-
TREPZ00273	EFN--GRGVSYCASCDGP--FFKGK-RILVVGGGDAACDEARFLAFLSDRVLLVHRRDK-
TREAZ03414	QFY--GRGVSYCATCDGP--FFKNK-KIFVVGGGDAACDEAQYLSRLSSQVILIHRRDR-
SPITD00734	EYT--GRGVSYCATCDGP--FFKGK-RMLVVGGGDAACDEAMYLSKLTDKIVHIHRRDR-
SPITZ01373	EYT--GRGVSYCATCDGP--FFKGK-RMLVVGGGDAACDEAMYLSKLTDKIVHIHRRDR-
TREPA00803	EFL--GRGVSYCATCDGP--FFRNK-HVVVIGGGDAACDESLVLSRLTDRVTMIHRRDT-
TREPS00802	EFL--GRGVSYCATCDGP--FFRNK-HVVVIGGGDAACDESLVLSRLTDRVTMIHRRDT-
TREPC00747	EFL--GRGVSYCATCDGP--FFRNK-HVVVIGGGDAACDESLVLSRLTDRVTMIHRRDT-
TREPM00823	EFL--GRGVSYCATCDGP--FFRNK-HVVVIGGGDAACDESLVLSRLTDRVTMIHRRDT-
TREPD00824	EFL--GRGVSYCATCDGP--FFRNK-HVVVIGGGDAACDESLVLSRLTDRVTMIHRRDT-
TREPU00781	EFL--GRGVSYCATCDGP--FFRNK-HVVVIGGGDAACDESLVLSRLTDRVTMIHRRDT-
ENCCU00216	EFW--QRGVSSCAVCDGF--AYTNK-ITCVIGGGDAAMEEALYLSGIAKKVYIIHRRNE-
HELM100964	ELW--GKGVSTCATCDGF--FYKNK-EVAVLGGGDTALEEAIYLTKMCKKVYLIHRRDG-
HELCP01490	EYW--GKGISTCATCDGF--FYKDQ-EVVVLGGGDTALEEAIYLSRICSKVHLIHRRNE-
ARCFU01526	AFI--GRGVSYCATCDGN--FFRGK-KVIVYGSGKEAIEDAIYLHDIGCEVTIVSRTPS-
FERPA02445	EFV--GKGVSYCATCDGN--FFRGK-RVAVVGGGNTAVTDAIYLHEIGCDVVLIHRRDE-
ARCVS01910	EFV--GRGVSYCATCDGH--FFRGK-RVLVIGGGNTAVTDAVYLKEIGCDVTLVHRRDA-
METEZ00677	TYY--GRGVSYCATCDAP--FYKER-DVIVVGGGNTAISDALILSNVANKVYQVHRRDE-
METHD00869	EFM--GKGVSYCATCDAP--FYKGK-TVMVIGGGESALTDALILSNIVKKVYIVHRRDK-
METMA02304	ELI--SKGVSYCAICDGP--FFRNK-IVAVVGGGNSAVTDALFLSKVAQKVYLVHRRDH-
METAC01311	EFI--SKGVSYCAICDGP--FFKNK-TVVVVGGGNSAVTDALLLSKVAQNVYLIHRRDR-
KOSOT00298	EFA--GKGVSYCATCDGH--FFAGK-HIAVIGGGNSALDEALFLSKIVDKITIVQNLPK-
MARPK01613	EFA--GRGVSYCATCDGH--FFKNQ-KVAVIGGGNTAVEEALYLSKIAKEVYIIHRRDK-
SLAHD02439	QFV--GKGISFCATCDAN--FFTDL-EVYVVGGGDSAVEEAMYLTGFARKVTIIHRRDE-
FILAD00976	EFV--GRGISYCATCDAA--FFSGL-PVYVIGGGDSAIEEAIYLTKFAREVTVIHRREG-
BUTPB02463	EFI--GKGVSYCATCDGA--FYRGK-VTAVNGGGDVAVEDAIFLSRFCSKVYLIHRRDE-
CLOPH00250	ELQ--GMGVSYCATCDGA--FFRGK-TVAVVGGGDVAVEDAIFLARICKEVHVIHRRDE-
CLOSW00592	ELT--GMGVSYCATCDGA--FFRNK-VAAVVGGGDVAIEDAIFLARMCKKVYLIHRRNK-
LACFC00207	EYS--GRGVSYCAVCDGA--FFRNK-HLIVVGGGDSAVEEGMYLTQFADKVTVLVRHDH-
CRYCD00853	ELA--GHGVSYCATCDGN--FFRGQ-DVVVVGGGNTAAADAVYLSRICNTVHLVHRRGE-
EGGLE01847	DLQ--GRGVSYCATCDGN--FFRDK-EVMVVGGGNTAAGDAIYLSRICKKVYLVHRRDK-
PYRFU01410	EFT--GRGVSYCATCDGP--LFVGK-EVIVVGGGNTALQEALYLHSIGVKVTLVHRRDK-
PYRHO01476	EFT--GRGVSYCATCDGP--LFVGK-EVIVVGGGNTALQEALYLHSIGVKVTLVHRRDK-
PYRAB00730	EFT--GRGVSYCATCDGP--LFVGK-EVIVVGGGNTALQEALYLHSIGVKVTLVHRRDK-
PYRSN00014	EFT--GRGVSYCATCDGP--LFVGK-EVIVVGGGNTALQEALYLHSIGVKVTLVHRRDK-
THEGJ00181	ELT--GKGVSYCATCDGP--LFKGK-KVIVVGGGNTALQEALYLKSIGVDVTLVHRRQK-
THEKO02097	ELT--GKGVSYCATCDGP--LFKGK-KVIVVGGGNTALQEALYLKSIGVDVTLVHRRQQ-
THEON01610	EFT--GRGVSYCATCDGP--LFKGK-KVIVVGGGNTALQEALYLKSIGVDVTLVHRRDQ-

```
THES401476      EFT--GRGVSYCATCDGP--LFKGK-KVVVVGGGNTALQEALYLKSIGVDVTLVHRREE-
SYNWW02368      EYL--GRGVSYCATCDGA--FFQGT-TVAVVGGGDSAVKEALYLTGIASKVYLIHRREG-
UNCTG00012      EFM--GRGVSFCAVCDAP--FYRDK-NVLVVGGGDSAIEEAAYISKFAKNVTVVHRRDK-
THEA101460      EFL--GRGVSYCAVCDGA--FFKDR-TVAVVGGGDSALEEALYLTKFANKVYLIHRRDK-
DENA201647      KFY--GRGVSFCATCDGS--FYKDK-EVAVVGGGESALEEGMYLTKFANKVTVIHRRDQ-
DEFDS00488      KFL--GRGISFCATCDGA--FYKDK-VVAVIGGGDSAVEEGHYLTKFAKKVYIVHRRDK-
CALNY01292      KFL--GRGISFCATCDGA--FYRGK-TVAVIGGGDSAVEEAHYLTRFAEKVYIVHRRDK-
SYNGF01652      KFH--GRGVSYCATCDGA--FYKEK-QVAVVGGGNAALEEGVYLTKFASKVTIIHRRDE-
DESB201123      RLT--GKGVSYCGTCDGP--FYRDQ-TVVCFGGGDTAAEEAIFLTRFARKVYLAHRRDQ-
DEIPM00845      KFW--GKGVSTCATCDGF--FYRGK-KVVVVGGGDAAVEEGLFLTKFADEVTLIHRRDT-
DEIRA01924      NFW--GKGVSTCATCDGF--FYKGK-KVVVIGGGDAAVEEGMFLTKFADEVTVIHRRDT-
DEIML01437      NFW--GRGVSTCATCDGF--FYRGK-KVVVIGGGDAAVEEGLFLTKFADEVTLIHRRDT-
DEIGD02532      HFW--GKGVSTCATCDGF--FYRGK-KVVVVGGGDAAVEEGLFLTKFADEVTLIHRRDT-
DEIDV00622      LFW--GKGVSTCATCDGF--FYRGK-KVVVVGGGDAAVEEGLFLTKFAEEVTLIHRRDT-
TRURR01079      TFY--GRGVSTCATCDGF--FYRGK-HVVVVGGGDAAVEEGMFLTKFAERVSVVHRRDE-
THETG01863      KFY--GRGVSTCATCDGF--FYRDK-EVVVVGGGDAAVEEGIFLTKFARKVTLVHRRDE-
THET201543      KFY--GRGVSTCATCDGF--FYRDK-EVVVVGGGDAAVEEGIFLTKFARKVTLVHRRDE-
THET801911      KFY--GRGVSTCATCDGF--FYRDK-EVVVVGGGDAAVEEGIFLTKFARKVTLVHRRDE-
OCEP502115      KFY--GRGVSTCATCDGF--FYRDK-DVVVVGGGDAAVEEGIFLTKFARKVTIIHRRDE-
DEHLB00585      ELV--GRGVSFCATCDGP--FFRNK-IVAVIGGGNGALNEAVHLTHFAERVFIIHRRDS-
DEHMG00439      EFS--GRGVSYCATCDAP--FYNDK-VVAVVGGGNMAFYEALHLSEFTKKVYLIHRRQG-
DEHMB00497      EFS--GRGVSYCATCDAP--FYNDK-VVAVVGGGNMAFYEALHLSEFAKKVYLIHRRQG-
DEHMC00427      EFS--GRGVSYCATCDAP--FYNDK-VVAVVGGGNMAFYEALHLSEFAKKVYLIHRRQG-
THELD00726      EFT--GRGVSYCAVCDGA--FFEEQ-EVAVIGGGNTAVEEALYLTQFASKVYIIHRRDS-
ANAMD00624      EFT--GRGVSYCAVCDGA--FFEDQ-VVAVIGGGNTAVEEADYLTRFASKVYIVHRRDS-
THEAS00815      EYT--GRGVSYCAVCDGA--FFEGE-EVAVIGGGNTAVEEACYLTQFASKVYIVHRRDS-
AMICL00707      EHI--GGGVSYCAVCDGA--FFEDQ-VIAVVGGGNTAVEEACYLTNFASKVYIIHRRDA-
CLOCE01684      KFR--GSGVSYCAVCDGA--FFRGK-TVAVVGGGDTAAEDALYLARFCPKVYIIHRRDT-
HYDS000616      EFL--NRGVSYCATCDGA--LFEDV-PIAVVGGGDSATQEAIFLTRFASKVYLIHRRDK-
HYDTT00224      KFL--NRGVSYCATCDGA--LFDGL-PIAVIGGGDSATQEALFLTRFGSIVYLIHRRDQ-
PELTS01405      QYT--GRGVSYCATCDGA--FFRGK-KVAVIGGGDAAVQEAIFLTKFAEKVFIIHRRNE-
DESRL02179      KFH--GRGVSYCATCDGA--FFRDK-TVAVVGGGDSAVEEAIFLTKFAAKVYIVHRRGQ-
BORBP00506      LFW--NKGISVCAICDGH--LFKGK-RVAVIGGGNTALSESIYLSKLVDKVYIIVRKDY-
BORAP00518      LFW--NKGISVCAICDGH--LYKGK-RVAVIGGGNTALSEAIYLSKLADKVYVLVRKDY-
BORBU00514      LFW--NKGISVCAICDGH--LFKGK-RVAVIGGGNTALSESIYLSKLVDKVYLIVRKNN-
BORBZ00490      LFW--NKGISVCAICDGH--LFKGK-RVAVIGGGNTALSESIYLSKLVDKVYLIVRKDN-
BORBN00493      LFW--NKGISVCAICDGH--LFKGK-RVAVIGGGNTALSESIYLSKLVDKVYLIVRKDN-
BORRA00486      LFW--NRGISVCAICDGH--LFKGK-TVAVIGGGNTAISEAIYLSKLAEKVYVIVRKNY-
BORDL00498      LFW--NRGISVCAICDGH--LFKGK-TLAVIGGGNTAISEAIYLSKLAEKVYVIVRKNY-
BORHD00497      LFW--NKGISVCAICDGH--LFKGK-TAAVIGGGNTAISEAIYLSKLLKNVYVIVRRDC-
BORT900497      LFW--NRGISVCAICDGH--LFKGK-TVAVIGGGNTAISEAIYLSKLSEKVYVIVRKDY-
METKA01560      ELE--GRGVSYCAICDGP--AFQNR-IVAIVGSGTHAANTALFLSEIAERVYVITPDGK-
MYCA500361      KFN--HRGVSYCVLCDGV--IFKNQ-PCAIIGGGNSAFEESISLASLASEVHIFVR-DG-
METVS00145      DYL--GKGVCYCVMCDAF--FFINK-EVIVLGRGTSAIMAAYNLKDIVKKITIVTDRPN-
METOI01431      KFI--GRGVSYCTTCDAF--FYLNK-EVIVIGRGTPAVMSALNLKDIAKKVTIITDKPE-
MYCHN00051      EFY--GKGISYCAICDGN--LYRER-PVIVVGGGNSAVEESIYLSDITSKVKLVHRRRE-
MYCSL00465      EYY--SKGVSYCAICEGN--LYTGE-EVIVVGGGNSALEESIYLTAMASNLKLVHRRRE-
MYCS300420      EYY--SKGVSYCAICEGN--LYTGE-EVIVVGGGNSALEESIYLTAMASNLKLVHRRRE-
DESK101258      ELT--GKGVSYCATCDGP--LFKDK-IVAVVGGGNAAFTSALYLAKIASHVYLIHRRSE-
STAHD01472      KLA--GRGISYCATCDGP--LFKGK-VVAVVGGGNSAFSSALYLASLASKVYIIHRREQ-
THEC100270      EFS--GRGVSYCATCDGP--LFKDK-VVAVVGGGNSALTSAIYLASLASKVYIIHRRDE-
NANEQ00478      L-----PGVSYCAECDAP--LFKGK-TVAVVGGGNTAFHDALVLSNYANKVYLIHRRDQ-
CALLD01225      TFT--GKGVSYCSICDAP--LYKGKDSVVVVGGGDAALEGALLLSGYVKKVYLVHRRDQ-
SULSO02155      EFA--GRGISYCSVCDAP--LFKNR-VVAVIGGGDSALEGAEILSSYSTKVYLIHRRDT-
SULS900210      EFA--GRGISYCSVCDAP--LFKNR-VVAVIGGGDSALEGAEILSSYSTKVYLIHRRDT-
SULIA00208      EFV--GKGISYCSVCDAP--LFKNR-VVAVVGGGDSALEGAEILSSYSTKVYLIHRRDS-
SULIM00207      EFV--GKGISYCSVCDAP--LFKNR-VVAVVGGGDSALEGAEILSSYSTKVYLIHRRDS-
SULIK00225      EFV--GKGISYCSVCDAP--LFKNR-VVAVVGGGDSALEGAEILSSYSTKVYLIHRRDS-
IGNH400907      EYF--GKGVSYCVVCDAP--FFKGE-PMALVGYDDHAMEEAVYMTSLASKVYIVTHGKK-
KORCO01040      EFF--GKGVSYCAVCDGP--LFKGK-RVIVVGGGNTAAISSIYLSKIAKEVILVHRRSS-
THESM01133      KFY--GRGVSYCATCDGP--LFRGK-HVIVVGGGNTALQEALYLNEIGVNVTLVHRREE-
THEBM01534      ELT--GRGVSYCATCDGP--LFVGK-EVIVVGGGNTALQEALYLHSIGVKVTLVHRRDK-
METST01363      EFV--GRGVSYCAVCDGT--FFVKK-EVLVIGGGNSAVTEALYLNRIGVKCSLVHRRDK-
METTH00703      DLL--GRGVCYCATCDGP--LYKGR-KVLMVGGGNSAAQEAVFLKNIGCDVSIVHRRDE-
METSL02406      EYV--GRGISYCSICDGM--LFKGR-DVVVVGGGNSAAEHALHLNDIGVNVKLIHRRGE-
```

```
METLA02406      EYV--GRGISYCSICDGM--LFKGR-DVVVVGGGNSAAEHALHLNDIGVNVKLIHRRGE-
METPW00194      EFI--GKGISYCSICDGM--FFRGK-EVLVVGGGNSAAEHALHLNDIGCKVKMVHRRDE-
RUBXD00226      RLM--GRGVSGCATCDGF--FFKDK-RVAVVGGGDTAMEEALFLSRYASEVVIIHRRDE-
RHOM400178      RLI--GRGVSACATCDGA--FFRGM-EVAVVGGGDTAMEEALFLTRFATRVYVIHRRDQ-
GARV400051      EYS--GRGVSYCATCDGF--FFRGK-PITVVGGGDSAFEEALFLTRFGSSVTLIHRRDE-
BIFAP01644      EFS--GRGVSYCATCDGF--FFKNK-PIVVVGGGDSAMSDADFLTRFGSSVTLIHRRQG-
SEGRD00014      ELL--GRGVSSCATCDGF--FFKDQ-DIAVIGGGDSAMEEATFLTRFAKSVTLIHRRDE-
GORB404535      SLL--GRGVSACATCDGF--FFKDQ-DIAVIGGGDSAMEEATFLTKFARSVTLVHRRDE-
GORPV04920      ELL--GRGVSACATCDGF--FFKDQ-DIAVIGGGDSAMEEATFLTKFARSVTLIHRREE-
MYCA904903      TLL--GRGVSSCATCDGF--FFKDQ-DIAVIGGGDSAMEEATFLTRFARSVTLIHRRDE-
MYCSS05356      ALL--GMGVSTCATCDGF--FFRDQ-DIAVIGGGDSAMEEATFLTRFARSVTVIHRREE-
MYCSJ05684      ALL--GMGVSTCATCDGF--FFRDQ-DIAVIGGGDSAMEEATFLTRFARSVTVIHRREE-
MYCSK05387      ALL--GMGVSTCATCDGF--FFRDQ-DIAVIGGGDSAMEEATFLTRFARSVTVIHRREE-
MYCS206581      ALT--GMGVSTCATCDGF--FFRDQ-DIVVVGGGDSAMEEATFLTRFARSVTLIHRRDE-
MYCCN05158      TLL--GMGVSTCATCDGF--FFRDQ-DIAVVGGGDSAMEEATFLTRFARSVTLIHRREE-
MYCVP05890      TLL--GMGVSTCATCDGF--FFRDQ-DIAVVGGGDSAMEEATFLTRFARSVTLIHRRDE-
MYCGI00775      EML--GMGVSTCATCDGF--FFRDQ-DIVVVGGGDSAMEEATFLTRFARSVTLIHRRDE-
MYCSR04994      EML--GMGVSTCATCDGF--FFRDQ-DIVVVGGGDSAMEEATFLTRFARSVTLIHRRDE-
AMYSD04545      RLL--GRGVSSCATCDGF--FFRDQ-DIAIIGGGDSAMEEATFLTRFARSVTIVHRREE-
MYCLE02681      ELL--GRGVSACATCDGS--FFRGQ-DIAVIGGGDSAMEEALFLTRFARSVTLVHRRDE-
MYCLB02681      ELL--GRGVSACATCDGS--FFRGQ-DIAVIGGGDSAMEEALFLTRFARSVTLVHRRDE-
MYCSD04323      ELL--GRGVSSCATCDGF--FFRDQ-DIAVIGGGDSAMEEATFLTRFARSVTLVHRREE-
MYCPA04306      DLL--GRGVSSCATCDGF--FFKDQ-DIAVIGGGDSAMEEATFLTRFARSVTLVHRREE-
MYCA105023      DLL--GRGVSSCATCDGF--FFKDQ-DIAVIGGGDSAMEEATFLTRFARSVTLLHRREE-
MYCUA04107      ELL--GRGVSSCATCDGF--FFRDQ-DIAVIGGGDSAMEEATFLTRFARSVTLVHRRDE-
MYCMM05378      ELL--GRGVSSCATCDGF--FFRDQ-DIAVIGGGDSAMEEATFLTRFARSVTLVHRRDE-
MYCA003905      ELL--GRGVSSCATCDGF--FFRDQ-DIAVIGGGDSAMEEATFLTRFARSVTLVHRRDE-
MYCTU03943      ELL--GRGVSSCATCDGF--FFRDQ-DIAVIGGGDSAMEEATFLTRFARSVTLVHRRDE-
MYCTF03846      ELL--GRGVSSCATCDGF--FFRDQ-DIAVIGGGDSAMEEATFLTRFARSVTLVHRRDE-
MYCTA03980      ELL--GRGVSSCATCDGF--FFRDQ-DIAVIGGGDSAMEEATFLTRFARSVTLVHRRDE-
MYCTK04010      ELL--GRGVSSCATCDGF--FFRDQ-DIAVIGGGDSAMEEATFLTRFARSVTLVHRRDE-
MYCTC03612      ELL--GRGVSSCATCDGF--FFRDQ-DIAVIGGGDSAMEEATFLTRFARSVTLVHRRDE-
MYCTD03548      ELL--GRGVSSCATCDGF--FFRDQ-DIAVIGGGDSAMEEATFLTRFARSVTLVHRRDE-
MYCCP03898      ELL--GRGVSSCATCDGF--FFRDQ-DIAVIGGGDSAMEEATFLTRFARSVTLVHRRDE-
MYCBO02863      ELL--GRGVSSCATCDGF--FFRDQ-DIAVIGGGDSAMEEATFLTRFARSVTLVHRRDE-
MYCBP03913      ELL--GRGVSSCATCDGF--FFRDQ-DIAVIGGGDSAMEEATFLTRFARSVTLVHRRDE-
MYCBT03913      ELL--GRGVSSCATCDGF--FFRDQ-DIAVIGGGDSAMEEATFLTRFARSVTLVHRRDE-
TSUPD04078      ALL--GRGVSACATCDGF--FFRDQ-DIAVVGGGDSAMEEALFLTKFAKSVTLIHRSEN-
ACTMD06897      ELL--GRGVSACATCDGF--FFRDQ-DIAVLGGGDSAMEEATFLTRFARSVTIIHRRED-
SACES08402      ELL--GRGVSACATCDGF--FFRDQ-DIAVVGGGDSAMEEATFLTRFARSVTIIHRREE-
SACVD03814      KLF--GRGVSSCATCDGF--FFRDQ-DIAVVGGGDSAMEEATFLTKFARSVTIIHRREE-
AMYMU09190      ELL--GRGVSACATCDGF--FFRDH-DIVVAGGGDSAMEEATFLTKFAKSVTIVHRRDE-
AMYMS10178      ELL--GRGVSACATCDGF--FFRDH-DIVVAGGGDSAMEEATFLTKFAKSVTIVHRRDE-
PSEUX06421      ELL--GRGVSACATCDGF--FFREQ-DIAVIGGGDSAMEEATFLTRFARSVTIVHRREE-
NOCFA05681      RLL--GRGVSACATCDGF--FFRGQ-DIVVVGGGDSAMEEATFLTKFASSVTIVHRREE-
NOCCG05476      ELL--GRGVSACATCDGF--FFKGQ-DIVVVGGGDSAMEEATFLTKFASSVTIVHRRDE-
RHOE406010      RLL--GRGVSACATCDGF--FFRDQ-DIVVVGGGDSAMEEATFLTRFARSVTLVHRREE-
RHOOB03468      KLL--GRGVSACATCDGF--FFKDQ-DIVVVGGGDSAMEEATFLTRFARSVTVVHRREE-
RHOE104515      RLL--GRGVSACATCDGF--FFRDQ-DIVVVGGGDSAMEEATFLTKFARSVTLVHRREE-
CORDI02302      TLL--GRGVSACATCDGF--FFRDH-HIAVIGGGDSAMEEADFLTKFGSKVSIVHRRDE-
CORD202219      TLL--GRGVSACATCDGF--FFRDH-HIAVIGGGDSAMEEADFLTKFGSKVSIVHRRDE-
CORDL02208      TLL--GRGVSACATCDGF--FFRDH-HIAVIGGGDSAMEEADFLTKFGSKVSIVHRRDE-
CORDJ02208      TLL--GRGVSACATCDGF--FFRDH-HIAVIGGGDSAMEEADFLTKFGSKVSIVHRRDE-
CORDH02221      TLL--GRGVSACATCDGF--FFRDH-HIAVIGGGDSAMEEADFLTKFGSKVSIVHRRDE-
CORD702316      TLL--GRGVSACATCDGF--FFRDH-HIAVIGGGDSAMEEADFLTKFGSKVSIVHRRDE-
CORD302337      TLL--GRGVSACATCDGF--FFRDH-HIAVIGGGDSAMEEADFLTKFGSKVSIVHRRDE-
CORDD02235      TLL--GRGVSACATCDGF--FFRDH-HIAVIGGGDSAMEEADFLTKFGSKVSIVHRRDE-
CORDV02170      TLL--GRGVSACATCDGF--FFRDH-HIAVIGGGDSAMEEADFLTKFGSKVSIVHRRDE-
CORDW02254      TLL--GRGVSACATCDGF--FFRDH-HIAVIGGGDSAMEEADFLTKFGSKVSIVHRRDE-
CORDK02230      TLL--GRGVSACATCDGF--FFRDH-HIAVIGGGDSAMEEADFLTKFGSKVSIVHRRDE-
COREF02870      ELL--GRGVSACATCDGF--FFRDQ-DIAVIGGGDSAMEEATFLTKFARSVTIVHRREE-
CORGL03082      ELL--GRGVSACATCDGF--FFRDQ-DIAVIGGGDSAMEEATFLTKFARSVTIVHRREE-
CORGK02974      ELL--GRGVSACATCDGF--FFRDQ-DIAVIGGGDSAMEEATFLTKFARSVTIVHRREE-
CORGB03038      ELL--GRGVSACATCDGF--FFRDQ-DIAVIGGGDSAMEEATFLTKFARSVTIVHRREE-
CORK402006      EHL--GLGVSACATCDGF--FFKDK-PIVVVGGGDSAMEEADFLTKFGSSVTIVHRRGE-
```

CORJK02028	EML--GRGVSACATCDGF--FFKDK-QIAVIGGGDSAMEEADFLTKFGETVTIIHRRDE-
CORVD02951	ELL--GHGVSACATCDGF--FFKGK-QIAVIGGGDSAMEEADFLTKFGEKVTLIHRRNE-
ARCHD01706	TFS--GKGVSYCATCDGF--FFKGK-EIAVVGGGDSAVTEAIFLSRFGSTVHVIHRRDE-
THET101205	EFM--GRGVSACATCDGF--FFRDK-KVVVVGGGDVAMEEALFLTRYASSVTVIHRRDQ-
PROAC02247	RLS--GRGVSWCATCDGF--FFTGK-DIAVVGGGDSAVEEATFLTRFANSVTLVHRRDQ-
PROAS02302	RLS--GRGVSWCATCDGF--FFTGK-DIAVVGGGDSAVEEATFLTRFANSVTLVHRRDQ-
CAERE29798	RLS--GHGVSWCATCDGF--FFREQ-EIVVVGGGDSAMEEALFLTRFASKVTVVHRRDE-
CELFA03749	RLS--GRGVSWCATCDGF--FFRDQ-EIIVVGGGDSAVEEATFLTRFGKRVTLVHRRDQ-
JONDD02484	RLS--GKGVSWCATCDGF--FFRDQ-DIIVVGGGDSAMEEATFLTRFARSVTIVHRRDE-
XYLCX03324	RLS--GRGVSWCATCDGF--FFRDQ-HIAVVGGGDSAMEEATFLTRFASKVTVVHRRDA-
ACIC102144	RLL--GRGVSACATCDGF--FFRDQ-DIAVVGGGDSAAEEALFLTRFARSVTLIHRRDA-
FRADG04042	RLL--GRGVSACATCDGF--FFRDQ-DILVVGGGDSALEEATFLTRFGRSVTVIHRRDR-
FRASU07065	KLL--GRGVSACATCDGF--FFRDQ-DIVVVGGGDSAMEEATFLTRFANSVTVVHRRDK-
FRASN07115	RLL--GRGVSACATCDGF--FFRDQ-DIAVVGGGDSALEEATFLTRFAKSVTIVHRRDR-
FRASC04424	RLL--GRGVSACATCDGF--FFRDQ-DIAVVGGGDSAMEEATFLTRFARSVTVIHRRDK-
FRAAA06712	RLL--GRGVSACATCDGF--FFRDH-DIAVVGGGDSAMEEATFLTRFARSVTLIHRRDK-
KYTSD02521	ELS--GHGISWCATCDGA--FFREQ-HIAVVGGGDSAVEEATFLTRFADKVTLVHRRDE-
KINRD04462	RLS--GRGVSWCATCDGF--FFREQ-DIVVVGGGDSAVEEATFLTRFARTVTIVHRRDE-
NOCDD04789	ELS--GRGTSWCATCDGF--FFRDQ-DIAVVGGGDTAMEEALFLTRFARSVTVIHRRDQ-
NOCAA01941	ELS--GRGTSWCATCDGF--FFRDQ-DIAVVGGGDTAMEEALFLTRFAKSVTVIHRRDE-
STRRD08913	RLS--GHGVSWCATCDGF--FFRGQ-DIVVVGGGDTAMEEAIFLTRFAGSVTVVHRRDE-
THECD04863	RLS--GRGVSWCATCDGF--FFRDQ-DIAVVGGGDSAMEEAIFLTKFAKSVTVVHRRDQ-
THEBD03528	RLS--GRGVSWCATCDGF--FFRDQ-DIAVVGGGDSAMEEAIFLTRFAKSVTVIHRRDT-
CATAD08894	RLS--GKGVSWCATCDGF--FFKGQ-DIVVVGGGDTAMEEALFLTRFGQTVTVVHRRDE-
KRIFD06917	QLS--GRGVSWCATCDGF--FFRQH-EIAVVGGGDTAIEEATFLTRFADKVTIVHRRDE-
KITSK03730	KLS--GRGVSWCATCDGF--FFRDQ-DIAVVGGGDTALEEATFLSRFAKSVTVIHRRNS-
STRBB05366	ELS--GRGVSWCATCDGF--FFKDQ-DIAVIGGGDTAMEEATFLSRFAKSVTVVHRRDT-
STRVP03660	TLS--GRGVSWCATCDGF--FFKDQ-DIAVVGGGDTAIEEATFLSRFAKSVTIVHRRDT-
STRSW04381	ALS--GRGVSWCATCDGF--FFKDQ-DIAVIGGGDTAMEEATFLSRFAKSVTVVHRRDT-
STRGG03562	ALS--GRGVSWCATCDGF--FFKDH-DIAVIGGGDTAMEEATFLSRFAKSVTIVHRRDT-
STRFA03098	ALS--GRGVSWCATCDGF--FFKDQ-DIAVIGGGDTAMEEATFLSRFAKSVTIVHRRDS-
STRAW04303	ALS--GRGVSWCATCDGF--FFKGQ-DIAVIGGGDTAMEEATFLSRFAKSVTIVHRRDT-
STRCO02929	ALS--GRGVSWCATCDGF--FFKDQ-DIAVIGGGDTAMEEATFLSRFAKSVTIVHRRDT-
STRHJ05105	TLS--GRGVSWCATCDGF--FFKDQ-DIVVIGGGDTAMEEATFLSRFARSVTIVHRRDT-
MONBE04991	TFW--QRGISACAVCDGAAPIYRNK-PLIVIGGGDSAMEEATFLTKYASKVYIVHRRDE-
CHLRE01313	GFW--NRGISACAVCDGAAPIFRNK-PIAVIGGGDSAMEEATFLTKYGSKVYIIHRRDS-
MEDTR25591	GYW--NRGISACAVCDGAAPIFRNK-PLAVIGGGDSAMEEATFLTKYGSEVYIIHRRDT-
SOLLC13750	GFW--NKGISACAVCDGAAPIFRNK-PLAVIGGGDSAMEEANFLTKYGSKVYIIHRRDE-
PRUPE10733	GFW--NRGISACAVCDGAAPIFRNK-PLAVIGGGDSAMEEANFLTKYGSEVHIIHRRDA-
MANES18605	TFW--NRGVSACAVCDGAAPIFRDK-PLAVIGGGDSAMEEATFLTKYGSKVYIIHRRDT-
THECC00884	GFW--NKGISACAVCDGAAPIFRDK-PLAVIGGGDSAMEESTYLTKYASKVYIIHRRDT-
PHYPA31147	GYW--NRGISACAVCDGAAPIFRNK-PLAVIGGGDSAMEEANFLTKYASKVYLIHRRDT-
AMBTC19471	GFW--NRGISACAVCDGAAPIFRNK-PLVVIGGGDSAMEEATFLTKYGSTVYIIHRRDE-
MUSAC26038	VFW--NRGISACAVCDGAAPIFRNR-PIAVVGGGDSAMEEANFLTKYGSRVYII------
MUSAM33177	VFW--NRGISACAVCDGAAPIFRNR-PIAVVGGGDSAMEEANFLTKYGSRVYII------
SETIT03079	TYW--NRGISACAVCDGAAPIFRNK-PIAVIGGGDSAMEEANFLTKYGSHVYIIHRRNT-
ORYBR12195	AYW--NRGISACAVCDGAAPIFRNK-PIAVIGGGDSAMEESNFLTKYGSHVYIIHRRNA-
COCLU07729	KYW--GNGVSACAVCDGSLPMFRDQ-PLVVIGGGDSAVEEALYLTKKAKKVTVLVRRDK-
PHANO13702	QYW--GFGVSACAVCDGSLPMFRDQ-PLVVIGGGDSAVEEALYLTKKAKKVTVLVRRDK-
PHAND10804	QYW--GFGVSACAVCDGSLPMFRDQ-PLVVIGGGDSAVEEALYLTKKAKKVTVLVRRDK-
AURPU02089	QYW--GNGVSACAVCDGSLPIFRQK-PLVVIGGGDSAVEEAIYLTKKASKVTVLVRKDQ-
ZYMTR07711	KFW--GNGVSACAVCDGSLPIFREK-PLVVIGGGDSAVEESLYLTKKASKVTVLVRKDF-
DICPU05926	KFW--SKGVSACAVCDGALPIYRNK-HLVVVGGGDTAAEEATFLTHFASKVTMLVRRNT-
ENTHI00522	KYW--QNGVSACAICDGAVPIFRNK-VLMVVGGGDAAMEEALHLTKYGSKVIILHRRDA-
LEPBA02231	VFW--QRGISACAVCDGALPIYRNK-ALAVVGGGDSAVEEANHLTKFASKVYLVVRRDQ-
LEPBP02301	VFW--QRGISACAVCDGALPIYRNK-ALAVVGGGDSAVEEANHLTKFASKVYLVVRRDQ-
LEPBL01462	TYW--QRGISACAVCDGALPIYRNK-ELAVVGGGDSAVEEASHLTKFASKVYLVHRRDS-
LEPBJ01276	TYW--QRGISACAVCDGALPIYRNK-ELAVVGGGDSAVEEASHLTKFASKVYLVHRRDS-
LEPIN02475	IYW--QRGISACAVCDGALPIYRNK-ELVVVGGGDSAVEEASHLTKFASKVYLVHRRDS-
LEPII01996	IYW--QRGISACAVCDGALPIYRNK-ELVVVGGGDSAVEEASHLTKFASKVYLVHRRDS-
LEPIC01426	IYW--QRGISACAVCDGALPIYRNK-ELVVVGGGDSAVEEASHLTKFASKVYLVHRRDS-
SPIAZ00697	RLW--QRGISACAVCDGALPLFRNQ-PLVVIGGGDSAAEEASFLTKYGSSVTMLVRRDE-
PENRW10140	TYW--QNGISACAVCDGAVPIFRNK-PLYVIGGGDSAAEEAMFLAKYGSSVTVLVRKDK-
PENCH09104	TYW--QNGISACAVCDGAVPIFRNK-PLYVIGGGDSAAEEAMFLAKYGSSVTVLVRKDK-
EURHE07269	KFW--QNGISACAVCDGAVPIFRNK-PLFVIGGGDSAAEEAMFLAKYGSHVTVLVRRDK-

ASPAC07301	KYW--QNGISACAVCDGAVPIFRNK-PLFVIGGGDSAAEEAMFLAKYGSSVTVLVRKDK-
EMENI10387	VYW--QNGISACAVCDGAVPIFRNK-PLFVIGGGDSAAEEAIFLTKYGSSVTVLVRRDK-
EMEND02596	VYW--QNGISACAVCDGAVPIFRNK-PLFVIGGGDSAAEEAIFLTKYGSSVTVLVRRDK-
ASPTN06742	KYW--QNGISACAVCDGAVPIFRNK-PLYVIGGGDSAAEEAMFLAKYGSSVTVLVRRDK-
ASPCL04014	TYW--QNGISACAVCDGAVPIFRNK-PLYVIGGGDSAAEEAMFLAKYGSSVTVLVRRDK-
ASPFU05647	TYW--QNGISACAVCDGAVPIFRNK-PLYVIGGGDSAAEEAMFLAKYGSSVTVLVRRDK-
NEOFI00452	TYW--QNGISACAVCDGAVPIFRNK-PLYVIGGGDSAAEEAMFLAKYGSSVTVLVRRDK-
CRYPA10563	KYW--NNGVSACAVCDGAAPIFRKK-PLVVIGGGDSAAEEAMFLTKYASHVTVLVRRDQ-
BLUGR03498	KYW--QSGISACAVCDGAVPIFRNK-PLVVIGGGDSAAEEAIFLTKYGSHVTVLVRKEK-
SCLS112814	KYW--QNGISACAVCDGAVPIFRNK-PLVVIGGGDSAAEEAMFLTKYGSHVTVLVRKDH-
MAGGR04266	KYW--QNGISACAVCDGAVPIFRNK-PLFVIGGGDSAAEEAMFLTKYGSHVTVLVRKDK-
NEUCR01575	KYW--QNGISACAVCDGAVPIFRNK-HLVVIGGGDSAAEEAMYLTKYGSHVTVLVRKDK-
NEUT908941	KYW--QNGISACAVCDGAVPIFRNK-HLVVIGGGDSAAEEAMYLTKYGSHVTVLVRKDK-
VERDA02342	KYW--QNGISACAVCDGAVPIYRNK-PLVVVGGGDSAAEEALFLTKYASHVTVLVRRDV-
COLSU12486	KYW--QNGVSACAVCDGAVPIFRNK-HLVVIGGGDSAAEEALYLTKYGSHVTVLVRRDV-
HYPAI01684	KYW--QNGISACAVCDGAVPIFRNK-PLFVIGGGDSAAEEATFLTKYGSHVTVLVRRDQ-
HYPVG06080	KYW--QNGISACAVCDGALPIFRNK-PLFVIGGGDSAAEEATFLTKYGSHVTVLVRRDV-
HYPJE05895	KYW--QNGISACAVCDGAVPIFRNK-PLFVIGGGDSAAEEATFLTKYGSHVTVLVRRDQ-
NECHA05020	KYW--QNGVSACAVCDGAVPIFRNK-PLFVIGGGDSAAEEATFLTKYASHVTVLVRRDV-
FUSO415847	KYW--QNGVSACAVCDGAVPIFRNK-PLFVIGGGDSAAEEATFLTKYASHVTVLVRRDV-
GIBZA01026	KYW--QNGVSACAVCDGAVPIFRNK-PLFVIGGGDSAAEEATFLTKYASHVTVLVRRDV-
SCHPO04025	TYW--QAGISACAVCDGAVPIYRNK-PLAVVGGGDSAAEEAQFLTKYGSKVYVLVRRDK-
YARLI03635	QYW--QQGISACAVCDGAVPIFRNK-PLAVVGGGDSAAEEALFLTKYGSKVYVIVRKDK-
ASHGO00946	TYW--QRGISACAVCDGAVPIFRNK-PLAVVGGGDSACEEASFLTKYGSKVFMLVRKDH-
KLULA02190	TYW--QQGISACAVCDGAVPIFRNK-PLAVIGGGDSACEEAQFLTKYGSKVYMLVRKDH-
ZYGRO00676	TYW--QQGISACAVCDGAVPIFRNK-PLAVIGGGDSACEEAGFLTKYGSKVYLLVRKDH-
DEKBR01813	KYW--QRGISACAVCDGAVPIFRNK-PLAVIGGGDSACEEALFLTKYGSKVYMLVRKDH-
PICPG04776	TYW--QQGISACAVCDGAIPLFRNK-PLAVVGGGDSACEEALFLTKYASKVYMIVRKDY-
CANTE00916	TYW--QQGISACAVCDGAVPIFRNK-PLAVIGGGDSACEEALFLTKYGSKVYLMVRRDQ-
LODEL03891	KYW--QQGISACAVCDGAVPIFRNN-PLAVVGGGDSACEEALFLTKYASKVYLLVRRDQ-
DEBHA05546	TYW--QQGISACAVCDGAVPIFRNK-PLAVVGGGDSACEEALFLTKYGSKVYLLVRRDQ-
SPAPN03477	TYW--QQGISACAVCDGAVPIFRNK-PLAVIGGGDSACEEALFLTKYGSKVYLLVRRDQ-
CANAW04800	TYW--QQGISACAVCDGAVPIFRNN-PLAVIGGGDSACEEAIFLTKYASKVFLLVRRDV-
PICST04701	TYW--QQGISACAVCDGAVPIFRNK-PLAVIGGGDSACEEALFLTKYASKVYLLVRRDV-
PUCGT10887	TYW--QSGISACAVCDGAVPIFRDK-PLAVIGGGDSACEEATYLTKYASKVFVLV-----
PUCGR11813	TYW--QSGISACAVCDGAVPIFRDK-PLAVIGGGDSACEEATYLTKYASKVFVLV-----
PHYBL11006	IYW--QNGISACAVCDGAVPIFRNK-PLVVIGGGDSAAEEAIYLTKYASHVHVLVRRDK-
USTMA03757	TYW--QSGISACAVCDGAVPIFRNK-PLVVVGGGDSAAEEAMYLTKYASHVHVLVRRDE-
USTHO04132	TYW--QSGISACAVCDGAVPIFRNK-PLVVVGGGDSAAEEAMYLTKYASHVHVLVRRDE-
WALSE04527	TYW--QSGISACAVCDGAAPIFRNK-SLAVIGGGDSAAEEATYLTKYASHVHVLVRRHE-
TREME07701	TYW--QSGISACAVCDGAVPIFRNK-PLAVIGGGDSAAEEATYLTKYGSHVYVLVRREE-
AURST04751	TYW--QSGISACAVCDGAVPIFRNK-PLAVIGGGDSAAEEAIYLTKYASRVFVLVRRDE-
FOMME10177	KYW--QSGISACAVCDGAVPIFRNR-PLAVIGGGDSAAEEATYLTKYGSHVYVLVRRDE-
CONPW06392	DYW--QSGISACAVCDGAVPIFRNK-PLAVIGGGDSAAEEATYLTKYGSHVYVLVRRGE-
STEHR07076	TYW--QSGISACAVCDGAVPIFRNK-PLAVIGGGDSAAEEATYLTKYGSHVYLLVRRNE-
HETAN06295	TYW--QSGISACAVCDGAVPIFRNK-PLAVIGGGDSAAEEATYLTKYGSHVYVLVRRNE-
GLOTR06982	TYW--QSGISACAVCDGAVPIFRNK-PLAVIGGGDSAAEEATYLTKYGSHVYVLVRRSE-
PUNST01981	TYW--QSGISACAVCDGAVPIFRNK-PLAVIGGGDSAAEEATYLTKYGSHIYVLVRRGE-
LACBI02877	TYW--QSGISACAVCDGAVPIFRNK-PLAVIGGGDSAAEEATYLTKYGSHVYVLVRRGE-
COPCI16429	TYW--QSGISACAVCDGAVPIFRNK-PLAVIGGGDSAAEEATYLTKYGSHVYVLVRRGE-
DICSQ11618	IYW--QSGISACAVCDGAVPIFRNK-PLAVIGGGDSAAEEATYLTKYGSHVYVLVRRGE-
TRAVS13180	IYW--QSGISACAVCDGAVPIFRNK-PLAVIGGGDSAAEEATYLTKYGSHVYVLVRRGE-
WOLCO03584	TYW--QSGISACAVCDGAVPIFRNK-PLAVIGGGDSAAEEATYLTKYGSHVYVIVRRAE-
FOMPI05979	TYW--QSGISACAVCDGAVPIFRNK-PLAVIGGGDSAAEEATYLTKYGSHVYVLVRRGE-
PHLGI10219	TYW--QSGISACAVCDGAVPIFRNK-PLAVIGGGDSAAEEATYLTKYGSHVYVLVRRGE-
PHACH05757	TYW--QSGISACAVCDGAVPIFRNK-PLAVIGGGDSAAEEATYLTKYGSHVYVLVRRGE-
RICTY00419	EFR--GFGVSSCAICDGF--FFKNQ-DIVVVGGGNSALEEALYLTNHANKVTVVHRRNS-
RICPR00429	EFR--GFGVSSCAICDGF--FFKNQ-EIVVVGGGNSALEEALYLTNHANKVTVVHRRNS-
RICPP00461	EFR--GFGVSSCAICDGF--FFKNQ-EIVVVGGGNSALEEALYLTNHANKVTVVHRRNS-
RICBR00434	EFM--GFGVSACATCDGF--FFKNQ-KVVVVGGGNSAVEEALYLTNHASKVTIVHRRDN-
RICB800988	EFM--GFGVSACATCDGF--FFKNQ-EVVVVGGGNSAVEEALYLTNHASKVTIVHRRDN-
RICCK00600	EFR--GFGVSACATCDGF--FFKNQ-EIVVVGGGNSAVEEALYLTNHANKVTIVHRRDS-
RICAH00614	DFM--GFGVSACATCDGF--FFKNQ-EIVVVGGGNSAVEEALYLTNHASKVTIVHRRDS-
RICAC00782	EFM--GFGVSACATCDGF--FFKNQ-EIVVVGGGNSAVEEALYLTNHASKVTIVHRRDS-
RICFE00656	EFR--GFGVSACATCDGF--FFKNQ-EIVVVGGGNSAVEEALYLTNHASKVTITHRRDS-

```
RICMS00042      KFR--GFGVSACATCDGF--FFKNQ-EIVVVGGGNSAVEEALYLTNHASKVTIVHRRDS-
RICM500453      KFR--GFGVSACATCDGF--FFKNQ-EIVVVGGGNSAVEEALYLTNHASKVTIVHRRDS-
RICR300665      KFR--GFGVSACATCDGF--FFKNQ-EIVVVGGGNSAVEEALYLTNHASKVTIVHRRDS-
RICAG00651      KFR--GFGVSACATCDGF--FFKNQ-EIVVVGGGNSAVEEALYLTNHASKVTIVHRRDS-
RICP300630      KFR--GFGVSACATCDGV--FFKNQ-AIVVVGGGNSAVEEALYLTNHANKVTIVHRRDS-
RICRS00632      KFR--GFGVSACATCDGV--FFKNQ-EIVVVGGGNSAVEEALYLTNHANKVTIVHRRDS-
RICRO00663      KFR--GFGVSACATCDGV--FFKNQ-EIVVVGGGNSAVEEALYLTNHANKVTIVHRRDS-
RICCN00618      KFR--GFGVSACATCDGF--FFKNQ-EIVVVGGGNSAVEEALYLTNHANKVTIVHRRDS-
RICPT00620      KFR--GFGVSACATCDGF--FFKNQ-EIVVVGGGNSAVEEALYLTNHANKVTIVHRRDS-
RICAE00497      KFR--GFGVSACATCDGF--FFKNQ-EIVVVGGGNSAVEEALYLTNHANKVTIVHRRDS-
RICJY00467      KFR--GFGVSACATCDGF--FFKNQ-EIVVVGGGNSAVEEALYLTNHANKVTIVHRRDS-
RICPU00072      KFR--GFGVSACATCDGF--FFKNQ-EIVVVGGGNSAVEEALYLTNHANKVTIVHRRDS-
RICS100537      KFR--GFGVSACATCDGF--FFKNQ-EIVVVGGGNSAVEEALYLTNHANKVTIVHRRDS-
BARBK00891      TFM--GGGVSACATCDGF--FYRDK-EVIVVGGGNRAVEEALYLSRLAKKVVVVHRRDH-
BARVW00932      TFM--GGGVSACATCDGF--FYRNK-DVIVVGGGNTAVEEALYLSHLAKSVSVVHRRDY-
BART100617      TFM--GGGVSACATCDGF--FYRDK-DVIVVGGGNTAVEEALYLSHIAKSVSVVHRREQ-
BARGA00466      TFM--GGGVSACATCDGF--FYRDK-DVIVVGGGNTAVEEALYLSHLAKSVSVVHRRGQ-
BARHE01107      VFM--GGGVSACATCDGF--FYRDK-DVIVVGGGNTAVEEALYLSHLAKRVSVVHRRDY-
BARQU00895      TFM--GGGVSACATCDGF--FYRDK-DVIVVGGGNTAVEEALYLSHLAKRVSIVHRRNH-
OCHA401642      TFM--GGGVSACATCDGF--FYRGK-DVVVVGGGNTAVEEALYLSHIAKSVTVVHRRDG-
BRUAB01390      TFM--GGGVSACSTCDGF--FYRSK-DVVVVGGGNTAVEEALYLSHIAKSVTIVHRRDG-
BRUA201507      TFM--GGGVSACSTCDGF--FYRSK-DVVVVGGGNTAVEEALYLSHIAKSVTIVHRRDG-
BRUA101339      TFM--GGGVSACSTCDGF--FYRSK-DVVVVGGGNTAVEEALYLSHIAKSVTIVHRRDG-
BRUSU01458      TFM--GGGVSACATCDGF--FYRGK-DVVVVGGGNTAVEEALYLSHIAKSVTIVHRRDG-
BRUME00510      TFM--GGGVSACATCDGF--FYRGK-DVVVVGGGNTAVEEALYLSHIAKSVTIVHRRDG-
BRUSI01461      TFM--GGGVSACATCDGF--FYRGK-DVVVVGGGNTAVEEALYLSHIAKSVTIVHRRDG-
BRUC201446      TFM--GGGVSACATCDGF--FYRGK-DVVVVGGGNTAVEEALYLSHIAKSVTIVHRRDG-
BRUMC01440      TFM--GGGVSACATCDGF--FYRGK-DVVVVGGGNTAVEEALYLSHIAKSVTIVHRRDG-
BRUMB01421      TFM--GGGVSACATCDGF--FYRGK-DVVVVGGGNTAVEEALYLSHIAKSVTIVHRRDG-
BRUM501487      TFM--GGGVSACATCDGF--FYRGK-DVVVVGGGNTAVEEALYLSHIAKSVTIVHRRDG-
BRUO201285      TFM--GGGVSACATCDGF--FYRGK-DVVVVGGGNTAVEEALYLSHIAKSVTIVHRRDG-
RHILO01973      DFM--GFGVSACATCDGF--FYRGK-DVAVVGGGNSAVEEALYLSNLAKSVTVIHRRSD-
CHESB02097      TFM--GFGLSACATCDGF--FYRGK-EVVVIGGGNTAVEEALYLSNLAKRVTVIHRRGE-
METPB00996      KFQ--GFGVSACATCDGF--FFRGK-DVTVVGGGNTAVEEALYLANLAKSVTVIHRRGE-
METEP01063      KFQ--GFGVSACATCDGF--FFRGK-DVTVVGGGNTAVEEALYLANLAKSVTVIHRRGE-
METEA00810      KFQ--GFGVSACATCDGF--FFRGK-DVTVVGGGNTAVEEALYLANLAKSVTVIHRRGE-
METED01453      KFQ--GFGVSACATCDGF--FFRGK-DVTVVGGGNTAVEEALYLANLAKSVTVIHRRGE-
METS403554      KFQ--GFGVSACATCDGF--FFRGK-EVLVVGGGNTAVEEALYLANLAAKVTVVHRRDA-
METNO05482      KFQ--GFGVSACATCDGF--FFRGK-EVVVVGGGNTAVEEALYLANLASKVTLVHRRDA-
METSZ03234      RFK--GFGVSACATCDGF--FFRGK-KVLVVGGGNSAVEEALYLSNLASHVTIIHRRDE-
BEII900056      TFK--GYGVSACATCDGF--FYRGK-DVFVVGGGNTAVEEALYLSNLASKVTIIHRGER-
METSB02743      AFK--GYGVSACATCDGF--FYRGK-DVIVVGGGNSAVEEALYLSHLAARVTVVHRGSA-
MAGMM00401      KLN--GFGVSACATCDGF--FFKNQ-DVCVVGGGDTAVEEALYLSNICNKVYLIHRRDA-
HYPNA00542      KFK--GFGVSACATCDGF--FYRGK-EVLVIGGGNTAVEEALFLTNFASKVTVIHRRDH-
KETVY00902      HFR--GFGVSACATCDGF--FYRGQ-EIVVVGGGNTAVEEALFLTNFASKVTLVHRRDS-
KETVW00472      HFR--GFGVSACATCDGF--FYRGQ-EIVVVGGGNTAVEEALFLTNFASKVTLVHRRDS-
ROSDO03242      KFK--GFGVSACATCDGF--FYRGQ-EIAVIGGGNTAVEEALFLTNFASKVTLVHRRDE-
ROSLO02615      KFK--GFGVSACATCDGF--FYRGQ-EIVVIGGGNTAVEEALFLTNFASKVTLVHRRDE-
RUEPO00888      KFK--GFGVSACATCDGF--FYRGQ-EIVVIGGGNTAVEEALFLTNFASKVTLIHRRDE-
RUEST00613      KFK--GFGVSACATCDGF--FYRGQ-EIVVIGGGNTAVEEALFLTNFASKVTLIHRRDE-
PHAIB02390      KFK--GFGVSACATCDGF--FYRGQ-EIVVIGGGNTAVEEALFLTNFASKVTLIHRRDE-
PARDP02130      KFK--GFGVSACATCDGF--FYRGR-EVVVVGGGNTAVEEALFLTNFASKVTLIHRRDS-
DINSH02620      HFK--GFGVSACATCDGF--FYRGK-EVVVIGGGNTAVEEALFLTNFASKVTLVHRRDS-
RHOCB02768      KFQ--GFGVSACATCDGF--FYRGK-EVVVVGGGNAAVEEAMFLTNFASKVTVIHRRDS-
RHOS500248      KFK--GFGVSACATCDGF--FYRGK-EVVVAGGGNSAVEEALFLTNFATKVTVIHRRDS-
RHOS400150      RFK--GFGVSACATCDGF--FYRGK-EVVVAGGGNTAVEEALFLTNFASKVTLVHRRDS-
RHOS100227      RFK--GFGVSACATCDGF--FYRGK-EVVVAGGGNTAVEEALFLTNFASKVTLVHRRDS-
RHOSK02952      RFK--GFGVSACATCDGF--FYRGK-EVVVAGGGNTAVEEALFLTNFASKVTLVHRRDS-
MIDMI00790      KFK--GFGVSGCATCDGF--FYKNK-KVIVVGGGNSALEEALYLTQHAAEVILIHRRDT-
ACEP301595      HLQ--GAGVSACATCDGF--FYRGK-NVVVVGGGNTAVEEALYLTHHAAHVTLVHRRDS-
MICAA01566      TFR--GLGVSACATCDGF--FFRGK-KVAVVGGGSAAVEEAIFLTNFCEKVTLIHRRDT-
TISMK03676      LLK--GFGLSACATCDGF--FYRNR-TVAVVGGGNTAVEEALYLTNHADKVYLIHRRDA-
AZOL402409      IYR--GFGVSACATCDGF--FFRGK-EVAVIGGGNSAVEEALYLTNHASKVTVIHRRNS-
PSEUV04348      KYQ--AGGVSACATCDGF--FYRGK-EVVVVGGGNTAVEEALYLANLASKVTLVHRRDA-
HIRBI01095      KFQ--GFGVSACATCDGF--FYRGK-EVIVVGGGNTAVEEALFLTNFASKVTLVHRRDS-
```

```
PARL102241      KFQ--GFGVSACATCDGF--FYRGK-EVLVVGGGNSAVEEALFLTNFATKVTVIHRRDT-
MARMM02139      KFQ--GFGVSACATCDGF--FYRGK-EVVVVGGGNTAVEEALFLTNFASKVTLVHRRDS-
PHEZH02588      KFQ--GFGVSACATCDGF--FYRGK-NVVVVGGGNTAVEEALFLTNFAAKVTLVHRRDE-
CAUCR02826      KFQ--GFGVSACATCDGF--FYRNK-DVIVVGGGNTAVEEALFLTSFASKVTLVHRKDE-
CAUCN02939      KFQ--GFGVSACATCDGF--FYRNK-DVIVVGGGNTAVEEALFLTSFASKVTLVHRKDE-
CAUST00891      KFQ--GFGVSACATCDGF--FYRNK-EVVVVGGGNTAVEEALFLTSFASKVTLVHRKDE-
PARBH01522      KFQ--GFGVSACATCDGF--FYRGR-TVGVVGGGNTAVEEALFLTNFADKVYLIHRRDS-
PELHB02084      KFK--GFGVSACATCDGF--FYRDK-TVLVIGGGNTAVEEALFLTNFASKVIIAHRRDQ-
HYPDA03273      HFQ--GHGVSACATCDGF--FYKGK-DVVVVGGGNTAVEEALFLTNFANKVTLVHRRDF-
HYPSM04642      HFK--GHGVSACATCDGF--FYKGK-DVVVIGGGNTAVEEALFLTNFANKVTLVHRRDF-
OLICO01107      KFK--GFGVSACATCDGF--FYRNK-DVFVVGGGNTAVEEALYLTNHASSVTLVHRRDS-
OLICM02790      KFK--GFGVSACATCDGF--FYRNK-DVFVVGGGNTAVEEALYLTNHASSVTLVHRRDS-
RHOPS01446      TYK--GFGVSACATCDGF--FYRGK-DVVVVGGGNTAVEEAMFLTNFASSVTVVHRRDH-
RHOPA04062      TYK--GFGVSACATCDGF--FYRGK-DVVVVGGGNTAVEEALFLTNFAASVTIVHRRDH-
RHOPT04533      TYK--GFGVSACATCDGF--FYRGK-DVVVVGGGNTAVEEALFLTNFAASVTIVHRRDH-
RHOPX04235      TYK--GFGVSACATCDGF--FYRGK-DVVVVGGGNTAVEEALFLTNFAASVTVVHRRDH-
BRADU07321      KFQ--GGGVSACATCDGF--FYRGK-EVIVVGGGNTAVEEALFLTNHASQVTIVHRRDH-
BRASO01239      TFK--GFGVSACATCDGF--FYRGK-EVIVVGGGNTAVEEALFLTNFASTVTVVHRRDH-
BRASB06352      TFK--GFGVSACATCDGF--FYRGK-EVIVVGGGNTAVEEALFLTNFASTVTVVHRRDH-
RHOPB03773      KFK--GFGVSACATCDGF--FYRGK-QVVVVGGGNTAVEEALFLTNFATKVTLVHRRDH-
NITWN02324      TFK--GFGVSACATCDGF--FYRGK-NVIVIGGGNTAVEEALFLTNFASQVTLVHRRDH-
NITHX02707      SFK--GFGVSACATCDGF--FYRGK-NVIVVGGGNTAVEEALFLTNFATQVTLVHRRDH-
AZOC501143      AYR--GFGVSACATCDGF--FYRGK-DVVVVGGGNTAVEEALFLTNFASKVTLVHRRDK-
XANP202679      AYR--GSGVSACATCDGF--FYRGK-DVVVVGGGNTAVEEALFLTNFASNVTVVHRRDS-
CHLTF01983      HLK--GYGVSACATCDGF--FFKDR-EVAVVGGGDTAVEEATFLTKYATKVTLIHRRRE-
IGNAJ02236      KYM--GYGVSACATCDGF--FFKGL-KVIVVGGGDTAMEEANFLTKFASEVIIVHRRDE-
MELRP00403      KYM--GYGVSACATCDGF--FFKDL-KVLVVGGGDTAMEEATYLTKFASEVTIIHRRDE-
ANADF00468      QYQ--GRGVSACATCDGF--FFRGV-DVAVVGGGDTAMEEAIFLTKYATKVYVIHRRSE-
CHLCH01125      RYR--GKGVSACATCDGF--FFRNC-HVMVVGGGDTAMEEALYLTKFASKVTLVHRRGE-
PELPB01419      RYR--GKGVSACATCDGF--FFRDC-KVFVVGGGDTAMEEALYLTKFASHVTLVHRREE-
CHLL200781      QYR--GRGVSACATCDGF--FFKEC-NVFVVGGGDTAMEEALYLTKFASKVTLVHRREE-
CHLTE00830      KYR--GRGVSACATCDGF--FFRNC-RVFVVGGGDTAMEEALYLTKFASEVVLVHRREE-
CHLP800854      TYR--GRGVSACATCDGF--FFRNC-RVFVVGGGDTAMEEALYLTKFASEVVLVHRREE-
CHLL701225      RYR--GRGVSACATCDGF--FFRDS-TVFVIGGGDTAMEEALYLTKFASRVVLVHRREE-
CHLPM00993      RYR--GRGVSACATCDGF--FFRDS-TVFVIGGGDTAMEEALYLTKFASRVVLVHRREE-
WOLTR00562      KFQ--GYGVSACATCDGA--FFRNK-IVAVIGGGNTAVEEAIFLTRFAKEVILIHRRDK-
WOLPP00135      KFQ--GYGVSACATCDGA--FFRNK-VVAVVGGGNTAVEEAIFLTRFAKEVILIHRRDN-
WOLPM00650      KFQ--GYGVSACATCDGA--FFRNK-VVAVIGGGNTAVEEAIFLTRFAKEVILIHRRDK-
WOLWR00416      EFQ--GYGVSACATCDGA--FFRNK-VVAVIGGGNTAVEEAIFLTRFAKEVILIHRRDK-
EHRCR00696      TFK--GRGVSACATCDGT--FFAGS-DIAVIGGGNTAVEEALYLTRYATKVFLIHRRDT-
ANAMM00345      FFM--GKGVSACATCDGS--FFKGE-VVAVIGGGNTAVEEAIYLTRSASKVFLVHRRDK-
ANAMF00341      FFM--GKGVSACATCDGS--FFKGE-VVAVIGGGNTAVEEAIYLTRSASKVFLVHRRDK-
ANAPZ00610      NFK--SKGVSACATCDGA--FFKDE-VVAVIGGGNTAVEEALYLTRSSKKVFLIHRRDK-
NEOSM00537      KYQ--GYGVSGCATCDGF--FFRDQ-DVIVIGGGNTAVEEALYLTRHAKKVYLLHRRER-
NEORI00513      KYQ--GYGVSGCATCDGF--FFRDQ-DVIVIGGGNTAVEEALYLTRHAKKVYLLHRRDR-
PELUB00076      AYR--GFGVSACATCDGF--FFKEK-VVAVVGGGNAAVEEAMFLTKFASKVKLIHRRDT-
PELSM00736      KFR--GFGVSACATCDGF--FYKDK-EVMVVGGGNAAVEEALFLTKFASKVTLVHRRDT-
PUNMI01409      TFN--GRGVSACATCDGF--FYKGR-DVAVIGGGNTAVEEALYLANICNKVTLVHRRDS-
ASTEC00651      TYQ--GFGVSACATCDGF--FYRNK-TVAVVGGGNTAVEEALFLTKFASKVYLIHRRDS-
ZYMMT00154      ALR--GKGISACATCDGF--FFRGK-KVAVIGGGNTAVEEALYLTNHSPEVTLIHRRDS-
ZYMMO00984      ALR--GKGISACATCDGF--FFRGK-KVVVIGGGNTAVEEALYLTNHSPEVTLIHRRDS-
ZYMMA00176      ALR--GKGISACATCDGF--FFRGK-KVVVIGGGNTAVEEALYLTNHSPEVTLIHRRDS-
ZYMMN00183      ALR--GKGISACATCDGF--FFRGK-KVVVIGGGNTAVEEALYLTNHSPEVTLIHRRDS-
SPHAL00143      ELG--GKGVSACATCDGF--FYRGK-KVVVIGGGNTAVEEALYLTNHSDDVTLIHRRDS-
SPHWW03978      ALG--GKGVSACATCDGF--FYRGK-KVAVIGGGNTAVEEALYLTNHSQDVTLIHRRDS-
SPHJU02313      LLQ--GKGVSACATCDGF--FYRGK-KVVVIGGGNTAVEEALYLTNHSHDVTLIHRRDS-
NOVAD02319      EFS--GKGVSACATCDGF--FYRGK-KVVVIGGGNTAVEEALYLTNHSPDVTLIHRRDS-
ERYLH00934      ELG--GKGVSACATCDGF--FYRGK-KVAVIGGGNTAVEEALYLTNHSDDVTLIHRRDE-
GRABC00720      RLQ--GAGVSACATCDGF--FFRGK-RVAVIGGGNTAVEEALYLTHHASEVVLIHRRDS-
GLUDA03075      QYQ--GSGVSACATCDGF--FYRGR-TVAVIGGGNTAVEEALYLTHHAEHVILIHRRDS-
KOMMN00774      EFQ--GSGVSACATCDGF--FYRGK-RVAVIGGGNTAVEEALYLTHHASHVTLIHRRDS-
HALVD01097      NLM--GYGLSTCATCDGA--FFRDE-KIMVIGGGDAAVEEANFLTKFASTVYLVHRREE-
HALHT01699      ELM--GYGVSTCATCDGA--FFRGE-DMLVVGGGDAAMEEAHFLTKFADTVYIAHRREE-
METI401038      ELE--TKGVSYCATCDGALPVFRNQ-PLVVVGGGDSACEEALYLTRFASSVYLVHRRDT-
PLAL201612      RFK--NYGVSACAVCDGALPRFRNK-PLVVVGGGDSAMEEATYLSKFASKVHLVHRRDE-
```

GEMAT01983	AQS--GGGVSACAVCDGAMPFYRQK-RLIVVGGGDTAMEEAMYLTKFASEVVIVHRRDS-
CYAAP02456	EFW--NNGISACAICDGASPLFRNT-EVAVVGGGDSAAEEALYLTKYASLVYLLVRRDR-
CHLPN00303	EFW--QKGVTACAVCDGASPIFKNK-DLYVIGGGDSALEEALYLTRYGSHVYVVHRRDK-
CHLPP00439	EFW--QKGVTACAVCDGASPIFKNK-DLYVIGGGDSALEEALYLTRYGSHVYVVHRRDK-
CHLPE00727	EFW--QKGVTACAVCDGASPIFKNK-DLFVVGGGDSALEEALFLTRYGKQVYVVHRRDS-
CHLTR00101	EFW--QKGVTACAVCDGASPIFRDK-DLFVVGGGDSALEEAMFLTRYGKRVFVVHRRDT-
CHLTA00102	EFW--QKGVTACAVCDGASPIFRDK-DLFVVGGGDSALEEAMFLTRYGKRVFVVHRRDT-
CHLTJ00101	EFW--QKGVTACAVCDGASPIFRDK-DLFVVGGGDSALEEAMFLTRYGKRVFVVHRRDT-
CHLTD00098	EFW--QKGVTACAVCDGASPIFRDK-DLFVVGGGDSALEEAMFLTRYGKRVFVVHRRDT-
CHLT700102	EFW--QKGVTACAVCDGASPIFRDK-DLFVVGGGDSALEEAMFLTRYGKRVFVVHRRDT-
CHLT000101	EFW--QKGVTACAVCDGASPIFRDK-DLFVVGGGDSALEEAMFLTRYGKRVFVVHRRDT-
CHLT500104	EFW--QKGVTACAVCDGASPIFRDK-DLFVVGGGDSALEEAMFLTRYGKRVFVVHRRDT-
CHLTL00098	EFW--QKGVTACAVCDGASPIFRDK-DLFVVGGGDSALEEAMFLTRYGKRVFVVHRRDT-
CHLTG00102	EFW--QKGVTACAVCDGASPIFRDK-DLFVVGGGDSALEEAMFLTRYGKRVFVVHRRDT-
CHLTS00101	EFW--QKGVTACAVCDGASPIFRDK-DLFVVGGGDSALEEAMFLTRYGKRVFVVHRRDT-
CHLT900102	EFW--QKGVTACAVCDGASPIFRDK-DLFVVGGGDSALEEAMFLTRYGKRVFVVHRRDT-
CHLTZ00101	EFW--QKGVTACAVCDGASPIFRDK-DLFVVGGGDSALEEAMFLTRYGKRVFVVHRRDT-
CHLT400432	EFW--QKGVTACAVCDGASPIFRDK-DLFVVGGGDSALEEAMFLTRYGKRVFVVHRRDT-
CHLT100103	EFW--QKGVTACAVCDGASPIFRDK-DLFVVGGGDSALEEAMFLTRYGKRVFVVHRRDT-
CHLT200350	EFW--QKGVTACAVCDGASPIFRDK-DLFVVGGGDSALEEAMFLTRYGKRVFVVHRRDT-
CHLTB00350	EFW--QKGVTACAVCDGASPIFRDK-DLFVVGGGDSALEEAMFLTRYGKRVFVVHRRDT-
CHLTC00378	EFW--QKGVTACAVCDGASPIFRDK-DLFVVGGGDSALEEAMFLTRYGKRVFVVHRRDT-
SIMNZ00831	EFW--QKGVTACAVCDGAMPIFRQK-ELYVIGGGDTAVEEAIFLTKYGKRVYIVHRRDK-
PARUW01706	EFW--QKGVTACAVCDGAAPIFRNR-PLFVIGGGDSAIEEATFLTKFGSRVFIVHRRDT-
WADCW00943	EFW--QKGVTACAVCDGAMPIFRNQ-KLYVIGGGDSACEEAIFLTKFGSEVFIVHRRDE-
SINAD04980	HYM--GYGLSTCATCDGA--LYRGK-QVAVVGGGDSAVEEANFLTRYAAKVTLIHRRDA-
BIFLB01558	EYS--GKGVSYCATCDGF--FFRGK-PIVVVGGGDSAMEEANFLARFGSSVTLIHRRDE-
BIFAB00498	EYS--GKGVSYCATCDGF--FFRGK-PIVVVGGGDSAMEEANFLARFGSSVTLIHRRDE-
BIFAV01561	EYS--GKGVSYCATCDGF--FFRGK-PIVVVGGGDSAMEEANFLARFGSSVTLIHRRDE-
BIFAS01557	EYS--GKGVSYCATCDGF--FFRGK-PIVVVGGGDSAMEEANFLARFGSSVTLIHRRDE-
BIFA001518	EYS--GKGVSYCATCDGF--FFRGK-PIVVVGGGDSAMEEANFLARFGSSVTLIHRRDE-
BIFBA01814	ELS--GHGVSYCATCDGF--FFKDK-PIVVVGGGDSAFEEALFLTRFGSSVTLIHRRDS-
BIFAA01619	EYA--GKGVSYCATCDGF--FFRNK-PIVVVGGGDSAFEEADFLSRFGSSVTLIHRRST-
BIFDB02112	EFS--GKGVSYCATCDGF--FFRDK-PIVVIGGGDSAFEEADFLSRFGSSVTLIHRRDS-
TERSS03091	TLI--GHGVSSCATCDGF--FFSGK-DICVIGGGDSAMEEALFLTRFATKVYVIHRSES-
GRATM03160	ALI--GHGVSSCATCDGF--FASGK-DIAVIGGGDSAMEEALFLTRFASKVTLINRTEK-
GRAMM03836	ALI--GHGVSSCATCDGF--FASGK-EIAVIGGGDSAMEEALFLTRFATKVTIINRSEN-
AKKM801247	GLV--GHGLTACATCDGA--FYRDV-PVCVVGGGDSACEEAMFLTRFASRVYLIHRRDT-
OPITP04130	ELY-GGKGVTTCATCDGA--FYRNM-DVAVIGGGDSAAEEALFLTRFASKVYLVHRRDT-
CORAD01794	EMF-GGKGVTTCATCDGA--FYRDM-EVVVVGGGDSACEEALFLTRFCSKVTLVHRRDE-
BUCCC00180	LYI--GKGISTCAICDGF--FYKNQ-IVAVVGGGNSAIEEVIYLSNIVKKIYLIHRRNK-
CENSY00347	AFA--GKGVSYCATCDGP--FFKNM-ELVVVGGGDSAMEEATFLTKFATTVHVVHRRKE-
NITMS00668	TFG--GKGVSYCATCDGP--FFRNQ-ELVVVGGGDSAVEEATFLTKFATTVHLVHRRDE-
MEIRD02823	KFY--GRGVSTCATCDGF--FYRGK-EVVVVGGGDAAVEEGLFLTKFASKVTLVHRRDT-
HERA203780	ELA--NRGVSYCAVCDGF--FFRDK-TLVVVGGGNSALDESLFLTRYAKEVHIIHRRDQ-
CHLAA02018	ELA--NRGVSYCATCDGF--FFRGK-KVVVVGGGNSALDEGLFLTRYVDELVIVHRRDT-
CHLSY02175	ELA--NRGVSYCATCDGF--FFRGK-KVVVVGGGNSALDEGLFLTRYVDELVIVHRRDT-
CHLAD02599	ELA--NRGVSYCATCDGF--FFRGK-KVVVVGGGNSALDEGLFLTRYVDELVIVHRRDT-
CALAS01745	EFT--GKGVSYCATCDGF--FFRGK-DVIVVGGGDSAAEEALFLTRYASRVRILHRRDK-
ANATU01696	ELT--GKGVSYCATCDGW--FFKDK-EVVVVGGGDSALEEGIFLTRYATKVTIIHRRDT-
SULMS00235	ALI--GHGISTCATCDGF--FFKKK-DVAIIGGGDKALEEAIFLSKLCNRIYIIIRKSK-
BLASB00041	RLT--GLGVSFCATCDGF--FHKGK-DVAVIGGGDTALEEANYLSKICKKVYLIVRKNY-
BLASP00551	KFL--GLGVSFCATCDGF--FHKGK-DVAVIGGGDTALEEASYLSKICRNVYLVVRKNY-
AZOPC00437	KYI--GRGVSACAVCDGF--FYRKK-IVAIVGGGDSACEEALYLASIANKVYLIVRKNH-
LEPBD01878	ENI--GRGVSACATCDGF--FYRGK-DVVVIGGGDTAMEEAVFLTKFANKVTVIHRRDT-
SALRD02577	RLI--GKGVSACATCDGS--FFRGE-TVAVVGGGDSAMEESTFLTKFAEKVYVLHRREE-
SALRM02866	RLI--GKGVSACATCDGS--FFRGE-TVAVVGGGDSAMEESTFLTKFAEKVYVLHRREE-
RIEPU00166	RFE--GKGVSFCATCDGF--FYRNQ-KIAVVGGGNNALEEAIYLSKIAKEVNLIHRSKK-
ORITB00015	TFR--GYGVSSCATCDGF--FFRNK-RVVVVGGGNTAVEEALHLTNLTSQVTLIYRGDEK
ORITI01234	TFR--GYGVSSCATCDGF--FFRNK-RVVVVGGGNTAVEEALHLTNLTTQVTLIYRGDEK
PREMB01541	KYR--GQGVSACATCDGF--FYRKR-TVAVVGGGDTACEEAMYLSGLANKVYMIVRKPQ-
PREDF01155	KYR--GQGVSACATCDGF--FYRKR-TVAVVGGGDTACEEAMYLSGLAKKVYMIVRKPY-
PREI702011	KYR--GQGVSACATCDGF--FYRKR-TVAVVGGGDTACEEAMYLSSLAKKVYMIVRKPY-
ALIFI01035	KFR--GMGVSACATCDGF--FYRKK-DVAVVGGGDTACEEATYLASICRKVYMIVRKPH-
ODOSD00362	KFR--GLGVSACATCDGF--FYRGK-DVAVVGGGDTACEEATYLAGLCRKVYLIVRKNY-

PRER201250	KYN--GQGVSACATCDGF--FYRKK-TVAVVGGGDTACEDALYLAGLAKKVYLIVRKPF-
PALPW00206	KYA--GSGVSACATCDGF--FYRKL-TVAVVGGGDTACEEATYLAGLASKVYLIVRKNF-
PORGI00953	KYA--GMGVSACATCDGF--FYRKK-KVAVVGGGDTACEEALYLASLAEHVYLIVRKNY-
PORG301168	KYA--GMGVSACATCDGF--FYRKK-KVAVVGGGDTACEEALYLASLAEHVYLIVRKNY-
BACV803426	KYA--GMGVSACATCDGF--FYRKK-TVAVVGGGDTACEEAVYLAGLAKQVYLIVRKPF-
BACT601486	KYA--GMGVSACATCDGF--FYRKK-TVAVVGGGDTACEEAVYLAGLASKVYLIVRKPF-
BACTN04290	KYA--GMGVSACATCDGF--FYRKK-VVAVVGGGDTACEEAIYLAGLASKVYLVVRKPY-
BACFR01032	KYA--GMGVSACATCDGF--FYRKK-VVAVVGGGDTACEEAIYLAGLASKVYLVVRKPY-
BACFN00913	KYA--GMGVSACATCDGF--FYRKK-VVAVVGGGDTACEEAIYLAGLASKVYLVVRKPY-
BACF600963	KYA--GMGVSACATCDGF--FYRKK-VVAVVGGGDTACEEAIYLAGLASKVYLVVRKPY-
OWEHD03413	AFM--GHGVSACAICDGF--FFKGL-NVVVVGGGDSAAEEATYLAKLCPKVTVLIRKDH-
PSYTT00390	RLR--GGGVSACAVCDGF--FYKGQ-DVAIVGGGDTACEEATYLSNICTKVTMLVRKGD-
NONDD00011	RLR--GGGVSACAVCDGF--FYKNQ-DVAIVGAGDTAAEEASYLAKICKSVTMLVRKDY-
ROBBH02711	RLR--GGGVSACAVCDGF--FYKGQ-DVAIVGGGDTAAEEASYLAKICNKVTMLVRKDH-
CELAD02592	KLR--GGGVSACAVCDGF--FYKNQ-DVAIVGAGDTAAEEASYLANICKSVTMLVRKDE-
CELLC02474	RLR--GGGVSACAVCDGF--FYKGQ-DVAIVGAGDTAAEEASYLANICNNVTMLVRRDE-
MARSH03072	RLR--GGGVSACAVCDGF--FYKGQ-DVAIVGAGDTAAEEASYLANICNKVTMLVRKDY-
MURRD00159	RLR--GGGVSACAVCDGF--FYKGQ-DVAIVGAGDTAAEEASYLANICTKVTMLVRKDY-
ZOBGA00152	RLR--GGGVSACAVCDGF--FYKGQ-DVAIVGAGDTAAEEASYLANICNKVTMLIRKDH-
GRAFK00949	KLR--GGGVSACAVCDGF--FYKGQ-DVAIVGGGDTAAEEATYLSNICNKVTMLVRKDY-
ZUNPS02344	KLR--GGGVSACAVCDGF--FYKNQ-DVAIVGGGDTAAEEATYLANICKKVTMLVRKDE-
AEQSU02079	KLR--GGGVSACAVCDGF--FYKNQ-DVAIVGAGDTAAEEATYLSNICNKVTMLVRKDA-
HALH105745	KFW--SKGVSACAVCDGF--FFRGQ-EVAVVGGGDTAAEEATYLAKLCPKVHLIVRRDQ-
SAPGL02893	RLM--NKGVSACAVCDGF--FFRGQ-EVSVVGGGDTAAEEALYLSKLCPKVHLFVRRDE-
AMOA500014	RLN--GRGVSACATCDGF--FFRGQ-DVAVVGGGDTAAEEALHLSKICNKVYVLVRSDK-
FLAIG01802	HYLKMGGGVSACAVCDGF--FYRNQ-EVVIVGAGDSACEEAHYLSKMCKKVTMLVRTDK-
FLACA02326	HYLNMGGGVSACAVCDGF--FYRNQ-EVVIVGAGDSACEEAHYLSKMCKKVTMLVRSEQ-
FLAJ100198	HYLNMGGGVSACAVCDGF--FYRNQ-EVVIVGAGDSACEEAHYLSKLCKKVTMLVRSEK-
FLABF01528	HYLQLGGGVSACAVCDGF--FYRNQ-EVVIVGAGDSACEEAHYLSKLCKKVTMLVRSEK-
CAPOD01333	HYLQTGGGVSACAVCDGF--FYRNQ-HVAIVGAGDSACEEALYLANLCSKVTMFVRRDE-
CAPCC00414	HYLKTGGGVSACAVCDGF--FYRKQ-KVAIVGAGDSACEEALYLSHICEKVTMLVRRDE-
FLELS01898	EYN--GKGVSACAVCDGF--FFRGQ-TVAIVGGGDTACEEASYLAKLCKKVYMIVRKDE-
SOLCM01166	KYN--GFGVSACAVCDGF--FFRKQ-DVAIVGAGDTAAEEATYLAKLCQKVYMIVRKGE-
PEDHD01598	KYN--GFGVSACAVCDGF--FFRGQ-DVAIVGAGDTAAEEATYLAKLCKKVYMLVRRDE-
SPHS203207	KYN--GFGVSACAVCDGF--FFKGQ-DVAIVGAGDTAAEEATYLAKLCRKVHMLVRRDE-
LEAB401188	RLN--GLGVSACAVCDGF--FYRGK-DVAIVGAGDTACEEAHYLSKLCKKVIMLVRRDE-
EMTOG00092	RLN--GYGVSACAVCDGF--FFRGQ-EVAIVGAGDTACEEAHYLSKLCKKVYMLVRRDE-
SPILD01737	RLN--GRGVSACAVCDGF--FFRGQ-DVAIVGAGDTAAEEASYLSNICRKVYMLVRRDE-
DYAFD00084	RLN--GHGVSACAVCDGF--FFRGQ-DVVVVGAGDTAAEEASYLAKLCRKVYLLVRRDE-
CYCMS03063	RLN--GKGVSACAVCDGF--FFRGK-DVAIVGGGDTACEEASYLANICNKVYMLVRRDE-
ECHVK03326	KLN--GKGVSACAVCDGF--FFRNQ-KVAIVGAGDTACEEASYLANICEKVYMLVRRDE-
BELBD02542	RLN--GKGVSACAVCDGF--FFKGQ-DVAIVGAGDTACEEASYLANICSKVYMIVRKNE-
NITGG02112	QFS--GRGVSYCATCDGP--FFKGE-DIVVVGGGDTALEEATFLTKFGKSVKIVHRRDT-
THEM700111	KLM--GRGVSSCATCDGA--FFRDQ-DVVVVGGGDTAMEEALYLARICRSVTVIHRRDR-
CREAS01350	EFL--GRGVSTCAPCDAP--FFKDM-EVIVVGGGDSAMEEALTLTKYAKNVTVVHRRDQ-
TURPD02772	AYK--GKGVSACATCDGF--FFRGK-EVAVVGGGDSAFEEANFLTKFASKVTLLHRRDT-
SORC507603	KLK--NKGVSACAVCDGA--LFRGQ-DVVVVGGGDTAMEEATYLSGLCSSVTLVHRRDE-
BDEBA00337	VYA--NRGVSACATCDGA--FFRNQ-EIGVVGGGDTAMEEAQFLTRFATKVYLIHRRDH-
STIAD02752	RYK--NRGVSACATCDGA--FYKKQ-DVLVVGGGDTAMEEATYLAKIVNRVTLLHRRDT-
MYXXD01876	TYK--NRGVSACATCDGA--FFKKQ-DVLVVGGGDTAMEEATYLAKIVNHVTLIHRRDT-
MYXFH03454	TYK--NRGVSACATCDGA--FFKKQ-DVLVVGGGDTAMEEATYLAKIVNHVTLIHRRDT-
CORCM01960	TYK--NRGVSACATCDGA--FFKKQ-DVLVVGGGDTAMEEATYLAKIVNHVTLIHRRDS-
MYXSD02209	QFK--NRGVSACATCDGA--FFKNQ-EVLVVGGGDTAMEEATYLAKIVKHVTLIHRRDS-
LEPFC02126	ALM--GAGVSACATCDGF--FFKER-EIVVIGGGDTALEEAIFLTRFGKSVTIVHRRDR-
LEPFM02294	ALM--GQGVSACATCDGF--FFKDK-EIVVVGGGDTAIEEALFLTRFGSKVTIIHRRDS-
SOLUE00582	KLI--GHGVSSCATCDGF--FYRGR-KIMVVGGGDSAMEEANFLSRFGSEVTLVHRREE-
KORVE01333	ELI--GHGVSSCATCDGF--FFSGK-EISVIGGGDSAMEEALFLTRFATKVHLIHRRDA-
ACIC502553	ALI--GHGVSSCATCDGF--FFSGK-EIAVVGGGDSAMEEALFLTRFATKVTLLHRRDH-
ACIFD00030	ELI--GHGVSTCATCDGF--FFRGH-DIAVVGGGDSALEEALFLTKFASSVTIIHRRDQ-
HALMS01402	ELI--GKGVSACATCDGF--FYRDQ-IVHIVGGGDTAMEEATFLTKFAKKVYVVHRRDS-
CHLPD01133	MYR--GKGVSACATCDGF--FFKQC-KVFVVGGGDTAMEEALYLTKFAAEVIIVHRREE-
CHLPB01137	KYQ--GKGVSACATCDGF--FFKES-EVYVVGGGDTAMEEALYLTRFASKVTVVHRRDE-
PROA200897	QYR--GRGVSACATCDGF--FFRDS-EVFVIGGGDTAMEEALFLTRFARHVTVVHRRNE-
WIGBR00492	LYK--GKGVSTCAVCDGF--FYKNK-IVAVIGGGNTALEEALYLSNIASKVYLIHRRDI-
BUCA500289	LFK--GKGVSTCAVCDGF--FYKNK-EVAVVGGGNTAIEETLYLSNFVKKVHLIHRGIN-

BUCAI00292 LFK--GKGVSTCAVCDGF--FYKNK-EVAVVGGGNTAIEETLYLSNFVKKVHLIHRGIN-
BUCAF00306 LFK--GKGVSTCAVCDGF--FYKNK-EVAVVGGGNTAIEETLYLSNFVKKVHLIHRGIN-
BUCAT00287 LFK--GKGVSTCAVCDGF--FYKNK-EVAVVGGGNTAIEETLYLSNFVKKVHLIHRGIN-
BUCA000302 LFK--GKGVSTCAVCDGF--FYKNK-EVAVVGGGNTAIEETLYLSNFVKKVHLIHRGIN-
BAUCH00290 EYK--GKGVSACATCDGF--FYRNL-KVAVIGGGNTAVEEALYLSNIAKEVHLIHRREI-
BLOVB00369 KYK--GKGVSSCATCDGF--FFNKE-TVAVIGGGNTAVEESLYLSNIAHTVHLVHRRSQ-
BLOFL00367 KYK--GKGVSSCATCDGF--FFNKQ-NVAVVGGGNTAIEESLYLSNIANQVYLIHRSMN-
BLOPB00375 KYK--GKGVSSCATCDGF--FFNKQ-IVAVIGGGNTAIEESLYLSNIASKVHLIHRRST-
BUCAP00289 KFK--GKGISTCAVCDGF--FYKNK-EIAVVGGGNTAIEETLYLSNFVKKIYLIHRRNN-
COXBU01001 AYM--GKGVSACATCDGF--FYRGK-KVAVVGGGNTAVEEALYLSHIASHVTLIHRRDK-
COXBN01188 AYM--GKGVSACATCDGF--FYRGK-KVAVVGGGNTAVEEALYLSHIASHVTLIHRRDK-
COXBR01117 AYM--GKGVSACATCDGF--FYRGK-KVAVVGGGNTAVEEALYLSHIASHVTLIHRRDK-
COXB200717 AYM--GKGVSACATCDGF--FYRGK-KVAVVGGGNTAVEEALYLSHIASHVTLIHRRDK-
COXB100914 AYM--GKGVSACATCDGF--FYRGK-KVAVVGGGNTAVEEALYLSHIASHVTLIHRRDK-
FRAP200241 KFM--GKGVSACATCDGF--FYKNK-DVAVVGGGNTAVEEALFLSNIAKSVTLIHRRDS-
FRANT01012 KFM--GKGVSACATCDGF--FYKNK-DVAVVGGGNTAVEEALFLSNIAKSVTLIHRRDS-
FRACN00522 KFM--GKGVSACATCDGF--FYKNK-DVAVVGGGNTAVEEALFLSNIAKSVTLIHRRDT-
FRATT00465 KFM--GKGVSACATCDGF--FYKNK-DVAVVGGGNTAVEEALFLSNIAKSVTLIHRRDT-
FRAT100465 KFM--GKGVSACATCDGF--FYKNK-DVAVVGGGNTAVEEALFLSNIAKSVTLIHRRDT-
FRATE00462 KFM--GKGVSACATCDGF--FYKNK-DVAVVGGGNTAVEEALFLSNIAKSVTLIHRRDT-
FRATW01216 KFM--GKGVSACATCDGF--FYKNK-DVAVVGGGNTAVEEALFLSNIAKSVTLIHRRDT-
FRATM01110 KFM--GKGVSACATCDGF--FYKNK-DVAVVGGGNTAVEEALFLSNIAKSVTLIHRRDT-
FRACF00566 KFM--GKGVSACATCDGF--FYKNK-DVAVVGGGNTAVEEALFLSNIAKSVTLIHRRDT-
FRATO01212 KFM--GKGVSACATCDGF--FYKNK-DVAVVGGGNTAVEEALFLSNIAKSVTLIHRRDT-
FRATH01486 KFM--GKGVSACATCDGF--FYKNK-DVAVVGGGNTAVEEALFLSNIAKSVTLIHRRDT-
FRATF01216 KFM--GKGVSACATCDGF--FYKNK-DVAVVGGGNTAVEEALFLSNIAKSVTLIHRRDT-
FRATN00564 KFM--GKGVSACATCDGF--FYKNK-DVAVVGGGNTAVEEALFLSNIAKSVTLIHRRDT-
ACIF500512 KFR--GKGVSACATCDGF--FYRGQ-AVAVVGGGNTAVEEALYLSNIAKHVTVIHRRNR-
ACIF200362 KFR--GKGVSACATCDGF--FYRGQ-AVAVVGGGNTAVEEALYLSNIAKHVTVIHRRNR-
DECAR01284 TFA--GKGVSACATCDGF--FYRNK-PVAVVGGGNTAVEEALYLANIASHVTVIHRREK-
NEIG100525 AFA--GKGVSACATCDGF--FYKNQ-DVAVVGGGNTAVEEALYLANIAKTVTLIHRRSE-
NEIG201314 AFA--GKGVSACATCDGF--FYKNQ-DVAVVGGGNTAVEEALYLANIAKTVTLIHRRSE-
NEIM800926 AFA--GKGVSACATCDGF--FYKNQ-DVAVVGGGNTAVEEALYLANIAKTVTLIHRRSE-
NEIMP01199 AFA--GKGVSACATCDGF--FYKNQ-DVAVVGGGNTAVEEALYLANIAKTVTLIHRRSE-
NEIMB01212 AFA--GKGVSACATCDGF--FYKNQ-DVAVVGGGNTAVEEALYLANIAKTVTLIHRRSE-
NEIMF01143 AFA--GKGVSACATCDGF--FYKNQ-DVAVVGGGNTAVEEALYLANIAKTVTLIHRRSE-
NEIML01119 AFA--GKGVSACATCDGF--FYKNQ-DVAVVGGGNTAVEEALYLANIAKTVTLIHRRSE-
NEIMM00780 AFA--GKGVSACATCDGF--FYKNQ-DVAVVGGGNTAVEEALYLANIAKTVTLIHRRSE-
NEIMH00827 AFA--GKGVSACATCDGF--FYKNQ-DVAVVGGGNTAVEEALYLANIAKTVTLIHRRSE-
NEIMG01168 AFA--GKGVSACATCDGF--FYKNQ-DVAVVGGGNTAVEEALYLANIAKTVTLIHRRSE-
NEIMN01254 AFA--GKGVSACATCDGF--FYKNQ-DVAVVGGGNTAVEEALYLANIAKTVTLIHRRSE-
NEIMO00810 AFA--GKGVSACATCDGF--FYKNQ-DVAVVGGGNTAVEEALYLANIAKTVTLIHRRSE-
NEIM701229 AFA--GKGVSACATCDGF--FYKNQ-DVAVVGGGNTAVEEALYLANIAKTVTLIHRRSE-
NEIMA01334 AFA--GKGVSACATCDGF--FYKNQ-DVAVVGGGNTAVEEALYLANIAKTVTLIHRRSE-
NEIMW01125 AFA--GKGVSACATCDGF--FYKNQ-DVAVVGGGNTAVEEALYLANIAKTVTLIHRRSE-
DICNV01037 QYL--GYGVSACATCDGF--FYRNK-PVMVIGGGNTALEEALYLSNIASSVTLVHRRDA-
VESOH00047 EFK--GKGVSACATCDGF--FYRGK-KVAVIGGGNTAVEEALFLSNIVDHVTIVHRKHK-
RUTMC00043 KFK--GKGVSACATCDGF--FYRGQ-KVAVIGGGNTAVEEALFLSNIADHVTIIHRKDK-
HALHL02255 RFM--GKGVSACATCDGF--FYRNQ-PVAVIGGGNSAVQEALYLSNICSHVTLVHRRNN-
PELPD03177 AFL--GKGVSGCATCDGF--FYRGK-DVAVIGGGSTAVEEALYLSNIARHVTIVHRRDE-
GEOS804030 AFK--GKGVSACATCDGF--FYRGK-PVAVIGGGSTAVEEALYLSNIASHVTVVHRRDK-
GEOBB03636 AFK--GKGVSACATCDGF--FYRGK-PVVVIGGGSTAVEEALYLSNIASHVTVVHRRDK-
GEOSM03700 AFK--GKGVSACATCDGF--FYRGK-PVVVIGGGSTAVEEALYLSNIASHVTVVHRRDK-
HALNC00046 KFK--GKGVSACATCDGF--FYKNQ-EIAVVGGGNTAVEEALYLANVASKVHVIHRRDK-
HAEPS00887 AYK--GRGVSACATCDGF--FYRNK-PVAVIGGGNTAVEEALYLSNVASVVHLVHRRDT-
GALAU02285 AYK--GRGVSACATCDGF--FYRNK-PVAVIGGGNTAVEEALYLANIASTVHLVHRRDE-
HISS201000 TYK--GRGVSACATCDGF--FYRNK-PVAVVGGGNTAVEEALYLANIASEVHLIHRRDT-
HAES101151 TYK--GRGVSACATCDGF--FYRNK-PVAVVGGGNTAVEEALYLANIASEVHLIHRRDT-
PASMU00573 AYK--GRGVSACATCDGF--FYRNK-PVAVIGGGNTAVEEALYLANIASEVHLVHRRDS-
PASMH00579 AYK--GRGVSACATCDGF--FYRNK-PVAVVGGGNTAVEEALYLANIASEVHLVHRRDS-
ACTSZ01560 NFK--GRGVSACATCDGF--FYRNK-PVAVVGGGNTAVEEALYLANIAGEVHLIHRREG-
AGGAN00985 NYK--GRGVSACATCDGF--FYRNK-PVAVVGGGNAAVEEALYLANIAGEVHLIHRRDS-
NITHN03181 AYM--GRGVSACATCDGF--FYRGK-PVAVIGGGNTAVEEALYLSNMADHVTVVHRRDR-
NITOC00311 AYM--GRGVSACATCDGF--FYRGK-PVAVIGGGNTAVEEALYLSNMADHVTVIHRRDR-
NITWC02498 AYM--GRGVSACATCDGF--FYRGK-PVAVIGGGNTAVEEALYLSNMADHVTVVHRRDR-

METNJ00648	AFK--GRGVSACATCDGF--FYKNQ-PVAVIGGGNTAVEEALYLSNIASKVTVIHRRDS-
METFJ01616	AFK--GRGVSACATCDGF--FYKNQ-PVAVVGGGNTAVEEALYLSNIASKVTVIHRRDS-
ALKEH00244	AFM--GKGVSACATCDGF--FYKNQ-PVAVIGGGNTAVEEALYLSNIASHVTLVHRRDS-
MARMS03166	KFM--GQGVSACATCDGF--FYRNQ-DVAVIGGGNTAVEEALYLSNIAKTVTVIHRRDK-
MARM102688	KFM--GQGVSACATCDGF--FYRGK-DVAVIGGGNTAVEEALYLANIANSVTVVHRRDK-
THICR00763	AFK--GKGVSACATCDGF--FYRNQ-KVAVVGGGNTAVEEALYLSNIASEVTVIHRRDS-
THICA00974	AFK--GKGVSACATCDGF--FYRNQ-KVAVIGGGNTAVEEALYLSNIAAEVTIIHRRDK-
THIV600851	TFR--GRGVSACATCDGF--FYRNQ-KVAVIGGGNTAVEEALYLSNIASEVTLVHRRDA-
THISH01973	AFK--GKGVSACATCDGF--FYRNQ-KVAVIGGGNTAVEEALYLSNIASHVTLVHRRDK-
METAA00813	AFK--GKGVSACATCDGF--FYKNK-PVAVIGGGNTAVEEALYLSNIASQVTIVHRRDK-
META200813	AFK--GKGVSACATCDGF--FYKNK-PVAVIGGGNTAVEEALYLSNIASQVTIVHRRDK-
METMM02895	DFK--GRGVSACATCDGF--FYRNK-PVAVIGGGNTAVEEALYLANIASEVVVVHRRDK-
FRAAD00813	KFK--GRGVSACATCDGF--FFREQ-VVVVIGGGNTAVEEALYLSNICSKVYLVHRRDS-
XYLFA01416	AFK--GRGVSACATCDGF--FYRDQ-DVAVIGGGNTAVEEALYLSNIARKVYLIHRRDK-
XYLFT00623	AFK--GRGVSACATCDGF--FYRDQ-DVAVIGGGNTAVEEALYLSNIARKVYLIHRRDK-
XYLF200667	AFK--GRGVSACATCDGF--FYRDQ-DVAVIGGGNTAVEEALYLSNIARKVYLIHRRDK-
XYLFG01622	AFK--GRGVSACATCDGF--FYRDQ-DVAVIGGGNTAVEEALYLSNIARKVYLIHRRDK-
XYLFM00690	AFK--GRGVSACATCDGF--FYRDQ-DVAVIGGGNTAVEEALYLSNIARKVYLIHRRDK-
PSEUP01680	AFK--GRGVSACATCDGF--FYRDQ-DVAVVGGGNTAVEEALYLSNIANRVYLIHRRDT-
STRM501972	EFK--GRGVSACATCDGF--FYRDQ-DVVVVGGGNTAVEEALYLSNIARKVYLVHRRDT-
PSEUU01524	KFK--GRGVSACATCDGF--FYRDQ-DVVVVGGGNTAVEEALYLSNIARKVYLVHRRDT-
XANAP01407	AFK--GRGVSACATCDGF--FYKDQ-DVVVVGGGNTAVEEALYLSNIARKVYLVHRRDT-
XANCP01918	AFK--GRGVSACATCDGF--FYKDQ-DVVVVGGGNTAVEEALYLSNIARKVYLVHRRDT-
XANC802154	AFK--GRGVSACATCDGF--FYKDQ-DVVVVGGGNTAVEEALYLSNIARKVYLVHRRDT-
XANCB02263	AFK--GRGVSACATCDGF--FYKDQ-DVVVVGGGNTAVEEALYLSNIARKVYLVHRRDT-
XANOR02379	AFK--GRGVSACATCDGF--FYKDQ-DVVVVGGGNTAVEEALYLSNIARKVYLVHRRDT-
XANOM02295	AFK--GRGVSACATCDGF--FYKDQ-DVVVVGGGNTAVEEALYLSNIARKVYLVHRRDT-
XANOP02318	AFK--GRGVSACATCDGF--FYKDQ-DVVVVGGGNTAVEEALYLSNIARKVYLVHRRDT-
XANAC01951	AFK--GRGVSACATCDGF--FYKDQ-DVVVVGGGNTAVEEALYLSNIARKVYLVHRRDT-
CYCSP01155	AFK--GKGVSACATCDGF--FYKNQ-RVAVIGGGNTAVEEALYLSNIAEHVTVIHRRDS-
GEOLS03275	AFK--GKGVSACATCDGF--FYRNK-PVAVIGGGNTAVEEALYLANIASHVTVVHRRDQ-
GEOUR03858	AFK--GKGVSACATCDGF--FYRGK-PVAVIGGGSTAVEEALYLSNIASHVTVIHRRDQ-
GEODF00692	AFK--GKGVSACATCDGF--FYRNK-PVAVIGGGNTAVEEALYLANIASHVFVIHRRDQ-
GEOSL00482	AFK--GKGVSACATCDGF--FYRGK-PVAVIGGGNTAVEEALYLSNIASHVTLVHRRDK-
GEOSK00469	AFK--GKGVSACATCDGF--FYRGK-PVAVIGGGNTAVEEALYLSNIASHVTLVHRRDK-
GEOMG02998	AFK--GKGVSACATCDGF--FYRGK-PVAVIGGGSTAVEEALYLSNIASHVTVIHRRDK-
MORCR00228	KFM--GQGVSACATCDGF--FYKNQ-KVAVVGGGNTAVEEALYLSNIASEVTLIHRRDT-
ACIAD00798	AFM--GQGVSACATCDGF--FYKNQ-KVMVVGGGNTAVEEALYLSNIASHVTLVHRRDS-
ACIBC00821	KFM--GQGVSACATCDGF--FYKNQ-NVMVVGGGNTAVEEALYLSNIAEHVTLVHRRDS-
ACIBY02725	KFM--GQGVSACATCDGF--FYKNQ-NVMVVGGGNTAVEEALYLSNIAEHVTLVHRRDS-
ACIB302691	KFM--GQGVSACATCDGF--FYKNQ-NVMVVGGGNTAVEEALYLSNIAEHVTLVHRRDS-
ACIB500882	KFM--GQGVSACATCDGF--FYKNQ-NVMVVGGGNTAVEEALYLSNIAEHVTLVHRRDS-
ACIB100850	KFM--GQGVSACATCDGF--FYKNQ-NVMVVGGGNTAVEEALYLSNIAEHVTLVHRRDS-
ACIBD00843	KFM--GQGVSACATCDGF--FYKNQ-NVMVVGGGNTAVEEALYLSNIAEHVTLVHRRDS-
ACIBS02229	KFM--GQGVSACATCDGF--FYKNQ-NVMVVGGGNTAVEEALYLSNIAEHVTLVHRRDS-
ACICP00137	NFM--GQGVSACATCDGF--FYKNQ-KVMVVGGGNTAVEEALYLSNIASHVTLVHRRDT-
ACISD03052	NFM--GQGVSACATCDGF--FYKNQ-KVMVVGGGNTAVEEALYLSNIASHVTLVHRRDS-
LEGLN02402	AYQ--GRGVSACATCDGF--FYRNK-AVCVIGGGNTAVEEALYLSNLASSVTLVHRRDS-
LEGPA01726	AYQ--GRGVSACATCDGF--FYRNK-SVCVIGGGNTAVEEALYLSNLASSVTLIHRRDS-
LEGPH00846	AYQ--GRGVSACATCDGF--FYRNK-SVCVIGGGNTAVEEALYLSNLASSVTLIHRRDS-
LEGPC01171	AYQ--GRGVSACATCDGF--FYRNK-SVCVIGGGNTAVEEALYLSNLASSVTLIHRRDS-
LEGP201857	AYQ--GRGVSACATCDGF--FYRNK-SVCVIGGGNTAVEEALYLSNLASSVTLIHRRDS-
LEGPL01727	AYQ--GRGVSACATCDGF--FYRNK-SVCVIGGGNTAVEEALYLSNLASSVTLIHRRDS-
COLP302668	AFM--GRGVSACATCDGF--FYKNQ-KVAVVGGGNTAVEEALYLSNIASEVHLIHRRDT-
KANKD01106	AFM--GKGVSACATCDGF--FYRQQ-KVAVIGGGNTAVEEALYLSNIASEVHLVHRRDE-
IDILO00659	AFM--GKGVSACATCDGF--FYKKQ-KVCVVGGGNTAVEEALYLSNIASEVHVIHRRDT-
PSEU901284	NFQ--GRGVSACATCDGF--FYKGQ-KVAVVGGGNTAVEEALYLSNIADEVHVIHRRDS-
PSEA602346	AFM--GKGVSACATCDGF--FYRNQ-KVAVIGGGNTAVEEALYLSNIASEVHVIHRRDT-
ALTSS01871	AFM--GKGVSACATCDGF--FYRNQ-KVAVIGGGNTAVEEALYLSNIASEVHVVHRRDT-
ALTMD01611	AFM--GKGVSACATCDGF--FYRNQ-KVAVVGGGNTAVEEALYLSNIASEVHVIHRRDS-
ALTME01705	AFM--GKGVSACATCDGF--FYRNQ-KVAVIGGGNTAVEEALYLSNIASEVHVIHRRDS-
ALTMB01797	AFM--GKGVSACATCDGF--FYRNQ-KVAVIGGGNTAVEEALYLSNIASEVHVIHRRDS-
ALTMS01652	AFM--GKGVSACATCDGF--FYRNQ-KVAVIGGGNTAVEEALYLSNIASEVHVIHRRDS-
SACD201683	AFM--GKGVSACATCDGF--FYRDK-KVIVVGGGNTAVEEALYLSNIASEVTLVHRRDS-
TERTT01748	AFQ--GKGVSACATCDGF--FYRDQ-KVAVVGGGNTAVEEALYLANIASEVTLIHRRDS-

```
SIMAS00609      AYM--GRGVSACATCDGF--FYKDQ-KVVVVGGGNTAVEEALYLSNIASEVTLIHRRDT-
ALCDB01962      AFM--GRGVSACATCDGF--FYRGQ-KVAVVGGGNTAVEEALYLSNIASEVVLIHRRDK-
CHRSD02939      KFM--GQGVSACATCDGF--FYRGQ-EVVVVGGGNTAVEEALYLSNIASKVTLVHRRDS-
HALED02811      RFM--GQGVSACATCDGF--FYRNQ-DVVVVGGGNTAVEEALYLSNIASKVTLVHRRDS-
SIDLE02655      AFM--GKGVSGCATCDGF--FYRNQ-DVAVIGGGNTAVEEALYLANIARHVTLVHRRDS-
GALCS01621      AFM--GRGVSGCATCDGF--FYRGQ-NVAVIGGGNTAVEEALYLANIAKHVTLVHRRDT-
LARHH01446      AFM--GRGVSGCATCDGF--FYKNQ-DVAVVGGGNTAVEEALYLANIARHVTLIHRREA-
CHRVO02807      AFA--GKGVSACATCDGF--FYRNQ-DVAVVGGGNTAVEEALYLANIARHVTLIHRRDT-
PSEUL01648      AFM--GKGVSACATCDGF--FYRNQ-DVAVIGGGNTAVEEALYLANIAKHVTLVHRRDS-
NITEU01859      AFM--GKGVSACATCDGF--FYKNQ-DVAVIGGGNTAVEEALYLSNIARKVTVVHRRDK-
NITEC00745      AFM--GKGVSACATCDGF--FYKNQ-DVAVIGGGNTAVEEALYLSNIARKVTVVHRRDK-
NITMU00023      AYM--GKGVSACATCDGF--FYKGQ-DVAVIGGGNTAVEEALYLSNIARSVTVVHRRDK-
NITSI03064      AFM--GRGVSGCATCDGF--FYKGQ-DVAVIGGGNTAVEEALYLANIARSVTVVHRREQ-
ACCPU02128      AYS--GRGVSACATCDGF--FYRNQ-PVAVIGGGNTAVEEALYLANIASHVTLVHRRDK-
THIDA01000      AFM--GKGVSGCATCDGF--FYKGQ-DVAVIGGGNSAVEEALYLANIARHVTLVHRRDT-
METS601221      KFS--GKGVSACATCDGF--FYRNQ-EVAVIGGGNTAVEEALYLANIASKVTLVHRRDK-
METGS01186      QFS--GKGVSACATCDGF--FYRNQ-EVAVIGGGNTAVEEALYLANIASKVTLVHRRDK-
METFK00972      AFA--GKGVSACATCDGF--FYRNQ-EVAVIGGGNTAVEEALYLANIASKVTLVHRRDK-
METML01428      AFS--GKGVSACATCDGF--FYRNQ-EVAVIGGGNTAVEEALYLANIASKVTLVHRRDK-
AROAE03899      KFS--GRGVSACATCDGF--FYKNQ-DVAVIGGGNTAVEEALYLANIAKKVTVVHRREK-
THASP01686      KFA--GRGVSACATCDGF--FYRNQ-DVAVIGGGNTAVEEALYLANIARKVTLVHRREK-
AZOSB01359      KFA--GRGVSACATCDGF--FYRNQ-DVAVIGGGNTAVEEALYLANIAKKVTVVHRREK-
BORA102682      KFM--GRGVSGCATCDGF--FYRNQ-DVVVIGGGNTAVEEALYLSNICRKVTLIHRRDK-
BORPA03415      AFM--GRGVSGCATCDGF--LYRNK-DVVVIGGGNTAVEEALYLSNICRKVTLIHRRDK-
BORBM03577      AFM--GRGVSGCATCDGF--FYRNK-DVVVIGGGNTAVEEALYLSNICRKVTLIHRRDK-
BORPE02280      AFM--GRGVSGCATCDGF--FYRNK-DVVVIGGGNTAVEEALYLSNICRKVTLIHRRDK-
BORPC02060      AFM--GRGVSGCATCDGF--FYRNK-DVVVIGGGNTAVEEALYLSNICRKVTLIHRRDK-
BORP102392      AFM--GRGVSGCATCDGF--FYRNK-DVVVIGGGNTAVEEALYLSNICRKVTLIHRRDK-
BORBR03870      AFM--GRGVSGCATCDGF--FYRNK-DVVVIGGGNTAVEEALYLSNICRKVTLIHRRDK-
BORPD01524      AFM--GRGVSGCATCDGF--FYKNQ-DVVVVGGGNTAVEEALYLSNICRKVTLIHRRDK-
ACHXA01179      AFM--GRGVSGCATCDGF--FYRNQ-DVVVVGGGNTAVEEALYLSNICRKVTLIHRRDK-
RHOFT03114      AFM--GRGVSGCATCDGF--FYRDQ-DVCVVGGGNTAVEEALYLSNIASKVYLIHRRDK-
VEREI01701      AFM--GKGVSGCATCDGF--FYRGQ-DVCVVGGGNTAVQEALYLSNIARKVTLVHRRDR-
VARPE01375      KFM--GRGVSACATCDGF--FYREQ-EVCVIGGGNTAVEEALYLANIANKVTLVHRRDK-
VARPS01266      KFM--GRGVSACATCDGF--FYREQ-EVCVIGGGNTAVEEALYLANIANKVTLVHRRDK-
DELAS05272      AFM--GRGVSGCATCDGF--FYREQ-PVCVVGGGNTAVEEALYLSNIASKVTLVHRRDK-
DELSC01235      AFM--GRGVSGCATCDGF--FYREQ-PVCVVGGGNTAVEEALYLSNIASKVTLVHRRDK-
COMT200883      AFM--GRGVSACATCDGF--FYREQ-PVCVIGGGNTAVEEALYLSNIASKVTLVHRRDK-
ACIAC03267      AFM--GRGVSGCATCDGF--FYREQ-DVCVVGGGNTAVEEALYLSNIARKVTLVHRRDK-
ACIAP03249      AFM--GRGVSGCATCDGF--FYREQ-DVCVVGGGNTAVEEALYLSNIARKVTLVHRRDK-
ACIET02648      AFM--GKGVSACATCDGF--FYREQ-DVCVVGGGNTAVEEALYLSNIARKVTLVHRRDK-
ALIDK03528      AFM--GRGVSACATCDGF--FYRDQ-DVCVIGGGNTAVEEALYLANIARKVTLVHRRDK-
RUBGI03408      AFM--GRGVSGCATCDGF--FYRDQ-AVCVVGGGNTAVEEALYLSNIASTVHLIHRRDK-
LEPCP00749      AFM--GRGVSGCATCDGF--FYRGD-VVCVVGGGNTAVEEALYLSNIASKVHLIHRRDK-
POLSJ03719      AFM--GRGVSGCATCDGF--FYRNQ-DVCVVGGGNTAVEEALYLSNIASKVTLVHRRDK-
POLNA03144      AFM--GRGVSGCATCDGF--FYRQQ-EVCVVGGGNTAVEEALYLSNIASKVVLVHRRDT-
METPP01110      AFM--GRGVSGCATCDGF--FYRDQ-EVCVIGGGNTAVEEALYLSNIASKVTLVHRRDK-
RAMTT03218      AFM--GKGVSACATCDGF--FYRGE-VTCVIGGGNTAVEEALYLSNIASKVYLVHRRDK-
RALPJ02457      AFA--GRGVSACATCDGF--FYKGQ-EVAVVGGGNTAVEEALYLANIASKVTLIHRRDK-
RALP102102      AFA--GRGVSACATCDGF--FYKGQ-EVAVVGGGNTAVEEALYLANIASKVTLIHRRDK-
RALSO02303      TFS--GRGVSACATCDGF--FYKGQ-EVAVVGGGNTAVEEALYLANIASKVTLIHRRDK-
RALS801097      TFS--GRGVSACATCDGF--FYKGQ-EVAVVGGGNTAVEEALYLANIATKVTLIHRRDK-
HERSS01878      AFM--GRGVSACATCDGF--FYRGK-EVAVVGGGNTAVEEALYLSNIASKVTIIHRRDK-
HERAR00955      AYM--GKGVSACATCDGF--FYREQ-EVAVIGGGNTAVEEALYLSNIATKVTLVHRRDK-
JANMA01152      AFM--GKGVSACATCDGF--FYRNQ-EVAVIGGGNTAVEEALYLSNIANKVTVVHRRDK-
THIK102324      AFM--GKGVSGCATCDGF--FYRNQ-PVCVVGGGNTAVEEALYLANIASKVTVIHRRDK-
POLSQ00682      EFM--GRGVSGCATCDGF--FYRNQ-DVCVVGGGNTAVEEALYLTGIAKKVTVIHRRDK-
POLNS00989      AFM--GHGVSGCATCDGF--FYRNQ-DVCVVGGGNTAVEEALYLTGIAKKVTVIHRRDK-
BURP800627      LFM--GRGVSACATCDGF--FYKNQ-HVAVVGGGNTAVEEALYLAGIARKVTVIHRRDK-
BURPP00988      AFM--GKGVSACATCDGF--FYKQQ-HVAVIGGGNTAVEEALYLSGIARKVTVIHRRDK-
BURSC00728      AFM--GKGVSACATCDGF--FYKQQ-HVAVIGGGNTAVEEALYLAGIAKKVTVIHRRDK-
BURXL00982      AFM--GKGVSACATCDGF--FYKQQ-HVAVIGGGNTAVEEALYLAGIAKKVTVIHRRDK-
BURSG00869      AFM--GKGVSACATCDGF--FYKQQ-HVAVVGGGNTAVEEALYLSGIAKKVTVIHRRDK-
BURRH00629      QFM--GKGVSACATCDGF--FYRNQ-DVAVIGGGNTAVEEALYLSGIAKKVTVIHRRDK-
BURGB00781      AFM--GKGVSACATCDGF--FYRNQ-DVAVIGGGNTAVEEALYLTGIAKKVTVIHRRDK-
```

```
BURGS00853      AFM--GKGVSACATCDGF--FYRNQ-EVAVIGGGNTAVEEALYLTGIAKKVTVIHRRDK-
BURPS02618      AFM--GRGVSACATCDGF--FYRGQ-NVAVVGGGNTAVEEALYLTGIAKKVTVIHRRDK-
BURMA01741      AFM--GRGVSACATCDGF--FYRGQ-NVAVVGGGNTAVEEALYLTGIAKKVTVIHRRDK-
BURP103016      AFM--GRGVSACATCDGF--FYRGQ-NVAVVGGGNTAVEEALYLTGIAKKVTVIHRRDK-
BURP002984      AFM--GRGVSACATCDGF--FYRGQ-NVAVVGGGNTAVEEALYLTGIAKKVTVIHRRDK-
BURM701866      AFM--GRGVSACATCDGF--FYRGQ-NVAVVGGGNTAVEEALYLTGIAKKVTVIHRRDK-
BURP602945      AFM--GRGVSACATCDGF--FYRGQ-NVAVVGGGNTAVEEALYLTGIAKKVTVIHRRDK-
BURM902465      AFM--GRGVSACATCDGF--FYRGQ-NVAVVGGGNTAVEEALYLTGIAKKVTVIHRRDK-
BURMS00740      AFM--GRGVSACATCDGF--FYRGQ-NVAVVGGGNTAVEEALYLTGIAKKVTVIHRRDK-
BURTA01515      AFM--GRGVSACATCDGF--FYRGQ-NVAVVGGGNTAVEEALYLTGIAKKVTVIHRRDK-
BURM102386      AFM--GKGVSACATCDGF--FYRGQ-DVAVIGGGNTAVEEALYLTGIAKKVTVIHRRDK-
BURL300890      LFM--GKGVSACATCDGF--FYRGQ-EVAVIGGGNTAVEEALYLTGIAKKVTVIHRRDK-
BURVG00860      AFM--GKGVSACATCDGF--FYRNQ-EVAVIGGGNTAVEEALYLTGIAKKVTVIHRRDK-
BURCM00829      AFM--GKGVSACATCDGF--FYRNQ-EVAVIGGGNTAVEEALYLTGIAKKVTVIHRRDK-
BURA400832      AFM--GKGVSACATCDGF--FYRNQ-EVAVIGGGNTAVEEALYLTGIAKKVTVIHRRDK-
BURCA00489      AFM--GKGVSACATCDGF--FYRNQ-EVAVIGGGNTAVEEALYLTGIAKKVTVIHRRDK-
BURCH00966      AFM--GKGVSACATCDGF--FYRNQ-EVAVIGGGNTAVEEALYLTGIAKKVTVIHRRDK-
BURCC00927      AFM--GKGVSACATCDGF--FYRNQ-EVAVIGGGNTAVEEALYLTGIAKKVTVIHRRDK-
BURCJ02922      AFM--GKGVSACATCDGF--FYRNQ-EVAVIGGGNTAVEEALYLTGIAKKVTVIHRRDK-
EDWI902348      AFK--GRGVSACATCDGF--FYRQQ-EIAVIGGGNTAVEEALYLANIAAKVHLIHRRDS-
EDWTF01971      AFK--GRGVSACATCDGF--FYRQQ-EIAVIGGGNTAVEEALYLANIAAKVHLIHRRDS-
EDWTE02176      AFK--GRGVSACATCDGF--FYRQQ-EIAVIGGGNTAVEEALYLANIAAKVHLIHRRDS-
SODGM01094      AYK--GKGVSACATCDGF--FYRNQ-KVAVVGGGNTAVEEALYLANIAEEVHLIHRRDT-
MOREP00394      TYK--GKGVSACATCDGF--FYCNQ-KVAVVGGGNTAVEEALYLANIAKEVHVIHRRDT-
RAHSY01432      AFK--GRGVSACATCDGF--FYRNQ-KVAVVGGGNTAVEEALYLANIASEVHLIHRRDN-
RAHAC01419      AFK--GRGVSACATCDGF--FYRNQ-KVAVVGGGNTAVEEALYLANIASEVHLIHRRDN-
ERWBE01489      AFK--GRGVSACATCDGF--FYRNQ-KVAVIGGGNTAVEEALYLANIAAEVHLIHRRDS-
PANAM01339      AFK--GKGVSACATCDGF--FYRNQ-KVAVIGGGNTAVEEALYLANIAAEVHLIHRRDS-
PANAA00662      AFK--GKGVSACATCDGF--FYRNQ-KVAVIGGGNTAVEEALYLANIAAEVHLIHRRDS-
PANSA01303      AFK--GKGVSACATCDGF--FYRNQ-KVAVIGGGNTAVEEALYLANIAAEVHLIHRRDS-
ERWT902154      EFK--GRGVSACATCDGF--FYRNQ-KVAVIGGGNTAVEEALYLANIAAEVHLIHRRDS-
ERWAC01330      AFK--GRGVSACATCDGF--FYRNQ-KVAVIGGGNTAVEEALYLANIAAEVHLIHRRDS-
ERWAE01326      AFK--GRGVSACATCDGF--FYRNQ-KVAVIGGGNTAVEEALYLANIAAEVHLIHRRDS-
ERWPE02241      EFK--GRGVSACATCDGF--FYRNQ-KVAVIGGGNTAVEEALYLANIAAEVHLIHRRDS-
ERWP602414      EFK--GRGVSACATCDGF--FYRNQ-KVAVIGGGNTAVEEALYLANIAAEVHLIHRRDS-
ERWSE02396      EFK--GRGVSACATCDGF--FYRNQ-KVAVIGGGNTAVEEALYLANIAAEVHLIHRRDS-
PECCP01694      AFK--GKGVSACATCDGF--FYRNQ-KVAVVGGGNTAVEEALYLSNIAAEVHLIHRRET-
PECWW01889      AFK--GKGVSACATCDGF--FYRNQ-KVAVVGGGNTAVEEALYLSNIAAEVHLIHRRET-
PECSS01870      AFK--GKGVSACATCDGF--FYRNQ-KVAVVGGGNTAVEEALYLSNIAAEVHLIHRRET-
PECAS02624      AFK--GKGVSACATCDGF--FYRNQ-KVAVVGGGNTAVEEALYLSNIAAEVHLIHRRET-
DICDC02220      AFK--GKGVSACATCDGF--FYRQQ-KVAVVGGGNTAVEEALYLSNIAAEVHLIHRRDS-
DICZE02284      AFK--GKGVSACATCDGF--FYRNQ-KVAVVGGGNTAVEEALYLSNIAAEVHLIHRRDS-
DICD302017      AFK--GKGVSACATCDGF--FYRNQ-KVAVVGGGNTAVEEALYLSNIAAEVHLIHRRDS-
DICD502250      AFK--GKGVSACATCDGF--FYRNQ-KVAVVGGGNTAVEEALYLSNIAAEVHLIHRRDS-
XENBS00829      AFK--GRGVSACATCDGF--FYRKQ-KVAVVGGGNTAVEEALYLANIAAEVHLIHRRDT-
XENNA01480      AFK--GRGVSACATCDGF--FYRKQ-KVAVVGGGNTAVEEALYLANIASEVHLIHRRDT-
PHOLL01537      AFK--GRGISACATCDGF--FYRNQ-KVAVVGGGNTAVEEALYLANIAAEVHLIHRRDT-
PHOAA02804      AFK--GRGISACATCDGF--FYRNQ-KVAVVGGGNTAVEEALYLANIASEVHLIHRRDT-
SERP501669      AFK--GKGVSACATCDGF--FYRNQ-KVAVVGGGNTAVEEALYLSNIAAEVHLIHRRDS-
SERSA01625      AFK--GKGVSACATCDGF--FYRNQ-KVAVVGGGNTAVEEALYLSNIAAEVHLIHRRDS-
YERPE01271      AFK--GKGVSACATCDGF--FYRNQ-KVAVVGGGNTAVEEALYLANIAAEVHLIHRRDT-
YERPS01390      AFK--GKGVSACATCDGF--FYRNQ-KVAVVGGGNTAVEEALYLANIAAEVHLIHRRDT-
YERPA00627      AFK--GKGVSACATCDGF--FYRNQ-KVAVVGGGNTAVEEALYLANIAAEVHLIHRRDT-
YERPN02489      AFK--GKGVSACATCDGF--FYRNQ-KVAVVGGGNTAVEEALYLANIAAEVHLIHRRDT-
YERPP02190      AFK--GKGVSACATCDGF--FYRNQ-KVAVVGGGNTAVEEALYLANIAAEVHLIHRRDT-
YERP302544      AFK--GKGVSACATCDGF--FYRNQ-KVAVVGGGNTAVEEALYLANIAAEVHLIHRRDT-
YERPB01460      AFK--GKGVSACATCDGF--FYRNQ-KVAVVGGGNTAVEEALYLANIAAEVHLIHRRDT-
YERPY02633      AFK--GKGVSACATCDGF--FYRNQ-KVAVVGGGNTAVEEALYLANIAAEVHLIHRRDT-
YERPG01392      AFK--GKGVSACATCDGF--FYRNQ-KVAVVGGGNTAVEEALYLANIAAEVHLIHRRDT-
YERPD01171      AFK--GKGVSACATCDGF--FYRNQ-KVAVVGGGNTAVEEALYLANIAAEVHLIHRRDT-
YERP100890      AFK--GKGVSACATCDGF--FYRNQ-KVAVVGGGNTAVEEALYLANIAAEVHLIHRRDT-
YERPZ01206      AFK--GKGVSACATCDGF--FYRNQ-KVAVVGGGNTAVEEALYLANIAAEVHLIHRRDT-
YERPH02469      AFK--GKGVSACATCDGF--FYRNQ-KVAVVGGGNTAVEEALYLANIAAEVHLIHRRDT-
YERE801438      AFK--GKGVSACATCDGF--FYRNQ-KVAVVGGGNTAVEEALYLANIAAEVHLIHRRDS-
YERE302565      AFK--GKGVSACATCDGF--FYRNQ-KVAVVGGGNTAVEEALYLANIAAEVHLIHRRDS-
```

```
YERE100392      AFK--GKGVSACATCDGF--FYRNQ-KVAVVGGGNTAVEEALYLANIAAEVHLIHRRDS-
PROMH00687      AFK--GRGVSACATCDGF--FYRNQ-KVAVVGGGNTAVEEALYLANIASEVHLIHRRDS-
PROSM03196      AFK--GRGVSACATCDGF--FYRNQ-KVVVVGGGNTAVEEALYLSNIASEVHLVHRRDS-
TOLAT02279      AFK--GRGVSACATCDGF--FYRGQ-KVAVVGGGNTAVEEALYLANIASEVHLIHRRDT-
AERVB02262      AFK--GRGVSACATCDGF--FYRNQ-EVAVIGGGNTAVEEALYLANIAKKVHLIHRRDE-
AERHH01811      AFK--GRGVSACATCDGF--FYRNQ-EVAVVGGGNTAVEEALYLANIAKKVHLIHRRDE-
AERS402185      AFK--GRGVSACATCDGF--FYRNQ-EVAVIGGGNTAVEEALYLANIAKKVHLIHRRDE-
PSYIN02070      AFK--GRGVSACATCDGF--FYKNQ-KVAVVGGGNTAVEEALYLSNIASEVHLIHRRTG-
SHELP02018      AFK--GKGVSACATCDGF--FYRNQ-KVAVIGGGNTAVEEALYLSNIASEVHLIHRRDS-
SHEVD02297      AFK--GKGVSACATCDGF--FYRNQ-KVAVVGGGNTAVEEALYLSNIASEVHLIHRRDS-
SHEPW02341      AFK--GRGVSACATCDGF--FYRNQ-KVAVIGGGNTAVEEALYLSNIASEVHLIHRRDT-
SHEPA02202      AFK--GRGVSACATCDGF--FYRNQ-KVAVIGGGNTAVEEALYLSNIASEVHLIHRRDS-
SHEHH02007      AFK--GRGVSACATCDGF--FYRNQ-KVAVIGGGNTAVEEALYLSNIASEVHLIHRRDS-
SHESH02117      AFK--GKGVSACATCDGF--FYRNQ-KVAVIGGGNTAVEEALYLSNIASEVHLIHRRDA-
SHEWM02454      EFK--GKGVSACATCDGF--FYRNQ-KVAVIGGGNTAVEEALYLSNIASEVHLIHRRDS-
SHEAM01758      AFK--GRGVSACATCDGF--FYRNQ-KVAVIGGGNTAVEEALYLSNIASEVHLIHRRDS-
SHEON02159      AFK--GRGVSACATCDGF--FYRNQ-KVAVIGGGNTAVEEALYLSNIAAEVHLIHRRDT-
SHESM01945      AFK--GRGVSACATCDGF--FYRNQ-KVAVIGGGNTAVEEALYLSNIAAEVHLIHRRDT-
SHESR01991      AFK--GRGVSACATCDGF--FYRNQ-KVAVIGGGNTAVEEALYLSNIAAEVHLIHRRDT-
SHESA02025      AFK--GRGVSACATCDGF--FYRNQ-KVAVIGGGNTAVEEALYLSNIAAEVHLIHRRDT-
SHESW01953      AFK--GRGVSACATCDGF--FYRNQ-KVAVIGGGNTAVEEALYLSNIAAEVHLIHRRDT-
SHEPC01976      AFK--GRGVSACATCDGF--FYRNQ-KVAVIGGGNTAVEEALYLSNIAAEVHLIHRRDT-
SHEP201929      AFK--GRGVSACATCDGF--FYRNQ-KVAVIGGGNTAVEEALYLSNIAAEVHLIHRRDT-
SHEB502016      AFK--GRGVSACATCDGF--FYRNQ-KVAVIGGGNTAVEEALYLSNIAAEVHLIHRRDT-
SHEB802168      AFK--GRGVSACATCDGF--FYRNQ-KVAVIGGGNTAVEEALYLSNIAAEVHLIHRRDT-
SHEB202117      AFK--GRGVSACATCDGF--FYRNQ-KVAVIGGGNTAVEEALYLSNIAAEVHLIHRRDT-
SHEB902270      AFK--GRGVSACATCDGF--FYRNQ-KVAVIGGGNTAVEEALYLSNIAAEVHLIHRRDT-
SHEB602258      AFK--GRGVSACATCDGF--FYRNQ-KVAVIGGGNTAVEEALYLSNIAAEVHLIHRRDT-
SHEDO01680      AFM--GRGVSACATCDGF--FYRNQ-KVAVVGGGNTAVEEALYLSNIASEVHLIHRRDS-
SHEFN01895      AFM--GRGVSACATCDGF--FYRNQ-KVAVIGGGNTAVEEALYLSNIASEVHLIHRRDS-
PSEHT01677      NFQ--GRGVSACATCDGF--FYKGQ-KVAVVGGGNTAVEEALYLSNIADEVHVIHRRDS-
ALISL01611      AFK--GRGVSACATCDGF--FYRNQ-KVAVIGGGNTAVEEALYLSNIAAEVHLIHRRDT-
VIBF100893      AFK--GRGVSACATCDGF--FYRNQ-KVAVVGGGNTAVEEALYLSNIAAEVHLIHRRDS-
VIBFM00891      AFK--GRGVSACATCDGF--FYRNQ-KVAVVGGGNTAVEEALYLSNIAAEVHLIHRRDS-
OCESG01399      AFQ--GRGVSACATCDGF--FYRNQ-KVAVVGGGNTAVEEALYLSNIAAEVHLIHRRDQ-
VIBA701872      TFK--GRGVSACATCDGF--FYRNQ-KVAVVGGGNTAVEEALYLSNIAAQVHLIHRRDS-
VIBVY01451      AFK--GRGVSACATCDGF--FYRNQ-KVAVVGGGNTAVEEALYLSNIAAEVHLIHRRDS-
VIBVU02545      AFK--GRGVSACATCDGF--FYRNQ-KVAVVGGGNTAVEEALYLSNIAAEVHLIHRRDS-
VIBVM01748      AFK--GRGVSACATCDGF--FYRNQ-KVAVVGGGNTAVEEALYLSNIAAEVHLIHRRDS-
VIBCH01157      AFK--GRGVSACATCDGF--FYRNQ-KVAVVGGGNTAVEEALYLSNIASEVHLVHRRDS-
VIBCM01114      AFK--GRGVSACATCDGF--FYRNQ-KVAVVGGGNTAVEEALYLSNIASEVHLVHRRDS-
VIBCJ02064      AFK--GRGVSACATCDGF--FYRNQ-KVAVVGGGNTAVEEALYLSNIASEVHLVHRRDS-
VIBC300725      AFK--GRGVSACATCDGF--FYRNQ-KVAVVGGGNTAVEEALYLSNIASEVHLIHRRDS-
FERBD01969      AFQ--GRGVSACATCDGF--FYRNK-PVAVVGGGNTAVEEALYLSNIASEVHLIHRRDS-
VIBFN02085      AFK--GRGVSACATCDGF--FYRNQ-KVAVVGGGNTAVEEALYLSNIAAEVHLIHRRDS-
VIBTL01089      AFK--GRGVSACATCDGF--FYRNQ-KVAVVGGGNTAVEEALYLSNIASEVHLVHRRDT-
VIBPA01248      TFK--GRGVSACATCDGF--FYRNQ-KVAVVGGGNTAVEEALYLSNIASEVHLIHRRDS-
VIBAE02128      AFK--GRGVSACATCDGF--FYRNQ-KVAVVGGGNTAVEEALYLSNIASEVHLIHRRDS-
VIBCB00599      AFK--GRGVSACATCDGF--FYRNQ-NVAVVGGGNTAVEEALYLSNIASEVHLIHRRDS-
PANVC00701      AFK--GKGVSACATCDGF--FYRNQ-KVAVIGGGNTAVEEALYLANIAAEVHLIHRRDS-
SHIBC02443      AFK--GRGVSACATCDGF--FYRNQ-KVAVIGGGNTAVEEALYLSNIASEVHLIHRRDS-
ENTBF02792      AFK--GRGVSACATCDGF--FYRNQ-KVAVIGGGNTAVEEALYLSNIAAEVHLIHRRDS-
KLEP700892      AFK--GRGVSACATCDGF--FYRNQ-KVAVIGGGNTAVEEALYLSNIASEVHLIHRRDS-
KLEPH01782      AFK--GRGVSACATCDGF--FYRNQ-KVAVIGGGNTAVEEALYLSNIASEVHLIHRRDS-
KLEP303551      AFK--GRGVSACATCDGF--FYRNQ-KVAVIGGGNTAVEEALYLSNIASEVHLIHRRDA-
KLEVT03420      AFK--GRGVSACATCDGF--FYRNQ-KVAVIGGGNTAVEEALYLSNIASEVHLIHRRDA-
ENTAK02985      AFK--GRGVSACATCDGF--FYRNQ-KVAVIGGGNTAVEEALYLSNIAAEVHLIHRRDT-
KLEOK03151      AFK--GRGVSACATCDGF--FYRNQ-KVAVIGGGNTAVEEALYLSNIASEVHLIHRRDG-
SALAR01925      AFK--GRGVSACATCDGF--FYRNQ-KVAVIGGGNTAVEEALYLSNIASEVHLIHRRDG-
SALBC00812      AFK--GRGVSACATCDGF--FYRNQ-KVAVIGGGNTAVEEALYLSNIASEVHLIHRRDG-
SALPC00926      AFK--GRGVSACATCDGF--FYRNQ-KVAVIGGGNTAVEEALYLSNIASEVHLIHRRDG-
SALTI00868      AFK--GRGVSACATCDGF--FYRNQ-KVAVIGGGNTAVEEALYLSNIASEVHLIHRRDG-
SALCH00905      AFK--GRGVSACATCDGF--FYRNQ-KVAVIGGGNTAVEEALYLSNIASEVHLIHRRDG-
SALPA01706      AFK--GRGVSACATCDGF--FYRNQ-KVAVIGGGNTAVEEALYLSNIASEVHLIHRRDG-
SALTY00927      AFK--GRGVSACATCDGF--FYRNQ-KVAVIGGGNTAVEEALYLSNIASEVHLIHRRDG-
```

SALPK01786 AFK--GRGVSACATCDGF--FYRNQ-KVAVIGGGNTAVEEALYLSNIASEVHLIHRRDG-
SALHS00978 AFK--GRGVSACATCDGF--FYRNQ-KVAVIGGGNTAVEEALYLSNIASEVHLIHRRDG-
SALEP00854 AFK--GRGVSACATCDGF--FYRNQ-KVAVIGGGNTAVEEALYLSNIASEVHLIHRRDG-
SALDC00926 AFK--GRGVSACATCDGF--FYRNQ-KVAVIGGGNTAVEEALYLSNIASEVHLIHRRDG-
SALA400892 AFK--GRGVSACATCDGF--FYRNQ-KVAVIGGGNTAVEEALYLSNIASEVHLIHRRDG-
SALG200876 AFK--GRGVSACATCDGF--FYRNQ-KVAVIGGGNTAVEEALYLSNIASEVHLIHRRDG-
SALTS00888 AFK--GRGVSACATCDGF--FYRNQ-KVAVIGGGNTAVEEALYLSNIASEVHLIHRRDG-
SALT400905 AFK--GRGVSACATCDGF--FYRNQ-KVAVIGGGNTAVEEALYLSNIASEVHLIHRRDG-
SALPS01908 AFK--GRGVSACATCDGF--FYRNQ-KVAVIGGGNTAVEEALYLSNIASEVHLIHRRDG-
SALT101040 AFK--GRGVSACATCDGF--FYRNQ-KVAVIGGGNTAVEEALYLSNIASEVHLIHRRDG-
SALTD00960 AFK--GRGVSACATCDGF--FYRNQ-KVAVIGGGNTAVEEALYLSNIASEVHLIHRRDG-
SALPB02472 AFK--GRGVSACATCDGF--FYRNQ-KVAVIGGGNTAVEEALYLSNIASEVHLIHRRDG-
SALNS00928 AFK--GRGVSACATCDGF--FYRNQ-KVAVIGGGNTAVEEALYLSNIASEVHLIHRRDG-
SALSV01000 AFK--GRGVSACATCDGF--FYRNQ-KVAVIGGGNTAVEEALYLSNIASEVHLIHRRDG-
ECOS500812 AFK--GRGVSACATCDGF--FYRNQ-KVAVIGGGNTAVEEALYLSNIASEVHLIHRRDG-
ECOL600981 AFK--GRGVSACATCDGF--FYRNQ-KVAVIGGGNTAVEEALYLSNIASEVHLIHRRDG-
ECOL500883 AFK--GRGVSACATCDGF--FYRNQ-KVAVIGGGNTAVEEALYLSNIASEVHLIHRRDG-
ECOUT00876 AFK--GRGVSACATCDGF--FYRNQ-KVAVIGGGNTAVEEALYLSNIASEVHLIHRRDG-
ECOK100776 AFK--GRGVSACATCDGF--FYRNQ-KVAVIGGGNTAVEEALYLSNIASEVHLIHRRDG-
ECOSM02148 AFK--GRGVSACATCDGF--FYRNQ-KVAVIGGGNTAVEEALYLSNIASEVHLIHRRDG-
ECOLU01057 AFK--GRGVSACATCDGF--FYRNQ-KVAVIGGGNTAVEEALYLSNIASEVHLIHRRDG-
ECO7I02136 AFK--GRGVSACATCDGF--FYRNQ-KVAVIGGGNTAVEEALYLSNIASEVHLIHRRDG-
ECO8100828 AFK--GRGVSACATCDGF--FYRNQ-KVAVIGGGNTAVEEALYLSNIASEVHLIHRRDG-
ECO4500862 AFK--GRGVSACATCDGF--FYRNQ-KVAVIGGGNTAVEEALYLSNIASEVHLIHRRDG-
ECOAB00879 AFK--GRGVSACATCDGF--FYRNQ-KVAVIGGGNTAVEEALYLSNIASEVHLIHRRDG-
ECO4400961 AFK--GRGVSACATCDGF--FYRNQ-KVAVIGGGNTAVEEALYLSNIASEVHLIHRRDG-
ECOUM02550 AFK--GRGVSACATCDGF--FYRNQ-KVAVIGGGNTAVEEALYLSNIASEVHLIHRRDG-
ECOKI00869 AFK--GRGVSACATCDGF--FYRNQ-KVAVIGGGNTAVEEALYLSNIASEVHLIHRRDG-
ECOC100920 AFK--GRGVSACATCDGF--FYRNQ-KVAVIGGGNTAVEEALYLSNIASEVHLIHRRDG-
ECOC200920 AFK--GRGVSACATCDGF--FYRNQ-KVAVIGGGNTAVEEALYLSNIASEVHLIHRRDG-
ECO8N00803 AFK--GRGVSACATCDGF--FYRNQ-KVAVIGGGNTAVEEALYLSNIASEVHLIHRRDG-
ECO2700862 AFK--GRGVSACATCDGF--FYRNQ-KVAVIGGGNTAVEEALYLSNIASEVHLIHRRDG-
ECO2600974 AFK--GSGVSACATCDGF--FYRNQ-KVAVIGGGNTAVEEALYLSNIASEVHLIHRRDG-
ECOH100942 AFK--GRGVSACATCDGF--FYRNQ-KVAVIGGGNTAVEEALYLSNIASEVHLIHRRDG-
SHIB301848 AFK--GRGVSACATCDGF--FYRNQ-KVAVIGGGNTAVEEALYLSNIASEVHLIHRRDG-
ECOLI00846 AFK--GRGVSACATCDGF--FYRNQ-KVAVIGGGNTAVEEALYLSNIASEVHLIHRRDG-
ECO5700970 AFK--GRGVSACATCDGF--FYRNQ-KVAVIGGGNTAVEEALYLSNIASEVHLIHRRDG-
SHISS00756 AFK--GRGVSACATCDGF--FYRNQ-KVAVIGGGNTAVEEALYLSNIASEVHLIHRRDG-
SHIBS00679 AFK--GRGVSACATCDGF--FYRNQ-KVAVIGGGNTAVEEALYLSNIASEVHLIHRRDG-
SHIDS01857 AFK--GRGVSACATCDGF--FYRNQ-KVAVIGGGNTAVEEALYLSNIASEVHLIHRRDG-
ECO2400906 AFK--GRGVSACATCDGF--FYRNQ-KVAVIGGGNTAVEEALYLSNIASEVHLIHRRDG-
ECODH00784 AFK--GRGVSACATCDGF--FYRNQ-KVAVIGGGNTAVEEALYLSNIASEVHLIHRRDG-
ECOHS00922 AFK--GRGVSACATCDGF--FYRNQ-KVAVIGGGNTAVEEALYLSNIASEVHLIHRRDG-
ECOLC02627 AFK--GRGVSACATCDGF--FYRNQ-KVAVIGGGNTAVEEALYLSNIASEVHLIHRRDG-
ECO5E00946 AFK--GRGVSACATCDGF--FYRNQ-KVAVIGGGNTAVEEALYLSNIASEVHLIHRRDG-
ECOSE00936 AFK--GRGVSACATCDGF--FYRNQ-KVAVIGGGNTAVEEALYLSNIASEVHLIHRRDG-
ECO5500894 AFK--GRGVSACATCDGF--FYRNQ-KVAVIGGGNTAVEEALYLSNIASEVHLIHRRDG-
ECO8A00877 AFK--GRGVSACATCDGF--FYRNQ-KVAVIGGGNTAVEEALYLSNIASEVHLIHRRDG-
ECOBB00863 AFK--GRGVSACATCDGF--FYRNQ-KVAVIGGGNTAVEEALYLSNIASEVHLIHRRDG-
ECO5T00943 AFK--GRGVSACATCDGF--FYRNQ-KVAVIGGGNTAVEEALYLSNIASEVHLIHRRDG-
ECOBW00723 AFK--GRGVSACATCDGF--FYRNQ-KVAVIGGGNTAVEEALYLSNIASEVHLIHRRDG-
ECO1000919 AFK--GRGVSACATCDGF--FYRNQ-KVAVIGGGNTAVEEALYLSNIASEVHLIHRRDG-
ECOBD02604 AFK--GRGVSACATCDGF--FYRNQ-KVAVIGGGNTAVEEALYLSNIASEVHLIHRRDG-
ECOD102659 AFK--GRGVSACATCDGF--FYRNQ-KVAVIGGGNTAVEEALYLSNIASEVHLIHRRDG-
ECOBR00856 AFK--GRGVSACATCDGF--FYRNQ-KVAVIGGGNTAVEEALYLSNIASEVHLIHRRDG-
ECOLX02610 AFK--GRGVSACATCDGF--FYRNQ-KVAVIGGGNTAVEEALYLSNIASEVHLIHRRDG-
ECO1A00939 AFK--GRGVSACATCDGF--FYRNQ-KVAVIGGGNTAVEEALYLSNIASEVHLIHRRDG-
ECOCB01054 AFK--GRGVSACATCDGF--FYRNQ-KVAVIGGGNTAVEEALYLSNIASEVHLIHRRDG-
ECOKO02859 AFK--GRGVSACATCDGF--FYRNQ-KVAVIGGGNTAVEEALYLSNIASEVHLIHRRDG-
ECO1E03320 AFK--GRGVSACATCDGF--FYRNQ-KVAVIGGGNTAVEEALYLSNIASEVHLIHRRDG-
ECOLW01217 AFK--GRGVSACATCDGF--FYRNQ-KVAVIGGGNTAVEEALYLSNIASEVHLIHRRDG-
SHIFL01399 AFK--GRGVSACATCDGF--FYRNQ-KVAVIGGGNTAVEEALYLSNIASEVHLIHRRDG-
SHIF800759 AFK--GRGVSACATCDGF--FYRNQ-KVAVIGGGNTAVEEALYLSNIASEVHLIHRRDG-
SHIF200805 AFK--GRGVSACATCDGF--FYRNQ-KVAVIGGGNTAVEEALYLSNIASEVHLIHRRDG-
CITK802118 AFK--GRGVSACATCDGF--FYRNQ-KVAVIGGGNTAVEEALYLANIAAEVHLIHRRDG-

```
CITRI00924      AFK--GRGVSACATCDGF--FYRNQ-KVAVIGGGNTAVEEALYLANIASEVHLIHRRDT-
CROS802385      AYK--GRGVSACATCDGF--FYRNQ-KVAVIGGGNTAVEEALYLANIASEVHLIHRRDT-
CROTZ01500      AYK--GRGVSACATCDGF--FYRNQ-KVAVIGGGNTAVEEALYLANIASEVHLIHRRDT-
ENTLS02865      AFK--GRGVSACATCDGF--FYRNQ-KVAVIGGGNTAVEEALYLANIAAEVHLIHRRDT-
ENT3801400      AFK--GRGVSACATCDGF--FYRNQ-KVAVIGGGNTAVEEALYLANIASEVHLIHRRDT-
ENTAL01384      AFK--GRGVSACATCDGF--FYRNQ-KVAVIGGGNTAVEEALYLANIASEVHLIHRRDT-
ENTCC02679      AFK--GRGVSACATCDGF--FYRNQ-KVAVIGGGNTAVEEALYLANIASEVHLIHRRDT-
                       *   *  *:.              *    *      :

STRT101547      --LRAQ-KVLQARAFAN---EKVKFIWDSVV-EEIKGDD---------------------
STRT201508      --LRAQ-KVLQARAFAN---EKVKFIWDSVV-EEIKGDD---------------------
STRTD01356      --LRAQ-KVLQARAFAN---EKVKFIWDSVV-EEIKGDD---------------------
STRTN01533      --LRAQ-KVLQARAFAN---EKVKFIWDSVV-EEIKGDD---------------------
STRE500345      --LRAQ-KVLQDRAFAN---EKVKFIWDSVV-EEIKGDD---------------------
STRE801625      --LRAQ-KVLQDRAFAN---EKVKFIWDSVV-EEIKGDD---------------------
STREH01636      --LRAQ-KVLQDRAFAN---EKVKFIWDSVV-EEIKGDD---------------------
STREC01656      --LRAQ-KILQERAFAN---EKLDFIWDSVV-REIQGND---------------------
STREM01495      --LRAQ-KILQERAFAN---EKLDFIWDSVV-KEIQGND---------------------
STRE401629      --LRAQ-KILQERAFAN---EKLDFIWDSVV-KEIQGND---------------------
STRS700387      --LRAQ-KILQERAFAN---EKLDFIWDSVV-KEIQGND---------------------
STRDG01631      --LRAQ-KILQDRAFAN---EKVDFIWDSVV-KEIQGND---------------------
STRP301388      --LRAQ-KILQDRAFAN---DKVDFIWDSVV-KEIKGND---------------------
STRPZ01205      --LRAQ-KILQDRAFAN---DKVDFIWDSVV-KEIKGND---------------------
STRPQ00464      --LRAQ-KILQDRAFAN---DKVDFIWDSVV-KEIKGND---------------------
STRPD01453      --LRAQ-KILQDRAFAN---DKVDFIWDSVV-KEIKGND---------------------
STRP601399      --LRAQ-KILQDRAFAN---DKVDFIWDSVV-KEIKGND---------------------
STRP801366      --LRAQ-KILQDRAFAN---DKVDFIWDSVV-KEIQGND---------------------
STRPF01453      --LRAQ-KILQDRAFAN---DKVDFIWDSVV-KEIQGND---------------------
STRPG00427      --LRAQ-KILQDRAFAN---DKVDFIWDSVV-KEIQGND---------------------
STRA300284      --LRAQ-KVLQDRAFAN---EKIKFVWDSVV-KEIKGNE---------------------
STRA500290      --LRAQ-KVLQDRAFAN---EKIKFVWDSVV-KEIKGNE---------------------
STRA100288      --LRAQ-KVLQDRAFAN---EKIKFVWDSVV-KEIKGNE---------------------
STRA200291      --LRAQ-KVLQDRAFAN---EKIKFVWDSVV-KEIKGNE---------------------
STRIC00348      --LRAQ-KILQDRAFAN---EKINFIWDSVV-KEIKGND---------------------
STRPX00375      --LRAQ-KILQDRAFAN---DKINFIWDSVV-KEIKGTD---------------------
STRMD00432      --LRAQ-KILQDRAFAN---DKINFIWDSVV-KEIKGTD---------------------
STRS201734      --LRAQ-KVIQDRAFAN---EKINFIWDSVV-EEIKGDD---------------------
STRSY01721      --LRAQ-KVIQDRAFAN---EKINFIWDSVV-EEIKGDD---------------------
STRSX01553      --LRAQ-KVIQDRAFAN---EKINFIWDSVV-EEIKGDD---------------------
STRSE01478      --LRAQ-KVIQDRAFAN---EKINFIWDSVV-EEIKGDD---------------------
STREJ01635      --LRAQ-KVIQDRAFAN---EKINFIWDSVV-EEIKGDD---------------------
STRGZ01544      --LRAQ-KVIQDRAFAN---EKINFIWDSVV-EEIKGDD---------------------
STRS401593      --LRAQ-KVIQDRAFAN---EKINFIWDSVV-EEIKGDD---------------------
LACGT00720      --LRAQ-KIIQERAFAN---DKIKFIWDSVL-EEIKGDE---------------------
LACGL00738      --LRAQ-KIIQERAFAN---DKIKFIWDSVL-EEIKGDE---------------------
STRSV01774      --LRAQ-KVLQDRAFAN---EKIRFVWDSVV-ESIHGDE---------------------
STRIJ00350      --LRAQ-KLLQDRAFAN---EKIHFIWDSVV-QEIKGDN---------------------
STROU01248      --LRAQ-KVLQDRAFAN---EKVNFIWDSVV-KEIKGEN---------------------
STRM601286      --LRAQ-KVLQDRAFAN---EKISFIWDSVV-KEIKGEN---------------------
STRES00962      --LRAQ-KVLQDRAFAN---EKISFIWDSVV-RKIKGEN---------------------
STRP701387      --LRAQ-KVLQDRAFAN---EKISFIWDSVV-REIKGEN---------------------
STRZT00756      --LRAQ-KVLQDRAFAN---EKISFIWDSVV-REIKGEN---------------------
STRP001079      --LRAQ-KVLQDRAFAN---EKISFIWDSVV-REIKGEN---------------------
STRZO01270      --LRAQ-KVLQDRAFAN---EKISFIWDSVV-REIKGEN---------------------
STRZ600815      --LRAQ-KVLQDRAFAN---EKISFIWDSVV-REIKGEN---------------------
STRET00826      --LRAQ-KVLQDRAFAN---EKISFIWDSVV-REIKGEN---------------------
STRPS01429      --LRAQ-KVLQERAFAN---EKISFIWDSVV-REIKGEN---------------------
STRZN01287      --LRAQ-KVLQERAFAN---EKISFIWDSVV-REIKGEN---------------------
STRR601306      --LRAQ-KVLQDRAFAN---EKISFIWDSVV-KEIKGEN---------------------
STRP201217      --LRAQ-KVLQDRAFAN---EKISFIWDSVV-KEIKGEN---------------------
STRZP01342      --LRAQ-KVLQDRAFAN---EKISFIWDSVV-KEIKGEN---------------------
STRZI01226      --LRAQ-KVLQDRAFAN---EKISFIWDSVV-KEIKGEN---------------------
STRPN01360      --LRAQ-KVLQDRAFAN---EKISFIWDSVV-REIKGEN---------------------
STRP401357      --LRAQ-KVLQDRAFAN---EKISFIWDSVV-REIKGEN---------------------
STRZJ01282      --LRAQ-KVLQDRAFAN---EKISFIWDSVV-REIKGEN---------------------
```

```
STRPJ01336      --LRAQ-KVLQDRAFAN---EKISFIWDSVV-REIKGEN---------------------
STRPI01436      --LRAQ-KVLQDRAFAN---EKISFIWDSVV-REIKGEN---------------------
MARHT00044      --LRAN-KVAQARAFNN---PKIDFLWSHVV-TEILGED---------------------
THEP300413      --LRAT-KIEQEKAFAN---EKIEFIWDTVV-VDVEGEY---------------------
THEPX00884      --LRAT-KIEQEKAFAN---EKIEFIWDTVV-VDVEGEY---------------------
THESX01857      --LRAT-KIEQEKAFAN---EKIEFIWDTVV-VDVEGEY---------------------
THEM301788      --LRAT-KIEQEKAFAN---EKIEFIWDTIV-VDVEGEY---------------------
THEIA01812      --LRAT-KIEQEKAFAN---EKIEFIWDTIV-VDVEGEY---------------------
THETC00443      --LRAT-KTLQDRAFAN---PKIEFIWDTVV-KDIQGEY---------------------
THESW01026      --LRAS-KTLQDRAFAN---PKIEFIWDTVV-KDIQGEY---------------------
THEXL00360      --LRAS-KTLQDRAFAN---PKIEFIWDTVV-KDIQGEY---------------------
THEID01127      --LRAQ-KILQERALSN---KKIIPIWNTVV-EEILGDD---------------------
THEOJ00147      --LRAT-KILQERAFKN---EKIKFIWDSVV-DEIKGGD---------------------
DESAS01237      --LRAA-KIIQQRAKDN---PKIKFIWHSVV-EEISGSS---------------------
DESK701373      --LRAT-KIVQERARQN---PRIEFIWHNVV-EEITGKE---------------------
KYRT200729      --LRAQ-PVLQERARSN---EKISFLFNRRP-VEVQGE----------------------
STACT00410      --LRAQ-KILQDRAFKN---EKMDFIWNHTL-AAINEEN---------------------
STAS101952      --LRAQ-KILQDRAFKN---EKIDFIWSHTL-KTINDKD---------------------
STALH01969      --LRAQ-KILQDRAFKN---DKVNFIWSHTL-KSINDKD---------------------
STAEQ00422      --LRAQ-NILQERAFKN---DKVDFIWSHTL-KTINEKD---------------------
STAES00543      --LRAQ-NILQERAFKN---DKVDFIWSHTL-KTINEKD---------------------
STAAB00714      --LRAQ-RILQDRAFKN---DKIDFIWSHTL-KSINEKD---------------------
STAA500765      --LRAQ-RILQDRAFKN---DKIDFIWSHTL-KSINEKD---------------------
STAAM00757      --LRAQ-RILQDRAFKN---DKIDFIWSHTL-KSINEKD---------------------
STAAW00726      --LRAQ-RILQDRAFKN---DKIDFIWSHTL-KSINEKD---------------------
STAAS00733      --LRAQ-RILQDRAFKN---DKIDFIWSHTL-KSINEKD---------------------
STAAN00727      --LRAQ-RILQDRAFKN---DKIDFIWSHTL-KSINEKD---------------------
STAAC00807      --LRAQ-RILQDRAFKN---DKIDFIWSHTL-KSINEKD---------------------
STAA300727      --LRAQ-RILQDRAFKN---DKIDFIWSHTL-KSINEKD---------------------
STAA800734      --LRAQ-RILQDRAFKN---DKIDFIWSHTL-KSINEKD---------------------
STAA100752      --LRAQ-RILQDRAFKN---DKIDFIWSHTL-KSINEKD---------------------
STAA200774      --LRAQ-RILQDRAFKN---DKIDFIWSHTL-KSINEKD---------------------
STAA900759      --LRAQ-RILQDRAFKN---DKIDFIWSHTL-KSINEKD---------------------
STAAE00716      --LRAQ-RILQDRAFKN---DKIDFIWSHTL-KSINEKD---------------------
STAAT00767      --LRAQ-RILQDRAFKN---DKIDFIWSHTL-KSINEKD---------------------
STAAD00695      --LRAQ-RILQDRAFKN---DKIDFIWSHTL-KSINEKD---------------------
STAA000815      --LRAQ-RILQDRAFKN---DKIDFIWSHTL-KSINEKD---------------------
STAAH02407      --LRAQ-RILQDRAFKN---DKIDFIWSHTL-KSINEKD---------------------
STAAF00763      --LRAQ-RILQDRAFKN---DKIDFIWSHTL-KSINEKD---------------------
STAAK00744      --LRAQ-RILQDRAFKN---DKIDFIWSHTL-KSINEKD---------------------
STAAJ00703      --LRAQ-RILQDRAFKN---DKIDFIWSHTL-KSINEKD---------------------
STAAG00690      --LRAQ-RILQDRAFKN---DKIDFIWSHTL-KSINEKD---------------------
STAA400732      --LRAQ-RILQDRAFKN---DKIDFIWSHTL-KSINEKD---------------------
STAAR00788      --LRAQ-RILQDRAFKN---DKIDFIWSHTL-KSINEKD---------------------
LISSS02378      --LRAQ-QILQDRAFKD---EKVDFIWNSTV-EEIIGDG---------------------
LISIN02590      --LRAQ-QILQDRAFKD---EKVDFIWNNTV-EEIIGDG---------------------
LISW602421      --LRAQ-QILQDRAFKD---EKVDFVWNSTV-EEIIGDG---------------------
EXISA00870      --LRAQ-KILQKRAFDN---EKIDFIWNHTL-KEITEKD---------------------
EXIS202358      --LRAQ-KILQKRAFDN---PKIDFIWNHTV-KQINEDN---------------------
EXIAB02180      --LRAQ-KILQKRAFDN---PKIDFIWNHTV-KQINEDN---------------------
OCEIH02462      --LRAQ-KIIQDRAFDN---EKIDFIWDTVA-ETINGTD---------------------
BACIE01058      --LRAQ-KILQDRAFDN---DKIEFEWNHVV-KEINGEG---------------------
BACCJ03468      --FRAQ-KILQDRAFAN---EKIDVIWNHTV-KEINGED---------------------
BACHD03507      --LRAQ-KILQQRAFDN---DKIEFIWDHVV-KQINGTD---------------------
BACPE03382      --LRAQ-KILQDRAFNN---DKIEFVWNHVV-NEINEEG---------------------
SOLSS00743      --LRAQ-KIIQDRAFAN---EKIDFIWNTTV-KEIHEVD---------------------
BACC600715      --LRAQ-KILQDRAFKN---EKIDFIWNHTV-KEIHEEN---------------------
ANOFW02486      --LRAQ-KILQQRAFAN---EKIDFIWNHTV-KQINEKD---------------------
GEOKA03042      --LRAQ-KILQDRAFAN---EKIDFIWNHTV-KQINEKD---------------------
GEOSY02976      --LRAQ-KILQDRAFAN---EKIDFIWNHTV-KQINEKD---------------------
GEOTN02952      --LRAQ-KILQDRAFAN---EKVDFIWNHTV-KQINEKD---------------------
GEOSW02588      --LRAQ-KILQDRAFAN---EKIDFIWNHTV-KQINGKD---------------------
GEOS000381      --LRAQ-KILQDRAFAN---EKIDFIWNHTV-KQINGKD---------------------
GEOTC00378      --LRAQ-KILQDRAFAN---EKIDFIWNHTV-KQINGKD---------------------
BACMD04906      --LRAQ-KILQQRAFDN---EKVDFIWNTTV-KEVNEKD---------------------
```

```
BACMQ04908      --LRAQ-KILQQRAFDN---EKVDFIWNTTV-KEVNEKD---------------------
BACWK04830      --LRAQ-KILQDRAFQN---EKVDFIWNHTI-KEINDAN---------------------
BACAN04889      --LRAQ-KILQDRAFQN---EKVDFIWNHTI-KEINEAN---------------------
BACC105210      --LRAQ-KILQDRAFQN---EKVDFIWNHTI-KEINEAN---------------------
BACC705051      --LRAQ-KILQDRAFQN---EKVDFIWNHTI-KEINEAN---------------------
BACC005112      --LRAQ-KILQDRAFQN---EKVDFIWNHTI-KEINEAN---------------------
BACC305043      --LRAQ-KILQDRAFQN---EKVDFIWNHTI-KEINEAN---------------------
BACAC05232      --LRAQ-KILQDRAFQN---EKVDFIWNHTI-KEINEAN---------------------
BACAA04720      --LRAQ-KILQDRAFQN---EKVDFIWNHTI-KEINEAN---------------------
BACT005085      --LRAQ-KILQDRAFQN---EKVDFIWNHTI-KEINEAN---------------------
BACC205124      --LRAQ-KILQDRAFQN---EKVDFIWNHTI-KEINEAS---------------------
BACC405064      --LRAQ-KILQDRAFQN---EKVDFIWNHTI-KEINEAS---------------------
BACT104712      --LRAQ-KILQDRAFQN---EKVDFIWNHTI-KEINEAS---------------------
BACLD03769      --LRAQ-SILQARAFDN---EKVDFLWNKTV-KEIHEQD---------------------
BACPZ03376      --LRAQ-SILQARAFDN---EKVDFLWNKTV-KEIHEEN---------------------
BACSU03600      --LRAQ-SILQARAFDN---EKVDFLWNKTV-KEIHEEN---------------------
BACST01653      --LRAQ-SILQARAFDN---EKVDFLWNKTV-KEIHEEN---------------------
BACPT03541      --LRAQ-SILQARAFDN---EKVDFLWNKTV-KEIHEEN---------------------
LEUGG00661      --LRAQ-KIIQDRAFAN---DKMSFRWHTEV-IEITGDS---------------------
LEUGJ00629      --LRAQ-KIIQDRAFAN---DKMSFRWHTEV-IEITGDS---------------------
LEUCJ00485      --LRAQ-QIIQDRAFAN---DKINFKWHTEV-VGIIGDK---------------------
LACAR00657      --LRAQ-PTLQKRAFAN---KKMKFVWNAQT-EEILGDG---------------------
LACA300647      --LRAQ-PTLQKRAFAN---KKMKFVWNAQT-EEILGDG---------------------
LACAL00643      --LRAQ-PTLQKRAFAN---KKMKFVWNAQT-EEILGDG---------------------
LACKZ00964      --LRAQ-PTLQKRAFAN---KKMKFVWNAQT-EEIVGDG---------------------
LACRJ00357      --LRAE-PIIQAEAMNN---DKIEFVYNTSV-TEIVGDD---------------------
LACRD00361      --LRAE-PIIQAEAMNN---DKIEFVYNTSV-TEIVGDD---------------------
LACRS01501      --LRAE-PIIQAEAMNN---DKIEFVYNTSV-TEIVGDD---------------------
LACSM00455      --LRAQ-RILQERAFDR---DNIDFIWNSTV-EEIQGDD---------------------
LACRG00878      --LRAQ-QIIQKRAFAN---DKMHFVWNAQV-QEIQGDD---------------------
LACRL00939      --LRAQ-QIIQKRAFAN---DKMHFVWNAQV-QEIQGDD---------------------
LACC300844      --LRAQ-QIIQKRAFAN---DKMHFVWNAQV-QEIQGDD---------------------
LACCZ00824      --LRAQ-QIIQKRAFAN---DKMHFVWNAQV-QEIQGDD---------------------
LACCB00988      --LRAQ-QIIQKRAFAN---DKMHFVWNAQV-QEIQGDD---------------------
LACCD01016      --LRAQ-QIIQKRAFAN---DKMHFVWNAQV-QEIQGDD---------------------
LACCC01014      --LRAQ-QIIQKRAFAN---DKMHFVWNAQV-QEIQGDD---------------------
LACBN01277      --LRAQ-KVIQDRAFAN---DKIDFVWNSNV-TEVLGDD---------------------
LACBA00605      --LRAQ-KILQDRAFAN---DKIEFVWNTNV-TEILGDD---------------------
LACPL00650      --LRAQ-QILQDRAFAN---PKMEFVWNSNV-TEIIGDD---------------------
LACPJ00628      --LRAQ-QILQDRAFAN---PKMEFVWNSNV-TEIIGDD---------------------
LACPS00585      --LRAQ-QILQDRAFAN---PKMEFVWNSNV-TEIIGDD---------------------
PEDCP00497      --LRAQ-KITQQRAFDN---DKMEFVWNSNV-TEIVGDD---------------------
CARS100359      --LRAQ-KILQDRAFKN---EKVDFIWDSTV-ENIYGDE---------------------
AERUA00263      --LRAQ-KILQDRAFAN---DKISFTWDSVV-KEIKGDG---------------------
ELUMP00590      --FRAA-QKTVEKVKAC---PNVEMVLNYTA-ESFEGDS---------------------
SPHPG02940      --FRAQ-DNLVDQVNRN---GHIETVMQQTL-TKIEG-----------------------
SPHGB01868      --FRAQ-DNLVEQIKRN---KNIELVMQHTI-TEIKG-----------------------
TREPZ00273      --FRAQ-KSLAERVLHD---PRIEVRLNTSP-VKILG-----------------------
TREAZ03414      --FRAQ-KAIADRVLHN---PNIRISFNTIM-KEIKG-----------------------
SPITD00734      --FRAQ-KALAQRVLNN---PHIEVRFNTVA-VEIRGEEVN-------------------
SPITZ01373      --FRAQ-KALAQRVLNN---PHIEVRFNTVA-VEIRGEEVN-------------------
TREPA00803      --LRAQ-KAIAERTLKN---PHIAVQWNTTL-EAVR------------------------
TREPS00802      --LRAQ-KAIAERTLKN---PHIAVQWNTTL-EAVR------------------------
TREPC00747      --LRAQ-KAIAERTLKN---PHIAVQWNTTL-EAVR------------------------
TREPM00823      --LRAQ-KAIAERTLKN---PHIAVQWNTTL-EAVR------------------------
TREPD00824      --LRAQ-KAIAERTLKN---PHIAVQWNTTL-EAVR------------------------
TREPU00781      --LRAQ-KAIAERTLKN---PHIAVQWNTTL-EAVR------------------------
ENCCU00216      --FRAR-NDMVEKARET---VNIEMVTPYVL-ESALGTS---------------------
HELM100964      --FRAA-PVTIEHAKNN---EKIEFLTPYVV-EEILGDA---------------------
HELCP01490      --FRAA-PSTITHAKNN---DKIHFITPAVI-EEILGDS---------------------
ARCFU01526      --FRAE-KALVEEVEK----RGIPVHYSTTI-RKIIGSG---------------------
FERPA02445      --LRAE-KALQEELFK----RNIPVIWDTVV-LRIEGNE---------------------
ARCVS01910      --LRAD-RALQDELFK----RNIPVIWNSVV-ERIEGSN---------------------
METEZ00677      --LRAS-KVLEDRARSR---DNIEFLWDTVL-EEVKG-----------------------
METHD00869      --LRAS-MILQERVSKK---PNIEIIWDNVP-EEIQG-----------------------
```

```
METMA02304      --LKAA-RVLQDRVDGT---PNIELILNSHV-LEIVGTREG-------------------
METAC01311      --LKAA-KVLQDRALAT---PNIEFILNTLV-QEIAGSREG-------------------
KOSOT00298      --LTAD-KLLQERIKAT---GKVDFIFNTVV-DRIEG-----------------------
MARPK01613      --LRAD-KRYQDKAFNT---ENIKFIWNSVV-KEIKG-----------------------
SLAHD02439      --LRAA-KSIQEKAFAN---PKLNFMWDSVV-EEVGGDE---------------------
FILAD00976      --FRAN-KASLDKAREN---PNIKWLLNRKV-VEIKGD----------------------
BUTPB02463      --LRAT-KVLQEELFGL---DNVEVIWDSVV-KEIKGED---------------------
CLOPH00250      --FRAA-KTFITKLLSL---ENVTVHWNSVV-DEIVGTD---------------------
CLOSW00592      --LRGA-KTLQTQLFHQ---KNVEVIWDTVV-EEIEGGD---------------------
LACFC00207      --LKAQ-MVAQERAKKN---GQMEFIFNTEV-TEIKGDD---------------------
CRYCD00853      --MRAT-AADRHRVAQA---DNVVFHGNKVV-DELQQQD---------------------
EGGLE01847      --LRAT-AIYHKRLEDL---PNLEFVWNAVP-RKLVADE---------------------
PYRFU01410      --FRAD-KILQDRF-RE---AGIPAILNTVV-TEIRGKD---------------------
PYRHO01476      --FRAD-KILQDRF-KQ---AGIPAILNTVV-TEIKGTN---------------------
PYRAB00730      --FRAD-KILQDRL-KQ---AGIPTILNTVV-TEIRGTN---------------------
PYRSN00014      --FRAD-KILQDRL-KE---AGIPTILNTVV-TEIRGTN---------------------
THEGJ00181      --FRAD-KILQDRF-RE---SGIPAILDTVV-TEIIGKD---------------------
THEKO02097      --FRAD-KILQDRF-KE---SGIPAILDTVV-TEIIGKD---------------------
THEON01610      --FRAD-KILQDRF-KA---SGIPMILNTVV-TEIKGTN---------------------
THES401476      --FRAD-KILQDRF-KE---SGIPAILNTVV-TEIKGDG---------------------
SYNWW02368      --FRAN-QTALEKMMNN---EKIELKLNRVV-KRVEGDA---------------------
UNCTG00012      --LRAA-KILQERMKLH---PNISVIYDSVP-KEIFGSD---------------------
THEA101460      --FRAV-KIIQDRVAKN---DKIEPIMNKVV-VSINGKD---------------------
DENA201647      --FRAS-KLATERAQNN---EKMEFIYDSVV-ESINGDT---------------------
DEFDS00488      --LRAA-KILQDRAFAN---PKIEFIWNSVV-KKVNGED---------------------
CALNY01292      --FRAA-KILQDRVFNN---PKIEIIWNAQL-IRVNGDN---------------------
SYNGF01652      --FNAA-HTALEHARNN---PKISFILDSVI-QSIEGTD---------------------
DESB201123      --LRAA-AVLQERVLAN---EKIEVLWSHAP-VSINGEN---------------------
DEIPM00845      --LRAN-KSAQARVFAN---EKMRFIWDTTV-EEILGDE---------------------
DEIRA01924      --LRAN-KVAQARAFAN---PKMKFIWDTAV-EEIQGAD---------------------
DEIML01437      --LRAN-KVAQARAFAN---PKMKFIWDTVP-EEILGEGEG-------------------
DEIGD02532      --LRAN-KVAQARAFAN---PKMKFVWDTVV-EEILGQE---------------------
DEIDV00622      --LRAN-KVAQARAFSN---PKMKFIWDTAV-DEIQGED---------------------
TRURR01079      --LRAN-KEAQRRAFAN---PKMHWVWNSVV-EEILGGE---------------------
THETG01863      --LRAN-KVAQKRAFQN---PKMHFLFSHVV-TEILGED---------------------
THET201543      --LRAN-KVAQKRAFQN---PKMHFLFSHVV-TEILGED---------------------
THET801911      --LRAN-KVAQKRAFQN---PKMHFLFSHVV-TEILGED---------------------
OCEP502115      --LRAN-KVAQERAFQN---PKIEFLWSHVV-TEVLGED---------------------
DEHLB00585      --LRAT-PVLQEKAKSD---PKISFIWNTDV-VAIEGKD---------------------
DEHMG00439      --FRAD-AVLMDKAKNK---SNIELVLDTVV-TSINGQD---------------------
DEHMB00497      --FRAD-AVLMDKAKNK---SNIELVLDTVV-TSINGQD---------------------
DEHMC00427      --FRAD-AVLMDKAKNK---SNIELVLDTVV-TSINGQD---------------------
THELD00726      --FRAD-KVAVERAMTN---DKIEVIWNTVV-KEIAGDD---------------------
ANAMD00624      --FRAD-RAVVEKAMSN---PKIEPVWNSVV-ERIEGED---------------------
THEAS00815      --FRAD-KVPVERAMSN---PKIVPIFDSVV-EAIEGGD---------------------
AMICL00707      --FRAD-RAATERTLSN---PKIEPIWNSVV-EKIEGDG---------------------
CLOCE01684      --MRAT-KILQNELCCN---NRIEFFWDSVV-EEIEGQF---------------------
HYDS000616      --LRAK-PLLQDRVFSN---PKIEFVPNKVV-KAIQGSD---------------------
HYDTT00224      --LRAQ-KHLQEKVFSN---PKIKFIPDTVV-EEISGNE---------------------
PELTS01405      --LRAT-RAVQEKAFGN---PKIEFVWDAVV-SEISGSD---------------------
DESRL02179      --LRAT-KILQKRAVDN---PKIEFLWHAVV-EEIVGEN---------------------
BORBP00506      --LRAI-AMLRDSVAKL---PNIEIFYNSEA-IEVDGES---------------------
BORAP00518      --LRAI-AMLRDNVAKL---PNIEILYNSEA-TEVDGKS---------------------
BORBU00514      --LRAI-AMLRDSVAKL---PNIEILYNSEA-IEVDGKS---------------------
BORBZ00490      --LRAI-AMLRDSVAKL---PNIEILYNSEA-IEVDGKS---------------------
BORBN00493      --LRAI-AMLRDSVAKL---PNIEILYNSEA-IEVDGKS---------------------
BORRA00486      --MKAI-DMLQKSIKTL---SNVNILYNCEA-REVSGEN---------------------
BORDL00498      --MKAI-DMLQKSIKTL---SNVNILYNCEA-REVSGEN---------------------
BORHD00497      --LKAI-AMLRRDVEKL---PNVEILYNCEA-IEVNGDN---------------------
BORT900497      --LKAI-AMLRQNVEKL---PNVEILYNCEA-IEVNGDN---------------------
METKA01560      --LESPDRALIERVLSC---RNVEVVHDEV--RRIVGDE---------------------
MYCA500361      --IIAE-KRLVDDVKKH---SKIIVHENSQV-LELIGQD---------------------
METVS00145      --LKAVEKIMEERLVEI---PNLEIIYNAKP-VKVVGEN---------------------
METOI01431      --LRAAENIMLERLNDA---ENVKIITNAKP-LKILGDD---------------------
MYCHN00051      --FRAE-AISVKKARNI---KNIEIYTPYIPIGAEVKDN---------------------
```

```
MYCSL00465      --FRGE-EILVKQLKGK---ENVEIHTPYKPKKVLVDGD---------------------
MYCS300420      --FRGE-EILVKQLKGK---ENVEIHTPYKPKKVLVDGD---------------------
DESK101258      --FRAF-NVYVETARSN---PKITILTNTII-KEIIGKN---------------------
STAHD01472      --FRAF-PAYIEKAKQN---PKIEFITNTVI-KEIIGDT---------------------
THEC100270      --FRAF-KVYVEKAMNN---PKIEILKNSVV-KEIIGDT---------------------
NANEQ00478      --FRAE-SYLVENAKKK---DNIEFILNSVV-KEIRGQN---------------------
CALLD01225      --FRAK-PFYVNQVLNK---KNIEILYNSVV-KEIKGDN---------------------
SULSO02155      --FKAQ-PIYVETVKKK---PNVEFVLNSVV-KEIKGDK---------------------
SULS900210      --FKAQ-PIYVETVKKK---PNVEFVLNSVV-KEIKGDK---------------------
SULIA00208      --FKAQ-PIYVETVKKK---PNVEFVLNSVV-KEIKGDK---------------------
SULIM00207      --FKAQ-PIYVETVKKK---PNVEFVLNSVV-KEIKGDK---------------------
SULIK00225      --FKAQ-PIYVETVKKK---PNVEFVLNSVV-KEIKGDK---------------------
IGNH400907      --IEAS-ESYMSVLRNN---PKVEILEGAKV-KEIVGDG---------------------
KORCO01040      --MRAE-KALVDNLMSR---ENVKFLFNSVL-TAIVGEG---------------------
THESM01133      --FRAD-KILQERF-KK---AGIPVLLNNVV-VEIKGNQ---------------------
THEBM01534      --FRAD-KILQDRF-KE---AGIPAILNTVV-IEIKGKE---------------------
METST01363      --LRCD-SQLEKDL-HN---ANIKIYWNTQL-KSVNGND---------------------
METTH00703      --LRAD-KYLQDKL-RE---MEIPVIWNSVV-KEIGGDE---------------------
METSL02406      --LRAQ-KYLQEKL-KE---EGIPILWNTTL-KEIKGNM---------------------
METLA02406      --LRAQ-KYLQEKL-KE---EGIPILWNTTL-KEIKGNM---------------------
METPW00194      --LRAQ-KYLQDKI-KK---EGIPIVWNSVV-KEIKGDT---------------------
RUBXD00226      --FRAS-KIMLGRARKN---PKISFITDTVV-TDVLGED---------------------
RHOM400178      --LRAS-KIMQERAFKN---EKITFIWNTVV-EDVLGEN---------------------
GARV400051      --FRAS-KIMVDRAKEN---EKIKFITNSVV-DEVHGNDV--------------------
BIFAP01644      --FRAS-KIMVDRAKAN---DKINFLLDSVV-TRINGDGS--------------------
SEGRD00014      --FRAS-RIMLERAKSN---PKIKFVLGASV-SRVLGST---------------------
GORB404535      --FRSS-KIMLERAKAN---DKIRFITNATV-RKVIGDG---------------------
GORPV04920      --FRAS-RIMLDRARNN---DKIRFVTNAAV-KSVIGDG---------------------
MYCA904903      --FRAS-KIMLERAYAD---PKITVLTNTKI-VGVEGTD---------------------
MYCSS05356      --FRAS-RIMLDRARAN---EKITFMTNTAV-TEIEGDP---------------------
MYCSJ05684      --FRAS-RIMLDRARAN---EKITFMTNTAV-TEIEGDP---------------------
MYCSK05387      --FRAS-RIMLDRARAN---EKITFMTNTAV-TEIEGDP---------------------
MYCS206581      --FRAS-KIMLERARAN---EKITFLTNTEI-TQIEGDP---------------------
MYCCN05158      --FRAS-KIMLERAQNN---EKITIRTNTAV-TAIEGEP---------------------
MYCVP05890      --FRAS-KIMLERAQAN---EKITFLTNTQV-TAIEGDP---------------------
MYCGI00775      --FRAS-KIMLERAQQN---EKITFLTNTQV-LSIEGDP---------------------
MYCSR04994      --FRAS-KIMLERAQQN---EKITFLTNTQV-LSIEGDP---------------------
AMYSD04545      --FRAS-RIMLERAKAN---EKITFITNAKV-EEVLGDN---------------------
MYCLE02681      --FRAS-KIMLGRARNN---DKIKFITNHTV-VAVNGYT---------------------
MYCLB02681      --FRAS-KIMLGRARNN---DKIKFITNHTV-VAVNGYT---------------------
MYCSD04323      --FRAS-KIMLNRARDN---DKIRFLTNAVP-VAVEGTD---------------------
MYCPA04306      --FRAS-RIMLERARAN---DKITIVTNKAV-EAVEGSE---------------------
MYCA105023      --FRAS-RIMLERARAN---DKITIVTNKAV-EAVEGSE---------------------
MYCUA04107      --FRAS-KIMTNRAQAN---DKIRILTNKIV-LAVDGET---------------------
MYCMM05378      --FRAS-KIMINRAQAN---DKIRILTNKIV-LAVDGET---------------------
MYCA003905      --FRAS-KIMLDRARNN---DKIRFLTNHTV-VAVDGDT---------------------
MYCTU03943      --FRAS-KIMLDRARNN---DKIRFLTNHTV-VAVDGDT---------------------
MYCTF03846      --FRAS-KIMLDRARNN---DKIRFLTNHTV-VAVDGDT---------------------
MYCTA03980      --FRAS-KIMLDRARNN---DKIRFLTNHTV-VAVDGDT---------------------
MYCTK04010      --FRAS-KIMLDRARNN---DKIRFLTNHTV-VAVDGDT---------------------
MYCTC03612      --FRAS-KIMLDRARNN---DKIRFLTNHTV-VAVDGDT---------------------
MYCTD03548      --FRAS-KIMLDRARNN---DKIRFLTNHTV-VAVDGDT---------------------
MYCCP03898      --FRAS-KIMLDRARNN---DKIRFLTNHTV-VAVDGDT---------------------
MYCBO02863      --FRAS-KIMLDRARNN---DKIRFLTNHTV-VAVDGDT---------------------
MYCBP03913      --FRAS-KIMLDRARNN---DKIRFLTNHTV-VAVDGDT---------------------
MYCBT03913      --FRAS-KIMLDRARNN---DKIRFLTNHTV-VAVDGDT---------------------
TSUPD04078      --FRAS-KIMLDRARDN---EKIRFLTDTKV-TAVQGEK---------------------
ACTMD06897      --FRAS-RIMLERARAN---EKIKWLLNSEV-TEVVGTGG--------------------
SACES08402      --FRSS-RIMLERARSN---EKIKWLLNAEV-AEVLGEG---------------------
SACVD03814      --FRAS-KIMLERARAN---EKIKWKLNSEV-IEVLGDD---------------------
AMYMU09190      --FRAS-KIMLERARAN---EKIKWQLNSQI-TGVLGDG---------------------
AMYMS10178      --FRAS-KIMLERARAN---EKIKWQLNSQI-TGVLGDG---------------------
PSEUX06421      --FRAS-RIMLERAQND---PKIKWRTNAEV-VRVDGDG---------------------
NOCFA05681      --FRAS-RIMLERAKAN---SKIKFVLNAEV-TEVHGDT---------------------
NOCCG05476      --FRAS-RIMLERAKAN---EKIRWVTNAEV-VRVNGDT---------------------
```

```
RHOE406010       --FRAS-RIMLERAKAN---EKIRFLTNAEP-VEVLGEN---------------------
RHOOB03468       --FRAS-RIMLERAKAN---AKIRFVTNAAP-VEVLGEN---------------------
RHOE104515       --FRAS-RIMLERAKAN---EKIRFVTNAEP-VEVLGEN---------------------
CORDI02302       --FRAS-AIMLERAKNN---PKIEFVTNKTV-SKVLG-DT--------------------
CORD202219       --FRAS-AIMLERAKNN---PKIEFVTNKTV-SKVLG-DT--------------------
CORDL02208       --FRAS-AIMLERAKNN---PKIEFVTNKTV-SKVLG-DT--------------------
CORDJ02208       --FRAS-AIMLERAKNN---PKIEFVTNKTV-SKVLG-DT--------------------
CORDH02221       --FRAS-AIMLERAKNN---PKIEFVTNKTV-SKVLG-DT--------------------
CORD702316       --FRAS-AIMLERAKNN---PKIEFVTNKTV-SKVLG-DT--------------------
CORD302337       --FRAS-AIMLERAKNN---PKIEFVTNKTV-SKVLG-DT--------------------
CORDD02235       --FRAS-AIMLERAKNN---PKIEFVTNKTV-SKVLG-DT--------------------
CORDV02170       --FRAS-AIMLERAKNN---PKIEFVTNKTV-SKVLG-DT--------------------
CORDW02254       --FRAS-AIMLERAKNN---PKIEFVTNKTV-SKVLG-DT--------------------
CORDK02230       --FRAS-AIMLERAKNN---PKIEFVTNKTV-SKVLG-DT--------------------
COREF02870       --FRAS-AIMLERAQKN---EKIRFVTNKTV-EEVIENDG--------------------
CORGL03082       --FRAS-AIMLERAQKN---EKIRFVTNKTV-EEVIEADG--------------------
CORGK02974       --FRAS-AIMLERAQKN---EKIRFVTNKTV-EEVIEADG--------------------
CORGB03038       --FRAS-AIMLERAQKN---EKIRFVTNKTV-EEVIEADG--------------------
CORK402006       --FRAS-NIMVQRAKDN---PKINFEMNAVV-TEVCG-DG--------------------
CORJK02028       --FRAS-KIMEERARNN---PKIKFLTNAKV-VEVKG-DK--------------------
CORVD02951       --FRAS-AIMEERARNN---PKIDMIMNATV-DEVLG-EG--------------------
ARCHD01706       --LRAS-KVMANRIEEI---ENIKVHWNSKV-AEIHGSG---------------------
THET101205       --LRAS-KIMQERAKSN---PKIDFIWNTVV-EEILGDQ---------------------
PROAC02247       --LRAS-QIMADRAAAD---PKITFAWNSEV-VAMQGES---------------------
PROAS02302       --LRAS-QIMADRAAAD---PKITFAWNSEV-VAMQGES---------------------
CAERE29798       --FRAS-RIMAQRVLDD---PKIEVAWNSEV-AAILGDE---------------------
CELFA03749       --LRAS-KIMADRAAAD---PKIEFAWNSEV-VAIHGTE---------------------
JONDD02484       --LRAS-KIMEERARNN---EKIRFIFNSEV-VAIHGDD---------------------
XYLCX03324       --LRAS-KIMAERALAN---PKIDFAWNSAV-EAISGDA---------------------
ACIC102144       --LRAS-KIMQDRVFAN---DKIRIRWNARV-IDVLGDD---------------------
FRADG04042       --LRAS-AIMQQRAFAN---DKIRFRWNAEV-VAILGEE---------------------
FRASU07065       --LRAS-AIMAERALAN---PKIKFRWNAVV-TGLEGDS---------------------
FRASN07115       --LRAS-AIMQERAFAN---PKVRFRWNAVV-EEVLGED---------------------
FRASC04424       --LRAS-AIMQERAFAN---EKIRFRWNAEV-TELLGAD---------------------
FRAAA06712       --LRAS-AIMQERAFSN---DKIRFRWNADV-AEILGED---------------------
KYTSD02521       --LRAS-QIMSDRAHAD---PKIEFAWNSEV-AEIHGDA---------------------
KINRD04462       --LRAS-RIMRKRAEDN---PKIRFAWNSEV-TGILGGD---------------------
NOCDD04789       --LRAS-RIMAERAHAN---DKITFLWNSAV-TEVLGEE---------------------
NOCAA01941       --LRAS-KIMADRAFAN---DKISFLWDTEV-TEVLGED---------------------
STRRD08913       --LRAS-KIMQDRAFAN---EKIRFVWDSEV-ADVLGES---------------------
THECD04863       --LRAS-KIMQDRAFAN---DKIRFVWDSEV-VDILGED---------------------
THEBD03528       --LRAS-KIMQDRAFAN---EKIRFLWNSQV-VDILGED---------------------
CATAD08894       --LRAS-KVMQERAFAN---PKISFVWDSEV-AGIEGET---------------------
KRIFD06917       --LRAS-KIMAERAFSN---DKIEFAWNSEV-AEIHGDD---------------------
KITSK03730       --LRAS-KAMQERAFAD---PKITFAWDSAV-EEIHGDP---------------------
STRBB05366       --LRAS-KAMQERAFAD---PKISFMWDSEV-TEIRGDN---------------------
STRVP03660       --LRAS-KAMQERAFAD---PKISFAWDSEV-TEIHGDQ---------------------
STRSW04381       --LRAS-KAMQDRAIAD---PKISFVWDSEV-AEIQGDQ---------------------
STRGG03562       --LRAS-KAMQDRAFAD---PKITFAWDSEV-AEIKGDQ---------------------
STRFA03098       --LRAS-KAMQDRAFAD---PKIKFAWDSEV-ATVHGDQ---------------------
STRAW04303       --LRAS-KAMQERAFAD---PKIKFVWDSEV-AEIKGDP---------------------
STRCO02929       --LRAS-KAMQERAFAD---PKISFVWDSEV-AEVQGDQ---------------------
STRHJ05105       --LRAS-KAMQERAFGD---PKIKFIWDSEV-AEIQGDQ---------------------
MONBE04991       --LRAS-KIMQNRAMNN---PKVEFVLSAEV-VEARGD----------------------
CHLRE01313       --FRAS-KIMAKRALEH---PKIEVLWNSVV-EEAYGN--E-------------------
MEDTR25591       --FRAS-KIMQSKALSN---EKIKVIWNSMV-VEAFGDG-E-------------------
SOLLC13750       --FRAS-KIMQNRALSN---PKIEVIWNSTV-VEAYGE----------------------
PRUPE10733       --FRAS-KIMQNRALSN---PKIRVVWNSEV-VEAYG-G-E-------------------
MANES18605       --FRAS-KIMQNRALSN---PKIEVIWNSVV-VEANGD----------------------
THECC00884       --FRAS-KIMQSRAVSN---PKIEVIWNSVV-VEAYGDG-E-------------------
PHYPA31147       --FRAS-KIMQARALGN---PKIEVIWNSSV-VEAKGNK---------------------
AMBTC19471       --FRAS-KIMQARALGN---PKIEVIWNSEV-VEAYGEE-N-------------------
MUSAC26038       -----------------------EVLWNSEV-VEAFGEA---------------------
MUSAM33177       -----------------------EVLWNSEV-VEAFGEA---------------------
SETIT03079       --FRAS-KIMQARALEN---PKIQVIWDSEV-VEAYGGA-G-------------------
```

```
ORYBR12195      --FRAS-KIMQARALTN---PKIQVVWDSEV-VEAYGGE-G-------------------
COCLU07729      --LRAS-RTNARRLTTH---PKIEVMFNTSG-VEIKGEEKP-------------------
PHANO13702      --LRAS-RTNARRLTTH---PKIEILYNTSA-SQIKGEDKK-------------------
PHAND10804      --LRAS-RTNARRLTTH---PKIEILYNTSA-SQIKGEDKK-------------------
AURPU02089      --LRAS-KANARRLSTN---PKVEIKYNTVA-TKITGEDKP-------------------
ZYMTR07711      --LRAS-KTNARRLANH---EKVEIRYNTQG-VEIKGEAGD-------------------
DICPU05926      --MRAS-KAMQERVKNN---AKINVLWDTVL-VEIKGDD---------------------
ENTHI00522      --FRAS-KTMQERVLNH---PKIEVIWNSEL-VELEGDG---------------------
LEPBA02231      --LRAS-QIMQKRAMEH---PKIEILWNQTV-VEAKGGA---------------------
LEPBP02301      --LRAS-QIMQKRAMEH---PKIEILWNQTV-VEAKGGA---------------------
LEPBL01462      --LRAS-KIMQKRATTH---PKIEIIWNSQV-KEAKGDG---------------------
LEPBJ01276      --LRAS-KIMQKRATTH---PKIEIIWNSQV-KEAKGDG---------------------
LEPIN02475      --LRAS-KIMQKRATTH---PKIEIIWNSQV-KEAKGDG---------------------
LEPII01996      --LRAS-KIMQKRATTH---PKIEIIWNSQV-KEAKGDG---------------------
LEPIC01426      --LRAS-KIMQKRATTH---PKIEIIWNSQV-KEAKGDG---------------------
SPIAZ00697      --LRAS-KAMQKRVFEN---EKITVMWNTEA-QEVLGEK---------------------
PENRW10140      --LRAS-NIMADRLLAH---PKCKVRFNTVA-TEVIGENKP-------------------
PENCH09104      --LRAS-NIMADRLLAH---PKCKVRFNTVA-TEVIGENKP-------------------
EURHE07269      --LRAS-KAMANRLLAH---PKVTVRFNSVA-TEVLGDAKP-------------------
ASPAC07301      --LRAS-KTMAQRLLAH---PKVTVRFNTVA-TEVLGEEKP-------------------
EMENI10387      --LRAS-KAMASRLLAN---PKVTVRFNTVA-TEVLGEKKL-------------------
EMEND02596      --LRAS-KAMASRLLAN---PKVTVRFNTVA-TEVLGEKKL-------------------
ASPTN06742      --LRAS-KAMASRLLSH---PKVNVRFNTVA-VEVLGEQKP-------------------
ASPCL04014      --LRAS-KAMANRLLAH---PKVTVRFNTVA-TEVLGEKKP-------------------
ASPFU05647      --LRAS-KAMAKRLLAH---PKVTVRFNTVA-TEVLGEKKP-------------------
NEOFI00452      --LRAS-KAMANRLLAH---PKVTVRFNTVA-TEVLGEKKP-------------------
CRYPA10563      --LRAS-NIMAKRLQNH---KDVTIKWNTVG-TEIKGDD---------------------
BLUGR03498      --LRAS-KTMANRLLNN---NKVTVKFNSVG-VEVMGNE---------------------
SCLS112814      --LRAS-KTMAKRLLAN---KKVTVKFNTVG-GEITGND---------------------
MAGGR04266      --LRAS-SIMAKRLLSH---PKVTVKFNSVG-VEVKGGE---------------------
NEUCR01575      --LRAS-SIMAHRLLNH---EKVTVRFNTVG-VEVKGDD---------------------
NEUT908941      --LRAS-SIMAHRLLNH---EKVTVRFNTVG-VEVKGDD---------------------
VERDA02342      --LRAS-AIMAKRLLGH---PKVTVRFHSGA-QEVKGDD---------------------
COLSU12486      --LRAS-QIMAKRLLSN---DKVTVRWNSVG-KEIKGGA---------------------
HYPAI01684      --LRAS-KAMATRLLKH---PKVTVRFNSVA-TEVKGDD---------------------
HYPVG06080      --LRAS-KAMAIRLLKH---PKVTVRFHSVA-TEVKGGE---------------------
HYPJE05895      --LRAS-KAMASRLLKH---PKVTVRFNTVA-TEVKGDK---------------------
NECHA05020      --LRAS-RTMANRLLAH---PKVTVRFNSHA-TEIKGGD---------------------
FUSO415847      --LRAS-RTMANRLLNH---PKVTVKFNSGA-TEIRGGE---------------------
GIBZA01026      --LRAS-RTMANRLLNH---PKCTVLFNSGA-TEIRGGE---------------------
SCHPO04025      --LRAS-PIMAKRLLAN---PKVEVLWNTVA-EEAQGDG---------------------
YARLI03635      --LRAS-AVMAKRLASH---PKVEILFNHVS-IEAKGDG---------------------
ASHGO00946      --MRAS-TIMQRRVERN---EKIEVLYNTAP-VEAKGDG---------------------
KLULA02190      --LRAS-QIMQRRAEQN---EKIEILYNHVT-LEAKGDG---------------------
ZYGRO00676      --LRAS-TIMQRRVQKN---DKIEILYNTVA-VEAKGDG---------------------
DEKBR01813      --MRAS-SIMQKRVQNN---PKMEVLYNTVS-VDTKGDG---------------------
PICPG04776      --LRAS-TIMQRRVEKN---EKLEVLYNTVS-KEAKGDG---------------------
CANTE00916      --LRAS-TIMQKRVKDN---EKLEILWNTEA-TEAKGDG---------------------
LODEL03891      --LRAS-SIMQKRAMNN---EKLEILWNTEA-KEALGDG---------------------
DEBHA05546      --LRAS-NIMQKRVQNN---DKLEILWNSEA-KEAKGDG---------------------
SPAPN03477      --LRAS-TIMQKRVLNN---DKLEVLWNTNA-VEAKGDG---------------------
CANAW04800      --LRAS-TIMQKRVTNN---EKIEVLWNTEA-LEAKGDG---------------------
PICST04701      --LRAS-TIMQRRVENN---EKLEILWNTEA-KEAKGDG---------------------
PUCGT10887      -----------------------TILWNTVP-VEAKGDG---------------------
PUCGR11813      -----------------------TILWNTVP-VEAKGDG---------------------
PHYBL11006      --LRAS-KVMASRLLKH---PKVTVHFNTVP-TETLGDG---------------------
USTMA03757      --LRAS-KIMAKRLLAH---PKVTVHFNTVP-IEAKGDG---------------------
USTHO04132      --LRAS-KIMAKRLLAH---PKVTVHFNTQP-AECKGDG---------------------
WALSE04527      --LRAS-KIMAQRLLKH---PKVTVHWNTQA-TEALGDG---------------------
TREME07701      --LRAS-KIMAKRLISH---PKVTVLWNTVA-TECKGDG---------------------
AURST04751      --LRAS-KIMAKRLTSH---PKVEIIWNTVA-TECLGDG---------------------
FOMME10177      --LRAS-KIMAKRLLSH---PKVTVLWNTVA-VECKGDG---------------------
CONPW06392      --LRAS-KIMAKRLMNH---PKITILWNTVA-TECLGDG---------------------
STEHR07076      --LRAS-KIMAKRVMNN---PKITILWNTVA-VECQGDG---------------------
HETAN06295      --LRAS-KIMAKRVLNN---PKITVLWNTVA-VECQGDG---------------------
```

```
GLOTR06982      --LRAS-KIMAKRLMNH---PKITILWNTVA-VECQGDG---------------------
PUNST01981      --LRAS-KIMAKRLMSH---PKVTILWNTVA-VECQGDG---------------------
LACBI02877      --LRAS-KIMAKRLQNN---PKITLLWHTVA-TECQGDG---------------------
COPCI16429      --LRAS-KIMQKRLLNN---PKITVLWNTVA-TECQGDG---------------------
DICSQ11618      --LRAS-KIMAKRLLSH---PKITILWNTVA-VECQGDG---------------------
TRAVS13180      --LRAS-KIMAKRLINH---PKITLLWNTVA-VECQGDG---------------------
WOLCO03584      --LRAS-KIMAKRLLNN---PKVTILWNTIA-TECQGDG---------------------
FOMPI05979      --LRAS-KIMAKRLQNN---PKVTILWNTFA-VECQGDG---------------------
PHLGI10219      --LRAS-KIMAKRLMNH---PKVTILWNTVA-VECQGDG---------------------
PHACH05757      --LRAS-KIMQKRLMNH---PKITILWNTVA-VECQGDG---------------------
RICTY00419      --FRAE-KILQDRLFKN---PKISVIWDHVI-DEIVGSNQP-------------------
RICPR00429      --FRAE-KILQDRLFKN---PKISVIWDHII-DEIVGSNKP-------------------
RICPP00461      --FRAE-KILQDRLFKN---PKISVIWDHII-DEIVGSNKP-------------------
RICBR00434      --FRAE-KILQERLFKN---PKISVIWDHVV-EEIVGNNNP-------------------
RICB800988      --FRAE-KILQERLFKN---PKISVIWDHVV-EEIVGNNNP-------------------
RICCK00600      --FRAE-KILQARLFKN---PKISVIWDHVV-DEIVGDNKP-------------------
RICAH00614      --FRAE-QILQDRLFKN---PKISVIWDHVV-DEIVGRNKP-------------------
RICAC00782      --FRAE-KILQDRLFKN---PKISVIWDHVV-DEIVGSNKP-------------------
RICFE00656      --FRAE-KILQDRLFKN---PKISVIWDHVV-DEIVGSDKP-------------------
RICMS00042      --FRAE-KILQDRLFKN---PKISVIWDHVV-DEIVGSNKP-------------------
RICM500453      --FRAE-KILQDRLFKN---PKISVIWDHVV-DEIVGNNKP-------------------
RICR300665      --FRAE-KILQDRLFKN---PKISVIWDHVV-DEIVGSNKP-------------------
RICAG00651      --FRAE-KILQDRLFKN---PKISVIWDHVI-DEIVGSNKP-------------------
RICP300630      --FRAE-KILQDRLFKN---SKISVIWDHVV-DEIVGSNKP-------------------
RICRS00632      --FRAE-KILQDRLFKN---SKISVIWDHVV-DEIVGSNKP-------------------
RICRO00663      --FRAE-KILQDRLFKN---SKISVIWDHVV-DEIVGSNKP-------------------
RICCN00618      --FRAE-KILQDRLFKN---SKISVIWDHVV-DEIVGSNKP-------------------
RICPT00620      --FRAE-KILQDRLFKN---SKISVIWDHVV-DEIVGSNKP-------------------
RICAE00497      --FRAE-KILQDRLFKN---SKISVIWDHVV-DEIVGSNKP-------------------
RICJY00467      --FRAE-KILQDRLFKN---SKISVIWDHVV-DEVVGSNKP-------------------
RICPU00072      --FRAE-KILQDRLFKN---SKISVIWDHVV-DEIVGSNKP-------------------
RICS100537      --FRAE-KILQDRLFKN---SKISVIWDHVV-DEIVGSNKP-------------------
BARBK00891      --FTAE-KILQDRLRSC---NNVHIIWDHVV-EEIIGLQTHA-----------------Q
BARVW00932      --FRAE-KILQDRLFAH---DNVRVLWDHVV-EEIVGLPAQD-----------------S
BART100617      --FRAE-KILQDRLFAC---NNVRVLWDHVV-EEIVGMPAQG-----------------A
BARGA00466      --FRAE-KILQDRLLAC---KNVRIIWDHVV-EEIVGLPAQG-----------------A
BARHE01107      --FRAE-KILQDRLFAR---DNVCVLWDHVV-EEIIGLPARA-----------------S
BARQU00895      --FRAE-KILQDRLFAC---DNVRVIWDHVV-EEIVGLPAHA-----------------S
OCHA401642      --FRAE-KIMQDRLLSR---QNVSVVWNSVI-DEILGTEAKP-----------------P
BRUAB01390      --FRAE-KIMQDRLLSR---ENVSVVWNSVI-DEILGTEARP-----------------P
BRUA201507      --FRAE-KIMQDRLLSR---ENVSVVWNSVI-DEILGTEARP-----------------P
BRUA101339      --FRAE-KIMQDRLLSR---ENVSVVWNSVI-DEILGTEARP-----------------P
BRUSU01458      --FRAE-KIMQDRLLSR---ENVSVVWNSVI-DEILGTEARP-----------------P
BRUME00510      --FRAE-KIMQDRLLSR---ENVSVVWNSVI-DEILGTEARP-----------------P
BRUSI01461      --FRAE-KIMQDRLLSR---ENVSVVWNSVI-DEILGTEARP-----------------P
BRUC201446      --FRAE-KIMQDRLLSR---ENVSVVWNSVI-DEILGTEARP-----------------P
BRUMC01440      --FRAE-KIMQDRLLSR---ENVSVVWNSVI-DEILGTEARP-----------------P
BRUMB01421      --FRAE-KIMQDRLLSR---ENVSVVWNSVI-DEILGTEARP-----------------P
BRUM501487      --FRAE-KIMQDRLLSR---ENVSVVWNSVI-DEILGTEARP-----------------P
BRUO201285      --FRAE-KIMQDRLLSR---ENVSVVWNSVI-DEILGTEARP-----------------P
RHILO01973      --FRAE-RILRERLLQK---DNVRVIWDTVV-DEITGRPGKA-----------------P
CHESB02097      --FRAE-RILQERLLRK---ENVEIMWDTVV-DEILGETPKP-----------------P
METPB00996      --FRAE-RILQERLFKH---ANVSVKWHHAV-EEICGSDTP-------------------
METEP01063      --FRAE-RILQERLFKH---ANVTVKWHHAV-EEICGSDTP-------------------
METEA00810      --FRAE-RILQERLFKH---ANVTVKWHHAV-EEICGSDTP-------------------
METED01453      --FRAE-RILQERLFKH---ANVTVKWHHAV-EEICGSDTP-------------------
METS403554      --FRAE-RILQERLFRH---PNVEVVWNHTV-EEICGRESP-------------------
METNO05482      --FRAE-RILQERLFRH---PNIEVVWNHTV-EEICGRESP-------------------
METSZ03234      --FRSE-RVLVKRLLAQ---ENVSILFNHVI-DEIVGDENP-------------------
BEII900056      --FRAE-RILQERLFKQ---DNVEVLFNQAI-EEICGTDQP-------------------
METSB02743      --FRAE-RILQERLFKR---PNVEIIWDHVV-DEIHGSDNP-------------------
MAGMM00401      --LRAE-KIMQDHMFAK---ENIIPVWDSVP-DEMLGDPNK-------------------
HYPNA00542      --FRAE-KILQDRLFKN---PKVEVIWNHAL-EEVVGDENP-------------------
KETVY00902      --LRAE-KILQERLFNH---PKVEVIWNHEI-VEVLGETNP-------------------
KETVW00472      --LRAE-KILQERLFNH---PKVEVIWNHEI-VEVLGETNP-------------------
```

```
ROSDO03242      --LRAE-KILIDRLMKN---PKIEPLWFHEL-DEVVGTENP-------------------
ROSLO02615      --LRAE-KILIDRLMKN---PKIEPLWFHEL-DEVVGTENP-------------------
RUEPO00888      --LRAE-KILQDRLFKN---EKIVPLWFNQL-EEVYGTDAP-------------------
RUEST00613      --LRAE-KILIDRLMKN---EKIEPLWFHEL-DEVVGTDAP-------------------
PHAIB02390      --LRAE-KILQDRLMKN---PKIEPLWFHQL-EEVVGTENP-------------------
PARDP02130      --LRAE-KILQERLFRN---PKVSVLWDHEL-VEVQGEQQP-------------------
DINSH02620      --LRAE-KILQDRLFKN---PKVEVIWDHTV-EEVVGTDTP-------------------
RHOCB02768      --FRAE-KILIERLKKN---PKIEVIWNATV-EEVLGTEAP-------------------
RHOS500248      --FRAE-KILQERLFKN---PKIEVIWNHTI-EEVLGTEAP-------------------
RHOS400150      --LRAE-KILQDRLFKH---PKIEVLWNHTI-EEVAGTEAP-------------------
RHOS100227      --LRAE-KILQDRLFKH---PKIEVLWNHTI-EEVAGTEAP-------------------
RHOSK02952      --LRAE-KILQDRLFKH---PKIEVLWNHTI-EEVAGTEAP-------------------
MIDMI00790      --LRAE-KISQDRLFNN---PKIKVVRNTVI-EEVLGAEDP-------------------
ACEP301595      --LRSE-KILQDRLFAN---PKVSVIWNSVV-EDILADGTP-------------------
MICAA01566      --LRAE-KIAQDRLFKN---PKVEVIWDSVV-EDILGDNSA-------------------
TISMK03676      --LRAE-KMLQERLFAN---PKIEMIWNAVV-DGYLAGGEP-------------------
AZOL402409      --FRAE-RIMQDRLFRH---PKIEVVWDSTV-EEIVGEGDGT----------------MG
PSEUV04348      --LRSE-KILQDRLFKN---EKIEVIWDSQV-EEILGTESP-------------------
HIRBI01095      --LRAE-RILQERLLNH---EKVEVVWDHQL-EEVVGDENP-------------------
PARL102241      --FRAE-KVMQERLFNH---PKIEVIWDSAI-DEILGTENP-------------------
MARMM02139      --LRAE-KVMQDRLFKH---EKIEIVWDSEV-EEVLGDSDP-------------------
PHEZH02588      --LRAE-RILQERLFNH---EKIEVIWDSAI-DEVVGQTDP-------------------
CAUCR02826      --LRAE-KILQERLLAH---PKIEVIWDSVI-DEVLGQTDP-------------------
CAUCN02939      --LRAE-KILQERLLAH---PKIEVIWDSVI-DEVLGQTDP-------------------
CAUST00891      --LRAE-KILQERLLAH---PKIEVVWDSVI-DEVKGDTDP-------------------
PARBH01522      --LRCE-AILRQRALDH---PKIELLWNRQV-DEVLGSDNP-------------------
PELHB02084      --FSAE-KIMQQRLFKH---PKIEVRWNTVL-DEVRGDNMP-------------------
HYPDA03273      --LRAE-KILQERLFKN---PKIEVIWDSAV-EEVVGTTTP-------------------
HYPSM04642      --LRAE-KILQERLFKN---PKIDVIWDSVV-EEVIGAPSP-------------------
OLICO01107      --LRAE-RILQERLFKH---PKIKVVWDSEI-AEIVGSQDP-------------------
OLICM02790      --LRAE-RILQERLFKH---PKIKVVWDSEI-AEIVGSQDP-------------------
RHOPS01446      --FRAE-RILQDRLFKN---PKIKVIWDSAI-DEICGSDNP-------------------
RHOPA04062      --FRAE-RILQDRLFKH---PKIKVIWDVAV-DEICGAENP-------------------
RHOPT04533      --FRAE-RILQDRLFKH---PKIKVIWDVAV-DEICGAENP-------------------
RHOPX04235      --FRAE-RILQDRLFKH---PKINVIWDVAV-EEICGAENP-------------------
BRADU07321      --FRAE-RILQERLFKH---PKIKVVWDSAV-DEICGTENP-------------------
BRASO01239      --FRAE-RILQERLFKH---PKIKVVWDSAL-DEICGASSP-------------------
BRASB06352      --FRAE-RILQERLFKH---PKIKVVWDSAL-DEICGASNP-------------------
RHOPB03773      --FRAE-RILQERLFKN---PKIEVVWDVAV-DEICGDESP-------------------
NITWN02324      --FRAE-RILQDRAFKH---PKIKVIWDSAI-DEICGSEDP-------------------
NITHX02707      --FRAE-RILQDRAFKH---PKIKVVWDSAL-DEICGTEDP-------------------
AZOC501143      --LRAE-RILQDRLFAH---PKVSVVWNAAI-DEILGVSEPV------------------
XANP202679      --FRSE-KILQERLFAH---PKVKVVWNAEL-AEVVGQDDPF------------------
CHLTF01983      --LRAS-KIMQARAFAN---PKITFLWETVV-EEVYGQRE-------------------T
IGNAJ02236      --FRAS-KIMLDRAKKN---PKIKFVTNKVI-KEVLGVEE-------------------G
MELRP00403      --FRAS-KIMLERARKN---PKIKFITNAVI-KEILGKEE-------------------D
ANADF00468      --LRAS-KIMQERAQKN---PKVELVLESVV-DEILGDG---------------------
CHLCH01125      --FRSS-KIMSLRVTKH---PKIEMLLNQVV-EEVLGDG---------------------
PELPB01419      --FRSS-KIMSLRTRKN---PKISMMLNEVV-DEILGDG---------------------
CHLL200781      --FRAS-KIMSLRAGKN---PKIATILNVVV-DEILGDG---------------------
CHLTE00830      --FRAS-KIMSLRASKN---EKITTMLNQVV-DEILGDD---------------------
CHLP800854      --FRAS-KIMSLRASKN---EKITTMLNEVV-DEILGDG---------------------
CHLL701225      --FRAS-KIMSLRASKN---PKIDTMLNVVV-DEILGDG---------------------
CHLPM00993      --FRSS-KIMSLRASKN---PKITTMLSRTV-DEILGDG---------------------
WOLTR00562      --LRAE-KIMQDRLFAN---NKIKVIWNHTV-EQVLGEEN--------------------
WOLPP00135      --LRAE-KVMQDRLFKN---DKIKVIWNHTV-EQVLGEEN--------------------
WOLPM00650      --LRAE-KVMQDRLFKN---DKIKVMWNHTV-EQILGEEN--------------------
WOLWR00416      --LRAE-KVMQDRLFKN---DKIKVMWNHTV-EQILGEEN--------------------
EHRCR00696      --LRAE-PIMQERLFSN---DKIQVIWNSVV-EEILGNKE--------------------
ANAMM00345      --LRAE-AVMQNRLFEN---SKIEVVWNSVV-KEILGDKE--------------------
ANAMF00341      --LRAE-AVMQNRLFEN---SKIEVVWNSVV-KEILGDKE--------------------
ANAPZ00610      --LRAE-AVMQQRLFSN---SKIEVIWNSVV-KEILGDSE--------------------
NEOSM00537      --LRAE-TVLQERLFAN---AKVELIWNSVL-DEILGRDS--------------------
NEORI00513      --LRAE-AVLQERLFAN---AKVELIWNSVL-DEVLGRDS--------------------
PELUB00076      --LRAE-KLLQKKLMEN---KKIEIIWDSAV-EEVIGDSE--------------------
```

```
PELSM00736      --LRAE-KLLQQKILSH---PKINIIWDSAV-KEIIGTDN--------------------
PUNMI01409      --LRAE-QIMQDRLFKK---DNISVEWNREV-AEVLGDDG--------------------
ASTEC00651      --VRSE-KILAERMYAE---PKIQPVWNSAI-DEILGETNE------------------F
ZYMMT00154      --LRAE-KILQRRLLAN---PAIRIQWNSVV-SEFIAGGD--------------------
ZYMMO00984      --LRAE-KIMQKRLLAN---PKIKIRWNSEV-AEFIAGED--------------------
ZYMMA00176      --LRAE-KIMQKRLLAN---PKIKIRWNSEV-AEFIAGED--------------------
ZYMMN00183      --LRAE-KIMQKRLLAN---PKIKIRWNSEV-AEFIAGED--------------------
SPHAL00143      --LRAE-KILQDRLFAN---PKIKVLWNKQV-ERFVAGEG--------------------
SPHWW03978      --LRAE-KILQDRLFAH---PNVKVLWNKQV-DRFVGGSG--------------------
SPHJU02313      --LRAE-KILQQRLFAH---PNIRVLWNQAV-ERFVGGGN--------------------
NOVAD02319      --LRAE-KILQDRLFAH---PNVKVLWNQKV-DSFVGEAG--------------------
ERYLH00934      --LRSE-KILQERLFKS---DKISTLWNKTV-ESFEAGED--------------------
GRABC00720      --LRAE-KILQDRLFAH---PKISVVWNSVV-EDITASGP--------------------
GLUDA03075      --LRAE-KILQDRLFAH---PKITVKWNCVV-DEIVATGT--------------------
KOMMN00774      --LRAE-KILQDRLHAN---PKISVIWNSAV-ERITGNGT--------------------
HALVD01097      --FRAE-DYWVDRLMEKVDEGEIELMLNTEA-TELHGSPEE-------------------
HALHT01699      --FRAE-DYWIDRVQQKVDEGEIEIMRNTEL-LEMHGSPEN-------------------
METI401038      --LRAS-PIMAERVFN---EPRIKPIWNSAV-EEILDHKQN-------------------
PLAL201612      --FRAS-KIMAARVLD---NPKIQVEWSSAV-TEVLGDDQH-------------------
GEMAT01983      --FRAS-KVMANRVLT---HPKVRVIWNSRV-TEVVGSDF--------------------
CYAAP02456      --LRAS-KTMQERLFA---HTQIKIHWHTVP-VVAYGDEI--------------------
CHLPN00303      --LRAS-KAMEARAQN---NEKITFLWNSEI-VKISGDSI--------------------
CHLPP00439      --LRAS-KAMEARAQN---NEKITFLWNSEI-VKISGDSI--------------------
CHLPE00727      --LRAS-KAMENKARA---NDKITFLWNSEI-IRISGDSL--------------------
CHLTR00101      --LRAS-KVMVNKAQA---NEKIFFLWNSEI-VKISGDTL--------------------
CHLTA00102      --LRAS-KVMVNKAQA---NEKIFFLWNSEI-VKISGDTL--------------------
CHLTJ00101      --LRAS-KVMVNKAQA---NEKIFFLWNSEI-VKISGDTL--------------------
CHLTD00098      --LRAS-KVMVNKAQA---NEKIFFLWNSEI-VKISGDTL--------------------
CHLT700102      --LRAS-KVMVNKAQA---NEKIFFLWNSEI-VKISGDTL--------------------
CHLT000101      --LRAS-KVMVNKAQA---NEKIFFLWNSEI-VKISGDTL--------------------
CHLT500104      --LRAS-KVMVNKAQA---NEKIFFLWNSEI-VKISGDTL--------------------
CHLTL00098      --LRAS-KVMVNKAQA---NEKIFFLWNSEI-VKISGDTL--------------------
CHLTG00102      --LRAS-KVMVNKAQA---NEKIFFLWNSEI-VKISGDTL--------------------
CHLTS00101      --LRAS-KVMVNKAQA---NEKIFFLWNSEI-VKISGDTL--------------------
CHLT900102      --LRAS-KVMVNKAQA---NEKIFFLWNSEI-VKISGDTL--------------------
CHLTZ00101      --LRAS-KVMVNKAQA---NEKIFFLWNSEI-VKISGDTL--------------------
CHLT400432      --LRAS-KVMVNKAQA---NEKIFFLWNSEI-VKISGDTL--------------------
CHLT100103      --LRAS-KVMVNKAQA---NEKIFFLWNSEI-VKISGDTL--------------------
CHLT200350      --LRAS-KVMVNKAQA---NEKIFFLWNSEI-VKISGDTL--------------------
CHLTB00350      --LRAS-KVMVNKAQA---NEKIFFLWNSEI-VKISGDTL--------------------
CHLTC00378      --LRAS-KVMVNKAQA---NEKIFFLWNSEI-VKISGDTL--------------------
SIMNZ00831      --LRAS-KIMAKHALE---HPKIEIIWNHVV-KKVEGGNV--------------------
PARUW01706      --LRAS-KIMQERAFN---NPKIEMIWNSEV-IQVKGDQI--------------------
WADCW00943      --LRAS-KIMQERAMN---HPKITILWDSVL-TKVEGDHV--------------------
SINAD04980      --LRAS-KIMQKRIFE---NPKVEYAWNSGV-HSYLGVDEPHRSL------TG-------
BIFLB01558      --FRAS-QIMVERTRE---NPKINLLMNTVV-ERINGDGK--------------------
BIFAB00498      --FRAS-QIMVERTRE---NPKINLLMNTVV-ERINGDGK--------------------
BIFAV01561      --FRAS-QIMVERTRE---NPKINLLMNTVV-ERINGDGK--------------------
BIFAS01557      --FRAS-QIMVERTRE---NPKINLLMNTVV-ERINGDGK--------------------
BIFA001518      --FRAS-QIMVERTRE---NPKINLLMNTVV-ERINGDGK--------------------
BIFBA01814      --FRAS-QIMVDRAQS---NPKLTLLTNTVV-TAIHGSAPEAKPASPAITIGGLKLPAAT
BIFAA01619      --FRAS-RIMVERAQR---NPKIDFMLDAVV-QEIRGDEN--------------------
BIFDB02112      --FRAS-QIMVERAKN---NPKIDFILNSVI-EEINGNDN--------------------
TERSS03091      --FRAS-KIMLERAMA---HEKIVMMTNTVV-EEVLGVEEK-------------------
GRATM03160      --FRAS-PIMLERAQA---HDKIHFLHNTIV-EEVLGVEEK-------------------
GRAMM03836      --FRAS-RIMLERAIA---HPQIEFLHNTLV-EECLGVEEK-------------------
AKKM801247      --LRAS-KIMAERTLS---NEKIFPMWNSTI-VSYKTDDKG-------------------
OPITP04130      --LRAS-KIMAARATS---HEKIVPVWNSIP-LAVEGVEQG-------------------
CORAD01794      --LRAS-KIMADRAVN---HEKIEMAWNSLP-QEVLADEQG-------------------
BUCCC00180      --FKAD-KILIDRLYKKMKEKNIIIYFDSII-TEILDYNNN-------------------
CENSY00347      --LRAS-KIMQERAHDD---EKIRFHLGYEV-KEIRGNGK--------------------
NITMS00668      --LRAS-KVMQDRAHSN---EKIKFHWDSAV-VDIKGDQK--------------------
MEIRD02823      --LRAN-KTAQARALAH---PKMQFIWDTVV-EEVLGEET--------------------
HERA203780      --LRAD-PVLIERAQNN---PKIKFIWDTVV-TKVEGTVK--------------------
CHLAA02018      --LRAD-PILQERAFSN---PKVRFIWNSVV-VSINGKDK--------------------
```

CHLSY02175	--LRAD-PILQERAFSN---PKVRFIWNSVV-VSINGKDK--------------------
CHLAD02599	--LRAD-PVLQERAFNN---PKVRFIWNSTV-VAINGKDK--------------------
CALAS01745	--FRAS-KILQDRVFAN---EKIEVIWNTVV-TEIFGENGT-------------------
ANATU01696	--LRAG-AILQSRARSN---PKIQFIWNTVV-TEILGEDA--------------------
SULMS00235	--MRAS-KIMQKKISNI---KNIKLLFNFEI-KKIIGKKK--------------------
BLASB00041	--LKAS-KILQFHLSKR---SNVNVLFCSQI-TKIIGNDF--------------------
BLASP00551	--FRAS-KALQYRISKI---NNVNIFFCSQI-TKIMGNDF--------------------
AZOPC00437	--LRAS-QIMQTRVLSN---TKIEVYFNTNV-IGLFGEEF--------------------
LEPBD01878	--LRAS-AIMQKRAKDN---SKIEWKLDYTP-KKVLADEK--------------------
SALRD02577	--LRAS-EIMQQRAFEN---DKIEFVWNTEV-IDVLGESA--------------------
SALRM02866	--LRAS-EIMQRRAFEN---DKIEFVWNTEV-IDVLGESA--------------------
RIEPU00166	--FRAE-KLLIDKIYKKVNQKKVFLYTNYVV-CDAEGN----------------------
ORITB00015	NGLKAE-AILQKRLFKKKQEGKITIIWNSTI-EEILGNDNG-------------------
ORITI01234	KGLKAE-AILQKRLFKKKQEGKFTIIWNSTI-EEILGNDNG-------------------
PREMB01541	--LRAA-EIMRKRVTEK---ENIEILYNTNT-LGLFGE----------------------
PREDF01155	--LRAA-EIMRKRVTEK---ENIEILYNTNT-IGLYGE----------------------
PREI702011	--LRAA-EIMRKRVEEK---ENIEILYNTNT-LGLFGE----------------------
ALIFI01035	--LRAS-KAMQERVFNT---TNIEVMFNHNT-AEVLGD----------------------
ODOSD00362	--LRAS-KAMQQRVFNT---ENIEVLFEHNT-LELYGS----------------------
PRER201250	--LRAS-QVMQQRVKEN---GNIEILFEHNT-LGLYGE----------------------
PALPW00206	--LRAS-KIMQERVLSN---PKIEVLFEHET-LGLTGE----------------------
PORGI00953	--LRAS-KVMQERVMNT---ANITVLFEHNT-VGLFGE----------------------
PORG301168	--LRAS-KVMQERVMNT---ANITVLFEHNT-VGLFGE----------------------
BACV803426	--LRAS-KIMQARVMNH---PNIKVLFEHNA-VGLYGE----------------------
BACT601486	--LRAS-KIMQERVMNH---DKIEVLFEHNA-VGLYGD----------------------
BACTN04290	--LRAS-KIMQERVQKH---EKIEVLFEHNV-VGLFGD----------------------
BACFR01032	--LRAS-KIMQERVRKH---DKIEVLFEHNV-VGLFGE----------------------
BACFN00913	--LRAS-KIMQERVRKH---DKIEVLFEHNV-VGLFGE----------------------
BACF600963	--LRAS-KIMQERVRKH---DKIEVLFEHNV-VGLFGE----------------------
OWEHD03413	--WKAS-KIMEERVMKT---PNIEVLFNTDT-VEVVGE----------------------
PSYTT00390	--MKAS-KAMQHRVEKA---KNIDLRYYSEI-DEVLGE----------------------
NONDD00011	--MRAS-KAMQHRVNSL---ENIKVLYNSEV-DEVLGD----------------------
ROBBH02711	--FRAS-KAMQNRVMSL---ENIDIRFNTEV-DEVLGD----------------------
CELAD02592	--MRAS-KAMQHRVHNI---KNITVLYNTEV-DEVLGG----------------------
CELLC02474	--MRAS-KAMQHRVNSI---SNITVKYNTEV-DEVLGE----------------------
MARSH03072	--MRAS-KAMQHRVNSL---PNLEVKYNTEV-DEVLGE----------------------
MURRD00159	--MRAS-KAMQHRVNSL---DNIEIRYNTEV-DEVLGE----------------------
ZOBGA00152	--MRAS-KAMQHRVQSI---KNIEIRYNTEV-DEVLGD----------------------
GRAFK00949	--MKAS-KAMQHRVENT---KNIDLRYNTEV-DEILGE----------------------
ZUNPS02344	--MRAS-KAMQHRVNNT---KNLEVRYNTEV-DEVIGD----------------------
AEQSU02079	--MRAS-KAMQHRVNNT---KNLEVLYNTEV-DEVLGD----------------------
HALH105745	--LRAS-KIMQERVLNA---ENIVIHWNSET-LEVLGE----------------------
SAPGL02893	--LRAS-KIMQERIEKA---ENVEVHWNTEI-EEILGD----------------------
AMOA500014	--MRAS-HIMQQRLISK---PNVSIFFNTGI-EEILGD----------------------
FLAIG01802	--FRAS-RIMEERVRKT---ENIDILVNTST-VEVLGD----------------------
FLACA02326	--FRAS-KIMEERVRKT---ENIQILMHTET-EEVLGD----------------------
FLAJ100198	--FRAS-KIMEERVRKT---ENIEILMNHDT-LEVLGD----------------------
FLABF01528	--FRAS-KIMEERVRKT---ENITILMHHDT-VEVQGD----------------------
CAPOD01333	--FRAS-QIMKERVLNS---PKIEVLFNTET-EEILGD----------------------
CAPCC00414	--FRAS-KIMENRVKKA---QNIEILFNTET-QEILGD----------------------
FLELS01898	--LRAS-KIMQQRVMTK---ENIEILWNSQT-EEILGD----------------------
SOLCM01166	--MRAS-KAMQHRVLNT---PNIEVLYHSET-KEILGD----------------------
PEDHD01598	--FRAS-KAMVHRVLNT---PNIEVIYNTET-REILGN----------------------
SPHS203207	--FRAS-KAMQHRVFNT---PNIEIHYNTEA-EEILGD----------------------
LEAB401188	--FRAS-QIMQERVKNT---PNIEILFNTET-VEILGD----------------------
EMTOG00092	--FRAS-LIMQERVKNT---PNIEVLFNTET-LEVLGK----------------------
SPILD01737	--MRAS-QFMQKRVKTA---HNIEILYNTST-EEVLGD----------------------
DYAFD00084	--MRAS-KIMQKRLETL---PNIEILWNTET-VKLNGE----------------------
CYCMS03063	--LRAS-QIMQNRVMKN---PKIEILWNHET-VEILGD----------------------
ECHVK03326	--MRAS-QIMQKRVTSN---PKIEILWNTET-EEILGE----------------------
BELBD02542	--MRAS-QIMQKRVMNN---PKIEILWNTET-EEILGD----------------------
NITGG02112	--LRAS-KILQEKAMEN---PKIEFIWNNVV-YDIKGD----------------------
THEM700111	--LRAS-KIMQERAMAH---DRIRFVWDTVV-EDILGE----------------------
CREAS01350	--LRAS-KILQERAFKN---PKIKFIWSTVV-EEIVGD----------------------
TURPD02772	--FRAS-KIMIDRARAN---PKIVIKTFVEV-AEVYGEAQ--------------------

```
SORC507603      --FRAS-KAMIQRVVEN---PKIKILYSHVV-EEVLDV-K--------------------
BDEBA00337      --FRAS-KIMVDRALKN---PKIEVLYNTEV-IEVLGD-G--------------------
STIAD02752      --LRAS-KIMQERVLKN---PKITVMWNSAV-DEVVGN-E--------------------
MYXXD01876      --LRAS-KVMQERARQN---PKISFMWDSAV-EEVLGD-A--------------------
MYXFH03454      --LRAS-KVMQERALQN---PKISFMWDSAV-EEVLGD-N--------------------
CORCM01960      --LRAS-KVMQERALNN---PKISFMWDSAV-EEVVGD-A--------------------
MYXSD02209      --LRAS-KVMQDRALKN---PKISFLWNSAV-EEVMGD-K--------------------
LEPFC02126      --LRAS-KAMQDRAHAN---EKIRFAWNKEV-LEVLDV-S--------------------
LEPFM02294      --LRAS-KIMQERAKAN---KKISFLWNKEV-VEVRDV-S--------------------
SOLUE00582      --FRAS-KIMLDRAQHN---PKIKFLLNTEV-EEVHDV-S--------------------
KORVE01333      --FRAS-KIMLDRARKH---EKISMITDTVV-EEVHDP-A--------------------
ACIC502553      --FRAS-RIMLERAMAH---PKIEFRTNVSV-EEVLGV-E--------------------
ACIFD00030      --LRAS-RIMQQRAFAN---EKIRFAWNRRV-VELLER----------------------
HALMS01402      --LRAS-KPMQERAFNN---EKIEFVWDSAV-TEIIAD----------------------
CHLPD01133      --FRAS-KIMSLRAGKN---PKISTMLNQVV-DEILGD----------------------
CHLPB01137      --FRAS-RIMSMRVEKN---PKIALELNKVV-EEILGD----------------------
PROA200897      --FRAS-KIMSLRAEKN---EKISVELNQVV-EEILGD----------------------
WIGBR00492      --FTAE-KILVDRIFKKRNNGNISIYNNFVV-KKIIGNDK--------------------
BUCA500289      --FRAE-KILLDRLEKKIKSQKIIIYLNSIV-KNILGNSS--------------------
BUCAI00292      --FRAE-KILLDRLEKKIKSQKIIIYLNSIV-KNILGNSS--------------------
BUCAF00306      --FRAE-KILLDRLEKKIKSQKIIIYLNSIV-KNILGNSS--------------------
BUCAT00287      --FRAE-KILLDRLEKKIKSQKIIIYLNSIV-KNILGNSS--------------------
BUCA000302      --FRAE-KILLDRLEKKIKSQKIIIYLNSIV-KNILGNSS--------------------
BAUCH00290      --FRAE-PIIMDRLMYKVNFGNIIIHKNKIV-QNILGDKS--------------------
BLOVB00369      --FTSE-KILIDRLMEKVYKGNIVLHTNCIV-KTILGNDL--------------------
BLOFL00367      --FRAE-KILMDRLMYKVNHENITVHTNCIV-QEILGNEK--------------------
BLOPB00375      --FSAE-KILINRLMNKVHSGNVVLHTNHIV-KEILGNGT--------------------
BUCAP00289      --FKAE-KILIDRLLKIVKTKKVILHLNSTI-EDILGNNK--------------------
COXBU01001      --LRAE-KMLSAQLIKKVEEGKVAIVWSHVI-EEVLGDDQ--------------------
COXBN01188      --LRAE-KMLSAQLIKKVEEGKVAIVWSHVI-EEVLGDDQ--------------------
COXBR01117      --LRAE-KMLSAQLIKKVEEGKVAIVWSHVI-EEVLGDDQ--------------------
COXB200717      --LRAE-KMLSAQLIKKVEEGKVAIVWSHVI-EEVLGDDQ--------------------
COXB100914      --LRAE-KMLSAQLIKKVEEGKVAIVWSHVI-EEVLGDDQ--------------------
FRAP200241      --LRSE-KILIDKLMEKAENGNVNIIWDTTL-EEVLGDDM--------------------
FRANT01012      --LRSE-KILIDKLMEKAENGNVNIIWDTTL-EEVLGDDM--------------------
FRACN00522      --LRSE-KILIDKLMEKAENGNINIIWDTTL-EEVLGDDM--------------------
FRATT00465      --LRSE-KILIDKLMEKAQHGNINIIWNTTL-EEVLGDDM--------------------
FRAT100465      --LRSE-KILIDKLMEKAQHGNINIIWNTTL-EEVLGDDM--------------------
FRATE00462      --LRSE-KILIDKLMEKAQHGNINIIWNTTL-EEVLGDDM--------------------
FRATW01216      --LRSE-KILIDKLMEKAQHGNINIIWNTTL-EEVLGDDM--------------------
FRATM01110      --LRSE-KILIDKLMEKAQHGNINIIWNTTL-EEVLGDDM--------------------
FRACF00566      --LRSE-KILIDKLMEKAQHGNINIIWNTTL-EEVLGDDM--------------------
FRATO01212      --LRSE-KILIDKLMEKAQHGNINIIWNTTL-EEVLGDDM--------------------
FRATH01486      --LRSE-KILIDKLMEKAQHGNINIIWNTTL-EEVLGDDM--------------------
FRATF01216      --LRSE-KILIDKLMEKAQHGNINIIWNTTL-EEVLGDDM--------------------
FRATN00564      --LRSE-KILIDKLMEKAQHGNINIIWNTTL-EEVLGDDM--------------------
ACIF500512      --FRAE-KILQDKLMAKE---NVTVIWDHSV-AEVLGDAS--------------------
ACIF200362      --FRAE-KILQDKLMAKE---NVTVIWDHSV-AEVLGDAS--------------------
DECAR01284      --FKAE-KIMQDKLFQREKEGKVTILWNSTL-DEVLGDDS--------------------
NEIG100525      --FRAE-KIMIDKLMKRVEEGKIILKLESNL-QEVLGDDR--------------------
NEIG201314      --FRAE-KIMIDKLMKRVEEGKIILKLESNL-QEVLGDDR--------------------
NEIM800926      --FRAE-KIMIDKLMKRVEEGKIILKLESNL-QEVLGDDR--------------------
NEIMP01199      --FRAE-KIMIDKLMKRVEEGKIILKLESNL-QEVLGDDR--------------------
NEIMB01212      --FRAE-KIMIDKLMKRVEEGKIILKLESNL-QEVLGDDR--------------------
NEIMF01143      --FRAE-KIMIDKLMKRVEEGKIILKLESNL-QEVLGDDR--------------------
NEIML01119      --FRAE-KIMIDKLMKRVEEGKIILKLESNL-QEVLGDDR--------------------
NEIMM00780      --FRAE-KIMIDKLMKRVEEGKIILKLESNL-QEVLGDDR--------------------
NEIMH00827      --FRAE-KIMIDKLMKRVEEGKIILKLESNL-QEVLGDDR--------------------
NEIMG01168      --FRAE-KIMIDKLMKRVEEGKIILKLESNL-QEVLGDDR--------------------
NEIMN01254      --FRAE-KIMIDKLMKRVEEGKIILKLESNL-QEVLGDDR--------------------
NEIMO00810      --FRAE-KIMIDKLMKRVEEGKIILKLESNL-QEVLGDDR--------------------
NEIM701229      --FRAE-KIMIDKLMKRVEEGKIILKLESNL-QEVLGDDR--------------------
NEIMA01334      --FRAE-KIMIDKLMKRVEEGKIILKLESNL-QEVLGDDR--------------------
NEIMW01125      --FRAE-KIMIDKLMKRVEEGKIILKLESNL-QEVLGDDR--------------------
DICNV01037      --FRAE-KIMVDRLMEKVNAGKIVVKYSAQL-QEVLGDDN--------------------
```

```
VESOH00047      --FSSE-KILSDQLIEKARTGNITIEYNHNL-DEVLGDVN--------------------
RUTMC00043      --FSSE-KILSNQLIEKAKIGNITIEYNHNL-DKVLGDTM--------------------
HALHL02255      --LRAE-KILQRQLMERVEQGRVDICWNRAL-DEVVGDDS--------------------
PELPD03177      --FRAE-KYLANLIIEKSKNGNITIEWDHVL-DEVLGDAS--------------------
GEOS804030      --FRAE-KILTDRLIEKTKNGNVTIEWNHQL-DEVLGDDS--------------------
GEOBB03636      --FRAE-KILTDKLIEKTKNGNVTIEWNHHL-EEVLGDES--------------------
GEOSM03700      --FRAE-KILADKLIEKTKNGNVTIEWNHHL-EEVLGDES--------------------
HALNC00046      --FKAE-NILIDRLMKKVDEGKVEIHWHSAI-DEILGDNT--------------------
HAEPS00887      --FRSE-KILIDRLMKKVEEGKVILHTHSVL-DEVLGDNM--------------------
GALAU02285      --FRSE-KILLDRLMKKVEEGKIVLHTNRTL-AEVCGDNM--------------------
HISS201000      --FRAE-KILIDRLNKKVEEGKIILHTNRTL-AEVLGDNM--------------------
HAES101151      --FRAE-KILIDRLNKKVEEGKIILHTNRTL-AEVLGDNM--------------------
PASMU00573      --FRSE-KILIDRLMKKVEEGKIILHTNRTL-DEVLGDNM--------------------
PASMH00579      --FRSE-KILIDRLMKKVEEGKIILHTNRTL-DEVLGDNM--------------------
ACTSZ01560      --FRAE-KILLGRLQKKVEEGKIILHTNRTL-DEVLGDNM--------------------
AGGAN00985      --FRAE-KILIDRLYKKVEEGKIILHTNRTL-DEVLGDDM--------------------
NITHN03181      --FRAE-KILANRLLKKATESNVAIEWNHVL-EEVLGDDA--------------------
NITOC00311      --FRAE-KILANRLLQKAAEGNVTIEWNHTL-EEVLGDEA--------------------
NITWC02498      --FRAE-KILANRLLQKAAKGNVTIEWNHVL-EEVLGDEA--------------------
METNJ00648      --FRSE-KILSNQLLKKAETGNVEILWNHTL-DEVLGDDA--------------------
METFJ01616      --FRAE-KILINQLMKKAESGNVEILWNSTL-DEVLGDDA--------------------
ALKEH00244      --LRAE-KILQRQLMERVEAGKVSIVWDHNL-DEVLGDDA--------------------
MARMS03166      --FRSE-KILADKLMEKAENGNVKIEWYSEL-DEVLGDDA--------------------
MARM102688      --FRSE-KILSDKLLEKAENGNVKIEWNSQL-DEVLGDDA--------------------
THICR00763      --FSSE-KILSKQLLEKAENGNINIEFNSAL-EEVLGDKM--------------------
THICA00974      --FSSE-KILSDQLIKKSKEGNVKIIYNSVV-DDVLGDKT--------------------
THIV600851      --LRAE-KILQRQLLEKADKGNIKLAWNQTL-DEILGDAT--------------------
THISH01973      --LRSE-KILQDHLFEKEKEGKVTIEWNHVL-DEVLGDDS--------------------
METAA00813      --FRSE-KILSEKLIEKSKNGNVVIEWDHTL-DEVLGDDM--------------------
META200813      --FRSE-KILSEKLIEKSKNGNVVIEWDHTL-DEVLGDDM--------------------
METMM02895      --FRSE-KILSDKLLEKANSGNVRIEWNHNL-DEVLGDDM--------------------
FRAAD00813      --LRAE-KIMQDKLFEKAAAGKVELVWNSEI-DEVLGDDS--------------------
XYLFA01416      --LRAE-KIMQNKLFSKAATGKIELIWNNAV-EEVLGNDA--------------------
XYLFT00623      --LRAE-KIMQNKLFSKAATGKIELIWNNAV-EEVLGNDA--------------------
XYLF200667      --LRAE-KIMQNKLFSKAATGKIELIWNNAV-EEVLGNDA--------------------
XYLFG01622      --LRAE-KIMQNKLFSKAATGKIELIWNNAV-EEVLGNDA--------------------
XYLFM00690      --LRAE-KIMQNKLFSKAATGKIELIWNNAV-EEVLGNDA--------------------
PSEUP01680      --LRAE-KIMQDKLQAKIDTGKIVPVWHHTV-EEVLGDEA--------------------
STRM501972      --LKAE-KIMQDKLFAKVAAGKIETVWHHQV-EEVLGNEA--------------------
PSEUU01524      --LRAE-KIMQDKLFAKVEAGKIEPVWHHTI-DEVLGDDA--------------------
XANAP01407      --LRAE-KIMQDKLFAKVAAGKIETVWHHAV-DEVLGNDA--------------------
XANCP01918      --LRAE-KIMQDKLFAKVAAGKIETVWHHAI-EEVLGNDA--------------------
XANC802154      --LRAE-KIMQDKLFAKVAAGKIETVWHHAI-EEVLGNDA--------------------
XANCB02263      --LRAE-KIMQDKLFAKVAAGKIETVWHHAI-EEVLGNDA--------------------
XANOR02379      --LRAE-KIMQDKLFAKVAAGKIETVWHHAI-DEVLGNDA--------------------
XANOM02295      --LRAE-KIMQDKLFAKVAAGKIETVWHHAI-DEVLGNDA--------------------
XANOP02318      --LRAE-KIMQDKLFAKVAAGKIETVWHHAI-DEVLGNDA--------------------
XANAC01951      --LRAE-KIMQDKLFAKVAAGKIETVWHHAI-DEVLGNDA--------------------
CYCSP01155      --FRSE-KILSDKLMKKVADGNISVEWFNQL-DEVLGDDM--------------------
GEOLS03275      --FRSE-KILADKLKARAAEGKVTIEWFHVL-DEVLGDAM--------------------
GEOUR03858      --FRSE-KILADKLIEKTKGGNVTIEWHHNL-DEVLGDET--------------------
GEODF00692      --FRAE-KILADRLIEKTKGGNVTIEWHHTL-DEVLGDES--------------------
GEOSL00482      --LRAE-KILADKLIEKTRGGNVTIEWNHVL-DEVLGDQA--------------------
GEOSK00469      --LRAE-KILADKLIEKTRGGNVTIEWNHVL-DEVLGDQA--------------------
GEOMG02998      --FRSE-KILADRLIEKTKGGNVTIEWHHVL-DEVLGDAT--------------------
MORCR00228      --LRAE-KILQDQLFEKVKDGNIKIEWNHQI-KEVVGDDM--------------------
ACIAD00798      --LRSE-KILQDHLFAKEKEGKISIVWNHQV-DEVLGDTT--------------------
ACIBC00821      --LRSE-KILQDHLFAKEKEGKISIVWNHEV-EEVLGDNT--------------------
ACIBY02725      --LRSE-KILQDHLFAKEKEGKISIVWNHEV-EEVLGDNT--------------------
ACIB302691      --LRSE-KILQDHLFAKEKEGKISIVWNHEV-EEVLGDNT--------------------
ACIB500882      --LRSE-KILQDHLFAKEKEGKISIVWNHEV-EEVLGDNT--------------------
ACIB100850      --LRSE-KILQDHLFAKEKEGKISIVWNHEV-EEVLGDNT--------------------
ACIBD00843      --LRSE-KILQDHLFAKEKEGKISIVWNHEV-EEVLGDNT--------------------
ACIBS02229      --LRSE-KILQDHLFAKEKESKISIVWNHEV-EEVLGDNT--------------------
ACICP00137      --LRSE-KILQDHLFAKEKEGKISIIWNHEV-EEVLGDNT--------------------
```

```
ACISD03052      --LRSE-KILQDHLFAKEKEGKISIIWNHEV-EEVLGDNT--------------------
LEGLN02402      --LRAE-KILQDKLFEKTQNGNIKIIWNSTL-EEVIGDGK--------------------
LEGPA01726      --LRAE-KILQNKLFEKAHSGNIKIVWNSTL-DEVLGDEK--------------------
LEGPH00846      --LRAE-KILQDKLFEKAHSGNIKIIWNSTL-DEVLGDGK--------------------
LEGPC01171      --LRAE-KILQDKLFEKAHSGNIKIIWNSTL-DEVLGDGK--------------------
LEGP201857      --LRAE-KILQDKLFEKAHSGNIKIIWNSTL-DEVLGDGK--------------------
LEGPL01727      --LRAE-KILQDKLFEKAHSGNIKIIWNSTL-DEVLGDEK--------------------
COLP302668      --FRSE-KILTDRLYEKVANGNIVLHTDRTL-DEVLGDNM--------------------
KANKD01106      --LRSE-KILQDKLFEKAKNGNVVLHWHRTL-EEIVGDDM--------------------
IDILO00659      --FRAE-KILVDRLHEKEQNGNVTFHLNKTL-DEVLGDDA--------------------
PSEU901284      --FRSE-KILADRLAEKAANGNVVMHYNRTL-DEVLGDQM--------------------
PSEA602346      --FRSE-KILSQRLLDKAENGNVTLHLNSTL-DEVLGDQM--------------------
ALTSS01871      --FRSE-KILEQRLRDKAENGNVVLHLDRTL-DEVMGDEM--------------------
ALTMD01611      --FRSE-KILEQRLREKAENGNVVLHLNRTL-DEVLGDEM--------------------
ALTME01705      --FRSE-KILEQRLRDKAENGNVVLHLNRTL-DEVLGDEM--------------------
ALTMB01797      --FRSE-KILEQRLRDKAENGNVVLHLNRTL-DEVLGDEM--------------------
ALTMS01652      --FRSE-KILEQRLRDKAENGNVVLHLNRTL-DEVLGDEM--------------------
SACD201683      --LRSE-KILQERLFAKEKNGNVKIMWNQNL-DEVLGDDS--------------------
TERTT01748      --LRSE-KILQDRLLEKAENGNVKLMWNQTL-DEVLGDDS--------------------
SIMAS00609      --LRSE-KILQDKLFEKAKNGNVKLLWNHTL-EEVLGDDA--------------------
ALCDB01962      --LRAE-KILQDKLFAK---DNVTVMWDHVL-DEVLGDDS--------------------
CHRSD02939      --LRAE-KILQDKLFEKVENGNMEVIWNHTL-EEVLGDNT--------------------
HALED02811      --LRAE-KILQDKLFDKVENGNIEVIWNHTL-DEVLGDNT--------------------
SIDLE02655      --FRAE-RIMVDHLMEKVKEGKITLQLNQTL-DEVLGDDS--------------------
GALCS01621      --FRAE-RIMIDHLMEKVAEGKITLQLNSTL-DEVLGDAS--------------------
LARHH01446      --FRAE-KIMVDKLMEKVAAGKITLKLNAVL-DEVLGDAS--------------------
CHRVO02807      --FRAE-KILVDKLMSRVAEGKISLKLNATL-DEVLGDDS--------------------
PSEUL01648      --FRAE-KILVDHLMHKVEEGKITLALNQTL-DEVLGDDS--------------------
NITEU01859      --FRSE-KIMIDKLMGKVKSGKIELALDHVL-EEVKGDDS--------------------
NITEC00745      --FRSE-KILIDKLMEKVQSGKIELALDHVL-EEVKGDDS--------------------
NITMU00023      --FRSE-KIMIDKLMDKVKNGNIKLELHYVL-DEVLGDES--------------------
NITSI03064      --FRSE-KILIDKLMEKTRSGNIKLALNHVL-DEVLGDQS--------------------
ACCPU02128      --LRSE-KILQDKLFAREKAGKVTIRWNCLL-EEVLGDSS--------------------
THIDA01000      --FKAE-KILINNLMDKVKEGKITLALDSTL-DEVLGDKT--------------------
METS601221      --FKAE-AILVDKVMERVKEGKIVLELHSVL-EEVLGDDS--------------------
METGS01186      --FKAE-AILVDKVMERVKEGKIVLELHSVL-EEVLGDDS--------------------
METFK00972      --FKAE-AILVDKLMERVKEGRIVLETFQTL-DEVLGDDS--------------------
METML01428      --FKAE-AILVDKLMARVAEGKIVLETFATL-DEVLGDNT--------------------
AROAE03899      --FRAE-KILIDKMMEKVAAGKIELALNSTL-DEVLGDNT--------------------
THASP01686      --FRAE-KIMIDKLMEKVAAGRIELALNATL-DEVLGDNT--------------------
AZOSB01359      --FRAE-KILIDKLMEKVEAGKIELVLNATL-DEVLGDNT--------------------
BORA102682      --FRAE-PILVDKLMDKVQNGNMELRVFSTL-EEVLGDDS--------------------
BORPA03415      --FRAE-PILVDKLMDKVANGNMELKLFHTL-DEVLGDDS--------------------
BORBM03577      --FRAE-PILVDKLMDKVANGNMELKLFHTL-DEVLGDDS--------------------
BORPE02280      --FRAE-PILVDKLMDKVANGNMELKLFHTL-DEVLGDDS--------------------
BORPC02060      --FRAE-PILVDKLMDKVANGNMELKLFHTL-DEVLGDDS--------------------
BORP102392      --FRAE-PILVDKLMDKVANGNMELKLFHTL-DEVLGDDS--------------------
BORBR03870      --FRAE-PILVDKLMDKVANGNMELKLFHTL-DEVLGDDS--------------------
BORPD01524      --FRAE-PILVDKLMDKVENGNMELKLFHTL-DEVLGDDS--------------------
ACHXA01179      --FRAE-PILVDKLMSKVENGNMELKLFHTL-EEVLGDDS--------------------
RHOFT03114      --FRAE-PILIDKLMEKVGSGKIVLKTSNTL-DEVLGDAS--------------------
VEREI01701      --FKAE-PILVDQIGQKVAAGKIELRLLHTL-DEVLGDHS--------------------
VARPE01375      --FRAE-PILIDKVKEKVAEGKIVLKLHNEL-DEVLGDGT--------------------
VARPS01266      --FRAE-PILIDKLMEKVAEGKIVLKLHNEL-DQVLGDDT--------------------
DELAS05272      --FKAE-PILVDKLMDKVRAGKIELKTHFVL-EEVLGDQS--------------------
DELSC01235      --FKAE-PILVDKLMDKVRAGKIELKTHFVL-EEVLGDQS--------------------
COMT200883      --FKAE-PILVDKLMEKVEEGKIELKTHFTL-DEVLGDQS--------------------
ACIAC03267      --FKAE-PILVDKLNEKVAAGKIELKVFHTL-DEVLGNDS--------------------
ACIAP03249      --FKAE-PILVDKLNEKVAAGKIELKVFHTL-DEVLGNDG--------------------
ACIET02648      --FRAE-PILIDKLMEKVAAGKIELKTFFTL-DEVLGDAT--------------------
ALIDK03528      --FRAE-PILVDKLMERVAAGKIELKTFFTL-DEVLGDAS--------------------
RUBGI03408      --FRAE-PIMVDKLMDKVKAGKIVLHLWATL-DEVLGDAS--------------------
LEPCP00749      --FRAE-AILIDKLNEKVAAGKMELHLFQTL-DEVLGDST--------------------
POLSJ03719      --FKAE-AILIDKLHEKVAAGKIELRLHHTL-DQVLGDAS--------------------
POLNA03144      --FKAE-AILIDKLHEKVASGKIELKLHHTL-DEVLGDAS--------------------
```

```
METPP01110      --FKAE-AILVDKLMEKVAAGKIELKLFHVL-DEVLGDSS--------------------
RAMTT03218      --FRAE-PILVDKVMQKVAEGKIELKLFKAL-DEVLGDET--------------------
RALPJ02457      --FRAE-PILIDRLHEQEKKGKIEIKTNMVL-DEILGDES--------------------
RALP102102      --FRAE-PILIDRLHEQEKKGKIEIKTNMVL-DEILGDDS--------------------
RALSO02303      --FRAE-PILVDRLLEQQKKGKIEIKYNTVL-DEVLGDDS--------------------
RALS801097      --FRAE-PILVDRLLEQQKKGKIEIKYNTVL-DEVLGDDS--------------------
HERSS01878      --FRAE-PILIDRLLHKVSEGKIAIQWHHTL-DEVVGDDS--------------------
HERAR00955      --FRAE-AILIDRLMAKVAEGKIEIKYNHTL-DEITGDDS--------------------
JANMA01152      --FRAE-AILIDRLMAKVAEGKIEVKWNSTL-EEVTGDDS--------------------
THIK102324      --FRAE-PILVDKLMARAAEGKVELKLWSEL-KEVLGDAS--------------------
POLSQ00682      --FRAE-PILNDRLMAKVAEGKVELKLNATL-DEVLGDEK--------------------
POLNS00989      --FRAE-PILNDRLMAKVAEGKVELKLNATL-DEVLGDEK--------------------
BURP800627      --FRAE-PILIDRLLEKEKEGVVEIKWDHVL-DEVQGNDG--------------------
BURPP00988      --FRAE-PILIDRLLAKEKEGLVEIKWDSTL-DEVTGDQS--------------------
BURSC00728      --FRAE-PILIDRLLAKEKEGVVEIKWNHVL-EEVTGDDS--------------------
BURXL00982      --FRAE-PILIDRLLAKEKEGIVEIKWNHTL-DEVTGDQS--------------------
BURSG00869      --FRAE-PILIDRLLAKEKEGVVEIKWNHTL-DEVTGDQS--------------------
BURRH00629      --FRAE-PILIDRLLAKEQEGIVQIKWNHVL-DEVLGDDS--------------------
BURGB00781      --FRAE-PILIDRLLEKEKEGKVEIKWNHVL-DEVTGDDS--------------------
BURGS00853      --FRAE-PILIDRLLEKEKEGKVEIKWNHVL-DEVTGDDS--------------------
BURPS02618      --FRAE-PILVDRLLEKEKEGAVEIKWDHVL-DEVTGDDS--------------------
BURMA01741      --FRAE-PILVDRLLEKEKEGAVEIKWDHVL-DEVTGDDS--------------------
BURP103016      --FRAE-PILVDRLLEKEKEGAVEIKWDHVL-DEVTGDDS--------------------
BURP002984      --FRAE-PILVDRLLEKEKEGAVEIKWDHVL-DEVTGDDS--------------------
BURM701866      --FRAE-PILVDRLLEKEKEGAVEIKWDHVL-DEVTGDDS--------------------
BURP602945      --FRAE-PILVDRLLEKEKEGAVEIKWDHVL-DEVTGDDS--------------------
BURM902465      --FRAE-PILVDRLLEKEKEGAVEIKWDHVL-DEVTGDDS--------------------
BURMS00740      --FRAE-PILVDRLLEKEKEGAVEIKWDHVL-DEVTGDDS--------------------
BURTA01515      --FRAE-PILVDRLLEKEKEGTVEIKWDHVL-DEVTGDDA--------------------
BURM102386      --FRAE-PILVDRLLEKQKEGVVDIKWDHVL-DEVTGEDS--------------------
BURL300890      --FRAE-PILIDRLLEKEKEGAVVIKWDHVL-DEVTGEDS--------------------
BURVG00860      --FRAE-PILIDRLLEKEKEGVVDIKWDHVL-DEVTGEES--------------------
BURCM00829      --FRAE-PILIDRLLEKEKEGVVDIKWDHVL-DEVTGEES--------------------
BURA400832      --FRAE-PILIDRLLEKEKEGVVDIKWDHVL-DEVTGEES--------------------
BURCA00489      --FRAE-PILIDRLLEKQKEGVVDIKWDHVL-DEVTGEES--------------------
BURCH00966      --FRAE-PILIDRLLEKQKEGVVDIKWDHVL-DEVTGEES--------------------
BURCC00927      --FRAE-PILIDRLLEKQKEGVVDIKWDHVL-DEVTGEES--------------------
BURCJ02922      --FRAE-PILIDRLLEKQKEGVVDIKWDHVL-DEVTGEES--------------------
EDWI902348      --FRAE-KILIDRLMDKVRSGNIVLHTNRTL-DEVLGDDM--------------------
EDWTF01971      --FRAE-KILIDRLMDKVRSGNIVLHTNRTL-DEVLGDDM--------------------
EDWTE02176      --FRAE-KILIDRLMDKVRSGNIVLHTNRTL-DEVLGDDM--------------------
SODGM01094      --FRSE-KILIDRLMDKVNNGNIVLHTNRTL-EEVLGDEM--------------------
MOREP00394      --FRSE-KILIERLMDKVNNGNIFLHTNCTL-EEVLGDDI--------------------
RAHSY01432      --FRAE-KILVDRLNDKVASGNIVLHTHKTL-NEVVGDQM--------------------
RAHAC01419      --FRAE-KILVDRLNDKVASGNIVLHTHKTL-NEVVGDQM--------------------
ERWBE01489      --FRAE-KILIDRLMEKVRNGNIVLHTNQTL-DEVIGDQM--------------------
PANAM01339      --FRAE-KILIDRLMEKVSNGNIVLHTHRTL-EEVVGDQM--------------------
PANAA00662      --FRAE-KILIDRLMEKVSNGNIVLHTHRTL-EEVVGDQM--------------------
PANSA01303      --FRAE-KILIDRLMEKVRNGNIVLHTDRTL-DEVVGDQM--------------------
ERWT902154      --FRAE-KILIDRLNEKVKNGNIVLHTDHTL-DEVLGDQM--------------------
ERWAC01330      --FRAE-KILIDRLNEKVKNGNIVLHTDHTL-DEVLGDQM--------------------
ERWAE01326      --FRAE-KILIDRLNEKVKNGNIVLHTDHTL-DEVLGDQM--------------------
ERWPE02241      --FRAE-KILIDRLNEKVKNGNIILHTDYTL-YEVLGDQM--------------------
ERWP602414      --FRAE-KILIDRLNEKVKNGNIILHTDYTL-YEVLGDQM--------------------
ERWSE02396      --FRAE-KILIDRLNEKVKNGNIILHTDYTL-DEVLGDQM--------------------
PECCP01694      --FRSE-KILIDRLMDKVKNGNIILHTNRTL-DEVLGDDM--------------------
PECWW01889      --FRSE-KILIDRLMDKVKNGNIILHTNRTL-DEVLGDNM--------------------
PECSS01870      --FRSE-KILIDRLMDKVKNGNIILHTNRTL-DEVLGDNM--------------------
PECAS02624      --FRSE-KILIDRLMEKVKNGNIILHTNRTL-DDVLGDDM--------------------
DICDC02220      --FRAE-KILIDRLMAKVDSGNIVLHTYRTL-DEVIGDDM--------------------
DICZE02284      --FRAE-KILIDRLMAKVSSGNIVLHTNRTL-DEVVGDEM--------------------
DICD302017      --FRAE-KILIDRLMAKVSSGNIVLHTNRTL-DEVLGDDM--------------------
DICD502250      --FRAE-KILIDRLMAKVSSGNIVLHTNRTL-DEVLGDDM--------------------
XENBS00829      --FRSE-KILIDRLMEKVKNGNIILHTDRTL-DEVLGDDM--------------------
XENNA01480      --FRSE-KILIDRLMDKVKNGNIILHTDRTL-DEVLGDDM--------------------
```

```
PHOLL01537      --FRSE-KILISRLMDKVKNGNIILHTDRIL-DEVLGDDM--------------------
PHOAA02804      --FRSE-KILISRLMDKVKNGNIILHTDRTL-DEVLGDDM--------------------
SERP501669      --FRSE-KILINRLMEKVKNGNIVLHTDHTL-DEVLGDQM--------------------
SERSA01625      --FRSE-KILIDRLMEKVKNGNIVLHTDHTL-DEVLGDQM--------------------
YERPE01271      --FRSE-KILIDRLMEKVKNGNIVLHTDRTL-DEVLGDDM--------------------
YERPS01390      --FRSE-KILIDRLMEKVKNGNIVLHTDRTL-DEVLGDDM--------------------
YERPA00627      --FRSE-KILIDRLMEKVKNGNIVLHTDRTL-DEVLGDDM--------------------
YERPN02489      --FRSE-KILIDRLMEKVKNGNIVLHTDRTL-DEVLGDDM--------------------
YERPP02190      --FRSE-KILIDRLMEKVKNGNIVLHTDRTL-DEVLGDDM--------------------
YERP302544      --FRSE-KILIDRLMEKVKNGNIVLHTDRTL-DEVLGDDM--------------------
YERPB01460      --FRSE-KILIDRLMEKVKNGNIVLHTDRTL-DEVLGDDM--------------------
YERPY02633      --FRSE-KILIDRLMEKVKNGNIVLHTDRTL-DEVLGDDM--------------------
YERPG01392      --FRSE-KILIDRLMEKVKNGNIVLHTDRTL-DEVLGDDM--------------------
YERPD01171      --FRSE-KILIDRLMEKVKNGNIVLHTDRTL-DEVLGDDM--------------------
YERP100890      --FRSE-KILIDRLMEKVKNGNIVLHTDRTL-DEVLGDDM--------------------
YERPZ01206      --FRSE-KILIDRLMEKVKNGNIVLHTDRTL-DEVLGDDM--------------------
YERPH02469      --FRSE-KILIDRLMEKVKNGNIVLHTDRTL-DEVLGDDM--------------------
YERE801438      --FRSE-KILIDRLMEKVKNGNIVLHTDRTL-DEVLGDDM--------------------
YERE302565      --FRSE-KILIDRLMEKVKNGNIVLHTDRTL-DEVLGDDM--------------------
YERE100392      --FRSE-KILIDRLMEKVKNGNIVLHTDRTL-DEVLGDDM--------------------
PROMH00687      --FRSE-KILIDRLMDKVKNGNIILHTDRTL-DEVLGDDM--------------------
PROSM03196      --FRAE-KILVDRLMDKVKNGNITLHTDRTL-DEVLGDDM--------------------
TOLAT02279      --FRSE-KILIKRLYDKVEQGIIKLHLDRTL-EEVLGDEM--------------------
AERVB02262      --FRAE-KILIKRLHDKIESGNIVLHTHQTL-DEVLGDQM--------------------
AERHH01811      --FRAE-KILIKRLHDKVASGNIVLHTHQTL-DEVLGDDM--------------------
AERS402185      --FRAE-KILIKLLHDKVASGNIVLHTHQTL-DEVLGDQM--------------------
PSYIN02070      --FRSE-KILLDRLNAKIANGNIILHTDRTL-DEVLGDEM--------------------
SHELP02018      --FRSE-KILTKRLMDKVANGNIVLHLDSTL-DEVVGDNM--------------------
SHEVD02297      --FRSE-KILTKRLMDKVENGNIILHLDNTL-DEVVGDQM--------------------
SHEPW02341      --FRSE-KILTKRLMDKVENGNIILHLDNTL-EEVVGDAM--------------------
SHEPA02202      --FRSE-KILTKRLMDKVANGNIILHLDNTL-DEVVGDQM--------------------
SHEHH02007      --FRSE-KILTKRLMDKVESGNIILHLDNTL-DEVVGDQM--------------------
SHESH02117      --FRSE-KILTKRLMDKVENGNIILHLDNTL-DEVVGDQM--------------------
SHEWM02454      --FRSE-KILTKRLMDKVANGNITLHLDNTL-DEVVGDQM--------------------
SHEAM01758      --FRSE-KILIDRLMDKVANGNIILHLDKTL-DEVVGDNM--------------------
SHEON02159      --FRSE-KILIDRLMDKVANGNIILHLNQTM-DEVVGDAM--------------------
SHESM01945      --FRSE-KILIDRLMDKVANGNIILHLDQTM-EEVVGDAM--------------------
SHESR01991      --FRSE-KILIDRLMDKVANGNIILHLDQTM-EEVVGDAM--------------------
SHESA02025      --FRSE-KILIDRLMDKVANGNIILHLDQTM-EEVVGDAM--------------------
SHESW01953      --FRSE-KILIDRLMDKVANGNIILHLNQTM-DEVVGDAM--------------------
SHEPC01976      --FRSE-KILIDRLMDKVANGNIILHLNQTM-DEVVGDAM--------------------
SHEP201929      --FRSE-KILIDRLMDKVANGNIILHLNQTM-DEVVGDAM--------------------
SHEB502016      --FRSE-KILIDRLMDKVANGNIILHLDQTM-DEVVGDAM--------------------
SHEB802168      --FRSE-KILIDRLMDKVANGNIILHLDQTM-DEVVGDAM--------------------
SHEB202117      --FRSE-KILIDRLMDKVANGNIILHLDQTM-DEVVGDAM--------------------
SHEB902270      --FRSE-KILIDRLMDKVANGNIILHLDQTM-DEVVGDAM--------------------
SHEB602258      --FRSE-KILIDRLMDKVANGNIILHLDQTM-DEVVGDAM--------------------
SHEDO01680      --FRSE-KILIDRLMDKVANGNIILHLDSTL-EEVTGDAM--------------------
SHEFN01895      --FRSE-KILINRLMDKVANGNIILHLDQTL-EEVTGNDM--------------------
PSEHT01677      --FRSE-KILADRLAEKAANGNVVMHYNRTL-DEVLGDQM--------------------
ALISL01611      --FRSE-KILIDRLMDKVENGNIILHTDRTL-DEVLGDEM--------------------
VIBF100893      --FRSE-KILIDRLMDKVENGNIILHTDRTL-EEVLGDDM--------------------
VIBFM00891      --FRSE-KILIDRLMDKVENGNIILHTDRTL-EEVLGDDM--------------------
OCESG01399      --FRSE-KILIDRLMDKVANGNIVLHTDRTL-DEVLGDDM--------------------
VIBA701872      --FRAE-KILINRLMDKVASGNIVLHTDRTL-EEVLGDET--------------------
VIBVY01451      --FRAE-KILINRLMDKVQNGNIVLHTDRVL-DEVLGDEM--------------------
VIBVU02545      --FRAE-KILINRLMDKVQNGNIVLHTDRVL-DEVLGDEM--------------------
VIBVM01748      --FRAE-KILINRLMDKVQNGNIVLHTDRVL-DEVLGDEM--------------------
VIBCH01157      --FRSE-KILIDRLMDKVANGNIVLHTHRTL-DEVLGDEM--------------------
VIBCM01114      --FRSE-KILIDRLMDKVANGNIVLHTHRTL-DEVLGDEM--------------------
VIBCJ02064      --FRSE-KILIDRLMDKVANGNIVLHTHRTL-DEVLGDEM--------------------
VIBC300725      --FRSE-KILIDRLMDKVANGNIVLHTHRTL-DEVLGDEM--------------------
FERBD01969      --FRAE-KILVKRLMDKVANGNIVLHTDRTL-DEVLGDEM--------------------
VIBFN02085      --FRAE-KILINRLMDKVENGNIVLHTDRTL-DEVLGDEM--------------------
VIBTL01089      --FRAE-KILVKRLMDKVENGNIVLHTDRTL-DEVLGDDM--------------------
```

```
VIBPA01248      --FRAE-KILINRLMDKVENGNIILHTDRTL-DEVLGDDM--------------------
VIBAE02128      --FRAE-KILVKRLMDKVESGNIVLHTDRTL-DEVLGDDM--------------------
VIBCB00599      --FRAE-KILVKRLMDKVESGNIVLHTDRTL-DEVLGDDM--------------------
PANVC00701      --FRAE-KILIDRLMEKVRNGNIVLHTHRTL-EEVTGDQM--------------------
SHIBC02443      --FRAE-KILINRLMDKVANGNIVLHTNRTL-DEVTGDQM--------------------
ENTBF02792      --FRAE-KILIKRLMDKVESGNIVLHTHRTL-EEVTGDQM--------------------
KLEP700892      --FRAE-KILIKRLMDKVASGNIVLHTDRTL-EEVTGDQM--------------------
KLEPH01782      --FRAE-KILIKRLMDKVASGNIVLHTDRTL-EEVTGDQM--------------------
KLEP303551      --FRAE-KILIKRLMDKVASGNIVLHTDRTL-EEVTGDQM--------------------
KLEVT03420      --FRAE-KILIKRLMDKVASGNIVLHTDRTL-EEVTGDQM--------------------
ENTAK02985      --FRAE-KILIKRLMDKVESGNIVLHTDRTL-EEVTGDQM--------------------
KLEOK03151      --FRAE-KILIKRLMDKVESGNIVLHTHRTL-EEVTGDQM--------------------
SALAR01925      --FRAE-KILIKRLMDKVENGNIVLHTNRTL-EEVTGDQM--------------------
SALBC00812      --FRAE-KILIKRLMDKVDNGNIVLHTNRTL-EEVTGDQM--------------------
SALPC00926      --FRAE-KILIKRLMDKVENGNIILHTNRTL-EEVTGDQM--------------------
SALTI00868      --FRAE-KILIKRLMDKVENGNIILHTNRTL-EEVTGDQM--------------------
SALCH00905      --FRAE-KILIKRLMDKVENGNIILHTNRTL-EEVTGDQM--------------------
SALPA01706      --FRAE-KILIKRLMDKVENGNIILHTNRTL-EEVTGDQM--------------------
SALTY00927      --FRAE-KILIKRLMDKVENGNIILHTNRTL-EEVTGDQM--------------------
SALPK01786      --FRAE-KILIKRLMDKVENGNIILHTNRTL-EEVTGDQM--------------------
SALHS00978      --FRAE-KILIKRLMDKVENGNIILHTNRTL-EEVTGDQM--------------------
SALEP00854      --FRAE-KILIKRLMDKVENGNIILHTNRTL-EEVTGDQM--------------------
SALDC00926      --FRAE-KILIKRLMDKVENGNIILHTNRTL-EEVTGDQM--------------------
SALA400892      --FRAE-KILIKRLMDKVENGNIILHTNRTL-EEVTGDQM--------------------
SALG200876      --FRAE-KILIKRLMDKVENGNIILHTNRTL-EEVTGDQM--------------------
SALTS00888      --FRAE-KILIKRLMDKVENGNIILHTNRTL-EEVTGDQM--------------------
SALT400905      --FRAE-KILIKRLMDKVENGNIILHTNRTL-EEVTGDQM--------------------
SALPS01908      --FRAE-KILIKRLMDKVENGNIILHTNRTL-EEVTGDQM--------------------
SALT101040      --FRAE-KILIKRLMDKVENGNIILHTNRTL-EEVTGDQM--------------------
SALTD00960      --FRAE-KILIKRLMDKVENGNIILHTNRTL-EEVTGDQM--------------------
SALPB02472      --FRAE-KILIKRLMDKVENGNIILHTNRTL-EEVTGDQM--------------------
SALNS00928      --FRAE-KILIKRLMDKVENGNIILHTNRTL-EEVTGDQM--------------------
SALSV01000      --FRAE-KILIKRLMDKVENGNIILHTNRTL-EEVTGDQM--------------------
ECOS500812      --FRAE-KILIKRLMDKVENGNIILHTNRTL-EEVTGDQM--------------------
ECOL600981      --FRAE-KILIKRLMDKVENGNIILHTNRTL-EEVTGDQM--------------------
ECOL500883      --FRAE-KILIKRLMDKVENGNIILHTNRTL-EEVTGDQM--------------------
ECOUT00876      --FRAE-KILIKRLMDKVENGNIILHTNRTL-EEVTGDQM--------------------
ECOK100776      --FRAE-KILIKRLMDKVENGNIILHTNRTL-EEVTGDQM--------------------
ECOSM02148      --FRAE-KILIKRLMDKVENGNIILHTNRTL-EEVTGDQM--------------------
ECOLU01057      --FRAE-KILIKRLMDKVENGNIILHTNRTL-EEVTGDQM--------------------
ECO7I02136      --FRAE-KILIKRLMDKVENGNIILHTNRTL-EEVTGDQM--------------------
ECO8100828      --FRAE-KILIKRLMDKVENGNIILHTNRTL-EEVTGDQM--------------------
ECO4500862      --FRAE-KILIKRLMDKVENGNIILHTNRTL-EEVTGDQM--------------------
ECOAB00879      --FRAE-KILIKRLMDKVENGNIILHTNRTL-EEVTGDQM--------------------
ECO4400961      --FRAE-KILIKRLMDKVENGNIILHTNRTL-EEVTGDQM--------------------
ECOUM02550      --FRAE-KILIKRLMDKVENGNIILHTNRTL-EEVTGDQM--------------------
ECOKI00869      --FRAE-KILIKRLMDKVENGNIILHTNRTL-EEVTGDQM--------------------
ECOC100920      --FRAE-KILIKRLMDKVENGNIILHTNRTL-EEVTGDQM--------------------
ECOC200920      --FRAE-KILIKRLMDKVENGNIILHTNRTL-EEVTGDQM--------------------
ECO8N00803      --FRAE-KILIKRLMDKVENGNIILHTNRTL-EEVTGDQM--------------------
ECO2700862      --FRAE-KILIKRLMDKVENGNIILHTNRTL-EEVTGDQM--------------------
ECO2600974      --FRAE-KILIKRLMDKVENGNIILHTNRTL-EEVTGDQM--------------------
ECOH100942      --FRAE-KILIKRLMDKVENGNIILHTNRTL-EEVAGDQM--------------------
SHIB301848      --FRAE-KILIKRLMDKVENGNIILHTNRML-EEVTGDQM--------------------
ECOLI00846      --FRAE-KILIKRLMDKVENGNIILHTNRTL-EEVTGDQM--------------------
ECO5700970      --FRAE-KILIKRLMDKVENGNIILHTNRTL-EEVTGDQM--------------------
SHISS00756      --FRAE-KILIKRLMDKVENGNIILHTNRTL-EEVTGDQM--------------------
SHIBS00679      --FRAE-KILIKRLMDKVENGNIILHTNRTL-EEVTGDQM--------------------
SHIDS01857      --FRAE-KILIKRLMDKVENGNIILHTNRTL-EEVTGDQM--------------------
ECO2400906      --FRAE-KILIKRLMDKVENGNIILHTNRTL-EEVTGDQM--------------------
ECODH00784      --FRAE-KILIKRLMDKVENGNIILHTNRTL-EEVTGDQM--------------------
ECOHS00922      --FRAE-KILIKRLMDKVENGNIILHTNRTL-EEVTGDQM--------------------
ECOLC02627      --FRAE-KILIKRLMDKVENGNIILHTNRTL-EEVTGDQM--------------------
ECO5E00946      --FRAE-KILIKRLMDKVENGNIILHTNRTL-EEVTGDQM--------------------
ECOSE00936      --FRAE-KILIKRLMDKVENGNIILHTNRTL-EEVTGDQM--------------------
```

```
ECO5500894      --FRAE-KILIKRLMDKVENGNIILHTNRTL-EEVTGDQM--------------------
ECO8A00877      --FRAE-KILIKRLMDKVENGNIILHTNRTL-EEVTGDQM--------------------
ECOBB00863      --FRAE-KILIKRLMDKVENGNIILHTNRTL-EEVTGDQM--------------------
ECO5T00943      --FRAE-KILIKRLMDKVENGNIILHTNRTL-EEVTGDQM--------------------
ECOBW00723      --FRAE-KILIKRLMDKVENGNIILHTNRTL-EEVTGDQM--------------------
ECO1000919      --FRAE-KILIKRLMDKVENGNIILHTNRTL-EEVTGDQM--------------------
ECOBD02604      --FRAE-KILIKRLMDKVENGNIILHTNRTL-EEVTGDQM--------------------
ECOD102659      --FRAE-KILIKRLMDKVENGNIILHTNRTL-EEVTGDQM--------------------
ECOBR00856      --FRAE-KILIKRLMDKVENGNIILHTNRTL-EEVTGDQM--------------------
ECOLX02610      --FRAE-KILIKRLMDKVENGNIILHTNRTL-EEVTGDQM--------------------
ECO1A00939      --FRAE-KILIKRLMDKVENGNIILHTNRTL-EEVTGDQM--------------------
ECOCB01054      --FRAE-KILIKRLMDKVENGNIILHTNRTL-EEVTGDQM--------------------
ECOKO02859      --FRAE-KILIKRLMDKVENGNIILHTNRTL-EEVTGDQM--------------------
ECO1E03320      --FRAE-KILIKRLMDKVENGNIILHTNRTL-EEVTGDQM--------------------
ECOLW01217      --FRAE-KILIKRLMDKVENGNIILHTNRTL-EEVTGDQM--------------------
SHIFL01399      --FRAE-KILIKRLMDKVENGNIILHTNRTL-EEVTGDQM--------------------
SHIF800759      --FRAE-KILIKRLMDKVENGNIILHTNRTL-EEVTGDQM--------------------
SHIF200805      --FRAE-KILIKRLMDKVENGNIILHTNRTL-EEVTGDQM--------------------
CITK802118      --FRAE-KILIKRLMDKVENGNIVLHTHRTL-EEVTGDQM--------------------
CITRI00924      --FRAE-KILIKRLMDKVENGNIVLHTHRTL-EEVTGDQM--------------------
CROS802385      --FRAE-KILVKRLMDKVESGNIVLHTHRTL-EEVTGDQM--------------------
CROTZ01500      --FRAE-KILIKRLMDKVESGNIVLHTHRTL-EEVTGDQM--------------------
ENTLS02865      --FRAE-KILIKRLMDKVESGNIVLHTNRTL-EEVTGDQM--------------------
ENT3801400      --FRAE-KILIKRLMDKVASGNIVLHTHRTL-EEVTGDQM--------------------
ENTAL01384      --FRAE-KILIKRLMDKVASGNIVLHTNRTL-EEVTGDQM--------------------
ENTCC02679      --FRAE-KILIKRLMDKVASGNIVLHTNRTL-EEVTGDQM--------------------


STRT101547      --IKVRSVDIK-----------N-----VKT-------G---EVTNHEF------GGVFV
STRT201508      --IKVRSVDIK-----------N-----VKT-------G---EVTNHEF------GGVFV
STRTD01356      --IKVRSVDIK-----------N-----VKT-------G---EVTNHEF------GGVFV
STRTN01533      --IKVRSVDIK-----------N-----VKT-------G---EVTNHEF------GGVFV
STRE500345      --IKVCSVDIK-----------N-----VKT-------G---EVTNHEF------GGVFV
STRE801625      --IKVRSVDIK-----------N-----VKT-------G---EVTNHEF------GGVFV
STREH01636      --IKVRSVDIK-----------N-----VKT-------G---EVTNHEF------GGVFV
STREC01656      --IKVSNVVIE-----------N-----VKT-------G---QVTNHDF------GGVFI
STREM01495      --IKVSNVVIE-----------N-----VKT-------G---QVTSHDF------GGVFI
STRE401629      --IKVSNVVIE-----------N-----VKT-------G---QVTSHDF------GGVFI
STRS700387      --IKVSNVVIE-----------N-----VKT-------G---QVTSHDF------GGVFI
STRDG01631      --IKVSNVLIE-----------N-----VKT-------G---QVTDHAF------GGVFI
STRP301388      --IKVSNVLIE-----------N-----VKT-------G---QVTDHAF------GGVFI
STRPZ01205      --IKVSNVLIE-----------N-----VKT-------G---QVTDHAF------GGVFI
STRPQ00464      --IKVSNVLIE-----------N-----VKT-------G---QVTDHAF------GGVFI
STRPD01453      --IKVSNVLIE-----------N-----VKT-------G---QVTDHAF------GGVFI
STRP601399      --IKVSNVLIE-----------N-----VKT-------G---QVTDHAF------GGVFI
STRP801366      --IKVSNVLIE-----------N-----VKT-------G---QVTDHAF------GGVFI
STRPF01453      --IKVSNVLIE-----------N-----VKT-------G---QVTDHAF------GGVFI
STRPG00427      --IKVSNVLIE-----------N-----VKT-------G---QVTDHAF------GGVFI
STRA300284      --IKVSGVTVE-----------N-----LKT-------G---EISEMTF------GGVFI
STRA500290      --IKVSGVTVE-----------N-----LKT-------G---EISEMTF------GGVFI
STRA100288      --IKVSGVTVE-----------N-----LKT-------G---EISEMTF------GGVFI
STRA200291      --IKVSGVTVE-----------N-----LKT-------G---EISEMTF------GGVFI
STRIC00348      --IKVSGVVVE-----------N-----VKT-------G---EISEHEF------GGIFI
STRPX00375      --IKVSGVTVE-----------N-----VKT-------G---ELSEYEF------GGIFI
STRMD00432      --IKVSGVTVE-----------N-----VKT-------G---ELSEHEF------GGIFI
STRS201734      --LRVQSVVIK-----------N-----VKT-------E---EVSELDF------GGVFI
STRSY01721      --LRVQSVVIK-----------N-----VKT-------E---EVSELDF------GGVFI
STRSX01553      --LRVQSVVIK-----------N-----VKT-------E---EVSELDF------GGVFI
STRSE01478      --LRVQSVVIK-----------N-----VKT-------E---EVSELDF------GGVFI
STREJ01635      --LRVQSVVIK-----------N-----VKT-------E---EVSELDF------GGVFI
STRGZ01544      --LRVQSVVIK-----------N-----VKT-------E---EVSELDF------GGVFI
STRS401593      --LRVQSVVIK-----------N-----VKT-------E---EVSELDF------GGVFI
LACGT00720      --RKIESVRYK-----------N-----VKT-------G---EVTEANF------GGLFI
LACGL00738      --RKIESVRYK-----------N-----VKT-------G---EVTEANF------GGLFI
STRSV01774      --RKVTGVTFK-----------N-----VKT-------G---EVSQAEF------GGIFI
```

```
STRIJ00350       --R-VQSVVVE-----------N-----VKT-------G---EVSEHEF------GGVFI
STROU01248       --R-VESVVFE-----------N-----VKT-------G---QVTEQAF------GGVFI
STRM601286       --R-VESVVFE-----------N-----VKT-------G---QVTEQAF------GGVFI
STRES00962       --R-VESVVFE-----------N-----VKT-------G---QVTEQAF------GGVFI
STRP701387       --R-VESVVFE-----------N-----VKT-------G---QVTEQAF------GGVFI
STRZT00756       --R-VESVVFE-----------N-----VKT-------G---QVTEQVF------GGVFI
STRP001079       --R-VESVVFE-----------N-----VKT-------G---QVTEQVF------GGVFI
STRZO01270       --R-VESVVFE-----------N-----VKT-------G---QVTEQVF------GGVFI
STRZ600815       --R-VESVVFE-----------N-----VKT-------G---QVTEQVF------GGVFI
STRET00826       --R-VESVVFE-----------N-----VKT-------G---QVTEQVF------GGVFI
STRPS01429       --R-VESVVFE-----------N-----VKT-------G---QVTEQAF------GGVFI
STRZN01287       --R-VESVVFE-----------N-----VKT-------G---QVTEQAF------GGVFI
STRR601306       --R-VESVVFE-----------N-----VKT-------G---QVTEQAF------GGVFI
STRP201217       --R-VESVVFE-----------N-----VKT-------G---QVTEQAF------GGVFI
STRZP01342       --R-VESVVFE-----------N-----VKT-------G---QVTEQAF------GGVFI
STRZI01226       --R-VESVVFE-----------N-----VKT-------G---QVTEQAF------GGVFI
STRPN01360       --R-VESVVFE-----------N-----VKT-------G---QVTEQAF------GGVFI
STRP401357       --R-VESVVFE-----------N-----VKT-------G---QVTEQAF------GGVFI
STRZJ01282       --R-VESVVFE-----------N-----VKT-------G---QVTEQAF------GGVFI
STRPJ01336       --R-VESVVFE-----------N-----VKT-------G---QVTEQAF------GGVFI
STRPI01436       --R-VESVVFE-----------N-----VKT-------G---QVTEQAF------GGVFI
MARHT00044       ---QVTGVKVK-----------N-----LKT-------G---EEYVYET------DGVFI
THEP300413       ---GVERLKLK-----------N-----VKT-------G---EESTLNV------DGVFV
THEPX00884       ---GVERLKLK-----------N-----VKT-------G---EESTLNV------DGVFV
THESX01857       ---GVERLKLK-----------N-----VKT-------G---EESTLNV------DGVFV
THEM301788       ---GVERLKLK-----------N-----VKT-------G---EESTLNV------DGVFV
THEIA01812       ---GVERLKLK-----------N-----VKT-------G---EESTLNV------DGVFV
THETC00443       ---GVEGLVLK-----------N-----VKT-------N---EESTLKV------DGVFI
THESW01026       ---GVEGLVLK-----------N-----VKT-------N---EETSLKV------DGVFI
THEXL00360       ---GVEGLVLK-----------N-----VKT-------N---EETTLKV------DGVFI
THEID01127       ---QVKAIKLK-----------N-----CKT-------G---EESSLEV------DGVFI
THEOJ00147       ---AVEEVVVR-----------N-----VKT-------G---EKTSVPV------DGIFV
DESAS01237       ---AVDAVKLK-----------D-----VRT-------G---EFSDLEV------GGVFV
DESK701373       ---TVTGVRIK-----------D-----VRT-------G---QTSELPV------DGVFI
KYRT200729       --RKVERLVLE-----------N-----AVT-------G---EQETVEA------DGIFI
STACT00410       --GKVGSVTLQ-----------S-----TID-------G---KEETLPA------DGVFV
STAS101952       --GKVGSVTLE-----------S-----TKD-------G---SEQTLDA------DGVFI
STALH01969       --GKVGSVTLV-----------S-----TKD-------G---SEQTLDA------DGVFI
STAEQ00422       --GKVGSVTLE-----------S-----TKD-------G---AEQTYDA------DGVFI
STAES00543       --GKVGSVTLE-----------S-----TKD-------G---AEQTYDA------DGVFI
STAAB00714       --GKVGSVTLT-----------S-----TKD-------G---SEETHEV------DGVFI
STAA500765       --GKVGSVTLT-----------S-----TKD-------G---SEETHEA------DGVFI
STAAM00757       --GKVGSVTLT-----------S-----TKD-------G---SEETHEA------DGVFI
STAAW00726       --GKVGSVTLT-----------S-----TKD-------G---SEETHEA------DGVFI
STAAS00733       --GKVGSVTLT-----------S-----TKD-------G---SEETHEA------DGVFI
STAAN00727       --GKVGSVTLT-----------S-----TKD-------G---SEETHEA------DGVFI
STAAC00807       --GKVGSVTLT-----------S-----TKD-------G---SEETHEA------DGVFI
STAA300727       --GKVGSVTLT-----------S-----TKD-------G---SEETHEA------DGVFI
STAA800734       --GKVGSVTLT-----------S-----TKD-------G---SEETHEA------DGVFI
STAA100752       --GKVGSVTLT-----------S-----TKD-------G---SEETHEA------DGVFI
STAA200774       --GKVGSVTLT-----------S-----TKD-------G---SEETHEA------DGVFI
STAA900759       --GKVGSVTLT-----------S-----TKD-------G---SEETHEA------DGVFI
STAAE00716       --GKVGSVTLT-----------S-----TKD-------G---SEETHEA------DGVFI
STAAT00767       --GKVGSVTLT-----------S-----TKD-------G---SEETHEA------DGVFI
STAAD00695       --GKVGSVTLT-----------S-----TKD-------G---SEETHEA------DGVFI
STAA000815       --GKVGSVTLT-----------S-----TKD-------G---SEETHEA------DGVFI
STAAH02407       --GKVGSVTLT-----------S-----TKD-------G---SEETHEA------DGVFI
STAAF00763       --GKVGSVTLT-----------S-----TKD-------G---SEETHEA------DGVFI
STAAK00744       --GKVGSVTLT-----------S-----TKD-------G---SEETHEA------DGVFI
STAAJ00703       --GKVGSVTLT-----------S-----TKD-------G---SEETHEA------DGVFI
STAAG00690       --GKVGSVTLT-----------S-----TKD-------G---SEETHEA------DGVFI
STAA400732       --GKVGSVTLT-----------S-----TKD-------G---SEETHEA------DGVFI
STAAR00788       --GKVGSVTLT-----------S-----TKD-------G---SEETHEA------DGVFI
LISSS02378       --KKVTGAKLV-----------S-----TVD-------G---SESIMPV------DGVFI
LISIN02590       --KKVTSVKLV-----------S-----TVD-------G---SESIMPV------DGVFI
```

```
LISW602421      --KKVTGAKLV-----------S-----TVD-------G---SESIMPV------DGVFI
EXISA00870      --GKVGGARLV-----------S-----TID-------G---TETDHEI------DGVFI
EXIS202358      --GKVGGIELI-----------N-----TKT-------A---ETTTYPI------DGVFI
EXIAB02180      --GKVGGIELI-----------N-----TKT-------A---ATTTYPI------DGVFI
OCEIH02462      --GKVSSVTLK-----------N-----TKT-------N---EVNNFDA------EGVFI
BACIE01058      --GKVSSVTLE-----------S-----TKD-------G---STKDFKT------DGVFI
BACCJ03468      --NKVSSVTIV-----------N-----KED-------G---SEREFKT------DGVFI
BACHD03507      --GKVSSVTIE-----------H-----AKT-------G---EQQDFKT------DGVFI
BACPE03382      --GKVGSVTIE-----------N-----TET-------G---ETKEFKT------DGVFI
SOLSS00743      --GKVGKVTLV-----------S-----TVD-------G---TETEEAA------DGVFV
BACC600715      --GKVGSVTLV-----------S-----TVD-------G---SEKPFKT------DGVFI
ANOFW02486      --GRVGSVTLV-----------H-----TQT-------G---EEREFPC------DGVFI
GEOKA03042      --GKVGSVTLV-----------H-----TQT-------G---EEREFPC------DGVFI
GEOSY02976      --GKVGSVTLV-----------H-----TQT-------G---EEREFPC------DGVFI
GEOTN02952      --GKVGSVTLV-----------H-----TQT-------G---EEREFPC------DGVFI
GEOSW02588      --GKVNSVTLV-----------H-----TQT-------G---EEREFPC------DGVFI
GEOS000381      --GKVNSVTLV-----------H-----TQT-------G---EEREFPC------DGVFI
GEOTC00378      --GKVNSVTLV-----------H-----TQT-------G---EEREFPC------DGVFI
BACMD04906      --GKVGSVTLV-----------D-----TKT-------G---EEREFGA------DGVFI
BACMQ04908      --GKVGSVTLV-----------D-----TKT-------G---EEREFGA------DGVFI
BACWK04830      --GKVGSVTLV-----------D-----VNS-------G---EEQEVKT------DGVFI
BACAN04889      --GKVGSVTLV-----------D-----VNS-------G---EEKEVKT------DGVFV
BACC105210      --GKVGSVTLV-----------D-----VNS-------G---EEKEVKT------DGVFV
BACC705051      --GKVGSVTLV-----------D-----VNS-------G---EEKEVKT------DGVFV
BACC005112      --GKVGSVTLV-----------D-----VNS-------G---EEKEVKT------DGVFV
BACC305043      --GKVGSVTLV-----------D-----VNS-------G---EEKEVKT------DGVFV
BACAC05232      --GKVGSVTLV-----------D-----VNS-------G---EEKEVKT------DGVFV
BACAA04720      --GKVGSVTLV-----------D-----VNS-------G---EEKEVKT------DGVFV
BACT005085      --GKVGSVTLV-----------D-----VNS-------G---EEKEVKT------DGVFV
BACC205124      --GKVGSVTLV-----------D-----VNS-------G---EEKEVKT------DGVFV
BACC405064      --GKVGSVTLV-----------D-----VNS-------G---EEKEVKT------DGVFV
BACT104712      --GKVGSVTLV-----------D-----VNS-------G---EEKEVKT------DGVFV
BACLD03769      --GKVGKVTLV-----------D-----TVT-------G---EEEEFRT------DGVFI
BACPZ03376      --GKVGNVTLV-----------D-----TVT-------G---EESEFKT------DGVFI
BACSU03600      --GKVGNVTLV-----------D-----TVT-------G---EESEFKT------DGVFI
BACST01653      --GKVGNVTLV-----------D-----TVT-------G---EESEFKT------DGVFI
BACPT03541      --GKVGNVTLV-----------D-----TVT-------G---EESEFKT------DGVFI
LEUGG00661      --DKVTGVRVI-----------D-----NQT-------H---EVSQINT------SGVFV
LEUGJ00629      --DKVTGVRVI-----------D-----NQT-------H---EVSQINT------SGVFV
LEUCJ00485      --EKVTGVTVI-----------N-----NQT-------Q---EKSHIDA------SGIFI
LACAR00657      --TRVTGIKCR-----------D-----KET-------G---VEKEIKA------AGVFI
LACA300647      --TRVTGIKCR-----------D-----KET-------G---VEKEIKA------AGVFI
LACAL00643      --TRVTGIKCR-----------D-----KET-------G---VEKEIKA------AGVFI
LACKZ00964      --NRVTSIKYR-----------D-----KET-------G---EEKELPT------AGVFI
LACRJ00357      --IKVTGVKTH-----------N-----NKT-------G---EDGEMAA------DGVFI
LACRD00361      --IKVTGVKTH-----------N-----NKT-------G---EDGEMAA------DGVFI
LACRS01501      --IKVTGVKTH-----------N-----NKT-------G---EDGEMAA------DGVFI
LACSM00455      --RKVTNVKIK-----------N-----NQT-------G---EETVVPA------AGVFV
LACRG00878      --MKVTGVKYR-----------D-----KET-------G---EEHVLPV------AGVFI
LACRL00939      --MKVTGVKYR-----------D-----KET-------G---EEHVLPV------AGVFI
LACC300844      --MKVTGVKYR-----------D-----KET-------G---EEHVLPV------AGVFI
LACCZ00824      --MKVTGVKYR-----------D-----KET-------G---EEHVLPV------AGVFI
LACCB00988      --MKVTGVKYR-----------D---RDKET-------G---EEHILPV------AGVFI
LACCD01016      --MKVTGVKYR-----------D---RDKET-------G---EEHILPV------AGVFI
LACCC01014      --MKVTGVKYR-----------D---RDKET-------G---EEHILPV------AGVFI
LACBN01277      --NKVTGVKVV-----------N-----NQT-------N---EESVLET------SGVFI
LACBA00605      --MKVTGVATK-----------N-----NQT-------G---ETGEIAA------SGVFI
LACPL00650      --KKVTGVKVN-----------N-----NKT-------G---EDSEIAV------DGVFI
LACPJ00628      --KKVTGVKVN-----------N-----NKT-------G---EDSEIAV------DGVFI
LACPS00585      --KKVTGVKVN-----------N-----NKT-------G---EDSEIAV------DGVFI
PEDCP00497      ---KVEGVKVN-----------N-----NKT-------G---EDSFIDA------SGVFI
CARS100359      --NKVTGVKVR-----------N-----VHT-------S---EVTEFPA------DGAFI
AERUA00263      --KQVTSILVE-----------N-----VKS-------H---EVTEVPA------GGVFI
ELUMP00590      --LGLNALKVK-----------N-----LND-------Q---TVRELPC------QGVFV
SPHPG02940      DGNRVTGVILK-----------N-----LVT-------G---EEYHRDF------DAVFI
```

```
SPHGB01868      DEKKVTSVILK-----------D-----LVK-------G---TEYEREF------DAVFI
TREPZ00273      -DKQVNAVILE-----------NTADHT---------------QYEEPV------NAVFI
TREAZ03414      -DKKTASVILE-----------KTTAECKPS-------G---EINEEAA------DAVFI
SPITD00734      GVKKVSSVLLK-----------R-----VDT-------G---ETYEEPI------DAVFI
SPITZ01373      GVKKVSSVLLK-----------R-----VDT-------G---ETYEEPI------DAVFI
TREPA00803      GETKVSSVLLK-----------D-----VKT-------G---ETRELAC------DAVFF
TREPS00802      GETKVSSVLLK-----------D-----VKT-------G---ETRELAC------DAVFF
TREPC00747      GETKVSSVLLK-----------D-----VKT-------G---ETRELAC------DAVFF
TREPM00823      GETKVSSVLLK-----------D-----VKT-------G---ETRELAC------DAVFF
TREPD00824      GETKVSSVLLK-----------D-----VKT-------G---ETRELAC------DAVFF
TREPU00781      GETKVSSVLLK-----------D-----VKT-------G---ETRELAC------DAVFF
ENCCU00216      ---KVERITIR-----------N-----VET-------G---EMKTIPM------DGVFF
HELM100964      --MGVVGVRIK-----------N-----TQT-------N---EERKLDV------PGIFI
HELCP01490      --SGVSGVRIR-----------H-----TDS-------N---ETEVISV------YGLFI
ARCFU01526      ---KVEKVVAY-----------N-----REK-------K---EEFEIEA------DGIFV
FERPA02445      ---KVERLVLL-----------N-----RKT-------N---EESVLEV------DGVFI
ARCVS01910      ---RVERVVLL-----------D-----RVK-------N---EKFVVEA------DGVFI
METEZ00677      N-NFVESALLR-----------D-----LNT-------N---ELSEISI------DGVFI
METHD00869      K-TGVENIILR-----------N-----LKT-------G---EMNTLPV------EGVFV
METMA02304      I-KKVEKIILE-----------D-----VNS-------R---ETRELST------NGVFI
METAC01311      V-KKVEKVILQ-----------D-----LNS-------K---ESRELST------NGVFI
KOSOT00298      S-DKVERLILK-----------N-----VET-------G---ELSTLEV------EGVFV
MARPK01613      D-KKVTQLVLE-----------N-----RET-------G---EITNFDV------DGVFV
SLAHD02439      --GVLATMKVR-----------N-----VKT-------D---EVTVIEADEDDGMFGLFG
FILAD00976      --GLVESIVVE-----------D-----VRD-------G---SREEIHASQEDGTMGIFA
BUTPB02463      ---KVTHLVVE-----------N-----VKN-------E---ETNDVNV------YGIFV
CLOPH00250      ---AVNAVKVR-----------N-----VAT-------E---ETSELNV------DGVFI
CLOSW00592      ---QVESLTIK-----------N-----SKT-------E---ETQKLAV------DGVFI
LACFC00207      --NKVTGVKTH-----------N-----NQT-------N---EDGYIDA------DGVFV
CRYCD00853      --GAVVGVVIR-----------D-----VNT-------G---ETQTIPT------RAVFV
EGGLE01847      --GKLAGVRLE-----------M-----LET-------G---EERDIAV------DGLFV
PYRFU01410      ---KVESVVLK-----------N-----VKT-------G---ETFEKKV------DGVFI
PYRHO01476      ---KVESVVLK-----------N-----VKT-------G---ETVEKKV------DGVFI
PYRAB00730      ---KVESVVLK-----------N-----VKT-------G---ETFEKKV------DGVFI
PYRSN00014      ---KVESVVLK-----------N-----VKT-------G---ETFEKKV------DGVFI
THEGJ00181      ---KVEAVRLK-----------N-----VKT-------G---EEKEMKV------DGVFI
THEKO02097      ---KVEAVRLK-----------N-----VKT-------G---EETVMEV------DGVFI
THEON01610      ---KVEAVKLK-----------N-----VKT-------G---EETEMAV------DGVFI
THES401476      ---KVEAVKLK-----------N-----RVT-------G---EETEMAV------DGVFI
SYNWW02368      ---LMKSLLLK-----------N-----LKT-------G---EEERLEV------EGLFV
UNCTG00012      ---SVEKVTIT-----------N-----VKT-------N---ESKGLII------DGVFV
THEA101460      ---FVESLTLR-----------D-----TVT-------G---QESELPV------DGVFI
DENA201647      ---KVECLTVK-----------N-----LKT-------G---NVTELAV------DGLFI
DEFDS00488      ---KIESITLY-----------D-----KKL-------G---KTHDLKV------DGVFV
CALNY01292      ---KVESLTIQ-----------D-----KLT-------G---KEFDLLI------DGIFV
SYNGF01652      ---KVEKIVVR-----------N-----LKN-------E---ELQEINV------DGVFI
DESB201123      ---GVQSVTMR-----------N-----LKT-------G---EQFDLPC------DGAFV
DEIPM00845      ---QVSAVRLK-----------N-----LKT-------G---EESVMET------DGVFI
DEIRA01924      ---SVSGVKLR-----------N-----LKT-------G---EVSELAT------DGVFI
DEIML01437      FTQQVRAVRLR-----------N-----LKT-------G---EVTDFQT------DGVFI
DEIGD02532      ---QVMGVRLK-----------N-----LKT-------G---ETSEFPT------DGVFI
DEIDV00622      ---HVTGVRLT-----------N-----LKT-------G---ETSEMST------DGVFI
TRURR01079      ---QVEGVRLH-----------N-----LKT-------G---ERSVLAA------DGVFV
THETG01863      ---QVTGVRLK-----------N-----LKT-------G---EEYVYPT------DGVFV
THET201543      ---QVTGVRLK-----------N-----LKT-------G---EEYVYPT------DGVFV
THET801911      ---QVTGVRLK-----------N-----LKT-------G---EEYVYPT------DGVFV
OCEP502115      ---HVTGVRVK-----------N-----LKT-------N---EEYVYPT------DGVFV
DEHLB00585      ---SVERLKLI-----------N-----TKT-------G---DESILEV------GGIFV
DEHMG00439      ---SIQSLSLN-----------N-----LKT-------L---KTSTLPI------DGLFV
DEHMB00497      ---SIQSLSLN-----------N-----LKT-------L---KTSTLPI------DGLFV
DEHMC00427      ---SIQSLSLN-----------N-----LKT-------L---KTSTLPI------DGLFV
THELD00726      ---MVTHLVLH-----------N-----VKT-------Q---EDSKLPV------AGVFV
ANAMD00624      ---MVEKVVIK-----------N-----VKT-------G---EISDLPV------AGVFV
THEAS00815      ---MVERLVLW-----------N-----VKT-------Q---EVSTLPV------SGVFI
AMICL00707      ---MVENLVLK-----------N-----VKT-------G---EVSDLPV------AGVFV
```

```
CLOCE01684      ---GVEGLKIK-----------N-----IKT-------G---EKSSIDV------DGLFV
HYDS000616      ---FVNSLLLE-----------D-----TKT-------G---EHSILSV------DGVFI
HYDTT00224      ---FVEKLILK-----------N-----TKS-------G---EVSELEV------EGVFI
PELTS01405      ---LVESVTVK-----------N-----VKN-------G---EERDLQV------DGVFI
DESRL02179      ---KVEVIKVK-----------D-----VQT-------G---DQTRVLV------DGIFV
BORBP00506      ---SVSSIKIF-----------N-----EKD-------N---VVNKLKV------NGVFM
BORAP00518      ---SISSVKIF-----------N-----KKD-------N---IVYKLEV------NAVFM
BORBU00514      ---SVSSVKIF-----------N-----KKD-------N---VVYELEV------SAVFM
BORBZ00490      ---SVSSVKIF-----------N-----KKD-------N---VVYELEV------SAVFM
BORBN00493      ---SVSSVKIF-----------N-----KKD-------N---VVYELEV------SAVFM
BORRA00486      ---IVSKIHIM-----------D-----NKH-------N---SAFELNV------DGIFI
BORDL00498      ---IVSKIHIM-----------D-----NKH-------N---SAFELNV------DGIFI
BORHD00497      ---VVSGMQIM-----------N-----SKD-------N---STFKLSV------DGIFI
BORT900497      ---IVSMVQII-----------D-----NKN-------N---STFELSV------NGIFV
METKA01560      ---RVEGVELS-------------------------------DGDILPC------EGVFI
MYCA500361      ---ELEAIKVN------------------IN-------G---EVSEMKI------KGLFP
METVS00145      ---KAEGVIVL------------------ID-------G---KEHLIPT------EGIFV
METOI01431      ---KAEGVLVS------------------LN-------G---EEKEIKA------DGIFI
MYCHN00051      ---KIIGLKVR-----------N-----VET-------E---EESLLEG------DCVFF
MYCSL00465      ---KVCGLLVT-----------H-----SET-------G---EEKVISG------KAVFI
MYCS300420      ---KVCGLLVT-----------H-----SET-------G---EEKVISG------KAVFI
DESK101258      ---HLEAVRIL-----------N-----TES-------N---KEEILKI------DGLFI
STAHD01472      ---KVRAVKLY-----------N-----KAT-------G---EEKIFEV------DGVFV
THEC100270      ---RVRAVRIE-----------N-----RAT-------G---EEKVIEV------DGVFV
NANEQ00478      ---KVESIIVQ-----------D------KE-------G---NTKELKV------DGVFV
CALLD01225      ---FVKSAVVL-----------N-----KEN-------N---NEREINI------DGIFI
SULSO02155      ---VVKQVVVE-----------N-----LKT-------G---EIKELNV------NGVFI
SULS900210      ---VVKQVVVE-----------N-----LKT-------G---EIKELNV------NGVFI
SULIA00208      ---VVRQVVVE-----------N-----LKT-------G---EIKELNV------NGVFV
SULIM00207      ---VVRQVVVE-----------N-----LKT-------G---EIKELNV------NGVFV
SULIK00225      ---VVRQVVVE-----------N-----LKT-------G---EIKELNV------NGVFV
IGNH400907      ---QKVTGLVV-----------E-----LPD-------G---TTKTLPV------RAVFI
KORCO01040      ---RVEGARVR-----------D-----LES-------G---EESFIEA------DGVFI
THESM01133      ---KVESVLLR-----------N-----IKT-------G---EIFEKKV------DGVFV
THEBM01534      ---KVESVVLK-----------N-----VKT-------G---EVFEKKV------DGVFI
METST01363      ---FLEEAVLY-----------N-----NET-------G---DETKVKV------SGIFI
METTH00703      ---RVEEVIIH-----------N-----RVT-------G---RDETLKV------DGVFI
METSL02406      ---FIESVVLY-----------N-----SKT-------K---QEQELKV------NGIFI
METLA02406      ---FIESVVLY-----------N-----SKT-------K---QEQELKV------NGIFI
METPW00194      ---FLKSVVIY-----------D-----RVR-------D---VEEELDI------AGMFI
RUBXD00226      ---AVEGLSLR-----------N-----VKT-------G---EESTLEV------EGFFV
RHOM400178      ---EVEGVRLR-----------N-----VKT-------G---EVSVLPV------KGLFI
GARV400051      ---EATSLTVR-----------N-----VIT-------G---ETQEVES------AGLFV
BIFAP01644      ---GVTSLDVR-----------N-----TAT-------G---QMSNIPA------NGVFV
SEGRD00014      ---SVTGVELS-----------H-----VAT-------G---QTSELPV------TGLFV
GORB404535      ---SVSGLELE-----------D-----TRT-------G---ETRNLDV------TGMFV
GORPV04920      ---SVTGLELI-----------D-----TRS-------G---DTQTVDV------TGMFV
MYCA904903      ---SVTGLKLE-----------N-----TVT-------G---QASELPV------TGMFV
MYCSS05356      ---KVTGIRLR-----------D-----TVT-------G---EESKLAV------TGVFV
MYCSJ05684      ---KVTGIRLR-----------D-----TVT-------G---EESKLAV------TGVFV
MYCSK05387      ---KVTGIRLR-----------D-----TVT-------G---EESKLAV------TGVFV
MYCS206581      ---KVTGVRLR-----------D-----TVT-------G---EESKLDV------TGVFV
MYCCN05158      ---KVTGIRLR-----------D-----TVT-------G---EESTLAV------TGVFV
MYCVP05890      ---KVTGIRLR-----------N-----TAT-------G---EESTLPV------TGVFV
MYCGI00775      ---KVTGIRLR-----------N-----SET-------G---EESRLEV------TGVFV
MYCSR04994      ---KVTGIRLR-----------N-----SET-------G---EESRLDV------TGVFV
AMYSD04545      ---SVTGLRLS-----------D-----TVT-------G---ESRELAV------TGMFV
MYCLE02681      ---TVTGLRLR-----------N-----TTT-------G---EETTLVV------TGVFV
MYCLB02681      ---TVTGLRLR-----------N-----TTT-------G---EETTLVV------TGVFV
MYCSD04323      ---TVTGLQVR-----------D-----TVT-------G---AETTLPV------TGVFV
MYCPA04306      ---TVTGLRLR-----------D-----TVT-------G---ETSTLAV------TGVFV
MYCA105023      ---TVTGLRLR-----------D-----TVT-------G---ETSTLAV------TGVFV
MYCUA04107      ---TVTGLQLR-----------D-----TVT-------G---EETTLPV------TGVFV
MYCMM05378      ---TVTGLQLR-----------D-----TVT-------G---EETTLPV------TGVFV
MYCA003905      ---TVTGLRVR-----------D-----TNT-------G---AETTLPV------TGVFV
```

```
MYCTU03943      ---TVTGLRVR-----------D-----TNT-------G---AETTLPV------TGVFV
MYCTF03846      ---TVTGLRVR-----------D-----TNT-------G---AETTLPV------TGVFV
MYCTA03980      ---TVTGLRVR-----------D-----TNT-------G---AETTLPV------TGVFV
MYCTK04010      ---TVTGLRVR-----------D-----TNT-------G---AETTLPV------TGVFV
MYCTC03612      ---TVTGLRVR-----------D-----TNT-------G---AETTLPV------TGVFV
MYCTD03548      ---TVTGLRVR-----------D-----TNT-------G---AETTLPV------TGVFV
MYCCP03898      ---TVTGLRVR-----------D-----TNT-------G---AETTLPV------TGVFV
MYCBO02863      ---TVTGLRVR-----------D-----TNT-------G---AETTLPV------TGVFV
MYCBP03913      ---TVTGLRVR-----------D-----TNT-------G---AETTLPV------TGVFV
MYCBT03913      ---TVTGLRVR-----------D-----TNT-------G---AETTLPV------TGVFV
TSUPD04078      ---SVESLAIE-----------N-----TVT-------G---ASATLEV------TGLFV
ACTMD06897      ---AVSGLKIR-----------D-----TRT-------G---ETAEHPF------TGFFL
SACES08402      ---TVSGVKVK-----------D-----TRT-------G---EVTEHPF------TGFFV
SACVD03814      ---KVSGLKLR-----------D-----TVT-------G---EESTLDV------TGFFL
AMYMU09190      ---KVEGLQLK-----------D-----TKD-------G---SESTLDV------SGFFV
AMYMS10178      ---KVEGLQLK-----------D-----TKD-------G---SESTLDV------SGFFV
PSEUX06421      ---SVAHLRLR-----------D-----TVT-------G---EESTLDV------TGMFV
NOCFA05681      ---SVTGLTLR-----------D-----TRT-------G---ETSHLAA------TGLFV
NOCCG05476      ---SVTGLTLR-----------D-----TRT-------G---ETSELAA------TGMFV
RHOE406010      ---SVTGLVVR-----------D-----TVT-------G---ETSTLEI------TGMFV
RHOOB03468      ---SVTGLVVR-----------D-----TVT-------G---ETSTLDV------TGMFV
RHOE104515      ---SVTGLVIR-----------D-----TVT-------G---EQSTLDV------TGMFV
CORDI02302      ---TVSGLELT-----------D-----TVT-------G---ETSVLDA------TAMFV
CORD202219      ---TVSGLELT-----------D-----TVT-------G---ETSVLDA------TAMFV
CORDL02208      ---TVSGLELT-----------D-----TVT-------G---ETSVLDA------TAMFV
CORDJ02208      ---TVSGLELT-----------D-----TVT-------G---ETSVLDA------TAMFV
CORDH02221      ---TVSGLELT-----------D-----TVT-------G---ETSVLDA------TAMFV
CORD702316      ---TVSGLELT-----------D-----TVT-------G---ETSVLDA------TAMFV
CORD302337      ---TVSGLELT-----------D-----TVT-------G---ETSVLDA------TAMFV
CORDD02235      ---TVSGLELT-----------D-----TVT-------G---ETSVLDA------TAMFV
CORDV02170      ---TVSGLELT-----------D-----TVT-------G---ETSVLDA------TAMFV
CORDW02254      ---TVSGLELT-----------D-----TVT-------G---ETSVLDA------TAMFV
CORDK02230      ---TVSGLELT-----------D-----TVT-------G---ETSVLDA------TAMFV
COREF02870      ---KVAGLKLK-----------D-----TVT-------G---ELSDLDV------TAMFV
CORGL03082      ---KVSGLKLN-----------D-----TVT-------G---EDSVLDV------TAMFV
CORGK02974      ---KVSGLKLN-----------D-----TVT-------G---EDSVLDV------TAMFV
CORGB03038      ---KVSGLKLN-----------D-----TVT-------G---EDSVLDV------TAMFV
CORK402006      ---PVDHLILE-----------D-----TQT-------G---EQRTLTA------SALFV
CORJK02028      ---SVETLVVE-----------D-----TQN-------G---EQKDVAM------DAMFV
CORVD02951      ---TVQALRLR-----------D-----TVT-------G---ETREIPM------DAMFV
ARCHD01706      ---GLEALTLE-----------D-----TVT-------G---ETSELPV------SGLFV
THET101205      ---NVKAIKVR-----------N-----VVT-------G---ELREIPV------DGVFV
PROAC02247      ---KLESITLR-----------D-----TKT-------G---EERQLEV------AGVFE
PROAS02302      ---KLESITLR-----------D-----TKT-------G---EERQLEV------AGVFE
CAERE29798      ---QVTGLTLR-----------D-----TVT-------G---AERTLGA------TGVFV
CELFA03749      ---KVTGVTLR-----------D-----TVT-------G---QTREHPA------TGVFV
JONDD02484      ---KVSGVRLR-----------D-----TVT-------Q---EETEHDA------TGVFI
XYLCX03324      ---QVSGVVLR-----------D-----TVT-------G---EVRPLDV------TGLFV
ACIC102144      ---RVEGVLLE-----------D-----TRT-------G---DQETLPV------TGLFI
FRADG04042      ---RVTGVQVR-----------D-----TVT-------G---ETSEIAA------SGLFV
FRASU07065      ---KLTGVRLK-----------D-----TVT-------G---ADDKLDV------TGLFV
FRASN07115      ---KVTGVRLR-----------D-----TVT-------D---KTDELAI------TGLFV
FRASC04424      ---KLTGVRLR-----------D-----TVT-------G---EAKDLPV------TGLFV
FRAAA06712      ---KLTGVRLR-----------D-----TVT-------G---ETDELEV------TGLFV
KYTSD02521      ---KLTGITLR-----------D-----TVT-------G---QERELPV------TGLFI
KINRD04462      ---KVEGLTLR-----------D-----TVT-------G---ETREIPA------TGLFI
NOCDD04789      ---RVSGLKVL-----------D-----TQT-------G---ETSTLDV------TGLFV
NOCAA01941      ---RVSGLKLR-----------N-----TRT-------G---EGDTLDV------TGLFV
STRRD08913      ---KVSGVRLR-----------N-----LKT-------G---EESELPA------TGLFI
THECD04863      ---RVTGVRVR-----------N-----RKT-------G---EESTLQV------TGLFI
THEBD03528      ---RVTGVRVR-----------N-----VHT-------G---EETELQV------TGVFI
CATAD08894      ---HVTGVKLR-----------N-----LKT-------G---EETLRPA------GALFV
KRIFD06917      ---KLTGVTLR-----------D-----TVT-------G---ETRALPV------SGLFI
KITSK03730      ---KLSGLTLR-----------D-----TNT-------G---DLRELPV------TGLFI
STRBB05366      ---KLSGLTLR-----------D-----VKT-------G---ESSELPV------TGLFI
```

```
STRVP03660      ---KLSGVTLR-----------D-----TKT-------G---ETRALPV------TGLFI
STRSW04381      ---KLAGLKLR-----------N-----LKT-------G---ETSELPV------TGLFI
STRGG03562      ---KLSGLTLR-----------N-----TKT-------G---ETSDLPV------TGLFI
STRFA03098      ---KLSSLTLR-----------N-----TKT-------G---ETSELPV------TGLFI
STRAW04303      ---KLAGLSLR-----------N-----LKT-------G---EISELPV------TGLFI
STRCO02929      ---KLAGLKLR-----------N-----VKT-------G---ELSDLPV------TGLFI
STRHJ05105      ---KLSGLKLR-----------N-----VKT-------G---ELSDLAA------TGLFI
MONBE04991      --KLMTSAIIE-----------D-----KKT-------G---EKREVEA------NGIFF
CHLRE01313      -KGLLGGVKVK-----------D-----VVT-------G---ELHDLPV------SGLFF
MEDTR25591      -NKKLGGLKVE-----------N-----VVT-------K---EVTDLKV------SGLFF
SOLLC13750      --KLLGGLKVK-----------N-----IVT-------G---EVSDLNV------SGLFF
PRUPE10733      -KGVLGGLKVK-----------N-----LVT-------G---EVSDLKV------SGLFF
MANES18605      --RLLGGLKVK-----------N-----IET-------G---QVCDLKV------SGLFF
THECC00884      -RGVLGGLKVK-----------N-----LLT-------G---EVSDLKV------SGLFF
PHYPA31147      -RGLLAELTVK-----------N-----LVT-------G---AESGLEV------SGLFF
AMBTC19471      -KGVLGGVKVK-----------N-----MKS-------G---EVKDLKV------SGLFF
MUSAC26038      -DGPLAGVKVR-----------N-----VVT-------G---EVSDLKV------SGLFF
MUSAM33177      -DGPLAGVKVR-----------N-----VVT-------G---EVSDLKV------SGLFF
SETIT03079      -GGPLAGVKVK-----------N-----LVS-------G---EVSDLQV------SGLFF
ORYBR12195      -GGPLAGVKVK-----------N-----LVD-------G---KITDLQV------AGLFF
COCLU07729      -NGLMTHLVVK-----------N-----NVT-------Q---EEQTLEA------KGLFY
PHANO13702      -NGLMTELVVK-----------N-----NVT-------K---EEQTVPA------RGLFY
PHAND10804      -NGLMTELVVK-----------N-----NVT-------K---EEQTVPA------RGLFY
AURPU02089      -RGLMTGLTIK-----------D-----IKT-------G---KEEDIAA------NGLFY
ZYMTR07711      -RGLMQSMVIK-----------N-----NKT-------G---ETEEIPA------NGLFY
DICPU05926      ---KVNAVGIE-----------N-----VKT-------G---EKSDLEA------QGLFY
ENTHI00522      --DLLNGAKIH-----------N-----LVS-------G---EYKVVPV------AGLFY
LEPBA02231      --GGLTSIVLE-----------S-----TKD-------K---TQKDLEV------GGLFY
LEPBP02301      --GGLTSIVLE-----------S-----TKD-------K---TQKDLEV------GGLFY
LEPBL01462      --KNLTALTLE-----------D-----TMS-------G---QKKELSV------GGLFY
LEPBJ01276      --KNLTALTLE-----------D-----TMS-------G---QKKELSV------GGLFY
LEPIN02475      --KSLTSLTLE-----------N-----TTN-------G---QKKELPV------GGLFY
LEPII01996      --KSLTSLTLE-----------N-----TTN-------G---QKKELPV------GGLFY
LEPIC01426      --KSLTSLTLE-----------N-----TTN-------G---QKKELPV------GGLFY
SPIAZ00697      ---MLEQVRVR-----------N-----NKT-------G---EEQLLPA------KGLFY
PENRW10140      -NGLMTHLRVK-----------D-----VLS-------N---AEEVVEA------NGLFY
PENCH09104      -NGLMTHLRVK-----------D-----VLS-------N---AEEVVEA------NGLFY
EURHE07269      -MGLMTHLRVQ-----------D-----TIS-------G---QEEVVDA------NGLFY
ASPAC07301      -MGLMTHLRVK-----------N-----VVS-------G---EEEVVDA------NGLFY
EMENI10387      -NGLMTHLRVK-----------N-----VLT-------G---EEETLEA------NGLFY
EMEND02596      -NGLMTHLRVK-----------N-----VLT-------G---EEETLEA------NGLFY
ASPTN06742      -MGLMTHLRVK-----------N-----TVT-------G---DEETVDA------NGLFY
ASPCL04014      -NGLMTHLKVK-----------N-----TVT-------G---DEEVVDA------NGLFY
ASPFU05647      -NGLMTHLRIK-----------N-----TVT-------G---EEEIVDA------NGLFY
NEOFI00452      -NGLMTHLRVK-----------N-----TVT-------G---EEEVVDA------NGLFY
CRYPA10563      -KGLMSHLVVK-----------N-----VQT-------G---AEETLEA------NGLFY
BLUGR03498      -KGLMDRLIIK-----------D-----VQT-------G---TQETIEA------NGLFY
SCLS112814      -KGLMTHMVFK-----------N-----VVT-------G---EEEKAEA------NGLFY
MAGGR04266      -DGLMSHMVIK-----------D-----VVT-------G---KEETLEA------NGLFY
NEUCR01575      -KGLMSHLVVK-----------D-----VTT-------G---KEETLEA------NGLFY
NEUT908941      -KGLMSHLVVK-----------D-----VTT-------G---KEETLEA------NGLFY
VERDA02342      -KGLMSHLVVR-----------D-----TRT-------G---AEETVEA------NGLFY
COLSU12486      -DGLMTHLVVQ-----------D-----TVT-------G---KEETLEA------NGLFY
HYPAI01684      -KGLMSHLVVK-----------N-----VVS-------G---EEETLEA------NGLFY
HYPVG06080      -DGLMSHLVIK-----------N-----VVT-------G---EEETVEA------NGLFY
HYPJE05895      -DGLMSHLVVK-----------N-----VVT-------G---AEETLEA------NGLFY
NECHA05020      -DGLMSHLVVK-----------N-----NVT-------G---EEETVEA------NGLFY
FUSO415847      -DGLMSHLVVK-----------N-----NKT-------G---QEEVHEA------NGLFY
GIBZA01026      -DGLMSHLVVK-----------N-----NKT-------G---EEKVHEA------NGLFY
SCHPO04025      --KLLNNLRIK-----------N-----TNT-------N---EVSDLQV------NGLFY
YARLI03635      --KLLNALEIE-----------N-----TLT-------G---EKRDLEV------NGLFY
ASHGO00946      --SLLDALRVR-----------D-----TRT-------G---EESDLPV------NGLFY
KLULA02190      --KYLNALKVK-----------N-----VKT-------N---EEYDLPV------NGLFY
ZYGRO00676      --KLLNTLRIK-----------N-----VKE-------N---QESDLQV------NGLFY
DEKBR01813      --KFLNALGIK-----------N-----VKT-------G---ETKDLAV------NGLFY
```

PICPG04776	--KLLSALTIE-----------N-----VVT-------K---EVKDLPV------SGLFY
CANTE00916	--KLLTGLEII-----------N-----NKT-------K---ETSLLPV------NGLFY
LODEL03891	--NLLQALKIY-----------N-----NKT-------K---EESELKV------NGLFY
DEBHA05546	--KLLQNISVY-----------N-----NKT-------K---ETKDLPV------NGLFY
SPAPN03477	--KLLNALTIK-----------N-----NKS-------G---ETKDLPV------NGLFY
CANAW04800	--KLLKSLRIV-----------N-----NKT-------K---KEKDLQV------NGLFY
PICST04701	--KLLQSLSIF-----------N-----NKT-------N---VTSDLPV------NGLFY
PUCGT10887	--NLLNSLLVK-----------D-----TKT-------G---EERTIDI------NGLFY
PUCGR11813	--NLLNSLLVK-----------D-----TKT-------G---EERTIDI------NGLFY
PHYBL11006	--KLLGSVATK-----------D-----TVT-------G---ATGSIPA------NGLFY
USTMA03757	--ELLQAVRVK-----------D-----TKT-------N---EERDMDA------NGLFY
USTHO04132	--ELLKAVRVK-----------D-----TKT-------G---EERDMDA------NGLFY
WALSE04527	--DLLQALTLK-----------D-----VQS-------G---ETRKLDV------NGLFY
TREME07701	--DLLQALVLK-----------D-----TKT-------G---ENRDLQV------NGLFY
AURST04751	--DLLNRLRIK-----------N-----LKT-------G---EEKELPV------NGLFY
FOMME10177	--NLLNALRIK-----------N-----TVT-------G---KEDDLQV------NGLFY
CONPW06392	--NLLNNLRIK-----------N-----IKT-------G---EEKDLPV------RGLFY
STEHR07076	--DLLNNLRIK-----------N-----THS-------G---AESDLAV------NGLFY
HETAN06295	--DLLNNLRIK-----------N-----TTT-------G---VESDLAV------NGLFY
GLOTR06982	--DLLNNLRIK-----------N-----VKT-------G---EEKDLPV------NGLFY
PUNST01981	--DLLNNLRIK-----------N-----VLS-------G---EERDLPV------NGLFY
LACBI02877	--DLLNNLRIK-----------N-----VQT-------G---EERDLPV------NGLFY
COPCI16429	--DLLNNLKIK-----------N-----VLT-------G---EERDLAV------NGLFY
DICSQ11618	--DLLNNLRIR-----------N-----VLT-------G---EEKDLAV------NGLFY
TRAVS13180	--DLLNNLRIK-----------N-----VQT-------G---EEKDLPV------NGLFY
WOLCO03584	--DLLNNLRIK-----------N-----VVT-------G---EEKDLPV------NGLFY
FOMPI05979	--DLLNNLRIK-----------N-----MLT-------G---EEKDLPV------SGLFY
PHLGI10219	--DLLNNLRIK-----------N-----IVS-------G---EEKDLPV------NGLFY
PHACH05757	--DLLNNLRIK-----------N-----VLT-------G---EEKDLPV------NGLFY
RICTY00419	--KTVTGVKIK-----------N-----VYT-------N---EINLVNC------SGVFI
RICPR00429	--KAVTGVKIQ-----------N-----VYT-------N---EINLVNC------SGVFI
RICPP00461	--KAVTGVKIQ-----------N-----VYT-------N---EINLVNC------SGVFI
RICBR00434	--KSVTGVKIQ-----------N-----VHT-------K---ETSLVNC------SGVFV
RICB800988	--KSVTGVKIQ-----------N-----VHT-------K---ETSLVNC------SGVFV
RICCK00600	--KSVTGVKIQ-----------N-----VHT-------K---EISLVNC------SGVFV
RICAH00614	--KSVTGVKIQ-----------N-----VHT-------K---EISLLNC------SGVFI
RICAC00782	--KSVTGVKIK-----------N-----VHT-------K---EISLLNC------SGVFI
RICFE00656	--KSVTGVKIQ-----------N-----VHT-------K---EISLVNC------SGVFI
RICMS00042	--KSVTGVKIQ-----------N-----VHT-------K---EISLLNC------SGVFI
RICM500453	--KSVTGVKIQ-----------N-----VHT-------K---EISLLNC------SGVFI
RICR300665	--KSVTGVKIQ-----------N-----VHT-------K---EISLLNC------SGVFI
RICAG00651	--KSVTGVKIQ-----------H-----VHT-------K---EISLLNC------SGVFI
RICP300630	--KSVTGVKIQ-----------N-----VHT-------K---ESSLLNC------SGVFI
RICRS00632	--KSVTGVKIQ-----------N-----VHT-------K---EISLLNC------SGVFI
RICRO00663	--KSVTGVKIQ-----------N-----VHT-------K---EISLLNC------SGVFI
RICCN00618	--KSVTGVKIQ-----------N-----VHT-------K---EISLLNC------SGVFI
RICPT00620	--KSVTGVKIQ-----------N-----VHT-------K---EISLLNC------SGVFI
RICAE00497	--KSVTGVKIQ-----------N-----VHT-------K---EISLLNC------SGVFI
RICJY00467	--KSVTGVKIQ-----------N-----VHT-------K---EISLLNC------SGVFI
RICPU00072	--KSVTGVKIQ-----------N-----VHT-------K---EISLLNC------SGVFI
RICS100537	--KSVTGVKIQ-----------N-----VHT-------K---EISLLNC------SGVFI
BARBK00891	TGSVVTGARLK-----------N-----VKT-------N---QKLKVDA------EGIFI
BARVW00932	RGAVVTGARLK-----------N-----VKT-------G---QEMKVNA------EGIFI
BART100617	RGAVVTGARLK-----------N-----VKT-------G---DEMKVEA------EGIFI
BARGA00466	VGAVVTGARLK-----------N-----VKT-------G---HEMTVNA------EGIFI
BARHE01107	IGAVVTGVRLK-----------N-----VKT-------G---QKVHVDT------DGIFI
BARQU00895	MGAVVTGVRLK-----------N-----IKT-------G---QKIKVDT------DGIFI
OCHA401642	MGATVTGVRLK-----------N-----VVT-------G---ETQELDT------HGVFV
BRUAB01390	MGATVTGVRLK-----------N-----IVT-------G---ETQERAT------HGVFI
BRUA201507	MGATVTGVRLK-----------N-----IVT-------G---ETQERAT------HGVFI
BRUA101339	MGATVTGVRLK-----------N-----IVT-------G---ETQERAT------HGVFI
BRUSU01458	MGATVTGVRLK-----------N-----IVT-------G---ETQERAT------HGVFI
BRUME00510	MGATVTGVRLK-----------N-----IVT-------G---ETQERAT------HGVFI
BRUSI01461	MGATVTGVRLK-----------N-----IVT-------G---ETQERAT------HGVFI
BRUC201446	MGATVTGVRLK-----------N-----IVT-------G---ETQERAT------HGVFI

```
BRUMC01440      MGATVTGVRLK-----------N-----IVT-------G---ETQERAT------HGVFI
BRUMB01421      MGATVTGVRLK-----------N-----IVT-------G---ETQERAT------HGVFI
BRUM501487      MGATVTGVRLK-----------N-----IVT-------G---ETQERAT------HGVFI
BRUO201285      MGATVTGVRLK-----------N-----IVT-------G---ETQERAT------HGVFI
RHILO01973      LPPSVEGLKLK-----------H-----AVT-------G---AETHLKV------DGVFV
CHESB02097      LPPSVTGIRVQ-----------N-----VQT-------G---EVRDLPV------DGVFV
METPB00996      --PSVTHLRLK-----------D-----LRT-------G---EIVEQPT------DGLFV
METEP01063      --PSVTHLRLK-----------D-----LRT-------G---AIVEQPT------DGLFV
METEA00810      --PSVTHLRLK-----------D-----LRT-------G---AIVEQPT------DGLFV
METED01453      --PSVTHLRLK-----------D-----LRT-------G---AIVEQPT------DGLFV
METS403554      -APSVTHVRLR-----------D-----VAT-------G---AVSERRA------DGVFV
METNO05482      -APSVTHVRLK-----------D-----VRT-------G---LISERKA------DGVFV
METSZ03234      --LSVTGARVK-----------N-----VAT-------G---AIHTLAA------DGVFV
BEII900056      --LNVTHVRLK-----------N-----TKT-------G---AIETRET------HGVFV
METSB02743      --PGVTHVTLK-----------N-----AVT-------G---ALQPIEA------HGVFV
MAGMM00401      --GGLTGVRIK-----------N-----VKT-------G---ALSELTV------TGCFI
HYPNA00542      --LGVTAARIK-----------D-----VTT-------G---AIRELPV------HGVFV
KETVY00902      --RGVEGVRLR-----------D-----VNT-------G---DVRDLAA------KGFFV
KETVW00472      --RGVEGVRLR-----------D-----VNT-------G---DVRDLAA------KGFFV
ROSDO03242      --LGVEAIRVR-----------H-----TKT-------S---EITEIPA------KGVFV
ROSLO02615      --LGVEAVRVR-----------N-----TKT-------S---EITEIPA------KGVFV
RUEPO00888      --LGVEGVKVR-----------N-----VKT-------G---EITDIPC------KGVFV
RUEST00613      --LGVEAVRVK-----------H-----TKT-------G---EITEIPA------KGVFV
PHAIB02390      --LGVEAVRVK-----------N-----VKT-------D---EITEIPC------KGVFV
PARDP02130      --PGVTGVRVR-----------H-----VRQ-------G---TEQVVPA------DGVFI
DINSH02620      --RGVEGVVIK-----------H-----RDT-------G---ETRTLPC------AGFFV
RHOCB02768      --LGVTGCRIR-----------D-----VQT-------G---AEREIPC------HGFFV
RHOS500248      --LGVTGVVAK-----------H-----AQT-------G---ETVEIPC------AGFFV
RHOS400150      --LGVTGIVAR-----------N-----VLT-------G---ETTEVPC------EGFFV
RHOS100227      --LGVTGIVAR-----------N-----VLT-------G---ETTEVPC------EGFFV
RHOSK02952      --LGVTGIVAR-----------N-----VLT-------G---ETTEVPC------EGFFV
MIDMI00790      --LSVTGVRLR-----------D-----ILT-------G---KEYTMDI------DGVFV
ACEP301595      --ETVCGVQLK-----------N-----TQD-------G---SKQTIPT------DGVFI
MICAA01566      GGAGVTGLSLK-----------N-----VKT-------G---DVSKLDV------DGMFV
TISMK03676      --RGVTGVRLK-----------D-----TAD-------G---SLRDLNV------DGVFI
AZOL402409      NPRAVTGVRIK-----------N-----VKS-------G---EERVIPV------AGVFV
PSEUV04348      --AVVTGVRLK-----------N-----RKT-------G---ETTEMST------DGVFI
HIRBI01095      --LGVTGVKLK-----------N-----VKT-------G---EIIDREA------HGVFI
PARL102241      --PGVTGARIK-----------N-----VKT-------G---ETRELKA------DGVFM
MARMM02139      --AGVTAVRVK-----------N-----VKT-------G---ATTDIPA------HGFFV
PHEZH02588      --LGVTGVRLK-----------N-----VKT-------G---ETREIPC------DGVFI
CAUCR02826      --MGVTGARLK-----------N-----VKT-------G---ETQEVAA------DGVFI
CAUCN02939      --MGVTGARLK-----------N-----VKT-------G---ETQEVAA------DGVFI
CAUST00891      --MGVTGVRLK-----------N-----IKT-------G---ATTDLAC------DGVFI
PARBH01522      --LGVEGVRLA-----------S-----TVG-------E---DSMELAI------HGLFI
PELHB02084      --PSVRGAVLR-----------D-----VST-------D---RTHEIEV------DGIFV
HYPDA03273      --KSVTGVVLK-----------N-----VKT-------G---VTSKIPA------DGFFV
HYPSM04642      --KSVTGIVLK-----------N-----IKT-------G---MTSELAT------DGFFV
OLICO01107      --SKVTHVRLR-----------N-----LKT-------G---AITERTT------DGVFI
OLICM02790      --SKVTHVRLR-----------N-----LKT-------G---AITERTT------DGVFI
RHOPS01446      --TKVTHVRLR-----------N-----VKT-------G---ATSDVRA------DGVFI
RHOPA04062      --TKVTHVRLK-----------N-----VKS-------G---TTSEVKA------DGVFI
RHOPT04533      --TKVTHVRLK-----------N-----VKS-------G---TTSEVKA------DGVFI
RHOPX04235      --TKVTHVRLK-----------N-----VRS-------G---ATSEIKA------DGVFI
BRADU07321      --NKVTHVRLK-----------N-----VKT-------G---KLTELKA------DGIFI
BRASO01239      --SKVTHVRLK-----------N-----VKT-------G---ALTELPA------DGVFI
BRASB06352      --SKVTHVRLK-----------N-----VKT-------G---AVSELPA------DGVFV
RHOPB03773      --TKVTHVRLK-----------N-----VKT-------G---ALTECAA------DGVFI
NITWN02324      --PKVTHVRLK-----------N-----VKS-------G---ELTEVEA------DGVFI
NITHX02707      --PKVTHVRLK-----------N-----VKT-------G---ALTEVAA------DGVFI
AZOC501143      --SKVTGVRLK-----------D-----VNT-------G---AHAELAT------DGVFI
XANP202679      --TKVTGAKLR-----------D-----VRT-------G---VLTELPA------DGIFI
CHLTF01983      G---VTGLRLR-----------N-----LKT-------D---EVFDYKC------DGLFV
IGNAJ02236      GKKRMTGVLLE-----------D-----TKD-------H---SVTQLDA------DGLFI
MELRP00403      GRKSVTGAILE-----------N-----TKD-------G---STTEIQA------DGIFI
```

```
ANADF00468       --KAVTGVRLR-----------S-----TKD-------G---SLREVPL------KGVFM
CHLCH01125       --FKVTGVRLK-----------N-----VAT-------G---EVSDHSC------DGLFL
PELPB01419       --QKVTGIRLK-----------N-----VLT-------G---ELTEHPC------DGVFL
CHLL200781       --SKVTGIRLK-----------D-----VRT-------G---ELIDHVC------DGVFV
CHLTE00830       --MKVTGIRLK-----------N-----VKT-------G---ELTEHAC------DGVFI
CHLP800854       --MKVTGIRLK-----------N-----VKT-------G---ELTEHAC------DGVFV
CHLL701225       --QKVTGIRLK-----------N-----VKT-------G---ELEEHAC------DGVFM
CHLPM00993       --QKVTGIRLK-----------H-----VET-------G---ELEELPC------DGVFM
WOLTR00562       -PKKVTGITIK-----------S-----TKI-------D---EIQELRV------NGVFI
WOLPP00135       -PKKVTGITIK-----------S-----TDI-------N---KTQELKV------DGVFI
WOLPM00650       -PKRVAGIAIK-----------S-----AEI-------D---KTQELKV------DGVFI
WOLWR00416       -PKRVAGIAIK-----------S-----AE-------------TQELKV------DGVFI
EHRCR00696       -SGNVEAIALK-----------S-----VKT-------G---DITTISV------KGVFI
ANAMM00345       -NKKVGALLLE-----------S-----TLD-------G---STRTLET------AGVFI
ANAMF00341       -NKKVGALLLE-----------S-----TLD-------G---STRTLET------AGVFI
ANAPZ00610       -GKKVTSLLLQ-----------S-----TVD-------S---TESLLDV------GGVFV
NEOSM00537       -PPEVTGVRVR-----------S-----LMD-------G---TFREILV------SGVFV
NEORI00513       -PPEVTGVRVR-----------S-----LID-------G---TFKEILV------SGVFV
PELUB00076       -PKNVKAIKIK-----------N-----LKT-------N---KIEEMKI------DGLFI
PELSM00736       -PKGVTGVLLE-----------N-----TKD-------K---TTKQLNT------HGVFV
PUNMI01409       ---GVTGVRLA-----------S-----TTG-------E---ADKEIAV------HGMFV
ASTEC00651       GGASVTGVRLK-----------N-----VHT-------G---DTHELPL------DGVFV
ZYMMT00154       --SALAAIELE-----------N-----TKT-------G---ALSRLEV------EGAFI
ZYMMO00984       --SALSAVKLK-----------D-----TKT-------G---EESLLET------EGAFI
ZYMMA00176       --SALSAVKLK-----------D-----TKT-------G---EESLLET------EGAFI
ZYMMN00183       --SALSAVKLK-----------D-----TKT-------G---EESLLET------EGAFI
SPHAL00143       -VSGLVGVDLI-----------D-----TVT-------G---AASHEPT------DGGFV
SPHWW03978       -NEGLVAIALR-----------D-----TET-------G---ETSELAV------DGGFV
SPHJU02313       -PEGLVGVDLV-----------D-----TVT-------G---HKSHIAT------DGAFV
NOVAD02319       --KGLTGVKLV-----------D-----TVT-------G---AESVVET------DGAFV
ERYLH00934       --GMLSHLVLK-----------D-----TQT-------G---ELSNLEV------DGAFV
GRABC00720       -PDRVDGIVLR-----------N-----TIK-------G---DTSTLPV------DGVFV
GLUDA03075       -PPVVTGLNLR-----------D-----ISS-------G---TNEHLAV------DGVFV
KOMMN00774       -PPVVTGLELR-----------D-----TKT-------G---AIHDIAV------DGVFV
HALVD01097       ---GVDHVTLVRNPEGHPSDKLD-----DPE-------T--EEFD-FDV------GAVFY
HALHT01699       ---GVDHVTLAQNDAGHPSEKLD-----ADG-------T--ETFD-FDV------GAVFI
METI401038       ---KVTGVWLR-----------N-----LKD-------G--SKNF-LEC------RGVFV
PLAL201612       ---GVTGVRLK-----------S-----LKD-------D--SERT-LEA------AGMFC
GEMAT01983       ----ITGVRLE-----------D-----VST-------G--ATSE-LEA------GGLFV
CYAAP02456       ----LQGIQVQ-----------N-----TLT-------K--EVSE-FSV------NGLFY
CHLPN00303       ----VRSVDIK-----------N-----VQT-------Q--EITT-REA------AGVFF
CHLPP00439       ----VRSVDIK-----------N-----VQT-------Q--EITT-REA------AGVFF
CHLPE00727       ----VRAVDIK-----------N-----NVT-------E--QVET-REA------AGVFF
CHLTR00101       ----VRSIDIY-----------N-----NVD-------E--TTTT-MEA------AGVFF
CHLTA00102       ----VRSIDIY-----------N-----NVD-------E--TTTT-MEA------AGVFF
CHLTJ00101       ----VRSIDIY-----------N-----NVD-------E--TTTT-MEA------AGVFF
CHLTD00098       ----VRSIDIY-----------N-----NVD-------E--TTTT-MEA------AGVFF
CHLT700102       ----VRSIDIY-----------N-----NVD-------E--TTTT-MEA------AGVFF
CHLT000101       ----VRSIDIY-----------N-----NVD-------E--TTTT-MEA------AGVFF
CHLT500104       ----VRSIDIY-----------N-----NVD-------E--TTTT-MEA------AGVFF
CHLTL00098       ----VRSIDIY-----------N-----NVD-------E--TTTT-MEA------AGVFF
CHLTG00102       ----VRSIDIY-----------N-----NVD-------E--TTTT-MEA------AGVFF
CHLTS00101       ----VRSIDIY-----------N-----NVD-------E--TTTT-MEA------AGVFF
CHLT900102       ----VRSIDIY-----------N-----NVD-------E--TTTT-MEA------AGVFF
CHLTZ00101       ----VRSIDIY-----------N-----NVD-------E--TTTT-MEA------AGVFF
CHLT400432       ----VRSIDIY-----------N-----NVD-------E--TTTT-MEA------AGVFF
CHLT100103       ----VRSIDIY-----------N-----NVD-------K--TTTT-MEA------AGVFF
CHLT200350       ----VRSIDIY-----------N-----NVD-------K--TTTT-MEA------AGVFF
CHLTB00350       ----VRSIDIY-----------N-----NVD-------K--TTTT-MEA------AGVFF
CHLTC00378       ----VRSIDIY-----------N-----NVD-------K--TTTT-MEA------AGVFF
SIMNZ00831       ----VESVTLQ-----------N-----VNT-------K--EETK-REA------AGLFF
PARUW01706       ----VKNLVIR-----------N-----VKT-------G--QEKE-YEA------AGLFF
WADCW00943       ----VKKVTIQ-----------N-----VKT-------K--EENV-HDA------GGVFF
SINAD04980       ----V-RLTSL-----------D-----GKA-------G--ATRD-ITV------DGLFL
BIFLB01558       ---GVTSLDLR-----------N-----TAT-------N--ETSK-IEA------NGVFV
```

```
BIFAB00498      ---GVTSLDLR-----------N-----TAT-------N--ETSK-IEA------NGVFV
BIFAV01561      ---GVTSLDLR-----------N-----TAT-------N--ETSK-IEA------NGVFV
BIFAS01557      ---GVTSLDLR-----------N-----TAT-------N--ETSK-IEA------NGVFV
BIFA001518      ---GVTSLDLR-----------N-----TAT-------N--ETSK-IEA------NGVFV
BIFBA01814      APQNVSSIDIR-----------N-----TAT-------G--ETGT-LDT------SAVFV
BIFAA01619      ---GVQKVEVR-----------N-----TAT-------E--KTSV-IPA------NGVFM
BIFDB02112      ---GVNSISIR-----------N-----LKN-------E--SVEV-ISA------NGVFV
TERSS03091      ---EVRGIVIR-----------N-----VKS-------G--QTSE-IPL------AGFFL
GRATM03160      ---DVKGLRLK-----------N-----RAN-------G--DEST-LPV------SFMFL
GRAMM03836      ---DLKGLKLV-----------N-----RKT-------G--ERWM-LPV------SFMFL
AKKM801247      ---ELEAVMLK-----------D-----VVE-------G--DETE-LPV------KCVFM
OPITP04130      ---GVSGLRVE-----------N-----VKT-------G--ETRV-LPV------KGVFI
CORAD01794      ---FTRALLVK-----------D-----AES-------G--EERE-IPC------KGAFI
BUCCC00180      ----INGIIIQ-----------S-----KID-------N---TLLNLKI------TGLFI
CENSY00347      ----VQQVVLA-----------S-----P---------D--GEEQ-MDT------GGVFV
NITMS00668      ----MQQAVLK-----------N-----LKT-------N--EEST-LDV------GGLFV
MEIRD02823      ----VTGVRLR-----------N-----LKS-------G--EVYD-HPT------DGVFV
HERA203780      ----VEGVALH-----------N-----VKT-------G--EEST-FAA------DGVFP
CHLAA02018      ----VESVTLR-----------N-----LQT-------G--EVSE-LPT------DGVFP
CHLSY02175      ----VESVTLR-----------N-----LQT-------G--EVSE-LPT------DGVFP
CHLAD02599      ----VESVTLR-----------N-----LKT-------G--EMSE-LPT------DGVFP
CALAS01745      ----VTGVKTR-----------N-----TVT-------G--EAGE-LKT------DGVFV
ANATU01696      ----VKAVRLK-----------N-----VVT-------G--EEST-FST------DGVFI
SULMS00235      ----LEYLICK-----------K-----NSS-------N--EEKK-INV------KAMFI
BLASB00041      ----LEGVQIF-----------N-----HEK-------K--MNTT-LLV------NGLFI
BLASP00551      ----LEGIQIF-----------N-----TKK-------K--ISRT-ILI------SGLFI
AZOPC00437      ----VEGIHLI-----------K-----YRG-------EANEEKIDMTI------DGFFL
LEPBD01878      ----VTGIELI-----------N-----NKT---------GETETLT-A------DGIFV
SALRD02577      ----VEGIEVI-----------N-----NET---------GETRLMGDV------TGFFL
SALRM02866      ----VEGIEVI-----------N-----NET---------GETRVMGDV------TGFFL
RIEPU00166      -EKGITSIKIK-----------S-----NLN----------GSEKNLFV------SGIFV
ORITB00015      -AKTVTGVRIK-----------N-----SKN----------NTISTLEV------DGVFI
ORITI01234      -AKTVTGVRIK-----------N-----SKN----------NTISTLEV------DGVFI
PREMB01541      --DGVEGAHLV-----------R-----FKG-------EENEEKFDINI------DGFFL
PREDF01155      --NGVEGAHLV-----------R-----FKG-------EKNEEKYDISI------DGFFL
PREI702011      --NGVEGAHLV-----------R-----FKG-------EENEEKYDIQI------DGFFL
ALIFI01035      -ESGVTGALLR-----------C-----ND-----------GKEVRIDI------AGFFL
ODOSD00362      -DMGLEGARLL-----------Y-----RKG-------EPEECEKDIKI------DGFFL
PRER201250      --NGVEGAHLV-----------K-----RKG-------EADEELVDIAI------DGFFL
PALPW00206      --NVVQGADLV-----------K-----RRG-------QSDEEKVHIDI------DGFFL
PORGI00953      --NGVEGAHLV-----------K-----RKG-------EPDEEMVDIAI------DGFFL
PORG301168      --NGVEGAHLV-----------K-----RKG-------EPDEEMVDIAI------DGFFL
BACV803426      --NGVEGVHLV-----------K-----RQG-------ECNEERYDLPI------DGFFL
BACT601486      --DGVEGVHLV-----------K-----RAG-------EADEEYYDLAI------DGFFL
BACTN04290      --NGVEGMNVV-----------K-----RWG-------ESDEERYSLPI------DGFFL
BACFR01032      --NGVEGMNLV-----------K-----RWE-------EPDEERYSLPI------DGFFL
BACFN00913      --NGVEGMNLV-----------K-----RWE-------EPDEERYSLPI------DGFFL
BACF600963      --NGVEGMNLV-----------K-----RWE-------EPDEERYSLPI------DGFFL
OWEHD03413      -K-TVTGVKVK-----------N-----NQT----------GEEQTIPA------EGFFV
PSYTT00390      -Q-VVEGLRIL-----------N-----NKT----------GVKEEIKI------TGLFI
NONDD00011      -Q-VVEGLRIV-----------N-----NQT----------QEKHEIEI------TGIFI
ROBBH02711      -Q-VVEGLRMV-----------N-----NQT----------GEKEEVAI------TGLFI
CELAD02592      -Q-VVEGLRMV-----------N-----NVT----------GEKSEIDI------TGLFI
CELLC02474      -Q-VVEGLRMV-----------N-----NQT----------QEKEDIAI------TGLFI
MARSH03072      -Q-VVEGLRMV-----------N-----NQT----------NEKEDIAI------TGLFV
MURRD00159      -Q-VVEGIRVV-----------N-----NQT----------GEKEEIPV------TGLFI
ZOBGA00152      -Q-VVEGLRMV-----------N-----NQT----------GEKEDIAI------TGLFI
GRAFK00949      -Q-VVEGLRMV-----------N-----NQT----------GDKEEIDI------TGFFV
ZUNPS02344      -Q-VVDGLRMV-----------N-----NKT----------GEKEEIAI------TGLFI
AEQSU02079      -T-VVEGLRMV-----------N-----NET----------GEKKEIEI------TGLFI
HALH105745      -N-EVEGVKLL-----------N-----NKT----------NETSVIPV------KGFFV
SAPGL02893      -E-GVTGVRLI-----------N-----NKT----------KENSELSL------GAVFI
AMOA500014      -Q-EVEGVRLF-----------D-----KRS----------QTSFELLL------QGFFV
FLAIG01802      -GQVVTAVKVK-----------N-----SVT----------GEEKEIPA------TGFFV
FLACA02326      -GQVVTAVKAK-----------N-----KST----------GEITEIPA------TGFFV
```

```
FLAJ100198       -NNVVHAIKAL-----------N-----KTT----------GETIEIPA------TGFFV
FLABF01528       -GQVVTSILAK-----------N-----KTT----------NELVTIPA------TGFFV
CAPOD01333       -GQVVTGIKVR-----------N-----NKT----------GEISEIAI------TGFFV
CAPCC00414       -GQVVSGLRVK-----------N-----NKT----------GETSEIPV------TGFFV
FLELS01898       -GENVTSARLL-----------N-----NQK----------NESYEIAV------DGFFV
SOLCM01166       -GQNVTGALIV-----------N-----NET----------GEEKTIDI------TGFFV
PEDHD01598       -GKNVTAVRVV-----------N-----NQT----------GAETDLPV------EGFFV
SPHS203207       -GKTVNGLRII-----------N-----NQT----------KEHQVLDI------TGFFV
LEAB401188       -Q-GVNGARLK-----------N-----NKT----------GEEFEISV------DGFFV
EMTOG00092       -D-GVTGARIK-----------N-----SKT----------NEESVIDV------TGFFV
SPILD01737       -E-DVTGVRVK-----------N-----SET----------GEERILDV------TGFFV
DYAFD00084       -H-GLESVLVK-----------N-----NKT----------GEEQLLEA------TGFFV
CYCMS03063       -E-EVTGAKVR-----------D-----RIS----------GEEKVLAV------EAFFV
ECHVK03326       -E-EVTGVRVK-----------N-----NQT----------GEEQELEV------TGFFV
BELBD02542       -D-EVTGARLI-----------N-----NVS----------KEVSEIKV------SGFFV
NITGG02112       --SKVATVMVK-----------N-----VAS----------GKETTIKA------GGLFV
THEM700111       --DKVEGVRVR-----------N-----VKT----------GEVSQIPC------AAVFV
CREAS01350       --KKVTGVKLR-----------D-----LKT----------NQVYFYRC------DGVFI
TURPD02772       -TGTVTGVKLK-----------D-----TRS----------GAIEDFAV------QGLFI
SORC507603       -ADMVTGVRVK-----------S-----TKT----------GDSHLVPA------AAMFV
BDEBA00337       -KGM-TGAKIK-----------G-----TLD----------GAVKELPI------TGLFL
STIAD02752       -KGM-TGAVVR-----------N-----LKT----------NDTQLLNA------TGLFV
MYXXD01876       -KGM-NGAVVR-----------N-----LKT----------GDSQQVKA------TGLFV
MYXFH03454       -KGM-NGAVVR-----------N-----LKT----------GDSQLVKA------TGLFV
CORCM01960       -KGM-TGAVVR-----------N-----LKT----------GDSKLVNA------HGLFV
MYXSD02209       -KGM-TGAVVR-----------N-----LKT----------GDSQLLAA------TGLFV
LEPFC02126       -KGQVTGVRVK-----------D-----SVT----------GEISEIPC------QGFFL
LEPFM02294       -AGKVTGVVLK-----------D-----VVD----------GSISEYPC------EGFFL
SOLUE00582       -KKLVTHVRLR-----------N-----NKS----------GEVWDQEV------DGFFL
KORVE01333       -QKEVTGLTLR-----------N-----LKT----------DKVWDLPV------SAMFL
ACIC502553       -EKDVRGVRLR-----------D-----TVT----------GEESELAI------SGLFL
ACIFD00030       -DGVLVGVRTE-----------D-----TVT----------GEREDLGV------TGLFI
HALMS01402       -QTGVTSIKVE-----------N-----LKT----------GEVTERPT------NGLFM
CHLPD01133       -GHKVTGIRLQ-----------N-----VLT----------GEKTEHQC------DGVFM
CHLPB01137       -EQNVTGIRMK-----------D-----VLT----------GEIQETSC------DGVFV
PROA200897       -GRKVTGIRLK-----------N-----VVS----------GESNTHSC------DGVFI
WIGBR00492       ---QVSSILLE-----------S-----TTS----------KKEKIISV------SGIFI
BUCA500289       ---GVTALLIE-----------Q-----NNS-------K-EKTESKIQV------SGLFV
BUCAI00292       ---GVTALLIE-----------Q-----KNS-------K-EKTESKIQV------SGLFV
BUCAF00306       ---GVTALLIE-----------Q-----KNS-------K-EKTESKIQV------SGLFV
BUCAT00287       ---GVTALLIE-----------Q-----KNS-------K-EKTESKIQV------SGLFV
BUCA000302       ---GVTALLIE-----------Q-----KNS-------K-EKTESKIQV------SGLFV
BAUCH00290       ---MVTGIQLQ-----------H-----SNA-------D-K--LETLAV------SGVFI
BLOVB00369       ---EVTGLRIK-----------N-----TIL-------L-K--EYTIDL------KGVFI
BLOFL00367       ---EVTGINIQ-----------D-----KNH-------N-K--EHIINV------QGVFI
BLOPB00375       ---DVTGLCMT-----------D-----LKQ-------H-K--EHIINL------QGIFI
BUCAP00289       ---GVTHLLIK-----------N-----KNL-------K-EKKKLKIAV------SGLFV
COXBU01001       ---GVTGVHLK-----------H-----VKE-------E-K--TQDLTI------DGLFI
COXBN01188       ---GVTGVHLK-----------H-----VKE-------E-K--TQDLTI------DGLFI
COXBR01117       ---GVTGVHLK-----------H-----VKE-------E-K--TQDLTI------DGLFI
COXB200717       ---GVTGVHLK-----------H-----VKE-------E-K--TQDLTI------DGLFI
COXB100914       ---GVTGVHLK-----------H-----VKE-------E-K--TQDLTI------DGLFI
FRAP200241       ---GVNALRLK-----------N-----VKT-------N-E--ESKIDV------MGVFI
FRANT01012       ---GVNALRLK-----------N-----VKT-------N-E--ESKIDV------MGVFI
FRACN00522       ---GVNALRIK-----------N-----IKT-------N-E--ESQIDV------AGVFI
FRATT00465       ---GVNALRIK-----------N-----IKT-------N-E--ESQMGV------AGVFI
FRAT100465       ---GVNALRIK-----------N-----IKT-------N-E--ESQMGV------AGVFI
FRATE00462       ---GVNALRIK-----------N-----IKT-------N-E--ESQMGV------AGVFI
FRATW01216       ---GVNALRIK-----------N-----IKT-------N-E--ESQMDV------AGVFI
FRATM01110       ---GVNALRIK-----------N-----IKT-------N-E--ESQMDV------AGVFI
FRACF00566       ---GVNALRIK-----------N-----IKT-------N-E--ESQVDV------AGVFI
FRATO01212       ---GVNALRIK-----------N-----IKT-------N-E--ESQIDV------AGVFI
FRATH01486       ---GVNALRIK-----------N-----IKT-------N-E--ESQIDV------AGVFI
FRATF01216       ---GVNALRIK-----------N-----IKT-------N-E--ESQIDV------AGVFI
FRATN00564       ---GVNALRIK-----------N-----IKT-------N-E--ESQIDV------AGVFI
```

```
ACIF500512      ---GVTGLRIQ-----------H-----VEN-------G-S--TQDLAL------MGVFI
ACIF200362      ---GVTGLRIQ-----------H-----VEN-------G-S--TQDLAL------MGVFI
DECAR01284      ---GVTGLSIK-----------N-----VKS-------G-E--TQKVDV------HGVFI
NEIG100525      ---GVNGALLK-----------N-----N-D-------G-S--DQQIAV------SGIFI
NEIG201314      ---GVNGALLK-----------N-----N-D-------G-S--DQQIAV------SGIFI
NEIM800926      ---GVNGALLK-----------N-----N-D-------G-S--EQQIAV------SGIFI
NEIMP01199      ---GVNGALLK-----------N-----N-D-------G-S--EQQIAV------SGIFI
NEIMB01212      ---GVNGALLK-----------N-----N-D-------G-S--EQQIAV------SGIFI
NEIMF01143      ---GVNGALLK-----------N-----N-D-------G-S--EQQIAV------SGIFI
NEIML01119      ---GVNGALLK-----------N-----N-D-------G-S--EQQIAV------SGIFI
NEIMM00780      ---GVNGALLK-----------N-----N-D-------G-S--EQQIAV------SGIFI
NEIMH00827      ---GVNGALLK-----------N-----N-D-------G-S--EQQIAV------SGIFI
NEIMG01168      ---GVNGALLK-----------N-----N-D-------G-S--EQQIAV------SGIFI
NEIMN01254      ---GVNGALLK-----------N-----N-D-------G-S--EQQIAV------SGIFI
NEIMO00810      ---GVNGALLK-----------N-----N-D-------G-S--EQQIAV------SGIFI
NEIM701229      ---GVNGALLK-----------N-----N-D-------G-S--EQQIAV------SGIFI
NEIMA01334      ---GVNGALLK-----------N-----N-D-------G-S--EQQIAV------SGIFI
NEIMW01125      ---GVNGALLK-----------N-----N-D-------G-S--EQQIAV------SGIFI
DICNV01037      ---GVTGAIIR-----------F-----NNG-------Q----IEQLAV------DGIFI
VESOH00047      ---GVTGIRLK-----------N-----NNN-------K----IKDIDV------HGVFI
RUTMC00043      ---GVTGLRLK-----------D-----NDG-------K----TKNIDV------HGVFI
HALHL02255      ---GVTGIRLR-----------H-----TDS-------G-E--VSEREV------SGVFI
PELPD03177      ---GVTGMRIR-----------H-----KSG-------Q-S--TKEIPL------HGVFI
GEOS804030      ---GVTGIRLR-----------H-----SSG-------S----DKILNV------HGCFV
GEOBB03636      ---GVTGVRLR-----------H-----TSG-------S----DKLISV------HGCFI
GEOSM03700      ---GVTGVRLR-----------H-----TSG-------S----DKVISA------HGCFI
HALNC00046      ---GVTGLRIK-----------Q-----VQT-------E-K--TTDIAL------TGVFI
HAEPS00887      ---GVTGVRLK-----------C-----TQT-------G-N--EEQITL------DGVFV
GALAU02285      ---GVTHLQLK-----------D-----THS-------E-Q--QETLSV------DGVFV
HISS201000      ---GVTGIRLK-----------N-----TQT-------E-N--VEDLKL------EGVFI
HAES101151      ---GVTGIRLK-----------N-----TQT-------E-N--VEDLKL------EGVFI
PASMU00573      ---GVTGVRLK-----------D-----TQS-------E-A--TEELSL------DGVFI
PASMH00579      ---GVTGVRLK-----------D-----TQS-------E-A--TEELSL------DGVFI
ACTSZ01560      ---GVTGVRLQ-----------D-----TQS-------D-A--TEELKL------DGVFI
AGGAN00985      ---GVTGVRLQ-----------D-----VQS-------G-A--KEDVAL------DGVFI
NITHN03181      ---GVTGIRIK-----------E-----VNT-------G-Q--TKELPL------HGVFI
NITOC00311      ---GVTGIRIR-----------E-----VDT-------N-Q--TKKLPL------HGVFI
NITWC02498      ---GVTGIRIK-----------E-----VDT-------N-Q--TKKLPL------HGVFI
METNJ00648      ---GVTGIRVK-----------S-----TET-------D-N--TQEIAV------QGVFI
METFJ01616      ---GVTGIRVK-----------H-----SQD-------E-T--TQEVAV------QGVFI
ALKEH00244      ---GVTGLRLK-----------H-----TQT-------G-E--TRQVDV------HGVFI
MARMS03166      ---GVTGVRIK-----------N-----TQT-------G-E--KKEIAV------AGLFV
MARM102688      ---GVTGIRIL-----------N-----NQS-------G-E--KKDLEI------AGLFV
THICR00763      ---GVNGLRIK-----------N-----NQT-------N-E--SKEIDV------AGVFI
THICA00974      ---GVTGLRIR-----------E-----RDG-------D-A--TQDLDV------AGVFI
THIV600851      ---GVTGIRIK-----------S-----TLD-------G-T--TRDIDL------QGVFI
THISH01973      ---GVTGMRIK-----------D-----VKS-------G-E--TKDIEL------MGVFI
METAA00813      ---GVTGIRIK-----------S-----TKS-------E-E--TKELDV------HGVFI
META200813      ---GVTGIRIK-----------S-----TKS-------E-E--TKELDV------HGVFI
METMM02895      ---GVTGIRIK-----------N-----SQD-------G-S--TKDIDV------HGVFI
FRAAD00813      ---GVTGVRVK-----------S-----TLD-------G-A--LREIEA------TGFFV
XYLFA01416      ---SVTGVRIR-----------S-----TQD-------S-S--TRDIDV------QGLFV
XYLFT00623      ---GVTGVRIR-----------S-----TQD-------S-S--TRDIDV------QGLFV
XYLF200667      ---GVTGVRIR-----------S-----TQD-------S-S--TRDIDV------QGLFV
XYLFG01622      ---GVTGVRIR-----------S-----TQD-------S-S--TRDIDV------QGLFV
XYLFM00690      ---GVTGVRIR-----------S-----TQD-------S-S--TRDIDV------QGLFV
PSEUP01680      ---GVTGLRVR-----------S-----VLD-------G-S--TRDLVV------HGFFV
STRM501972      ---GVTGVRVK-----------S-----TLD-------G-S--TRDIDA------HGFFV
PSEUU01524      ---GVTGVRVR-----------S-----TLD-------G-S--TRDIAA------HGFFV
XANAP01407      ---GVTGVRVK-----------S-----VID-------G-S--TRELQA------QGFFV
XANCP01918      ---GVTGVRVK-----------S-----TLD-------G-S--TRDIDA------HGFFV
XANC802154      ---GVTGVRVK-----------S-----TLD-------G-S--TRDIDA------HGFFV
XANCB02263      ---GVTGVRVK-----------S-----TLD-------G-S--TRDIDA------HGFFV
XANOR02379      ---GVTGVRVK-----------S-----TID-------G-S--TRDIDA------HGFFV
XANOM02295      ---GVTGVRVK-----------S-----TID-------G-S--TRDIDA------HGFFV
```

```
XANOP02318      ---GVTGVRVK-----------S-----TID-------G-S--TRDIDA------HGFFV
XANAC01951      ---GVTGVRVK-----------S-----TID-------G-S--TRDIDA------HGFFV
CYCSP01155      ---GVTGIRIK-----------N-----TQD-------N-S--TKEIDL------EGVFI
GEOLS03275      ---GVTGMRIK-----------D-----VRD-------G-S--TKEIKL------DGVFV
GEOUR03858      ---GVTGIRIR-----------H-----T-S-------G-S--TKDLQV------HGCFI
GEODF00692      ---GVTGIRIR-----------H-----T-S-------G-S--TKDLSV------HGCFI
GEOSL00482      ---GVTGVRIR-----------H-----T-S-------G-S--TKELDV------HGCFI
GEOSK00469      ---GVTGVRIR-----------H-----T-S-------G-S--TKELDV------HGCFI
GEOMG02998      ---GVTGIRIR-----------H-----T-S-------G-S--TKDIPV------HGCFI
MORCR00228      ---GVTGVMIE-----------S-----TQD-------G-S--IKQLDI------MGLFV
ACIAD00798      ---GVTSVRLK-----------S-----TQD-------E-S--TQTIDV------QGLFV
ACIBC00821      ---GVTGVRLK-----------S-----TKD-------D-S--KQEVQV------QGLFI
ACIBY02725      ---GVTGVRLK-----------S-----TKD-------D-S--KQEVQV------QGLFI
ACIB302691      ---GVTGVRLK-----------S-----TKD-------D-S--KQEVQV------QGLFI
ACIB500882      ---GVTGVRLK-----------S-----TKD-------D-S--KQEVQV------QGLFI
ACIB100850      ---GVTGVRLK-----------S-----TKD-------D-S--KQEVQV------QGLFI
ACIBD00843      ---GVTGVRLK-----------S-----TKD-------D-S--KQEVQV------QGLFI
ACIBS02229      ---GVTGVRLK-----------S-----TKD-------D-S--KQEVQV------QGLFI
ACICP00137      ---GVTSVRLK-----------S-----TQD-------D-S--KQDVEV------HGLFV
ACISD03052      ---GVTSVRLK-----------S-----TQD-------E-S--KQDVEV------HGLFV
LEGLN02402      ---KVTGALIR-----------N-----VQN-------D-S--KASLDV------DGVFI
LEGPA01726      ---KVTGAMIR-----------N-----LKT-------G-E--TDQLAM------DGIFI
LEGPH00846      ---KVTGAMIR-----------N-----LKT-------G-E--TDQLAM------DGIFI
LEGPC01171      ---KVTGAMIR-----------N-----LKT-------G-E--TDQLAM------DGIFI
LEGP201857      ---KVTGAMIR-----------N-----LKT-------G-E--TDQLAM------DGIFI
LEGPL01727      ---KVTGAMIR-----------N-----LKT-------G-E--TDQLAI------DGIFI
COLP302668      ---GVTGLRLK-----------E-----MGS-------D-A--TEELEV------SGVFI
KANKD01106      ---GVTGIRIK-----------S-----TQD-------D-S--IEELEL------QGVFI
IDILO00659      ---GVTGVRIK-----------D-----TQD-------G-S--TEELEV------AGCFI
PSEU901284      ---GVTGVRIK-----------D-----VAS-------D-A--TEELDL------AGVFI
PSEA602346      ---GVTGVRIK-----------S-----TTS-------D-E--SKELDV------MGLFV
ALTSS01871      ---GVTKIRIK-----------E-----TDG-------E-A--TEEIDV------MGLFV
ALTMD01611      ---GVTKIRIK-----------E-----TEG-------D-A--TEELDV------MGLFI
ALTME01705      ---GVTKVRIK-----------D-----TNS-------D-A--TEELDV------MGLFV
ALTMB01797      ---GVTKVRIK-----------D-----TNS-------D-A--TEELDV------MGLFV
ALTMS01652      ---GVTKIRIK-----------D-----TNS-------D-A--TEEIDV------MGLFV
SACD201683      ---GVTGARLK-----------S-----SID-------G-S--TQEIDA------SGVFI
TERTT01748      ---GVNGLRLK-----------S-----TQD-------G-S--TQEIDV------AGVFI
SIMAS00609      ---GVTGVRVK-----------S-----TQD-------G-S--TQDLDA------QGAFI
ALCDB01962      ---GVNGIRVK-----------S-----TKD-------D-S--TEELEL------QGLFI
CHRSD02939      ---GVTGVRLI-----------S-----TVD-------G-A--SREIQA------PGLFV
HALED02811      ---GVTGARLK-----------S-----TED-------G-S--TRELDA------PGVFI
SIDLE02655      ---GVTGMRIK-----------Q-----VQG-------S-A--TQEIKV------AGVFI
GALCS01621      ---GVTGMRVK-----------D-----VQS-------G-D--KQDIEL------TGVFI
LARHH01446      ---GVTGARLK-----------F-----KD--------D-H--AEEITV------AGVFI
CHRVO02807      ---GVTGVRVK-----------L-----N-D-------G-G--SENLAL------TGVFI
PSEUL01648      ---GVTGARLK-----------S-----TTD-------G-S--TRDIAL------TGVFI
NITEU01859      ---GVTGIRIR-----------H-----VRD-------D-T--ARDLEL------QGVFI
NITEC00745      ---GVTGVRIR-----------H-----VRD-------D-T--ARDLDL------QGVFI
NITMU00023      ---GVTGMRIK-----------N-----VLD-------D-S--TKTIDL------QGVFI
NITSI03064      ---GVTGMRIK-----------S-----TKD-------D-S--TQKIDL------QGVFI
ACCPU02128      ---GVTGMRIR-----------N-----KLT-------D-A--TEDLSL------MGVFI
THIDA01000      ---GVTGMRIK-----------S-----TKD-------G-A--TRDIAL------TGVFI
METS601221      ---GVTGMRIK-----------N-----NET-------G-A--TKDIPL------QGVFI
METGS01186      ---GVTGMRIK-----------N-----NET-------G-A--TKDIPL------QGVFI
METFK00972      ---GVTGIRLK-----------D-----VNS-------G-A--TKEIAL------KGVFI
METML01428      ---GVTGMRIK-----------N-----VND-------G-S--TKDIAL------QGVFI
AROAE03899      ---GVTGMRVR-----------D-----ANT-------G-A--TRDVAL------QGVFI
THASP01686      ---GVTGMRIK-----------G-----VNN-------G-A--TREVAL------QGVFI
AZOSB01359      ---GVTGMRVR-----------N-----E-S-------G-A--TQDIAL------QGVFI
BORA102682      ---GVTGVRIR-----------N-----VET-------G-A--TEDLAA------LGCFV
BORPA03415      ---GVTGVRIR-----------S-----TET-------D-A--TEDLDV------AGCFV
BORBM03577      ---GVTGVRIR-----------S-----TET-------D-A--TEDLDV------AGCFV
BORPE02280      ---GVTGVRIR-----------S-----TET-------D-A--TEDLDV------DGCFV
BORPC02060      ---GVTGVRIR-----------S-----TET-------D-A--TEDLDV------DGCFV
```

```
BORP102392      ---GVTGVRIR-----------S-----TET-------D-A--TEDLDV------DGCFV
BORBR03870      ---GVTGVRIR-----------S-----TET-------D-A--TEDLDV------AGCFV
BORPD01524      ---GVTGVRVR-----------H-----TDT-------G-A--TEDLPV------TGAFI
ACHXA01179      ---GVTGVRVR-----------H-----VDT-------G-A--TEDMTV------TGAFI
RHOFT03114      ---GVTGVRLK-----------N-----AST-------G-S--TEELRL------QGCFI
VEREI01701      ---GVTGVRIK-----------S-----TRD-------G-S--TQSIAL------QGCFI
VARPE01375      ---GVTGIRIK-----------N-----TQT-------G-E--TEQIDL------KGCFI
VARPS01266      ---GVTGIRIK-----------N-----TQS-------G-A--TEQIDL------KGCFI
DELAS05272      ---GVTGIRIK-----------S-----TQD-------G-H--AEDIQL------QGCFI
DELSC01235      ---GVTGIRIK-----------S-----TQD-------G-H--AEDIQL------QGCFI
COMT200883      ---GVTGIRIK-----------S-----TQD-------G-H--TEEVKL------QGAFI
ACIAC03267      ---GVTGIRIR-----------S-----TQD-------G-S--TQDIAL------QGCFI
ACIAP03249      ---GVTGIRIK-----------S-----TLD-------G-S--TQDIAL------QGCFI
ACIET02648      ---GVTGIRLK-----------H-----TED-------G-H--TEDIAL------QGCFI
ALIDK03528      ---GVTGIRIK-----------H-----TGD-------G-H--TEDIAL------QGCFI
RUBGI03408      ---GVTGVRLK-----------S-----VND-------G-T--TEELKV------NGCFI
LEPCP00749      ---GVTGVRLK-----------S-----TTD-------D-S--TTELAV------KGCFI
POLSJ03719      ---GVTGVRLK-----------S-----TLH-------D-T--AQDLAL------KGCFI
POLNA03144      ---GVTGVRLR-----------S-----TKT-------D-A--TQDITL------KGCFI
METPP01110      ---GVTGVRLK-----------N-----TQT-------G-E--TQDLML------KGCFI
RAMTT03218      ---GVTGIRLK-----------D-----ANT-------G-A--TEDLQL------KGCFI
RALPJ02457      ---GVTGARLK-----------G-----THENE-----G-K--TEEIKL------AGVFI
RALP102102      ---GVTGARLK-----------G-----THENE-----G-K--TEEIKV------AGVFI
RALSO02303      ---GVTGVRLR-----------G-----VNGNA-----T-G--AEEVRL------AGVFI
RALS801097      ---GVTGVRLR-----------G-----VNGNHIGGNPD-G--TEELKL------AGVFI
HERSS01878      ---GVTGIKIK-----------S-----TQD-------D-A--ITEIPV------HGLFI
HERAR00955      ---GVTGINIR-----------S-----TTD-------D-S--ITAIKL------HGVFI
JANMA01152      ---GVTGMTIK-----------S-----TAD-------G-S--ITPIKL------HGVFV
THIK102324      ---GVTGVRIH-----------N-----SQT-------G-A--DEDIAL------NGCFI
POLSQ00682      ---GVTGVRIK-----------K-----E-D-------G-S--TEDIAV------TGAFI
POLNS00989      ---GVTGVRIK-----------K-----Q-D-------G-S--TEDIVV------TGAFI
BURP800627      ---GVNGLRIK-----------N-----VKT-------G-A--TTDIEL------QGLFV
BURPP00988      ---GVTGLRIK-----------N-----TKT-------G-A--TEDIAL------QGIFV
BURSC00728      ---GVTGLRIK-----------H-----TQT-------G-E--TTDIAL------QGLFV
BURXL00982      ---GVTGLRIK-----------H-----TQT-------G-E--TTDLAL------QGLFV
BURSG00869      ---GVTGVRIK-----------H-----TQT-------G-E--TTDIAL------QGLFV
BURRH00629      ---GVTGLRIK-----------H-----TKT-------G-E--SADLAV------HGLFV
BURGB00781      ---GVTGVRIK-----------H-----TGT-------G-E--TEQIAL------QGVFV
BURGS00853      ---GVTGVTIK-----------H-----TGT-------G-E--TEQLTL------QGVFV
BURPS02618      ---GVSGVRIK-----------H-----VTT-------G-A--TEDVAV------QGLFI
BURMA01741      ---GVSGVRIK-----------H-----VTT-------G-A--TEDVAV------QGLFI
BURP103016      ---GVSGVRIK-----------H-----VTT-------G-A--TEDVAV------QGLFI
BURP002984      ---GVSGVRIK-----------H-----VTT-------G-A--TEDVAV------QGLFI
BURM701866      ---GVSGVRIK-----------H-----VTT-------G-A--TEDVAV------QGLFI
BURP602945      ---GVSGVRIK-----------H-----VTT-------G-A--TEDVAV------QGLFI
BURM902465      ---GVSGVRIK-----------H-----VTT-------G-A--TEDVAV------QGLFI
BURMS00740      ---GVSGVRIK-----------H-----VTT-------G-A--TEDVAV------QGLFI
BURTA01515      ---GVSGVRIK-----------H-----VTT-------G-A--TEDVAV------QGLFI
BURM102386      ---GVTGVRIK-----------H-----VKT-------G-A--TEDIAV------QGLFV
BURL300890      ---GVTGLRIK-----------N-----VKT-------G-A--TEDLAV------QGVFV
BURVG00860      ---GVTGLRIK-----------N-----VKT-------G-A--TQDLTV------QGVFV
BURCM00829      ---GVTGLRIK-----------N-----VKT-------G-A--TQDLNV------QGVFV
BURA400832      ---GVTGLRIK-----------N-----VKT-------G-A--TQDLHV------QGVFV
BURCA00489      ---GVTGLRIK-----------N-----VKT-------G-A--TEDLLV------QGVFV
BURCH00966      ---GVTGLRIK-----------N-----VKT-------G-A--TEDLLV------QGVFV
BURCC00927      ---GVTGLRIK-----------N-----VKT-------G-A--TEDLLV------QGVFV
BURCJ02922      ---GVTGLRIK-----------N-----VKT-------G-A--TEDLQV------QGVFV
EDWI902348      ---GVNALRLK-----------D-----TQNG----------ACEALAV------SGLFV
EDWTF01971      ---GVNALRLK-----------D-----TQSG----------ACEELAV------SGLFV
EDWTE02176      ---GVNALRLK-----------D-----TQSG----------ACEELAV------SGLFV
SODGM01094      ---GVTGVRLK-----------E-----VAGS----------AHEDLAV------AGVFI
MOREP00394      ---GVTGVRLK-----------D-----VLSN----------ACEELAV------NGVFI
RAHSY01432      ---GVTGASLL-----------D-----VRTD----------EKSQVDV------AGVFI
RAHAC01419      ---GVTGASLM-----------D-----VRTD----------EKSQVDV------AGVFI
ERWBE01489      ---GVSSLRLR-----------S-----ALDD---------QQTEELNV------AGLFV
```

```
PANAM01339       ---GVTGLTLR-----------S-----TLDD----------KTESLEV------AGLFV
PANAA00662       ---GVTGLTLR-----------S-----TLDD----------KTESLEV------AGLFV
PANSA01303       ---GVTGLKLL-----------S-----TKGE----------AAESLEV------AGLFV
ERWT902154       ---GVSALRLR-----------S-----TKDD----------RSKDLAV------AGLFV
ERWAC01330       ---GVSALRLR-----------S-----TKDQ----------QTKDLAV------AGLFV
ERWAE01326       ---GVSALRLR-----------S-----TKDQ----------QTKDLAV------AGLFV
ERWPE02241       ---GVSALRLR-----------S-----TKDD----------QTKELAV------AGLFV
ERWP602414       ---GVSALRLR-----------S-----TKDD----------QTKELAV------AGLFV
ERWSE02396       ---GVSALRLR-----------S-----TKDD----------QTEELAV------AGLFV
PECCP01694       ---GVTGVRIR-----------D-----TQSD----------AAEELEL------AGVFI
PECWW01889       ---GVTGVRIR-----------D-----TQSD----------AAEELEL------AGVFI
PECSS01870       ---GVTGVRIR-----------D-----TQSD----------AAEELEL------AGVFI
PECAS02624       ---GVTGVRIR-----------D-----TQSD----------AAEELEL------AGVFI
DICDC02220       ---GVTGVRLR-----------N-----TQSD----------ATEQLDI------AGVFI
DICZE02284       ---GVTGVRLR-----------E-----AAGD----------ATEQLDV------AGVFI
DICD302017       ---GVTGVRLR-----------D-----AAGH----------ATEQLDV------AGVFI
DICD502250       ---GVTGVRLR-----------E-----AAGE----------AVEQLDV------AGVFI
XENBS00829       ---GVTGIRIH-----------D-----TKSD----------NAEELDV------TGVFI
XENNA01480       ---GVTGVRIR-----------D-----TKSD----------NTEEVDV------AGVFI
PHOLL01537       ---GVTGVRLR-----------D-----TKSG----------ATEELAV------TGTFI
PHOAA02804       ---GVTGVRLR-----------D-----TKSD----------TTEELAV------TGAFI
SERP501669       ---GVTGVRIR-----------S-----TKAE---------DQTQELEL------AGVFI
SERSA01625       ---GVTGVRIR-----------S-----TKAD---------NATQELAL------AGVFI
YERPE01271       ---GVTGVRLK-----------S-----THSD----------ETEELAV------AGVFI
YERPS01390       ---GVTGVRLK-----------S-----THSD----------ETEELAV------AGVFI
YERPA00627       ---GVTGVRLK-----------S-----THSD----------ETEELAV------AGVFI
YERPN02489       ---GVTGVRLK-----------S-----THSD----------ETEELAV------AGVFI
YERPP02190       ---GVTGVRLK-----------S-----THSD----------ETEELAV------AGVFI
YERP302544       ---GVTGVRLK-----------S-----THSD----------ETEELAV------AGVFI
YERPB01460       ---GVTGVRLK-----------S-----THSD----------ETEELAV------AGVFI
YERPY02633       ---GVTGVRLK-----------S-----THSD----------ETEELAV------AGVFI
YERPG01392       ---GVTGVRLK-----------S-----THSD----------ETEELAV------AGVFI
YERPD01171       ---GVTGVRLK-----------S-----THSD----------ETEELAV------AGVFI
YERP100890       ---GVTGVRLK-----------S-----THSD----------ETEELAV------AGVFI
YERPZ01206       ---GVTGVRLK-----------S-----THSD----------ETEELAV------AGVFI
YERPH02469       ---GVTGVRLK-----------S-----THSD----------ETEELAV------AGVFI
YERE801438       ---GVTGVRLK-----------S-----TKNNE-----T-EEETEELAV------AGVFI
YERE302565       ---GVTGVRLK-----------S-----TKNNE-----T-----EELAV------AGVFI
YERE100392       ---GVTGVRLK-----------S-----TKNNE-----T-----EELAV------AGVFI
PROMH00687       ---GVTKVRLK-----------D-----TKSD----------KTEELEV------MGVFI
PROSM03196       ---GVTGVRLR-----------N-----TKTD----------ETEELEV------MGAFI
TOLAT02279       ---GVTGVRLR-----------N-----LKDD----------SVEDVEV------MGAFI
AERVB02262       ---GVTGVRLR-----------N-----TQDN----------STSELPL------MGVFI
AERHH01811       ---GVTGVRLR-----------N-----TQND----------TTTDLPL------MGVFI
AERS402185       ---GVTGVRLR-----------S-----TQDD----------TTTDLPL------MGVFI
PSYIN02070       ---GVTGLRLK-----------D-----TLSD----------KTENLEV------MGVFI
SHELP02018       ---GVTGVKIK-----------S-----TKDE----------SITEFDV------MGVFV
SHEVD02297       ---GVTGLKMK-----------S-----TKDG----------AISELEL------MGVFI
SHEPW02341       ---GVNGLKMK-----------S-----TKDG----------SIKDLEV------AGVFV
SHEPA02202       ---GVTGLKMK-----------S-----TKDG----------SIKDLEV------AGVFV
SHEHH02007       ---GVTGLKMK-----------S-----TKDD----------SITDLEV------AGVFV
SHESH02117       ---GVTGLKMK-----------S-----TKDG----------SITDLEV------MGVFV
SHEWM02454       ---GVTGLKMK-----------S-----TKDG----------AIKDLEV------MGVFV
SHEAM01758       ---GVTGVKLK-----------S-----TKDD----------STEAFDV------AGVFV
SHEON02159       ---GVTGLKMK-----------S-----TKDG----------AITDLAV------AGVFV
SHESM01945       ---GVTGLKMK-----------S-----TKDG----------SITDLAV------AGVFV
SHESR01991       ---GVTGLKMK-----------S-----TKDG----------AITDLAV------AGVFV
SHESA02025       ---GVTGLKMK-----------S-----TKDG----------AITDLAV------AGVFV
SHESW01953       ---GVTGLKMK-----------S-----TKDD----------SITDLAV------AGVFV
SHEPC01976       ---GVTGLKMK-----------S-----TKDD----------SITDLAV------AGVFV
SHEP201929       ---GVTGLKMK-----------S-----TKDD----------SITDLAV------AGVFV
SHEB502016       ---GVNGLKMK-----------S-----TKDG----------SITDLAV------AGVFV
SHEB802168       ---GVNGLKMK-----------S-----TKDG----------SITDLAV------AGVFV
SHEB202117       ---GVNGLKMK-----------S-----TKDG----------SITDLAV------AGVFV
SHEB902270       ---GVNGLKMK-----------S-----TKDG----------SITDLAV------AGVFV
```

```
SHEB602258       ---GVNGLKMK-----------S-----TKDG----------SITDLAV------AGVFV
SHEDO01680       ---GVTGLRKK-----------S-----TKDG----------VVTDLAV------AGVFI
SHEFN01895       ---GVTGLKKK-----------S-----TKDG----------SVTDLEV------AGVFV
PSEHT01677       ---GVTGVRIK-----------D-----ANSD----------ATEELDL------AGVFI
ALISL01611       ---GVTGVRLK-----------D-----TKSD----------MTEDLDV------MGAFI
VIBF100893       ---GVTGVRLK-----------D-----TKSD----------ATEDLEV------MGAFI
VIBFM00891       ---GVTGVRLK-----------D-----TKSD----------ATENLEV------MGAFI
OCESG01399       ---GVTGVRLK-----------H-----TQSD----------ATEQLDV------AGVFI
VIBA701872       ---GVTGVRMK-----------H-----TQSE----------TTEDLDV------MGVFI
VIBVY01451       ---GVTGVRLK-----------D-----VKTG----------GTEELDV------MGAFI
VIBVU02545       ---GVTGVRLK-----------D-----VKTG----------GTEELDV------MGAFI
VIBVM01748       ---GVTGVRLK-----------D-----VKTG----------GTEELDV------MGAFI
VIBCH01157       ---GVTGVRLK-----------D-----TQSD----------MTENLDV------MGVFI
VIBCM01114       ---GVTGVRLK-----------D-----TQSD----------MTENLDV------MGVFI
VIBCJ02064       ---GVTGVRLK-----------D-----TQSD----------MTENLDV------MGVFI
VIBC300725       ---GVTGVRLK-----------D-----TQSD----------MTENLDV------MGVFI
FERBD01969       ---GVTGARIK-----------D-----TRSE----------ATEELKV------DGVFI
VIBFN02085       ---GVTGVRLK-----------D-----TQSG----------ETEEFDV------MGVFI
VIBTL01089       ---GVTGVRIK-----------D-----TQSD----------KTEDIEV------MGAFI
VIBPA01248       ---GVTGVRIK-----------D-----VNTG----------TTEDLEV------MGAFI
VIBAE02128       ---GVTGVRIK-----------D-----VNTG----------ATEDLEV------MGAFI
VIBCB00599       ---GVTGVRIK-----------D-----VNSG----------ATEDLAV------MGAFI
PANVC00701       ---GVTGLTLR-----------S-----TQNS---------DETEALEV------AGLFV
SHIBC02443       ---GVSGLRLR-----------D-----TQDT---------SNTENLEV------TGLFV
ENTBF02792       ---GVSGLRLR-----------D-----TQNS---------DNIETLDV------AGLFV
KLEP700892       ---GVSGLRLR-----------D-----TKNS---------DNVESLEV------AGLFV
KLEPH01782       ---GVSGLRLR-----------D-----TKNS---------DNVESLEV------AGLFV
KLEP303551       ---GVSGLRLR-----------D-----TKNS---------DNVESLEV------AGLFV
KLEVT03420       ---GVSGLRLR-----------D-----TKNS---------DNVESLEV------AGLFV
ENTAK02985       ---GVSGLRLR-----------D-----TKNS---------DNVETLDV------AGLFV
KLEOK03151       ---GVSGLRLR-----------D-----TQNA---------DNVESLEV------AGLFV
SALAR01925       ---GVTGLRLR-----------D-----TQQS---------DNIETLDI------AGLFV
SALBC00812       ---GVTGLRLR-----------D-----TQQR---------DNIETLDV------AGLFV
SALPC00926       ---GVTGLRLR-----------D-----TQQS---------DNIETLDI------AGLFV
SALTI00868       ---GVTGLRLR-----------D-----TQQS---------DNIETLDI------AGLFV
SALCH00905       ---GVTGLRLR-----------D-----TQQS---------DNIETLDI------AGLFV
SALPA01706       ---GVTGLRLR-----------D-----TQQS---------DNIETLDI------AGLFV
SALTY00927       ---GVTGLRLR-----------D-----TQQS---------DNIETLDI------AGLFV
SALPK01786       ---GVTGLRLR-----------D-----TQQS---------DNIETLDI------AGLFV
SALHS00978       ---GVTGLRLR-----------D-----TQQS---------DNIETLDI------AGLFV
SALEP00854       ---GVTGLRLR-----------D-----TQQS---------DNIETLDI------AGLFV
SALDC00926       ---GVTGLRLR-----------D-----TQQS---------DNIETLDI------AGLFV
SALA400892       ---GVTGLRLR-----------D-----TQQS---------DNIETLDI------AGLFV
SALG200876       ---GVTGLRLR-----------D-----TQQS---------DNIETLDI------AGLFV
SALTS00888       ---GVTGLRLR-----------D-----TQQS---------DNIETLDI------AGLFV
SALT400905       ---GVTGLRLR-----------D-----TQQS---------DNIETLDI------AGLFV
SALPS01908       ---GVTGLRLR-----------D-----TQQS---------DNIETLDI------AGLFV
SALT101040       ---GVTGLRLR-----------D-----TQQS---------DNIETLDI------AGLFV
SALTD00960       ---GVTGLRLR-----------D-----TQQS---------DNIETLDI------AGLFV
SALPB02472       ---GVTGLRLR-----------D-----TQQS---------DNIETLDI------AGLFV
SALNS00928       ---GVTGLRLR-----------D-----TQQS---------DNIETLDI------AGLFV
SALSV01000       ---GVTGLRLR-----------D-----TQQS---------DNIETLDI------AGLFV
ECOS500812       ---GVTGVRLR-----------D-----TQNT---------DNIESLDV------AGLFV
ECOL600981       ---GVTGVRLR-----------D-----TQNS---------DNIESLDV------AGLFV
ECOL500883       ---GVTGVRLR-----------D-----TQNS---------DNIESLDV------AGLFV
ECOUT00876       ---GVTGVRLR-----------D-----TQNS---------DNIESLDV------AGLFV
ECOK100776       ---GVTGVRLR-----------D-----TQNS---------DNIESLDV------AGLFV
ECOSM02148       ---GVTGVRLR-----------D-----TQNS---------DNIESLDV------AGLFV
ECOLU01057       ---GVTGVRLR-----------D-----TQNS---------DNIESLDV------AGLFV
ECO7I02136       ---GVTGVRLR-----------D-----TQNS---------DNIESLDV------AGLFV
ECO8100828       ---GVTGVRLR-----------D-----TQNS---------DNIESLDV------AGLFV
ECO4500862       ---GVTGVRLR-----------D-----TQNS---------DNIESLDV------AGLFV
ECOAB00879       ---GVTGVRLR-----------D-----TQNS---------DNIESLDV------AGLFV
ECO4400961       ---GVTGVRLR-----------D-----TQNS---------DNIESLDV------AGLFV
ECOUM02550       ---GVTGVRLR-----------D-----TQNS---------DNIESLDV------AGLFV
```

```
ECOKI00869      ---GVTGVRLR-----------D-----TQNS---------DNIESLDV------AGLFV
ECOC100920      ---GVTGVRLR-----------D-----TQNS---------DNIESLDV------AGLFV
ECOC200920      ---GVTGVRLR-----------D-----TQNS---------DNIESLDV------AGLFV
ECO8N00803      ---GVTGVRLR-----------D-----TQNS---------DNIESLDV------AGLFV
ECO2700862      ---GVTGVRLR-----------D-----TQNS---------DNIESLDV------AGLFV
ECO2600974      ---GVTGVRLR-----------D-----TQNS---------DNIESLDV------AGLFV
ECOH100942      ---GVTGVRLR-----------D-----TQNS---------DNIESLDV------AGLFV
SHIB301848      ---GVTGVRLR-----------D-----TQNS---------DNIESLDV------AGLFV
ECOLI00846      ---GVTGVRLR-----------D-----TQNS---------DNIESLDV------AGLFV
ECO5700970      ---GVTGVRLR-----------D-----TQNS---------DNIESLDV------AGLFV
SHISS00756      ---GVTGVRLR-----------D-----TQNS---------DNIESLDV------AGLFV
SHIBS00679      ---GVTGVRLR-----------D-----TQNS---------DNIESLDV------AGLFV
SHIDS01857      ---GVTGVRLR-----------D-----TQNS---------DNIESLDV------AGLFV
ECO2400906      ---GVTGVRLR-----------D-----TQNS---------DNIESLDV------AGLFV
ECODH00784      ---GVTGVRLR-----------D-----TQNS---------DNIESLDV------AGLFV
ECOHS00922      ---GVTGVRLR-----------D-----TQNS---------DNIESLDV------AGLFV
ECOLC02627      ---GVTGVRLR-----------D-----TQNS---------DNIESLDV------AGLFV
ECO5E00946      ---GVTGVRLR-----------D-----TQNS---------DNIESLDV------AGLFV
ECOSE00936      ---GVTGVRLR-----------D-----TQNS---------DNIESLDV------AGLFV
ECO5500894      ---GVTGVRLR-----------D-----TQNS---------DNIESLDV------AGLFV
ECO8A00877      ---GVTGVRLR-----------D-----TQNS---------DNIESLDV------AGLFV
ECOBB00863      ---GVTGVRLR-----------D-----TQNS---------DNIESLDV------AGLFV
ECO5T00943      ---GVTGVRLR-----------D-----TQNS---------DNIESLDV------AGLFV
ECOBW00723      ---GVTGVRLR-----------D-----TQNS---------DNIESLDV------AGLFV
ECO1000919      ---GVTGVRLR-----------D-----TQNS---------DNIESLDV------AGLFV
ECOBD02604      ---GVTGVRLR-----------D-----TQNS---------DNIESLDV------AGLFV
ECOD102659      ---GVTGVRLR-----------D-----TQNS---------DNIESLDV------AGLFV
ECOBR00856      ---GVTGVRLR-----------D-----TQNS---------DNIESLDV------AGLFV
ECOLX02610      ---GVTGVRLR-----------D-----TQNS---------DNIESLDV------AGLFV
ECO1A00939      ---GVTGVRLR-----------D-----TQNS---------DNIESLDV------AGLFV
ECOCB01054      ---GVTGVRLR-----------D-----TQNS---------DNIESLDV------AGLFV
ECOKO02859      ---GVTGVRLR-----------D-----TQNS---------DNIESLDV------AGLFV
ECO1E03320      ---GVTGVRLR-----------D-----TQNS---------DNIESLDV------AGLFV
ECOLW01217      ---GVTGVRLR-----------D-----TQNS---------DNIESLDV------AGLFV
SHIFL01399      ---GVTGVRLR-----------D-----TQNS---------DNIESLDV------AGLFV
SHIF800759      ---GVTGVRLR-----------D-----TQNS---------DNIESLDV------AGLFV
SHIF200805      ---GVTGVRLR-----------D-----TQNS---------DNIESLDV------AGLFV
CITK802118      ---GVTGLRLR-----------D-----TQNP---------DNIESLDV------AGLFV
CITRI00924      ---GVTGLRLR-----------D-----TQNA---------DNIEALDV------AGLFV
CROS802385      ---GVSGLRLR-----------D-----TVNP---------ENVETLDV------AGLFV
CROTZ01500      ---GVSGLRLR-----------D-----TVNP---------ENVETLDV------AGLFV
ENTLS02865      ---GVSGLRIR-----------D-----TQNS---------DIVESLEV------AGLFV
ENT3801400      ---GVSGLRLR-----------D-----TQNS---------DNVESLEV------AGLFV
ENTAL01384      ---GVAGLRIR-----------D-----TQNT---------DNVESLEV------AGLFV
ENTCC02679      ---GVAGLRIR-----------D-----TQNT---------DNVETLEV------AGLFV
                                                                          *

STRT101547      YVGLDPVSDYLTGL------DITDQNGWVITD----DK-------------------MAT
STRT201508      YVGLDPVSDYLTGL------DITDQNGWVITD----DK-------------------MAT
STRTD01356      YVGLDPVSDYLTGL------DITDQNGWVITD----DK-------------------MAT
STRTN01533      YVGLDPVSDYLTGL------DITDQNGWVITD----DK-------------------MAT
STRE500345      YVGLDPVSDYLTGL------DVTDQDGWVITD----DK-------------------MAT
STRE801625      YVGLDPVSDYLTGL------DITDQDGWVITD----DK-------------------MAT
STREH01636      YVGLDPVSDYLTGL------DITDQDGWVITD----DK-------------------MAT
STREC01656      YVGLIPVTQMVSEL------GITNQEGWIVTD----DQ-------------------MKT
STREM01495      YVGLIPVTQMVSEL------GITNQEGWILTD----DQ-------------------MKT
STRE401629      YVGLIPVTQMVSEL------GITNQEGWIVTD----DQ-------------------MKT
STRS700387      YVGLIPVTQMVSEL------GITNQEGWIVTD----DQ-------------------MKT
STRDG01631      YVGMIPVTGMVSEL------GITDSEGWIVTD----DH-------------------MRT
STRP301388      YVGMNPVTDMVKDL------EITDQEGWIITD----DH-------------------MRT
STRPZ01205      YVGMNPVTDMVKDL------EITDQEGWIITD----DH-------------------MRT
STRPQ00464      YVGMNPVTDMVKDL------EITDQEGWIITD----DH-------------------MRT
STRPD01453      YVGMNPVTDMVKDL------EITDQEGWIITD----DH-------------------MRT
STRP601399      YVGMIPVTGMVKDL------KITDSEGWIITD----DH-------------------MRT
STRP801366      YVGMNPVTGMVKDL------EITDSEGWIITD----DH-------------------MRT
```

```
STRPF01453      YVGMNPVTGMVKDL------EITDSEGWIITD----DH--------------------MRT
STRPG00427      YVGMNPVTGMVKDL------EITDSEGWIITD----DH--------------------MRT
STRA300284      YVGLKPHSSMVSEL------GITDETGWVLTD----TN--------------------MKT
STRA500290      YVGLKPHSSMVSEL------GITDETGWVLTD----TN--------------------MKT
STRA100288      YVGLKPHSSMVSEL------GITDETGWVLTD----TN--------------------MKT
STRA200291      YVGLKPHSSMVSEL------GITDETGWVLTD----TN--------------------MKT
STRIC00348      YVGVDPVSKMVEGL------GITDEAGWVITD----DH--------------------MKT
STRPX00375      YVGVNPVTSMVADL------GITDEAGWVITD----ER--------------------MMT
STRMD00432      YVGVNPVTSMVADL------GITDEAGWVITD----ER--------------------MMT
STRS201734      YVGLDPMTDTVADL------GITDEAGWVITN----EK--------------------MET
STRSY01721      YVGLDPMTDTVADL------GITDEAGWVITN----EK--------------------MET
STRSX01553      YVGLDPMTDTVADL------GITDEAGWVITN----EK--------------------MET
STRSE01478      YVGLDPMTDTVADL------GITDEAGWVITN----EK--------------------MET
STREJ01635      YVGLDPMTDTVADL------GITDEAGWVITN----EK--------------------MET
STRGZ01544      YVGLDPMTDTVADL------GITDEAGWVITN----EK--------------------MET
STRS401593      YVGLDPMTDTVADL------GITDEAGWVITN----EK--------------------MET
LACGT00720      YVGLDAVSEFARDL------GITDEEGWIITD----AT--------------------MKT
LACGL00738      YVGLDAVSEFARDL------GITDEEGWIITD----AT--------------------MKT
STRSV01774      YVGLDPVSEFAADL------GITDEAGWILTD----HQ--------------------MKT
STRIJ00350      YVGLDPVSEFVKDL------GITNESGWIVTD----HH--------------------MKT
STROU01248      YVGLDPVSDFVKDL------NIQDQSGWIVTD----NH--------------------MKT
STRM601286      YVGLDPLSDFVKEL------NIQDQAGWIVTD----NH--------------------MKT
STRES00962      YVGLDPLSDFVKEL------NIQDQAGWIVTD----NH--------------------MKT
STRP701387      YVGLDPLSDFVKEL------NIQDQAGWIVTD----NH--------------------MKT
STRZT00756      YVGLDPLSDFVKEL------NIQDQAGWIVTD----NH--------------------MKT
STRP001079      YVGLDPLSDFVKEL------NIQDQAGWIVTD----NH--------------------MKT
STRZO01270      YVGLDPLSDFVKEL------NIQDQAGWIVTD----NH--------------------MKT
STRZ600815      YVGLDPLSDFVKEL------NIQDQAGWIVTD----NH--------------------MKT
STRET00826      YVGLDPLSDFVKEL------NIQDQAGWIVTD----NH--------------------MKT
STRPS01429      YVGLDPLSDFVKEL------NIQDQAGWIVTD----NH--------------------MKT
STRZN01287      YVGLDPLSDFVKEL------NIQDQAGWIVTD----NH--------------------MKT
STRR601306      YVGLDPLSDFVKEL------NIQDQAGWIVTD----SH--------------------MKT
STRP201217      YVGLDPLSDFVKEL------NIQDQAGWIVTD----SH--------------------MKT
STRZP01342      YVGLDPLSDFVKEL------NIQDQAGWIVTD----SH--------------------MKT
STRZI01226      YVGLDPLSDFVKEL------NIQDQAGWIVTD----SH--------------------MKT
STRPN01360      YVGLDPLSDFVKEL------NIQDQAGWIVTD----NH--------------------MKT
STRP401357      YVGLDPLSDFVKEL------NIQDQAGWIVTD----NH--------------------MKT
STRZJ01282      YVGLDPLSDFVKEL------NIQDQAGWIVTD----NH--------------------MKT
STRPJ01336      YVGLDPLSDFVKEL------NIQDQAGWIVTD----NH--------------------MKT
STRPI01436      YVGLDPLSDFVKEL------NIQDQAGWIVTD----SH--------------------MKT
MARHT00044      FIGHEPNTAYLKGV------LELRPDGYIKVT----DE--------------------VYT
THEP300413      AIGYAPNTELVKGI------VDLDEYGYIMTD----DD--------------------MRT
THEPX00884      AIGYAPNTELVKGI------VDLDEYGYIMTD----DD--------------------MRT
THESX01857      AIGYAPNTELVKGI------VDLDEYGYIMTD----DD--------------------MRT
THEM301788      AIGYAPNTELVKGI------VDLDNYGYIMTD----DD--------------------MRT
THEIA01812      AIGYAPNTELVKGI------VDLDNYGYIMTD----DD--------------------MRT
THETC00443      AIGLSPNSDLVRGI------VDTDEYGYILTD----ED--------------------MKT
THESW01026      AIGLSPNSELVKGI------VDTDEYGYIITD----ED--------------------MKT
THEXL00360      AIGLSPNSELVKGI------VDTDEYGYIITD----ED--------------------MKT
THEID01127      FIGITPNSDFVKDL------LTLDEKGFIITD----SE--------------------MRT
THEOJ00147      AIGWDPNTAIVKDL------VQLNERGYIVTD----EN--------------------MAT
DESAS01237      YVGTRPSSELVKDL------IELDSRGYIMTD----ED--------------------MKT
DESK701373      YIGYNPNSYIVKEL------VKLDERGYILTD----AN--------------------MQT
KYRT200729      YIGLKPNTEFLKGT------PIVNAEGWIPTD----DR--------------------FRT
STACT00410      YIGMKPLTAPFIDL------GITNDAGYIVTN----DE--------------------MET
STAS101952      YIGMKPLTVPFQDL------GITNEVGYVLTN----ED--------------------MST
STALH01969      YIGMKPLTAPFLDL------GITNETGYIVTK----DD--------------------MST
STAEQ00422      YIGMKPLTAPFKNL------GITNDAGYIVTQ----DD--------------------MST
STAES00543      YIGMKPLTAPFKNL------GITNDAGYIVTQ----DD--------------------MST
STAAB00714      YIGMKPLTAPFKDL------GITNDVGYIVTK----DD--------------------MTT
STAA500765      YIGMKPLTAPFKDL------GITNDVGYIETK----DD--------------------MTT
STAAM00757      YIGMKPLTAPFKDL------GITNDVGYIVTK----DD--------------------MTT
STAAW00726      YIGMKPLTAPFKDL------GITNDVGYIVTK----DD--------------------MTT
STAAS00733      YIGMKPLTAPFKDL------GITNDVGYIVTK----DD--------------------MTT
STAAN00727      YIGMKPLTAPFKDL------GITNDVGYIVTK----DD--------------------MTT
```

```
STAAC00807    YIGMKPLTAPFKDL------GITNDVGYIVTK----DD-------------------MTT
STAA300727    YIGMKPLTAPFKDL------GITNDVGYIVTK----DD-------------------MTT
STAA800734    YIGMKPLTAPFKDL------GITNDVGYIVTK----DD-------------------MTT
STAA100752    YIGMKPLTAPFKDL------GITNDVGYIVTK----DD-------------------MTT
STAA200774    YIGMKPLTAPFKDL------GITNDVGYIVTK----DD-------------------MTT
STAA900759    YIGMKPLTAPFKDL------GITNDVGYIVTK----DD-------------------MTT
STAAE00716    YIGMKPLTAPFKDL------GITNDVGYIVTK----DD-------------------MTT
STAAT00767    YIGMKPLTAPFKDL------GITNDVGYIVTK----DD-------------------MTT
STAAD00695    YIGMKPLTAPFKDL------GITNDVGYIVTK----DD-------------------MTT
STAA000815    YIGMKPLTAPFKDL------GITNDVGYIVTK----DD-------------------MTT
STAAH02407    YIGMKPLTAPFKDL------GITNDVGYIVTK----DD-------------------MTT
STAAF00763    YIGMKPLTAPFKDL------GITNDVGYIVTK----DD-------------------MTT
STAAK00744    YIGMKPLTAPFKDL------GITNDVGYIVTK----DD-------------------MTT
STAAJ00703    YIGMKPLTAPFKDL------GITNDVGYIVTK----DD-------------------MTT
STAAG00690    YIGMKPLTAPFKDL------GITNDVGYIVTK----DD-------------------MTT
STAA400732    YIGMKPLTAPFKDL------GITNDVGYIVTK----DD-------------------MTT
STAAR00788    YIGMKPLTAPFKDL------GITNDVGYIVTK----DD-------------------MTT
LISSS02378    YVGLVPLTKAFLNL------GITDDEGYIITD----EE-------------------MRT
LISIN02590    YVGLVPLTKAFLSL------GITDEEGYIVTD----EE-------------------MRT
LISW602421    YVGLVPLTKAFLNL------GITDDEGYIVTD----EE-------------------MRT
EXISA00870    YIGMNPITGFVQDL------GITNEQGYIVTN----EA-------------------MET
EXIS202358    YIGMNPITGYVQDL------GILNDQGYVVTN----EA-------------------MET
EXIAB02180    YIGMNPITGYVQDL------GILNDQGYVVTN----EA-------------------MET
OCEIH02462    YIGMVPLNQAFLSL------GITNDEGYIATN----EN-------------------MET
BACIE01058    YIGMDPINEPFLNL------GITNEDGYVVTN----EN-------------------MET
BACCJ03468    YIGMLPINEPFLNL------GITNADGYVETN----DE-------------------MET
BACHD03507    YIGMLPLNEAVKNL------NILNDEGYIVTN----EE-------------------MET
BACPE03382    YIGMLPLNASVKNL------GILNEEGYVVTN----EE-------------------MET
SOLSS00743    YVGMLPLTAPFASL------NILNEAGYIVTN----EK-------------------MET
BACC600715    YIGMVPLTKPFENL------GITNEAGYIVTN----EE-------------------MET
ANOFW02486    YIGMVPLTKPFASL------GITNENGYIETN----EL-------------------MET
GEOKA03042    YIGMVPLSKPFANL------GITNENGYIVTN----EK-------------------MET
GEOSY02976    YIGMVPLSKPFANL------GITNENGYIVTN----EK-------------------MET
GEOTN02952    YIGMVPLSKPFANL------GITNENGYIVTN----EK-------------------MET
GEOSW02588    YIGMLPLSKPFESL------GITNENGYIETN----EL-------------------MET
GEOS000381    YIGMLPLSKPFVNL------GITNENGYIVTN----ER-------------------MET
GEOTC00378    YIGMLPLSKPFVNL------GITNENGYIVTN----ER-------------------MET
BACMD04906    YVGMVPLTKPFESL------NITNKEGYIETN----EQ-------------------MET
BACMQ04908    YVGMVPLTKPFESL------NITNKEGYIETN----EQ-------------------MET
BACWK04830    YIGMLPLSKPFVEL------GITNENGYVETN----ER-------------------MET
BACAN04889    YIGMLPLSKPFVEL------GITNENGYLETN----ER-------------------MET
BACC105210    YIGMLPLSKPFVEL------GITNENGYLETN----ER-------------------MET
BACC705051    YIGMLPLSKPFVEL------GITNENGYLETN----ER-------------------MET
BACC005112    YIGMLPLSKPFVEL------GITNENGYLETN----ER-------------------MET
BACC305043    YIGMLPLSKPFVEL------GITNENGYLETN----ER-------------------MET
BACAC05232    YIGMLPLSKPFVEL------GITNENGYLETN----ER-------------------MET
BACAA04720    YIGMLPLSKPFVEL------GITNENGYLETN----ER-------------------MET
BACT005085    YIGMLPLSKPFVEL------GITNENGYLETN----ER-------------------MET
BACC205124    YIGMLPLSKPFVEL------GITNENGYLETN----ER-------------------MET
BACC405064    YIGMLPLSKPFVEL------GITNENGYLETN----ER-------------------MET
BACT104712    YIGMLPLSKPFVEL------GITNENGYLETN----ER-------------------MET
BACLD03769    YIGMLPLSEPFKNL------GITNEEGYIVTN----EQ-------------------MET
BACPZ03376    YIGMLPLSKPFENL------GITNEEGYIETN----DR-------------------MET
BACSU03600    YIGMLPLSKPFENL------GITNEEGYIETN----DR-------------------MET
BACST01653    YIGMLPLSKPFENL------GITNEEGYIETN----DR-------------------MET
BACPT03541    YIGMLPLSKPFENL------GITNEEGYIETN----DR-------------------MET
LEUGG00661    YVGLKPNTQGFENL------DITNDDGWVITD----EQ-------------------MRT
LEUGJ00629    YVGLKPNTQGFENL------DITNDDGWVITD----EQ-------------------MRT
LEUCJ00485    YVGLEPNTQVFGNL------NITNDEGWIITD----EH-------------------MQT
LACAR00657    YVGVLPQTKPFENL------GILNEQGWIPTN----EH-------------------MET
LACA300647    YVGVLPQTKPFENL------GILNEQGWIPTN----EH-------------------MET
LACAL00643    YVGVLPQTKPFENL------GILNEQGWIPTN----EH-------------------MET
LACKZ00964    YVGVLPQTAPFKEL------GILDDQGWIVTD----DH-------------------MKT
LACRJ00357    YVGNFPMTAAFKNL------DILDDQGWVKTD----ER-------------------MRT
LACRD00361    YVGNFPMTAAFKNL------DILDDQGWVKTD----ER-------------------MRT
```

```
LACRS01501    YVGNFPKTAAFKNL------DILDDQGWVKTD----ER-------------------MRT
LACSM00455    YVGILPMTDAFRNI------GITDEASWIDTN----EL-------------------MET
LACRG00878    YVGIMPMTEPFQDL------GILDDHGWIPTD----EH-------------------MRT
LACRL00939    YVGIMPMTEPFQDL------GILDDHGWIPTD----EH-------------------MRT
LACC300844    YVGIMPMTEAFQDL------GVLDEHGWIPTD----EH-------------------MRT
LACCZ00824    YVGIMPMTEAFQDL------GVLDEHGWIPTD----EH-------------------MRT
LACCB00988    YVGIMPMTEAFQDL------GVLDEHGWIPTD----EH-------------------MRT
LACCD01016    YVGIMPMTEAFQDL------GVLDEHGWIPTD----EH-------------------MRT
LACCC01014    YVGIMPMTEAFQDL------GVLDEHGWIPTD----EH-------------------MRT
LACBN01277    YVGLLPMTDAFKDL------DITDEDGWIQTN----DQ-------------------MET
LACBA00605    YVGTLPMTDAFTDL------GITDDAGWIKTN----DH-------------------MAT
LACPL00650    YVGINPITKPFSNL------GITDENGWIETN----DH-------------------MET
LACPJ00628    YVGINPITKPFSNL------GITDENGWIETN----DH-------------------MET
LACPS00585    YVGINPITKPFSNL------GITDENGWIETN----DH-------------------MET
PEDCP00497    YVGVQPMTAPFKGL------GILDEDGWIPTD----EL-------------------MHT
CARS100359    YVGILPNTDKFRDL------GITDEEGWIPTN----ET-------------------MET
AERUA00263    YVGLLPNSEAFRDL------GITDEEGWILTD----EN-------------------MAT
ELUMP00590    AIGGVPQTAFLKNS-----GVELEEDGIIKID----ES-------------------GKT
SPHPG02940    FVGMLPQTALLDAK-------LLDKSGYVITN----EK-------------------MET
SPHGB01868    FVGMLPQTDLLDKT-------ILDESGYVITN----DR-------------------MET
TREPZ00273    FVGTTPQTSLVPDA-------EKDEAGYIVTD----QR-------------------MAS
TREAZ03414    FTGTDPQTALVSDV-------KKDETGYIITD----QR-------------------MAS
SPITD00734    FIGSDPQTGFVEGV-------EKDESGYIITN----QE-------------------MMS
SPITZ01373    FIGSDPQTGFVEGV-------EKDESGYIITN----QE-------------------MMS
TREPA00803    FIGMVPITGLLPDA-------EKDSTGYIVTD----DE-------------------MRT
TREPS00802    FIGMVPITGLLPDA-------EKDSTGYIVTD----DE-------------------MRT
TREPC00747    FIGMVPITGLLPDA-------EKDSTGYIVTD----DE-------------------MRT
TREPM00823    FIGMVPITGLLPDA-------EKDSTGYIVTD----DE-------------------MRT
TREPD00824    FIGMVPITGLLPDA-------EKDSTGYIVTD----DE-------------------MRT
TREPU00781    FIGMVPITGLLPDA-------EKDSTGYIVTD----DE-------------------MRT
ENCCU00216    GIGHDPNTSFLKNT-----KVEMDLNGYIVVR----DE-------------------VYT
HELM100964    FVGYDVNNQILKQKD-GKMLCDTDEYGSVLVD----LS-------------------MRT
HELCP01490    FVGYDVNTATLKQDD-GSMLCECDEWGSVKVD----LS-------------------MKT
ARCFU01526    AIGMRPATDVVAEL-----GVERDSMGYIKVD----KE-------------------QRT
FERPA02445    AVGIVPATDIVMDL-----GVERDAAGYIKVD----KR-------------------QRT
ARCVS01910    AVGIRPQTEIVVNL-----GVERDSKGYIKVD----RR-------------------QAT
METEZ00677    YVGIDPNTDLID--------VEKDESGFIITN----EF-------------------MET
METHD00869    YIGILPNTEFID--------VKKNSSGFILTN----EK-------------------LET
METMA02304    YVGIHPNTEFVD--------VEKDEGGFIKTD----RW-------------------MET
METAC01311    YVGIQPNTEFVN--------VEKNNEGFIITN----RW-------------------MET
KOSOT00298    FIGLVPNTGFLKGK------VKTNDWGYIMTD----EH-------------------MET
MARPK01613    FVGLTPVTELFKGK------IELDDYGYIPVD----EH-------------------RET
SLAHD02439    FIGFLPNTDLYEGQ------IEL-ERGYVVTD----DN-------------------MKT
FILAD00976    FIGLIPQTELFKGL------LDMDEQGYIISD----DK-------------------MQT
BUTPB02463    AIAIVPSTDLFENM------IECDEEGYVIAN----ED-------------------GAT
CLOPH00250    AVGNVPNSEVYTPA------VAVDRGGYIIAD----ES-------------------CET
CLOSW00592    AVGINPQSEAFNNL------VEMD-HGYIKAA----ED-------------------CET
LACFC00207    YVGVVPMTSAFKDL------GILDERGWVKTD----EK-------------------MAT
CRYCD00853    AIGKLPNTEAFVGQ------VPLTDAGYIRTE----EN-------------------GAT
EGGLE01847    AVGTEPNTEFLGGA------LQCDETGYIVAD----ES-------------------CAT
PYRFU01410    FIGYEPKTDFVKHL------GITDEYGYIKVD----MY-------------------MRT
PYRHO01476    FIGYEPKTDFVKHL------GITDEYGYIPVD----MY-------------------MRT
PYRAB00730    FIGYEPKTDFVKHL------GITDEYGYIKVD----MY-------------------MRT
PYRSN00014    FIGYEPKTDFVKHL------GITDEYGYIKVD----MY-------------------MRT
THEGJ00181    FIGYEPKTDFVKHL------GITDDYGYIPVD----MH-------------------MRT
THEKO02097    FIGYEPKTDFVKHL------GITDEYGYIPVD----MY-------------------MRT
THEON01610    FIGYEPKTDFVKHL------GITDEYGYIPVD----MH-------------------MRT
THES401476    FIGYEPKTDFVKHL------GITDEYGYIPVD----MH-------------------MRT
SYNWW02368    SIGLVARTELFGGM------LES-REGYIVTS----DN-------------------MMS
UNCTG00012    FTGLIPNTLFLSG-------VALDKTGYIITD----ED-------------------MNT
THEA101460    FIGNEPNVAPVAHL------VETTEQGFIITD----EE-------------------MKT
DENA201647    FIGQSAKTELVKDL------VDTDEWGFIIAT----ES-------------------TQT
DEFDS00488    FIGWNADTEAFKGL------LELDESGFIIAD----ET-------------------THT
CALNY01292    FIGWLADTEHFKGL------LEMDESGFIVAN----ES-------------------TKT
SYNGF01652    YIGSQPNSQFARKY------FTTNDTGYMMTD----EF-------------------LRS
```

```
DESB201123    FVGTRPSTGFCAGA------VELDQQGFIKTF----GD-------------------QKT
DEIPM00845    FIGHIPNTSFLGDT------VKLRDDGYVEVR----DD-------------------IYT
DEIRA01924    FIGHVPNTAFVKDT------VSLRDDGYVDVR----DE-------------------IYT
DEIML01437    FIGHVPNTDFVRDT------VKLRDDGYVEVT----DD-------------------IYT
DEIGD02532    FIGHVPNTEFVRDT------VKLRPDGYVEVT----DE-------------------IYT
DEIDV00622    FIGHTPNTEFVKDT------VKLRPDGYVDVT----DE-------------------IYT
TRURR01079    YIGHVPNTAYLEGL------VKLRESGYIDVR----DE-------------------IYT
THETG01863    FIGHEPNTAFLKGV------VELRPDGYIAVR----DE-------------------VFT
THET201543    FIGHEPNTAFLKGV------VELRPDGYIAVR----DE-------------------VFT
THET801911    FIGHEPNTAFLKGV------VELRPDGYIAVR----DE-------------------VFT
OCEP502115    FVGSVPNTDFLKGV------VELRPDGYVAVT----DE-------------------ILT
DEHLB00585    AVGLIPNTDYLKGV------VDLDRNGAVVTN----VR-------------------MET
DEHMG00439    AVGLQPNTTYLKGV------VDMDKNGSILVN----DQ-------------------MET
DEHMB00497    AVGLQPNTTYLKGV------VDMDKNGSILVN----NQ-------------------MET
DEHMC00427    AVGLQPNTTYLKGV------VDMDKNGSILVN----DQ-------------------MET
THELD00726    FVGMEPNSKFLGDM------VDAAPGGWIKTN----EK-------------------MET
ANAMD00624    FVGMDPNSEFVKGL------VEMKDGGWIVTN----DR-------------------LET
THEAS00815    FVGTAPNSGFASSL------VETSPGGWIKTN----SR-------------------MET
AMICL00707    FVGQAPHDECIRGL------VEAKKGGWIVTN----DD-------------------MET
CLOCE01684    AIGLNPNNSLVKDK------LELTKEGYVITD----DR-------------------MRT
HYDS000616    FIGMIPATDIVKDL------VELDEYGYIKVN----DN-------------------MET
HYDTT00224    FIGLEPNTGFLKGS------VELDEKGYVITD----EK-------------------MMT
PELTS01405    YVGQKPNSDLVRDI------VSLDEKGYIITD----EN-------------------MMT
DESRL02179    YVGTRANSELVQDL------VKLDDRGYIITD----EN-------------------MST
BORBP00506    AVGYKPNTEFLKGF------LDLDEEGFIITK----DI-------------------VKT
BORAP00518    AVGYKPNTEFLKGF------LDLDEEGFIITK----DV-------------------VKT
BORBU00514    AVGYKPNTEFLKGF------LDLDEEGFIVTK----DV-------------------VKT
BORBZ00490    AVGYKPNTEFLKGF------LDLDEEGFIVTK----DV-------------------VKT
BORBN00493    AVGYKPNTEFLKGF------LDLDEEGFIVTK----DV-------------------VKT
BORRA00486    AIGYKPNTEFVKGF------LELDDDGYISTQ----DI-------------------VKT
BORDL00498    AIGYKPNTEFVKGF------LKLDDDGYISTQ----DI-------------------VKT
BORHD00497    AIGYKPNTDFLKGF------LELDEDGYISTQ----AL-------------------VKT
BORT900497    AIGYKPNTEFVKGF------LELDEDGYISTQ----DL-------------------VKT
METKA01560    AAGKVPNSEPFRDL------VETDDRGYIVVD----SE-------------------MRT
MYCA500361    YIGQLPATDFLDKE-----I--LNERKFIEVD----HN-------------------MET
METVS00145    SYGYVPNTEFLGDT-----NIELKKGNFINVD----KD-------------------CKT
METOI01431    SMGHIPNSEFLESS-----GIALNKKGFIIVD----KS-------------------CRT
MYCHN00051    YVGLIPENEFLKSI-----DIEKDEWGFLKVD----EN-------------------MRT
MYCSL00465    FIGLLPETDFLSSL-----ALKRDERGFILVD----HE-------------------MRT
MYCS300420    FIGLLPETDFLSSL-----ALKRDERGFILVD----HE-------------------MRT
DESK101258    EIGLEPPVEFFRRI-----GLETDETGRARVN----ID-------------------RST
STAHD01472    EIGLIPPKELFEKI-----GIETDEYGYAKVG----PD-------------------QST
THEC100270    EIGLKPPREFFEKI-----GLEVDETGHAVVK----VD-------------------RST
NANEQ00478    AIGMETNIELAKKL-----GVQLDERGKIIVD----RC-------------------QRT
CALLD01225    EIGFEPPKDWYQSL-----GLETDEAGYIKVD----DW-------------------MRT
SULSO02155    EIGFDPPTDFAKSN-----GIETDTNGYIKVD----EW-------------------MRT
SULS900210    EIGFDPPTDFAKSN-----GIETDTNGYIKVD----EW-------------------MRT
SULIA00208    EIGFDPPTDFAKSN-----GIETDTNGYIKVD----EW-------------------MRT
SULIM00207    EIGFDPPTDFAKSN-----GIETDTNGYIKVD----EW-------------------MRT
SULIK00225    EIGFDPPTDFAKSN-----GIETDTNGYIKVD----EW-------------------MRT
IGNH400907    SYGEVPSTEIFKKA-----GVEVDERGFIKID----PY-------------------TRT
KORCO01040    LIGVEPNSKIAREA-----GVETDERGFIKVD----CW-------------------QRT
THESM01133    FIGYEPKTDFVKHL-----GIT-DEQGYILVD----MY-------------------MRT
THEBM01534    FIGYEPKTDFVKHL-----GIT-DEWGYIPVD----MY-------------------MRT
METST01363    AIGYTPNNKLIKDF-----GISCDDLGYIEVD----EN-------------------MKT
METTH00703    AIGEEPLNQLAVDL-----GVEVDKGGYIITD----KF-------------------QRT
METSL02406    AVGEVPNSQIANDL-----GVEIDDLNYIVAD----RD-------------------QKT
METLA02406    AVGEVPNSQIANDL-----GVEIDDLNYIVAD----RD-------------------QKT
METPW00194    AVGEIPSSQLAGPI-----GVEIDRGGYIITD----KD-------------------QKT
RUBXD00226    AIGHDPATEIFRG-----Q-LEMDEGGYLLQK---E-H-------------------TMT
RHOM400178    AIGHKPNTEVFRG-----W-LEMDEQGYIKTK---NCS-------------------THT
GARV400051    AIGHTPATKFLND-----Q-LKLNEDGTIVVD---GAT-------------------TRT
BIFAP01644    AIGFTPQTAFFNG-----Q-VDLDQDGYILVK---GGS-------------------TKT
SEGRD00014    AIGHDPRAELVVG-----Q-VDQDEDGYVKVQ---APS-------------------SAT
GORB404535    AIGHDPRSELVRG-----Q-VDLDSEGYVQVQ---GRT-------------------TYT
```

```
GORPV04920    AIGHDPRSELVRG-----Q-VDLDSDGYVQVQ---GRT-------------------TYT
MYCA904903    AVGHDPRSELVKD-----V-VDVDPDGYVLVR---DRS-------------------TYT
MYCSS05356    AIGHVPRSDIVRG-----Q-VDVDEDGYVLVE---GRT-------------------TNT
MYCSJ05684    AIGHVPRSDIVRG-----Q-VDVDEDGYVLVE---GRT-------------------TNT
MYCSK05387    AIGHVPRSDIVRG-----Q-VDVDEDGYVLVE---GRT-------------------TNT
MYCS206581    AIGHDPRSELVRG-----Q-VELDDEGYVKVQ---GRT-------------------TYT
MYCCN05158    AIGHDPRSELVRG-----Q-VDLDDEGYVQVE---GRT-------------------SYT
MYCVP05890    AVGHDPRSELVRD-----Q-VEVDDAGYVKVQ---GRT-------------------TYT
MYCGI00775    AIGHDPRSELVRG-----Q-VDVDDEGYVSVQ---GRT-------------------TYT
MYCSR04994    AIGHDPRSELVRG-----Q-VDVDDEGYVSVQ---GRT-------------------TYT
AMYSD04545    AVGHDPRSELVRD-----Q-LDVDEDGYVLVK---TPT-------------------THA
MYCLE02681    AIGHEPRSSLVSD-----V-VDIDPDGYVLVK---GRT-------------------TST
MYCLB02681    AIGHEPRSSLVSD-----V-VDIDPDGYVLVK---GRT-------------------TST
MYCSD04323    AIGHDPRSEMVRE-----A-VQVDADGYVLVE---QPS-------------------SRT
MYCPA04306    AIGHDPRSELVRD-----V-LDTDPDGYVLVQ---GRT-------------------TAT
MYCA105023    AIGHDPRSELVRD-----V-LDTDPDGYVLVQ---GRT-------------------TAT
MYCUA04107    AIGHEPRSSLVRD-----A-VDVDPDGYVLVN---GRT-------------------TGT
MYCMM05378    AIGHEPRSSLVRD-----A-VDVDPDGYVLVN---GRT-------------------TGT
MYCA003905    AIGHEPRSGLVRE-----A-IDVDPDGYVLVQ---GRT-------------------TST
MYCTU03943    AIGHEPRSGLVRE-----A-IDVDPDGYVLVQ---GRT-------------------TST
MYCTF03846    AIGHEPRSGLVRE-----A-IDVDPDGYVLVQ---GRT-------------------TST
MYCTA03980    AIGHEPRSGLVRE-----A-IDVDPDGYVLVQ---GRT-------------------TST
MYCTK04010    AIGHEPRSGLVRE-----A-IDVDPDGYVLVQ---GRT-------------------TST
MYCTC03612    AIGHEPRSGLVRE-----A-IDVDPDGYVLVQ---GRT-------------------TST
MYCTD03548    AIGHEPRSGLVRE-----A-IDVDPDGYVLVQ---GRT-------------------TST
MYCCP03898    AIGHEPRSGLVRE-----A-IDVDPDGYVLVQ---GRT-------------------TST
MYCBO02863    AIGHEPRSGLVRE-----A-IDVDPDGYVLVQ---GRT-------------------TST
MYCBP03913    AIGHEPRSGLVRE-----A-IDVDPDGYVLVQ---GRT-------------------TST
MYCBT03913    AIGHEPRSGLVRE-----A-IDVDPDGYVLVQ---GRT-------------------TST
TSUPD04078    AIGHDPRSALVAD-----Q-VTVDEEGYVQVE---GRS-------------------TAT
ACTMD06897    AIGHDPRSALVRG-----Q-VDVDDEGYVKVQ---PHS-------------------TYT
SACES08402    AIGHDPRSELVRG-----Q-VDVDDAGYVKVE---GQT-------------------TYT
SACVD03814    AIGHDPRSELVRG-----Q-VDTDEDGYVLTK---GRT-------------------SHT
AMYMU09190    AIGHDPRSQLVKG-----Q-VELDEDGYVLTQ---GRT-------------------SFT
AMYMS10178    AIGHDPRSQLVKG-----Q-VELDEDGYVLTQ---GRT-------------------SFT
PSEUX06421    AIGHDPRSELVRD-----V-LDVDDDGYVRVD---SPS-------------------TRT
NOCFA05681    AIGHDPRSELVRD-----Q-VELDAEGYVVVE---HPT-------------------TAT
NOCCG05476    AIGHDPRSELVRG-----Q-VELDDEGYVKVA---DPS-------------------TAT
RHOE406010    AIGHDPRSELVKG-----Q-VDLDDAGYVRVA---PGS-------------------TAT
RHOOB03468    AIGHDPRSELVKG-----Q-VDLDEAGYVIVQ---NPT-------------------TAT
RHOE104515    AIGHDPRSELVKG-----Q-VDVDDAGYVKVQ---SPT-------------------TAT
CORDI02302    AIGHDPRSAMFRD-----V-VTTDAAGYVVVE---HPS-------------------TKT
CORD202219    AIGHDPRSAMFRD-----V-VTTDAAGYVVVE---HPS-------------------TKT
CORDL02208    AIGHDPRSAMFRD-----V-VTTDAAGYVVVE---HPS-------------------TKT
CORDJ02208    AIGHDPRSAMFRD-----V-VTTDAAGYVVVE---HPS-------------------TKT
CORDH02221    AIGHDPRSAMFRD-----V-VTTDAAGYVVVE---HPS-------------------TKT
CORD702316    AIGHDPRSAMFRD-----V-VTTDAAGYVVVE---HPS-------------------TKT
CORD302337    AIGHDPRSAMFRD-----V-VTTDAAGYVVVE---HPS-------------------TKT
CORDD02235    AIGHDPRSAMFRD-----V-VTTDAAGYVVVE---HPS-------------------TKT
CORDV02170    AIGHDPRSAMFRD-----V-VTTDAAGYVVVE---HPS-------------------TKT
CORDW02254    AIGHDPRSAMFRD-----V-VTTDAAGYVVVE---HPS-------------------TKT
CORDK02230    AIGHDPRSAMFRD-----V-VTTDAAGYVVVE---HPS-------------------TKT
COREF02870    AIGHDPRSEMLVG-----Q-VELDSSNYVVAE---HPS-------------------TRT
CORGL03082    AIGHDPRSEILAG-----Q-VEVDPSNYVLVQ---EPS-------------------TRT
CORGK02974    AIGHDPRSEILAG-----Q-VEVDPSNYVLVQ---EPS-------------------TRT
CORGB03038    AIGHDPRSEILAG-----Q-VEVDPSNYVLVQ---EPS-------------------TRT
CORK402006    AIGHDPRSEMVRD-----V-IDTDDKGYILVQ---EPS-------------------TRT
CORJK02028    AIGHDPRTAVFEG-----Q-VELQDNGYVKVE---EPS-------------------TRT
CORVD02951    AIGHDPRTTVFEG-----Q-VELQDNGYVKVA---EPS-------------------TAT
ARCHD01706    AIGHKPRSGMLDG-----Q-VDLDDHGYIVVD---SPS-------------------AKT
THET101205    AIGHKPNTDIFVG-----Q-VELDEKGYIKAQD--SWS-------------------TAT
PROAC02247    AIGHDPRSELVRG-----Q-VDLDDAGYVVCA---EPS-------------------TRT
PROAS02302    AIGHDPRSELVRG-----Q-VDLDDAGYVVCA---EPS-------------------TRT
CAERE29798    AIGHDPRSELVIG-----Q-VDTDADGYVRVA---HPS-------------------TRT
CELFA03749    AIGHVPRTDLLVG-----Q-VDLDDNGYVSVQ---GRS-------------------TLT
```

```
JONDD02484    AIGHDPRSELINT-----Q-VDTDANGYVLVK---GRS-------------------TLT
XYLCX03324    AIGHEPRTELVKG-----Q-VDLDDEGYILVE---GRS-------------------TRT
ACIC102144    AIGHEPRSELFRG-----Q-LEIDAAGYIQVA---HPT-------------------TRT
FRADG04042    AIGHEPRTELVRG-----Q-LDLDAAGYVAVE---HPS-------------------TRT
FRASU07065    AIGHVPRSELFRD-----Q-VTLDDEGYVLVE---APS-------------------TRT
FRASN07115    AIGHLPRTDLIHD-----Q-LELDGEGYIKVT---HPT-------------------TQT
FRASC04424    AIGHLPRTELVRG-----Q-LDLDDAGYISVA---HPT-------------------TKT
FRAAA06712    AIGHEPRTELLRG-----Q-LELDDAGYVKVA---HPT-------------------TKT
KYTSD02521    AIGHDPRNELLTG-----Q-VDLDESGYVMVE---GRS-------------------TRT
KINRD04462    AIGHEPRTDLVKG-----Q-VDLDENGFVLVE---GRS-------------------TRT
NOCDD04789    AIGHDPRVDLFDK-----Q-IELDEEGYVKVD---FPS-------------------TRT
NOCAA01941    AIGHDPRVELFKD-----Q-IDLDEEGYVQVE---FPS-------------------TRT
STRRD08913    AIGHDPRTELVKG-----Q-VELDDEGYIKVD---SPS-------------------TRT
THECD04863    AIGHDPRSELFKG-----Q-LDIDENGYLVVD---SPS-------------------TRT
THEBD03528    AIGHEPRSALVKG-----Q-VALDEQGYIIVD---SPT-------------------TRT
CATAD08894    AIGHLPRTELFHG-----V-LDLDDEGYIKVD---SPT-------------------TRT
KRIFD06917    AIGHDPRSELVKG-----Q-VHLDEEGYVLVQ---PGS-------------------TAT
KITSK03730    AIGHDPRTDLFKG-----Q-LDLDAEGYLKVE---APS-------------------TRT
STRBB05366    AIGHDPRTELFKG-----Q-LDLDDEGYLKVA---SPS-------------------TRT
STRVP03660    AVGHDPRTELFKG-----Q-LDLDEEGYLKVA---APS-------------------TRT
STRSW04381    AIGHDPRTELFKG-----Q-LDLDDEGYLKVA---SPS-------------------TRT
STRGG03562    AVGHDPRTELFKG-----Q-LELDDEGYLKVE---APS-------------------TRT
STRFA03098    AVGHDPRTELFKG-----Q-LDLDDEGYLKVE---APS-------------------TRT
STRAW04303    AIGHDPRTELFKG-----Q-LDLDEEGYLKVD---APS-------------------TRT
STRCO02929    AIGHDPRTELFKG-----Q-LDLDPEGYLKVD---APS-------------------TRT
STRHJ05105    AIGHDPRTELFKG-----Q-LDLDEEGYLKVD---SPS-------------------TRT
MONBE04991    AIGHEPASAFLNG-----Q-LELDEDRYIVT---KPDS-------------------TAT
CHLRE01313    AIGHQPATAFLNG-----Q-LALDAEGYIVT---APDS-------------------TAT
MEDTR25591    AIGHEPATKFLDG-----Q-LELDSDGYVVT---KPGT-------------------TKT
SOLLC13750    AIGHVPATKFLDK-----Q-LELDSDGYVVT---KPGT-------------------TLT
PRUPE10733    AIGHEPATKFLGG-----Q-LELHPDGYVAT---KPGT-------------------TQT
MANES18605    AIGHEPATKFLDG-----Q-LQLDSDGYVVT---KPGT-------------------TQT
THECC00884    AIGHEPATKFLEG-----Q-LQLDSDGYILT---KPGT-------------------TQT
PHYPA31147    AIGHEPASKFLGG-----Q-VETDADGYIVT---KPGT-------------------TQT
AMBTC19471    AIGHVPATAFLRG-----Q-LEMDGEGYVLT---KPGT-------------------TQT
MUSAC26038    AIGHEPATKFLGG-----Q-LELDSDGYVVT---KPGT-------------------THT
MUSAM33177    AIGHEPATKFLGG-----Q-LELDSDGYVVT---KPGT-------------------THT
SETIT03079    AIGHEPATKFLGG-----Q-LELDSDGYVAT---KPGS-------------------THT
ORYBR12195    AIGHEPATKFLGG-----Q-LELDADGYVAT---KPGS-------------------THT
COCLU07729    AVGHDPATALFKG-----Q-LDMDEDGYLIT---KPGE-------------------GLT
PHANO13702    AVGHDPATEIFKG-----Q-LDMDEDGYLIT---KPGE-------------------GLT
PHAND10804    AVGHDPATEIFKG-----Q-LDMDEDGYLIT---KPGE-------------------GLT
AURPU02089    AVGHEPATTLFKD-----Q-LKMDEDGYLIT---EPGT-------------------THT
ZYMTR07711    AVGHDPATHLFNG-----Q-VKMDEDGYIIT---TPGT-------------------TET
DICPU05926    AIGHTPNSSFLGG-----Q-LDVDETGYIKT---VPGS-------------------SKT
ENTHI00522    AIGHSPNSKFLGG-----Q-VKTADDGYILT---E--G-------------------PKT
LEPBA02231    AIGHVPNTEIFKG-----Q-LNLDETGYIIT---KPGT-------------------TQT
LEPBP02301    AIGHVPNTEIFKG-----Q-LNLDETGYIIT---KPGT-------------------TQT
LEPBL01462    AIGHKPNTDIFKG-----I-LDLDESGYIKT---VPGS-------------------TKT
LEPBJ01276    AIGHKPNTDIFKG-----I-LDLDESGYIKT---VPGS-------------------TKT
LEPIN02475    AIGHKPNTDIFQG-----I-LDLDESGYIKT---VPGS-------------------TKT
LEPII01996    AIGHKPNTDIFQG-----I-LDLDESGYIKT---VPGS-------------------TKT
LEPIC01426    AIGHKPNTDIFQG-----I-LDLDESGYIKT---VPGS-------------------TKT
SPIAZ00697    AIGHKPNTDFLGD-----A-LDKDETGYLVT---VPGT-------------------TRT
PENRW10140    AVGHDPASGLVKG-----Q-VELDDEGYIIT---KPGT-------------------SFT
PENCH09104    AVGHDPASGLVKG-----Q-VELDDEGYIIT---KPGT-------------------SFT
EURHE07269    AVGHDPANALVKE-----Q-VDLDEDGYIIT---QAGT-------------------SYT
ASPAC07301    AVGHDPATALVKG-----Q-INLDEDGYIVT---QPGT-------------------SYT
EMENI10387    AVGHDPATALVKG-----Q-VELDEDGYIAT---KPGT-------------------SFT
EMEND02596    AVGHDPATALVKG-----Q-VELDEDGYIAT---KPGT-------------------SFT
ASPTN06742    AVGHDPATGLVKG-----Q-VDLDEDGYILT---KPGT-------------------SYT
ASPCL04014    AVGHDPATALIKG-----Q-IDLDEDGYIIT---KPGT-------------------SYT
ASPFU05647    AVGHDPATALVKG-----Q-IDLDEDGYIIT---KPGT-------------------SYT
NEOFI00452    AVGHDPATALVKG-----Q-IDLDEDGYIIT---KPGT-------------------SYT
CRYPA10563    AIGHDPASTLVKG-----Q-IDTDEDGYIVT---KNGT-------------------PLT
```

```
BLUGR03498     AVGHDPATALVKG-----Q-LATDEEGYIIT---KPGT-------------------SYT
SCLS112814     AVGHDPATALFKE-----Q-IDTDSEGYIVT---KPGT-------------------SYT
MAGGR04266     AIGHDPATQLVKG-----Q-LETDEEGYIVT---KPGT-------------------PLT
NEUCR01575     AIGHDPATALVKG-----Q-LETDADGYVVT---KPGT-------------------TLT
NEUT908941     AIGHDPATALVKG-----Q-LETDADGYVVT---KPGT-------------------TLT
VERDA02342     AIGHVPATSLVQG-----Q-LDTDDEGYVIT---QPGT-------------------PLT
COLSU12486     AIGHEPATSLVKG-----Q-LETDSEGYVVT---KPGT-------------------PLT
HYPAI01684     AVGHDPATNLVKG-----Q-VDVDSEGYIIT---KPGT-------------------TET
HYPVG06080     AVGHDPATALVKG-----Q-VDTDSEGYIVT---KPGT-------------------TET
HYPJE05895     AVGHDPATGLVKG-----Q-VDVDAEGYIIT---KPGT-------------------TET
NECHA05020     AIGHDPATSLVKG-----Q-IKMDEDGYIQT---IPGT-------------------TNT
FUSO415847     AIGHDPATTLVKG-----Q-VDMDEDGYIKT---VPGT-------------------TYT
GIBZA01026     AIGHDPATTLVKG-----Q-VDMDEDGYIKT---IPGT-------------------TYT
SCHPO04025     AIGHIPATKLVAE-----Q-IELDEAGYIKT---INGT-------------------PRT
YARLI03635     AIGHIPATSIVKG-----Q-VETDEEGYVVT---VPGT-------------------ANT
ASHGO00946     AIGHTPATQLVAG-----Q-VDLDESGYVKT---VPGS-------------------TLT
KLULA02190     AIGHTPATNIVAG-----Q-VDLDEAGYVKT---VPGS-------------------TLT
ZYGRO00676     AIGHTPATSIVQG-----Q-VTTDESGYIKT---IPGT-------------------AET
DEKBR01813     AIGHKPATEIFKD-----Q-IECDETGYIKT---KPDS-------------------SLT
PICPG04776     AIGHIPATKIFAD-----Q-LESDEDGYLKT---IPGS-------------------SLT
CANTE00916     AIGHTPATAVFGK-----Q-IETDDTGYIKT---TPGS-------------------THT
LODEL03891     AIGHIPATQIFAN-----Q-LKMDDDGYIQT---KPGT-------------------AQT
DEBHA05546     AIGHIPATQIFAK-----Q-LETDDQNYILT---KPGT-------------------AET
SPAPN03477     AIGHVPATKIFEG-----Q-LDMDETGYIKT---VPGT-------------------AST
CANAW04800     AIGHIPATKIFAD-----Q-LKTDEAGYIQT---TPGT-------------------AST
PICST04701     AIGHTPATQVFAN-----Q-LKTDSEGYIVT---TPGT-------------------AET
PUCGT10887     AIGHEPATALVRG-----Q-LKTDEDGYICT---KPGT-------------------SET
PUCGR11813     AIGHEPATALVRG-----Q-LKTDEDGYICT---KPGT-------------------SET
PHYBL11006     AIGHVPATELVKG-----Q-VNLDNDGYIST---IPGT-------------------AET
USTMA03757     AIGHLPATSLVKG-----Q-VDLDSDGYIIT---TPGT-------------------AQT
USTHO04132     AIGHLPATSLVKS-----Q-VELDSDGYIIT---TPGT-------------------AQT
WALSE04527     AIGHEPATQLVRS-----Q-VDTDEDGYIKT---VPGT-------------------TQT
TREME07701     AIGHEPATALVKD-----Q-LELDADGYIQV---VPGT-------------------AQT
AURST04751     AIGHEPATELVRS-----Q-LECDGDGYIKT---VPGT-------------------TQT
FOMME10177     AIGHEPATAIVRG-----Q-VQTDSDGYIVT---VPGT-------------------SQT
CONPW06392     AIGHEPATSLVSS-----Q-LQTDSDGYIIT---IPGT-------------------TQT
STEHR07076     AIGHEPATSLVRD-----Q-LQTDADGYIIT---VPGT-------------------TQT
HETAN06295     AIGHEPATSLVRD-----Q-LQTDADGYIIT---VPGT-------------------TQT
GLOTR06982     AIGHEPATSLVRD-----Q-LQTDADGYIVT---VPGT-------------------TQT
PUNST01981     AIGHEPATALVRD-----Q-LQTDDDGYIVT---VPGT-------------------TQT
LACBI02877     AIGHEPATALVRS-----Q-LETDPDGYIIT---VPGT-------------------TQT
COPCI16429     AIGHEPATALVRN-----Q-LQTDPDGYIIT---VPGT-------------------TQT
DICSQ11618     AIGHEPATQLVRG-----Q-LQTDPDGYIVT---VPGT-------------------TQT
TRAVS13180     AIGHEPATQLVRN-----Q-LQTDLDGYIVT---VPGT-------------------TQT
WOLCO03584     AIGHEPATALVRG-----Q-LQTDPDGYIIT---VPGT-------------------TQT
FOMPI05979     AIGHEPATALVRG-----Q-LQTDPEGYIIT---VPGT-------------------TQT
PHLGI10219     AIGHEPATALVRG-----Q-LQTDTDGYIVT---VPGT-------------------TQT
PHACH05757     AIGHEPATALVRG-----Q-LQTDPDGYIVT---VPGT-------------------TQT
RICTY00419     AIGHTPNTTLFNG-----Q-IAIDDDNYIIT---QTGS-------------------TRT
RICPR00429     AIGHAPNTALFKG-----Q-IAIDDDNYIVT---QSGS-------------------TRT
RICPP00461     AIGHAPNTALFKG-----Q-IAIDDDNYIVT---QSGS-------------------TRT
RICBR00434     AIGHKPNTALFAE-----Q-VTMDNDNYIIT---TPGS-------------------TKT
RICB800988     AIGHKPNTALFAE-----Q-VTMDNDNYIIT---TPGS-------------------TKT
RICCK00600     AIGHAPNTSLFTG-----Q-IAMDYDNYIIT---KPGT-------------------TKT
RICAH00614     AIGHVPNTGLFTG-----Q-IAMDDDNYIIT---ESGT-------------------TKT
RICAC00782     AIGHAPNTGLFTG-----Q-IAMDDDNYIIT---KSGT-------------------TKT
RICFE00656     AIGHAPNTGLFTG-----Q-IAMDDDNYIIT---KSGT-------------------TKT
RICMS00042     AIGHVPNTGLFTG-----Q-IVMDDDNYIIT---KSGT-------------------TRT
RICM500453     AIGHAPNTGLFTG-----Q-IVMDDDNYIIT---KSGT-------------------TRT
RICR300665     AIGHAPNTGLFTG-----Q-IVMDDDNYIIT---KSGT-------------------TRT
RICAG00651     AIGHAPNTGLFTG-----Q-IVMDDDNYIIT---KSGT-------------------TRT
RICP300630     AIGHAPNTGLFTG-----Q-IVMDDDNYIIT---KSGT-------------------TRT
RICRS00632     AIGHAPNTGLFTG-----Q-IAMDDDNYIIT---KSGT-------------------TRT
RICRO00663     AIGHAPNTGLFTG-----Q-IAMDDDNYIIT---KSGT-------------------TRT
RICCN00618     AIGHAPNTGLFTG-----Q-IVMDDDNYIIT---KSGT-------------------TRT
```

```
RICPT00620     AIGHAPNTGLFTG-----Q-IVMDDDNYIIT---KSGT-------------------TRT
RICAE00497     AIGHAPNTGLFTG-----Q-IVMDDDNYIIT---KSGT-------------------TRT
RICJY00467     AIGHAPNTGLFTG-----Q-IVMDDDNYIIT---KSGT-------------------TRT
RICPU00072     AIGHAPNTGLFTG-----Q-IVMDDDNYIIT---KSGT-------------------TRT
RICS100537     AIGHAPNTGLFTG-----Q-IVMDDDNYIIT---KSGT-------------------TRT
BARBK00891     AIGHDPVVSLFEG-----Q-LKQKPEGYLWT---EPDS-------------------TAT
BARVW00932     AIGHDPAVSLFEG-----Q-LKQKPGGYLWT---VPDS-------------------TAT
BART100617     AIGHDPAVSLFEG-----Q-LKQKQSGYLWT---APDS-------------------TAT
BARGA00466     AIGHDPAVSLFEG-----Q-LKQKRGGYLWT---APDS-------------------TAT
BARHE01107     AIGHDPAVSLFEG-----Q-LKQKSGGYLWT---APDS-------------------TAT
BARQU00895     AIGHDPAVSLFEG-----Q-LKQKPGGYLWT---APDS-------------------TAT
OCHA401642     AIGHAPAVSLFEG-----K-LKQKPNGYLWT---APDS-------------------TAT
BRUAB01390     AIGHAPAVSLFEG-----K-LKQKPNGYLWT---APDS-------------------TAT
BRUA201507     AIGHAPAVSLFEG-----K-LKQKPNGYLWT---APDS-------------------TAT
BRUA101339     AIGHAPAVSLFEG-----K-LKQKPNGYLWT---APDS-------------------TAT
BRUSU01458     AIGHAPAVSLFEG-----K-LKQKPNGYLWT---APDS-------------------TAT
BRUME00510     AIGHAPAVSLFEG-----K-LKQKPNGYLWT---APDS-------------------TAT
BRUSI01461     AIGHAPAVSLFEG-----K-LKQKPNGYLWT---APDS-------------------TAT
BRUC201446     AIGHAPAVSLFEG-----K-LKQKPNGYLWT---APDS-------------------TAT
BRUMC01440     AIGHAPAVSLFEG-----K-LKQKPNGYLWT---APDS-------------------TAT
BRUMB01421     AIGHAPAVSLFEG-----K-LKQKPNGYLWT---APDS-------------------TAT
BRUM501487     AIGHAPAVSLFEG-----K-LKQKPNGYLWT---APDS-------------------TAT
BRUO201285     AIGHAPAVSLFEG-----K-LKQKPNGYLWT---APDS-------------------TAT
RHILO01973     AIGHAPAVELFVG-----K-LKQKPNGYLWT---APNS-------------------TRT
CHESB02097     AIGHAPAVELFEG-----K-LKQKPNGYLWT---EPNS-------------------TRT
METPB00996     AIGHQPATGIFEG-----Q-LPMRHGGYITV---TPGT-------------------TAT
METEP01063     AIGHQPATGIFEG-----Q-LPMRHGGYITV---TPGT-------------------TAT
METEA00810     AIGHQPATGIFEG-----Q-LPMRHGGYITV---TPGT-------------------TAT
METED01453     AIGHQPATGIFEG-----Q-LPMRHGGYITV---TPGT-------------------TAT
METS403554     AIGHQPATALFEG-----Q-LPLRAGGYLAV---TPGT-------------------AAT
METNO05482     AIGHQPATAIFEG-----Q-LPLRAGGYLTV---TPGT-------------------TST
METSZ03234     AIGHSPASELFRG-----Q-LTIKPSGYIAV---EPGT-------------------TRT
BEII900056     AIGHQPASELFAG-----Q-LTMKANGYLWT---APDS-------------------TAT
METSB02743     AIGHKPASELFAG-----Q-LDIKPNGYIQT---APNS-------------------TAT
MAGMM00401     AIGHRPNTEIFGD-----Q-LDKDANGYLVT---KPGS-------------------TAM
HYPNA00542     AIGHAPSTELFVG-----K-LPMRESGYLIT---EPGS-------------------PRT
KETVY00902     AIGHAPASELVQG-----Q-LELYNGGYVKV---TPGS-------------------TAT
KETVW00472     AIGHAPASELVQG-----Q-LELYNGGYVKV---TPGS-------------------TAT
ROSDO03242     AIGHAPANELVKD-----V-LETHMGGYVVT---KPDS-------------------TET
ROSLO02615     AIGHAPANELVKD-----V-LETHMGGYVVT---KPDS-------------------TET
RUEPO00888     AIGHAPANELVKD-----V-LELHNGGYVSV---KPGT-------------------TET
RUEST00613     AIGHAPATELVKD-----V-LETHNGGYVKV---KPGT-------------------TET
PHAIB02390     AIGHAPATELVKD-----V-LETHNGGYVKV---TPGT-------------------TET
PARDP02130     AIGHAPASELVKD-----Q-LELHHGGYVKV---EPGT-------------------TRT
DINSH02620     AIGHAPASELVID-----Q-LETHMGGYVVT---APDS-------------------TAT
RHOCB02768     AIGHAPASELVKD-----Q-LELHHGGYVKV---EPGT-------------------TRT
RHOS500248     AIGHAPASELVKD-----Q-LELHHGGYVKV---EPGT-------------------TRT
RHOS400150     AIGHAPASELVKD-----Q-LELHHGGYVKV---EPGT-------------------TRT
RHOS100227     AIGHAPASELVKD-----Q-LELHHGGYVKV---EPGT-------------------TRT
RHOSK02952     AIGHAPASELVKD-----Q-LELHHGGYVKV---EPGT-------------------TRT
MIDMI00790     AIGHKPNTALFAG-----Q-LDLDEDMYIVT---KKGT-------------------TLT
ACEP301595     AIGHAPNTAIFRD-----Q-LTLDAEGYIET---TPGT-------------------TRT
MICAA01566     AIGHVPATEIFKG-----K-VNLDSEGYIVT---APDS-------------------TAT
TISMK03676     AIGHAPATALFQG-----Q-LELDEEGYLIT---RPDS-------------------TRT
AZOL402409     AIGHVPATGIFQG-----K-VKMDDSGYILT---APDS-------------------TAT
PSEUV04348     AIGHAPAVDLVKD-----Q-VKMKESGYIWT---EADS-------------------TQT
HIRBI01095     AIGHAPATSLFDG-----Q-LEMKQGGYLIT---APDS-------------------SKT
PARL102241     AIGHSPATELFKD-----Q-LKTKAGGYLVT---APDS-------------------TAT
MARMM02139     AIGHAPATELFLD-----Q-LETKDGGYLVV---EPGT-------------------PKT
PHEZH02588     AIGHAPASELFKG-----Q-LEMDASGYLKV---KPGT-------------------AST
CAUCR02826     AIGHAPSSELFAG-----Q-LETGSGGYLKV---KPGT-------------------AST
CAUCN02939     AIGHAPSSELFAG-----Q-LETGSGGYLKV---KPGT-------------------AST
CAUST00891     AIGHAPSSELFAG-----Q-LETGPGGYLKV---KPGT-------------------AST
PARBH01522     AIGHAPATQIFEG-----H-LEMKPNGYIVT---APDS-------------------TAT
PELHB02084     AIGHAPATEIFNG-----K-LEMKHGGYIVT---APDS-------------------TAT
```

```
HYPDA03273      AIGHAPATELFKG-----Q-LETTPSGYLIT---APDS-------------------TAT
HYPSM04642      AIGHAPATELFKG-----Q-LETTPSGYLIT---APDS-------------------TAT
OLICO01107      AIGHAPATELVQG-----Q-LALTPSGYIEI---APHS-------------------TAT
OLICM02790      AIGHAPATELVQG-----Q-LALTPSGYIEI---APHS-------------------TAT
RHOPS01446      AIGHAPATELVKD-----Q-LKLKPSGYVEV---APNS-------------------TAT
RHOPA04062      AIGHAPATELFKD-----Q-IKLKPSGYVEV---AANS-------------------TAT
RHOPT04533      AIGHAPATELFKD-----Q-IKLKPSGYVEV---AANS-------------------TAT
RHOPX04235      AIGHAPATELFKD-----Q-VKLKPSGYVEV---AANS-------------------TAT
BRADU07321      AIGHAPATELVKD-----Q-VKLKPSGYVEV---APNS-------------------TAT
BRASO01239      AIGHAPATELVAG-----Q-LKLKPSGYVEV---VPGS-------------------TAT
BRASB06352      AIGHAPATELVAG-----Q-LKLKPSGYVEV---VPGT-------------------TAT
RHOPB03773      AIGHAPATELVKG-----K-IRLKPSGYVEV---APNS-------------------TAT
NITWN02324      AIGHAPATELVTG-----K-VKLKPSGYVEV---APNS-------------------TAT
NITHX02707      AIGHAPATELVAG-----K-VKLKSSGYIEV---APNS-------------------TAT
AZOC501143      AIGHAPSTALVEG-----Q-LRIKSGGYVWT---APDS-------------------TAT
XANP202679      AIGHAPATELVKD-----Q-LKLKESGYVWT---APDS-------------------TAT
CHLTF01983      AIGHQPNTSLFVG-----Q-LELNPQGYILT---KPGS-------------------THT
IGNAJ02236      AIGHKPNTELFKN-----Y-LEMDETGYLIV---KPGS-------------------TYT
MELRP00403      AIGHQPNTSLFKG-----Q-LEMDETGYIIT---KPGS-------------------THT
ANADF00468      GIGHEPNTGIFKG-----Q-LAMNDVGYLEV---RSPS-------------------TAT
CHLCH01125      AIGHAPNTVLFQD-----Q-LELDDYGYIQT---KKSS-------------------TET
PELPB01419      AIGHAPNSGLFKD-----Q-LDLDDYGYIKT---KKSS-------------------TET
CHLL200781      AIGHAPNAGLFNG-----Q-LAIDDYGYIET---KKSS-------------------TET
CHLTE00830      AIGHEPNAKLFKG-----Q-LDMDDYGYILT---KDHS-------------------TET
CHLP800854      AIGHEPNAKLFQG-----Q-LDMDDYGYILT---KPHS-------------------TET
CHLL701225      AIGHAPNAKMFEG-----Q-LDIDDYGYILT---KKSS-------------------TET
CHLPM00993      AIGHAPNAQMFAG-----Q-IETDDYGYIVT---KKAS-------------------TET
WOLTR00562      AIGHAPNTGVFKS-----F-IEMDEQGYIIT---KPGT-------------------TLT
WOLPP00135      AIGHAPNTGIFKG-----F-VEMDQQGYIIT---KPGT-------------------TLT
WOLPM00650      AIGHAPNTGIFKG-----F-VEMDEQGYIIT---KPGT-------------------TLT
WOLWR00416      AIGHAPNTGIFKG-----F-VEMDEQGYIIT---KPGT-------------------TLT
EHRCR00696      AIGHTPNTQILTTKDNGNI-VDLDNEGYIIT---KPGS-------------------TVT
ANAMM00345      AIGHKPNTQVLADM-PGRH-VELDPDGYVIT---SPDS-------------------TAT
ANAMF00341      AIGHKPNTQVLADM-PGRH-VELDPDGYVIT---SPDS-------------------TAT
ANAPZ00610      AIGHTPNTQVLKNM-LGAK-VNLDDEGYVIT---APGT-------------------TIT
NEOSM00537      AIGHAPNTALFSG-----I-LEMDSAGYIKT---SSSD-------------------TST
NEORI00513      AIGHTPNTALFSG-----I-LEMDSAGYIKT---AGSD-------------------TST
PELUB00076      AIGHDPATALFKE-----Q-LEMDKEGYLLT---KPDS-------------------TET
PELSM00736      AIGHDPATKIFKD-----Q-IKMDTEGYILT---DPDS-------------------TKT
PUNMI01409      AIGHDPATAAFKE-----A-VELDDEGYIIA---EKGG-------------------TRT
ASTEC00651      AIGHAPSSQLFQG-----Q-LETNAGGYLKV---EPGT-------------------PKT
ZYMMT00154      AIGHKPATDLFQG-----H-IKLDNEGYIEV---IPGT-------------------THT
ZYMMO00984      AIGHKPATELFQG-----H-LKLDDEGYIEV---TPGT-------------------TQT
ZYMMA00176      AIGHKPATELFQG-----H-LKLDDEGYIEV---TPGT-------------------TQT
ZYMMN00183      AIGHKPATELFQG-----H-LKLDDEGYIEV---TPGT-------------------TQT
SPHAL00143      AIGHSPSTELFRG-----K-LPLDADGYLQV---TPGT-------------------SLT
SPHWW03978      AIGHSPATELFRG-----H-LELDEDGYLVV---EKGG-------------------TRT
SPHJU02313      AIGHQPATELFVD-----K-LPMD-EGYLLV---EKGT-------------------TKT
NOVAD02319      AIGHAPATELFKG-----K-LELDESGYIVV---QPGT-------------------PKT
ERYLH00934      AIGHAPATELFKG-----K-LPMDETGYLLV---EPGT-------------------PKT
GRABC00720      AIGHSPNTAIFRG-----K-LELDEEGYVVT---RPGT-------------------PCT
GLUDA03075      AIGHAPNTAVFKD-----Q-VETDDEGYIVT---NPGG-------------------TRT
KOMMN00774      AIGHAPTTAIFRD-----I-VEIDTDGYIMT---TPGS-------------------TRT
HALVD01097      AIGHTPNADYLDG-----TGVQRDDDGYIVA---KGGSG---------------GGQTAT
HALHT01699      AIGHTPNTDYLED-----TGVELDDTGYIQT---HGGTG---------------GDQTAT
METI401038      AIGHVPNTQIFEG-----K-LEMDERKLIKV-----FQ------------------GTKT
PLAL201612      AIGHTPNTDFLAG-----Q-IETDDKGYVVY---KTPF------------------RTET
GEMAT01983      AIGHTPNTRFLNG-----Q-LETTEHGYIKV----APW------------------RTAT
CYAAP02456      AIGHTPNTRLFQG-----Q-ITLDEQGYIIT----KGK------------------STAT
CHLPN00303      AIGHKPNTDFLGG-----Q-LTLDESGYIVT----EKG------------------TSKT
CHLPP00439      AIGHKPNTDFLGG-----Q-LTLDESGYIVT----EKG------------------TSKT
CHLPE00727      AIGHKPNTDFLAG-----Q-VNIDESGYILT----NKG------------------TTQT
CHLTR00101      AIGHQPNTAFLGG-----Q-VALDENGYIIT----EKG------------------SSRT
CHLTA00102      AIGHQPNTAFLGG-----Q-VALDENGYIIT----EKG------------------SSRT
CHLTJ00101      AIGHQPNTAFLGG-----Q-VALDENGYIIT----EKG------------------SSRT
```

```
CHLTD00098    AIGHQPNTAFLGG-----Q-VALDENGYIIT----EKG------------------SSRT
CHLT700102    AIGHQPNTAFLGG-----Q-VALDENGYIIT----EKG------------------SSRT
CHLT000101    AIGHQPNTAFLGG-----Q-VALDENGYIIT----EKG------------------SSRT
CHLT500104    AIGHQPNTAFLGG-----Q-VALDENGYIIT----EKG------------------SSRT
CHLTL00098    AIGHQPNTAFLGG-----Q-VALDENGYIIT----EKG------------------SSRT
CHLTG00102    AIGHQPNTAFLGG-----Q-VALDENGYIIT----EKG------------------SSRT
CHLTS00101    AIGHQPNTAFLGG-----Q-VALDENGYIIT----EKG------------------SSRT
CHLT900102    AIGHQPNTAFLGG-----Q-VALDENGYIIT----EKG------------------SSRT
CHLTZ00101    AIGHQPNTAFLGG-----Q-VALDENGYIIT----EKG------------------SSRT
CHLT400432    AIGHQPNTAFLGG-----Q-VALDENGYIIT----EKG------------------SSRT
CHLT100103    AIGHQPNTAFLGG-----Q-VALDENGYIIT----EKG------------------SSRT
CHLT200350    AIGHQPNTAFLGG-----Q-VALDENGYIIT----EKG------------------SSRT
CHLTB00350    AIGHQPNTAFLGG-----Q-VALDENGYIIT----EKG------------------SSRT
CHLTC00378    AIGHQPNTAFLGG-----Q-VALDENGYIIT----EKG------------------SSRT
SIMNZ00831    AIGHQPNTTFLKG-----Q-LETDSHGYLLV----KPS------------------TTLT
PARUW01706    AIGHNPNTEFLKN-----Q-LELHSNGYLKV----FKG------------------TQT-
WADCW00943    AIGHLPNTSFLNE-----Q-IELHDNGYIKV----SPG------------------TTRT
SINAD04980    AIGHTPNSTIFRE-----K-LAMTPDGYILT---PTALAWKGTPAPEGLRDQLVNFGTAT
BIFLB01558    AIGHVPQTGFLGG-----Q-LDLDDHGYIRI---DETS----------------TGATRT
BIFAB00498    AIGHVPQTGFLGG-----Q-LDLDDHGYIRI---DETS----------------TGATRT
BIFAV01561    AIGHVPQTGFLGG-----Q-LDLDDHGYIRI---DETS----------------TGATRT
BIFAS01557    AIGHVPQTGFLGG-----Q-LDLDDHGYIRI---DETS----------------TGATRT
BIFA001518    AIGHVPQTGFLGG-----Q-LDLDDHGYIRI---DETS----------------TGATRT
BIFBA01814    AIGHTPATEFIAD-----V-VSTDDDGYITV---AEAG-------------------THT
BIFAA01619    AIGHTPATAFLNG-----L-VDVDSAGYITV---DGAS-------------------TRT
BIFDB02112    AIGHTPATTFLDG-----V-VNRDEAGYIQV---EGAS-------------------TRT
TERSS03091    GIGHIPNAKMFAG-----Q-MDLDEDGYILS---KEQVF---------------CTHNGE
GRATM03160    GIGHEPNAKAFAG-----M-MDLDDDGYILC---KDDVY---------------CTKNGE
GRAMM03836    GIGHVPNASMFKG-----Q-IDLDGDGYILT---EHNVF---------------CTNQGI
AKKM801247    AIGHTPNTSFLGD-----L-VDRDDAGYIIR---ETGM-------------------MAT
OPITP04130    AIGHVPNSGPFAE-----A-LDVDEGGYFKP---SVGS------------------QVKT
CORAD01794    AIGHIPNTDFAEG-----I-LERDAEGYIVP---VLGS------------------QVLT
BUCCC00180    AIGHIPNSQIFSK-----Y-IDIKNNYVKINYKNTNMK-------------------TQT
CENSY00347    AIGHDPNTELFKG-----Q-LETDDQGYIVL---KG-A-------------------SQT
NITMS00668    AIGHEPNTKLFKN-----Q-IDLDDEGYVVL---KN-K-------------------THT
MEIRD02823    FIGHEPNTGFLQG-----V-VELRPDGYVAV---RD-E-------------------IFT
HERA203780    YIGHIPNTWLFKD-----Q-IELDENGYIVS---PG-R-------------------ART
CHLAA02018    YIGHVPNTDLFRG-----I-LELDEGGYIVT---DG-R-------------------TRT
CHLSY02175    YIGHVPNTDLFRG-----I-LELDEGGYIVT---DG-R-------------------TRT
CHLAD02599    YIGHVPNTELFRG-----I-LELDEGGYIVT---DG-R-------------------TRT
CALAS01745    FIGHLPNNDLFHG-----K-LEMDEDGYLIT---DR-K-------------------MRT
ANATU01696    FIGHKPNTQLFHG-----Q-LEMDEGGYIIT---DK-K-------------------MQT
SULMS00235    AIGNSPNTNIFKN-----K-IYLDKNGYILT---KKNS-------------------TKT
BLASB00041    AIGHIPNTEIFKN-----K-LDLDDRGYVIV---KKGS-------------------TMT
BLASP00551    AIGHSPNTKLFKN-----Q-LNLDERGYIIV---EKGS-------------------TST
AZOPC00437    AIGRKPNSEIFRT-----Y-ISTDSNGYIKT---QHGT-------------------TKT
LEPBD01878    AIGRTPNTKFLEG-----K-VEIDERGYIVT---KGKS-------------------SKT
SALRD02577    AIGHTPNTGPFEG-----W-VQMDETGYIQT---EGAS-------------------TYT
SALRM02866    AIGHTPNTGPFEG-----W-VQMDETGYIQT---EGAS-------------------TYT
RIEPU00166    AVGSNPNTSIFQG-----Q-LDLQD-GYIKTSLKSRCM-------------------TET
ORITB00015    AIGHAPNTSLFTN-----Q-LDMDSYGYII-TK--PNS-------------------TQT
ORITI01234    AIGHAPNTSLFTN-----Q-LDMDSYGYII-TK--PNS-------------------TQT
PREMB01541    AIGHTPNTDLFKG-----Q-LEMDDHGFII-TK--PKS-------------------TAT
PREDF01155    AIGHTPNTELFKG-----Q-LEMDDHGFIV-TR--PKS-------------------TAT
PREI702011    AIGHLPNTNLFKG-----Q-LELDPQGFIV-TK--GTS-------------------TAT
ALIFI01035    AIGHHPNTELFAD-----Q-LTLDAEGYIK-TE--AGT-------------------SKT
ODOSD00362    AIGHHPNSEVFSK-----Y-VKVDEQGYII-TE--GAS-------------------TRT
PRER201250    AIGHKPNTDIFKE-----W-LDMDETGYLK-KI--DGT-------------------PKT
PALPW00206    AIGHKPNSDIFKP-----W-IETDEVGYIK-TI--PGT-------------------PRT
PORGI00953    AIGHTPNSKIFAD-----Y-LDLDEVGYIL-TE--GSS-------------------PRT
PORG301168    AIGHTPNSKIFAD-----Y-LDLDEVGYIL-TE--GSS-------------------PRT
BACV803426    AIGHKPNSDIFKD-----Y-LDTDEVGYII-TE--AGT-------------------PRT
BACT601486    AIGHKPNSDIFKP-----Y-IDTDEVGYII-TE--PDT-------------------PRT
BACTN04290    AIGHKPNSDIFKE-----Y-IDTDEVGYII-TD--GDS-------------------PRT
BACFR01032    AIGHKPNSDIFKP-----Y-LDTDEVGYIT-TD--GDS-------------------PRT
```

```
BACFN00913     AIGHKPNSDIFKP-----Y-LDTDEVGYIT-TD--GDS-------------------PRT
BACF600963     AIGHKPNSDIFKP-----Y-LDTDEVGYIT-TD--GDS-------------------PRT
OWEHD03413     AIGHKPNTDIFKG-----Q-LDMDSTGYLI-TK--PGT-------------------ATT
PSYTT00390     AIGHKPNTDMFKG-----Y-LDMDEIGYII-TK--PKS-------------------TKT
NONDD00011     AIGHKPNTDIFKG-----Q-LEMGEDGYLK-TV--SGS-------------------TKT
ROBBH02711     AIGHKPNTDIFKG-----Q-LDMDATGYIV-TH--GKS-------------------TRT
CELAD02592     AIGHKPNTDIFKG-----Q-LDMDETGYLI-TK--GKS-------------------TKT
CELLC02474     AIGHKPNTDIFKG-----Q-LDMDETGYLI-TE--GKS-------------------TKT
MARSH03072     AIGHKPNTDIFKG-----K-IDMDDTGYIV-TK--GKS-------------------TKT
MURRD00159     AIGHKPNTDIFKG-----Q-LDMDETGYII-TQ--PKS-------------------TKT
ZOBGA00152     AIGHKPNTDIFKG-----Q-LDMDETGYII-TK--GKS-------------------TKT
GRAFK00949     AIGHKPNTDIFKG-----W-LDMDDTGYVI-TQ--AKS-------------------TRT
ZUNPS02344     AIGHKPNTDIFKG-----H-LDMDDTGYLI-TK--GKS-------------------TKT
AEQSU02079     AIGHKPNTDIFKG-----Q-LEMDDTGYLI-TQ--GKS-------------------TKT
HALH105745     AIGHQPNTDIFKG-----W-LDMDETGYLK-TV--PGR-------------------TLT
SAPGL02893     AIGHKPNTDIFKE-----W-LPTDETGYLIVDQ--PGT-------------------SKT
AMOA500014     AIGHQPNTALFAP-----Y-ITLDNKGYIQ-TT--PGS-------------------TKT
FLAIG01802     AIGHKPNTDIFAD-----F-IDLDETGYIK-NV--PGT-------------------SKT
FLACA02326     AIGHKPNTDIFKD-----Y-ITLDETGYIL-NI--PGT-------------------SKT
FLAJ100198     AIGHKPNTDIFKD-----Y-ITLDETGYII-NT--PGT-------------------SKT
FLABF01528     AIGHQPNTAIFKD-----F-IDLDETGYIK-NI--AGS-------------------SKT
CAPOD01333     AIGHKPNTDVFKP-----Y-IDTDEVGYIK-NV--AGT-------------------SLT
CAPCC00414     AIGHKPNTDIFKP-----F-IETDEVGYIK-NV--AGT-------------------SLT
FLELS01898     AIGHKPNTDIFIG-----Q-LDMDESNYLI-TQ--DNS-------------------SLT
SOLCM01166     AIGHKPNTDVFKG-----W-LDMDENGYII-TQ--PGS-------------------TKT
PEDHD01598     AIGHKPNTDIFKG-----W-LEMDDTGYLK-TI--PGS-------------------TKT
SPHS203207     AIGHKPNTDIFKG-----W-LDMDESGYLI-TK--ADS-------------------TQT
LEAB401188     AIGHQPNTQIFQQ-----Y-LQLDETGYIL-VE--KGT-------------------TKT
EMTOG00092     AIGHQPNTQIFAD-----F-IKLDTEGYIE-TE--KGS-------------------SRT
SPILD01737     AIGHKPNTDIFQD-----Y-LDLDENGYII-TE--KGS-------------------TRT
DYAFD00084     AIGHKPNTDIFKG-----Y-VNMDETGYIQ-TI--KGS-------------------SCT
CYCMS03063     AIGHQPNTAVFEN-----Q-IDTDENGYIL-TQ--PGS-------------------TKT
ECHVK03326     AIGHKPNTDIFKD-----F-LDMNEAGYIN-TQ--PGS-------------------TKT
BELBD02542     AIGHEPNTAIFKD-----F-INMDPSGYIK-TI--PGT-------------------TKT
NITGG02112     AIGHEPNTSIFKG-----Q-LELDDKGYVI-LK--EH--------------------TKT
THEM700111     AIGHKPNTEFLRG-----H-LDLDERGYII-A---DG--------------------PRT
CREAS01350     AIGYEPNTEIFKG-----Q-LEMDEKGYIK-VY--DQ--------------------TKT
TURPD02772     AIGHVPNTALFKG-----Q-LNMDETGYLV-TQ--PDR-------------------TAT
SORC507603     AIGHTPMTDLFVG-----Q-LDTHPNGYLK-TV--PGS-------------------TRT
BDEBA00337     AIGHKPNTDLFKG-----V-LDMNETGYLI-TQ--PNT-------------------TYT
STIAD02752     AIGHTPNTHLFQG-----V-LETHPSGYLK-TV--PGS-------------------TRT
MYXXD01876     AIGHTPNTELFQG-----V-LETHQGGYLK-TV--PGS-------------------TRT
MYXFH03454     AIGHTPNTELFQG-----V-LETHQGGYLK-TV--PGS-------------------TRT
CORCM01960     AIGHTPNTELFQG-----I-LETHQGGYLK-TI--PGS-------------------TRT
MYXSD02209     AIGHTPNTELFQG-----I-LETHQSGYLK-TV--PGS-------------------TRT
LEPFC02126     GIGHTPNSHFVKG-----L-CDMDDVGYIR-TH--DG--------------------TKT
LEPFM02294     GIGHTPNSRFLDG-----V-VERDSNGYIK-TF--TG--------------------SRT
SOLUE00582     AIGHIPNTKVFKG-----Q-IETDPDGYIL-SK--GG--------------------ART
KORVE01333     GIGHHPNATAFGD-----Q-LDKDADGYLI-TK--DY--------------------VFT
ACIC502553     GIGHEPNAGMFAG-----Q-IDLDDDGYIR-TH--DQ--------------------VHT
ACIFD00030     AIGHAPSTEIVKG-----Q-LEMDDAGYLI-TK--PGS-------------------TET
HALMS01402     GIGHSPNTGFLNG-----Q-IDLDDHGFII-TK--GAH-------------------PDT
CHLPD01133     AIGHAPNAELFKG-----Q-LRIDDYGYIE-TK--KTS-------------------TET
CHLPB01137     AIGHTPNSSIFER-----Q-LDIDEYGYIK-TE--KSS-------------------TET
PROA200897     AIGHSPNSGLFEG-----Q-LDIDEYGYIE-TA--GSS-------------------SVT
WIGBR00492     AIGHIPNTKIFEN-----Q-ITLK-NYYICIS---NK-Y------------NK-KYRTST
BUCA500289     AIGYTPNTNIFVN-----K-LKMK-DGYIQVT---RQ-E------------HG-NY-TQT
BUCAI00292     AIGYTPNTNIFVN-----K-LKMK-DGYIQVT---RQ-E------------HG-NY-TQT
BUCAF00306     AIGYTPNTNIFVN-----K-LKMK-DGYIQVT---RQ-E------------HG-NY-TQT
BUCAT00287     AIGYTPNTNIFVN-----K-LKMK-DGYIQVT---RQ-E------------HG-NY-TQT
BUCA000302     AIGYTPNTNIFVN-----K-LKMK-DGYIQVT---RQ-E------------HG-NY-TQT
BAUCH00290     AIGHSPNTTIFEN-----Q-LVLQ-NGYIKVQ---AG-I------------NG--NATAT
BLOVB00369     AIGYNPNTSIFCH-----Q-LKLNDNGYICVN---SK-N------------DNNNATTAT
BLOFL00367     AIGHDPNTAIFNN-----Q-LTLNNHGYIHVK---SG-L------------DG--SFTAT
BLOPB00375     AIGYNPNTSIFND-----Q-LVLN-NGYICVH---SG-M------------NG--NATAT
```

```
BUCAP00289    AIGYIPNTDIFTD-----Q-LKMKD-GYIKIK---KG-T------------HG--NYTQT
COXBU01001    AIGHDPNTKIFKE-----Q-LEMDEAGYLRAK---SG-L------------QG--NATAT
COXBN01188    AIGHDPNTKIFKE-----Q-LEMDEAGYLRAK---SG-L------------QG--NATAT
COXBR01117    AIGHDPNTKIFKE-----Q-LEMDEAGYLRAK---SG-L------------QG--NATAT
COXB200717    AIGHDPNTKIFKE-----Q-LEMDEAGYLRAK---SG-L------------QG--NATAT
COXB100914    AIGHDPNTKIFKE-----Q-LEMDEAGYLRAK---SG-L------------QG--NATAT
FRAP200241    AIGHTPNTGIFKD-----Q-LEME-NGYIKVK---SG-L------------AG--DATQT
FRANT01012    AIGHTPSTDIFKD-----Q-IEME-NGYIKVK---SG-L------------AG--DATQT
FRACN00522    AIGHTPNTSIFAG-----Q-LEME-NGYIKVK---SG-L------------TG--DATQT
FRATT00465    AIGHTPNTSIFAG-----Q-LEME-NGYIKVK---SG-L------------AG--DATQT
FRAT100465    AIGHTPNTSIFAG-----Q-LEME-NGYIKVK---SG-L------------AG--DATQT
FRATE00462    AIGHTPNTSIFAG-----Q-LEME-NGYIKVK---SG-L------------AG--DATQT
FRATW01216    AIGHTPNTSIFAG-----Q-LEME-NGYIKVK---SG-L------------AG--DATQT
FRATM01110    AIGHTPNTSIFAG-----Q-LEME-NGYIKVK---SG-L------------AG--DATQT
FRACF00566    AIGHTPNTSIFAG-----Q-LEME-NGYIKVK---SG-L------------AG--DATQT
FRATO01212    AIGHTPNTSIFAG-----Q-LEME-NGYIKVK---SG-L------------AG--DATQT
FRATH01486    AIGHTPNTSIFAG-----Q-LEME-NGYIKVK---SG-L------------AG--DATQT
FRATF01216    AIGHTPNTSIFAG-----Q-LEME-NGYIKVK---SG-L------------AG--DATQT
FRATN00564    AIGHTPNTSIFAG-----Q-LEME-NGYIKVK---SG-L------------AG--DATQT
ACIF500512    AIGHQPNTGIFKG-----Q-LKMDEGGYLVTR---GG-R------------DG--MATAT
ACIF200362    AIGHQPNTGIFKG-----Q-LKMDEGGYLVTR---GG-R------------DG--MATAT
DECAR01284    AIGHKPNTDIFAG-----Q-LEMDN-GYLVTQ---AG-R------------NG--NATQT
NEIG100525    AIGHKPNTDIFKG-----Q-LEMDEAGYLKTK---GG-T------------AD--NVGAT
NEIG201314    AIGHKPNTDIFKG-----Q-LEMDEAGYLKTK---GG-T------------AD--NVGAT
NEIM800926    AIGHKPNTDIFKG-----Q-LETDEAGYLKTK---GG-T------------AD--NVGAT
NEIMP01199    AIGHKPNTDIFKG-----Q-LETDEAGYLKTK---GG-T------------AD--NVGAT
NEIMB01212    AIGHKPNTDIFKG-----Q-LEMDEAGYLKTK---GG-T------------AD--NVGAT
NEIMF01143    AIGHKPNTDIFKG-----Q-LEMDEAGYLKTK---GG-T------------AD--NVGAT
NEIML01119    AIGHKPNTDIFKG-----Q-LEMDEAGYLKTK---GG-T------------AD--NVGAT
NEIMM00780    AIGHKPNTDIFKG-----Q-LEMDEAGYLKTK---GG-T------------AD--NVGAT
NEIMH00827    AIGHKPNTDIFKG-----Q-LEMDEAGYLKTK---GG-T------------AD--NVGAT
NEIMG01168    AIGHKPNTDIFKG-----Q-LEMDEAGYLKTK---GG-T------------AD--NVGAT
NEIMN01254    AIGHKPNTDIFKG-----Q-LEMDEAGYLKTK---GG-T------------AD--NVGAT
NEIMO00810    AIGHKPNTDIFKG-----Q-LEMDEAGYLKTK---GG-T------------AD--NVGAT
NEIM701229    AIGHKPNTDIFKG-----Q-LEMDEAGYLKTK---GG-T------------AD--NVGAT
NEIMA01334    AIGHKPNTDIFKG-----Q-LEMDEAGYLKTK---GG-T------------AD--NVGAT
NEIMW01125    AIGHKPNTDIFKG-----Q-LEMDEAGYLKTK---GG-T------------AD--NVGAT
DICNV01037    AIGHQPNTELFKN-----Q-LATDAHHYLSVH---SG-S------------NG--NATQT
VESOH00047    AIGHTPNTAIFKG-----H-LEMIN-DYIKVQ---SG-T------------QG--NATQT
RUTMC00043    AIGHTPNTAIFEG-----H-LEMSH-GYIKVQ---SG-T------------QG--NAMQT
HALHL02255    AIGHSPNTGLFEG-----Q-LAMSS-GYIRVN---SG-L------------DG--NATAT
PELPD03177    AVGHSPNTKIFEG-----Q-LEMDN-GYIRTK---CASV------------EG--NMTAT
GEOS804030    AIGHQPNTRLFEG-----Q-LEMDE-GYIRTN---CG-Y------------EG--NSTAT
GEOBB03636    AIGHQPNTHIFDG-----Q-LEMDE-GYIRTN---CG-Y------------EG--NSTST
GEOSM03700    AIGHQPNTHIFDG-----Q-LEMDE-GYIRTN---CG-Y------------EG--NSTST
HALNC00046    AIGHAPNTGIFEG-----E-LDLA-GGYIQVK---SG-T------------QG--NATAT
HAEPS00887    AIGHSPNTEIFKD-----Q-LELN-NGYIVVK---SG-L------------DG--NATAT
GALAU02285    AIGHAPNTDIFAG-----Q-LALN-NGYIVVK---SG-L------------QG--NATAT
HISS201000    AIGHAPNTELFAN-----Q-LELN-NGYIVVK---SG-L------------EG--NATAT
HAES101151    AIGHAPNTELFAN-----Q-LELN-NGYIVVK---SG-L------------EG--NATAT
PASMU00573    AIGHAPNTDIFAE-----Q-LELN-NGYIVVK---SG-L------------EG--NATAT
PASMH00579    AIGHAPNTDIFAE-----Q-LELN-NGYIVVK---SG-L------------EG--NATAT
ACTSZ01560    AIGHAPNTEIFQG-----Q-LELN-NGYIVVK---SG-L------------EG--NATAT
AGGAN00985    AIGHAPNTEIFQG-----Q-LELN-NGYIVVK---SG-L------------EG--NATAT
NITHN03181    AIGHKPNTDIFTG-----Q-LEME-HGYILVR---GG-A------------KG--FATQT
NITOC00311    AIGHKPNTDIFTG-----Q-LEME-HGYIQVR---GG-F------------KG--FATQT
NITWC02498    AIGHKPNTDIFTG-----Q-LAME-QGYIQVR---GG-F------------KG--FATQT
METNJ00648    AIGHQPNTWMFKG-----Q-LEMQ-HDYIILK---KG--------------------TQT
METFJ01616    AIGHQPNTSMFKE-----Q-LDMQ-HDYIILK---KG--------------------QQT
ALKEH00244    AIGHAPNTGLFQD-----Q-LEMN-GGYIVVK---SG-L------------NG--NATAT
MARMS03166    AIGHKPNTDIFQG-----Q-LDMK-DGYLKVQ---SG-T------------HG--NATQT
MARM102688    AIGHKPNTDIFEG-----Q-LEMK-DGYIKVQ---SG-L------------EG--NATQT
THICR00763    AIGHTPNTGIFDG-----Q-LDMD-HGYLKVQ---SG-L------------QG--NATQT
THICA00974    AIGHTPNTDIFAG-----Q-LEMI-NGYIKVK---SG-L------------EG--NATAT
THIV600851    AIGHKPNTQIFEG-----Q-VDMA-GGYIKVQ---SG-T------------RG--NATAT
```

```
THISH01973    AIGHKPNTAIFDG-----Q-LEME-NGYIKVK---SG-T------------GG--NATAT
METAA00813    AIGHTPNTDIFQG-----Q-LEME-HGYIKVK---TG-L------------QG--NATAT
META200813    AIGHTPNTDIFQG-----Q-LEME-HGYIKVK---TG-L------------QG--NATAT
METMM02895    AIGHTPNTDIFTG-----Q-LDMQ-HGYIVVN---SG-I------------QG--NATAT
FRAAD00813    AIGHTPNTGIFKD-----Q-LEMT-DGYLKIR---SG-L------------EG--LATMT
XYLFA01416    AIGHHPNTDLFAG-----Q-LAMN-NGYLQIH---SG-T------------AG--NVTQT
XYLFT00623    AIGHHPNTDLFAG-----Q-LAMN-NGYLQIH---SG-T------------AG--NVTQT
XYLF200667    AIGHHPNTDLFAG-----Q-LAMN-NGYLQIH---SG-T------------AG--NVTQT
XYLFG01622    AIGHHPNTDLFAG-----Q-LAMN-NGYLQIH---SG-T------------AG--NVTQT
XYLFM00690    AIGHHPNTDLFAG-----Q-LAMN-NGYLQIH---SG-T------------AG--NVTQT
PSEUP01680    AIGHTPNTSLFDG-----Q-LAMN-NGYLIIK---SG-L------------DG--NATQT
STRM501972    AIGHHPNTQLFDG-----Q-LAMN-NGYLEIR---SG-L------------GG--NATQT
PSEUU01524    AIGHQPNTGLFDG-----Q-LAMD-NGYITIR---SG-L------------GG--NATAT
XANAP01407    AIGHHPNTQLFDG-----Q-LTMH-NGYLEIR---SG-L------------GG--GATET
XANCP01918    AIGHHPNTQLFDG-----Q-LAMN-NGYLEIR---SG-L------------NG--AATET
XANC802154    AIGHHPNTQLFDG-----Q-LAMN-NGYLEIR---SG-L------------NG--AATET
XANCB02263    AIGHHPNTQLFDG-----Q-LAMN-NGYLEIR---SG-L------------NG--AATET
XANOR02379    AIGHHPNTQLFDG-----Q-LAMN-NGYLEIR---SG-L------------NG--AATET
XANOM02295    AIGHHPNTQLFDG-----Q-LAMN-NGYLEIR---SG-L------------NG--AATET
XANOP02318    AIGHHPNTQLFDG-----Q-LAMN-NGYLEIR---SG-L------------NG--AATET
XANAC01951    AIGHHPNTQLFDG-----Q-LAMN-NGYLEIR---SG-L------------NG--AATET
CYCSP01155    AIGHSPNTQIFDG-----Q-LDMS-HGYLHTE---RG-S------------SG--NATQT
GEOLS03275    AIGHSPNTAIFDG-----Q-LEMK-DGYLRTK---GG-S------------DG--FATQT
GEOUR03858    AIGHQPNTDIFEG-----Q-LEMD-NGYIRTQ---CG-N------------EG--NMTAT
GEODF00692    AIGHTPNTQMFEG-----Q-LDME-GGYIRTL---CG-T------------DG--NMTAT
GEOSL00482    AIGHTPNTHLFEG-----Q-LEMD-EGYIRTQ---CG-A------------EG--NFTAT
GEOSK00469    AIGHTPNTHLFEG-----Q-LEMD-EGYIRTQ---CG-A------------EG--NFTAT
GEOMG02998    AIGHAPNTQLFEG-----Q-LEMD-NGYIRTL---CG-G------------EG--NVTAT
MORCR00228    AIGHKPNTKLFDG-----Q-LNMQ-DGYIVVN---SG-L------------NG--NATAT
ACIAD00798    AIGHKPNTEMFKD-----Q-LELR-DGYIQVQ---SG-T------------KG--NATAT
ACIBC00821    AIGHKPNTSMFEG-----Q-LNLR-DGYIQVQ---SG-T------------SG--NATAT
ACIBY02725    AIGHKPNTSMFEG-----Q-LNLR-DGYIQVQ---SG-T------------SG--NATAT
ACIB302691    AIGHKPNTSMFEG-----Q-LNLR-DGYIQVQ---SG-T------------SG--NATAT
ACIB500882    AIGHKPNTSMFEG-----Q-LNLR-DGYIQVQ---SG-T------------SG--NATAT
ACIB100850    AIGHKPNTSMFEG-----Q-LNLR-DGYIQVQ---SG-T------------SG--NATAT
ACIBD00843    AIGHKPNTSMFEG-----Q-LNLR-DGYIQVQ---SG-T------------SG--NATAT
ACIBS02229    AIGHKPNTSMFEG-----Q-LNLR-DGYIQVQ---SG-T------------SG--NATAT
ACICP00137    AIGHKPNTGMFEG-----Q-LNLR-DGYIQVQ---SG-T------------SG--NATAT
ACISD03052    AIGHKPNTGMFDG-----Q-LNLR-DGYIQVQ---SG-T------------SG--NATAT
LEGLN02402    AIGHTPNTDLFKD-----Q-LIMN-NGYIVIK---SG-L------------DG--MATST
LEGPA01726    AIGHTPNTAIFQD-----Q-LAMR-DGYILIK---SG-L------------DG--MATST
LEGPH00846    AIGHTPNTAIFQD-----Q-LAMR-DGYILIK---SG-L------------DG--MATST
LEGPC01171    AIGHTPNTAIFQD-----Q-LAMR-DGYILIK---SG-L------------DG--MATST
LEGP201857    AIGHTPNTAIFQD-----Q-LAMR-DGYILIK---SG-L------------DG--MATST
LEGPL01727    AIGHTPNTAIFQD-----Q-LAMR-DGYILIK---SG-L------------DG--MATST
COLP302668    AIGHKPNTDIFKD-----Q-LDMK-DGYLTIQ---SG-T------------QG--NATQT
KANKD01106    AIGHKPNTDIFEG-----Q-LEMK-DGYIVVK---SG-L------------AG--NATAT
IDILO00659    AIGHQPNTGIFDG-----E-LEMQ-DGYIQVQ---SG-L------------QG--NATAT
PSEU901284    AIGHKPNTDMFEG-----Q-LEMK-DGYLVVE---SG-L------------NG--NATQT
PSEA602346    AIGHQPNTGIFEG-----Q-LEMK-DGYIKVN---SG-T------------AG--NATQT
ALTSS01871    AIGHKPNTDIFAE-----Q-LEMK-DGYIVVN---SG-L------------HG--NATQT
ALTMD01611    AIGHKPNTDIFDG-----Q-LEMK-DGYITVN---SG-T------------NG--NATQT
ALTME01705    AIGHKPNTDIFDG-----Q-LEMK-DGYIVVN---SG-T------------NG--NATQT
ALTMB01797    AIGHKPNTDIFDG-----Q-LEMK-DGYIVVN---SG-T------------NG--NATQT
ALTMS01652    AIGHKPNTDIFDG-----Q-LEMK-DGYIVVN---SG-T------------NG--NATQT
SACD201683    AIGHKPNTDIFQG-----Q-LEMK-DGYIVIK---SG-L------------NG--EATST
TERTT01748    AIGHKPNTDIFEG-----Q-LDMH-NGYIKIK---SG-L------------EG--AATST
SIMAS00609    AIGHKPNTDIFAG-----Q-LEMK-DGYIVVK---SG-L------------HG--NATST
ALCDB01962    AIGHKPNTDIFAG-----Q-LDMH-DGYIRIR---SG-L------------EG--NATAT
CHRSD02939    AIGHSPNTGIFEG-----Q-LEMN-NGYIRVK---SG-L------------EG--NATMT
HALED02811    AIGHSPNTGIFEG-----Q-LDMA-GGYIKVQ---SG-L------------EG--NATAT
SIDLE02655    AIGHKPNTDIFAG-----Q-LEMD-NGYIVTR---GG-R------------LG--NATAT
GALCS01621    AIGHKPNTEIFAG-----Q-LEMN-NGYISTR---GG-S------------TG--NATAA
LARHH01446    AIGHKPNTDLFKG-----Q-LDMDETGYLITR---GG-R------------DG--NATAT
CHRVO02807    AIGHKPNTDIFKG-----Q-LEMDSTGYLITK---GG-R------------DG--NATLT
```

```
PSEUL01648    AIGHKPNTDIFKG-----Q-LEMDSTGYIITK---GG-R------------EG--NATAT
NITEU01859    AIGHHPNTDLFQG-----Q-LEMK-NGYIITQ---GG-N------------EG--NATAT
NITEC00745    AIGHHPNTDLFQG-----Q-LEMK-NGYIITQ---GG-N------------EG--NATAT
NITMU00023    AIGHKPNTDIFQG-----Q-LKME-NGYIMTQ---AG-I------------HG--NATAT
NITSI03064    AIGHKPNTDIFTG-----Q-LQME-NGYIVTH---SG-G------------QG--NATAT
ACCPU02128    AIGHKPNTDLFAG-----Q-LAME-GGYLLTQ---GG-R------------HG--NATAT
THIDA01000    AIGHKPNTGIFEG-----Q-LELE-GGYIVTR---GG-N------------KG--NATAT
METS601221    AIGHKPNTDIFAG-----Q-LEME-GGYIVTK---AG-R------------NG--NATAT
METGS01186    AIGHKPNTDIFAG-----Q-LEME-GGYIVTK---AG-R------------NG--NATAT
METFK00972    AIGHKPNTDIFEG-----Q-LEME-GGYIVTK---AG-R------------EG--NFTAT
METML01428    AIGHKPNTDIFAG-----Q-LEME-GGYIVTQ---MG-R------------AG--NATAT
AROAE03899    AIGHKPNTDIFEG-----Q-LEME-NGYIITK---GG-R------------EG--DATAT
THASP01686    AIGHKPNTDIFEG-----Q-LQME-GGYIVTQ---GG-R------------NG--NATQT
AZOSB01359    AIGHKPNTDIFKG-----Q-LEMDETGYLITQ---GG-R------------NG--NATQT
BORA102682    AIGHQPNTGIFEG-----Q-LDMK-DGYIVTK---SG-L------------NG--LATMT
BORPA03415    AIGHQPNTGIFEG-----Q-LEMK-DGYIITK---SG-L------------SG--MATMT
BORBM03577    AIGHQPNTGIFEG-----Q-LEMK-DGYIITK---SG-L------------SG--MATMT
BORPE02280    AIGHQPNTGIFEG-----Q-LEMK-DGYIITK---SG-L------------SG--MATMT
BORPC02060    AIGHQPNTGIFEG-----Q-LEMK-DGYIITK---SG-L------------SG--MATMT
BORP102392    AIGHQPNTGIFEG-----Q-LEMK-DGYIITK---SG-L------------SG--MATMT
BORBR03870    AIGHQPNTGIFEG-----Q-LEMK-DGYIITK---SG-L------------SG--MATMT
BORPD01524    AIGHQPNTGIFEG-----Q-LDMK-DGYIVTK---SG-L------------SG--MATMT
ACHXA01179    AIGHQPNTEIFQG-----Q-LEMK-DGYIVTK---SG-L------------SG--MATMT
RHOFT03114    AIGHTPNTEIFKG-----Q-LDME-GGYIITQ---GG-N------------KG--FATQT
VEREI01701    AIGHSPNTDLFQG-----Q-LAMD-NGYLITQ---GG-L------------NG--MATQT
VARPE01375    AIGHHPNTDIFQG-----Q-LEMK-DNYILTR---SG-L------------QG--FATMT
VARPS01266    AIGHHPNTDIFQG-----Q-LEMK-DNYILTR---SG-L------------QG--FATMT
DELAS05272    AIGHAPNTEIFKG-----H-LEMDETGYIITQ---GG-L------------KG--FATQT
DELSC01235    AIGHAPNTEIFKG-----H-LEMDETGYIITQ---GG-L------------KG--FATQT
COMT200883    AIGHHPNTDIFQG-----Q-LELE-NGYIITQ---GG-L------------KG--FATQT
ACIAC03267    AIGHAPNTGIFEG-----Q-LTLE-NGYIVTQ---GG-L------------KG--FATQT
ACIAP03249    AIGHAPNTGIFEG-----Q-LTLE-NGYIVTQ---GG-L------------KG--FATQT
ACIET02648    AIGHAPNTDIFQG-----Q-LAME-NGYIVTQ---GG-L------------KG--FATQT
ALIDK03528    AIGHAPNTDIFQG-----Q-LAME-NGYLITQ---GG-L------------KG--FATQT
RUBGI03408    AIGHQPNTEIFKG-----Q-LEMK-DGYVLTK---GG-S------------EG--FATMT
LEPCP00749    AIGHQPNTDIFKG-----Q-LEMK-DGYIVTR---TG-L------------NG--FATMT
POLSJ03719    AIGHQPNTEIFAG-----Q-LEMK-DGYIITK---TG-L------------QG--FATMT
POLNA03144    AIGHQPNTDIFAG-----Q-LEMK-GGYIITQ---TG-L------------QG--MATMT
METPP01110    AIGHRPNTEIFQG-----Q-LEMK-DGYIITR---TG-L------------NG--FATMT
RAMTT03218    AIGHSPNTEIFKG-----Q-LEMK-DGYILTK---SG-L------------NG--FATMT
RALPJ02457    AIGHKPNTDIFRG-----Q-LDMNDTGYIRTK---SG-L------------DG--MATAT
RALP102102    AIGHKPNTDIFRG-----Q-LDMNDTGYIRTK---SG-L------------DG--MATAT
RALSO02303    AIGHKPNTDLFKG-----Q-LDMNETGYLRTQ---SG-L------------VG--NATAT
RALS801097    AIGHKPNTDLFKG-----Q-LDMNETGYLRTQ---SG-L------------TG--NATAT
HERSS01878    AIGHKPNTSIFEG-----Q-LSMQ-NGYLKTR---TG-T------------EG--FATAT
HERAR00955    AIGHKPNTSIFEG-----Q-LDMH-NGYIKTK---TG-L------------EG--MATST
JANMA01152    AIGHKPNTSIFEG-----Q-LEMN-NGYIKTK---TG-L------------EG--MATST
THIK102324    AIGHQPNTEIFKG-----Q-LDMK-DGYIITR---SG-N------------EG--MATAT
POLSQ00682    AIGHKPNTELFVG-----Q-LDMH-NGYLKTH---SG-L------------EG--NATAT
POLNS00989    AIGHKPNTELFVG-----Q-LDMN-NGYLKTH---SG-L------------EG--NATAT
BURP800627    AIGHKPNTDLFEG-----Q-LEMK-NGYIITN---GG-L------------NG--NATGT
BURPP00988    AIGHKPNTDIFAG-----Q-LEMK-NGYIITK---GG-L------------NG--FATAT
BURSC00728    AIGHKPNTDIFEG-----Q-LEMK-NGYIITK---GG-L------------NG--FATAT
BURXL00982    AIGHKPNTDIFAG-----Q-LEMK-NGYIITK---GG-L------------NG--FATAT
BURSG00869    AIGHKPNTDIFEG-----Q-LEMK-NGYIITK---GG-L------------NG--FATAT
BURRH00629    AIGHKPNTDIFEG-----Q-LEMR-NGYIVTN---AG-L------------NG--NATAT
BURGB00781    AIGHKPNTDIFQG-----Q-LEMK-DGYIVTK---SG-L------------AG--NATAT
BURGS00853    AIGHKPNTDLFQG-----Q-LEMK-DGYIVTK---SG-L------------AG--NATAT
BURPS02618    AIGHKPNTDIFKG-----Q-LEMK-DGYIITN---SG-L------------SG--NATGT
BURMA01741    AIGHKPNTDIFKG-----Q-LEMK-DGYIITN---SG-L------------SG--NATGT
BURP103016    AIGHKPNTDIFKG-----Q-LEMK-DGYIITN---SG-L------------SG--NATGT
BURP002984    AIGHKPNTDIFKG-----Q-LEMK-DGYIITN---SG-L------------SG--NATGT
BURM701866    AIGHKPNTDIFKG-----Q-LEMK-DGYIITN---SG-L------------SG--NATGT
BURP602945    AIGHKPNTDIFKG-----Q-LEMK-DGYIITN---SG-L------------SG--NATGT
BURM902465    AIGHKPNTDIFKG-----Q-LEMK-DGYIITN---SG-L------------SG--NATGT
```

```
BURMS00740   AIGHKPNTDIFKG-----Q-LEMK-DGYIITN---SG-L------------SG--NATGT
BURTA01515   AIGHKPNTDIFKG-----Q-LEMK-DGYIITN---GG-L------------SG--NATGT
BURM102386   AIGHKPNTDLFQG-----Q-LEMK-DGYILTK---SG-L------------QG--NATAT
BURL300890   AIGHKPNTDLFQG-----Q-LEMK-DGYILTK---SG-L------------HG--NATST
BURVG00860   AIGHKPNTDLFQG-----Q-LEMK-DGYILTK---SG-L------------QG--NATST
BURCM00829   AIGHKPNTDLFQG-----Q-LEMK-DGYILTK---SG-L------------HG--NATST
BURA400832   AIGHKPNTDLFQG-----Q-LEMK-DGYILTK---SG-L------------HG--NATST
BURCA00489   AIGHKPNTDLFQG-----Q-LEMK-DGYILTK---SG-L------------QG--NATST
BURCH00966   AIGHKPNTDLFQG-----Q-LEMK-DGYILTK---SG-L------------QG--NATST
BURCC00927   AIGHKPNTDLFQG-----Q-LEMK-DGYILTK---SG-L------------QG--NATST
BURCJ02922   AIGHKPNTDLFQG-----Q-LEMK-DGYILTK---SG-L------------QG--NATST
EDWI902348   AIGHSPNTAIFDG-----Q-LTLS-NGYIQVQ---SG-L------------NG--NATQT
EDWTF01971   AIGHSPNTAIFDG-----Q-LTLN-NGYIQVQ---SG-L------------NG--NATQT
EDWTE02176   AIGHSPNTAIFDG-----Q-LTLN-NGYIQVQ---SG-L------------NG--NATQT
SODGM01094   AIGHSPNTAIFGD-----Q-LALQ-NGYLRVQ---SG-I------------NG--NATAT
MOREP00394   AIGHRPNTAIFGS-----Q-LALQ-NGYLLVQ---SG-I------------NG--NATST
RAHSY01432   AIGHSPNTGIFEG-----Q-LELK-DGYIKVQ---SG-S------------HG--NATQT
RAHAC01419   AIGHSPNTGIFEG-----Q-LELK-DGYIKVQ---SG-S------------HG--NATQT
ERWBE01489   AIGHSPNTGIFKD-----Q-LELE-NGYIKVQ---SG-L------------QG--NATQT
PANAM01339   AIGHSPNTAIFDG-----Q-LALE-NGYIKVQ---SG-L------------HG--NATQT
PANAA00662   AIGHSPNTAIFDG-----Q-LALE-NGYIKVQ---SG-L------------HG--NATQT
PANSA01303   AIGHSPNTAIFAG-----Q-LALE-NGYIKVQ---SG-L------------HG--NATQT
ERWT902154   AIGHSPNTAIFDG-----Q-LALE-NGYIKVQ---SG-T------------HG--NATQT
ERWAC01330   AIGHSPNTAIFNG-----Q-LALE-NGYIKVQ---SG-I------------HG--NATQT
ERWAE01326   AIGHSPNTAIFNG-----Q-LALE-NGYIKVQ---SG-I------------HG--NATQT
ERWPE02241   AIGHSPNTAIFNG-----Q-LALE-NGYIKVQ---SG-I------------HG--NATQT
ERWP602414   AIGHSPNTAIFNG-----Q-LALE-NGYIKVQ---SG-I------------HG--NATQT
ERWSE02396   AIGHSPNTAIFNG-----Q-LALE-NGYIKVQ---SG-I------------HG--NATQT
PECCP01694   AIGHSPNTAVFGG-----Q-LELE-NGYIKVQ---SG-I------------HG--NATQT
PECWW01889   AIGHSPNTAVFGG-----Q-LALE-NGYIKVQ---SG-I------------HG--NATQT
PECSS01870   AIGHSPNTAVFGG-----Q-LALE-NGYIKVQ---SG-I------------HG--NATQT
PECAS02624   AIGHSPNTAVFGG-----Q-LALK-NGYIKVQ---SG-I------------HG--NATQT
DICDC02220   AIGHSPNTAIFNG-----Q-LALE-NGYIKVQ---SG-T------------NG--NATQT
DICZE02284   AIGHSPNTAIFGD-----Q-LALE-NGYIKVQ---SG-I------------HG--NATQT
DICD302017   AIGHSPNTAIFGD-----Q-LELE-NGYIKVQ---SG-I------------HG--NATQT
DICD502250   AIGHSPNTAIFGD-----Q-LALE-NGYIKVQ---SG-I------------HG--NATQT
XENBS00829   AIGHSPNTGIFEG-----Q-LDLQ-NGYIKVQ---SG-L------------HG--NATQT
XENNA01480   AIGHSPNTGIFDG-----Q-LELE-GGYLKVQ---SG-L------------HG--NATQT
PHOLL01537   AIGHTPNTAIFES-----Q-LELD-NGYIKVQ---SG-L------------QG--NATQT
PHOAA02804   AIGHTPNTAIFAG-----Q-LELD-NGYIKVQ---SG-L------------QG--NATQT
SERP501669   AIGHSPNTSIFGG-----Q-LELE-NGYIKVQ---SG-I------------HG--NATQT
SERSA01625   AIGHSPNTSIFGG-----Q-LELE-NGYIKVQ---SG-I------------HG--NATQT
YERPE01271   AIGHSPNTGIFSD-----Q-LALE-NGYIKVQ---SG-L------------QG--NATQT
YERPS01390   AIGHSPNTGIFSD-----Q-LALE-NGYIKVQ---SG-L------------QG--NATQT
YERPA00627   AIGHSPNTGIFSD-----Q-LALE-NGYIKVQ---SG-L------------QG--NATQT
YERPN02489   AIGHSPNTGIFSD-----Q-LALE-NGYIKVQ---SG-L------------QG--NATQT
YERPP02190   AIGHSPNTGIFSD-----Q-LALE-NGYIKVQ---SG-L------------QG--NATQT
YERP302544   AIGHSPNTGIFSD-----Q-LALE-NGYIKVQ---SG-L------------QG--NATQT
YERPB01460   AIGHSPNTGIFSD-----Q-LALE-NGYIKVQ---SG-L------------QG--NATQT
YERPY02633   AIGHSPNTGIFSD-----Q-LALE-NGYIKVQ---SG-L------------QG--NATQT
YERPG01392   AIGHSPNTGIFSD-----Q-LALE-NGYIKVQ---SG-L------------QG--NATQT
YERPD01171   AIGHSPNTGIFSD-----Q-LALE-NGYIKVQ---SG-L------------QG--NATQT
YERP100890   AIGHSPNTGIFSD-----Q-LALE-NGYIKVQ---SG-L------------QG--NATQT
YERPZ01206   AIGHSPNTGIFSD-----Q-LALE-NGYIKVQ---SG-L------------QG--NATQT
YERPH02469   AIGHSPNTGIFSD-----Q-LALE-NGYIKVQ---SG-L------------QG--NATQT
YERE801438   AIGHSPNTAIFDG-----Q-LALE-NGYIKVQ---SG-I------------QG--NATQT
YERE302565   AIGHSPNTAIFGG-----Q-LALE-NGYIKVQ---SG-I------------QG--NATQT
YERE100392   AIGHSPNTAIFGG-----Q-LALE-NGYIKVQ---SG-I------------QG--NATQT
PROMH00687   AIGHSPNTAIFED-----Q-LALD-NGYIKVQ---SG-T------------QG--NATQT
PROSM03196   AIGHSPNTAIFDG-----Q-LDLE-NGYIKVQ---SG-T------------HG--NATQT
TOLAT02279   AIGHSPNTAIFDN-----Q-LEMQ-NGYLKVK---GG-S------------DG--FATQT
AERVB02262   AIGHQPNTQIFDG-----Q-LEMQ-NGYLKVR---GG-L------------DG--FATQT
AERHH01811   AIGHQPNTQIFAG-----Q-LDMQ-NGYLKVR---GG-L------------DG--FATQT
AERS402185   AIGHQPNTQIFEG-----Q-LAMQ-NGYLIVR---GG-L------------DG--FATQT
PSYIN02070   AIGHKPNTGMFAG-----Q-LEMN-HGYLKVQ---SG-N------------DG--NATQT
```

SHELP02018 AIGHKPNTQMFEG-----Q-LEMN-NGYIKVQ---SG-L------------GG--NATQT
SHEVD02297 AIGHSPNTGMFEG-----Q-LDMN-HGYIKVQ---SG-L------------NG--NATQT
SHEPW02341 AIGHSPNTGMFEG-----Q-LEMN-NGYIKVE---SG-L------------NG--NATQA
SHEPA02202 AIGHKPNTSMFEG-----Q-LEMN-NGYIKVQ---SG-L------------DG--NATQT
SHEHH02007 AIGHKPNTEMFEG-----Q-LEMN-NGYIKVQ---SG-L------------DG--NATQT
SHESH02117 AIGHKPNTQMFEG-----Q-LEMN-NGYIKVQ---SG-L------------DG--NATQA
SHEWM02454 AIGHKPNTQMFEG-----Q-LEMN-NGYIKVQ---SG-L------------DG--NATQA
SHEAM01758 AIGHSPNTGIFEG-----Q-LEMN-NGYIKVQ---SG-L------------NG--NATQT
SHEON02159 AIGHSPNTGIFEG-----Q-LEMN-NGYLKVQ---SG-L------------QG--NATQT
SHESM01945 AIGHSPNTGIFEG-----Q-LEMN-NGYLKVQ---SG-L------------QG--NATQT
SHESR01991 AIGHSPNTGIFEG-----Q-LEMN-NGYLKVQ---SG-L------------QG--NATQT
SHESA02025 AIGHSPNTGIFEG-----Q-LEMN-NGYLKVQ---SG-L------------QG--NATQT
SHESW01953 AIGHSPNTSIFEG-----Q-LEMN-HGYLKVQ---SG-L------------QG--NATQT
SHEPC01976 AIGHSPNTSIFEG-----Q-LEMN-HGYLKVQ---SG-L------------QG--NATQT
SHEP201929 AIGHSPNTSIFEG-----Q-LEMN-HGYLKVQ---SG-L------------QG--NATQT
SHEB502016 AIGHSPNTGIFEG-----Q-LEMN-HGYLKVQ---SG-L------------QG--NATQT
SHEB802168 AIGHSPNTGIFEG-----Q-LEMN-HGYLKVQ---SG-L------------QG--NATQT
SHEB202117 AIGHSPNTGIFEG-----Q-LEMN-HGYLKVQ---SG-L------------QG--NATQT
SHEB902270 AIGHSPNTGIFEG-----Q-LEMN-HGYLKVQ---SG-L------------QG--NATQT
SHEB602258 AIGHSPNTGIFEG-----Q-LEMN-HGYLKVQ---SG-L------------QG--NATQT
SHEDO01680 AIGHSPNTGIFAG-----Q-LEMK-HGYLQVN---SG-L------------QG--NATQT
SHEFN01895 AIGHSPNTGIFAG-----Q-LEMN-HGYLQVN---SG-L------------QG--NATQT
PSEHT01677 AIGHKPNTDMFVG-----Q-LEMK-DGYIIVE---SG-L------------NG--NATQT
ALISL01611 AIGHQPNTAIFNG-----Q-LDME-NGYIKVK---SG-L------------EG--NATQT
VIBF100893 AIGHQPNTAIFNG-----Q-LDME-NGYIKVK---SG-L------------EG--NATQT
VIBFM00891 AIGHQPNTAIFNG-----Q-LDME-NGYIKVK---SG-L------------EG--NATQT
OCESG01399 AIGHQPNTGIFEG-----Q-LEME-NGYLKVQ---SG-L------------HG--NATQT
VIBA701872 AIGHKPNTDMFAG-----K-LEMK-EGYIVVR---SG-L------------DG--NATQT
VIBVY01451 AIGHSPNTQIFQG-----Q-LDMK-DGYILVK---SG-L------------EG--NATQT
VIBVU02545 AIGHSPNTQIFQG-----Q-LDMK-DGYILVK---SG-L------------EG--NATQT
VIBVM01748 AIGHSPNTQIFQG-----Q-LDMK-DGYILVK---SG-L------------EG--NATQT
VIBCH01157 AIGHQPNSQIFEG-----Q-LEMK-NGYIVVK---SG-L------------EG--NATQT
VIBCM01114 AIGHQPNSQIFEG-----Q-LEMK-NGYIVVK---SG-L------------EG--NATQT
VIBCJ02064 AIGHQPNSQIFEG-----Q-LEMK-NGYIVVK---SG-L------------EG--NATQT
VIBC300725 AIGHQPNSQIFEG-----Q-LEMK-NGYIVVK---SG-L------------EG--NATQT
FERBD01969 AIGHQPNTQIFEG-----Q-LEMK-DGYILVQ---SG-L------------EG--NATAT
VIBFN02085 AIGHQPNTAIFEG-----Q-LDMK-DGYIIVK---SG-L------------EG--NATQT
VIBTL01089 AIGHQPNTEIFKG-----Q-VDMK-DDYIIVQ---SG-L------------EG--NATQT
VIBPA01248 AIGHQPNTQIFEG-----Q-LEMK-DGYIVVK---SG-L------------EG--NATQT
VIBAE02128 AIGHQPNTQIFEG-----Q-VDMK-DGYIIVK---SG-L------------EG--NATQT
VIBCB00599 AIGHQPNTQIFEG-----Q-VDMK-DGYILVQ---SG-L------------EG--NATQT
PANVC00701 AIGHSPNTAIFEG-----Q-LALE-NGYIKVQ---SG-L------------HG--NATQT
SHIBC02443 AIGHSPNTAIFAG-----Q-LALE-NGYIKVN---SG-S------------NG--NATQT
ENTBF02792 AIGHSPNTAIFEG-----Q-LELE-NGYIKVQ---SG-I------------HG--NATQT
KLEP700892 AIGHSPNTAIFEG-----Q-LELE-NGYIKVQ---SG-I------------HG--NATQT
KLEPH01782 AIGHSPNTAIFEG-----Q-LELE-NGYIKVQ---SG-I------------HG--NATQT
KLEP303551 AIGHSPNTAIFEG-----Q-LELE-NGYIKVQ---SG-I------------HG--NATQT
KLEVT03420 AIGHSPNTAIFEG-----Q-LELE-NGYIKVQ---SG-I------------HG--NATQT
ENTAK02985 AIGHSPNTAIFEG-----Q-LALE-NGYIKVQ---SG-I------------HG--NATQT
KLEOK03151 AIGHSPNTAIFAG-----Q-LELE-NGYIKVQ---SG-I------------HG--NATQT
SALAR01925 AIGHSPNTAIFEG-----Q-LELE-NGYIKVQ---SG-T------------HG--NATQT
SALBC00812 AIGHSPNTAIFEG-----Q-LELE-NGYIKVQ---SG-T------------HG--NATQT
SALPC00926 AIGHSPNTALFEG-----Q-LELE-NGYIKVQ---SG-T------------HG--NATQT
SALTI00868 AIGHSPNTALFEG-----Q-LELE-NGYIKVQ---SG-T------------HG--NATQT
SALCH00905 AIGHSPNTALFEG-----Q-LELE-NGYIKVQ---SG-T------------HG--NATQT
SALPA01706 AIGHSPNTALFEG-----Q-LELE-NGYIKVQ---SG-T------------HG--NATQT
SALTY00927 AIGHSPNTALFEG-----Q-LELE-NGYIKVQ---SG-T------------HG--NATQT
SALPK01786 AIGHSPNTALFEG-----Q-LELE-NGYIKVQ---SG-T------------HG--NATQT
SALHS00978 AIGHSPNTALFEG-----Q-LELE-NGYIKVQ---SG-T------------HG--NATQT
SALEP00854 AIGHSPNTALFEG-----Q-LELE-NGYIKVQ---SG-T------------HG--NATQT
SALDC00926 AIGHSPNTALFEG-----Q-LELE-NGYIKVQ---SG-T------------HG--NATQT
SALA400892 AIGHSPNTALFEG-----Q-LELE-NGYIKVQ---SG-T------------HG--NATQT
SALG200876 AIGHSPNTALFEG-----Q-LELE-NGYIKVQ---SG-T------------HG--NATQT
SALTS00888 AIGHSPNTALFEG-----Q-LELE-NGYIKVQ---SG-T------------HG--NATQT
SALT400905 AIGHSPNTALFEG-----Q-LELE-NGYIKVQ---SG-T------------HG--NATQT

```
SALPS01908      AIGHSPNTALFEG-----Q-LELE-NGYIKVQ---SG-T------------HG--NATQT
SALT101040      AIGHSPNTALFEG-----Q-LELE-NGYIKVQ---SG-T------------HG--NATQT
SALTD00960      AIGHSPNTALFEG-----Q-LELE-NGYIKVQ---SG-T------------HG--NATQT
SALPB02472      AIGHSPNTALFEG-----Q-LELE-NGYIKVQ---SG-T------------HG--NATQT
SALNS00928      AIGHSPNTALFEG-----Q-LELE-NGYIKVQ---SG-T------------HG--NATQT
SALSV01000      AIGHSPNTALFEG-----Q-LELE-NGYIKVQ---SG-T------------HG--NATQT
ECOS500812      AIGHSPNTAIFEG-----Q-LELE-NGYIKVQ---SG-I------------HG--NATQT
ECOL600981      AIGHSPNTAIFEG-----Q-LELE-NGYIKVQ---SG-I------------HG--NATQT
ECOL500883      AIGHSPNTAIFEG-----Q-LELE-NGYIKVQ---SG-I------------HG--NATQT
ECOUT00876      AIGHSPNTAIFEG-----Q-LELE-NGYIKVQ---SG-I------------HG--NATQT
ECOK100776      AIGHSPNTAIFEG-----Q-LELE-NGYIKVQ---SG-I------------HG--NATQT
ECOSM02148      AIGHSPNTAIFEG-----Q-LELE-NGYIKVQ---SG-I------------HG--NATQT
ECOLU01057      AIGHSPNTAIFEG-----Q-LELE-NGYIKVQ---SG-I------------HG--NATQT
ECO7I02136      AIGHSPNTAIFEG-----Q-LELE-NGYIKVQ---SG-I------------HG--NATQT
ECO8100828      AIGHSPNTAIFEG-----Q-LELE-NGYIKVQ---SG-I------------HG--NATQT
ECO4500862      AIGHSPNTAIFEG-----Q-LELE-NGYIKVQ---SG-I------------HG--NATQT
ECOAB00879      AIGHSPNTAIFEG-----Q-LELE-NGYIKVQ---SG-I------------HG--NATQT
ECO4400961      AIGHSPNTAIFEG-----Q-LELE-NGYIKVQ---SG-I------------HG--NATQT
ECOUM02550      AIGHSPNTAIFEG-----Q-LELE-NGYIKVQ---SG-I------------HG--NATQT
ECOKI00869      AIGHSPNTAIFEG-----Q-LELE-NGYIKVQ---SG-I------------HG--NATQT
ECOC100920      AIGHSPNTAIFEG-----Q-LELE-NGYIKVQ---SG-I------------HG--NATQT
ECOC200920      AIGHSPNTAIFEG-----Q-LELE-NGYIKVQ---SG-I------------HG--NATQT
ECO8N00803      AIGHSPNTAIFEG-----Q-LELE-NGYIKVQ---SG-I------------HG--NATQT
ECO2700862      AIGHSPNTAIFEG-----Q-LELE-NGYIKVQ---SG-I------------HG--NATQT
ECO2600974      AIGHSPNTAIFEG-----Q-LELE-NGYIKVQ---SG-I------------HG--NATQT
ECOH100942      AIGHSPNTAIFEG-----Q-LELE-NGYIKVQ---SG-I------------HG--NATQT
SHIB301848      AIGHSPNTAIFEG-----Q-LELE-NGYIKVQ---SG-I------------HG--NATQT
ECOLI00846      AIGHSPNTAIFEG-----Q-LELE-NGYIKVQ---SG-I------------HG--NATQT
ECO5700970      AIGHSPNTAIFEG-----Q-LELE-NGYIKVQ---SG-I------------HG--NATQT
SHISS00756      AIGHSPNTAIFEG-----Q-LELE-NGYIKVQ---SG-I------------HG--NATQT
SHIBS00679      AIGHSPNTAIFEG-----Q-LELE-NGYIKVQ---SG-I------------HG--NATQT
SHIDS01857      AIGHSPNTAIFEG-----Q-LELE-NGYIKVQ---SG-I------------HG--NATQT
ECO2400906      AIGHSPNTAIFEG-----Q-LELE-NGYIKVQ---SG-I------------HG--NATQT
ECODH00784      AIGHSPNTAIFEG-----Q-LELE-NGYIKVQ---SG-I------------HG--NATQT
ECOHS00922      AIGHSPNTAIFEG-----Q-LELE-NGYIKVQ---SG-I------------HG--NATQT
ECOLC02627      AIGHSPNTAIFEG-----Q-LELE-NGYIKVQ---SG-I------------HG--NATQT
ECO5E00946      AIGHSPNTAIFEG-----Q-LELE-NGYIKVQ---SG-I------------HG--NATQT
ECOSE00936      AIGHSPNTAIFEG-----Q-LELE-NGYIKVQ---SG-I------------HG--NATQT
ECO5500894      AIGHSPNTAIFEG-----Q-LELE-NGYIKVQ---SG-I------------HG--NATQT
ECO8A00877      AIGHSPNTAIFEG-----Q-LELE-NGYIKVQ---SG-I------------HG--NATQT
ECOBB00863      AIGHSPNTAIFEG-----Q-LELE-NGYIKVQ---SG-I------------HG--NATQT
ECO5T00943      AIGHSPNTAIFEG-----Q-LELE-NGYIKVQ---SG-I------------HG--NATQT
ECOBW00723      AIGHSPNTAIFEG-----Q-LELE-NGYIKVQ---SG-I------------HG--NATQT
ECO1000919      AIGHSPNTAIFEG-----Q-LELE-NGYIKVQ---SG-I------------HG--NATQT
ECOBD02604      AIGHSPNTAIFEG-----Q-LELE-NGYIKVQ---SG-I------------HG--NATQT
ECOD102659      AIGHSPNTAIFEG-----Q-LELE-NGYIKVQ---SG-I------------HG--NATQT
ECOBR00856      AIGHSPNTAIFEG-----Q-LELE-NGYIKVQ---SG-I------------HG--NATQT
ECOLX02610      AIGHSPNTAIFEG-----Q-LELE-NGYIKVQ---SG-I------------HG--NATQT
ECO1A00939      AIGHSPNTAIFEG-----Q-LELE-NGYIKVQ---SG-I------------HG--NATQT
ECOCB01054      AIGHSPNTAIFEG-----Q-LELE-NGYIKVQ---SG-I------------HG--NATQT
ECOKO02859      AIGHSPNTAIFEG-----Q-LELE-NGYIKVQ---SG-I------------HG--NATQT
ECO1E03320      AIGHSPNTAIFEG-----Q-LELE-NGYIKVQ---SG-I------------HG--NATQT
ECOLW01217      AIGHSPNTAIFEG-----Q-LELE-NGYIKVQ---SG-I------------HG--NATQT
SHIFL01399      AIGHSPNTAIFEG-----Q-LELE-NGYIKVQ---SG-I------------HG--NATQT
SHIF800759      AIGHSPNTAIFEG-----Q-LELE-NGYIKVQ---SG-I------------HG--NATQT
SHIF200805      AIGHSPNTAIFEG-----Q-LELE-NGYIKVQ---SG-I------------HG--NATQT
CITK802118      AIGHSPNTAIFEG-----Q-LELE-NGYIKVQ---SG-I------------HG--NATQT
CITRI00924      AIGHSPTTAIFEG-----Q-LELE-NGYIKVQ---SG-I------------HG--NATQT
CROS802385      AIGHSPNTAIFEG-----Q-LELE-NGYIKVQ---SG-I------------HG--NATQT
CROTZ01500      AIGHSPNTAIFDG-----Q-LELE-NGYIKVQ---SG-I------------HG--NATQT
ENTLS02865      AIGHSPNTAVFDG-----Q-LELE-NGYIKVQ---SG-I------------HG--NATQT
ENT3801400      AIGHSPNTGIFDG-----Q-LELE-NGYIKVQ---SG-I------------HG--NATQT
ENTAL01384      AIGHSPNTAIFDG-----Q-LELE-NGYIKVQ---SG-I------------HG--NATQT
ENTCC02679      AIGHSPNTAIFEG-----Q-LELE-NGYIKVQ---SG-I------------HG--NATQT
                  .
```

STRT101547	SVPGIFAIGDVRQ-KDL----RQITTAVGEGAIAGIEAYNYVTALGDK------------
STRT201508	SVPGIFAIGDVRQ-KDL----RQITTAVGEGAIAGIEAYNYVTALGDK------------
STRTD01356	SVPGIFAIGDVRQ-KDL----RQITTAVGEGAIAGIEAYNYVTALGDK------------
STRTN01533	SVPGIFAIGDVRQ-KDL----RQITTAVGEGAIAGIEAYNYVTALGDK------------
STRE500345	RIPGIFAIGDVRQ-KDL----RQITTAVGDGATAGIEAYNYVTALGDK------------
STRE801625	RIPGIFAIGDVRQ-KDL----RQITTAVGDGATAGIEAYNYVTALGDK------------
STREH01636	RIPGIFAIGDVRQ-KDL----RQITTAVGDGATAGIEAYNYVTALGDK------------
STREC01656	SIPGIFAVGDVRQ-KQL----RQITTAVGDGAIAGQGVYHYIESLSS-------------
STREM01495	SIPGIFAVGDVRQ-KQL----RQITTAVGDGAIAGQGVYHYIENLTS-------------
STRE401629	SIPGIFAVGDVRQ-KQL----RQITTAVGDGAIAGQGVYHYIESLSS-------------
STRS700387	SIPGIFAVGDVRQ-KQL----RQITTAVGDGAIAGQGVYHYIESLSS-------------
STRDG01631	SIPGIFAIGDVRQ-KDL----RQITTAVGDGAIAGQGVYHYIENMPS-------------
STRP301388	SIPGIFAIGDVRQ-KDL----RQITTAVGDGAIAGQGVYHYLESFPS-------------
STRPZ01205	SIPGIFAIGDVRQ-KDL----RQITTAVGDGAIAGQGVYHYLESFPS-------------
STRPQ00464	SIPGIFAIGDVRQ-KDL----RQITTAVGDGAIAGQGVYHYLESFPS-------------
STRPD01453	SIPGIFAIGDVRQ-KDL----RQITTAVGDGAIAGQGVYHYLESFSS-------------
STRP601399	SIPGIFAIGDVRQ-KDL----RQITTAVGDGAIAGQGVYHYLESFPS-------------
STRP801366	SIPGIFAIGDVRQ-KDL----RQITTAVGDGAIAGQGVYHYLESFPS-------------
STRPF01453	SIPGIFAIGDVRQ-KDL----RQITTAVGDGAIAGQGVYHYLESFPS-------------
STRPG00427	SIPGIFAIGDVRQ-KDL----RQITTAVGDGAIAGQGVYHYLESFPS-------------
STRA300284	SIPGLYAIGDVRQ-KDL----RQIATAVGEGAIAGQGVYNYITEHF--------------
STRA500290	SIPGLYAIGDVRQ-KDL----RQIATAVGEGAIAGQGVYNYITEHF--------------
STRA100288	SIPGLYAIGDVRQ-KDL----RQIATAVGEGAIAGQGVYNYITEHF--------------
STRA200291	SIPGLYAIGDVRQ-KDL----RQIATAVGEGAIAGQGVYNYITEHF--------------
STRIC00348	TIPGIFAIGDVRK-KEL----RQITTAVGDGAIAGQGVYQYIENMK--------------
STRPX00375	PKAGIFAIGDVRQ-KEL----RQIATAVGDGAIAGQGVYQYIENMT--------------
STRMD00432	PKAGIFAIGDVRQ-KEL----RQIATAVGDGAIAGQGVYQYIENMK--------------
STRS201734	TQSGIYAIGDIRQ-NQL----RQIATAVGNGAVAGQEVYNYITELAE-------------
STRSY01721	TQSGIYAIGDIRQ-NQL----RQIATAVGNGAVAGQEVYNYITELAE-------------
STRSX01553	TQSGIYAIGDIRQ-NQL----RQIATAVGNGAVAGQEVYNYITELAE-------------
STRSE01478	TQSGIYAIGDIRQ-NQL----RQIATAVGNGAVAGQEVYNYITELAE-------------
STREJ01635	TQSGIYAIGDIRQ-NQL----RQIATAVGNGAVAGQEVYNYITELAE-------------
STRGZ01544	TQSGIYAIGDIRQ-NQL----RQIATAVGNGAVAGQEVYNYITELAE-------------
STRS401593	TQSGIYAIGDIRQ-NQL----RQIATAVGNGAVAGQEVYNYITRTS--------------
LACGT00720	SIPGIFAVGDVRQ-KDF----RQITTAVGDGAQAAQEAYKYVEG----------------
LACGL00738	SIPGIFAVGDVRQ-KDF----RQITTAVGDGAQAAQEAYKYVEG----------------
STRSV01774	SVAGIYAVGDVRQ-KDL----RQITTAVGDGAIASQEAYKYLTEQA--------------
STRIJ00350	AISGIYAIGDVRE-KDL----RQITTAVGDGAIAGQEVYKYITENC--------------
STROU01248	AVDGIFAVGDVRQ-KDL----RQVTTAVGDGAIAGQEAYKFITEHS--------------
STRM601286	AVDGIFAVGDVRQ-KDL----RQVATAVGDGAIAGQEAYKFITEHC--------------
STRES00962	AVDGIFAVGDVRL-KDL----RQVTTAVGDGAIAGQEAYKFITEHS--------------
STRP701387	AVDGIFAVGDVRL-KDL----RQVTTAVGDGAIAGQEAYKFITEHS--------------
STRZT00756	AVDGIFAVGDVRL-KDL----RQVTTAVGDGAIAGQEAYKFITEHS--------------
STRP001079	AVDGIFAVGDVRL-KDL----RQVTTAVGDGAIAGQEAYKFITEHS--------------
STRZO01270	AVDGIFAVGDVRL-KDL----RQVTTAVGDGAIAGQEAYKFITEHS--------------
STRZ600815	AVDGIFAVGDVRL-KDL----RQVTTAVGDGAIAGQEAYKFITEHS--------------
STRET00826	AVDGIFAVGDVRL-KDL----RQVTTAVGDGAIAGQEAYKFITEHS--------------
STRPS01429	AVDGIFAVGDVRL-KDL----RQVTTAVGDGAIAGQEAYKFITEHS--------------
STRZN01287	AVDGIFAVGDVRL-KDL----RQVTTAVGDGAIAGQEAYKFITEHS--------------
STRR601306	AVDGIFAVGDVRL-KDL----RQVTTAVGDGAIAGQEAYKFITEHS--------------
STRP201217	AVDGIFAVGDVRL-KDL----RQVTTAVGDGAIAGQEAYKFITEHS--------------
STRZP01342	AVDGIFAVGDVRL-KDL----RQVTTAVGDGAIAGQEAYKFITEHS--------------
STRZI01226	AVDGIFAVGDVRL-KDL----RQVTTAVGDGAIAGQEAYKFITEHS--------------
STRPN01360	AVDGIFAVGDVRL-KDL----RQVTTAVGDGAIAGQEAYKFITEHS--------------
STRP401357	AVDGIFAVGDVRL-KDL----RQVTTAVGDGAIAGQEAYKFITEHS--------------
STRZJ01282	AVDGIFAVGDVRL-KDL----RQVTTAVGDGAIAGQEAYKFITEHS--------------
STRPJ01336	AVDGIFAVGDVRL-KDL----RQVTTAVGDGAIAGQEAYKFITEHS--------------
STRPI01436	AVDGIFAVGDVRL-KDL----RQVTTAVGDGAIAGQEAYKFITEHS--------------
MARHT00044	SVPGVFAAGDVAD-PIY----RQLTTSVGAGTRAAMVAERYIAEQEETAQTRA-------
THEP300413	NIPGVFAAGDIRH-KSL----RQVITAAGDGAIAAYVAEKYIDSLKK-------------
THEPX00884	NIPGVFAAGDIRH-KSL----RQVITAAGDGAIAAYVAEKYIDSLKK-------------
THESX01857	NIPGVFAAGDIRH-KSL----RQVITAAGDGAIAAYVAEKYIDSLKK-------------
THEM301788	NIPGVFAAGDIRH-KSL----RQVITAAGDGATAAYVAEKYIDSLKK-------------
THEIA01812	NIPGVFAAGDIRH-KSL----RQVITAAGDGATAAYVAEKYIDSLKK-------------

THETC00443	NIPGVFAAGDVRK-KTL----RQVVTATADGAIAAYVAEKYIDSLE--------------
THESW01026	NIQGVFAAGDVRK-KTL----RQVVTATADGAIAAYVAEKYIDSL---------------
THEXL00360	NVPGVFAAGDVRK-KTL----RQVVTATADGAIAAYVAEKYIDSL---------------
THEID01127	NVPGIFAAGDVRS-KSC----RQIVTACGDGATAAFLAEHFLAEK---------------
THEOJ00147	NVPGLFAAGDIRE-KSL----RQVVTAVADGAIAAVSAEKYLEEHQMR------------
DESAS01237	SHAGIFAVGDVRQ-KTL----RQVVTAVADGAIAAVAAEKYLEEL---------------
DESK701373	SCPGLFAAGDVRQ-KSL----RQVVTAVADGAIAAVSAEKYLEQNR--------------
KYRT200729	AVPGVFAAGDVRE-TWL----RQVVTAVAEGAMAAMSAYYYVEEQS--------------
STACT00410	KLPGIYAAGDVRE-KGL----RQIVTATGDGSIAAQNAQAYIEEVKDKLATEA-------
STAS101952	SIPGIYAAGDVRE-KGL----RQIVTATGDGSIAAQSAIAYIEHIKDTIEA---------
STALH01969	AVPGIYAAGDVRD-KGL----RQIVTATGDGSIAAQSAIDYIEELKDKEA----------
STAEQ00422	KVRGIFAAGDVRD-KGL----RQIVTATGDGSIAAQSAADYITELKDN------------
STAES00543	KVRGIFAAGDVRD-KGL----RQIVTATGDGSIAAQSAADYITELKDN------------
STAAB00714	SVPGIFAAGDVRD-KGL----RQIVTATGDGSIAAQSAAEYIEHLNDQA-----------
STAA500765	SVPGIFAAGDVRD-KGL----RQIVTATGDGSIAAQSAAEYIEHLNDQA-----------
STAAM00757	SVPGIFAAGDVRD-KGL----RQIVTATGDGSIAAQSAAEYIEHLNDQA-----------
STAAW00726	SVPGIFAAGDVRD-KGL----RQIVTATGDGSIAAQSAAEYIEHLNDQA-----------
STAAS00733	SVPGIFAAGDVRD-KGL----RQIVTATGDGSIAAQSAAEYIEHLNDQA-----------
STAAN00727	SVPGIFAAGDVRD-KGL----RQIVTATGDGSIAAQSAAEYIEHLNDQA-----------
STAAC00807	SVPGIFAAGDVRD-KGL----RQIVTATGDGSIAAQSAAEYIEHLNDQA-----------
STAA300727	SVPGIFAAGDVRD-KGL----RQIVTATGDGSIAAQSAAEYIEHLNDQA-----------
STAA800734	SVPGIFAAGDVRD-KGL----RQIVTATGDGSIAAQSAAEYIEHLNDQA-----------
STAA100752	SVPGIFAAGDVRD-KGL----RQIVTATGDGSIAAQSAAEYIEHLNDQA-----------
STAA200774	SVPGIFAAGDVRD-KGL----RQIVTATGDGSIAAQSAAEYIEHLNDQA-----------
STAA900759	SVPGIFAAGDVRD-KGL----RQIVTATGDGSIAAQSAAEYIEHLNDQA-----------
STAAE00716	SVPGIFAAGDVRD-KGL----RQIVTATGDGSIAAQSAAEYIEHLNDQA-----------
STAAT00767	SVPGIFAAGDVRD-KGL----RQIVTATGDGSIAAQSAAEYIEHLNDQA-----------
STAAD00695	SVPGIFAAGDVRD-KGL----RQIVTATGDGSIAAQSAAEYIEHLNDQA-----------
STAA000815	SVPGIFAAGDVRD-KGL----RQIVTATGDGSIAAQSAAEYIEHLNDQA-----------
STAAH02407	SVPGIFAAGDVRD-KGL----RQIVTATGDGSIAAQSAAEYIEHLNDQA-----------
STAAF00763	SVPGIFAAGDVRD-KGL----RQIVTATGDGSIAAQSAAEYIEHLNDQA-----------
STAAK00744	SVPGIFAAGDVRD-KGL----RQIVTATGDGSIAAQSAAEYIEHLNDQA-----------
STAAJ00703	SVPGIFAAGDVRD-KGL----RQIVTATGDGSIAAQSAAEYIEHLNDQA-----------
STAAG00690	SVPGIFAAGDVRD-KGL----RQIVTATGDGSIAAQSAAEYIEHLNDQA-----------
STAA400732	SVPGIFAAGDVRD-KGL----RQIVTATGDGSIAAQSAAEYIEHLNDQA-----------
STAAR00788	SAPGIFAAGDVRD-KGL----RQIVTATGDGSIAAQSAAEYIEHLNDQA-----------
LISSS02378	NRPGIFAAGDVRA-KSL----RQIVTATGDGGLAGQNAQKYVEELKEALEAETAK-----
LISIN02590	NLPGIFAAGDVRA-KSL----RQIVTATGDGGLAGQNAQKYVEELKEALEAEAAK-----
LISW602421	NLPGIFAAGDVRA-KSL----RQIVTATGDGGLAGQNAQKYVEELKEALEAETAK-----
EXISA00870	NIPGIFAAGDVRE-KTL----RQVVTATNDGSIAAQNAQHFIEGLLESLQEQNA------
EXIS202358	NIKGIFAAGDVRE-KTL----RQVVTATNDGSIAAQNAQHYIEALLEELQESSQV-----
EXIAB02180	NIKGIFAAGDVRE-KTL----RQVVTATNDGSIAAQNAQHYIEALLEELQESSQV-----
OCEIH02462	SIPGIFAAGDIRD-KEL----RQIVTATGDGSIAAEAAIKYVEDLEEKIKSK--------
BACIE01058	DIPGIFAAGDIRD-KHL----RQIVTATGDGSIAAQTAQHYVENLMEELNATAEKV----
BACCJ03468	KIPGIFAAGDIRE-KTL----RQIVTATGDGSIAAQSAQHYVENLVESLQANKS------
BACHD03507	SVPGIFAAGDVRE-KSL----RQIVTATGDGSLAAQNVQHYIEELAEKVKN---------
BACPE03382	KVPGIYAAGDIRE-KSL----RQIVTATGDGSLAAQNVQHYLESLAEGTK----------
SOLSS00743	SIPGIYAAGDVRD-KML----RQIVTATGDGSIAAQSAQHYVEEIKEKINQ---------
BACC600715	KIPGIFAAGDVRE-KTL----RQIVTATGDGSIAAQNAQHYVEELKEAMKAG--------
ANOFW02486	KVPGIFAAGDVRE-KSL----RQIVTATGDGSIAAQSAQHYVEELKEKLNIQ--------
GEOKA03042	KVPGIFAAGDVRE-KTL----RQIVTATGDGSIAAQSAQHYVEELKEKLNKQGVS-----
GEOSY02976	KVPGIFAAGDVRE-KTL----RQIVTATGDGSIAAQSAQHYVEELKEKLHKQGVS-----
GEOTN02952	KVPGIFAAGDVRE-KML----RQIVTATGDGSIAAQSAQHYVEELKEKLNAQGAN-----
GEOSW02588	KVPGIFAAGDVRE-KSL----RQIVTATGDGSIAAQSAQHYVEELKEKLNIK--------
GEOS000381	KVPGIFAAGDVRE-KSL----RQIVTATGDGSIAAQSAQHYVEELKEKLNIK--------
GEOTC00378	KVPGIFAAGDVRE-KSL----RQIVTATGDGSIAAQSAQHYVEELKEKLNIK--------
BACMD04906	KVPGVFAAGDVRE-KAL----RQIVTATGDGSIAAQAAQHYVEELKEKLKAIQH------
BACMQ04908	KVPGVFAAGDVRE-KAL----RQIVTATGDGSIAAQAAQHYVEELKEKLKAIQQ------
BACWK04830	KVPGIFAAGDVRE-KML----RQIVTATGDGSIAAQSAQHYIEELVEELKTVTEK-----
BACAN04889	KVPGIFAAGDVRE-KML----RQIVTATGDGSIAAQSAQHYVEELLEELKTVSEK-----
BACC105210	KVPGIFAAGDVRE-KML----RQIVTATGDGSIAAQSAQHYVEELLEELKTVSEK-----
BACC705051	KVPGIFAAGDVRE-KML----RQIVTATGDGSIAAQSAQHYVEELLEELKTVSEK-----
BACC005112	KVPGIFAAGDVRE-KML----RQIVTATGDGSIAAQSAQHYVEELLEELKTVSEK-----
BACC305043	KVPGIFAAGDVRE-KML----RQIVTATGDGSIAAQSAQHYVEELLEELKTVSEK-----
BACAC05232	KVPGIFAAGDVRE-KML----RQIVTATGDGSIAAQSAQHYVEELLEELKTVSEK-----

BACAA04720	KVPGIFAAGDVRE-KML----RQIVTATGDGSIAAQSAQHYVEELLEELKTVSEK-----
BACT005085	KVPGIFAAGDVRE-KML----RQIVTATGDGSIAAQSAQHYVEELLEELKTVREK-----
BACC205124	KIPGIFAAGDVRE-KML----RQIVTATGDGSIAAQSAQHYVEELLEELKTVSEK-----
BACC405064	KIPGIFAAGDVRE-KML----RQIVTATGDGSIAAQSAQHYVEELLEELKTVSEK-----
BACT104712	KIPGIFAAGDVRE-KML----RQIVTATGDGSIAAQSAQHYVEELLEELKTVSEK-----
BACLD03769	KVEGIFAAGDIRE-KTL----RQIVTATGDGSIAAQSVQHYIEELKEKEKAVK-------
BACPZ03376	KVEGIFAAGDIRE-KSL----RQIVTATGDGSIAAQSVQHYVEELQETLKTLK-------
BACSU03600	KVEGIFAAGDIRE-KSL----RQIVTATGDGSIAAQSVQHYVEELQETLKTLK-------
BACST01653	KVEGIFAAGDIRE-KSL----RQIVTATGDGSIAAQSVQHYVEELQETLKTLK-------
BACPT03541	KVEGIFAAGDIRE-KSL----RQIVTATGDGSIAAQSVQHYVEELQETLKTLK-------
LEUGG00661	GIPGVFAVGDVRA-KEL----RQITTAVGDGSIAGQGVYDYISALPAAVNEQVI------
LEUGJ00629	GIPGVFAVGDVRA-KEL----RQITTAVGDGSIAGQGVYDYISALPAAVNEQVI------
LEUCJ00485	SIPGIFAIGDVRA-TEL----RQITTAVGDGGVAGQGAYDYISQLSVQDTDKVK------
LACAR00657	KVPGIFALGDVRD-KDL----RQIANAVGEGSVAGQAAYNYYQDLKD-------------
LACA300647	KVPGIFALGDVRD-KDL----RQIANAVGEGSVAGQAAYNYYQDLKD-------------
LACAL00643	KVPGIFALGDVRD-KDL----RQIANAVGEGSVAGQAAYNYYQDLKD-------------
LACKZ00964	KVPGIFALGDVRE-KDL----RQIANAVGEGSVAGQAAYNYYQDLKDRN-----------
LACRJ00357	AVPGIFAIGDVRE-TPL----RQVATAVGDGAIAGQQVYQYIKSMA--------------
LACRD00361	AVPGIFAIGDVRE-TPL----RQVATAVGDGAIAGQQVYQYIKSMA--------------
LACRS01501	AVPGIFAIGDVRE-TPL----RQVATAVGDGAIAGQQVYQYIKSMD--------------
LACSM00455	KVPGIFAVGDVRK-KHL----RQITTAVGDGGIAGQGVFDYVESLKSNK-----------
LACRG00878	KVPGIFAIGDVRA-KDL----RQITTAVGEGGTAGQGVFNYIQSLNDTSVEVKA------
LACRL00939	KVPGIFAIGDVRA-KDL----RQITTAVGEGGTAGQGVFNYIQSLNDTSVEVKA------
LACC300844	KVPGVFAIGDVRA-KDL----RQITTAVGEGGTAGQGVFNYIQSLNDTNIEIKA------
LACCZ00824	KVPGVFAIGDVRA-KDL----RQITTAVGEGGTAGQGVFNYIQSLNDTNIEIKA------
LACCB00988	KVPGVFAIGDVRA-KDL----RQITTAVGEGGTAGQGVFNYIQSLNDTNIEIKA------
LACCD01016	KVPGVFAIGDVRA-KDL----RQITTAVGEGGTAGQGVFNYIQSLNDTNIEIKA------
LACCC01014	KVPGVFAIGDVRA-KDL----RQITTAVGEGGTAGQGVFNYIQSLNDTNIEIKA------
LACBN01277	SIPGIYAIGDVRQ-KDL----RQIATAVGEGGIAGQQAFKYVESLADTSKSSVSEH----
LACBA00605	AVPGIFAIGDVRQ-KDL----RQITTAVGDGGIAGQAVFSYLEDLKSQAASAQ-------
LACPL00650	KVPGIFAVGDVRK-KDL----RQVATAVGEGGTAGQGVYTYITALGDKVNN---------
LACPJ00628	KVPGIFAVGDVRK-KDL----RQVATAVGEGGTAGQGVYTYITALGDKVNN---------
LACPS00585	KVPGIFAVGDVRK-KDL----RQVATAVGEGGTAGQGVYTYITALGDKVNN---------
PEDCP00497	KVPGIFAVGDVRK-KNL----RQIATAVGEGGTAAQQAFDYIQEINSKQNV---------
CARS100359	SQPGIFAIGDVRL-TPL----RQVATAVGDGSLAGNAVFHYVEKLKESLEAKAISSK---
AERUA00263	AVPGIFACGDVRK-KKL----RQVSTAVGDAGSAGQEAYQYVEALND-------------
ELUMP00590	NIEGIFAAGDCTTEQQF----RQVVIAAGAGAKAAVEAINYINLN---------------
SPHPG02940	KEKGLYAAGDVRD-TVF----RQLITAASDGAIAAHCASEYIDEIEGREYR---------
SPHGB01868	SIAGLYAAGDVRT-TVF----RQLITAASDGAIAAHCASEYIDELEGRAYR---------
TREPZ00273	SIPGIFVAGDVRA-SPF----RQVVVAAAEGAIAAHCAAEYIENFC--------------
TREAZ03414	SLPGLYAAGDVRA-TPF----RQVVVAAAEGAIAAHSAAEYIDALRGQAY----------
SPITD00734	SIPGLFAAGDVRN-TPF----RQIVVGAGEGAVAAHCAAKYIDELKGEAYE---------
SPITZ01373	SIPGLFAAGDVRN-TPF----RQIVVGAGEGAVAAHCAAKYIDELKGEAYE---------
TREPA00803	SVEGIFAAGDVRA-KSF----RQVITATSDGALAAHAAASYIDTLQN-------------
TREPS00802	SVEGIFAAGDVRA-KSF----RQVITATSDGALAAHAAASYIDTLQN-------------
TREPC00747	SVEGIFAAGDVRA-KSF----RQVITATSDGALAAHAAASYIDTLQN-------------
TREPM00823	SVEGIFAAGDVRA-KSF----RQVITATSDGALAAHAAASYIDTLQN-------------
TREPD00824	SVEGIFAAGDVRA-KSF----RQVITATSDGALAAHAAASYIDTLQN-------------
TREPU00781	SVEGIFAAGDVRA-KSF----RQVITAASDGALAAHAAASYIDTLQN-------------
ENCCU00216	NIPGLFAAGDVCD-KKY----RQAVTAAASGAISGMKAVEFLCKE---------------
HELM100964	NIPGFFAAGDIRV-QAP----KQVVCAAGDGATAALQAIAYLDSHK--------------
HELCP01490	SVEGLFAAGDVRT-QAS----KQVVCAAGDGATAALSAIAYLEHK---------------
ARCFU01526	NVEGVFAAGDCCD-NPL----KQVVTACGDGAVAAYSAYKYLTS----------------
FERPA02445	NVPGVFAAGDCCD-NPL----KQVVTACADGAIAANSAFEYIKMMQL-------------
ARCVS01910	SVPGVFAAGDCCD-NPL----KQVVTACSDGAIAANSAFEYIMSKS--------------
METEZ00677	SVEGIYAAGDCRK-SPL----WQVITAASDGAIAAAKAYEYIRNKG--------------
METHD00869	SVQGIYAAGDCRD-TTI----WQVVTAVADGAVAAVSAHEYIMNLELEQ-----------
METMA02304	SEKGIYAAGDCRD-TPI----WQLVTAVRDGAIAATAAYEYIEKIR--------------
METAC01311	SEKGIYAAGDCRD-TPI----WQLVAAVRDGAIAATAANEYIESLK--------------
KOSOT00298	NVPGVYAIGDVRE-KEV----RQIVTAAADGAIAVSHASRTYFDEE--------------
MARPK01613	NVKGVFAAGDVIQ-KEL----RQIITAAADGAIAASFAVREYFN----------------
SLAHD02439	NVPGVFAAGDLRK-KSL----RQVVTAAADGAIAATQALKYCED----------------
FILAD00976	SVEGVFVAGDCRQ-KLL----RQVVTAVSDGAIATVAAEKYIEETNN-------------
BUTPB02463	SAPGIFVAGDSRK-KRL----RQIVTAVADGANAVTSVQDFLVGKEK-------------
CLOPH00250	NVPGIFAAGDIRT-KEL----RQVITAASDGAVAVTGVERYLNHL---------------

```
CLOSW00592      NVPGIFAAGDVRT-KQL----RQVSTAVSDGANAITSVERYLTRI---------------
LACFC00207      SVPGVYAVGDVRN-TVL----RQIATAVGDGAIAGQQIFNYISEND--------------
CRYCD00853      EVAGVYAAGDVRE-KLL----RQVATAISDGAIAAEAAAESCSA----------------
EGGLE01847      EVPGVFAAGDVRT-KFL----HQVVTAVSDGAVCAEEAAEYLAI----------------
PYRFU01410      KVPGIFAAGDITN--VF----KQIAVAVGQGAIAANSAKEFLENWNKKDVE---------
PYRHO01476      KVPGIFAAGDITN--VF----KQIAVAVGQGAIAANSAKEFIESWNGKTIE---------
PYRAB00730      KVPGIFAAGDITN--VF----KQIAVAVGQGAIAANSAKEFIESWNGKSIE---------
PYRSN00014      KVPGIFAAGDITN--VF----KQIAVAVGQGAIAANSAKEFIESWNGKTIE---------
THEGJ00181      KVPGIFAAGDITN--VF----KQIAVAVGQGAIAANSAKEFLEKWAEKNGE---------
THEKO02097      KVPGIFAAGDITN--VF----KQIAVAVGQGAIAANSAKEFLEEWKNKNSE---------
THEON01610      KVKGIFAAGDITN--VF----KQIAVAVGQGAIAANSAKELLEEWGEKNGE---------
THES401476      KMKGIFAAGDITN--VF----KQIAVAVGQGAIAANSAKELIDEWNSKVVE---------
SYNWW02368      SIPGIFAAGDVRA-KKE----RQVATAVGEGALAGIAVSEYLQE----------------
UNCTG00012      SSTGIFACGDIRK-KQL----RQVVTAASDGAQAAVSAQRCIENL---------------
THEA101460      KTPGLFAAGDVRH-KPL----KQVVTATADGAVAAMSATKYLEEKEG-------------
DENA201647      SEPGIYVAGDVRT-KEY----RQITTAVSDGTVAAKSCEKYITDNFS-------------
DEFDS00488      NVPGIFAAGDVRK-KEL----RQIVTAVADGAVAAKMAERYIEENFPQEG----------
CALNY01292      NIPGIFAAGDVRT-KEL----RQVVTAVSDGAMAAKAAERYIEETFGSI-----------
SYNGF01652      NVDGVFAAGDIRN-TPL----RQVATAVGDGALAAVEAEKYISRKFK-------------
DESB201123      SMPGVFAAGDCCG-KLL----RQIVVAAGEGATAAYAAQRYLEEHE--------------
DEIPM00845      SVPMLFAAGDVSD-WVY----RQLATSVGTGTRAAMTAERALAELEAAFPHGESAQA--Q
DEIRA01924      NIPMLFAAGDVSD-YIY----RQLATSVGAGTRAAMMTERQLAALEVEGEEVTAAD----
DEIML01437      SVPGIFAAGDVSD-YVY----RQLATSVGAGTRAAMSAERMLAALEVEETAAD-------
DEIGD02532      SVPLLFAAGDVSD-YVY----RQLATSVGAGTRAAMSAERALAALELETTTAAD------
DEIDV00622      SVPMLFAAGDISD-YIY----RQLATSVGAGTRAAMSVERSLAALEVETETAAD------
TRURR01079      NVEGLFAAGDVAD-EIY----RQLGTSVGAGTRAAMAAERYLAEREAHAHVSRSEADILH
THETG01863      SEPGIFAAGDVAD-PIY----RQLTTSVGAGTRAAMMAERYLAEEAEKVKG---------
THET201543      SEPGIFAAGDVAD-PIY----RQLTTSVGAGTRAAMMAERYLAEEAEKVKG---------
THET801911      SEPGIFAAGDVAD-PIY----RQLTTSVGAGTRAAMMAERYLAEEAEKVKG---------
OCEP502115      SVPGVFAAGDVAD-PIY----KQLATSVGAGTRAAMTAEKWLMEQEQAEPASS-------
DEHLB00585      SVSGIYAAGDVRA-DSA----RQVVSAAGDGATAASYAQNYLSGIKTQQ-----------
DEHMG00439      SVSGILSAGDIRS-GSI----RQVISAAGDGAVAALSAKRYLDL----------------
DEHMB00497      SVSGILSAGDIRS-GSI----RQVISAAGDGAVAALSAKRYLDL----------------
DEHMC00427      SVSGILSAGDIRS-GSI----RQVISAAGDGAVAALSAKRYLDL----------------
THELD00726      SVEGIFAAGDVRD-KYL----RQVITAAGDGATAAMAAYAYIAEQLHLRQKV------FE
ANAMD00624      SVEGIFAAGDVRD-KFL----RQVVTAAGDGATAAMAAYSYVSEQLHLQKVL------FE
THEAS00815      SVEGIFAAGDVRD-TFL----RQVVTAAGDGAVAAMSAYSYITEQLHLQKIL------LE
AMICL00707      SVEGVFAAGDVRD-KNL----RQVVTAASDGAIAAMAASAYINEQVHLRSTL------LD
CLOCE01684      NIPGVFAAGDLRE-KYL----RQVITAAADGASAAYNAEKYIIENMKNIT----------
HYDS000616      SVSGIFAAGDCRS-GQT----GQVVVAAGEGCIAAMAAERYLQNSE--------------
HYDTT00224      SLEGVFAAGDCRS-GST----GQVAVAVGEGCIAGMQAEKYIEDNF--------------
PELTS01405      SQPGLYAAGDVRR-KLL----RQVVTAVADGAVAAVAAEKYLEEAH--------------
DESRL02179      GVPGLYAAGDVRQ-KSL----RQVVTATADGAIAAVEVEKYLAGH---------------
BORBP00506      SVDGVFSCGDVSN-KLY----AQAITAAAEGFIASVELGNFLK-----------------
BORAP00518      SVDGVFSCGDVSN-KLY----AQAITAAAEGFIASVELGNFLK-----------------
BORBU00514      SVDGVFSCGDVSN-KLY----AQAITAAAEGFIASVELGNFLK-----------------
BORBZ00490      SVDGVFSCGDVSN-KLY----AQAITAAAEGFIASVELGNFLK-----------------
BORBN00493      SVDGVFSCGDVSN-KLY----AQAITAAAEGFIASVELGNFLK-----------------
BORRA00486      SVNGVFSCGDVSN-KLY----AQAITAAAEGFLASVEVRNFLG-----------------
BORDL00498      SVNGVFSCGDVSN-KLY----AQAITAAAEGFLASVEVRNFLG-----------------
BORHD00497      SVKGVFSCGDVSN-KLY----AQAITAAAEGFLASVEVRNFLG-----------------
BORT900497      SVEGVFSCGDVSN-KLY----AQAITAAAEGFLASVELRNFLG-----------------
METKA01560      SLEGVYSAGDVTT-IPH----RNVPSAVYQGSVAGINAAEYALKSR--------------
MYCA500361      KIKNIFAAGDVVA-KDV----RQITTATSDGTIAAKTINSRIVK----------------
METVS00145      NVEGIYACGDVTG-GI-----LQVSKAVGEGVTAFNSALTYLQKMH--------------
METOI01431      NIDGIFACGDITG-GV-----LQVSKSVGEGAVALASASQYLNNYKNDK-----------
MYCHN00051      SVPGIYAAGDIIS-KNL----RQVVTATNDGAIAAISIKSYIDSLEESD-----------
MYCSL00465      NLKGIFAAGDVIN-KEL----RQIVTAMNDGAIAAIAIKNFIKSIQT-------------
MYCS300420      NLKGIFAAGDVIN-KEL----RQIVTAMNDGAIAAIAIKNFIKSIQT-------------
DESK101258      NLPGIFVAGDAAG-GPYKYRFEQIITAAADGAIAADAACKYICALKGPSST---------
STAHD01472      NLPGVYVAGDAAG-GQCKYRFEQIITAAAEGAKAADAAFKYILQNIKK------------
THEC100270      NLEGVFVAGDAAG-GPYKYRFEQIITAAADGAIAADAAFKYILSVKKT------------
NANEQ00478      NIQYVYAAGNITN-NCC--DLDQIVTSMAQGAIAAKSAYEDLLK----------------
CALLD01225      NIEGVFAAGDATS-KWK--GFRQIVTAAASGSIAAYSAYTYLTEKSSKR-----------
SULSO02155      SVPGVFAAGDCTS-AWL--GFRQVITAVAQGAVAATSAYRYVTEKKGKK-----------
```

SULS900210	SVPGVFAAGDCTS-AWL--GFRQVITAVAQGAVAATSAYRYVTEKKGKK-----------
SULIA00208	SVPGIFAAGDCTS-MWL--GFRQVITSVAQGAVAATSAYRYVTEKKGKK-----------
SULIM00207	SVPGIFAAGDCTS-MWL--GFRQVITSVAQGAVAATSAYRYVTEKKGKK-----------
SULIK00225	SVPGIFAAGDCTS-MWL--GFRQVITSVAQGAVAATSAYRYVTEKKGKK-----------
IGNH400907	NVPGVFAVGDVTG-IG-----FQIVIAAGHGATAALEASKYVKRIKRKVAVKQ-------
KORCO01040	NIPGVYAVGDVTG-EP-----LQIAKAVGDGVRAAVDIYDRIFGGSYARASSQG------
THESM01133	NIKGLFAAGDITN-VF-----KQIAVAVGQGAIAANSAKDILENWKSQMNEE--------
THEBM01534	KAPGIFAAGDITN-VF-----KQIAVAVGQGAIAANSAKEFIESWMEKNGA---------
METST01363	SVDGIYAAGDITG-GV-----KQVIVAAGQAAQAVTHISELLLI----------------
METTH00703	NVPLVYAAGDITG-GL-----NQWVTACAEGAIAATYAYREIQSY---------------
METSL02406	NIPGVYTAGDITG-GV-----NQLVVACGEGAVAAVNAYNYIKRTE--------------
METLA02406	NIPGVYTAGDITG-GV-----NQLVVACGEGAVAAVNAYNYIKRTE--------------
METPW00194	NVPHVYAAGDVTG-GL-----KQIIVACAEGAVAAESAYNDIKMEE--------------
RUBXD00226	SVPGVFAAGDVAD-RRY----RQAVTAAGDGCRAAIDAERWLEEQGEAQEAEDPGVWTAG
RHOM400178	NIPGVFACGDVQD-RVY----RQAVTAAGSGCMAAIDAERWLAEQEHAAASTTVAG----
GARV400051	SVEGVFAAGDVVD-NMY----RQAISAAGMGCRAALDAQTYLNEKYA-------------
BIFAP01644	SAPGVFAAGDVTD-KVY----RQAISAAGMGCRAALDAQEYLTSLDNR------------
SEGRD00014	STPGVFAAGDLVD-RHY----RQAITAAGSGCVAAIDAERWLAAAETAQAAPRHALSAQH
GORB404535	SIPGVFAAGDLVD-HTY----RQAITAAGSGCAASIDAERWLAEQGEAADLTVVADVTVD
GORPV04920	SLPGVFAAGDLVD-HTY----RQAITAAGSGCAAAIDAERWLAEQGDIDPTPVEADVLVV
MYCA904903	SVEGVFAAGDLVD-RTY----RQAVTAAGSGCAAAIDAERWLAETAHSQTLIEA------
MYCSS05356	SVEGVFAAGDLVD-RTY----RQAITAAGSGCSAAIDAERWLAEIGVAPSDQEISAPI--
MYCSJ05684	SVEGVFAAGDLVD-RTY----RQAITAAGSGCSAAIDAERWLAEIGVAPSDQEISAPI--
MYCSK05387	SVEGVFAAGDLVD-RTY----RQAITAAGSGCSAAIDAERWLAEIGVAPSDQEISAPI--
MYCS206581	SLDGVFAAGDLVD-HTY----RQAITAAGSGCAASIDAERWLAEQD--------------
MYCCN05158	SVEGVFAAGDLVD-HTY----RQAITAAGSGCAASIDAERWLADRAEPGERTSTTTD---
MYCVP05890	SVEGVFAAGDLVD-HTY----RQAITAAGSGCAASIDAERWLAEHTEPGERSSTTTD---
MYCGI00775	SVEGVFAAGDLVD-HTY----RQAITAAGSGCAAAIDAERWLAEHAEPGERTSTTTDDTA
MYCSR04994	SVEGVFAAGDLVD-HTY----RQAITAAGSGCAAAIDAERWLAEHAEPGERTSTTTDDSA
AMYSD04545	SLPGVFAAGDLVD-RTY----RQAITAAGTGCAAAIDAERWLAEHAASSAAADPAMALT-
MYCLE02681	SMDGVFAAGDLVD-RTY----RQAITAAGSGCAAAIDAERWLAEHAGSKANETTEETGDV
MYCLB02681	SMDGVFAAGDLVD-RTY----RQAITAAGSGCAAAIDAERWLAEHAGSKANETTEETGDV
MYCSD04323	SLPGVFAAGDLVD-RSY----RQAITAAGTGCTAAIDAERWLAEAADAAEAAMD------
MYCPA04306	SIPGVFAAGDLVD-RTY----RQAVTAAGSGCAAAIDAERWLAEHAESSAAAQGDATEFP
MYCA105023	SIPGVFAAGDLVD-RTY----RQAVTAAGSGCAAAIDAERWLAEHAESSAAAQGDATEFP
MYCUA04107	SLEGVFAAGDLVD-RTY----RQAVTAAGSGCAAAIDAERWLAEHEATGDA---------
MYCMM05378	SLEGVFAAGDLVD-RTY----RQAVTAAGSGCAAAIDAERWLAEHEATGDA---------
MYCA003905	SLPGVFAAGDLVD-RTY----RQAVTAAGSGCAAAIDAERWLAEHAATGEA---------
MYCTU03943	SLPGVFAAGDLVD-RTY----RQAVTAAGSGCAAAIDAERWLAEHAATGEA---------
MYCTF03846	SLPGVFAAGDLVD-RTY----RQAVTAAGSGCAAAIDAERWLAEHAATGEA---------
MYCTA03980	SLPGVFAAGDLVD-RTY----RQAVTAAGSGCAAAIDAERWLAEHAATGEA---------
MYCTK04010	SLPGVFAAGDLVD-RTY----RQAVTAAGSGCAAAIDAERWLAEHAATGEA---------
MYCTC03612	SLPGVFAAGDLVD-RTY----RQAVTAAGSGCAAAIDAERWLAEHAATGEA---------
MYCTD03548	SLPGVFAAGDLVD-RTY----RQAVTAAGSGCAAAIDAERWLAEHAATGEA---------
MYCCP03898	SLPGVFAAGDLVD-RTY----RQAVTAAGSGCAAAIDAERWLAEHAATGEA---------
MYCBO02863	SLPGVFAAGDLVD-RTY----RQAVTAAGSGCAAAIDAERWLAEHAATGEA---------
MYCBP03913	SLPGVFAAGDLVD-RTY----RQAVTAAGSGCAAAIDAERWLAEHAATGEA---------
MYCBT03913	SLPGVFAAGDLVD-RTY----RQAVTAAGSGCAAAIDAERWLAEHAATGEA---------
TSUPD04078	GVPGVFAAGDLVD-HTY----RQAITAAGSGCSAAIDAERWLAHHREAAVAQ--------
ACTMD06897	NVDGVFAAGDLVD-HTY----RQAITAAGSGCSAAIDAERWLAEHGAG-EAAVTAEHVGG
SACES08402	NLPGVFAAGDLVD-HTY----RQAITAAGSGCSAAIDAERWLAEHGTP-EAALAAEHVGG
SACVD03814	NLDGVFAAGDLVD-RTY----RQAITAAGSGCAAAIDAERWLAEHTTVAEASESSEFVGG
AMYMU09190	NLDGVFAAGDLVD-RTY----RQAITAAGSGCSAAIDAERWLAEHGDS-DAHEAAELVGG
AMYMS10178	NLDGVFAAGDLVD-RTY----RQAITAAGSGCSAAIDAERWLAEHGDS-DAHEAAELVGG
PSEUX06421	AVEGVFACGDLVD-HSY----RQAITAAGSGCSAAIDAERWLAEAPIPAELA--G----G
NOCFA05681	KIPGVFAAGDLVD-HTY----RQAITAAGTGCRAAIDAERWLAEQGDITDNTLDN----A
NOCCG05476	AVPGVFAAGDLVD-HTY----RQAITAAGTGCRAAIDAERWLAEQGDITSNTLDH----A
RHOE406010	SIDGVFAAGDLVD-HTY----RQAITAAGTGCSAAIDAERWLADRGDITANTLDA----A
RHOOB03468	SVDGVFAAGDLVD-HIY----QQAITAAGTGCSAAMDAERWLAERGDITANTVAA----A
RHOE104515	SAAGVFAAGDLVD-HTY----RQAITAAGTGCSAAIDAERWLADQGDITENTLAA----A
CORDI02302	NVPGVFAVGDLVD-NHY----QQAITAAGSGCRGAIDAEHYLAALNS-------------
CORD202219	NVPGVFAVGDLVD-NHY----QQAITAAGSGCRGAIDAEHYLAALNS-------------
CORDL02208	NVPGVFAVGDLVD-NHY----QQAITAAGSGCRGAIDAEHYLAALNS-------------
CORDJ02208	NVPGVFAVGDLVD-NHY----QQAITAAGSGCRGAIDAEHYLAALNS-------------
CORDH02221	NVPGVFAVGDLVD-NHY----QQAITAAGSGCRGAIDAEHYLAALNS-------------

```
CORD702316      NVPGVFAVGDLVD-NHY----QQAITAAGSGCRGAIDAEHYLAALNS-------------
CORD302337      NVPGVFAVGDLVD-NHY----QQAITAAGSGCRGAIDAEHYLAALNS-------------
CORDD02235      NVPGVFAVGDLVD-NHY----QQAITAAGSGCRGAIDAEHYLAALNS-------------
CORDV02170      NVPGVFAVGDLVD-NHY----QQAITAAGSGCRGAIDAEHYLAALNS-------------
CORDW02254      NVPGVFAVGDLVD-NHY----QQAITAAGSGCRGAIDAEHYLAALNS-------------
CORDK02230      NVPGVFAVGDLVD-NHY----QQAITAAGSGCRGAIDAEHYLAALNS-------------
COREF02870      NVPGVFAAGDLVD-SHY----QQAITAAGSGCRAAIDAENYLANQI--------------
CORGL03082      NLDGVFAAGDLVD-SHY----QQAITAAGSGCRAAIDAEHYLASLA--------------
CORGK02974      NLDGVFAAGDLVD-SHY----QQAITAAGSGCRAAIDAEHYLASLA--------------
CORGB03038      NLDGVFAAGDLVD-SYY----QQAITAAGSGCRAAIDAEHYLASLA--------------
CORK402006      SIPGVFAAGDVVD-SHY----QQAITAAGSGCRAALDVEHYLVDLRG-------------
CORJK02028      SLPGVFAAGDLVD-SHY----QQAITAAGSGCRAALDAEAYLATL---------------
CORVD02951      SLPGVFAAGDLVD-HHY----QQAITAAGSGCRAALDAEAYLAAL---------------
ARCHD01706      SLPGVFACGDVVD-STY----QQAVTAAGTGCVAALDAEAYLIRMEAEAAAQ--------
THET101205      NVDGVFAAGDVRD-HKY----RQAVTAAGDGCKAAMDAERWLESQGVQVDISDEIYSLPK
PROAC02247      NVPGVFACGDLVD-SHY----QQAVTAAGSGCRAALDAERFLTELGR-------------
PROAS02302      NVPGVFACGDLVD-SHY----QQAVTAAGSGCRAALDAERFLTELGR-------------
CAERE29798      NLAGVFAAGDLVD-HTY----RQAITAAGTGCAAAQDAQHYLSNLAPATVSAPELLA---
CELFA03749      NLPGVFAAGDVVD-HTY----RQAITAAGSGCAAALDAQHYLAGLADTAHDVVDPVPATV
JONDD02484      NLPGVFACGDLVD-HTY----RQAITAAGSGCSAALDAQHYLESLND-------------
XYLCX03324      NLPGVFACGDAVD-HTY----RQAITAAGSGCSAALDAQAYLTTLDDALVGAVAATEGVN
ACIC102144      SVPGVFACGDVVD-YIY----RQAVTAAGTGCAAAIDAERWLAEQDAGLSTSVLTTTGSA
FRADG04042      NLPGVFACGDVVD-HIY----RQAITAAGTGCAAALDAERHLAAREGEAGRPPRA-----
FRASU07065      NLDGVFAAGDVVD-HTY----RQAITAAGTGCAAALDAERWIAAHEESA-----------
FRASN07115      NIAGVFACGDVVD-HTY----RQAITAAGTGCSAALDAERYIAGLEGAADSAAEA-----
FRASC04424      NLDGVFACGDVVD-HTY----RQAITAAGTGCAAALDAERFIAAGEGPGEVTTVA-----
FRAAA06712      NLDGVFACGDVVD-HTY----RQAITAAGTGCAAALDAERFIAAGEGPGETTTEA-----
KYTSD02521      NLEGVFAAGDLVD-HTY----QQAITAAGSGCQAALDAERYLSTLDSSPVDTAQIEPAAD
KINRD04462      NVPGVFAAGDVVD-HEY----MQAITAAGMGCSAALDAEKYLAARGDAGLDAQ---PARE
NOCDD04789      NLEGVFAAGDVVD-HQY----RQAITAAGTGCSAALDAERYLAARGN-------------
NOCAA01941      NLEGVFAAGDVVD-HQY----RQAITAAGTGCSAALDAERFLAEHGN-------------
STRRD08913      NLDGVFAAGDVVD-HTY----RQAITAAGTGTAASLDAERWLSDHDDN------------
THECD04863      KIPGVFACGDVVD-HTY----RQAITAAGSGCAAAIDAERWLADQGEAELVHQ---PTA-
THEBD03528      NIEGVFAAGDVVD-HTY----RQAITAAGTGCAAALDAERWLAERA--------------
CATAD08894      SLPAVYACGDVVD-HTY----RQAITAAGTGCAAALDAERHLSELAHHQPVTV-------
KRIFD06917      NLPGVFAAGDLVD-HTY----RQAITAAGTGCSAALDAERFLATLEHVEQSAA---ATAA
KITSK03730      NVPGVFAAGDVVD-HTY----RQAITAAGTGCSAALDAERYLAALSDADEAPA---AALA
STRBB05366      NLPGVFAAGDVVD-HTY----RQAISAAGTGCSAALDAERYLAALADGANATA-------
STRVP03660      NLTGVFGAGDVVD-HTY----RQAITAAGTGCSAALDAERFLAALADSEKLAE---TPAV
STRSW04381      NLTGVFGAGDVVD-HTY----RQAITAAGTGCAAALDAERYLAALADGDQTAE---PEKT
STRGG03562      NLTGVFGAGDVVD-HTY----RQAITAAGTGCSAALDAERFLAALADAEPA-E---PEKT
STRFA03098      NLTGVFGAGDVVD-HTY----RQAITAAGTGCSAALDAERFLAALSDEKPA-E---PEKT
STRAW04303      NLSGVFGAGDVVD-HTY----RQAITAAGTGCSAALDAERFLSALAAAEDAAE---PEKT
STRCO02929      NLTGVFGAGDVVD-HTY----RQAITAAGTGCSAALDAERFLAALSD-EDKAE---PEKT
STRHJ05105      NLTGVFAAGDVVD-HTY----RQAITAAGTGCSAALDAERFLAALAD-EEKAE---PEKT
MONBE04991      SIPGVFACGDVQD-KKY----RQAITAAGTGCMSALEAEHYLAALEDSQ-----------
CHLRE01313      SVPGVFAAGDVQD-KKW----RQAITAAGTGCMAALEAEHFISAHEAEPEADGAKEPAAA
MEDTR25591      SVEGVFAAGDVQD-KKY----RQAITAAGSGCMAALDAEHFLQGVGLQQDKS-D------
SOLLC13750      SVRGVFAAGDVQD-KKY----RQAITAAGSGCMAALDAEHYLQEIGAQVGKS-D------
PRUPE10733      SVRGVFAAGDVQD-KKY----RQAVTAAGTGCMAALEAEHYLQEIGSQEGKR-D------
MANES18605      SVRGVFAAGDVQD-KKY----RQAVTAAGTGCMAALEAEHYLQEIGSQEGKS-D------
THECC00884      SVRGVFAAGDVQD-KKY----RQAVTAAGTGCMAALEAEHYLQEIGSQEGKS-D------
PHYPA31147      SVMGVFAAGDVQD-KKY----RQAITAAGTGCMAALEAEHYLQEHGMQEGKS-EDEPADL
AMBTC19471      SIRGVFAAGDVQD-KKY----RQAITAAGTGCMAALEAEHYLQEIGELEGKY-D------
MUSAC26038      SVKGVFAAGDVQD-KKY----RQAITAAGSGCMAALDAEHYLQEVGAQEGKT-D------
MUSAM33177      SVKGVFAAGDVQD-KKY----RQAITAAGSGCMAALDAEHYLQEVGAQEGKT-D------
SETIT03079      SVKGVFAAGDVQD-KKY----RQAITAAGSGCMAALDAEHYLQEVGAQEGKS-D------
ORYBR12195      SVEGVFAAGDVQD-KKY----RQAITAAGSGCMAALDAEHYLQEVAAQEGKA-D------
COCLU07729      SVEGVFAAGDVQD-KRY----RQAITSAGSGCIAALEAEKWLAERDDSVGNELETEN---
PHANO13702      SVEGVFAAGDVQD-KRY----RQAITSAGSGCVAALEAEKWLAEHDDSVGNELETEN---
PHAND10804      SVEGVFAAGDVQD-KRY----RQAITSAGSGCVAALEAEKWLAEHDDSVGNELETEN---
AURPU02089      NIEGVFAAGDVQD-KKY----RQAITSAGSGCIAALEAEKFLTENDA-SVDNSLENQ---
ZYMTR07711      NIPGVFAAGDVQD-KKY----RQAVTSAGSGCMAALEAEKFLAENEDVPVENGVEAD---
DICPU05926      SVEGVFACGDVQD-KIY----RQAITAAGSGCMAALDCERYLSH----------------
ENTHI00522      SVDGVFACGDVCD-RVY----RQAIVAAGSGCMAALSCEKWLQTH---------------
```

LEPBA02231	NVEGVFAAGDVQD-KVY----RQAITAAGSGCMAALEAERWLEGH---------------
LEPBP02301	NVEGVFAAGDVQD-KVY----RQAITAAGSGCMAALEAERWLEGH---------------
LEPBL01462	SIEGIFAAGDVQD-KIY----RQAVSAAGSGCMAALDAERWLESREE-------------
LEPBJ01276	SIEGIFAAGDVQD-KIY----RQAVSAAGSGCMAALDAERWLESREE-------------
LEPIN02475	NIEGIFAAGDVQD-KIY----RQAVSAAGSGCMAALDAERWLESREEE------------
LEPII01996	NIEGIFAAGDVQD-KIY----RQAVSAAGSGCMAALDAERWLESREEE------------
LEPIC01426	NIEGIFAAGDVQD-KIY----RQAVSAAGSGCMAALDAERWLESREEE------------
SPIAZ00697	NVPGVFACGDVQD-KHY----RQAVTAAGSGCMAALDAEKFLIEE---------------
PENRW10140	NVEGVFACGDVQD-KRY----RQAITSAGSGCVAALEAEKFIAETETHQEAKPVL-----
PENCH09104	NVEGVFACGDVQD-KRY----RQAITSAGSGCVAALEAEKFIAETETHQEAKPVL-----
EURHE07269	SREGVFACGDVQD-KRY----RQAITSAGSGCIAALEAEKFIAEAESESPSQAETQATTK
ASPAC07301	SVEGVFACGDVQD-KRY----RQAITSAGSGCIAALEAEKYIAEKESNDEEPVTSVE---
EMENI10387	SVEGVFACGDVQD-KRY----RQAITSAGSGCIAALEAERFIGESESNEEIPPAHANPAL
EMEND02596	SVEGVFACGDVQD-KRY----RQAITSAGSGCIAALEAERFIGESESNEEIPPAHANPAL
ASPTN06742	SLEGVFACGDVQD-KRY----RQAITSAGSGCMAALDAEKFIAEHESPEEPAPVIETEKS
ASPCL04014	SREGVFACGDVQD-KRY----RQAITSAGSGCIAALEAEKFIAESEGPEEEAVV---SKT
ASPFU05647	SREGVFACGDVQD-KRY----RQAITSAGSGCIAALEAEKFIAEAESPEEEPVAVSAQKS
NEOFI00452	SREGVFACGDVQD-KRY----RQAITSAGSGCIAALEAEKFIAEAEGPEEEPVAVSAQKS
CRYPA10563	NIEGVYAAGDVQD-KRY----RQAITSAGSGCMAALEAEKFLSEHEEAEAPVTAEQ----
BLUGR03498	NVEGVFAAGDVQD-KRY----RQAITSAGSGCIAALEAERFLAEHEDVRENGDQ------
SCLS112814	NVEGVFAAGDVQD-KRY----RQAITSAGSGCIAALEAEKFLAEQEDGENDLEKTD----
MAGGR04266	SVEGVFACGDVQD-KRY----RQAITSAGSGCMAAMDAEKFLAEQEDVEPDREARM----
NEUCR01575	SVEGVFAAGDVQD-KRY----RQAITSAGTGCMAALDAEKFLSEHEETPAEHRDTS----
NEUT908941	SVEGVFAAGDVQD-KRY----RQAITSAGTGCMAALDAEKFLSEHEETPAEHRDTS----
VERDA02342	NIEGVFAAGDVQD-KRY----RQAITSAGSGCQAALDAEKYISEMEDPNLKDVVNH----
COLSU12486	NIEGVFAAGDVQD-KRY----RQAITSAGTGCMAAMDAEKYLAELEDGDAKDPNNN----
HYPAI01684	SIPGVFAAGDVQD-KRY----RQAITSAGTGCMAALEAEKYLTELE--------------
HYPVG06080	SIPGVFAAGDVQD-KRY----RQAITSAGTGCMAALEAEKYITEME--------------
HYPJE05895	SVPGVFAAGDVQD-KRY----RQAITSAGTGCMAALEAEKYITEME--------------
NECHA05020	SIEGVFAAGDVQD-KRY----RQAITSAGTGCMAALDAEKFISEHEDQPN----------
FUSO415847	NVEGVFAAGDVQD-KRY----RQAITSAGTGCMAALEAEKFLADHEDDQRADDRPN----
GIBZA01026	NVEGVFAAGDVQD-KRY----RQAITSAGTGCMAALEAEKFLADHEDDERADERPN----
SCHPO04025	SIPGFFAAGDVQD-KVF----RQAITSAGSGCQAALLAMHYLEELEDTD-----------
YARLI03635	SVKGVFAAGDVQD-KRY----RQAITSAGTGCMAALDCEKLLAEEE--------------
ASHGO00946	NVPGLFAAGDVQD-SRY----RQAVTSAGSGCMAALDAEKFLSELE--------------
KLULA02190	NVPGVFAAGDVQD-ARY----RQAITSAGSGCMAALDAEKYITELE--------------
ZYGRO00676	NVPGFFAAGDVQD-SKY----RQAITSAGTGCMAGLDAERYLSSLE--------------
DEKBR01813	SVPGVFAAGDVQD-KKY----RQAITSAGTGCMAALDCEHYLSSLEA-------------
PICPG04776	SIPGVFAAGDVQD-KRY----RQAITSAGTGCMAALDAEKFLSENE--------------
CANTE00916	SVPGVFAAGDVQD-KIY----RQAITSAGSGCQAALDCESFLAEQSV-------------
LODEL03891	SVAGVFAAGDVQD-KIY----RQAITSAGTGCMAALECEKFIAEQED-------------
DEBHA05546	SIPGVFAAGDVQD-KRY----RQAITSAGTGCMAALDCEKFLSEEEAK------------
SPAPN03477	SVPGVFAAGDVQD-KIY----RQAITSAGTGCMAALECEKFISEQE--------------
CANAW04800	SIEGVFAAGDVQD-KIY----RQAITSAGSGCMAALECEKFISEQEA-------------
PICST04701	SIEGVYAAGDVQD-KKY----RQAITSAGTGCMAALDCEKFLSEQE--------------
PUCGT10887	SIPGVFAAGDVQD-KKY----RQAITSAGTGCIAALECERFLAEEEAMDRDG------EP
PUCGR11813	SIPGVFAAGDVQD-KKY----RQAITSAGTGCIAALECERFLAEEEAMDRDG------EP
PHYBL11006	NIVGLFAAGDVQD-KRY----RQAVTSAGSGCMAALDAERYLSELEAEE-----------
USTMA03757	SVKGVFAAGDVAD-KKY----RQAITSAGTGCIAALEAERLLAEEEVLDPVDAAQ-----
USTHO04132	SVKGVFAAGDVAD-KKY----RQAITSAGTGCIAALEAERLLAEEEILDPVDAAA-----
WALSE04527	NIKGLFAAGDVQD-KRY----RQAITSAGSGCMAALECERLLAEEEVEESA---------
TREME07701	SVKGVFAAGDVQD-KRY----RQAITSAGSGCMAALEAERLISEEEAEDGEID---TADV
AURST04751	SVRGVFAAGDVQD-KIY----RQAITSAGSGCMAALEAERFLAEEEEGIE----------
FOMME10177	SVKGVFAAGDVQD-KRY----RQAVTSAGSGCMAALEAERLLAEEEAGEF---------
CONPW06392	SVKGVFAAGDVQD-KRY----RQAITSAGSGCMAALEVERLLTEEEEGMDE---------
STEHR07076	SVKGVFAAGDVQD-KRY----RQAITSAGSGCMAALEVEQLIAEEEEELGEE---------
HETAN06295	SVYGVFAAGDVQD-KRY----RQAITSAGSGCMAALEVEKLIAEEEEIGE----------
GLOTR06982	SVKGVFAAGDVQD-KKY----RQAITSAGSGCMAALEVEKLLAEEEEMLEEEWRSENMKV
PUNST01981	SVKGVYAAGDVQD-KRY----RQAITSAGSGCMAALEVERLLAEEEEEELEEWRAEGEGH
LACBI02877	SVKGVYAAGDVQD-KKF----RQAITSAGSGCMAALEVEKLLAEEEEF------------
COPCI16429	SVKGVFAAGDVQD-KRY----RQAITSAGSGCMAALEVEKLIAEEEELGEY---------
DICSQ11618	SVKGVFAAGDVQD-KRY----RQAITSAGSGCMAALEAERLIAEEEEGIED---------
TRAVS13180	SVKGVFAAGDVQD-KRY----RQAITSAGSGCMAALEAERLIAEEEEGIED---------
WOLCO03584	SVKGVFAAGDVQD-KRY----RQAITSAGSGCMAALEAERLIAEEEEGIED---------
FOMPI05979	SVKGVFAAGDVQD-KRY----RQAITSAGSGCMAALEAERLIAEEEEGLED---------

```
PHLGI10219      SVKGVFAAGDVQD-KRY----RQAITSAGSGCMAALEAERLIAEEEEGVEED--------
PHACH05757      SVRGVFAAGDVQD-KRY----RQAITSAGSGCMAALEAERLIAEEEEGVEE---------
RICTY00419      SVEGVFAAGDVQD-KIY----RQAITAAASGCMAALEVAKFLNK----------------
RICPR00429      NVEGVFAAGDVQD-KIY----RQAVTAAASGCMAALEVAKFLNK----------------
RICPP00461      NVEGVFAAGDVQD-KIY----RQAVTAAASGCMAALEVAKFLNK----------------
RICBR00434      NIEGVFAAGDVQD-KIY----RQAITAAGTGCMAALEAEKFLNK----------------
RICB800988      NIEGVFAAGDVQD-KIY----RQAITAAGTGCMAALEAEKFLNK----------------
RICCK00600      SVEGVFAAGDVQD-KIY----RQAVTAAGSGCMAALEAEKFLNN----------------
RICAH00614      NVEGVFAAGDVQD-KIY----RQAVTAAGTGCMAALEAEKFLNK----------------
RICAC00782      NVEGVFAAGDVQD-KIY----RQAVTAAGTGCMAALEAEKFLNK----------------
RICFE00656      SVEGVFAAGDVQD-KIY----RQAVTAAGSGCMAALEVEKFLNK----------------
RICMS00042      SVEGVFAAGDVQD-KIY----RQAVTAAGTGCMAALEAEKFLNK----------------
RICM500453      SVEGVFAAGDVQD-KIY----RQAVTAVGTGCMAALEAETFLNK----------------
RICR300665      SVEGVFAAGDVQD-KIY----RQAVTAVGTGCMAALEAEKFLNK----------------
RICAG00651      SVEGVFAAGDVQD-KIY----RQAVTAAGTGCMAALEAEKFLNK----------------
RICP300630      SVEGVFAAGDVQD-KIY----RQAVTAAGTGCMAALEAAKFLNK----------------
RICRS00632      SVEGVFAAGDVQD-KIY----RQAVTAAGTGCMAALEAEKFLNK----------------
RICRO00663      SVEGVFAAGDVQD-KIY----RQAVTAAGTGCMAALEAEKFLNK----------------
RICCN00618      SVEGVFAAGDVQD-KIY----RQAVTAAGTGCMAALEAEKFLNK----------------
RICPT00620      SVEGVFAAGDVQD-KIY----RQAVTAAGTGCMAALEAEKFLNK----------------
RICAE00497      SVEGVFAAGDVQD-KIY----RQAVTAAGTGCMAALEAEKFLNK----------------
RICJY00467      SVEGVFAAGDVQD-KIY----RQAVTAAGTGCMAALEAEKFLNK----------------
RICPU00072      SVEGVFAAGDVQD-KIY----RQAVTAAGTGCMAALEAEKFLNK----------------
RICS100537      SVEGVFAAGDVQD-KIY----RQAVTAAGTGCMAALEAEKFLNK----------------
BARBK00891      SIPGVFAAGDVAD-NVF----RQAITAAGRGCMAALEVERFLDLQT--------------
BARVW00932      SIAGVFAAGDVAD-EVF----RQAVTAAGRGCMAALEAERFLDLQA--------------
BART100617      SIAGVFAAGDVTD-ETF----RQAVTAAGRGCMAALEAERFLDLSKR-------------
BARGA00466      SIAGVFAAGDVTD-EIF----RQAVTAAGRGCMAALEAERFLDISKR-------------
BARHE01107      SIAGVFAAGDVVD-DVF----RQAITAAGRGCMAALEAERFLELQI--------------
BARQU00895      SIAGVFAAGDVVD-NIF----RQAITAAGMGCMAALEAERFLDLQI--------------
OCHA401642      DVPGIFAAGDVTD-DIY----RQAVTAAGLGCMAALEAERWLAAQEPLHEAAE-------
BRUAB01390      DVPGIFAAGDVTD-DIY----RQAVTAAGMGCMAALEAERWLAAQEPLHEAAE-------
BRUA201507      DVPGIFAAGDVTD-DIY----RQAVTAAGMGCMAALEAERWLAAQEPLHEAAE-------
BRUA101339      DVPGIFAAGDVTD-DIY----RQAVTAAGMGCMAALEAERWLAAQEPLHEAAE-------
BRUSU01458      DVPGIFAAGDVTD-DIY----RQAVTAAGMGCMAALEAERWLAAQEPLHEAAE-------
BRUME00510      DVPGIFAAGDVTD-DIY----RQAVTAAGMGCMAALEAERWLAAQEPLHEAAE-------
BRUSI01461      DVPGIFAAGDVTD-DIY----RQAVTAAGMGCMAALEAERWLAAQEPLHEAAE-------
BRUC201446      DVPGIFAAGDVTD-DIY----RQAVTAAGMGCMAALEAERWLAAQEPLHEAAE-------
BRUMC01440      DVPGIFAAGDVTD-DIY----RQAVTAAGMGCMAALEAERWLAAQEPLHEAAE-------
BRUMB01421      DVPGIFAAGDVTD-DIY----RQAVTAAGMGCMAALEAERWLAAQEPLHEAAE-------
BRUM501487      DVPGIFAAGDVTD-DIY----RQAVTAAGMGCMAALEAERWLAAQEPLHEAAE-------
BRUO201285      DVPGIFAAGDVTD-DIY----RQAVTAAGMGCMAALEAERWLAAQEPLHEAAE-------
RHILO01973      DVPGVFAAGDVTD-DVY----RQAVTAAGLGCMAALEAEKYLAGIEVHREAAE-------
CHESB02097      DVPGVFAAGDVAD-DVY----RQAVTAAGLGCMAALEAERYLAELETHREAAE-------
METPB00996      EIPGVFAAGDVTD-DVY----RQAITAAGMGCMAALEAEKFLANLDVGESPAKAAAE---
METEP01063      DIPGVFAAGDVTD-DVY----RQAITAAGMGCMAALEAEKFLANLDVGESPAKAAAE---
METEA00810      DIPGVFAAGDVTD-DVY----RQAITAAGMGCMAALEAEKFLANLDVGESPAKAAAE---
METED01453      DIPGVFAAGDVTD-DVY----RQAITAAGMGCMAALEAEKFLANLDVGESPAKAAAE---
METS403554      EIPGVFAAGDVTD-DVY----RQAITAAGMGCMAALEAEKYLANLAIGETPRRAAAE---
METNO05482      EIPGVFAAGDVTD-DVY----RQAVTAAGMGCMAALEAEKYLANLAIGETPRQAAAE---
METSZ03234      SMPGVYAAGDVAD-DVY----RQAVTAAGLGCMAALEVEKFLLDAPKP---AVKEIA---
BEII900056      NIPGVFAAGDVTD-DIY----RQAVTAAGMGCMAALEAERWLLAEGEL---RHAAAE---
METSB02743      NIPGVFAAGDVTD-DIY----RQAVTAAGMGCMAALEAERWLAAEEHR---RIAAE----
MAGMM00401      NIEGVFAAGDVQD-TVF----RQAITAAGTGCMAAIEAERWLAAHA--------------
HYPNA00542      AIPGVFAAGDVTD-ETY----RQAVTAAGMGCMAALDAERFLSEQEAHVEAAVPAE----
KETVY00902      SVPGVFAAGDLTD-DKY----RQAITSAGMGCQAALDAEHWLQAQG--------------
KETVW00472      SVPGVFAAGDLTD-DKY----RQAITSAGMGCQAALDAEHWLQAQG--------------
ROSDO03242      SIPGVFAAGDLTD-HIY----RQAVTSAGMGCMAALEAEKFLAAQDAEAQADEGVAAE--
ROSLO02615      SIPGVFAAGDLTD-HIY----RQAVTSAGMGCMAALEAEKFLAENETVARTDEKVAAAE-
RUEPO00888      SIPGIFAAGDLTD-HKY----RQAVTSAGMGCMAALDAERFLAEQE--------------
RUEST00613      SIPGIFAAGDLTD-HKY----RQAVTSAGMGCMAALDAERFLAEQE--------------
PHAIB02390      SIPGIFAAGDLTD-HKY----RQAVTSAGMGCMAALDAERFLAEQE--------------
PARDP02130      SIPGVFAAGDLTD-HIY----RQAVTSAGMGCMAALDAERFLAEHDMAGEPNPHAAEIPA
DINSH02620      SIPGVFAAGDLTD-HEY----RQAVTSAGMGCMAALEAERFLAEMDGETTADAGVYAAPV
RHOCB02768      GIPGVFAAGDLTD-HTY----RQAITSAGMGCMAALDAERWLAEQG--------------
```

```
RHOS500248      SIPGVFAAGDLTD-HVY----RQAVTSAGMGCMAALDAEHFLASQ---------------
RHOS400150      SIPGVFAAGDLTD-HVY----RQAVTSAGMGCMAALDAERFLAGA---------------
RHOS100227      SIPGVFAAGDLTD-HVY----RQAVTSAGMGCMAALDAERFLAGA---------------
RHOSK02952      SIPGVFAAGDLTD-HVY----RQAVTSAGMGCMAALDAERFLAGA---------------
MIDMI00790      SIPGVFAAGDAQD-KIY----RQAVTAAGTGCMAALDALKYLQE----------------
ACEP301595      SVPGVFAAGDVQD-KTY----RQAVTAAGTGCMAALDAERYLAGLA--------------
MICAA01566      NIPGVFAAGDVKD-KTF----RQAVTAAGMGCMAALEADRYLAALESDHRAQAAE-----
TISMK03676      SIPGVFAAGDVQD-KVF----RQAVTAAGTGCMAALEAERFLAGTTHAPADAVAE-----
AZOL402409      DIPGVFAAGDVKD-KIY----RQAVTAAGMGCMAALEAERWLAHHEPAAG-------AHG
PSEUV04348      SIPGVYAAGDVTD-EHY----RQAVTAAGMGCMAALEAERYLAALEAETEAAE-------
HIRBI01095      SVPGVYAAGDVTD-DKF----RQAVTAAGMGCMAALESDHFLAELELNNK----------
PARL102241      SIPGVFAAGDVTD-DIY----RQAVTAAGMGCMAALESERWLAGKDAEKAGIGPDPVERA
MARMM02139      SIPGVFAAGDVTD-DVY----RQAVTAAGMGCMAALDAERFLASQE--------------
PHEZH02588      AIQGVYAAGDVTD-DVY----RQAVTAAGMGCMAALEAVRFLAEEDHRKAHH---PISHG
CAUCR02826      AIEGVYAAGDVTD-DVY----RQAVTAAGMGCMAALEAVRFLAEEDHKAAHH---PISHA
CAUCN02939      AIEGVYAAGDVTD-DVY----RQAVTAAGMGCMAALEAVRFLAEEDHKAAHH---PISHA
CAUST00891      AIKGVYAAGDVTD-DVY----RQAVTAAGMGCMAALEAVRFLAEEDHKKAHH---PISHA
PARBH01522      SVPGVYAAGDVTD-ETY----RQAVTAAGMGCMAALEAEKFLAGMAIAEAAE--------
PELHB02084      SVPGVFAAGDVTD-DVY----RQAVTAAGMGCMAALEAERYLAAHEMAEAAE--------
HYPDA03273      AIPGVFAAGDVKD-EVF----RQAVTAAGMGCMAALEAERYLAKVEAQADAAE-------
HYPSM04642      AIPGVFAAGDVKD-EVF----RQAVTAAGMGCMAALEAERYLAQMEDQADAAE-------
OLICO01107      SVPGVFAAGDVAD-EHY----RQAVTAAGMGCMAALETERFLAARASERAAAE-------
OLICM02790      SVPGVFAAGDVAD-EHY----RQAVTAAGMGCMAALETERFLAARASERAAAE-------
RHOPS01446      SIPGLFAAGDVAD-ETY----RQAVTAAGLGCMAALEAERFLALRASDREAAE-------
RHOPA04062      SVPGVFAAGDVAD-ETY----RQAVTAAGMGCMAALEAERFLALRAGDRQAAE-------
RHOPT04533      SVPGVFAAGDVAD-ETY----RQAVTAAGMGCMAALEAERFLALRAGDRQAAE-------
RHOPX04235      SVPGVFAAGDVAD-ETY----RQAVTAAGMGCMAALEAERFLALRAGDRQAAE-------
BRADU07321      SVPGLFAAGDVAD-ETY----RQAVTAAGLGCMAALEAERFLALRASERAAAE-------
BRASO01239      SVPGVFAAGDVAD-ETY----RQAITAAGMGCMAALEAERFIAHAASERAAAE-------
BRASB06352      SVPGVFAAGDVAD-ETY----RQAITAAGMGCMAALEAERFLAHAASERAAAE-------
RHOPB03773      SVPGLFAAGDVAD-ETY----RQAVTAAGLGCMAALEAERFLAASASERVAAE-------
NITWN02324      SVPGLFAAGDVAD-EVY----RQAVTAAGLGCMAALEAERFLAAHAGDRAAAE-------
NITHX02707      SVPGLFAAGDVAD-EVY----RQAVTAAGLGCMAALEAERFLAAHASDRAAAE-------
AZOC501143      SVPGVFAAGDVAD-DVF----RQAVTAAGRGCMAALEAERFLAQHEVAEAAE--------
XANP202679      SVPGVFAAGDVAD-DVY----RQAVTAAGRGCMAALEAERFLAHQGVAKAAE--------
CHLTF01983      SVPGVFAAGDVQD-FTY----RQAVTAAGTGCMAAIDAERWLEEQAHAAH----------
IGNAJ02236      NVEGVFAAGDVAD-KKY----RQAITAAGTGCMAALDAERWLEAQES-------------
MELRP00403      SVEGVFAAGDVAD-KKY----RQAITAAGTGCMAALDAQHWLEEKELA------------
ANADF00468      SVPGVFAAGDVAD-PSY----RQAISAAGSGCMAAIDAERFLGEHTTETWD---------
CHLCH01125      NVPGVFACGDVQD-YTY----RQAVTAVGTGCMAAVDAERYLETIR--------------
PELPB01419      NVPGVFACGDVQD-FTY----RQAVTAVGTGCMAAVDAERFLETIR--------------
CHLL200781      SVQGVFACGDVQD-YTY----RQAVTAVGSGCMAAVDAERFLESIR--------------
CHLTE00830      SVKGVFACGDVQD-FTY----RQAVTAVGTGCMAAIEAERFLESIR--------------
CHLP800854      SVKGVFACGDVQD-FTY----RQAVTAVGTGCMSAIEAERFLESIR--------------
CHLL701225      SVQGVFACGDVQD-FIY----RQAVTAVGTGCMAAVDAERYLETIR--------------
CHLPM00993      SVAGVFACGDVQD-YTY----RQAVTAVGTGCMAAVDAERFLESIR--------------
WOLTR00562      SRAGVFAAGDVQD-KVY----RQAVVAAGTGCMAALDAEKFLECTSK-------------
WOLPP00135      SRAGVFAAGDVQD-KVY----RQAVVAAGTGCMAALDAEKFLES----------------
WOLPM00650      SRAGVFAAGDVQD-KVY----RQAVVAAGTGCMAALDAEKFLES----------------
WOLWR00416      SRAGVFAAGDVQD-KVY----RQAVVAAGTGCMAALDAEKFLES----------------
EHRCR00696      SHPGIFAAGDVQD-KIY----RQAVVAAGSGCMAALEAAKFLSEQ---------------
ANAMM00345      SCPGIFAAGDVRD-KVY----RQAVVAAGTGCMAALDAEKFLSERGITS-----------
ANAMF00341      SCPGIFAAGDVRD-KVY----RQAVVAAGTGCMAALDAEKFLSERGITS-----------
ANAPZ00610      SCPGIFAAGDVCD-KVY----RQAVVAAGSGCMAALDAEKYLAEKGIE------------
NEOSM00537      SVSGIFACGDVQD-PVY----RQAVTAAGTGCMAALDAIRFLDCI---------------
NEORI00513      SVSGIFACGDVQD-PVY----RQAVTAAGTGCMAALDAMKFLDCI---------------
PELUB00076      NIPGVYAAGDVKD-KTF----RQAVTAAGMGCMAALEAEKFLSH----------------
PELSM00736      NIKGIFAAGDVKD-KTF----RQAVTAAGMGCMAALEAEKLLSEKN--------------
PUNMI01409      SVDGIFAAGDCVD-KIY----RQAVTAAGMGCMAALDAERWLGEQS--------------
ASTEC00651      AIKGVFAAGDVTD-DVY----RQAVTAAGMGCMAALEAIRLLAEEDHHRQDAKIGAW---
ZYMMT00154      SVEGVFACGDVMD-KHY----RQAVTAAGTGCMAALDAERFLGEIDFK------------
ZYMMO00984      SIKGIFACGDVMD-KHY----RQAVTAAGTGCMAALEAERFLGEIDFKED----------
ZYMMA00176      SIKGIFACGDVMD-KHY----RQAVTAAGTGCMAALEAERFLGEIDFKED----------
ZYMMN00183      SIKGIFACGDVMD-KHY----RQAVTAAGTGCMAALEAERFLGEIDFKED----------
SPHAL00143      AIPGVFAAGDVTD-KIY----RQAVTAAGMGCMAALDAERYLAEAEYHAMVDA-------
```

SPHWW03978	SVPGVFGAGDVSD-KVY----RQAVTAAGMGCMAALDVERFLAEADFEARATVAA-----
SPHJU02313	AIPGVFAAGDVSD-KVY----RQAVTAAGMGCMAALDVEKFLAEADFEAVAAE-------
NOVAD02319	AIPGVFACGDVMD-HTY----RQAVTAAGTGCMAALDAERFLAELDFKAAQAVIA-----
ERYLH00934	AIPGVFAAGDVMD-HIY----RQAVTAAGVGCMAALDAERFLANLEIDIDGHVHAPQTEA
GRABC00720	SIPGVFAAGDVQD-KIY----RQAVTAAGTGCMAALEAEKWVAEHTDSSMLAAE------
GLUDA03075	SVPGVFAAGDVQD-RIY----RQAVTAAGTGCMAALEAERYLAGMSH-------------
KOMMN00774	SVPGVFAAGDVQD-KIF----RQAVTAAGTGCMAALEAERFLAATV--------------
HALVD01097	DVPGIFAAGDVVD-YHY----QQAATAGGMGVKAALDADDYLEELEREEKQAAAGAAE--
HALHT01699	DVDGIFGAGDVVD-YHY----QQAVTAAGMGCKAAIDADEYLESQAEAAAEAESEAAAEA
METI401038	TVPGVFACGDCVD-GLY----RQAVTAAGMGCMAALDAERYLRGIEA-------------
PLAL201612	SVEGVFAAGDVAD-SYY----RQAITSAGTGCMSALDAERWLASQGIH------------
GEMAT01983	TVDGVFAAGDVID-DYY----RQAITSAGTGCMAALEAERWLAHHGIGESPVLETGESTI
CYAAP02456	NVEGVWAAGDVQD-HHY----RQAITAAGTGCMAALEAERWLSQKGSLSVPSLV------
CHLPN00303	SVPGVFAAGDVQD-KYY----RQAVTSAGSGCIAALDAERFLG-----------------
CHLPP00439	SVPGVFAAGDVQD-KYY----RQAVTSAGSGCIAALDAERFLG-----------------
CHLPE00727	SIPGVFAAGDVQD-KHY----RQAITSAGTGCMAALEAERFLS-----------------
CHLTR00101	SVPGVFAAGDVQD-KYY----RQAITSAGSGCMAALDAERFLEN----------------
CHLTA00102	SVPGVFAAGDVQD-KYY----RQAITSAGSGCMAALDAERFLENRALCLV----------
CHLTJ00101	SVPGVFAAGDVQD-KYY----RQAITSAGSGCMAALDAERFLEN----------------
CHLTD00098	SVPGVFAAGDVQD-KYY----RQAITSAGSGCMAALDAERFLEN----------------
CHLT700102	SVPGVFAAGDVQD-KYY----RQAITSAGSGCMAALDAERFLEN----------------
CHLT000101	SVPGVFAAGDVQD-KYY----RQAITSAGSGCMAALDAERFLEN----------------
CHLT500104	SVPGVFAAGDVQD-KYY----RQAITSAGSGCMAALDAERFLEN----------------
CHLTL00098	SVPGVFAAGDVQD-KYY----RQAITSAGSGCMAALDAERFLEN----------------
CHLTG00102	SVPGVFAAGDVQD-KYY----RQAITSAGSGCMAALDAERFLEN----------------
CHLTS00101	SVPGVFAAGDVQD-KYY----RQAITSAGSGCMAALDAERFLEN----------------
CHLT900102	SVPGVFAAGDVQD-KYY----RQAITSAGSGCMAALDAERFLEN----------------
CHLTZ00101	SVPGVFAAGDVQD-KYY----RQAITSAGSGCMAALDAERFLEN----------------
CHLT400432	SVPGVFAAGDVQD-KYY----RQAITSAGSGCMAALDAERFLEN----------------
CHLT100103	SVPGVFAAGDVQD-KYY----RQAITSAGSGCMAALDAERFLEN----------------
CHLT200350	SVPGVFAAGDVQD-KYY----RQAITSAGSGCMAALDVERFLEN----------------
CHLTB00350	SVPGVFAAGDVQD-KYY----RQAITSAGSGCMAALDVERFLEN----------------
CHLTC00378	SVPGVFAAGDVQD-KYY----RQAITSAGSGCMAALDVERFLEN----------------
SIMNZ00831	NVEGVFAAGDVQD-KVY----RQAISAAGSGCMAALDAERFLVAKGINV-----------
PARUW01706	SIKGVFAAGDVQD-FEY----RQAITAAGSGCMAALDAERWLAEKGLDN-----------
WADCW00943	SKAHVYACGDVQD-FTY----RQAITAAGTGCMAALEAERELAEKGHLD-----------
SINAD04980	SVEGVFAAGDVVD-THY----RQAITAAGSGCAAAIDCEKWLESIHG-------------
BIFLB01558	SVPGVFAAGDCAD-AIY----QQAIVAAGSGCRAALDVQSYLEDIPEGI-----------
BIFAB00498	SVPGVFAAGDCAD-AIY----QQAIVAAGSGCRAALDVQSYLEDIPEGI-----------
BIFAV01561	SVPGVFAAGDCAD-AIY----QQAIVAAGSGCRAALDVQSYLEDIPEGI-----------
BIFAS01557	SVPGVFAAGDCAD-AIY----QQAIVAAGSGCRAALDVQSYLEDIPEGI-----------
BIFA001518	SVPGVFAAGDCAD-AIY----QQAIVAAGSGCRAALDVQSYLEDIPEGI-----------
BIFBA01814	SAPGVFAAGDCVD-RTY----RQAISAAGMGCRAALDAQQYLTA----------------
BIFAA01619	SEPGVFAAGDCMD-SVY----RQAISAAGMGCRAALDAQAYLDSLK--------------
BIFDB02112	SAPGIFAAGDCVD-SVY----RQAISAAGMGCRAALDAQSYLQ-----------------
TERSS03091	VIPGVFAAGDVQD-RKY----RQAITAAGTGCMAALEVEKYLEEHGR-------------
GRATM03160	ILPGVFACGDIKD-RKY----RQAITAAGSGCMAALEVEKYLEEHGR-------------
GRAMM03836	QLHGVYACGDVQD-RRY----RQAITAAGTGCMAALEVEKYLEETGR-------------
AKKM801247	RTPGLFAAGDVAD-PHY----RQAISSAGMGCSAAIEAERYLLGL---------------
OPITP04130	KVPGVYVAGDCAD-HVY----RQAITAAGMGCQAAIEAERWLAEHGT-------------
CORAD01794	KVPGLFAAGDCVD-HVY----RQAISAAGMGCQAAIEAERWLLEQS--------------
BUCCC00180	NIPGIFAAGDVSD-SIY----QQAITASASGCMAAIDAEKYLNNISKKKTLFNIN-----
CENSY00347	SVPGVFAAGDVHD-RRY----RQAITAAGFGCMAGIDVNNYLDE----------------
NITMS00668	NVEGVFAAGDVHD-RSY----RQAITAAGYGCMAAIDVDKYLTESADNQE----------
MEIRD02823	SVPGLFAAGDVAD-PIY----RQLSTSVGAGTRAAMMAERYLAEQEHAAVH---------
HERA203780	NVPGVFVAGDVED-HVY----RQAITAAGSGCMAAMEASWFLDQIEHEQTSLAQW-----
CHLAA02018	NIPGIFAAGDVTD-HIY----RQAVTAAGDGCRAAMEATWYLAEQEHARSKATVSVA---
CHLSY02175	NIPGIFAAGDVTD-HIY----RQAVTAAGDGCRAAMEATWYLAEQEHARSKATVSVA---
CHLAD02599	NIPGIFAAGDVAD-HIY----RQAVTAAGDGCRAAMEATWYLAEQEHARAKATATASA--
CALAS01745	NVPGVFAAGEIQD-KVF----KQVATSVGQGCAAAMSATRFLEELEDGRAVDLRTDPREF
ANATU01696	SVPGVYAAGEAAD-PIY----RQVITSAGMGAAAAMQAAKFLEEHD--------------
SULMS00235	NIPGVFAAGDVKD-KIY----RQAITSAGTGCMAAIEVEKYLSSLEE-------------
BLASB00041	NKPGVFAAGDVQD-PNY----RQAITSAGTGCMAALDLERYLYHEKM-------------
BLASP00551	NKPGVFAAGDVQD-STY----RQAVTSAGTGCMAALDLEKYLCLC---------------
AZOPC00437	NLPGVFAAGDVSD-PNY----QQAITAAGAGCMAAIEVERYLNEMKH-------------

```
LEPBD01878      STSGIFAAGDVQD-GRY----QQAIIAAGSGAIAGLDVEEYLRENNL-------------
SALRD02577      DVPGVFACGDAQD-STY----RQAVTAAGTGCKAAIDAERWLSEHGAAEAPRADANRQPV
SALRM02866      DVPGVFACGDAQD-STY----RQAVTAAGTGCKAAIDAERWLSEHGAAEAPRADANRQPV
RIEPU00166      SVKGVFAAGDVSD-SFY----RQAISSAGTGCMAAIDAERYLYKVGDI------------
ORITB00015      SIEGIFAAGDVQD-NKY----RQAVTAASSGCMAALDVQDFLKK----------------
ORITI01234      SIEGIFAAGDVQD-NKY----RQAVTAASSGCMAALDVQDFLKKIE--------------
PREMB01541      NIEGVYAAGDVAD-PTY----RQGVVAAGTGAMAAIEVDRFLQKQ---------------
PREDF01155      SVEGVYAAGDVAD-PTY----RQGVVAAGTGAMAAIEVDRFLQKQ---------------
PREI702011      SVEGVYAAGDVAD-PTY----RQGVVAAGSGAKAAIEAERFLQDKGEK------------
ALIFI01035      NIEGVFAAGDVRD-PHY----RQAITAAASGCIAAIDCERFILNRAE-------------
ODOSD00362      NVPGVFAAGDIMD-PVY----RQGIAAAGSGCRAAIDAERYIGELESK------------
PRER201250      KVPGVFVAGDCAD-PVY----RQAISAAGSGCQAAIEAERFLLNK---------------
PALPW00206      NVSGVFAAGDVSD-PHY----RQAITAAGTGCQAAIEAERYLSEKGL-------------
PORGI00953      KVPGVFAAGDVAD-PHY----RQAITAAGSGCKAAIEAERYLGEHGL-------------
PORG301168      KVPGVFAAGDVAD-PHY----RQAITAAGSGCKAAIEAERYLGEHGL-------------
BACV803426      KVPGVFAAGDVAD-PHY----RQAITAAGSGCKAAIEAERYLSANDLL------------
BACT601486      KLPGVFAAGDVAD-PHY----RQAITAAGSGCKAAIEAERYLSAKGEI------------
BACTN04290      KVPGVFAAGDVAD-PHY----RQAITAAGSGCKAAIEAERYLSAKGII------------
BACFR01032      KVPGVFAAGDVAD-PHY----RQAITAAGSGCKAAIEAERYLSEKGLI------------
BACFN00913      KVPGVFAAGDVAD-PHY----RQAITAAGSGCKAAIEAERYLSEKGLI------------
BACF600963      KVPGVFAAGDVAD-PHY----RQAITAAGSGCKAAIEAERYLSEKGLI------------
OWEHD03413      NLPGVFATGDAQD-RIY----RQAVTAAGSGCMGALEAERYLQEFEEEVEAAQIVG----
PSYTT00390      NYPGVFACGDSQD-KEY----RQAVTAAGTGCMAALDAERYLGSLEGILENEKEAVAN--
NONDD00011      NLPGVFASGDVQD-KIY----RQAVTAAGTGCMAALDAERYLADIGVVEEVKAGDYSIS-
ROBBH02711      NKPGVFASGDVQD-KEY----RQAVTAAGTGCMAALDAERYLSEVEAGEGEEVEQQ----
CELAD02592      NLPGVFACGDSQD-KEY----RQAITAAGTGCMSALDAERYLAAIETPASMA--------
CELLC02474      NIPGVFASGDVQD-KEY----RQAVTAAGTGCMAALDAERYLASLEEIAQ----------
MARSH03072      NLPGVFACGDAQD-KEY----RQAVTAAGTGCMAALDAERYLAAIEAPEEVA--------
MURRD00159      NKPGVFASGDAQD-KIY----RQAVTAAGTGCMAALDAERYLASVESKEVLAS-------
ZOBGA00152      NKPGVFASGDAQD-KEY----RQAVTAAGTGCMAALDAERYLATVETPQETV--------
GRAFK00949      NIPGVFASGDVQD-KIY----RQAVTAAGTGCMAALDAERYLAEIETEEDIQKPQTRPET
ZUNPS02344      NIPGVFASGDVQD-KEY----RQAITAAGTGCMAAMDAERYLAEIETSEEEEKEVTA---
AEQSU02079      TKPGVFASGDVQD-KEY----RQAVTAAGTGCMAALDAERYLATIETEVTV---------
HALH105745      NIEGVFASGDAQD-NIY----RQAVTAAGTGCMAALDAERYLAAKGVI------------
SAPGL02893      KIPGVFVSGDAAD-KVY----RQAITAAGTGCMAALDAERYLAEEGII------------
AMOA500014      NIPGVFAAGDVQD-SIY----RQAITAAGTGCMAALDAERFLATEGQ-------------
FLAIG01802      NVAGVFVAGDAAD-HVY----RQAITAAGTGCMAALDAERYLASKE--------------
FLACA02326      NVAGVFVAGDAAD-HVY----RQAITAAGTGCMAALDAERYLAAKEY-------------
FLAJ100198      NVEGVFVAGDAAD-HVY----RQAITAAGTGCMAALDAERYLASQE--------------
FLABF01528      NVAGVFVAGDAAD-HVY----RQAITAAGTGCMAALDAERYLASL---------------
CAPOD01333      NVAGVFVAGDAAD-TRY----RQAITAAGSGCMAALDAEKYLLTLE--------------
CAPCC00414      NVKGVFVAGDAAD-SRY----RQAITAAGSGCMAALDAERYLATLEDE------------
FLELS01898      NIPGVFASGDAQD-KIY----RQAVTAAGTGCMAALDAERYLVEREALIDA---------
SOLCM01166      NVEGVFAAGDVQD-HIY----RQAVTAAGSGCMAALDAERYIAAKEHSSVYAEDLSN---
PEDHD01598      NVEGVFASGDVQD-SYY----RQAITAAGSGCMAALDAERYLAAKEHEIKVIS-------
SPHS203207      NVEGVFAAGDVQD-HIY----RQAVTAAGSGCMAALEAERYLAAKEHAIIETAD------
LEAB401188      NVPGVFACGDAQD-NVY----RQAITAAGTGCMAALDAERYLAAH---------------
EMTOG00092      NIEGVFACGDAQD-KIY----RQAVTAAGSGCMAALDAERYLAAQGLH------------
SPILD01737      NIPGVFACGDAQD-NIY----RQAVTAAGTGCMAALDAERYLVTLEMQGEEIVH------
DYAFD00084      NIKGVFACGDAQD-NVY----RQAITAAGTGCMAALDAERFLVEREVDVIIEEETA----
CYCMS03063      NVPGVFACGDAQD-HEY----RQAVTAAGTGCMAALDAERFLTSQE--------------
ECHVK03326      NIEGVFACGDAQD-HVY----RQAVTAAGTGCMSALDAERFLAEKELA------------
BELBD02542      NIEGVFACGDAQD-HIY----RQAITAAGTGCMAALDAERYLASLE--------------
NITGG02112      SVDGVFAAGDVHD-HRY----RQAVTAAGFGCMAAIDVERWLSERKHIKK----------
THEM700111      RIPGVFVAGDVRD-HRY----RQAVTAAAEGCKAAMEAAWFLEGTSDVVPAAPGQEAGVR
CREAS01350      NIEGIFVAGEAAD-YKY----RQAITAAGDGCKAALDAIKYIEDLIAKGIIKD-------
TURPD02772      NIPGVFAAGDVKD-HYY----RQAITAAGSGCQAAIEAERYLESVHAAKQTA--------
SORC507603      NIPGVFAAGDVQD-WNY----RQAVTAAGTGCMAALDAERWLAQQGGH------------
BDEBA00337      NIPGVFAAGDVQD-HVY----RQAITAAGTGCMAAIDAERWLEAQEAH------------
STIAD02752      NIEGVFACGDVQD-STY----RQAITAAGTGCMAAIEAERWLIEQGK-------------
MYXXD01876      NIEGVFACGDVQD-HYY----RQAITAAGTGCMAAIDAERWLIEHGE-------------
MYXFH03454      NIEGVFACGDVQD-HYY----RQAITAAGTGCMAAIDAERWLIEHGE-------------
CORCM01960      NIEGVFACGDVQD-SYY----RQAITAAGTGCMAAIDAERWLIEHGE-------------
MYXSD02209      NIPGVFACGDVQD-SYY----RQAITAAGTGCMAAIDAERWLIEEGE-------------
LEPFC02126      SVPGIFAAGDVQD-PTY----RQAISAAGSGCMAAMDAERFLEAGGHG------------
```

LEPFM02294	SAPGIFAAGDVQD-PVY----RQAITAAGSGCMAAIDAERYLESLAEPQ-----------
SOLUE00582	NIAGVFHAGDVQD-RTY----RQAITAAGAGCMAAIEVERFLEAEGH-------------
KORVE01333	KVPGVFASGDVQD-RRY----RQAITAAGSGCMAAIEAERFLEEHGG-------------
ACIC502553	RVPGVYACGDVQD-RRY----RQAITAAGSGCMAALEVEKFLEEHGR-------------
ACIFD00030	SVDGVFAAGDVQD-HTY----RQAITAAGSGCQAALDVERFLAARE--------------
HALMS01402	NVEGVFACGDVQD-SYY----RQAISAAGSGCQAAIRAERFLEEN---------------
CHLPD01133	SVPGVFACGDVQD-YTY----RQAITAAGTGCMAAVDAERFLESIR--------------
CHLPB01137	SVKGVFACGDVQD-YTY----RQAITAAGSGCMAAVDAERYLDSIR--------------
PROA200897	SVQGVFACGDVQD-YTY----RQAITAAGSGCKSALDAERYLESIR--------------
WIGBR00492	SIPGIFSAGDVSD-YLY----RQAITASGFGCMAALDAEKYLSSL---------------
BUCA500289	SIPGIFAAGDVID-HVY----RQAITSSASGCMAALDSERYINSLV--------------
BUCAI00292	SIPGIFAAGDVID-HVY----RQAITSSASGCMAALDSERYINSLV--------------
BUCAF00306	SIPGIFAAGDVID-HVY----RQAITSSASGCMAALDSERYINSLV--------------
BUCAT00287	SIPGIFAAGDVID-HVY----RQAITSSASGCMAALDSERYINSLV--------------
BUCA000302	SIPGIFAAGDVID-HVY----RQAITSSASGCMAALDSERYINSLV--------------
BAUCH00290	SVPGVFAAGDVID-KNY----RQAITAASSGCMAALDAQRYINTQNK-------------
BLOVB00369	SVPGIFAAGDVMD-YMY----RQAITASGTGCMAAIDAERFLSTIQNN------------
BLOFL00367	SVNGIFAAGDVID-YHY----RQAITAAGSGCMAAIDAERFLSNIQK-------------
BLOPB00375	SIPGIFAAGDVMD-HSY----RQAITAAGSGCMAAIDAERYLSTIMY-------------
BUCAP00289	NIPGVFAAGDVID-HVY----RQAITSSASGCMAALDSERYLNSLS--------------
COXBU01001	NIPGVFAAGDVTD-HVY----RQAITAAGMGCMAALDAERYLDSLNQA------------
COXBN01188	NIPGVFAAGDVTD-HVY----RQAITAAGMGCMAALDAERYLDSLNQA------------
COXBR01117	NIPGVFAAGDVTD-HVY----RQAITAAGMGCMAALDAERYLDSLNQA------------
COXB200717	NIPGVFAAGDVTD-HVY----RQAITAAGMGCMAALDAERYLDSLNQA------------
COXB100914	NIPGVFAAGDVTD-HVY----RQAITAAGMGCMAALDAERYLDSLNQA------------
FRAP200241	NIKGVFAAGDVAD-HVY----KQAVTSAGTGCMAALDAEKYLDNLNQ-------------
FRANT01012	NIKGVFAAGDVAD-HVY----KQAVTSAGTGCMAALDAEKYLDNLNQ-------------
FRACN00522	NIEGVFAAGDVAD-HIY----KQAVTSAGTGCMAALDAEKYLDNLGQ-------------
FRATT00465	NIKGVFAAGDVAD-HVY----KQAVTSAGTGCMAALDAEKYLDNLDQ-------------
FRAT100465	NIKGVFAAGDVAD-HVY----KQAVTSAGTGCMAALDAEKYLDNLDQ-------------
FRATE00462	NIKGVFAAGDVAD-HVY----KQAVTSAGTGCMAALDAEKYLDNLDQ-------------
FRATW01216	NIKGVFAAGDVAD-HVY----KQAVTSAGTGCMAALDAEKYLDNLDQ-------------
FRATM01110	NIKGVFAAGDVAD-HVY----KQAVTSAGTGCMAALDAEKYLDNLDQ-------------
FRACF00566	NIKGVFAAGDVAD-HVY----KQAVTSAGTGCMAALDAEKYLDNLDR-------------
FRATO01212	NIKGVFAAGDVAD-HVY----KQAVTSAGTGCMAALDAEKYLDNLDQ-------------
FRATH01486	NIKGVFAAGDVAD-HVY----KQAVTSAGTGCMAALDAEKYLDNLDQ-------------
FRATF01216	NIKGVFAAGDVAD-HVY----KQAVTSAGTGCMAALDAEKYLDNLDQ-------------
FRATN00564	NIKGVFAAGDVAD-HVY----KQAVTSAGTGCMAALDAEKYLDNLDQ-------------
ACIF500512	SIEGVFAAGDVQD-YVY----RQAVTSAGTGCMAALDAERWLEQQQEG------------
ACIF200362	SIEGVFAAGDVQD-YVY----RQAVTSAGTGCMAALDAERWLEQQQEG------------
DECAR01284	SIPGIFAAGDVQD-QIY----KQACTSAGSGCMAALDAERYLDNLGHA------------
NEIG100525	NIEGVWAAGDVKD-HTY----RQAITSAASGCQAALDAERWLGSQNI-------------
NEIG201314	NIEGVWAAGDVKD-HTY----RQAITSAASGCQAALDAERWLGSQNI-------------
NEIM800926	NIEGVWAAGDVKD-HTY----RQAITSAASGCQAALDAERWLGSQNI-------------
NEIMP01199	NIEGVWAAGDVKD-HTY----RQAITSAASGCQAALDAERWLGSQNI-------------
NEIMB01212	NIEGVWAAGDVKD-HTY----RQAITSAASGCQAALDAERWLGSQNI-------------
NEIMF01143	NIEGVWAAGDVKD-HTY----RQAITSAASGCQAALDAERWLGSQNI-------------
NEIML01119	NIEGVWAAGDVKD-HTY----RQAITSAASGCQAALDAERWLGSQNI-------------
NEIMM00780	NIEGVWAAGDVKD-HTY----RQAITSAASGCQAALDAERWLGSQNI-------------
NEIMH00827	NIEGVWAAGDVKD-HTY----RQAITSAASGCQAALDAERWLGSQNI-------------
NEIMG01168	NIEGVWAAGDVKD-HTY----RQAITSAASGCQAALDAERWLGSQNI-------------
NEIMN01254	NIEGVWAAGDVKD-HTY----RQAITSAASGCQAALDAERWLGSQNI-------------
NEIMO00810	NIEGVWAAGDVKD-HTY----RQAITSAASGCQAALDAERWLGSQNI-------------
NEIM701229	NIEGVWAAGDVKD-HTY----RQAITSAASGCQAALDAERWLGSQNI-------------
NEIMA01334	NIEGVWAAGDVKD-HTY----RQAITSAASGCQAALDAERWLGSQNI-------------
NEIMW01125	NIEGVWAAGDVKD-HTY----RQAITSAASGCQAALDAERWLGSQNI-------------
DICNV01037	SIDGVFAAGDVAD-PVY----RQAITSAASGCMAALDAERYLATLKN-------------
VESOH00047	SIEGVFAAGDVSD-YVY----RQAITSAGSGCMSALDAERFLGE----------------
RUTMC00043	SVEGIFAAGDVTD-HIY----RQAITSAGSGCMSALDAERFLGE----------------
HALHL02255	SAPGVFAAGDVCD-HVY----RQAVTSAGTGCMAALDVERYLERLAHS------------
PELPD03177	SVEGVFAAGDVQD-YIY----RQAVTSAASGCMAAKDAEHYLAALRH-------------
GEOS804030	NIPGVFAAGDVQD-RNY----KQAITSAGTGCMAALDADRYLEMLKA-------------
GEOBB03636	NIPGVFAAGDVQD-RNY----KQAITSAGTGCMAALDADRYLEMLKA-------------
GEOSM03700	NIPGVFAAGDVQD-RNY----KQAITSAGTGCMAALDADRYLEMLKA-------------
HALNC00046	SVPGVFAAGDVMD-HVY----RQAITSAGTGCMAALDAKTYLEGLE--------------

```
HAEPS00887      SVAGVFAAGDVMD-HNY----RQAITSAGTGCMAALDAEHFLEYCS--------------
GALAU02285      SVEGVFAAGDVMD-QHY----RQAITSAGTGCMAALDAERYLDAQQAKGA----------
HISS201000      SVQGVFAAGDVMD-HNY----RQAITSAGTGCMAALDAERFLDTQK--------------
HAES101151      SVQGVFAAGDVMD-HNY----RQAITSAGTGCMAALDAERFLDTQK--------------
PASMU00573      SVDGVFAAGDVMD-HNY----RQAITSAGTGCMAALDAERYLDAQK--------------
PASMH00579      SVDGVFAAGDVMD-HNY----RQAITSAGTGCMAALDAERYLDAQK--------------
ACTSZ01560      SVEGVFAAGDVMD-HNY----RQAITSAGTGCMAALDAERFLDAQE--------------
AGGAN00985      SVEGIFAAGDVMD-HNY----RQAITSAGTGCMAALDAERFLDTQ---------------
NITHN03181      SIRGVFAAGDVID-SVY----RQAVTSAGTGCMAALDAEKYLDGLEEK------------
NITOC00311      SVRGVFAAGDVID-PTY----RQAVTSAGTGCMAALDADKYLDGLEER------------
NITWC02498      SVQGVFAAGDVID-PTY----RQAVTSAGTGCMAALDAEKYLDGLEEQ------------
METNJ00648      SIPGVFAAGDVSD-PIY----RQAITSAGAGCMAALDAERFLESEK--------------
METFJ01616      SIPGVFAAGDVSD-PIY----RQAITSAGAGCMAALDAERFLESQK--------------
ALKEH00244      SIPGVFAAGDVMD-HVY----RQAVTSAGTGCMAALDAEKYLDALHLE------------
MARMS03166      SVEGVFAAGDVSD-HIY----RQAITSAGTGCMAALDAERYLDGIADA------------
MARM102688      SIPGVYAAGDVMD-HIY----RQAITSAGTGCMAALDAEKFLDDME--------------
THICR00763      SIPGIFAAGDVMD-QVY----KQAITSAGAGCMAALDAEKFLDQLEAQG-----------
THICA00974      SIPGVFAAGDVMD-QVY----KQAITSSGAGCMAALDVEKFLESQEG-------------
THIV600851      SVPGVFAAGDVMD-PIY----RQAITSAGTGCMAALDAEKFLDAMAVGGV----------
THISH01973      SVPGIFAAGDVMD-HVY----RQAVTSAGTGCMAALDAERFLEENR--------------
METAA00813      SVPGVFAAGDVMD-AVY----KQAITSAGAGCMAALDAEKYLDEME--------------
META200813      SVPGVFAAGDVMD-AVY----KQAITSAGAGCMAALDAEKYLDEME--------------
METMM02895      SVPGVFAAGDVMD-SVY----KQAITSAGAGCMAALDAEKFLDELTG-------------
FRAAD00813      SVPGVFAAGDVAD-HVY----RQAITSAGFGCMAALDAERWLDQHAPAA-----------
XYLFA01416      SVEGVFAAGDVAD-QHY----RQAITSAGFGCMAALDAERFLDKGN--------------
XYLFT00623      SVEGVFAAGDVAD-QHY----RQAITSAGFGCMAALDAERFLDKGN--------------
XYLF200667      SVEGVFAAGDVAD-QHY----RQAITSAGFGCMAALDAERFLDKGN--------------
XYLFG01622      SVEGVFAAGDVAD-QHY----RQAITSAGFGCMAALDAERFLDKGN--------------
XYLFM00690      SVEGVFAAGDVAD-QHY----RQAITSAGFGCMAALDAERFLDKGN--------------
PSEUP01680      TVPGVFAAGDVAD-QHY----RQAITSAGFGCMAALDAERFLDKGT--------------
STRM501972      SVEGVFAAGDVAD-QHY----RQAITSAGFGCMAALDAERYLDALKISDQQQHKNAA---
PSEUU01524      SVEGVFAAGDVAD-QHY----RQAITSAGFGCMAALDAERYLDKGA--------------
XANAP01407      SVPGVFAAGDVAD-QHY----RQAITSAGFGCMAALDAERYLDKSA--------------
XANCP01918      SVAGVFAAGDVAD-QHY----RQAITSAGFGCMAALDAERYLDKSA--------------
XANC802154      SVAGVFAAGDVAD-QHY----RQAITSAGFGCMAALDAERYLDKSA--------------
XANCB02263      SVAGVFAAGDVAD-QHY----RQAITSAGFGCMAALDAERYLDKSA--------------
XANOR02379      SVAGVFAAGDVAD-QHY----RQAITSAGFGCMAALDAERFLDKSA--------------
XANOM02295      SVAGVFAAGDVAD-QHY----RQAITSAGFGCMAALDAERFLDKSA--------------
XANOP02318      SVAGVFAAGDVAD-QHY----RQAITSAGFGCMAALDAERFLDKSA--------------
XANAC01951      SVAGVFAAGDVAD-QHY----RQAITSAGFGCMAALDAERFLDKSA--------------
CYCSP01155      SIPGIFAAGDVAD-SIY----RQAITSAGSGCMAALDAERFLDALEDA------------
GEOLS03275      SIPGIFAAGDVQD-YTY----RQAITSAGTGCMAALDAERYLDGLGG-------------
GEOUR03858      SVPGVFAAGDVQD-QTY----KQAITSAGTGCMAALDAERYLDMLKA-------------
GEODF00692      SIPGVFAAGDVQD-QYY----RQAITSAGTGCMAALDAERYLDMLKP-------------
GEOSL00482      SIPGVFAAGDVQD-QHY----RQAITSAGTGCMAALDAERYLDMLKP-------------
GEOSK00469      SIPGVFAAGDVQD-QHY----RQAITSAGTGCMAALDAERYLDMLKP-------------
GEOMG02998      SIPGVFAAGDVQD-QYY----KQAITSAGTGCMAALDAEKYLETLLS-------------
MORCR00228      NINGVFAAGDVAD-HTY----RQAITSAGTGCMAALDAEKYLDSLTI-------------
ACIAD00798      SVAGVFAAGDVAD-NIY----RQAITSAGSGCMAALDADFYLDSI---------------
ACIBC00821      SVAGVFAAGDVAD-SIY----RQAITSAGSGCMAALDAEKYLDNL---------------
ACIBY02725      SVAGVFAAGDVAD-SIY----RQAITSAGSGCMAALDAEKYLDNL---------------
ACIB302691      SVAGVFAAGDVAD-SIY----RQAITSAGSGCMAALDAEKYLDNL---------------
ACIB500882      SVAGVFAAGDVAD-SIY----RQAITSAGSGCMAALDAEKYLDNL---------------
ACIB100850      SVAGVFAAGDVAD-SIY----RQAITSAGSGCMAALDAEKYLDNL---------------
ACIBD00843      SVAGVFAAGDVAD-SIY----RQAITSAGSGCMAALDAEKYLDNL---------------
ACIBS02229      SVAGVFAAGDVAD-SIY----RQAITSAGSGCMAALDAEKYLDNL---------------
ACICP00137      SVAGVFAAGDVAD-SIY----RQAITSAGSGCMAALDAEKYLDNL---------------
ACISD03052      SVAGVFAAGDVAD-SIY----RQAITSAGSGCMAALDAEKYLDNL---------------
LEGLN02402      SIPGVFACGDVAD-HVY----RQAITSAGFGCMAALDAEQYLDLNVHK------------
LEGPA01726      SVPGVFACGDVAD-HVY----RQAITSAGFGCMAALDAEKYLDSI---------------
LEGPH00846      SVPGVFACGDVAD-HVY----RQAITSAGFGCMAALDAEKYLDSI---------------
LEGPC01171      SVPGVFACGDVAD-HVY----RQAITSAGFGCMAALDAEKYLDSI---------------
LEGP201857      SVPGVFACGDVAD-HVY----RQAITSAGFGCMAALDAEKYLDSI---------------
LEGPL01727      SVPGVFACGDVAD-HVY----RQAITSAGFGCMAALDAEKYLDSI---------------
COLP302668      SVEGVFAAGDVAD-HIY----RQAITSAGAGCMAALDSERYLDAL---------------
```

```
KANKD01106      SVEGVFAAGDVCD-HVY----RQAVTSAGTGCMAALDAEKYLDQ----------------
IDILO00659      SVPGVFAAGDVMD-HIY----RQAITSAGTGCMAALDAERYLDALTKS------------
PSEU901284      SVPGVFAAGDVSD-HIY----RQAITSAGTGCMAALDAERFLDNL---------------
PSEA602346      SVEGVFAAGDVSD-HVY----RQAVTSAGTGCMAALDAEKFLDAL---------------
ALTSS01871      SVEGVFAAGDVSD-HIY----RQAITSAGTGCMAALDAEKYLDGFMPEQG----------
ALTMD01611      SVEGVFAAGDVSD-HIY----RQAITSAGTGCMAALDAEKYLDGIMPEQG----------
ALTME01705      SVEGVFAAGDVSD-HIY----RQAITSAGTGCMAALDAEKYLDGTMPEQG----------
ALTMB01797      SVEGVFAAGDVSD-HIY----RQAITSAGTGCMAALDAEKYLDGTMPEQG----------
ALTMS01652      SVEGVFAAGDVSD-HIY----RQAITSAGTGCMAALDAEKYLDGTMPEQG----------
SACD201683      SVPGVFAAGDVAD-HIY----RQAITSAGFGCMAALDAEKFLDS----------------
TERTT01748      NVPGVFAAGDVAD-HIY----RQAVTSAGAGCMAALDAEKYLDNLV--------------
SIMAS00609      SVPGVFAAGDVAD-HIY----RQAITSAGAGCMAALDAEKYLDDL---------------
ALCDB01962      SVAGVFAAGDVAD-HVY----RQAVTSAGSGCMAALDAERYLETLED-------------
CHRSD02939      SVPGVFAAGDVMD-HVY----RQAVTSAGTGCMAALDAERYLDGLE--------------
HALED02811      SVPGVFAAGDVMD-HVY----RQAVTSAGSGCMAALDAERYLDEL---------------
SIDLE02655      SIDGVFAAGDVQD-YIY----RQACTSAATGCMAALDADKYLSELR--------------
GALCS01621      SISGVFAAGDVQD-QIY----RQAVTSAATGCMAALDADKYLASIGKL------------
LARHH01446      SIEGVFAAGDVQD-HIY----RQAVTSAASGCQAALDAERFLDH----------------
CHRVO02807      SIDGVFAAGDVQD-HIY----RQAVTSAASGCQAALDAERYLDSLK--------------
PSEUL01648      SVPGVFAAGDVQD-HIY----RQAVTSAASGCQAALDAERFLDGK---------------
NITEU01859      SIPGIFAAGDVQD-HIY----RQAITSAGSGCMAALDAERYLEKSA--------------
NITEC00745      SIPGVFAAGDVQD-HIY----RQAVTSAGSGCMAALDAERYLEKSS--------------
NITMU00023      SIPGVFAAGDVQD-HIY----RQAVTSAGTGCMAALDAEKYLDVLV--------------
NITSI03064      SIPGVFAAGDVQD-HIY----RQAVTSAASGCMAALDAEKYLDNLK--------------
ACCPU02128      SIAGVFAAGDVQD-HIY----RQACTSAGSGCMAALDAERYLDSLGLAG-----------
THIDA01000      SVEGVFAAGDVQD-HIY----RQAVTSAGTGCMAALDAERYLDALGKTK-----------
METS601221      SVPGVFAAGDVQD-HIY----RQAVTSAGSGCMAALDAERYLDQLK--------------
METGS01186      SVPGVFAAGDVQD-HIY----RQAVTSAGSGCMAALDAERYLDQLK--------------
METFK00972      SVPGVFAAGDVQD-HIY----RQAVTSAGTGCMAALDAERYLDQLS--------------
METML01428      SVPGVFAAGDVQD-HIY----RQAVTSAGTGCMAALDAERYLDHLK--------------
AROAE03899      SVAGVFAAGDVQD-HIY----RQAVTSAGTGCMAALDAERYLDTLAG-------------
THASP01686      SVPGVFAAGDVQD-HIY----RQAVTSAGTGCMAALDAERYLDALGV-------------
AZOSB01359      NIAGVFAAGDVQD-HIY----RQAVTSAGSGCMAALDAERYLDALS--------------
BORA102682      SVPGVFAAGDVQD-HIY----RQAITSAGTGCMAALDAQRWLENAGN-------------
BORPA03415      SVPGVFAAGDVQD-HVY----RQAITSAGTGCMAALDAQRWLENAGQ-------------
BORBM03577      SVPGVFAAGDVQD-HVY----RQAITSAGTGCMAALDAQRWLENAGQ-------------
BORPE02280      SVPGVFAAGDVQD-HVY----RQAITSAGTGCMAALDAQRWLENAGQ-------------
BORPC02060      SVPGVFAAGDVQD-HVY----RQAITSAGTGCMAALDAQRWLENAGQ-------------
BORP102392      SVPGVFAAGDVQD-HVY----RQAITSAGTGCMAALDAQRWLENAGQ-------------
BORBR03870      SVPGVFAAGDVQD-HVY----RQAITSAGTGCMAALDAQRWLENAGQ-------------
BORPD01524      SVPGVFAAGDVQD-HVY----RQAITSAGTGCMAALDAQRWLENAQ--------------
ACHXA01179      SVPGVFAAGDVQD-HVY----RQAITSAGTGCMAALDAQRWLENAGQ-------------
RHOFT03114      SVPGIFAAGDVQD-HVY----RQAITSAGTGCMAALDAQRFLEQE---------------
VEREI01701      SVPGVFAAGDVQD-HVY----RQAITSAGTGCMAALDAQRFLEQARN-------------
VARPE01375      SIPGVFAAGDVQD-NVY----RQAITSAGTGCMAALDAQRFLEQDGTL------------
VARPS01266      SIPGVFAAGDVQD-NVY----RQAITSAGTGCMAALDAQRFLEQDGTL------------
DELAS05272      SVPGVFAAGDVQD-HVY----RQAITSAGTGCMAALDAQRFLEQE---------------
DELSC01235      SVHGVFAAGDVQD-HVY----RQAITSAGTGCMAALDAQRFLEQE---------------
COMT200883      SVPGVFAAGDCQD-HVY----RQAITSAGTGCMAALDAQRFLEQES--------------
ACIAC03267      SVPGVFAAGDVQD-HVY----RQAITSAGTGCMAALDAQRFLEQEGVI------------
ACIAP03249      SVPGVFAAGDVQD-HVY----RQAITSAGTGCMAALDAQRFLEQESVI------------
ACIET02648      SVPGVFAAGDVQD-HVY----RQAITSAGTGCMAALDAQRFLEQQE--------------
ALIDK03528      SVPGVFAAGDVQD-HVY----RQAITSAGTGCMAALDAQRFLEQQ---------------
RUBGI03408      SVPGVFAAGDVQD-HVY----RQAITSAGTGCMAALDAQRYLEQSGL-------------
LEPCP00749      SVPGVFAAGDVQD-HVY----RQAITSAGTGCMAALDAQRFLEQGGAAG-----------
POLSJ03719      SVPGVFAAGDVQD-HVY----RQAITSAGTGCMAALDAQRFLEQS---------------
POLNA03144      SIPGVFAAGDVQD-NIY----RQAITSAGTGCMAALDAQRFLEQSD--------------
METPP01110      SVPGVFAAGDVQD-HVY----RQAITSAGTGCMAALDAQRFLEQG---------------
RAMTT03218      SVPGVFAAGDVQD-HVY----RQAITSAGTGCMAALDAQRFLEQQE--------------
RALPJ02457      NIPGVFAAGDVQD-HIY----RQAITSAGTGCMAALDAQRYLESLE--------------
RALP102102      NIPGVFAAGDVQD-HIY----RQAITSAGTGCMAALDAQRYLESLE--------------
RALSO02303      NIPGVFAAGDVQD-HIY----RQAITSAGTGCMAALDAQRYLENQE--------------
RALS801097      NIPGVFAAGDVQD-HIY----RQAITSAGTGCMAALDAQRYLENLE--------------
HERSS01878      SIDGVFAAGDVQD-HVY----RQAITSAGTGCMAALDAQRYLEGLE--------------
HERAR00955      SVPGVFAAGDVQD-HIY----RQAVTSAGTGCMAALDAQRYLESQE--------------
```

JANMA01152	SVPGVFAAGDVQD-HVY----RQAITSAGTGCMAALDAQRYLESQE--------------
THIK102324	SVPGVFAAGDVQD-HVY----RQAITSAGTGCMAALDAQRYLENAES-------------
POLSQ00682	NIPGVFAAGDVQD-HIY----RQAITSAGTGCMAALDAQRYLETLS--------------
POLNS00989	NIPGVFAAGDVQD-HIY----RQAITSAGTGCMAALDAQRYLETLE--------------
BURP800627	SVPGVFAAGDVQD-HVY----RQAITSAGTGCMAALDAQRYLESIHESIGERAMSAEADR
BURPP00988	SVAGVFAAGDVQD-HVY----RQAITSAGTGCMAALDAQRYLETIDEMAGEHAMSQEAER
BURSC00728	SVPGVFAAGDVQD-HIY----RQAITSAGTGCMAALDAQRYLETINEMAGEHAMSAEADR
BURXL00982	SVPGVFAAGDVQD-HVY----RQAITSAGTGCMAALDAQRYLETIDEMAGEHAMSQEAGR
BURSG00869	SVPGVFAAGDVQD-HVY----RQAITSAGTGCMAALDAQRYLETINEMAGEHAMSQEAER
BURRH00629	SIAGVFAAGDVQD-HIY----RQAITSAGTGCMAALDAQRYLESIHETTAPHVMSHEANR
BURGB00781	SVPGVFAAGDVQD-HIY----RQAITSAGTGCMAALDAQRYLESLHDAN-----------
BURGS00853	NIPGVFAAGDVQD-HIY----RQAITSAGTGCMAALDAQRYLESLHDAK-----------
BURPS02618	SVPGVFAAGDVQD-HIY----RQAITSAGTGCMAALDAQRYLESLHDHK-----------
BURMA01741	SVPGVFAAGDVQD-HIY----RQAITSAGTGCMAALDAQRYLESLHDHK-----------
BURP103016	SVPGVFAAGDVQD-HIY----RQAITSAGTGCMAALDAQRYLESLHDHK-----------
BURP002984	SVPGVFAAGDVQD-HIY----RQAITSAGTGCMAALDAQRYLESLHDHK-----------
BURM701866	SVPGVFAAGDVQD-HIY----RQAITSAGTGCMAALDAQRYLESLHDHK-----------
BURP602945	SVPGVFAAGDVQD-HIY----RQAITSAGTGCMAALDAQRYLESLHDHK-----------
BURM902465	SVPGVFAAGDVQD-HIY----RQAITSAGTGCMAALDAQRYLESLHDHK-----------
BURMS00740	SVPGVFAAGDVQD-HIY----RQAITSAGTGCMAALDAQRYLESLHDHK-----------
BURTA01515	SVPGVFAAGDVQD-HIY----RQAITSAGTGCMAALDAQRYLESLHDHK-----------
BURM102386	SVPGVFAAGDVQD-NVY----RQAITSAGTGCMAALDAQRYLESLHDKK-----------
BURL300890	SVAGVFAAGDVQD-NVY----RQAITSAGTGCMAALDAQRYLESLHDKK-----------
BURVG00860	SVPGVFAAGDVQD-NVY----RQAITSAGTGCMAALDAQRYLETLHDKK-----------
BURCM00829	SVPGVFAAGDVQD-NVY----RQAITSAGTGCMAALDAQRYLEGLHDKK-----------
BURA400832	SVPGVFAAGDVQD-NVY----RQAITSAGTGCMAALDAQRYLEGLHDKK-----------
BURCA00489	SVPGVFAAGDVQD-NVY----RQAITSAGTGCMAALDAQRYLESLHDKK-----------
BURCH00966	SVPGVFAAGDVQD-NVY----RQAITSAGTGCMAALDAQRYLESLHDKK-----------
BURCC00927	SVPGVFAAGDVQD-NVY----RQAITSAGTGCMAALDAQRYLESLHDKK-----------
BURCJ02922	SVPGVFAAGDVQD-NVY----RQAITSAGTGCMAALDAQRYLESLHDKK-----------
EDWI902348	SVPGVFAAGDVMD-HIY----RQAITSAGTGCMAALDAERYLDSLADTARHA--------
EDWTF01971	SVPGVFAAGDVMD-HIY----RQAITSAGTGCMAALDAERYLDSLADAARHA--------
EDWTE02176	SVPGVFAAGDVMD-HIY----RQAITSAGTGCMAALDAERYLDSLADAARHA--------
SODGM01094	SIPGVFAAGDVMD-HNY----RQAITSAGTGCMAALDAERYLDGLAQAGSL---------
MOREP00394	SIPGVFAAGDVMD-HHY----RQAITSSGTGCMAALDAERYLDGLA--------------
RAHSY01432	SIPGVFAAGDVMD-HIY----RQAITSAGTGCMAALDAERYLDGLSQAPDL---------
RAHAC01419	SIPGVFAAGDVMD-HIY----RQAITSAGTGCMAALDAERYLDGLSQAPDL---------
ERWBE01489	SIPGVFAAGDVMD-HIY----RQAITSAGTGCMAALDAERFLDGLVKNSQ----------
PANAM01339	SIPGVFAAGDVMD-HIY----RQAITSAGTGCMAALDAERYLDGLVKNDK----------
PANAA00662	SIPGVFAAGDVMD-HIY----RQAITSAGTGCMAALDAERYLDGLVKNDK----------
PANSA01303	SIPGVFAAGDVMD-HIY----RQAITSAGTGCMAALDAERYLDGLVKNVK----------
ERWT902154	SIPGVFAAGDVMD-HIY----RQAITSAGTGCMAALDAERYLDGLVKNNQ----------
ERWAC01330	SVPGVFAAGDVMD-HIY----RQAITSAGTGCMAALDAERYLDGLVKKQSVTC-------
ERWAE01326	SVPGVFAAGDVMD-HIY----RQAITSAGTGCMAALDAERYLDGLVKKQSVTC-------
ERWPE02241	SIAGVFAAGDVMD-HIY----RQAITSAGTGCMAALDAERYLDGLVKNNQ----------
ERWP602414	SIAGVFAAGDVMD-HIY----RQAITSAGTGCMAALDAERYLDGLVKNNQ----------
ERWSE02396	SIAGVFAAGDVMD-HIY----RQAITSAGTGCMAALDAERYLDGLVKNNQ----------
PECCP01694	SIPGVFAAGDVMD-HIY----RQAITSAGTGCMAALDAERYLDGLTVNK-----------
PECWW01889	SIPGVFAAGDVMD-HIY----RQAITSAGTGCMAALDAERYLDGLTINK-----------
PECSS01870	SIPGVFAAGDVMD-HIY----RQAITSAGTGCMAALDAERYLDGLTINK-----------
PECAS02624	SIPGVFAAGDVMD-HIY----RQAITSAGTGCMAALDAERYLDGLTVK------------
DICDC02220	SIPGVFAAGDVMD-HNY----RQAITSAGTGCMAALDAERYLDSLNDK------------
DICZE02284	SIPGVFAAGDVMD-HIY----RQAITSAGTGCMAALDAERYLDALAG-------------
DICD302017	SIPGVFAAGDVMD-HIY----RQAITSAGTGCMAALDAERYLDALVK-------------
DICD502250	SIPGVFAAGDVMD-HIY----RQAITSAGTGCMAALDAERYLDALAK-------------
XENBS00829	SIPGVFAAGDVMD-HVY----RQAITSAGTGCMAALDAERFLDGLATK------------
XENNA01480	SIPGIFAAGDVMD-HVY----RQAITSAGTGCMAALDAERFLDGLATK------------
PHOLL01537	SIPGVFAAGDVMD-HTY----RQAITSAGTGCMAALDAERFLDGLAAK------------
PHOAA02804	SIPGVFAAGDVMD-HTY----RQAITSAGTGCMAALDAERFLDKLATEPK----------
SERP501669	TIPGVFAAGDVMD-HIY----RQAITSAGTGCMAALDAERYLDGIAGAEVL---------
SERSA01625	TIPGVFAAGDVMD-HIY----RQAITSAGTGCMAALDAERYLDGIAGAEVL---------
YERPE01271	SIPGVFAAGDVMD-HIY----RQAITSAGTGCMAALDAERYLDGLANDK-----------
YERPS01390	SIPGVFAAGDVMD-HIY----RQAITSAGTGCMAALDAERYLDGLANDK-----------
YERPA00627	SIPGVFAAGDVMD-HIY----RQAITSAGTGCMAALDAERYLDGLANDK-----------
YERPN02489	SIPGVFAAGDVMD-HIY----RQAITSAGTGCMAALDAERYLDGLANDK-----------

```
YERPP02190      SIPGVFAAGDVMD-HIY----RQAITSAGTGCMAALDAERYLDGLANDK-----------
YERP302544      SIPGVFAAGDVMD-HIY----RQAITSAGTGCMAALDAERYLDGLANDK-----------
YERPB01460      SIPGVFAAGDVMD-HIY----RQAITSAGTGCMAALDAERYLDGLANDK-----------
YERPY02633      SIPGVFAAGDVMD-HIY----RQAITSAGTGCMAALDAERYLDGLANDK-----------
YERPG01392      SIPGVFAAGDVMD-HIY----RQAITSAGTGCMAALDAERYLDGLANDK-----------
YERPD01171      SIPGVFAAGDVMD-HIY----RQAITSAGTGCMAALDAERYLDGLANDK-----------
YERP100890      SIPGVFAAGDVMD-HIY----RQAITSAGTGCMAALDAERYLDGLANDK-----------
YERPZ01206      SIPGVFAAGDVMD-HIY----RQAITSAGTGCMAALDAERYLDGLANDK-----------
YERPH02469      SIPGVFAAGDVMD-HIY----RQAITSAGTGCMAALDAERYLDGLANDK-----------
YERE801438      SIPGVFAAGDVMD-HIY----RQAITSAGTGCMAALDAERYLDGLVNDK-----------
YERE302565      SIPGVFAAGDVMD-HIY----RQAITSAGTGCMAALDAERYLDGLVNDK-----------
YERE100392      SIPGVFAAGDVMD-HIY----RQAITSAGTGCMAALDAERYLDGLVNDK-----------
PROMH00687      SIEGVFAAGDVMD-HIY----RQAITSAGTGCMAALDAERYLDALKSN------------
PROSM03196      SIPGVFAAGDVMD-HIY----RQAITSAGTGCMAALDAERYLDGLADKK-----------
TOLAT02279      SIEGVFAAGDVAD-HIY----RQAITSAGTGCMAALDAERYLDSLAK-------------
AERVB02262      SIEGVFAAGDVAD-HNY----RQAITSAGTGCMAALDAERYLDAQ---------------
AERHH01811      SIEGVFAAGDVAD-HTY----RQAITSAGTGCMAALDAERYLDAQ---------------
AERS402185      SIEGVFAAGDVAD-HNY----RQAITSAGTGCMAALDAERYLDAQ---------------
PSYIN02070      SIKGVFAAGDVCD-HIY----RQAITSSGTGCMAALDAERYLDNL---------------
SHELP02018      SIPGVFAAGDVMD-QHY----RQAITSAGTGCMAALDAERFLDAQ---------------
SHEVD02297      SIEGVFAAGDVMD-QHY----RQAITSAGTGCMAALDAERYLDALK--------------
SHEPW02341      SIEGVFAAGDVMD-QHY----RQAITSAGTGCMAALDAERYLDAK---------------
SHEPA02202      SIEGVFAAGDVMD-QHY----RQAITSAGTGCMAALDAERYLDAK---------------
SHEHH02007      SIEGVFAAGDVMD-QHY----RQAITSAGTGCMAALDAERFLDAKN--------------
SHESH02117      SIEGVFAAGDVMD-QHY----RQAITSAGTGCMAALDAERYLDSK---------------
SHEWM02454      SIEGVFAAGDVMD-QHY----RQAITSAGTGCMAALDAERYLDAKNS-------------
SHEAM01758      SIEGVFAAGDVMD-QHY----RQAITSAGTGCMAALDAERYLDAKH--------------
SHEON02159      SIEGVFAAGDVMD-QHY----RQAITSAGTGCMAALDAERYLDAKK--------------
SHESM01945      SIEGVFAAGDVMD-QHY----RQAITSAGTGCMAALDAERYLDAKK--------------
SHESR01991      SIEGVFAAGDVMD-QHY----RQAITSAGTGCMAALDAERYLDTKK--------------
SHESA02025      SIEGVFAAGDVMD-QHY----RQAITSAGTGCMAALDAERYLDAKK--------------
SHESW01953      SIEGVFACGDVMD-QHY----RQAITSAGTGCMAALDAERYLDAKK--------------
SHEPC01976      SIEGVFACGDVMD-QHY----RQAITSAGTGCMAALDAERYLDAKK--------------
SHEP201929      SIEGVFACGDVMD-QHY----RQAITSAGTGCMAALDAERYLDAKK--------------
SHEB502016      SIEGVYACGDVMD-QHY----RQAITSAGTGCMAALDAERYLDAKK--------------
SHEB802168      SIEGVYACGDVMD-QHY----RQAITSAGTGCMAALDAERYLDAKK--------------
SHEB202117      SIEGVYACGDVMD-QHY----RQAITSAGTGCMAALDAERYLDAKK--------------
SHEB902270      SIEGVYACGDVMD-QHY----RQAITSAGTGCMAALDAERYLDAKK--------------
SHEB602258      SIEGVYACGDVMD-QHY----RQAITSAGTGCMAALDAERYLDAKK--------------
SHEDO01680      SIEGVYACGDVMD-QHY----RQAITSAGTGCMAALDAERYLDAKK--------------
SHEFN01895      SIEGVYACGDVMD-QHY----RQAITSAGTGCMAALDAERYLDAKK--------------
PSEHT01677      SVPGVFAAGDVSD-HIY----RQAITSAGTGCMAALDAERFLDNL---------------
ALISL01611      NIEGIFAAGDVMD-HNY----RQAITSAGTGCMAALDAERFLDALDVKES----------
VIBF100893      SVEGIFAAGDVMD-HNY----RQAITSAGTGCMAALDAERFLDALEAKES----------
VIBFM00891      SVEGIFAAGDVMD-HNY----RQAITSAGTGCMAALDAERFLDALEAKES----------
OCESG01399      SIEGVFAAGDVMD-HIY----RQAITSAGTGCMAALDAERYLDAQAQL------------
VIBA701872      SIEGVFAAGDVMD-HNY----RQAITSAGTGCMAALDAERYLDSLNDK------------
VIBVY01451      SVEGIFAAGDVMD-HNY----RQAITSAGTGCMAALDAERYLDSLNDK------------
VIBVU02545      SVEGIFAAGDVMD-HNY----RQAITSAGTGCMAALDAERYLDSLNDK------------
VIBVM01748      SVEGIFAAGDVMD-HNY----RQAITSAGTGCMAALDAERYLDSLNDK------------
VIBCH01157      SIEGVFAAGDVMD-HNY----RQAITSAGTGCMAALDAERYLDSQGK-------------
VIBCM01114      SIEGVFAAGDVMD-HNY----RQAITSAGTGCMAALDAERYLDSQGK-------------
VIBCJ02064      SIEGVFAAGDVMD-HNY----RQAITSAGTGCMAALDAERYLDSQGK-------------
VIBC300725      SIEGVFAAGDVMD-HNY----RQAITSAGTGCMAALDAERYLDSQGK-------------
FERBD01969      SVPGVFAAGDVMD-HNY----RQAITSAGTGCMAALDAERFLDAQ---------------
VIBFN02085      SIEGIFAAGDVMD-HNY----RQAITSAGTGCMAALDAERFLDALNDK------------
VIBTL01089      SIPGVFAAGDVMD-HNY----RQAITSAGTGCMAALDAERFLDGLNDK------------
VIBPA01248      SIEGVFAAGDVMD-HNY----RQAITSAGTGCMAALDAERFLDALSDK------------
VIBAE02128      SIEGVFAAGDVMD-HNY----RQAITSAGTGCMAALDAERFLDSLGDK------------
VIBCB00599      SIEGVFAAGDVMD-HNY----RQAITSAGTGCMAALDAERFLDALTDK------------
PANVC00701      SIPGVFAAGDVMD-HIY----RQAITSAGTGCMAALDAERFLDGLVKNDR----------
SHIBC02443      SIPGVFAAGDVMD-HIY----RQAITSAGTGCMAALDAERYLDGLAEQQR----------
ENTBF02792      SIPGVFAAGDVMD-HIY----RQAITSAGTGCMAALDAERYLDGLADACK----------
KLEP700892      SIPGVFAAGDVMD-HIY----RQAITSAGTGCMAALDAERYLDGLADACK----------
KLEPH01782      SIPGVFAAGDVMD-HIY----RQAITSAGTGCMAALDAERYLDGLADACK----------
```

KLEP303551 SIPGVFAAGDVMD-HIY----RQAITSAGTGCMAALDAERYLDGLADACK----------
KLEVT03420 SIPGVFAAGDVMD-HIY----RQAITSAGTGCMAALDAERYLDGLADACK----------
ENTAK02985 SIPGVFAAGDVMD-HIY----RQAITSAGTGCMAALDAERYLDGLADACK----------
KLEOK03151 SIPGVFAAGDVMD-HIY----RQAITSAGTGCMAALDAERYLDGLADACK----------
SALAR01925 SIPGVFAAGDVMD-HIY----RQAITSAGTGCMAALDAERYLDSLADTSE----------
SALBC00812 SIPGVFAAGDVMD-HIY----RQAITSAGTGCMAALDAERYLDGLADASK----------
SALPC00926 SIPGVFAAGDVMD-HIY----RQAITSAGTGCMAALDAERYLDGLADASK----------
SALTI00868 SIPGVFAAGDVMD-HIY----RQAITSAGTGCMAALDAERYLDGLADASK----------
SALCH00905 SIPGVFAAGDVMD-HIY----RQAITSAGTGCMAALDAERYLDGLADASK----------
SALPA01706 SIPGVFAAGDVMD-HIY----RQAITSAGTGCMAALDAERYLDGLADASK----------
SALTY00927 SIPGVFAAGDVMD-HIY----RQAITSAGTGCMAALDAERYLDGLADASK----------
SALPK01786 SIPGVFAAGDVMD-HIY----RQAITSAGTGCMAALDAERYLDGLADASK----------
SALHS00978 SIPGVFAAGDVMD-HIY----RQAITSAGTGCMAALDAERYLDGLADASK----------
SALEP00854 SIPGVFAAGDVMD-HIY----RQAITSAGTGCMAALDAERYLDGLADASK----------
SALDC00926 SIPGVFAAGDVMD-HIY----RQAITSAGTGCMAALDAERYLDGLADASK----------
SALA400892 SIPGVFAAGDVMD-HIY----RQAITSAGTGCMAALDAERYLDGLADASK----------
SALG200876 SIPGVFAAGDVMD-HIY----RQAITSAGTGCMAALDAERYLDGLADASK----------
SALTS00888 SIPGVFAAGDVMD-HIY----RQAITSAGTGCMAALDAERYLDGLADASK----------
SALT400905 SIPGVFAAGDVMD-HIY----RQAITSAGTGCMAALDAERYLDGLADASK----------
SALPS01908 SIPGVFAAGDVMD-HIY----RQAITSAGTGCMAALDAERYLDGLADASK----------
SALT101040 SIPGVFAAGDVMD-HIY----RQAITSAGTGCMAALDAERYLDGLADASK----------
SALTD00960 SIPGVFAAGDVMD-HIY----RQAITSAGTGCMAALDAERYLDGLADASK----------
SALPB02472 SIPGVFAAGDVMD-HIY----RQAITSAGTGCMAALDAERYLDGLADASK----------
SALNS00928 SIPGVFAAGDVMD-HIY----RQAITSAGTGCMAALDAERYLDGLADASK----------
SALSV01000 SIPGVFAAGDVMD-HIY----RQAITSAGTGCMAALDAERYLDGLADASK----------
ECOS500812 SIPGVFAAGDVMD-HIY----RQAITSAGTGCMAALDAERYLDGLADAK-----------
ECOL600981 SIPGVFAAGDVMD-HIY----RQAITSAGTGCMAALDAERYLDGLADAK-----------
ECOL500883 SIPGVFAAGDVMD-HIY----RQAITSAGTGCMAALDAERYLDGLADAK-----------
ECOUT00876 SIPGVFAAGDVMD-HIY----RQAITSAGTGCMAALDAERYLDGLADAK-----------
ECOK100776 SIPGVFAAGDVMD-HIY----RQAITSAGTGCMAALDAERYLDGLADAK-----------
ECOSM02148 SIPGVFAAGDVMD-HIY----RQAITSAGTGCMAALDAERYLDGLADAK-----------
ECOLU01057 SIPGVFAAGDVMD-HIY----RQAITSAGTGCMAALDAERYLDGLADAK-----------
ECO7I02136 SIPGVFAAGDVMD-HIY----RQAITSAGTGCMAALDAERYLDGLADAK-----------
ECO8100828 SIPGVFAAGDVMD-HIY----RQAITSAGTGCMAALDAERYLDGLADAK-----------
ECO4500862 SIPGVFAAGDVMD-HIY----RQAITSAGTGCMAALDAERYLDGLADAK-----------
ECOAB00879 SIPGVFAAGDVMD-HIY----RQAITSAGTGCMAALDAERYLDGLADAK-----------
ECO4400961 SIPGVFAAGDVMD-HIY----RQAITSAGTGCMAALDAERYLDGLADAK-----------
ECOUM02550 SIPGVFAAGDVMD-HIY----RQAITSAGTGCMAALDAERYLDGLADAK-----------
ECOKI00869 SIPGVFAAGDVMD-HIY----RQAITSAGTGCMAALDAERYLDGLADAK-----------
ECOC100920 SIPGVFAAGDVMD-HIY----RQAITSAGTGCMAALDAERYLDGLADAK-----------
ECOC200920 SIPGVFAAGDVMD-HIY----RQAITSAGTGCMAALDAERYLDGLADAK-----------
ECO8N00803 SIPGVFAAGDVMD-HIY----RQAITSAGTGCMAALDAERYLDGLADAK-----------
ECO2700862 SIPGVFAAGDVMD-HIY----RQAITSAGTGCMAALDAERYLDGLADAK-----------
ECO2600974 SIPGVFAAGDVMD-HIY----RQAITSAGTGCMAALDAERYLDGLADAK-----------
ECOH100942 SIPGVFAAGDVMD-HIY----RQAITSAGTGCMAALDAERYLDGLADAK-----------
SHIB301848 SIPGVFAAGDVMD-HIY----RQAITSAGTGCMAALDAERYLDGLADAK-----------
ECOLI00846 SIPGVFAAGDVMD-HIY----RQAITSAGTGCMAALDAERYLDGLADAK-----------
ECO5700970 SIPGVFAAGDVMD-HIY----RQAITSAGTGCMAALDAERYLDGLADAK-----------
SHISS00756 SIPGVFAAGDVMD-HIY----RQAITSAGTGCMAALDAERYLDGLADAK-----------
SHIBS00679 SIPGVFAAGDVMD-HIY----RQAITSAGTGCMAALDAERYLDGLADAK-----------
SHIDS01857 SIPGVFAAGDVMD-HIY----RQAITSAGTGCMAALDAERYLDGLADAK-----------
ECO2400906 SIPGVFAAGDVMD-HIY----RQAITSAGTGCMAALDAERYLDGLADAK-----------
ECODH00784 SIPGVFAAGDVMD-HIY----RQAITSAGTGCMAALDAERYLDGLADAK-----------
ECOHS00922 SIPGVFAAGDVMD-HIY----RQAITSAGTGCMAALDAERYLDGLADAK-----------
ECOLC02627 SIPGVFAAGDVMD-HIY----RQAITSAGTGCMAALDAERYLDGLADAK-----------
ECO5E00946 SIPGVFAAGDVMD-HIY----RQAITSAGTGCMAALDAERYLDGLADAK-----------
ECOSE00936 SIPGVFAAGDVMD-HIY----RQAITSAGTGCMAALDAERYLDGLADAK-----------
ECO5500894 SIPGVFAAGDVMD-HIY----RQAITSAGTGCMAALDAERYLDGLADAK-----------
ECO8A00877 SIPGVFAAGDVMD-HIY----RQAITSAGTGCMAALDAERYLDGLADAK-----------
ECOBB00863 SIPGVFAAGDVMD-HIY----RQAITSAGTGCMAALDAERYLDGLADAK-----------
ECO5T00943 SIPGVFAAGDVMD-HIY----RQAITSAGTGCMAALDAERYLDGLADAK-----------
ECOBW00723 SIPGVFAAGDVMD-HIY----RQAITSAGTGCMAALDAERYLDGLADAK-----------
ECO1000919 SIPGVFAAGDVMD-HIY----RQAITSAGTGCMAALDAERYLDGLADAK-----------
ECOBD02604 SIPGVFAAGDVMD-HIY----RQAITSAGTGCMAALDAERYLDGLADAK-----------
ECOD102659 SIPGVFAAGDVMD-HIY----RQAITSAGTGCMAALDAERYLDGLADAK-----------

```
ECOBR00856       SIPGVFAAGDVMD-HIY----RQAITSAGTGCMAALDAERYLDGLADAK-----------
ECOLX02610       SIPGVFAAGDVMD-HIY----RQAITSAGTGCMAALDAERYLDGLADAK-----------
ECO1A00939       SIPGVFAAGDVMD-HIY----RQAITSAGTGCMAALDAERYLDGLADAK-----------
ECOCB01054       SIPGVFAAGDVMD-HIY----RQAITSAGTGCMAALDAERYLDGLADAK-----------
ECOKO02859       SIPGVFAAGDVMD-HIY----RQAITSAGTGCMAALDAERYLDGLADAK-----------
ECO1E03320       SIPGVFAAGDVMD-HIY----RQAITSAGTGCMAALDAERYLDGLADAK-----------
ECOLW01217       SIPGVFAAGDVMD-HIY----RQAITSAGTGCMAALDAERYLDGLADAK-----------
SHIFL01399       SIPGVFAAGDVMD-HIY----RQAITSAGTGCMAALDAERYLDGLADAK-----------
SHIF800759       SIPGVFAAGDVMD-HIY----RQAITSAGTGCMAALDAERYLDGLADAK-----------
SHIF200805       SIPGVFAAGDVMD-HIY----RQAITSAGTGCMAALDAERYLDGLADAK-----------
CITK802118       SVPGVFAAGDVMD-HIY----RQAITSAGTGCMAALDAERYLDGLADACK----------
CITRI00924       SVPGVFAAGDVMD-HIY----RQAITSAGTGCMAALDAERYLDGLADACK----------
CROS802385       SIPGVFAAGDVMD-HIY----RQAITSAGTGCMAALDAERYLDGLAEKQS----------
CROTZ01500       SIPGVFAAGDVMD-HIY----RQAITSAGTGCMAALDAERYLDGLAEKQS----------
ENTLS02865       SIPGVFAAGDVMD-HIY----RQAITSAGTGCMAALDAERYLDGLADKAK----------
ENT3801400       SIPGVFAAGDVMD-HIY----RQAITSAGTGCMAALDAERYLDGLAELSK----------
ENTAL01384       SIPGVFAAGDVMD-HIY----RQAITSAGTGCMAALDAERYLDGLAETGK----------
ENTCC02679       SIPGVFAAGDVMD-HIY----RQAITSAGTGCMAALDAERYLDGLAEQGK----------
                     .   *:            :   .   .

STRT101547       ------------------------------------------------------------
STRT201508       ------------------------------------------------------------
STRTD01356       ------------------------------------------------------------
STRTN01533       ------------------------------------------------------------
STRE500345       ------------------------------------------------------------
STRE801625       ------------------------------------------------------------
STREH01636       ------------------------------------------------------------
STREC01656       ------------------------------------------------------------
STREM01495       ------------------------------------------------------------
STRE401629       ------------------------------------------------------------
STRS700387       ------------------------------------------------------------
STRDG01631       ------------------------------------------------------------
STRP301388       ------------------------------------------------------------
STRPZ01205       ------------------------------------------------------------
STRPQ00464       ------------------------------------------------------------
STRPD01453       ------------------------------------------------------------
STRP601399       ------------------------------------------------------------
STRP801366       ------------------------------------------------------------
STRPF01453       ------------------------------------------------------------
STRPG00427       ------------------------------------------------------------
STRA300284       ------------------------------------------------------------
STRA500290       ------------------------------------------------------------
STRA100288       ------------------------------------------------------------
STRA200291       ------------------------------------------------------------
STRIC00348       ------------------------------------------------------------
STRPX00375       ------------------------------------------------------------
STRMD00432       ------------------------------------------------------------
STRS201734       ------------------------------------------------------------
STRSY01721       ------------------------------------------------------------
STRSX01553       ------------------------------------------------------------
STRSE01478       ------------------------------------------------------------
STREJ01635       ------------------------------------------------------------
STRGZ01544       ------------------------------------------------------------
STRS401593       ------------------------------------------------------------
LACGT00720       ------------------------------------------------------------
LACGL00738       ------------------------------------------------------------
STRSV01774       ------------------------------------------------------------
STRIJ00350       ------------------------------------------------------------
STROU01248       ------------------------------------------------------------
STRM601286       ------------------------------------------------------------
STRES00962       ------------------------------------------------------------
STRP701387       ------------------------------------------------------------
STRZT00756       ------------------------------------------------------------
STRP001079       ------------------------------------------------------------
STRZO01270       ------------------------------------------------------------
```

```
STRZ600815      ------------------------------------------------------------
STRET00826      ------------------------------------------------------------
STRPS01429      ------------------------------------------------------------
STRZN01287      ------------------------------------------------------------
STRR601306      ------------------------------------------------------------
STRP201217      ------------------------------------------------------------
STRZP01342      ------------------------------------------------------------
STRZI01226      ------------------------------------------------------------
STRPN01360      ------------------------------------------------------------
STRP401357      ------------------------------------------------------------
STRZJ01282      ------------------------------------------------------------
STRPJ01336      ------------------------------------------------------------
STRPI01436      ------------------------------------------------------------
MARHT00044      ------------------------------------------------------------
THEP300413      ------------------------------------------------------------
THEPX00884      ------------------------------------------------------------
THESX01857      ------------------------------------------------------------
THEM301788      ------------------------------------------------------------
THEIA01812      ------------------------------------------------------------
THETC00443      ------------------------------------------------------------
THESW01026      ------------------------------------------------------------
THEXL00360      ------------------------------------------------------------
THEID01127      ------------------------------------------------------------
THEOJ00147      ------------------------------------------------------------
DESAS01237      ------------------------------------------------------------
DESK701373      ------------------------------------------------------------
KYRT200729      ------------------------------------------------------------
STACT00410      ------------------------------------------------------------
STAS101952      ------------------------------------------------------------
STALH01969      ------------------------------------------------------------
STAEQ00422      ------------------------------------------------------------
STAES00543      ------------------------------------------------------------
STAAB00714      ------------------------------------------------------------
STAA500765      ------------------------------------------------------------
STAAM00757      ------------------------------------------------------------
STAAW00726      ------------------------------------------------------------
STAAS00733      ------------------------------------------------------------
STAAN00727      ------------------------------------------------------------
STAAC00807      ------------------------------------------------------------
STAA300727      ------------------------------------------------------------
STAA800734      ------------------------------------------------------------
STAA100752      ------------------------------------------------------------
STAA200774      ------------------------------------------------------------
STAA900759      ------------------------------------------------------------
STAAE00716      ------------------------------------------------------------
STAAT00767      ------------------------------------------------------------
STAAD00695      ------------------------------------------------------------
STAA000815      ------------------------------------------------------------
STAAH02407      ------------------------------------------------------------
STAAF00763      ------------------------------------------------------------
STAAK00744      ------------------------------------------------------------
STAAJ00703      ------------------------------------------------------------
STAAG00690      ------------------------------------------------------------
STAA400732      ------------------------------------------------------------
STAAR00788      ------------------------------------------------------------
LISSS02378      ------------------------------------------------------------
LISIN02590      ------------------------------------------------------------
LISW602421      ------------------------------------------------------------
EXISA00870      ------------------------------------------------------------
EXIS202358      ------------------------------------------------------------
EXIAB02180      ------------------------------------------------------------
OCEIH02462      ------------------------------------------------------------
BACIE01058      ------------------------------------------------------------
BACCJ03468      ------------------------------------------------------------
BACHD03507      ------------------------------------------------------------
```

```
BACPE03382      ------------------------------------------------------------
SOLSS00743      ------------------------------------------------------------
BACC600715      ------------------------------------------------------------
ANOFW02486      ------------------------------------------------------------
GEOKA03042      ------------------------------------------------------------
GEOSY02976      ------------------------------------------------------------
GEOTN02952      ------------------------------------------------------------
GEOSW02588      ------------------------------------------------------------
GEOS000381      ------------------------------------------------------------
GEOTC00378      ------------------------------------------------------------
BACMD04906      ------------------------------------------------------------
BACMQ04908      ------------------------------------------------------------
BACWK04830      ------------------------------------------------------------
BACAN04889      ------------------------------------------------------------
BACC105210      ------------------------------------------------------------
BACC705051      ------------------------------------------------------------
BACC005112      ------------------------------------------------------------
BACC305043      ------------------------------------------------------------
BACAC05232      ------------------------------------------------------------
BACAA04720      ------------------------------------------------------------
BACT005085      ------------------------------------------------------------
BACC205124      ------------------------------------------------------------
BACC405064      ------------------------------------------------------------
BACT104712      ------------------------------------------------------------
BACLD03769      ------------------------------------------------------------
BACPZ03376      ------------------------------------------------------------
BACSU03600      ------------------------------------------------------------
BACST01653      ------------------------------------------------------------
BACPT03541      ------------------------------------------------------------
LEUGG00661      ------------------------------------------------------------
LEUGJ00629      ------------------------------------------------------------
LEUCJ00485      ------------------------------------------------------------
LACAR00657      ------------------------------------------------------------
LACA300647      ------------------------------------------------------------
LACAL00643      ------------------------------------------------------------
LACKZ00964      ------------------------------------------------------------
LACRJ00357      ------------------------------------------------------------
LACRD00361      ------------------------------------------------------------
LACRS01501      ------------------------------------------------------------
LACSM00455      ------------------------------------------------------------
LACRG00878      ------------------------------------------------------------
LACRL00939      ------------------------------------------------------------
LACC300844      ------------------------------------------------------------
LACCZ00824      ------------------------------------------------------------
LACCB00988      ------------------------------------------------------------
LACCD01016      ------------------------------------------------------------
LACCC01014      ------------------------------------------------------------
LACBN01277      ------------------------------------------------------------
LACBA00605      ------------------------------------------------------------
LACPL00650      ------------------------------------------------------------
LACPJ00628      ------------------------------------------------------------
LACPS00585      ------------------------------------------------------------
PEDCP00497      ------------------------------------------------------------
CARS100359      ------------------------------------------------------------
AERUA00263      ------------------------------------------------------------
ELUMP00590      ------------------------------------------------------------
SPHPG02940      ------------------------------------------------------------
SPHGB01868      ------------------------------------------------------------
TREPZ00273      ------------------------------------------------------------
TREAZ03414      ------------------------------------------------------------
SPITD00734      ------------------------------------------------------------
SPITZ01373      ------------------------------------------------------------
TREPA00803      ------------------------------------------------------------
TREPS00802      ------------------------------------------------------------
TREPC00747      ------------------------------------------------------------
```

```
TREPM00823      ------------------------------------------------------------
TREPD00824      ------------------------------------------------------------
TREPU00781      ------------------------------------------------------------
ENCCU00216      ------------------------------------------------------------
HELM100964      ------------------------------------------------------------
HELCP01490      ------------------------------------------------------------
ARCFU01526      ------------------------------------------------------------
FERPA02445      ------------------------------------------------------------
ARCVS01910      ------------------------------------------------------------
METEZ00677      ------------------------------------------------------------
METHD00869      ------------------------------------------------------------
METMA02304      ------------------------------------------------------------
METAC01311      ------------------------------------------------------------
KOSOT00298      ------------------------------------------------------------
MARPK01613      ------------------------------------------------------------
SLAHD02439      ------------------------------------------------------------
FILAD00976      ------------------------------------------------------------
BUTPB02463      ------------------------------------------------------------
CLOPH00250      ------------------------------------------------------------
CLOSW00592      ------------------------------------------------------------
LACFC00207      ------------------------------------------------------------
CRYCD00853      ------------------------------------------------------------
EGGLE01847      ------------------------------------------------------------
PYRFU01410      ------------------------------------------------------------
PYRHO01476      ------------------------------------------------------------
PYRAB00730      ------------------------------------------------------------
PYRSN00014      ------------------------------------------------------------
THEGJ00181      ------------------------------------------------------------
THEKO02097      ------------------------------------------------------------
THEON01610      ------------------------------------------------------------
THES401476      ------------------------------------------------------------
SYNWW02368      ------------------------------------------------------------
UNCTG00012      ------------------------------------------------------------
THEA101460      ------------------------------------------------------------
DENA201647      ------------------------------------------------------------
DEFDS00488      ------------------------------------------------------------
CALNY01292      ------------------------------------------------------------
SYNGF01652      ------------------------------------------------------------
DESB201123      ------------------------------------------------------------
DEIPM00845      PA-------------------------------------TATD-----------------
DEIRA01924      ------------------------------------------------------------
DEIML01437      ------------------------------------------------------------
DEIGD02532      ------------------------------------------------------------
DEIDV00622      ------------------------------------------------------------
TRURR01079      PE-------------------------------------AVS------------------
THETG01863      ------------------------------------------------------------
THET201543      ------------------------------------------------------------
THET801911      ------------------------------------------------------------
OCEP502115      ------------------------------------------------------------
DEHLB00585      ------------------------------------------------------------
DEHMG00439      ------------------------------------------------------------
DEHMB00497      ------------------------------------------------------------
DEHMC00427      ------------------------------------------------------------
THELD00726      PE-------------------------------------HAFIFLYSSIDEAQMSLVSQL
ANAMD00624      PE-------------------------------------KVVSFFYSSIEPEQIKLATDI
THEAS00815      PP-------------------------------------RVNALFYSSIDQEQVRLVGRV
AMICL00707      PE-------------------------------------RVVAFFYSSIVESQVRLSNAV
CLOCE01684      ------------------------------------------------------------
HYDS000616      ------------------------------------------------------------
HYDTT00224      ------------------------------------------------------------
PELTS01405      ------------------------------------------------------------
DESRL02179      ------------------------------------------------------------
BORBP00506      ------------------------------------------------------------
BORAP00518      ------------------------------------------------------------
BORBU00514      ------------------------------------------------------------
```

```
BORBZ00490      ------------------------------------------------------------
BORBN00493      ------------------------------------------------------------
BORRA00486      ------------------------------------------------------------
BORDL00498      ------------------------------------------------------------
BORHD00497      ------------------------------------------------------------
BORT900497      ------------------------------------------------------------
METKA01560      ------------------------------------------------------------
MYCA500361      ------------------------------------------------------------
METVS00145      ------------------------------------------------------------
METOI01431      ------------------------------------------------------------
MYCHN00051      ------------------------------------------------------------
MYCSL00465      ------------------------------------------------------------
MYCS300420      ------------------------------------------------------------
DESK101258      ------------------------------------------------------------
STAHD01472      ------------------------------------------------------------
THEC100270      ------------------------------------------------------------
NANEQ00478      ------------------------------------------------------------
CALLD01225      ------------------------------------------------------------
SULSO02155      ------------------------------------------------------------
SULS900210      ------------------------------------------------------------
SULIA00208      ------------------------------------------------------------
SULIM00207      ------------------------------------------------------------
SULIK00225      ------------------------------------------------------------
IGNH400907      ------------------------------------------------------------
KORCO01040      PS-------------------------------------RTS------------------
THESM01133      ------------------------------------------------------------
THEBM01534      ------------------------------------------------------------
METST01363      ------------------------------------------------------------
METTH00703      ------------------------------------------------------------
METSL02406      ------------------------------------------------------------
METLA02406      ------------------------------------------------------------
METPW00194      ------------------------------------------------------------
RUBXD00226      KDRSPAG-----SG----------------------------------------------
RHOM400178      ------------------------------------------------------------
GARV400051      ------------------------------------------------------------
BIFAP01644      ------------------------------------------------------------
SEGRD00014      ------------------------------------------------------------
GORB404535      AGA---------------------------------------------------------
GORPV04920      DAHS--------------------------------------------------------
MYCA904903      ------------------------------------------------------------
MYCSS05356      ------------------------------------------------------------
MYCSJ05684      ------------------------------------------------------------
MYCSK05387      ------------------------------------------------------------
MYCS206581      ------------------------------------------------------------
MYCCN05158      --D------SDLIGAQQ-------------------------------------------
MYCVP05890      --D------SDLIGAQQ-------------------------------------------
MYCGI00775      TDD------TDLIGAQQ-------------------------------------------
MYCSR04994      TDD------TDLIGAQQ-------------------------------------------
AMYSD04545      ------------------------------------------------------------
MYCLE02681      DSTDTTDWSTAMTDAKNAGVTIEVTDASFFADVLSSNKPVLVDFWATWCGPCKM-VAPVL
MYCLB02681      DSTDTTDWSTAMTDAKNAGVTIEVTDASFFADVLSSNKPVLVDFWATWCGPCKM-VAPVL
MYCSD04323      PATETLM------GAPQ-------------------------------------------
MYCPA04306      GSTDTLI------GAPQ-------------------------------------------
MYCA105023      GSTDTLI------GAPQ-------------------------------------------
MYCUA04107      DSTDTLI------GAQQ-------------------------------------------
MYCMM05378      DSTDTLI------GAQQ-------------------------------------------
MYCA003905      DSTDALI------GAQR-------------------------------------------
MYCTU03943      DSTDALI------GAQR-------------------------------------------
MYCTF03846      DSTDALI------GAQR-------------------------------------------
MYCTA03980      DSTDALI------GAQR-------------------------------------------
MYCTK04010      DSTDALI------GAQR-------------------------------------------
MYCTC03612      DSTDALI------GAQR-------------------------------------------
MYCTD03548      DSTDALI------GAQR-------------------------------------------
MYCCP03898      DSTDALI------GAQR-------------------------------------------
MYCBO02863      DSTDALI------GAQR-------------------------------------------
```

```
MYCBP03913      DSTDALI------GAQR-------------------------------------------
MYCBT03913      DSTDALI------GAQR-------------------------------------------
TSUPD04078      ------------------------------------------------------------
ACTMD06897      GYGEPAT------TG---------------------------------------------
SACES08402      GYSEPVP------TS---------------------------------------------
SACVD03814      GYATRAN-----------------------------------------------------
AMYMU09190      GYGPGTN-----------------------------------------------------
AMYMS10178      GYGPGTN-----------------------------------------------------
PSEUX06421      GYGTAAP------EAAAAAH----------------------------------------
NOCFA05681      GEPVAVN------AN---------------------------------------------
NOCCG05476      GQPVAVP------AN---------------------------------------------
RHOE406010      GHTVDVV------GAP--------------------------------------------
RHOOB03468      GSPVDA------------------------------------------------------
RHOE104515      GEPVAVD------A----------------------------------------------
CORDI02302      ------------------------------------------------------------
CORD202219      ------------------------------------------------------------
CORDL02208      ------------------------------------------------------------
CORDJ02208      ------------------------------------------------------------
CORDH02221      ------------------------------------------------------------
CORD702316      ------------------------------------------------------------
CORD302337      ------------------------------------------------------------
CORDD02235      ------------------------------------------------------------
CORDV02170      ------------------------------------------------------------
CORDW02254      ------------------------------------------------------------
CORDK02230      ------------------------------------------------------------
COREF02870      ------------------------------------------------------------
CORGL03082      ------------------------------------------------------------
CORGK02974      ------------------------------------------------------------
CORGB03038      ------------------------------------------------------------
CORK402006      ------------------------------------------------------------
CORJK02028      ------------------------------------------------------------
CORVD02951      ------------------------------------------------------------
ARCHD01706      ------------------------------------------------------------
THET101205      ------------------------------------------------------------
PROAC02247      ------------------------------------------------------------
PROAS02302      ------------------------------------------------------------
CAERE29798      ------------------------------------------------------------
CELFA03749      APVLVQEA----------------------------------------------------
JONDD02484      ------------------------------------------------------------
XYLCX03324      R-----------------------------------------------------------
ACIC102144      ------------------------------------------------------------
FRADG04042      PAEAVAGAAAS-------------------------------------------------
FRASU07065      ------------------------------------------------------------
FRASN07115      PRVPAPELAATT------------------------------------------------
FRASC04424      PRVVTPTPQPA-------------------------------------------------
FRAAA06712      PRVSTPAPQPA-------------------------------------------------
KYTSD02521      ERVPGDVPA---------------------------------------------------
KINRD04462      ETETPDVPDSETTDRPQPRA----------------------------------------
NOCDD04789      ------------------------------------------------------------
NOCAA01941      ------------------------------------------------------------
STRRD08913      ------------------------------------------------------------
THECD04863      ------------------------------------------------------------
THEBD03528      ------------------------------------------------------------
CATAD08894      ------------------------------------------------------------
KRIFD06917      ATEPVSV-----------------------------------------------------
KITSK03730      V-----------------------------------------------------------
STRBB05366      ------------------------------------------------------------
STRVP03660      ------------------------------------------------------------
STRSW04381      TV----------------------------------------------------------
STRGG03562      PAV---------------------------------------------------------
STRFA03098      PAV---------------------------------------------------------
STRAW04303      TV----------------------------------------------------------
STRCO02929      AV----------------------------------------------------------
STRHJ05105      AV----------------------------------------------------------
MONBE04991      ------------------------------------------------------------
```

```
CHLRE01313      AA----------------------APVADGNL----------------------------
MEDTR25591      ------------------------------------------------------------
SOLLC13750      ------------------------------------------------------------
PRUPE10733      ------------------------------------------------------------
MANES18605      ------------------------------------------------------------
THECC00884      ------------------------------------------------------------
PHYPA31147      TS----------------------NGTGEGPSTIV-KPPMVMANGLSRV-----------
AMBTC19471      ------------------------------------------------------------
MUSAC26038      ------------------------------------------------------------
MUSAM33177      ------------------------------------------------------------
SETIT03079      ------------------------------------------------------------
ORYBR12195      ------------------------------------------------------------
COCLU07729      ---------------QAEKS----QTNGVVPEY-R-SNPLL-------------------
PHANO13702      ---------------QAEKS----QTNGVVPEY-R-SNPLL-------------------
PHAND10804      ---------------QAEKS----QTNGVVPEY-R-SNPLL-------------------
AURPU02089      ---------------EVKKS----KTDGDAPEY-R-QNPLL-------------------
ZYMTR07711      ---------------KGAKK----GDSSEAPEY-R-SNPLL-------------------
DICPU05926      ------------------------------------------------------------
ENTHI00522      ------------------------------------------------------------
LEPBA02231      ------------------------------------------------------------
LEPBP02301      ------------------------------------------------------------
LEPBL01462      ------------------------------------------------------------
LEPBJ01276      ------------------------------------------------------------
LEPIN02475      ------------------------------------------------------------
LEPII01996      ------------------------------------------------------------
LEPIC01426      ------------------------------------------------------------
SPIAZ00697      ------------------------------------------------------------
PENRW10140      ------------------------------------------------------------
PENCH09104      ------------------------------------------------------------
EURHE07269      AEGSTIQPAAQEVPGTERRK----DPQGGAAEY-Q-QNPLL-------------------
ASPAC07301      --KSTVQPT-QEV-NG-IKK----ESEGATAEY-K-SNPLL-------------------
EMENI10387      ------------------------------------------------------------
EMEND02596      ------------------------------------------------------------
ASPTN06742      N---------------------------TGTEY-K-SNPLL-------------------
ASPCL04014      GDRSTVQPAAQEVDGE-VKK----DPNGAVPEY-K-SNPLL-------------------
ASPFU05647      ADNSTIQPAAQEVNGD-VKK----DPKGAVPEY-K-SNPLL-------------------
NEOFI00452      ADNSTVQPAAQEVNGE-VKK----DPKGAVAEY-K-SNPLL-------------------
CRYPA10563      -----------------N-W----GEKA--------------------------------
BLUGR03498      ------------------------------------------------------------
SCLS112814      -----------------AEK----GSNVVVPEY-R-SNPLL-------------------
MAGGR04266      ------------------------------------------------------------
NEUCR01575      -----------------AVQ----GNL---------------------------------
NEUT908941      -----------------AVQ----GNL---------------------------------
VERDA02342      -----------------AR-----------------------------------------
COLSU12486      -----------------PN-----------------------------------------
HYPAI01684      ------------------------------------------------------------
HYPVG06080      ------------------------------------------------------------
HYPJE05895      ------------------------------------------------------------
NECHA05020      ------------------------------------------------------------
FUSO415847      -----------------LN-----------------------------------------
GIBZA01026      -----------------PN-----------------------------------------
SCHPO04025      ------------------------------------------------------------
YARLI03635      ------------------------------------------------------------
ASHGO00946      ------------------------------------------------------------
KLULA02190      ------------------------------------------------------------
ZYGRO00676      ------------------------------------------------------------
DEKBR01813      ------------------------------------------------------------
PICPG04776      ------------------------------------------------------------
CANTE00916      ------------------------------------------------------------
LODEL03891      ------------------------------------------------------------
DEBHA05546      ------------------------------------------------------------
SPAPN03477      ------------------------------------------------------------
CANAW04800      ------------------------------------------------------------
PICST04701      ------------------------------------------------------------
PUCGT10887      V-----VPQP-GYLGTDK-------------P----------------------------
```

```
PUCGR11813      V-----VPQP-GYLGTDK-------------P----------------------------
PHYBL11006      ------------------------------------------------------------
USTMA03757      --------AN-HYTGTDK-------------A----------------------------
USTHO04132      --------AN-HYTGTDK-------------E----------------------------
WALSE04527      ------------------------------------------------------------
TREME07701      H-----VPAN-GYMGTDK-------------E----------------------------
AURST04751      ------------------------------------------------------------
FOMME10177      ------------------------------------------------------------
CONPW06392      ------------------------------------------------------------
STEHR07076      ------------------------------------------------------------
HETAN06295      ------------------------------------------------------------
GLOTR06982      D-----LPKH-R------------------------------------------------
PUNST01981      K-----LPED-EAMKIH-------------------------------------------
LACBI02877      ------------------------------------------------------------
COPCI16429      ------------------------------------------------------------
DICSQ11618      ------------------------------------------------------------
TRAVS13180      ------------------------------------------------------------
WOLCO03584      ------------------------------------------------------------
FOMPI05979      ------------------------------------------------------------
PHLGI10219      ------------------------------------------------------------
PHACH05757      ------------------------------------------------------------
RICTY00419      ------------------------------------------------------------
RICPR00429      ------------------------------------------------------------
RICPP00461      ------------------------------------------------------------
RICBR00434      ------------------------------------------------------------
RICB800988      ------------------------------------------------------------
RICCK00600      ------------------------------------------------------------
RICAH00614      ------------------------------------------------------------
RICAC00782      ------------------------------------------------------------
RICFE00656      ------------------------------------------------------------
RICMS00042      ------------------------------------------------------------
RICM500453      ------------------------------------------------------------
RICR300665      ------------------------------------------------------------
RICAG00651      ------------------------------------------------------------
RICP300630      ------------------------------------------------------------
RICRS00632      ------------------------------------------------------------
RICRO00663      ------------------------------------------------------------
RICCN00618      ------------------------------------------------------------
RICPT00620      ------------------------------------------------------------
RICAE00497      ------------------------------------------------------------
RICJY00467      ------------------------------------------------------------
RICPU00072      ------------------------------------------------------------
RICS100537      ------------------------------------------------------------
BARBK00891      ------------------------------------------------------------
BARVW00932      ------------------------------------------------------------
BART100617      ------------------------------------------------------------
BARGA00466      ------------------------------------------------------------
BARHE01107      ------------------------------------------------------------
BARQU00895      ------------------------------------------------------------
OCHA401642      ------------------------------------------------------------
BRUAB01390      ------------------------------------------------------------
BRUA201507      ------------------------------------------------------------
BRUA101339      ------------------------------------------------------------
BRUSU01458      ------------------------------------------------------------
BRUME00510      ------------------------------------------------------------
BRUSI01461      ------------------------------------------------------------
BRUC201446      ------------------------------------------------------------
BRUMC01440      ------------------------------------------------------------
BRUMB01421      ------------------------------------------------------------
BRUM501487      ------------------------------------------------------------
BRUO201285      ------------------------------------------------------------
RHILO01973      ------------------------------------------------------------
CHESB02097      ------------------------------------------------------------
METPB00996      ------------------------------------------------------------
METEP01063      ------------------------------------------------------------
```

```
METEA00810      ------------------------------------------------------------
METED01453      ------------------------------------------------------------
METS403554      ------------------------------------------------------------
METNO05482      ------------------------------------------------------------
METSZ03234      ------------------------------------------------------------
BEII900056      ------------------------------------------------------------
METSB02743      ------------------------------------------------------------
MAGMM00401      ------------------------------------------------------------
HYPNA00542      ------------------------------------------------------------
KETVY00902      ------------------------------------------------------------
KETVW00472      ------------------------------------------------------------
ROSDO03242      ------------------------------------------------------------
ROSLO02615      ------------------------------------------------------------
RUEPO00888      ------------------------------------------------------------
RUEST00613      ------------------------------------------------------------
PHAIB02390      ------------------------------------------------------------
PARDP02130      ------------------------------------------------------------
DINSH02620      NAA------E--------------------------------------------------
RHOCB02768      ------------------------------------------------------------
RHOS500248      ------------------------------------------------------------
RHOS400150      ------------------------------------------------------------
RHOS100227      ------------------------------------------------------------
RHOSK02952      ------------------------------------------------------------
MIDMI00790      ------------------------------------------------------------
ACEP301595      ------------------------------------------------------------
MICAA01566      ------------------------------------------------------------
TISMK03676      ------------------------------------------------------------
AZOL402409      DPS------PAGT------W----------------------------------------
PSEUV04348      ------------------------------------------------------------
HIRBI01095      ------------------------------------------------------------
PARL102241      RQA------ETA------------------------------------------------
MARMM02139      ------------------------------------------------------------
PHEZH02588      EAQ------KIGA------W----------------------------------------
CAUCR02826      EAN------KIGV------W----------------------------------------
CAUCN02939      EAN------KIGV------W----------------------------------------
CAUST00891      EAN------KIGV------W----------------------------------------
PARBH01522      ------------------------------------------------------------
PELHB02084      ------------------------------------------------------------
HYPDA03273      ------------------------------------------------------------
HYPSM04642      ------------------------------------------------------------
OLICO01107      ------------------------------------------------------------
OLICM02790      ------------------------------------------------------------
RHOPS01446      ------------------------------------------------------------
RHOPA04062      ------------------------------------------------------------
RHOPT04533      ------------------------------------------------------------
RHOPX04235      ------------------------------------------------------------
BRADU07321      ------------------------------------------------------------
BRASO01239      ------------------------------------------------------------
BRASB06352      ------------------------------------------------------------
RHOPB03773      ------------------------------------------------------------
NITWN02324      ------------------------------------------------------------
NITHX02707      ------------------------------------------------------------
AZOC501143      ------------------------------------------------------------
XANP202679      ------------------------------------------------------------
CHLTF01983      ------------------------------------------------------------
IGNAJ02236      ------------------------------------------------------------
MELRP00403      ------------------------------------------------------------
ANADF00468      ------------------------------------------------------------
CHLCH01125      ------------------------------------------------------------
PELPB01419      ------------------------------------------------------------
CHLL200781      ------------------------------------------------------------
CHLTE00830      ------------------------------------------------------------
CHLP800854      ------------------------------------------------------------
CHLL701225      ------------------------------------------------------------
CHLPM00993      ------------------------------------------------------------
```

```
WOLTR00562      ------------------------------------------------------------
WOLPP00135      ------------------------------------------------------------
WOLPM00650      ------------------------------------------------------------
WOLWR00416      ------------------------------------------------------------
EHRCR00696      ------------------------------------------------------------
ANAMM00345      ------------------------------------------------------------
ANAMF00341      ------------------------------------------------------------
ANAPZ00610      ------------------------------------------------------------
NEOSM00537      ------------------------------------------------------------
NEORI00513      ------------------------------------------------------------
PELUB00076      ------------------------------------------------------------
PELSM00736      ------------------------------------------------------------
PUNMI01409      ------------------------------------------------------------
ASTEC00651      ------------------------------------------------------------
ZYMMT00154      ------------------------------------------------------------
ZYMMO00984      ------------------------------------------------------------
ZYMMA00176      ------------------------------------------------------------
ZYMMN00183      ------------------------------------------------------------
SPHAL00143      ------------------------------------------------------------
SPHWW03978      ------------------------------------------------------------
SPHJU02313      ------------------------------------------------------------
NOVAD02319      ------------------------------------------------------------
ERYLH00934      AE----------------------------------------------------------
GRABC00720      ------------------------------------------------------------
GLUDA03075      ------------------------------------------------------------
KOMMN00774      ------------------------------------------------------------
HALVD01097      ------------------------------------------------------------
HALHT01699      DD----------------------------------------------------------
METI401038      ------------------------------------------------------------
PLAL201612      ------------------------------------------------------------
GEMAT01983      STA------PGGH------S----A-----------------------------------
CYAAP02456      ------------------------------------------------------------
CHLPN00303      ------------------------------------------------------------
CHLPP00439      ------------------------------------------------------------
CHLPE00727      ------------------------------------------------------------
CHLTR00101      ------------------------------------------------------------
CHLTA00102      ------------------------------------------------------------
CHLTJ00101      ------------------------------------------------------------
CHLTD00098      ------------------------------------------------------------
CHLT700102      ------------------------------------------------------------
CHLT000101      ------------------------------------------------------------
CHLT500104      ------------------------------------------------------------
CHLTL00098      ------------------------------------------------------------
CHLTG00102      ------------------------------------------------------------
CHLTS00101      ------------------------------------------------------------
CHLT900102      ------------------------------------------------------------
CHLTZ00101      ------------------------------------------------------------
CHLT400432      ------------------------------------------------------------
CHLT100103      ------------------------------------------------------------
CHLT200350      ------------------------------------------------------------
CHLTB00350      ------------------------------------------------------------
CHLTC00378      ------------------------------------------------------------
SIMNZ00831      ------------------------------------------------------------
PARUW01706      ------------------------------------------------------------
WADCW00943      ------------------------------------------------------------
SINAD04980      ------------------------------------------------------------
BIFLB01558      ------------------------------------------------------------
BIFAB00498      ------------------------------------------------------------
BIFAV01561      ------------------------------------------------------------
BIFAS01557      ------------------------------------------------------------
BIFA001518      ------------------------------------------------------------
BIFBA01814      ------------------------------------------------------------
BIFAA01619      ------------------------------------------------------------
BIFDB02112      ------------------------------------------------------------
TERSS03091      ------------------------------------------------------------
```

GRATM03160	--
GRAMM03836	--
AKKM801247	--
OPITP04130	--
CORAD01794	--
BUCCC00180	--
CENSY00347	--
NITMS00668	--
MEIRD02823	--
HERA203780	--
CHLAA02018	--
CHLSY02175	--
CHLAD02599	--
CALAS01745	MTP------RAPA------M----A-----------------------------------
ANATU01696	--
SULMS00235	--
BLASB00041	--
BLASP00551	--
AZOPC00437	--
LEPBD01878	--
SALRD02577	EA--
SALRM02866	EA--
RIEPU00166	--
ORITB00015	--
ORITI01234	--
PREMB01541	--
PREDF01155	--
PREI702011	--
ALIFI01035	--
ODOSD00362	--
PRER201250	--
PALPW00206	--
PORGI00953	--
PORG301168	--
BACV803426	--
BACT601486	--
BACTN04290	--
BACFR01032	--
BACFN00913	--
BACF600963	--
OWEHD03413	--
PSYTT00390	--
NONDD00011	--
ROBBH02711	--
CELAD02592	--
CELLC02474	--
MARSH03072	--
MURRD00159	--
ZOBGA00152	--
GRAFK00949	V---
ZUNPS02344	--
AEQSU02079	--
HALH105745	--
SAPGL02893	--
AMOA500014	--
FLAIG01802	--
FLACA02326	--
FLAJ100198	--
FLABF01528	--
CAPOD01333	--
CAPCC00414	--
FLELS01898	--
SOLCM01166	--
PEDHD01598	--
SPHS203207	--

```
LEAB401188        ------------------------------------------------------------
EMTOG00092        ------------------------------------------------------------
SPILD01737        ------------------------------------------------------------
DYAFD00084        ------------------------------------------------------------
CYCMS03063        ------------------------------------------------------------
ECHVK03326        ------------------------------------------------------------
BELBD02542        ------------------------------------------------------------
NITGG02112        ------------------------------------------------------------
THEM700111        ------------------------------------------------------------
CREAS01350        ------------------------------------------------------------
TURPD02772        ------------------------------------------------------------
SORC507603        ------------------------------------------------------------
BDEBA00337        ------------------------------------------------------------
STIAD02752        ------------------------------------------------------------
MYXXD01876        ------------------------------------------------------------
MYXFH03454        ------------------------------------------------------------
CORCM01960        ------------------------------------------------------------
MYXSD02209        ------------------------------------------------------------
LEPFC02126        ------------------------------------------------------------
LEPFM02294        ------------------------------------------------------------
SOLUE00582        ------------------------------------------------------------
KORVE01333        ------------------------------------------------------------
ACIC502553        ------------------------------------------------------------
ACIFD00030        ------------------------------------------------------------
HALMS01402        ------------------------------------------------------------
CHLPD01133        ------------------------------------------------------------
CHLPB01137        ------------------------------------------------------------
PROA200897        ------------------------------------------------------------
WIGBR00492        ------------------------------------------------------------
BUCA500289        ------------------------------------------------------------
BUCAI00292        ------------------------------------------------------------
BUCAF00306        ------------------------------------------------------------
BUCAT00287        ------------------------------------------------------------
BUCA000302        ------------------------------------------------------------
BAUCH00290        ------------------------------------------------------------
BLOVB00369        ------------------------------------------------------------
BLOFL00367        ------------------------------------------------------------
BLOPB00375        ------------------------------------------------------------
BUCAP00289        ------------------------------------------------------------
COXBU01001        ------------------------------------------------------------
COXBN01188        ------------------------------------------------------------
COXBR01117        ------------------------------------------------------------
COXB200717        ------------------------------------------------------------
COXB100914        ------------------------------------------------------------
FRAP200241        ------------------------------------------------------------
FRANT01012        ------------------------------------------------------------
FRACN00522        ------------------------------------------------------------
FRATT00465        ------------------------------------------------------------
FRAT100465        ------------------------------------------------------------
FRATE00462        ------------------------------------------------------------
FRATW01216        ------------------------------------------------------------
FRATM01110        ------------------------------------------------------------
FRACF00566        ------------------------------------------------------------
FRATO01212        ------------------------------------------------------------
FRATH01486        ------------------------------------------------------------
FRATF01216        ------------------------------------------------------------
FRATN00564        ------------------------------------------------------------
ACIF500512        ------------------------------------------------------------
ACIF200362        ------------------------------------------------------------
DECAR01284        ------------------------------------------------------------
NEIG100525        ------------------------------------------------------------
NEIG201314        ------------------------------------------------------------
NEIM800926        ------------------------------------------------------------
NEIMP01199        ------------------------------------------------------------
NEIMB01212        ------------------------------------------------------------
```

```
NEIMF01143      ------------------------------------------------------------
NEIML01119      ------------------------------------------------------------
NEIMM00780      ------------------------------------------------------------
NEIMH00827      ------------------------------------------------------------
NEIMG01168      ------------------------------------------------------------
NEIMN01254      ------------------------------------------------------------
NEIMO00810      ------------------------------------------------------------
NEIM701229      ------------------------------------------------------------
NEIMA01334      ------------------------------------------------------------
NEIMW01125      ------------------------------------------------------------
DICNV01037      ------------------------------------------------------------
VESOH00047      ------------------------------------------------------------
RUTMC00043      ------------------------------------------------------------
HALHL02255      ------------------------------------------------------------
PELPD03177      ------------------------------------------------------------
GEOS804030      ------------------------------------------------------------
GEOBB03636      ------------------------------------------------------------
GEOSM03700      ------------------------------------------------------------
HALNC00046      ------------------------------------------------------------
HAEPS00887      ------------------------------------------------------------
GALAU02285      ------------------------------------------------------------
HISS201000      ------------------------------------------------------------
HAES101151      ------------------------------------------------------------
PASMU00573      ------------------------------------------------------------
PASMH00579      ------------------------------------------------------------
ACTSZ01560      ------------------------------------------------------------
AGGAN00985      ------------------------------------------------------------
NITHN03181      ------------------------------------------------------------
NITOC00311      ------------------------------------------------------------
NITWC02498      ------------------------------------------------------------
METNJ00648      ------------------------------------------------------------
METFJ01616      ------------------------------------------------------------
ALKEH00244      ------------------------------------------------------------
MARMS03166      ------------------------------------------------------------
MARM102688      ------------------------------------------------------------
THICR00763      ------------------------------------------------------------
THICA00974      ------------------------------------------------------------
THIV600851      ------------------------------------------------------------
THISH01973      ------------------------------------------------------------
METAA00813      ------------------------------------------------------------
META200813      ------------------------------------------------------------
METMM02895      ------------------------------------------------------------
FRAAD00813      ------------------------------------------------------------
XYLFA01416      ------------------------------------------------------------
XYLFT00623      ------------------------------------------------------------
XYLF200667      ------------------------------------------------------------
XYLFG01622      ------------------------------------------------------------
XYLFM00690      ------------------------------------------------------------
PSEUP01680      ------------------------------------------------------------
STRM501972      ------------------------------------------------------------
PSEUU01524      ------------------------------------------------------------
XANAP01407      ------------------------------------------------------------
XANCP01918      ------------------------------------------------------------
XANC802154      ------------------------------------------------------------
XANCB02263      ------------------------------------------------------------
XANOR02379      ------------------------------------------------------------
XANOM02295      ------------------------------------------------------------
XANOP02318      ------------------------------------------------------------
XANAC01951      ------------------------------------------------------------
CYCSP01155      ------------------------------------------------------------
GEOLS03275      ------------------------------------------------------------
GEOUR03858      ------------------------------------------------------------
GEODF00692      ------------------------------------------------------------
GEOSL00482      ------------------------------------------------------------
GEOSK00469      ------------------------------------------------------------
```

```
GEOMG02998      ------------------------------------------------------------
MORCR00228      ------------------------------------------------------------
ACIAD00798      ------------------------------------------------------------
ACIBC00821      ------------------------------------------------------------
ACIBY02725      ------------------------------------------------------------
ACIB302691      ------------------------------------------------------------
ACIB500882      ------------------------------------------------------------
ACIB100850      ------------------------------------------------------------
ACIBD00843      ------------------------------------------------------------
ACIBS02229      ------------------------------------------------------------
ACICP00137      ------------------------------------------------------------
ACISD03052      ------------------------------------------------------------
LEGLN02402      ------------------------------------------------------------
LEGPA01726      ------------------------------------------------------------
LEGPH00846      ------------------------------------------------------------
LEGPC01171      ------------------------------------------------------------
LEGP201857      ------------------------------------------------------------
LEGPL01727      ------------------------------------------------------------
COLP302668      ------------------------------------------------------------
KANKD01106      ------------------------------------------------------------
IDILO00659      ------------------------------------------------------------
PSEU901284      ------------------------------------------------------------
PSEA602346      ------------------------------------------------------------
ALTSS01871      ------------------------------------------------------------
ALTMD01611      ------------------------------------------------------------
ALTME01705      ------------------------------------------------------------
ALTMB01797      ------------------------------------------------------------
ALTMS01652      ------------------------------------------------------------
SACD201683      ------------------------------------------------------------
TERTT01748      ------------------------------------------------------------
SIMAS00609      ------------------------------------------------------------
ALCDB01962      ------------------------------------------------------------
CHRSD02939      ------------------------------------------------------------
HALED02811      ------------------------------------------------------------
SIDLE02655      ------------------------------------------------------------
GALCS01621      ------------------------------------------------------------
LARHH01446      ------------------------------------------------------------
CHRVO02807      ------------------------------------------------------------
PSEUL01648      ------------------------------------------------------------
NITEU01859      ------------------------------------------------------------
NITEC00745      ------------------------------------------------------------
NITMU00023      ------------------------------------------------------------
NITSI03064      ------------------------------------------------------------
ACCPU02128      ------------------------------------------------------------
THIDA01000      ------------------------------------------------------------
METS601221      ------------------------------------------------------------
METGS01186      ------------------------------------------------------------
METFK00972      ------------------------------------------------------------
METML01428      ------------------------------------------------------------
AROAE03899      ------------------------------------------------------------
THASP01686      ------------------------------------------------------------
AZOSB01359      ------------------------------------------------------------
BORA102682      ------------------------------------------------------------
BORPA03415      ------------------------------------------------------------
BORBM03577      ------------------------------------------------------------
BORPE02280      ------------------------------------------------------------
BORPC02060      ------------------------------------------------------------
BORP102392      ------------------------------------------------------------
BORBR03870      ------------------------------------------------------------
BORPD01524      ------------------------------------------------------------
ACHXA01179      ------------------------------------------------------------
RHOFT03114      ------------------------------------------------------------
VEREI01701      ------------------------------------------------------------
VARPE01375      ------------------------------------------------------------
VARPS01266      ------------------------------------------------------------
```

```
DELAS05272      ------------------------------------------------------------
DELSC01235      ------------------------------------------------------------
COMT200883      ------------------------------------------------------------
ACIAC03267      ------------------------------------------------------------
ACIAP03249      ------------------------------------------------------------
ACIET02648      ------------------------------------------------------------
ALIDK03528      ------------------------------------------------------------
RUBGI03408      ------------------------------------------------------------
LEPCP00749      ------------------------------------------------------------
POLSJ03719      ------------------------------------------------------------
POLNA03144      ------------------------------------------------------------
METPP01110      ------------------------------------------------------------
RAMTT03218      ------------------------------------------------------------
RALPJ02457      ------------------------------------------------------------
RALP102102      ------------------------------------------------------------
RALSO02303      ------------------------------------------------------------
RALS801097      ------------------------------------------------------------
HERSS01878      ------------------------------------------------------------
HERAR00955      ------------------------------------------------------------
JANMA01152      ------------------------------------------------------------
THIK102324      ------------------------------------------------------------
POLSQ00682      ------------------------------------------------------------
POLNS00989      ------------------------------------------------------------
BURP800627      ------------------------------------------------------------
BURPP00988      ------------------------------------------------------------
BURSC00728      ------------------------------------------------------------
BURXL00982      ------------------------------------------------------------
BURSG00869      ------------------------------------------------------------
BURRH00629      ------------------------------------------------------------
BURGB00781      ------------------------------------------------------------
BURGS00853      ------------------------------------------------------------
BURPS02618      ------------------------------------------------------------
BURMA01741      ------------------------------------------------------------
BURP103016      ------------------------------------------------------------
BURP002984      ------------------------------------------------------------
BURM701866      ------------------------------------------------------------
BURP602945      ------------------------------------------------------------
BURM902465      ------------------------------------------------------------
BURMS00740      ------------------------------------------------------------
BURTA01515      ------------------------------------------------------------
BURM102386      ------------------------------------------------------------
BURL300890      ------------------------------------------------------------
BURVG00860      ------------------------------------------------------------
BURCM00829      ------------------------------------------------------------
BURA400832      ------------------------------------------------------------
BURCA00489      ------------------------------------------------------------
BURCH00966      ------------------------------------------------------------
BURCC00927      ------------------------------------------------------------
BURCJ02922      ------------------------------------------------------------
EDWI902348      ------------------------------------------------------------
EDWTF01971      ------------------------------------------------------------
EDWTE02176      ------------------------------------------------------------
SODGM01094      ------------------------------------------------------------
MOREP00394      ------------------------------------------------------------
RAHSY01432      ------------------------------------------------------------
RAHAC01419      ------------------------------------------------------------
ERWBE01489      ------------------------------------------------------------
PANAM01339      ------------------------------------------------------------
PANAA00662      ------------------------------------------------------------
PANSA01303      ------------------------------------------------------------
ERWT902154      ------------------------------------------------------------
ERWAC01330      ------------------------------------------------------------
ERWAE01326      ------------------------------------------------------------
ERWPE02241      ------------------------------------------------------------
ERWP602414      ------------------------------------------------------------
```

```
ERWSE02396      ------------------------------------------------------------
PECCP01694      ------------------------------------------------------------
PECWW01889      ------------------------------------------------------------
PECSS01870      ------------------------------------------------------------
PECAS02624      ------------------------------------------------------------
DICDC02220      ------------------------------------------------------------
DICZE02284      ------------------------------------------------------------
DICD302017      ------------------------------------------------------------
DICD502250      ------------------------------------------------------------
XENBS00829      ------------------------------------------------------------
XENNA01480      ------------------------------------------------------------
PHOLL01537      ------------------------------------------------------------
PHOAA02804      ------------------------------------------------------------
SERP501669      ------------------------------------------------------------
SERSA01625      ------------------------------------------------------------
YERPE01271      ------------------------------------------------------------
YERPS01390      ------------------------------------------------------------
YERPA00627      ------------------------------------------------------------
YERPN02489      ------------------------------------------------------------
YERPP02190      ------------------------------------------------------------
YERP302544      ------------------------------------------------------------
YERPB01460      ------------------------------------------------------------
YERPY02633      ------------------------------------------------------------
YERPG01392      ------------------------------------------------------------
YERPD01171      ------------------------------------------------------------
YERP100890      ------------------------------------------------------------
YERPZ01206      ------------------------------------------------------------
YERPH02469      ------------------------------------------------------------
YERE801438      ------------------------------------------------------------
YERE302565      ------------------------------------------------------------
YERE100392      ------------------------------------------------------------
PROMH00687      ------------------------------------------------------------
PROSM03196      ------------------------------------------------------------
TOLAT02279      ------------------------------------------------------------
AERVB02262      ------------------------------------------------------------
AERHH01811      ------------------------------------------------------------
AERS402185      ------------------------------------------------------------
PSYIN02070      ------------------------------------------------------------
SHELP02018      ------------------------------------------------------------
SHEVD02297      ------------------------------------------------------------
SHEPW02341      ------------------------------------------------------------
SHEPA02202      ------------------------------------------------------------
SHEHH02007      ------------------------------------------------------------
SHESH02117      ------------------------------------------------------------
SHEWM02454      ------------------------------------------------------------
SHEAM01758      ------------------------------------------------------------
SHEON02159      ------------------------------------------------------------
SHESM01945      ------------------------------------------------------------
SHESR01991      ------------------------------------------------------------
SHESA02025      ------------------------------------------------------------
SHESW01953      ------------------------------------------------------------
SHEPC01976      ------------------------------------------------------------
SHEP201929      ------------------------------------------------------------
SHEB502016      ------------------------------------------------------------
SHEB802168      ------------------------------------------------------------
SHEB202117      ------------------------------------------------------------
SHEB902270      ------------------------------------------------------------
SHEB602258      ------------------------------------------------------------
SHEDO01680      ------------------------------------------------------------
SHEFN01895      ------------------------------------------------------------
PSEHT01677      ------------------------------------------------------------
ALISL01611      ------------------------------------------------------------
VIBF100893      ------------------------------------------------------------
VIBFM00891      ------------------------------------------------------------
OCESG01399      ------------------------------------------------------------
```

```
VIBA701872      ------------------------------------------------------------
VIBVY01451      ------------------------------------------------------------
VIBVU02545      ------------------------------------------------------------
VIBVM01748      ------------------------------------------------------------
VIBCH01157      ------------------------------------------------------------
VIBCM01114      ------------------------------------------------------------
VIBCJ02064      ------------------------------------------------------------
VIBC300725      ------------------------------------------------------------
FERBD01969      ------------------------------------------------------------
VIBFN02085      ------------------------------------------------------------
VIBTL01089      ------------------------------------------------------------
VIBPA01248      ------------------------------------------------------------
VIBAE02128      ------------------------------------------------------------
VIBCB00599      ------------------------------------------------------------
PANVC00701      ------------------------------------------------------------
SHIBC02443      ------------------------------------------------------------
ENTBF02792      ------------------------------------------------------------
KLEP700892      ------------------------------------------------------------
KLEPH01782      ------------------------------------------------------------
KLEP303551      ------------------------------------------------------------
KLEVT03420      ------------------------------------------------------------
ENTAK02985      ------------------------------------------------------------
KLEOK03151      ------------------------------------------------------------
SALAR01925      ------------------------------------------------------------
SALBC00812      ------------------------------------------------------------
SALPC00926      ------------------------------------------------------------
SALTI00868      ------------------------------------------------------------
SALCH00905      ------------------------------------------------------------
SALPA01706      ------------------------------------------------------------
SALTY00927      ------------------------------------------------------------
SALPK01786      ------------------------------------------------------------
SALHS00978      ------------------------------------------------------------
SALEP00854      ------------------------------------------------------------
SALDC00926      ------------------------------------------------------------
SALA400892      ------------------------------------------------------------
SALG200876      ------------------------------------------------------------
SALTS00888      ------------------------------------------------------------
SALT400905      ------------------------------------------------------------
SALPS01908      ------------------------------------------------------------
SALT101040      ------------------------------------------------------------
SALTD00960      ------------------------------------------------------------
SALPB02472      ------------------------------------------------------------
SALNS00928      ------------------------------------------------------------
SALSV01000      ------------------------------------------------------------
ECOS500812      ------------------------------------------------------------
ECOL600981      ------------------------------------------------------------
ECOL500883      ------------------------------------------------------------
ECOUT00876      ------------------------------------------------------------
ECOK100776      ------------------------------------------------------------
ECOSM02148      ------------------------------------------------------------
ECOLU01057      ------------------------------------------------------------
ECO7I02136      ------------------------------------------------------------
ECO8100828      ------------------------------------------------------------
ECO4500862      ------------------------------------------------------------
ECOAB00879      ------------------------------------------------------------
ECO4400961      ------------------------------------------------------------
ECOUM02550      ------------------------------------------------------------
ECOKI00869      ------------------------------------------------------------
ECOC100920      ------------------------------------------------------------
ECOC200920      ------------------------------------------------------------
ECO8N00803      ------------------------------------------------------------
ECO2700862      ------------------------------------------------------------
ECO2600974      ------------------------------------------------------------
ECOH100942      ------------------------------------------------------------
SHIB301848      ------------------------------------------------------------
```

```
ECOLI00846      ------------------------------------------------------------
ECO5700970      ------------------------------------------------------------
SHISS00756      ------------------------------------------------------------
SHIBS00679      ------------------------------------------------------------
SHIDS01857      ------------------------------------------------------------
ECO2400906      ------------------------------------------------------------
ECODH00784      ------------------------------------------------------------
ECOHS00922      ------------------------------------------------------------
ECOLC02627      ------------------------------------------------------------
ECO5E00946      ------------------------------------------------------------
ECOSE00936      ------------------------------------------------------------
ECO5500894      ------------------------------------------------------------
ECO8A00877      ------------------------------------------------------------
ECOBB00863      ------------------------------------------------------------
ECO5T00943      ------------------------------------------------------------
ECOBW00723      ------------------------------------------------------------
ECO1000919      ------------------------------------------------------------
ECOBD02604      ------------------------------------------------------------
ECOD102659      ------------------------------------------------------------
ECOBR00856      ------------------------------------------------------------
ECOLX02610      ------------------------------------------------------------
ECO1A00939      ------------------------------------------------------------
ECOCB01054      ------------------------------------------------------------
ECOKO02859      ------------------------------------------------------------
ECO1E03320      ------------------------------------------------------------
ECOLW01217      ------------------------------------------------------------
SHIFL01399      ------------------------------------------------------------
SHIF800759      ------------------------------------------------------------
SHIF200805      ------------------------------------------------------------
CITK802118      ------------------------------------------------------------
CITRI00924      ------------------------------------------------------------
CROS802385      ------------------------------------------------------------
CROTZ01500      ------------------------------------------------------------
ENTLS02865      ------------------------------------------------------------
ENT3801400      ------------------------------------------------------------
ENTAL01384      ------------------------------------------------------------
ENTCC02679      ------------------------------------------------------------


STRT101547      ------------------------------------------------------------
STRT201508      ------------------------------------------------------------
STRTD01356      ------------------------------------------------------------
STRTN01533      ------------------------------------------------------------
STRE500345      ------------------------------------------------------------
STRE801625      ------------------------------------------------------------
STREH01636      ------------------------------------------------------------
STREC01656      ------------------------------------------------------------
STREM01495      ------------------------------------------------------------
STRE401629      ------------------------------------------------------------
STRS700387      ------------------------------------------------------------
STRDG01631      ------------------------------------------------------------
STRP301388      ------------------------------------------------------------
STRPZ01205      ------------------------------------------------------------
STRPQ00464      ------------------------------------------------------------
STRPD01453      ------------------------------------------------------------
STRP601399      ------------------------------------------------------------
STRP801366      ------------------------------------------------------------
STRPF01453      ------------------------------------------------------------
STRPG00427      ------------------------------------------------------------
STRA300284      ------------------------------------------------------------
STRA500290      ------------------------------------------------------------
STRA100288      ------------------------------------------------------------
STRA200291      ------------------------------------------------------------
STRIC00348      ------------------------------------------------------------
STRPX00375      ------------------------------------------------------------
```

```
STRMD00432       ------------------------------------------------------------
STRS201734       ------------------------------------------------------------
STRSY01721       ------------------------------------------------------------
STRSX01553       ------------------------------------------------------------
STRSE01478       ------------------------------------------------------------
STREJ01635       ------------------------------------------------------------
STRGZ01544       ------------------------------------------------------------
STRS401593       ------------------------------------------------------------
LACGT00720       ------------------------------------------------------------
LACGL00738       ------------------------------------------------------------
STRSV01774       ------------------------------------------------------------
STRIJ00350       ------------------------------------------------------------
STROU01248       ------------------------------------------------------------
STRM601286       ------------------------------------------------------------
STRES00962       ------------------------------------------------------------
STRP701387       ------------------------------------------------------------
STRZT00756       ------------------------------------------------------------
STRP001079       ------------------------------------------------------------
STRZO01270       ------------------------------------------------------------
STRZ600815       ------------------------------------------------------------
STRET00826       ------------------------------------------------------------
STRPS01429       ------------------------------------------------------------
STRZN01287       ------------------------------------------------------------
STRR601306       ------------------------------------------------------------
STRP201217       ------------------------------------------------------------
STRZP01342       ------------------------------------------------------------
STRZI01226       ------------------------------------------------------------
STRPN01360       ------------------------------------------------------------
STRP401357       ------------------------------------------------------------
STRZJ01282       ------------------------------------------------------------
STRPJ01336       ------------------------------------------------------------
STRPI01436       ------------------------------------------------------------
MARHT00044       ------------------------------------------------------------
THEP300413       ------------------------------------------------------------
THEPX00884       ------------------------------------------------------------
THESX01857       ------------------------------------------------------------
THEM301788       ------------------------------------------------------------
THEIA01812       ------------------------------------------------------------
THETC00443       ------------------------------------------------------------
THESW01026       ------------------------------------------------------------
THEXL00360       ------------------------------------------------------------
THEID01127       ------------------------------------------------------------
THEOJ00147       ------------------------------------------------------------
DESAS01237       ------------------------------------------------------------
DESK701373       ------------------------------------------------------------
KYRT200729       ------------------------------------------------------------
STACT00410       ------------------------------------------------------------
STAS101952       ------------------------------------------------------------
STALH01969       ------------------------------------------------------------
STAEQ00422       ------------------------------------------------------------
STAES00543       ------------------------------------------------------------
STAAB00714       ------------------------------------------------------------
STAA500765       ------------------------------------------------------------
STAAM00757       ------------------------------------------------------------
STAAW00726       ------------------------------------------------------------
STAAS00733       ------------------------------------------------------------
STAAN00727       ------------------------------------------------------------
STAAC00807       ------------------------------------------------------------
STAA300727       ------------------------------------------------------------
STAA800734       ------------------------------------------------------------
STAA100752       ------------------------------------------------------------
STAA200774       ------------------------------------------------------------
STAA900759       ------------------------------------------------------------
STAAE00716       ------------------------------------------------------------
STAAT00767       ------------------------------------------------------------
```

```
STAAD00695      ------------------------------------------------------------
STAA000815      ------------------------------------------------------------
STAAH02407      ------------------------------------------------------------
STAAF00763      ------------------------------------------------------------
STAAK00744      ------------------------------------------------------------
STAAJ00703      ------------------------------------------------------------
STAAG00690      ------------------------------------------------------------
STAA400732      ------------------------------------------------------------
STAAR00788      ------------------------------------------------------------
LISSS02378      ------------------------------------------------------------
LISIN02590      ------------------------------------------------------------
LISW602421      ------------------------------------------------------------
EXISA00870      ------------------------------------------------------------
EXIS202358      ------------------------------------------------------------
EXIAB02180      ------------------------------------------------------------
OCEIH02462      ------------------------------------------------------------
BACIE01058      ------------------------------------------------------------
BACCJ03468      ------------------------------------------------------------
BACHD03507      ------------------------------------------------------------
BACPE03382      ------------------------------------------------------------
SOLSS00743      ------------------------------------------------------------
BACC600715      ------------------------------------------------------------
ANOFW02486      ------------------------------------------------------------
GEOKA03042      ------------------------------------------------------------
GEOSY02976      ------------------------------------------------------------
GEOTN02952      ------------------------------------------------------------
GEOSW02588      ------------------------------------------------------------
GEOS000381      ------------------------------------------------------------
GEOTC00378      ------------------------------------------------------------
BACMD04906      ------------------------------------------------------------
BACMQ04908      ------------------------------------------------------------
BACWK04830      ------------------------------------------------------------
BACAN04889      ------------------------------------------------------------
BACC105210      ------------------------------------------------------------
BACC705051      ------------------------------------------------------------
BACC005112      ------------------------------------------------------------
BACC305043      ------------------------------------------------------------
BACAC05232      ------------------------------------------------------------
BACAA04720      ------------------------------------------------------------
BACT005085      ------------------------------------------------------------
BACC205124      ------------------------------------------------------------
BACC405064      ------------------------------------------------------------
BACT104712      ------------------------------------------------------------
BACLD03769      ------------------------------------------------------------
BACPZ03376      ------------------------------------------------------------
BACSU03600      ------------------------------------------------------------
BACST01653      ------------------------------------------------------------
BACPT03541      ------------------------------------------------------------
LEUGG00661      ------------------------------------------------------------
LEUGJ00629      ------------------------------------------------------------
LEUCJ00485      ------------------------------------------------------------
LACAR00657      ------------------------------------------------------------
LACA300647      ------------------------------------------------------------
LACAL00643      ------------------------------------------------------------
LACKZ00964      ------------------------------------------------------------
LACRJ00357      ------------------------------------------------------------
LACRD00361      ------------------------------------------------------------
LACRS01501      ------------------------------------------------------------
LACSM00455      ------------------------------------------------------------
LACRG00878      ------------------------------------------------------------
LACRL00939      ------------------------------------------------------------
LACC300844      ------------------------------------------------------------
LACCZ00824      ------------------------------------------------------------
LACCB00988      ------------------------------------------------------------
LACCD01016      ------------------------------------------------------------
```

```
LACCC01014      ------------------------------------------------------------
LACBN01277      ------------------------------------------------------------
LACBA00605      ------------------------------------------------------------
LACPL00650      ------------------------------------------------------------
LACPJ00628      ------------------------------------------------------------
LACPS00585      ------------------------------------------------------------
PEDCP00497      ------------------------------------------------------------
CARS100359      ------------------------------------------------------------
AERUA00263      ------------------------------------------------------------
ELUMP00590      ------------------------------------------------------------
SPHPG02940      ------------------------------------------------------------
SPHGB01868      ------------------------------------------------------------
TREPZ00273      ------------------------------------------------------------
TREAZ03414      ------------------------------------------------------------
SPITD00734      ------------------------------------------------------------
SPITZ01373      ------------------------------------------------------------
TREPA00803      ------------------------------------------------------------
TREPS00802      ------------------------------------------------------------
TREPC00747      ------------------------------------------------------------
TREPM00823      ------------------------------------------------------------
TREPD00824      ------------------------------------------------------------
TREPU00781      ------------------------------------------------------------
ENCCU00216      ------------------------------------------------------------
HELM100964      ------------------------------------------------------------
HELCP01490      ------------------------------------------------------------
ARCFU01526      ------------------------------------------------------------
FERPA02445      ------------------------------------------------------------
ARCVS01910      ------------------------------------------------------------
METEZ00677      ------------------------------------------------------------
METHD00869      ------------------------------------------------------------
METMA02304      ------------------------------------------------------------
METAC01311      ------------------------------------------------------------
KOSOT00298      ------------------------------------------------------------
MARPK01613      ------------------------------------------------------------
SLAHD02439      ------------------------------------------------------------
FILAD00976      ------------------------------------------------------------
BUTPB02463      ------------------------------------------------------------
CLOPH00250      ------------------------------------------------------------
CLOSW00592      ------------------------------------------------------------
LACFC00207      ------------------------------------------------------------
CRYCD00853      ------------------------------------------------------------
EGGLE01847      ------------------------------------------------------------
PYRFU01410      ------------------------------------------------------------
PYRHO01476      ------------------------------------------------------------
PYRAB00730      ------------------------------------------------------------
PYRSN00014      ------------------------------------------------------------
THEGJ00181      ------------------------------------------------------------
THEKO02097      ------------------------------------------------------------
THEON01610      ------------------------------------------------------------
THES401476      ------------------------------------------------------------
SYNWW02368      ------------------------------------------------------------
UNCTG00012      ------------------------------------------------------------
THEA101460      ------------------------------------------------------------
DENA201647      ------------------------------------------------------------
DEFDS00488      ------------------------------------------------------------
CALNY01292      ------------------------------------------------------------
SYNGF01652      ------------------------------------------------------------
DESB201123      ------------------------------------------------------------
DEIPM00845      ------------------------------------------------------------
DEIRA01924      ------------------------------------------------------------
DEIML01437      ------------------------------------------------------------
DEIGD02532      ------------------------------------------------------------
DEIDV00622      ------------------------------------------------------------
TRURR01079      ------------------------------------------------------------
THETG01863      ------------------------------------------------------------
```

```
THET201543      ------------------------------------------------------------
THET801911      ------------------------------------------------------------
OCEP502115      ------------------------------------------------------------
DEHLB00585      ------------------------------------------------------------
DEHMG00439      ------------------------------------------------------------
DEHMB00497      ------------------------------------------------------------
DEHMC00427      ------------------------------------------------------------
THELD00726      EELTSH-VK--DLALIDGYKNARMVEKLGVKSMPVLLELSKGNILRSEKI-----ESLED
ANAMD00624      EK---TYHR--KVILVDGYKNKRMCEKLGIDELPKAVVLNKGTLVRSKKI-----LGVQD
THEAS00815      EEWASSKNV--QVTLVDGYRNLRMMEKLGVRELPTAMVLKEGREVSRASV-----TSEED
AMICL00707      EEMSKETGE--KVAIIDGYRNARMVEKLELGEMPVVVELKKGVVASKKTV-----ATASE
CLOCE01684      ------------------------------------------------------------
HYDS000616      ------------------------------------------------------------
HYDTT00224      ------------------------------------------------------------
PELTS01405      ------------------------------------------------------------
DESRL02179      ------------------------------------------------------------
BORBP00506      ------------------------------------------------------------
BORAP00518      ------------------------------------------------------------
BORBU00514      ------------------------------------------------------------
BORBZ00490      ------------------------------------------------------------
BORBN00493      ------------------------------------------------------------
BORRA00486      ------------------------------------------------------------
BORDL00498      ------------------------------------------------------------
BORHD00497      ------------------------------------------------------------
BORT900497      ------------------------------------------------------------
METKA01560      ------------------------------------------------------------
MYCA500361      ------------------------------------------------------------
METVS00145      ------------------------------------------------------------
METOI01431      ------------------------------------------------------------
MYCHN00051      ------------------------------------------------------------
MYCSL00465      ------------------------------------------------------------
MYCS300420      ------------------------------------------------------------
DESK101258      ------------------------------------------------------------
STAHD01472      ------------------------------------------------------------
THEC100270      ------------------------------------------------------------
NANEQ00478      ------------------------------------------------------------
CALLD01225      ------------------------------------------------------------
SULSO02155      ------------------------------------------------------------
SULS900210      ------------------------------------------------------------
SULIA00208      ------------------------------------------------------------
SULIM00207      ------------------------------------------------------------
SULIK00225      ------------------------------------------------------------
IGNH400907      ------------------------------------------------------------
KORCO01040      ------------------------------------------------------------
THESM01133      ------------------------------------------------------------
THEBM01534      ------------------------------------------------------------
METST01363      ------------------------------------------------------------
METTH00703      ------------------------------------------------------------
METSL02406      ------------------------------------------------------------
METLA02406      ------------------------------------------------------------
METPW00194      ------------------------------------------------------------
RUBXD00226      ------------------------------------------------------------
RHOM400178      ------------------------------------------------------------
GARV400051      ------------------------------------------------------------
BIFAP01644      ------------------------------------------------------------
SEGRD00014      ------------------------------------------------------------
GORB404535      ------------------------------------------------------------
GORPV04920      ------------------------------------------------------------
MYCA904903      ------------------------------------------------------------
MYCSS05356      ------------------------------------------------------------
MYCSJ05684      ------------------------------------------------------------
MYCSK05387      ------------------------------------------------------------
MYCS206581      ------------------------------------------------------------
MYCCN05158      ------------------------------------------------------------
MYCVP05890      ------------------------------------------------------------
```

```
MYCGI00775      ------------------------------------------------------------
MYCSR04994      ------------------------------------------------------------
AMYSD04545      ------------------------------------------------------------
MYCLE02681      EEIASEQRNQLTVAKLDVDTNPEMAREFQVVSIPTMILFQGGQPVKRIVGAKGKAALLRD
MYCLB02681      EEIASEQRNQLTVAKLDVDTNPEMAREFQVVSIPTMILFQGGQPVKRIVGAKGKAALLRD
MYCSD04323      ------------------------------------------------------------
MYCPA04306      ------------------------------------------------------------
MYCA105023      ------------------------------------------------------------
MYCUA04107      ------------------------------------------------------------
MYCMM05378      ------------------------------------------------------------
MYCA003905      ------------------------------------------------------------
MYCTU03943      ------------------------------------------------------------
MYCTF03846      ------------------------------------------------------------
MYCTA03980      ------------------------------------------------------------
MYCTK04010      ------------------------------------------------------------
MYCTC03612      ------------------------------------------------------------
MYCTD03548      ------------------------------------------------------------
MYCCP03898      ------------------------------------------------------------
MYCBO02863      ------------------------------------------------------------
MYCBP03913      ------------------------------------------------------------
MYCBT03913      ------------------------------------------------------------
TSUPD04078      ------------------------------------------------------------
ACTMD06897      ------------------------------------------------------------
SACES08402      ------------------------------------------------------------
SACVD03814      ------------------------------------------------------------
AMYMU09190      ------------------------------------------------------------
AMYMS10178      ------------------------------------------------------------
PSEUX06421      ------------------------------------------------------------
NOCFA05681      ------------------------------------------------------------
NOCCG05476      ------------------------------------------------------------
RHOE406010      ------------------------------------------------------------
RHOOB03468      ------------------------------------------------------------
RHOE104515      ------------------------------------------------------------
CORDI02302      ------------------------------------------------------------
CORD202219      ------------------------------------------------------------
CORDL02208      ------------------------------------------------------------
CORDJ02208      ------------------------------------------------------------
CORDH02221      ------------------------------------------------------------
CORD702316      ------------------------------------------------------------
CORD302337      ------------------------------------------------------------
CORDD02235      ------------------------------------------------------------
CORDV02170      ------------------------------------------------------------
CORDW02254      ------------------------------------------------------------
CORDK02230      ------------------------------------------------------------
COREF02870      ------------------------------------------------------------
CORGL03082      ------------------------------------------------------------
CORGK02974      ------------------------------------------------------------
CORGB03038      ------------------------------------------------------------
CORK402006      ------------------------------------------------------------
CORJK02028      ------------------------------------------------------------
CORVD02951      ------------------------------------------------------------
ARCHD01706      ------------------------------------------------------------
THET101205      ------------------------------------------------------------
PROAC02247      ------------------------------------------------------------
PROAS02302      ------------------------------------------------------------
CAERE29798      ------------------------------------------------------------
CELFA03749      ------------------------------------------------------------
JONDD02484      ------------------------------------------------------------
XYLCX03324      ------------------------------------------------------------
ACIC102144      ------------------------------------------------------------
FRADG04042      ------------------------------------------------------------
FRASU07065      ------------------------------------------------------------
FRASN07115      ------------------------------------------------------------
FRASC04424      ------------------------------------------------------------
FRAAA06712      ------------------------------------------------------------
```

KYTSD02521 --
KINRD04462 --
NOCDD04789 --
NOCAA01941 --
STRRD08913 --
THECD04863 --
THEBD03528 --
CATAD08894 --
KRIFD06917 --
KITSK03730 --
STRBB05366 --
STRVP03660 --
STRSW04381 --
STRGG03562 --
STRFA03098 --
STRAW04303 --
STRCO02929 --
STRHJ05105 --
MONBE04991 --
CHLRE01313 --
MEDTR25591 --
SOLLC13750 --
PRUPE10733 --
MANES18605 --
THECC00884 --
PHYPA31147 --
AMBTC19471 --
MUSAC26038 --
MUSAM33177 --
SETIT03079 --
ORYBR12195 --
COCLU07729 --
PHANO13702 --
PHAND10804 --
AURPU02089 --
ZYMTR07711 --
DICPU05926 --
ENTHI00522 --
LEPBA02231 --
LEPBP02301 --
LEPBL01462 --
LEPBJ01276 --
LEPIN02475 --
LEPII01996 --
LEPIC01426 --
SPIAZ00697 --
PENRW10140 --
PENCH09104 --
EURHE07269 --
ASPAC07301 --
EMENI10387 --
EMEND02596 --
ASPTN06742 --
ASPCL04014 --
ASPFU05647 --
NEOFI00452 --
CRYPA10563 --
BLUGR03498 --
SCLS112814 --
MAGGR04266 --
NEUCR01575 --
NEUT908941 --
VERDA02342 --
COLSU12486 --
HYPAI01684 --

HYPVG06080 --
HYPJE05895 --
NECHA05020 --
FUSO415847 --
GIBZA01026 --
SCHPO04025 --
YARLI03635 --
ASHGO00946 --
KLULA02190 --
ZYGRO00676 --
DEKBR01813 --
PICPG04776 --
CANTE00916 --
LODEL03891 --
DEBHA05546 --
SPAPN03477 --
CANAW04800 --
PICST04701 --
PUCGT10887 --
PUCGR11813 --
PHYBL11006 --
USTMA03757 --
USTHO04132 --
WALSE04527 --
TREME07701 --
AURST04751 --
FOMME10177 --
CONPW06392 --
STEHR07076 --
HETAN06295 --
GLOTR06982 --
PUNST01981 --
LACBI02877 --
COPCI16429 --
DICSQ11618 --
TRAVS13180 --
WOLCO03584 --
FOMPI05979 --
PHLGI10219 --
PHACH05757 --
RICTY00419 --
RICPR00429 --
RICPP00461 --
RICBR00434 --
RICB800988 --
RICCK00600 --
RICAH00614 --
RICAC00782 --
RICFE00656 --
RICMS00042 --
RICM500453 --
RICR300665 --
RICAG00651 --
RICP300630 --
RICRS00632 --
RICRO00663 --
RICCN00618 --
RICPT00620 --
RICAE00497 --
RICJY00467 --
RICPU00072 --
RICS100537 --
BARBK00891 --
BARVW00932 --
BART100617 --

```
BARGA00466      ------------------------------------------------------------
BARHE01107      ------------------------------------------------------------
BARQU00895      ------------------------------------------------------------
OCHA401642      ------------------------------------------------------------
BRUAB01390      ------------------------------------------------------------
BRUA201507      ------------------------------------------------------------
BRUA101339      ------------------------------------------------------------
BRUSU01458      ------------------------------------------------------------
BRUME00510      ------------------------------------------------------------
BRUSI01461      ------------------------------------------------------------
BRUC201446      ------------------------------------------------------------
BRUMC01440      ------------------------------------------------------------
BRUMB01421      ------------------------------------------------------------
BRUM501487      ------------------------------------------------------------
BRUO201285      ------------------------------------------------------------
RHILO01973      ------------------------------------------------------------
CHESB02097      ------------------------------------------------------------
METPB00996      ------------------------------------------------------------
METEP01063      ------------------------------------------------------------
METEA00810      ------------------------------------------------------------
METED01453      ------------------------------------------------------------
METS403554      ------------------------------------------------------------
METNO05482      ------------------------------------------------------------
METSZ03234      ------------------------------------------------------------
BEII900056      ------------------------------------------------------------
METSB02743      ------------------------------------------------------------
MAGMM00401      ------------------------------------------------------------
HYPNA00542      ------------------------------------------------------------
KETVY00902      ------------------------------------------------------------
KETVW00472      ------------------------------------------------------------
ROSDO03242      ------------------------------------------------------------
ROSLO02615      ------------------------------------------------------------
RUEPO00888      ------------------------------------------------------------
RUEST00613      ------------------------------------------------------------
PHAIB02390      ------------------------------------------------------------
PARDP02130      ------------------------------------------------------------
DINSH02620      ------------------------------------------------------------
RHOCB02768      ------------------------------------------------------------
RHOS500248      ------------------------------------------------------------
RHOS400150      ------------------------------------------------------------
RHOS100227      ------------------------------------------------------------
RHOSK02952      ------------------------------------------------------------
MIDMI00790      ------------------------------------------------------------
ACEP301595      ------------------------------------------------------------
MICAA01566      ------------------------------------------------------------
TISMK03676      ------------------------------------------------------------
AZOL402409      ------------------------------------------------------------
PSEUV04348      ------------------------------------------------------------
HIRBI01095      ------------------------------------------------------------
PARL102241      ------------------------------------------------------------
MARMM02139      ------------------------------------------------------------
PHEZH02588      ------------------------------------------------------------
CAUCR02826      ------------------------------------------------------------
CAUCN02939      ------------------------------------------------------------
CAUST00891      ------------------------------------------------------------
PARBH01522      ------------------------------------------------------------
PELHB02084      ------------------------------------------------------------
HYPDA03273      ------------------------------------------------------------
HYPSM04642      ------------------------------------------------------------
OLICO01107      ------------------------------------------------------------
OLICM02790      ------------------------------------------------------------
RHOPS01446      ------------------------------------------------------------
RHOPA04062      ------------------------------------------------------------
RHOPT04533      ------------------------------------------------------------
RHOPX04235      ------------------------------------------------------------
```

```
BRADU07321      ------------------------------------------------------------
BRASO01239      ------------------------------------------------------------
BRASB06352      ------------------------------------------------------------
RHOPB03773      ------------------------------------------------------------
NITWN02324      ------------------------------------------------------------
NITHX02707      ------------------------------------------------------------
AZOC501143      ------------------------------------------------------------
XANP202679      ------------------------------------------------------------
CHLTF01983      ------------------------------------------------------------
IGNAJ02236      ------------------------------------------------------------
MELRP00403      ------------------------------------------------------------
ANADF00468      ------------------------------------------------------------
CHLCH01125      ------------------------------------------------------------
PELPB01419      ------------------------------------------------------------
CHLL200781      ------------------------------------------------------------
CHLTE00830      ------------------------------------------------------------
CHLP800854      ------------------------------------------------------------
CHLL701225      ------------------------------------------------------------
CHLPM00993      ------------------------------------------------------------
WOLTR00562      ------------------------------------------------------------
WOLPP00135      ------------------------------------------------------------
WOLPM00650      ------------------------------------------------------------
WOLWR00416      ------------------------------------------------------------
EHRCR00696      ------------------------------------------------------------
ANAMM00345      ------------------------------------------------------------
ANAMF00341      ------------------------------------------------------------
ANAPZ00610      ------------------------------------------------------------
NEOSM00537      ------------------------------------------------------------
NEORI00513      ------------------------------------------------------------
PELUB00076      ------------------------------------------------------------
PELSM00736      ------------------------------------------------------------
PUNMI01409      ------------------------------------------------------------
ASTEC00651      ------------------------------------------------------------
ZYMMT00154      ------------------------------------------------------------
ZYMMO00984      ------------------------------------------------------------
ZYMMA00176      ------------------------------------------------------------
ZYMMN00183      ------------------------------------------------------------
SPHAL00143      ------------------------------------------------------------
SPHWW03978      ------------------------------------------------------------
SPHJU02313      ------------------------------------------------------------
NOVAD02319      ------------------------------------------------------------
ERYLH00934      ------------------------------------------------------------
GRABC00720      ------------------------------------------------------------
GLUDA03075      ------------------------------------------------------------
KOMMN00774      ------------------------------------------------------------
HALVD01097      ------------------------------------------------------------
HALHT01699      ------------------------------------------------------------
METI401038      ------------------------------------------------------------
PLAL201612      ------------------------------------------------------------
GEMAT01983      ------------------------------------------------------------
CYAAP02456      ------------------------------------------------------------
CHLPN00303      ------------------------------------------------------------
CHLPP00439      ------------------------------------------------------------
CHLPE00727      ------------------------------------------------------------
CHLTR00101      ------------------------------------------------------------
CHLTA00102      ------------------------------------------------------------
CHLTJ00101      ------------------------------------------------------------
CHLTD00098      ------------------------------------------------------------
CHLT700102      ------------------------------------------------------------
CHLT000101      ------------------------------------------------------------
CHLT500104      ------------------------------------------------------------
CHLTL00098      ------------------------------------------------------------
CHLTG00102      ------------------------------------------------------------
CHLTS00101      ------------------------------------------------------------
CHLT900102      ------------------------------------------------------------
```

CHLTZ00101 --
CHLT400432 --
CHLT100103 --
CHLT200350 --
CHLTB00350 --
CHLTC00378 --
SIMNZ00831 --
PARUW01706 --
WADCW00943 --
SINAD04980 --
BIFLB01558 --
BIFAB00498 --
BIFAV01561 --
BIFAS01557 --
BIFA001518 --
BIFBA01814 --
BIFAA01619 --
BIFDB02112 --
TERSS03091 --
GRATM03160 --
GRAMM03836 --
AKKM801247 --
OPITP04130 --
CORAD01794 --
BUCCC00180 --
CENSY00347 --
NITMS00668 --
MEIRD02823 --
HERA203780 --
CHLAA02018 --
CHLSY02175 --
CHLAD02599 --
CALAS01745 --
ANATU01696 --
SULMS00235 --
BLASB00041 --
BLASP00551 --
AZOPC00437 --
LEPBD01878 --
SALRD02577 --
SALRM02866 --
RIEPU00166 --
ORITB00015 --
ORITI01234 --
PREMB01541 --
PREDF01155 --
PREI702011 --
ALIFI01035 --
ODOSD00362 --
PRER201250 --
PALPW00206 --
PORGI00953 --
PORG301168 --
BACV803426 --
BACT601486 --
BACTN04290 --
BACFR01032 --
BACFN00913 --
BACF600963 --
OWEHD03413 --
PSYTT00390 --
NONDD00011 --
ROBBH02711 --
CELAD02592 --
CELLC02474 --

```
MARSH03072      ------------------------------------------------------------
MURRD00159      ------------------------------------------------------------
ZOBGA00152      ------------------------------------------------------------
GRAFK00949      ------------------------------------------------------------
ZUNPS02344      ------------------------------------------------------------
AEQSU02079      ------------------------------------------------------------
HALH105745      ------------------------------------------------------------
SAPGL02893      ------------------------------------------------------------
AMOA500014      ------------------------------------------------------------
FLAIG01802      ------------------------------------------------------------
FLACA02326      ------------------------------------------------------------
FLAJ100198      ------------------------------------------------------------
FLABF01528      ------------------------------------------------------------
CAPOD01333      ------------------------------------------------------------
CAPCC00414      ------------------------------------------------------------
FLELS01898      ------------------------------------------------------------
SOLCM01166      ------------------------------------------------------------
PEDHD01598      ------------------------------------------------------------
SPHS203207      ------------------------------------------------------------
LEAB401188      ------------------------------------------------------------
EMTOG00092      ------------------------------------------------------------
SPILD01737      ------------------------------------------------------------
DYAFD00084      ------------------------------------------------------------
CYCMS03063      ------------------------------------------------------------
ECHVK03326      ------------------------------------------------------------
BELBD02542      ------------------------------------------------------------
NITGG02112      ------------------------------------------------------------
THEM700111      ------------------------------------------------------------
CREAS01350      ------------------------------------------------------------
TURPD02772      ------------------------------------------------------------
SORC507603      ------------------------------------------------------------
BDEBA00337      ------------------------------------------------------------
STIAD02752      ------------------------------------------------------------
MYXXD01876      ------------------------------------------------------------
MYXFH03454      ------------------------------------------------------------
CORCM01960      ------------------------------------------------------------
MYXSD02209      ------------------------------------------------------------
LEPFC02126      ------------------------------------------------------------
LEPFM02294      ------------------------------------------------------------
SOLUE00582      ------------------------------------------------------------
KORVE01333      ------------------------------------------------------------
ACIC502553      ------------------------------------------------------------
ACIFD00030      ------------------------------------------------------------
HALMS01402      ------------------------------------------------------------
CHLPD01133      ------------------------------------------------------------
CHLPB01137      ------------------------------------------------------------
PROA200897      ------------------------------------------------------------
WIGBR00492      ------------------------------------------------------------
BUCA500289      ------------------------------------------------------------
BUCAI00292      ------------------------------------------------------------
BUCAF00306      ------------------------------------------------------------
BUCAT00287      ------------------------------------------------------------
BUCA000302      ------------------------------------------------------------
BAUCH00290      ------------------------------------------------------------
BLOVB00369      ------------------------------------------------------------
BLOFL00367      ------------------------------------------------------------
BLOPB00375      ------------------------------------------------------------
BUCAP00289      ------------------------------------------------------------
COXBU01001      ------------------------------------------------------------
COXBN01188      ------------------------------------------------------------
COXBR01117      ------------------------------------------------------------
COXB200717      ------------------------------------------------------------
COXB100914      ------------------------------------------------------------
FRAP200241      ------------------------------------------------------------
FRANT01012      ------------------------------------------------------------
```

```
FRACN00522      ------------------------------------------------------------
FRATT00465      ------------------------------------------------------------
FRAT100465      ------------------------------------------------------------
FRATE00462      ------------------------------------------------------------
FRATW01216      ------------------------------------------------------------
FRATM01110      ------------------------------------------------------------
FRACF00566      ------------------------------------------------------------
FRATO01212      ------------------------------------------------------------
FRATH01486      ------------------------------------------------------------
FRATF01216      ------------------------------------------------------------
FRATN00564      ------------------------------------------------------------
ACIF500512      ------------------------------------------------------------
ACIF200362      ------------------------------------------------------------
DECAR01284      ------------------------------------------------------------
NEIG100525      ------------------------------------------------------------
NEIG201314      ------------------------------------------------------------
NEIM800926      ------------------------------------------------------------
NEIMP01199      ------------------------------------------------------------
NEIMB01212      ------------------------------------------------------------
NEIMF01143      ------------------------------------------------------------
NEIML01119      ------------------------------------------------------------
NEIMM00780      ------------------------------------------------------------
NEIMH00827      ------------------------------------------------------------
NEIMG01168      ------------------------------------------------------------
NEIMN01254      ------------------------------------------------------------
NEIMO00810      ------------------------------------------------------------
NEIM701229      ------------------------------------------------------------
NEIMA01334      ------------------------------------------------------------
NEIMW01125      ------------------------------------------------------------
DICNV01037      ------------------------------------------------------------
VESOH00047      ------------------------------------------------------------
RUTMC00043      ------------------------------------------------------------
HALHL02255      ------------------------------------------------------------
PELPD03177      ------------------------------------------------------------
GEOS804030      ------------------------------------------------------------
GEOBB03636      ------------------------------------------------------------
GEOSM03700      ------------------------------------------------------------
HALNC00046      ------------------------------------------------------------
HAEPS00887      ------------------------------------------------------------
GALAU02285      ------------------------------------------------------------
HISS201000      ------------------------------------------------------------
HAES101151      ------------------------------------------------------------
PASMU00573      ------------------------------------------------------------
PASMH00579      ------------------------------------------------------------
ACTSZ01560      ------------------------------------------------------------
AGGAN00985      ------------------------------------------------------------
NITHN03181      ------------------------------------------------------------
NITOC00311      ------------------------------------------------------------
NITWC02498      ------------------------------------------------------------
METNJ00648      ------------------------------------------------------------
METFJ01616      ------------------------------------------------------------
ALKEH00244      ------------------------------------------------------------
MARMS03166      ------------------------------------------------------------
MARM102688      ------------------------------------------------------------
THICR00763      ------------------------------------------------------------
THICA00974      ------------------------------------------------------------
THIV600851      ------------------------------------------------------------
THISH01973      ------------------------------------------------------------
METAA00813      ------------------------------------------------------------
META200813      ------------------------------------------------------------
METMM02895      ------------------------------------------------------------
FRAAD00813      ------------------------------------------------------------
XYLFA01416      ------------------------------------------------------------
XYLFT00623      ------------------------------------------------------------
XYLF200667      ------------------------------------------------------------
```

```
XYLFG01622      ------------------------------------------------------------
XYLFM00690      ------------------------------------------------------------
PSEUP01680      ------------------------------------------------------------
STRM501972      ------------------------------------------------------------
PSEUU01524      ------------------------------------------------------------
XANAP01407      ------------------------------------------------------------
XANCP01918      ------------------------------------------------------------
XANC802154      ------------------------------------------------------------
XANCB02263      ------------------------------------------------------------
XANOR02379      ------------------------------------------------------------
XANOM02295      ------------------------------------------------------------
XANOP02318      ------------------------------------------------------------
XANAC01951      ------------------------------------------------------------
CYCSP01155      ------------------------------------------------------------
GEOLS03275      ------------------------------------------------------------
GEOUR03858      ------------------------------------------------------------
GEODF00692      ------------------------------------------------------------
GEOSL00482      ------------------------------------------------------------
GEOSK00469      ------------------------------------------------------------
GEOMG02998      ------------------------------------------------------------
MORCR00228      ------------------------------------------------------------
ACIAD00798      ------------------------------------------------------------
ACIBC00821      ------------------------------------------------------------
ACIBY02725      ------------------------------------------------------------
ACIB302691      ------------------------------------------------------------
ACIB500882      ------------------------------------------------------------
ACIB100850      ------------------------------------------------------------
ACIBD00843      ------------------------------------------------------------
ACIBS02229      ------------------------------------------------------------
ACICP00137      ------------------------------------------------------------
ACISD03052      ------------------------------------------------------------
LEGLN02402      ------------------------------------------------------------
LEGPA01726      ------------------------------------------------------------
LEGPH00846      ------------------------------------------------------------
LEGPC01171      ------------------------------------------------------------
LEGP201857      ------------------------------------------------------------
LEGPL01727      ------------------------------------------------------------
COLP302668      ------------------------------------------------------------
KANKD01106      ------------------------------------------------------------
IDILO00659      ------------------------------------------------------------
PSEU901284      ------------------------------------------------------------
PSEA602346      ------------------------------------------------------------
ALTSS01871      ------------------------------------------------------------
ALTMD01611      ------------------------------------------------------------
ALTME01705      ------------------------------------------------------------
ALTMB01797      ------------------------------------------------------------
ALTMS01652      ------------------------------------------------------------
SACD201683      ------------------------------------------------------------
TERTT01748      ------------------------------------------------------------
SIMAS00609      ------------------------------------------------------------
ALCDB01962      ------------------------------------------------------------
CHRSD02939      ------------------------------------------------------------
HALED02811      ------------------------------------------------------------
SIDLE02655      ------------------------------------------------------------
GALCS01621      ------------------------------------------------------------
LARHH01446      ------------------------------------------------------------
CHRVO02807      ------------------------------------------------------------
PSEUL01648      ------------------------------------------------------------
NITEU01859      ------------------------------------------------------------
NITEC00745      ------------------------------------------------------------
NITMU00023      ------------------------------------------------------------
NITSI03064      ------------------------------------------------------------
ACCPU02128      ------------------------------------------------------------
THIDA01000      ------------------------------------------------------------
METS601221      ------------------------------------------------------------
```

```
METGS01186      ------------------------------------------------------------
METFK00972      ------------------------------------------------------------
METML01428      ------------------------------------------------------------
AROAE03899      ------------------------------------------------------------
THASP01686      ------------------------------------------------------------
AZOSB01359      ------------------------------------------------------------
BORA102682      ------------------------------------------------------------
BORPA03415      ------------------------------------------------------------
BORBM03577      ------------------------------------------------------------
BORPE02280      ------------------------------------------------------------
BORPC02060      ------------------------------------------------------------
BORP102392      ------------------------------------------------------------
BORBR03870      ------------------------------------------------------------
BORPD01524      ------------------------------------------------------------
ACHXA01179      ------------------------------------------------------------
RHOFT03114      ------------------------------------------------------------
VEREI01701      ------------------------------------------------------------
VARPE01375      ------------------------------------------------------------
VARPS01266      ------------------------------------------------------------
DELAS05272      ------------------------------------------------------------
DELSC01235      ------------------------------------------------------------
COMT200883      ------------------------------------------------------------
ACIAC03267      ------------------------------------------------------------
ACIAP03249      ------------------------------------------------------------
ACIET02648      ------------------------------------------------------------
ALIDK03528      ------------------------------------------------------------
RUBGI03408      ------------------------------------------------------------
LEPCP00749      ------------------------------------------------------------
POLSJ03719      ------------------------------------------------------------
POLNA03144      ------------------------------------------------------------
METPP01110      ------------------------------------------------------------
RAMTT03218      ------------------------------------------------------------
RALPJ02457      ------------------------------------------------------------
RALP102102      ------------------------------------------------------------
RALSO02303      ------------------------------------------------------------
RALS801097      ------------------------------------------------------------
HERSS01878      ------------------------------------------------------------
HERAR00955      ------------------------------------------------------------
JANMA01152      ------------------------------------------------------------
THIK102324      ------------------------------------------------------------
POLSQ00682      ------------------------------------------------------------
POLNS00989      ------------------------------------------------------------
BURP800627      ------------------------------------------------------------
BURPP00988      ------------------------------------------------------------
BURSC00728      ------------------------------------------------------------
BURXL00982      ------------------------------------------------------------
BURSG00869      ------------------------------------------------------------
BURRH00629      ------------------------------------------------------------
BURGB00781      ------------------------------------------------------------
BURGS00853      ------------------------------------------------------------
BURPS02618      ------------------------------------------------------------
BURMA01741      ------------------------------------------------------------
BURP103016      ------------------------------------------------------------
BURP002984      ------------------------------------------------------------
BURM701866      ------------------------------------------------------------
BURP602945      ------------------------------------------------------------
BURM902465      ------------------------------------------------------------
BURMS00740      ------------------------------------------------------------
BURTA01515      ------------------------------------------------------------
BURM102386      ------------------------------------------------------------
BURL300890      ------------------------------------------------------------
BURVG00860      ------------------------------------------------------------
BURCM00829      ------------------------------------------------------------
BURA400832      ------------------------------------------------------------
BURCA00489      ------------------------------------------------------------
```

BURCH00966 --
BURCC00927 --
BURCJ02922 --
EDWI902348 --
EDWTF01971 --
EDWTE02176 --
SODGM01094 --
MOREP00394 --
RAHSY01432 --
RAHAC01419 --
ERWBE01489 --
PANAM01339 --
PANAA00662 --
PANSA01303 --
ERWT902154 --
ERWAC01330 --
ERWAE01326 --
ERWPE02241 --
ERWP602414 --
ERWSE02396 --
PECCP01694 --
PECWW01889 --
PECSS01870 --
PECAS02624 --
DICDC02220 --
DICZE02284 --
DICD302017 --
DICD502250 --
XENBS00829 --
XENNA01480 --
PHOLL01537 --
PHOAA02804 --
SERP501669 --
SERSA01625 --
YERPE01271 --
YERPS01390 --
YERPA00627 --
YERPN02489 --
YERPP02190 --
YERP302544 --
YERPB01460 --
YERPY02633 --
YERPG01392 --
YERPD01171 --
YERP100890 --
YERPZ01206 --
YERPH02469 --
YERE801438 --
YERE302565 --
YERE100392 --
PROMH00687 --
PROSM03196 --
TOLAT02279 --
AERVB02262 --
AERHH01811 --
AERS402185 --
PSYIN02070 --
SHELP02018 --
SHEVD02297 --
SHEPW02341 --
SHEPA02202 --
SHEHH02007 --
SHESH02117 --
SHEWM02454 --
SHEAM01758 --

```
SHEON02159      ------------------------------------------------------------
SHESM01945      ------------------------------------------------------------
SHESR01991      ------------------------------------------------------------
SHESA02025      ------------------------------------------------------------
SHESW01953      ------------------------------------------------------------
SHEPC01976      ------------------------------------------------------------
SHEP201929      ------------------------------------------------------------
SHEB502016      ------------------------------------------------------------
SHEB802168      ------------------------------------------------------------
SHEB202117      ------------------------------------------------------------
SHEB902270      ------------------------------------------------------------
SHEB602258      ------------------------------------------------------------
SHEDO01680      ------------------------------------------------------------
SHEFN01895      ------------------------------------------------------------
PSEHT01677      ------------------------------------------------------------
ALISL01611      ------------------------------------------------------------
VIBF100893      ------------------------------------------------------------
VIBFM00891      ------------------------------------------------------------
OCESG01399      ------------------------------------------------------------
VIBA701872      ------------------------------------------------------------
VIBVY01451      ------------------------------------------------------------
VIBVU02545      ------------------------------------------------------------
VIBVM01748      ------------------------------------------------------------
VIBCH01157      ------------------------------------------------------------
VIBCM01114      ------------------------------------------------------------
VIBCJ02064      ------------------------------------------------------------
VIBC300725      ------------------------------------------------------------
FERBD01969      ------------------------------------------------------------
VIBFN02085      ------------------------------------------------------------
VIBTL01089      ------------------------------------------------------------
VIBPA01248      ------------------------------------------------------------
VIBAE02128      ------------------------------------------------------------
VIBCB00599      ------------------------------------------------------------
PANVC00701      ------------------------------------------------------------
SHIBC02443      ------------------------------------------------------------
ENTBF02792      ------------------------------------------------------------
KLEP700892      ------------------------------------------------------------
KLEPH01782      ------------------------------------------------------------
KLEP303551      ------------------------------------------------------------
KLEVT03420      ------------------------------------------------------------
ENTAK02985      ------------------------------------------------------------
KLEOK03151      ------------------------------------------------------------
SALAR01925      ------------------------------------------------------------
SALBC00812      ------------------------------------------------------------
SALPC00926      ------------------------------------------------------------
SALTI00868      ------------------------------------------------------------
SALCH00905      ------------------------------------------------------------
SALPA01706      ------------------------------------------------------------
SALTY00927      ------------------------------------------------------------
SALPK01786      ------------------------------------------------------------
SALHS00978      ------------------------------------------------------------
SALEP00854      ------------------------------------------------------------
SALDC00926      ------------------------------------------------------------
SALA400892      ------------------------------------------------------------
SALG200876      ------------------------------------------------------------
SALTS00888      ------------------------------------------------------------
SALT400905      ------------------------------------------------------------
SALPS01908      ------------------------------------------------------------
SALT101040      ------------------------------------------------------------
SALTD00960      ------------------------------------------------------------
SALPB02472      ------------------------------------------------------------
SALNS00928      ------------------------------------------------------------
SALSV01000      ------------------------------------------------------------
ECOS500812      ------------------------------------------------------------
ECOL600981      ------------------------------------------------------------
```

```
ECOL500883      ------------------------------------------------------------
ECOUT00876      ------------------------------------------------------------
ECOK100776      ------------------------------------------------------------
ECOSM02148      ------------------------------------------------------------
ECOLU01057      ------------------------------------------------------------
ECO7I02136      ------------------------------------------------------------
ECO8100828      ------------------------------------------------------------
ECO4500862      ------------------------------------------------------------
ECOAB00879      ------------------------------------------------------------
ECO4400961      ------------------------------------------------------------
ECOUM02550      ------------------------------------------------------------
ECOKI00869      ------------------------------------------------------------
ECOC100920      ------------------------------------------------------------
ECOC200920      ------------------------------------------------------------
ECO8N00803      ------------------------------------------------------------
ECO2700862      ------------------------------------------------------------
ECO2600974      ------------------------------------------------------------
ECOH100942      ------------------------------------------------------------
SHIB301848      ------------------------------------------------------------
ECOLI00846      ------------------------------------------------------------
ECO5700970      ------------------------------------------------------------
SHISS00756      ------------------------------------------------------------
SHIBS00679      ------------------------------------------------------------
SHIDS01857      ------------------------------------------------------------
ECO2400906      ------------------------------------------------------------
ECODH00784      ------------------------------------------------------------
ECOHS00922      ------------------------------------------------------------
ECOLC02627      ------------------------------------------------------------
ECO5E00946      ------------------------------------------------------------
ECOSE00936      ------------------------------------------------------------
ECO5500894      ------------------------------------------------------------
ECO8A00877      ------------------------------------------------------------
ECOBB00863      ------------------------------------------------------------
ECO5T00943      ------------------------------------------------------------
ECOBW00723      ------------------------------------------------------------
ECO1000919      ------------------------------------------------------------
ECOBD02604      ------------------------------------------------------------
ECOD102659      ------------------------------------------------------------
ECOBR00856      ------------------------------------------------------------
ECOLX02610      ------------------------------------------------------------
ECO1A00939      ------------------------------------------------------------
ECOCB01054      ------------------------------------------------------------
ECOKO02859      ------------------------------------------------------------
ECO1E03320      ------------------------------------------------------------
ECOLW01217      ------------------------------------------------------------
SHIFL01399      ------------------------------------------------------------
SHIF800759      ------------------------------------------------------------
SHIF200805      ------------------------------------------------------------
CITK802118      ------------------------------------------------------------
CITRI00924      ------------------------------------------------------------
CROS802385      ------------------------------------------------------------
CROTZ01500      ------------------------------------------------------------
ENTLS02865      ------------------------------------------------------------
ENT3801400      ------------------------------------------------------------
ENTAL01384      ------------------------------------------------------------
ENTCC02679      ------------------------------------------------------------


STRT101547      ---------
STRT201508      ---------
STRTD01356      ---------
STRTN01533      ---------
STRE500345      ---------
STRE801625      ---------
STREH01636      ---------
```

```
STREC01656      ---------
STREM01495      ---------
STRE401629      ---------
STRS700387      ---------
STRDG01631      ---------
STRP301388      ---------
STRPZ01205      ---------
STRPQ00464      ---------
STRPD01453      ---------
STRP601399      ---------
STRP801366      ---------
STRPF01453      ---------
STRPG00427      ---------
STRA300284      ---------
STRA500290      ---------
STRA100288      ---------
STRA200291      ---------
STRIC00348      ---------
STRPX00375      ---------
STRMD00432      ---------
STRS201734      ---------
STRSY01721      ---------
STRSX01553      ---------
STRSE01478      ---------
STREJ01635      ---------
STRGZ01544      ---------
STRS401593      ---------
LACGT00720      ---------
LACGL00738      ---------
STRSV01774      ---------
STRIJ00350      ---------
STROU01248      ---------
STRM601286      ---------
STRES00962      ---------
STRP701387      ---------
STRZT00756      ---------
STRP001079      ---------
STRZO01270      ---------
STRZ600815      ---------
STRET00826      ---------
STRPS01429      ---------
STRZN01287      ---------
STRR601306      ---------
STRP201217      ---------
STRZP01342      ---------
STRZI01226      ---------
STRPN01360      ---------
STRP401357      ---------
STRZJ01282      ---------
STRPJ01336      ---------
STRPI01436      ---------
MARHT00044      ---------
THEP300413      ---------
THEPX00884      ---------
THESX01857      ---------
THEM301788      ---------
THEIA01812      ---------
THETC00443      ---------
THESW01026      ---------
THEXL00360      ---------
THEID01127      ---------
THEOJ00147      ---------
DESAS01237      ---------
DESK701373      ---------
KYRT200729      ---------
```

```
STACT00410      ---------
STAS101952      ---------
STALH01969      ---------
STAEQ00422      ---------
STAES00543      ---------
STAAB00714      ---------
STAA500765      ---------
STAAM00757      ---------
STAAW00726      ---------
STAAS00733      ---------
STAAN00727      ---------
STAAC00807      ---------
STAA300727      ---------
STAA800734      ---------
STAA100752      ---------
STAA200774      ---------
STAA900759      ---------
STAAE00716      ---------
STAAT00767      ---------
STAAD00695      ---------
STAA000815      ---------
STAAH02407      ---------
STAAF00763      ---------
STAAK00744      ---------
STAAJ00703      ---------
STAAG00690      ---------
STAA400732      ---------
STAAR00788      ---------
LISSS02378      ---------
LISIN02590      ---------
LISW602421      ---------
EXISA00870      ---------
EXIS202358      ---------
EXIAB02180      ---------
OCEIH02462      ---------
BACIE01058      ---------
BACCJ03468      ---------
BACHD03507      ---------
BACPE03382      ---------
SOLSS00743      ---------
BACC600715      ---------
ANOFW02486      ---------
GEOKA03042      ---------
GEOSY02976      ---------
GEOTN02952      ---------
GEOSW02588      ---------
GEOS000381      ---------
GEOTC00378      ---------
BACMD04906      ---------
BACMQ04908      ---------
BACWK04830      ---------
BACAN04889      ---------
BACC105210      ---------
BACC705051      ---------
BACC005112      ---------
BACC305043      ---------
BACAC05232      ---------
BACAA04720      ---------
BACT005085      ---------
BACC205124      ---------
BACC405064      ---------
BACT104712      ---------
BACLD03769      ---------
BACPZ03376      ---------
BACSU03600      ---------
```

BACST01653	---------
BACPT03541	---------
LEUGG00661	---------
LEUGJ00629	---------
LEUCJ00485	---------
LACAR00657	---------
LACA300647	---------
LACAL00643	---------
LACKZ00964	---------
LACRJ00357	---------
LACRD00361	---------
LACRS01501	---------
LACSM00455	---------
LACRG00878	---------
LACRL00939	---------
LACC300844	---------
LACCZ00824	---------
LACCB00988	---------
LACCD01016	---------
LACCC01014	---------
LACBN01277	---------
LACBA00605	---------
LACPL00650	---------
LACPJ00628	---------
LACPS00585	---------
PEDCP00497	---------
CARS100359	---------
AERUA00263	---------
ELUMP00590	---------
SPHPG02940	---------
SPHGB01868	---------
TREPZ00273	---------
TREAZ03414	---------
SPITD00734	---------
SPITZ01373	---------
TREPA00803	---------
TREPS00802	---------
TREPC00747	---------
TREPM00823	---------
TREPD00824	---------
TREPU00781	---------
ENCCU00216	---------
HELM100964	---------
HELCP01490	---------
ARCFU01526	---------
FERPA02445	---------
ARCVS01910	---------
METEZ00677	---------
METHD00869	---------
METMA02304	---------
METAC01311	---------
KOSOT00298	---------
MARPK01613	---------
SLAHD02439	---------
FILAD00976	---------
BUTPB02463	---------
CLOPH00250	---------
CLOSW00592	---------
LACFC00207	---------
CRYCD00853	---------
EGGLE01847	---------
PYRFU01410	---------
PYRHO01476	---------
PYRAB00730	---------
PYRSN00014	---------

THEGJ00181	---------
THEKO02097	---------
THEON01610	---------
THES401476	---------
SYNWW02368	---------
UNCTG00012	---------
THEA101460	---------
DENA201647	---------
DEFDS00488	---------
CALNY01292	---------
SYNGF01652	---------
DESB201123	---------
DEIPM00845	---------
DEIRA01924	---------
DEIML01437	---------
DEIGD02532	---------
DEIDV00622	---------
TRURR01079	---------
THETG01863	---------
THET201543	---------
THET801911	---------
OCEP502115	---------
DEHLB00585	---------
DEHMG00439	---------
DEHMB00497	---------
DEHMC00427	---------
THELD00726	ARKFIG-IN
ANAMD00624	IEELI----
THEAS00815	LGLFLE-L-
AMICL00707	LSEFLK---
CLOCE01684	---------
HYDS000616	---------
HYDTT00224	---------
PELTS01405	---------
DESRL02179	---------
BORBP00506	---------
BORAP00518	---------
BORBU00514	---------
BORBZ00490	---------
BORBN00493	---------
BORRA00486	---------
BORDL00498	---------
BORHD00497	---------
BORT900497	---------
METKA01560	---------
MYCA500361	---------
METVS00145	---------
METOI01431	---------
MYCHN00051	---------
MYCSL00465	---------
MYCS300420	---------
DESK101258	---------
STAHD01472	---------
THEC100270	---------
NANEQ00478	---------
CALLD01225	---------
SULSO02155	---------
SULS900210	---------
SULIA00208	---------
SULIM00207	---------
SULIK00225	---------
IGNH400907	---------
KORCO01040	---------
THESM01133	---------
THEBM01534	---------

METST01363	---------
METTH00703	---------
METSL02406	---------
METLA02406	---------
METPW00194	---------
RUBXD00226	---------
RHOM400178	---------
GARV400051	---------
BIFAP01644	---------
SEGRD00014	---------
GORB404535	---------
GORPV04920	---------
MYCA904903	---------
MYCSS05356	---------
MYCSJ05684	---------
MYCSK05387	---------
MYCS206581	---------
MYCCN05158	---------
MYCVP05890	---------
MYCGI00775	---------
MYCSR04994	---------
AMYSD04545	---------
MYCLE02681	LSDVVPNLN
MYCLB02681	LSDVVPNLN
MYCSD04323	---------
MYCPA04306	---------
MYCA105023	---------
MYCUA04107	---------
MYCMM05378	---------
MYCA003905	---------
MYCTU03943	---------
MYCTF03846	---------
MYCTA03980	---------
MYCTK04010	---------
MYCTC03612	---------
MYCTD03548	---------
MYCCP03898	---------
MYCBO02863	---------
MYCBP03913	---------
MYCBT03913	---------
TSUPD04078	---------
ACTMD06897	---------
SACES08402	---------
SACVD03814	---------
AMYMU09190	---------
AMYMS10178	---------
PSEUX06421	---------
NOCFA05681	---------
NOCCG05476	---------
RHOE406010	---------
RHOOB03468	---------
RHOE104515	---------
CORDI02302	---------
CORD202219	---------
CORDL02208	---------
CORDJ02208	---------
CORDH02221	---------
CORD702316	---------
CORD302337	---------
CORDD02235	---------
CORDV02170	---------
CORDW02254	---------
CORDK02230	---------
COREF02870	---------
CORGL03082	---------

```
CORGK02974      ---------
CORGB03038      ---------
CORK402006      ---------
CORJK02028      ---------
CORVD02951      ---------
ARCHD01706      ---------
THET101205      ---------
PROAC02247      ---------
PROAS02302      ---------
CAERE29798      ---------
CELFA03749      ---------
JONDD02484      ---------
XYLCX03324      ---------
ACIC102144      ---------
FRADG04042      ---------
FRASU07065      ---------
FRASN07115      ---------
FRASC04424      ---------
FRAAA06712      ---------
KYTSD02521      ---------
KINRD04462      ---------
NOCDD04789      ---------
NOCAA01941      ---------
STRRD08913      ---------
THECD04863      ---------
THEBD03528      ---------
CATAD08894      ---------
KRIFD06917      ---------
KITSK03730      ---------
STRBB05366      ---------
STRVP03660      ---------
STRSW04381      ---------
STRGG03562      ---------
STRFA03098      ---------
STRAW04303      ---------
STRCO02929      ---------
STRHJ05105      ---------
MONBE04991      ---------
CHLRE01313      ---------
MEDTR25591      ---------
SOLLC13750      ---------
PRUPE10733      ---------
MANES18605      ---------
THECC00884      ---------
PHYPA31147      ---------
AMBTC19471      ---------
MUSAC26038      ---------
MUSAM33177      ---------
SETIT03079      ---------
ORYBR12195      ---------
COCLU07729      ---------
PHANO13702      ---------
PHAND10804      ---------
AURPU02089      ---------
ZYMTR07711      ---------
DICPU05926      ---------
ENTHI00522      ---------
LEPBA02231      ---------
LEPBP02301      ---------
LEPBL01462      ---------
LEPBJ01276      ---------
LEPIN02475      ---------
LEPII01996      ---------
LEPIC01426      ---------
SPIAZ00697      ---------
```

PENRW10140 ---------
PENCH09104 ---------
EURHE07269 ---------
ASPAC07301 ---------
EMENI10387 ---------
EMEND02596 ---------
ASPTN06742 ---------
ASPCL04014 ---------
ASPFU05647 ---------
NEOFI00452 ---------
CRYPA10563 ---------
BLUGR03498 ---------
SCLS112814 ---------
MAGGR04266 ---------
NEUCR01575 ---------
NEUT908941 ---------
VERDA02342 ---------
COLSU12486 ---------
HYPAI01684 ---------
HYPVG06080 ---------
HYPJE05895 ---------
NECHA05020 ---------
FUSO415847 ---------
GIBZA01026 ---------
SCHPO04025 ---------
YARLI03635 ---------
ASHGO00946 ---------
KLULA02190 ---------
ZYGRO00676 ---------
DEKBR01813 ---------
PICPG04776 ---------
CANTE00916 ---------
LODEL03891 ---------
DEBHA05546 ---------
SPAPN03477 ---------
CANAW04800 ---------
PICST04701 ---------
PUCGT10887 ---------
PUCGR11813 ---------
PHYBL11006 ---------
USTMA03757 ---------
USTHO04132 ---------
WALSE04527 ---------
TREME07701 ---------
AURST04751 ---------
FOMME10177 ---------
CONPW06392 ---------
STEHR07076 ---------
HETAN06295 ---------
GLOTR06982 ---------
PUNST01981 ---------
LACBI02877 ---------
COPCI16429 ---------
DICSQ11618 ---------
TRAVS13180 ---------
WOLCO03584 ---------
FOMPI05979 ---------
PHLGI10219 ---------
PHACH05757 ---------
RICTY00419 ---------
RICPR00429 ---------
RICPP00461 ---------
RICBR00434 ---------
RICB800988 ---------
RICCK00600 ---------

RICAH00614	---------
RICAC00782	---------
RICFE00656	---------
RICMS00042	---------
RICM500453	---------
RICR300665	---------
RICAG00651	---------
RICP300630	---------
RICRS00632	---------
RICRO00663	---------
RICCN00618	---------
RICPT00620	---------
RICAE00497	---------
RICJY00467	---------
RICPU00072	---------
RICS100537	---------
BARBK00891	---------
BARVW00932	---------
BART100617	---------
BARGA00466	---------
BARHE01107	---------
BARQU00895	---------
OCHA401642	---------
BRUAB01390	---------
BRUA201507	---------
BRUA101339	---------
BRUSU01458	---------
BRUME00510	---------
BRUSI01461	---------
BRUC201446	---------
BRUMC01440	---------
BRUMB01421	---------
BRUM501487	---------
BRUO201285	---------
RHILO01973	---------
CHESB02097	---------
METPB00996	---------
METEP01063	---------
METEA00810	---------
METED01453	---------
METS403554	---------
METNO05482	---------
METSZ03234	---------
BEII900056	---------
METSB02743	---------
MAGMM00401	---------
HYPNA00542	---------
KETVY00902	---------
KETVW00472	---------
ROSDO03242	---------
ROSLO02615	---------
RUEPO00888	---------
RUEST00613	---------
PHAIB02390	---------
PARDP02130	---------
DINSH02620	---------
RHOCB02768	---------
RHOS500248	---------
RHOS400150	---------
RHOS100227	---------
RHOSK02952	---------
MIDMI00790	---------
ACEP301595	---------
MICAA01566	---------
TISMK03676	---------

AZOL402409	---------
PSEUV04348	---------
HIRBI01095	---------
PARL102241	---------
MARMM02139	---------
PHEZH02588	---------
CAUCR02826	---------
CAUCN02939	---------
CAUST00891	---------
PARBH01522	---------
PELHB02084	---------
HYPDA03273	---------
HYPSM04642	---------
OLICO01107	---------
OLICM02790	---------
RHOPS01446	---------
RHOPA04062	---------
RHOPT04533	---------
RHOPX04235	---------
BRADU07321	---------
BRASO01239	---------
BRASB06352	---------
RHOPB03773	---------
NITWN02324	---------
NITHX02707	---------
AZOC501143	---------
XANP202679	---------
CHLTF01983	---------
IGNAJ02236	---------
MELRP00403	---------
ANADF00468	---------
CHLCH01125	---------
PELPB01419	---------
CHLL200781	---------
CHLTE00830	---------
CHLP800854	---------
CHLL701225	---------
CHLPM00993	---------
WOLTR00562	---------
WOLPP00135	---------
WOLPM00650	---------
WOLWR00416	---------
EHRCR00696	---------
ANAMM00345	---------
ANAMF00341	---------
ANAPZ00610	---------
NEOSM00537	---------
NEORI00513	---------
PELUB00076	---------
PELSM00736	---------
PUNMI01409	---------
ASTEC00651	---------
ZYMMT00154	---------
ZYMMO00984	---------
ZYMMA00176	---------
ZYMMN00183	---------
SPHAL00143	---------
SPHWW03978	---------
SPHJU02313	---------
NOVAD02319	---------
ERYLH00934	---------
GRABC00720	---------
GLUDA03075	---------
KOMMN00774	---------
HALVD01097	---------

```
HALHT01699      ---------
METI401038      ---------
PLAL201612      ---------
GEMAT01983      ---------
CYAAP02456      ---------
CHLPN00303      ---------
CHLPP00439      ---------
CHLPE00727      ---------
CHLTR00101      ---------
CHLTA00102      ---------
CHLTJ00101      ---------
CHLTD00098      ---------
CHLT700102      ---------
CHLT000101      ---------
CHLT500104      ---------
CHLTL00098      ---------
CHLTG00102      ---------
CHLTS00101      ---------
CHLT900102      ---------
CHLTZ00101      ---------
CHLT400432      ---------
CHLT100103      ---------
CHLT200350      ---------
CHLTB00350      ---------
CHLTC00378      ---------
SIMNZ00831      ---------
PARUW01706      ---------
WADCW00943      ---------
SINAD04980      ---------
BIFLB01558      ---------
BIFAB00498      ---------
BIFAV01561      ---------
BIFAS01557      ---------
BIFA001518      ---------
BIFBA01814      ---------
BIFAA01619      ---------
BIFDB02112      ---------
TERSS03091      ---------
GRATM03160      ---------
GRAMM03836      ---------
AKKM801247      ---------
OPITP04130      ---------
CORAD01794      ---------
BUCCC00180      ---------
CENSY00347      ---------
NITMS00668      ---------
MEIRD02823      ---------
HERA203780      ---------
CHLAA02018      ---------
CHLSY02175      ---------
CHLAD02599      ---------
CALAS01745      ---------
ANATU01696      ---------
SULMS00235      ---------
BLASB00041      ---------
BLASP00551      ---------
AZOPC00437      ---------
LEPBD01878      ---------
SALRD02577      ---------
SALRM02866      ---------
RIEPU00166      ---------
ORITB00015      ---------
ORITI01234      ---------
PREMB01541      ---------
PREDF01155      ---------
```

```
PREI702011        ---------
ALIFI01035        ---------
ODOSD00362        ---------
PRER201250        ---------
PALPW00206        ---------
PORGI00953        ---------
PORG301168        ---------
BACV803426        ---------
BACT601486        ---------
BACTN04290        ---------
BACFR01032        ---------
BACFN00913        ---------
BACF600963        ---------
OWEHD03413        ---------
PSYTT00390        ---------
NONDD00011        ---------
ROBBH02711        ---------
CELAD02592        ---------
CELLC02474        ---------
MARSH03072        ---------
MURRD00159        ---------
ZOBGA00152        ---------
GRAFK00949        ---------
ZUNPS02344        ---------
AEQSU02079        ---------
HALH105745        ---------
SAPGL02893        ---------
AMOA500014        ---------
FLAIG01802        ---------
FLACA02326        ---------
FLAJ100198        ---------
FLABF01528        ---------
CAPOD01333        ---------
CAPCC00414        ---------
FLELS01898        ---------
SOLCM01166        ---------
PEDHD01598        ---------
SPHS203207        ---------
LEAB401188        ---------
EMTOG00092        ---------
SPILD01737        ---------
DYAFD00084        ---------
CYCMS03063        ---------
ECHVK03326        ---------
BELBD02542        ---------
NITGG02112        ---------
THEM700111        ---------
CREAS01350        ---------
TURPD02772        ---------
SORC507603        ---------
BDEBA00337        ---------
STIAD02752        ---------
MYXXD01876        ---------
MYXFH03454        ---------
CORCM01960        ---------
MYXSD02209        ---------
LEPFC02126        ---------
LEPFM02294        ---------
SOLUE00582        ---------
KORVE01333        ---------
ACIC502553        ---------
ACIFD00030        ---------
HALMS01402        ---------
CHLPD01133        ---------
CHLPB01137        ---------
```

```
PROA200897      ---------
WIGBR00492      ---------
BUCA500289      ---------
BUCAI00292      ---------
BUCAF00306      ---------
BUCAT00287      ---------
BUCA000302      ---------
BAUCH00290      ---------
BLOVB00369      ---------
BLOFL00367      ---------
BLOPB00375      ---------
BUCAP00289      ---------
COXBU01001      ---------
COXBN01188      ---------
COXBR01117      ---------
COXB200717      ---------
COXB100914      ---------
FRAP200241      ---------
FRANT01012      ---------
FRACN00522      ---------
FRATT00465      ---------
FRAT100465      ---------
FRATE00462      ---------
FRATW01216      ---------
FRATM01110      ---------
FRACF00566      ---------
FRATO01212      ---------
FRATH01486      ---------
FRATF01216      ---------
FRATN00564      ---------
ACIF500512      ---------
ACIF200362      ---------
DECAR01284      ---------
NEIG100525      ---------
NEIG201314      ---------
NEIM800926      ---------
NEIMP01199      ---------
NEIMB01212      ---------
NEIMF01143      ---------
NEIML01119      ---------
NEIMM00780      ---------
NEIMH00827      ---------
NEIMG01168      ---------
NEIMN01254      ---------
NEIMO00810      ---------
NEIM701229      ---------
NEIMA01334      ---------
NEIMW01125      ---------
DICNV01037      ---------
VESOH00047      ---------
RUTMC00043      ---------
HALHL02255      ---------
PELPD03177      ---------
GEOS804030      ---------
GEOBB03636      ---------
GEOSM03700      ---------
HALNC00046      ---------
HAEPS00887      ---------
GALAU02285      ---------
HISS201000      ---------
HAES101151      ---------
PASMU00573      ---------
PASMH00579      ---------
ACTSZ01560      ---------
AGGAN00985      ---------
```

NITHN03181	---------
NITOC00311	---------
NITWC02498	---------
METNJ00648	---------
METFJ01616	---------
ALKEH00244	---------
MARMS03166	---------
MARM102688	---------
THICR00763	---------
THICA00974	---------
THIV600851	---------
THISH01973	---------
METAA00813	---------
META200813	---------
METMM02895	---------
FRAAD00813	---------
XYLFA01416	---------
XYLFT00623	---------
XYLF200667	---------
XYLFG01622	---------
XYLFM00690	---------
PSEUP01680	---------
STRM501972	---------
PSEUU01524	---------
XANAP01407	---------
XANCP01918	---------
XANC802154	---------
XANCB02263	---------
XANOR02379	---------
XANOM02295	---------
XANOP02318	---------
XANAC01951	---------
CYCSP01155	---------
GEOLS03275	---------
GEOUR03858	---------
GEODF00692	---------
GEOSL00482	---------
GEOSK00469	---------
GEOMG02998	---------
MORCR00228	---------
ACIAD00798	---------
ACIBC00821	---------
ACIBY02725	---------
ACIB302691	---------
ACIB500882	---------
ACIB100850	---------
ACIBD00843	---------
ACIBS02229	---------
ACICP00137	---------
ACISD03052	---------
LEGLN02402	---------
LEGPA01726	---------
LEGPH00846	---------
LEGPC01171	---------
LEGP201857	---------
LEGPL01727	---------
COLP302668	---------
KANKD01106	---------
IDILO00659	---------
PSEU901284	---------
PSEA602346	---------
ALTSS01871	---------
ALTMD01611	---------
ALTME01705	---------
ALTMB01797	---------

ALTMS01652	---------
SACD201683	---------
TERTT01748	---------
SIMAS00609	---------
ALCDB01962	---------
CHRSD02939	---------
HALED02811	---------
SIDLE02655	---------
GALCS01621	---------
LARHH01446	---------
CHRVO02807	---------
PSEUL01648	---------
NITEU01859	---------
NITEC00745	---------
NITMU00023	---------
NITSI03064	---------
ACCPU02128	---------
THIDA01000	---------
METS601221	---------
METGS01186	---------
METFK00972	---------
METML01428	---------
AROAE03899	---------
THASP01686	---------
AZOSB01359	---------
BORA102682	---------
BORPA03415	---------
BORBM03577	---------
BORPE02280	---------
BORPC02060	---------
BORP102392	---------
BORBR03870	---------
BORPD01524	---------
ACHXA01179	---------
RHOFT03114	---------
VEREI01701	---------
VARPE01375	---------
VARPS01266	---------
DELAS05272	---------
DELSC01235	---------
COMT200883	---------
ACIAC03267	---------
ACIAP03249	---------
ACIET02648	---------
ALIDK03528	---------
RUBGI03408	---------
LEPCP00749	---------
POLSJ03719	---------
POLNA03144	---------
METPP01110	---------
RAMTT03218	---------
RALPJ02457	---------
RALP102102	---------
RALSO02303	---------
RALS801097	---------
HERSS01878	---------
HERAR00955	---------
JANMA01152	---------
THIK102324	---------
POLSQ00682	---------
POLNS00989	---------
BURP800627	---------
BURPP00988	---------
BURSC00728	---------
BURXL00982	---------

BURSG00869	---------
BURRH00629	---------
BURGB00781	---------
BURGS00853	---------
BURPS02618	---------
BURMA01741	---------
BURP103016	---------
BURP002984	---------
BURM701866	---------
BURP602945	---------
BURM902465	---------
BURMS00740	---------
BURTA01515	---------
BURM102386	---------
BURL300890	---------
BURVG00860	---------
BURCM00829	---------
BURA400832	---------
BURCA00489	---------
BURCH00966	---------
BURCC00927	---------
BURCJ02922	---------
EDWI902348	---------
EDWTF01971	---------
EDWTE02176	---------
SODGM01094	---------
MOREP00394	---------
RAHSY01432	---------
RAHAC01419	---------
ERWBE01489	---------
PANAM01339	---------
PANAA00662	---------
PANSA01303	---------
ERWT902154	---------
ERWAC01330	---------
ERWAE01326	---------
ERWPE02241	---------
ERWP602414	---------
ERWSE02396	---------
PECCP01694	---------
PECWW01889	---------
PECSS01870	---------
PECAS02624	---------
DICDC02220	---------
DICZE02284	---------
DICD302017	---------
DICD502250	---------
XENBS00829	---------
XENNA01480	---------
PHOLL01537	---------
PHOAA02804	---------
SERP501669	---------
SERSA01625	---------
YERPE01271	---------
YERPS01390	---------
YERPA00627	---------
YERPN02489	---------
YERPP02190	---------
YERP302544	---------
YERPB01460	---------
YERPY02633	---------
YERPG01392	---------
YERPD01171	---------
YERP100890	---------
YERPZ01206	---------

```
YERPH02469      ---------
YERE801438      ---------
YERE302565      ---------
YERE100392      ---------
PROMH00687      ---------
PROSM03196      ---------
TOLAT02279      ---------
AERVB02262      ---------
AERHH01811      ---------
AERS402185      ---------
PSYIN02070      ---------
SHELP02018      ---------
SHEVD02297      ---------
SHEPW02341      ---------
SHEPA02202      ---------
SHEHH02007      ---------
SHESH02117      ---------
SHEWM02454      ---------
SHEAM01758      ---------
SHEON02159      ---------
SHESM01945      ---------
SHESR01991      ---------
SHESA02025      ---------
SHESW01953      ---------
SHEPC01976      ---------
SHEP201929      ---------
SHEB502016      ---------
SHEB802168      ---------
SHEB202117      ---------
SHEB902270      ---------
SHEB602258      ---------
SHEDO01680      ---------
SHEFN01895      ---------
PSEHT01677      ---------
ALISL01611      ---------
VIBF100893      ---------
VIBFM00891      ---------
OCESG01399      ---------
VIBA701872      ---------
VIBVY01451      ---------
VIBVU02545      ---------
VIBVM01748      ---------
VIBCH01157      ---------
VIBCM01114      ---------
VIBCJ02064      ---------
VIBC300725      ---------
FERBD01969      ---------
VIBFN02085      ---------
VIBTL01089      ---------
VIBPA01248      ---------
VIBAE02128      ---------
VIBCB00599      ---------
PANVC00701      ---------
SHIBC02443      ---------
ENTBF02792      ---------
KLEP700892      ---------
KLEPH01782      ---------
KLEP303551      ---------
KLEVT03420      ---------
ENTAK02985      ---------
KLEOK03151      ---------
SALAR01925      ---------
SALBC00812      ---------
SALPC00926      ---------
SALTI00868      ---------
```

```
SALCH00905      ---------
SALPA01706      ---------
SALTY00927      ---------
SALPK01786      ---------
SALHS00978      ---------
SALEP00854      ---------
SALDC00926      ---------
SALA400892      ---------
SALG200876      ---------
SALTS00888      ---------
SALT400905      ---------
SALPS01908      ---------
SALT101040      ---------
SALTD00960      ---------
SALPB02472      ---------
SALNS00928      ---------
SALSV01000      ---------
ECOS500812      ---------
ECOL600981      ---------
ECOL500883      ---------
ECOUT00876      ---------
ECOK100776      ---------
ECOSM02148      ---------
ECOLU01057      ---------
ECO7I02136      ---------
ECO8100828      ---------
ECO4500862      ---------
ECOAB00879      ---------
ECO4400961      ---------
ECOUM02550      ---------
ECOKI00869      ---------
ECOC100920      ---------
ECOC200920      ---------
ECO8N00803      ---------
ECO2700862      ---------
ECO2600974      ---------
ECOH100942      ---------
SHIB301848      ---------
ECOLI00846      ---------
ECO5700970      ---------
SHISS00756      ---------
SHIBS00679      ---------
SHIDS01857      ---------
ECO2400906      ---------
ECODH00784      ---------
ECOHS00922      ---------
ECOLC02627      ---------
ECO5E00946      ---------
ECOSE00936      ---------
ECO5500894      ---------
ECO8A00877      ---------
ECOBB00863      ---------
ECO5T00943      ---------
ECOBW00723      ---------
ECO1000919      ---------
ECOBD02604      ---------
ECOD102659      ---------
ECOBR00856      ---------
ECOLX02610      ---------
ECO1A00939      ---------
ECOCB01054      ---------
ECOKO02859      ---------
ECO1E03320      ---------
ECOLW01217      ---------
SHIFL01399      ---------
```

```
SHIF800759      ---------
SHIF200805      ---------
CITK802118      ---------
CITRI00924      ---------
CROS802385      ---------
CROTZ01500      ---------
ENTLS02865      ---------
ENT3801400      ---------
ENTAL01384      ---------
ENTCC02679      ---------
```
